# Proteomics of *Fusarium oxysporum* Race 1 and Race 4 Reveals Enzymes Involved in Carbohydrate Metabolism and Ion Transport That Might Play Important Roles in Banana Fusarium Wilt

**DOI:** 10.1371/journal.pone.0113818

**Published:** 2014-12-02

**Authors:** Yong Sun, Xiaoping Yi, Ming Peng, Huicai Zeng, Dan Wang, Bo Li, Zheng Tong, Lili Chang, Xiang Jin, Xuchu Wang

**Affiliations:** Key Laboratory of Tropical Crop Biotechnology, Ministry of Agriculture, Institute of Tropical Biosciences and Biotechnology, Chinese Academy of Tropical Agricultural Sciences, Haikou City, Hainan Province, China; Northeast Forestry University, China

## Abstract

Banana Fusarium wilt is a soil–spread fungal disease caused by *Fusarium oxysporum*. In China, the main virulence fungi in banana are *F. oxysporum* race 1 (F1, weak virulence) and race 4 (F4, strong virulence). To date, no proteomic analyses have compared the two races, but the difference in virulence between F1 and F4 might result from their differentially expressed proteins. Here we report the first comparative proteomics of F1 and F4 cultured under various conditions, and finally identify 99 protein species, which represent 59 unique proteins. These proteins are mainly involved in carbohydrate metabolism, post-translational modification, energy production, and inorganic ion transport. Bioinformatics analysis indicated that among the 46 proteins identified from F4 were several enzymes that might be important for virulence. Reverse transcription PCR analysis of the genes for 15 of the 56 proteins revealed that their transcriptional patterns were similar to their protein expression patterns. Taken together, these data suggest that proteins involved in carbohydrate metabolism and ion transport may be important in the pathogenesis of banana Fusarium wilt. Some enzymes such as catalase-peroxidase, galactosidase and chitinase might contribute to the strong virulence of F4. Overexpression or knockout of the genes for the F4-specific proteins will help us to further understand the molecular mechanism of *Fusarium*-induced banana wilt.

## Introduction

Banana is one of the most important tropical fruits worldwide [1]. Its production is heavily constrained by Fusarium wilt, a lethal disease in banana plants (*Musa* spp.) that is caused by *Fusarium oxysporum* f. sp. *Cubense* [2], [3]. Banana Fusarium wilt probably originated in Southeast Asia, but was first reported in Australia [2], [4]. Four physiological races of *F. oxysporum* have been reported to infect banana and eventually lead to Fusarium wilt of leaves [4]. However, only two races, *F. oxysporum* race 1 (F1) and race 4 (F4), have been shown to endanger banana production in China. F1 is a weakly virulent species that mainly infects the *Musa* Spp. Longyajiao and Fenjiao varieties. By contrast, F4 is strongly virulent and infects almost all banana varieties [2], [5]. The first internal symptom of Fusarium wilt is a reddish brown discoloration in the xylem that then spreads to feeder roots [6]. Vascular discoloration progresses to the rhizome, then joins the cortex, and ultimately proceeds up to large portions of the pseudostem. In severely affected 4–month–old plants, the older leaves turn yellow or split longitudinally, and then, the young leaves entirely collapse [2], [6].

Recent research has focused on characterization of the infection process of *F. oxysporum*, identification of pathotoxins and virulence genes in the fungus, and development of the fungal infection detection and monitoring methods, including chemical staining and fluorescent protein markers. Using GFP-tagged *F. oxysporum*, it was observed that the potential invasion sites in banana are the epidermal cells of root caps and elongation zones as well as wounded lateral root bases [7]. *F. oxysporum* produces several toxins, such as fusaric acid and beauvericin, during the growth and infection stages [8], [9]. Several of the virulence genes and transcription factors show to be related to Fusarium wilt are involved in signal transduction, defense responses, and colonization [10]–[14].

As a soil-borne disease, banana Fusarium wilt is difficult to control by traditional techniques. Many banana researchers therefore turn to chemical drugs or biological control strategies. Drugs have been developed that inhibit the growth of fungi or elicit resistance in the plant [2], and some fungi have shown antagonism against *F. oxysporum* [15]. However, the effects of these two control methods are often dose-dependent and the detailed molecular mechanism of pathogenesis remain unclear [2].

To further understand the host-pathogen interaction mechanisms, global changes in gene expressions upon pathogen invasion has been examined and some signaling components identified [16], [17]. However, quantitative mRNA data do not, necessarily, reflect the protein expression patterns. Conversely, protein abundance does not always correlate with mRNA level, particularly for certain low-abundance proteins. Moreover, many proteins undergo post-translational modifications that are important for their enzyme activity and subcellular localization [18]. Therefore, it is necessary to use proteome-based expression profiling to study plant–pathogen interactions. Recently, several fungal proteomics studies have analyzed the differentially expressed proteins upon fungal infection [3], [11], [16]. To our knowledge, no comparative proteomics study of the different races of *F. oxysporum* has been performed. Based on the borax/PVPP/phenol protocol [19] and improved Coomassie brilliant blue G-250 staining method [20], we optimized the protein extraction protocol for *F. oxysporum* and obtained high–quality two-dimensional electrophoresis (2-DE) profiles of F1 [3]. Here, we further performed comparative proteomics of F1 and F4 under different culture conditions and positively identified 99 differentially expressed proteins. These results provide insights that will be useful toward further investigations of the detailed molecular mechanisms of the pathogenesis of Fusarium wilt.

## Materials and Methods

### Culture conditions and growth analysis

The two races of *F. oxysporum* were isolated from banana leaves and mantained in our laboratory [3], [5]. Single spores from F1 and F4 were separated and grown on potato dextrose agar for one week at 27°C. A small piece of each plaque was transferred to the KK liquid medium (0.1% K_2_HPO_4_ w/v, 0.05% KCl w/v, 0.05% MgSO_4_ 7H_2_O w/v, 0.001% Fe-Na EDTA w/v, 0.2% aspartic acid w/v, 1% D –galactose w/v, pH 5.0) and cultured under in a shaking chamber at 27°C, and 120 rpm for additional two days. Both races were then examined by light microscopy. Similarly, the growth and dry weight content of the two races at different time point were determined as described [5]. For each sample, 10 biological replicates were examined and the results shown are the mean ± standard deviation (n = 10). Finally, ∼1 mL of the above KK liquid medium containing F1 or F4 was added to 200 mL of fresh KK liquid medium, and the cultures were shaken at 27°C, and 120 rpm for an additional 2, 4, or 6 days. The cultures were centrifuged at 15,000×*g* for 15 min at 4°C, and the bottom fraction containing mycelia was washed twice with KK liquid and partially dried by filtration through three sheets of 1 mm Whatman No. 2 filter paper in a Buchner funnel. The samples were immediately frozen and ground in liquid nitrogen, and the ground fungal mycelia were stored at −80°C for proteomic analysis.

### Protein extraction and quantification

The frozen collected mycelia were added to protein extraction buffer at a ratio of 1∶3 (w/v) to isolate proteins by the borax/PVPP/phenol protocol [19] with minor modifications as described [3]. The air-dried protein pellets were dissolved in the lysis buffer (7 M urea, 2 M thiourea, 2% CHAPS, 13 mM dithiothreitol), and protein concentrations were determined by Bradford assay using a spectrophotometer (Shimadzu UV-160, Kyoto, Japan). Bovine serum albumin was used as standard.

2-DE and gel analysis

Approximately 1200 µg of protein was used to perform 2-DE with 24 cm, pH 4–7 linear gradient IPG strips (GE Healthcare, Uppsala, Sweden) as described [3], [19]. The gels were visualized with the by Coomassie brilliant blue G-250, ammonium sulfate and phosphoric acid (GAP) staining method as described [20]. Gel images were statistically analyzed using ImageMaster 2D Platinum software (Version 5.0,GE Healthcare). Three representative gels from three corresponding biological replicates (n = 3) were used to determine the differential protein spots. The protein spots with p-value<0.05 and relative volume ratio ≥2.0 or ≤0.5 (two fold change) were deemed differential.

### Protein identification via mass spectrometry

The differential protein spots were identified by matrix-assisted laser desorption/ionization time-of-flight/time-of-flight mass spectrometry (MALDI TOF/TOF MS) as described [21]. Briefly, the differential protein spots were manually excised, in-gel digested with trypsin, then dried in a vacuum. Mass spectra were obtained on an Ultraflex II MALDI-TOF/TOF Mass Spectrometer (Bruker Daltonics, Bremen, Germany). The spectra were analyzed with the flexAnalysis software (Version 3.2, Bruker-Daltonics, USA) and searched against the taxonomy of fungi (1,941,581 sequences) in the non-redundant NCBI database (NCBInr 20130706, including 26,682,258 sequences and 9,281,362,451 residues) using Mascot software (Version 2.3) as described [21]. The peptide mass fingerprint (PMF) search parameters were: 300 ppm tolerance as the maximum mass error, MH^+^ monoisotopic mass values, allowance of oxidation (M) modifications, allowance for one missed cleavage, trypsin as the enzyme, and fixed modification of cysteine by carboxymethylation (carbamidomethylation, C). In addition, an tandem MS(MS/MS) ion search in the LIFT mode was performed under the above search conditions except with a ion tolerance of ±0.3 Da. Good matches were classified as those having a threshold score (confidence intervals >95%) >75 for PMF and >45 for MS/MS. The identification focused on the protein spots with higher Mascot scores, maximum peptide coverage, matched peptide sequences from different species, particular position on the 2-DE gel, and with identification results obtained from both PMF- and MS/MS-based searches. Additionally, a Decoy database search was also performed to estimate the percentage of false-positive identifications for all samples. Protein identifications determined to be false with the decoy database were excluded from further study. Detailed information about the protein identification is provided in Data S1-S3. In addition, an in-house Blast-P search at NCBI (http://www.ncbi.nlm.) was performed to confirm all of the identifications and find their homologous proteins.

### Functional analysis of the identified proteins

The identified proteins were further searched against UniProt (http://www.ebi.uniprot.org) to confirm their functions. These proteins were further divided into different groups by the Functional Catalogue software (http://mips.gsf.de/projects/funcat) to obtain their corresponding COG (Cluster of Orthologous Groups of proteins) codes. Then, gene ontology (GO) pathway analysis was performed with Blast2-GO software (http://blast2go.bioinfo/). Functional classifications of the identified proteins from the different races are provided in Data S2–S4.

### Reverse transcription PCR analysis

Total RNA was isolated from the same F1 and F4 samples used in the proteomic analysis. cDNA was generated using a reverse transcriptase kit (TaKaRa, Tokyo, Japan). Reverse transcription (RT)-PCR reactions were repeated 4 times for each biological replicate. The actin gene (NCBI No. JQ965663) of *F. oxysporum* was used as an internal control to normalize the template cDNA. The primers used are provided in Data S5.

### Statistical analysis

The statistical results are presented as the mean ± standard deviation. Statistical analyses, including two-way analysis of variance, Duncan's multiple range tests, and multi-variant analysis were performed with a 5% level of significance using SPSS software (Version 12.0).

## Results

### Growth patterns and protein 2-DE profiles of F1 and F4

**Figure 1:**
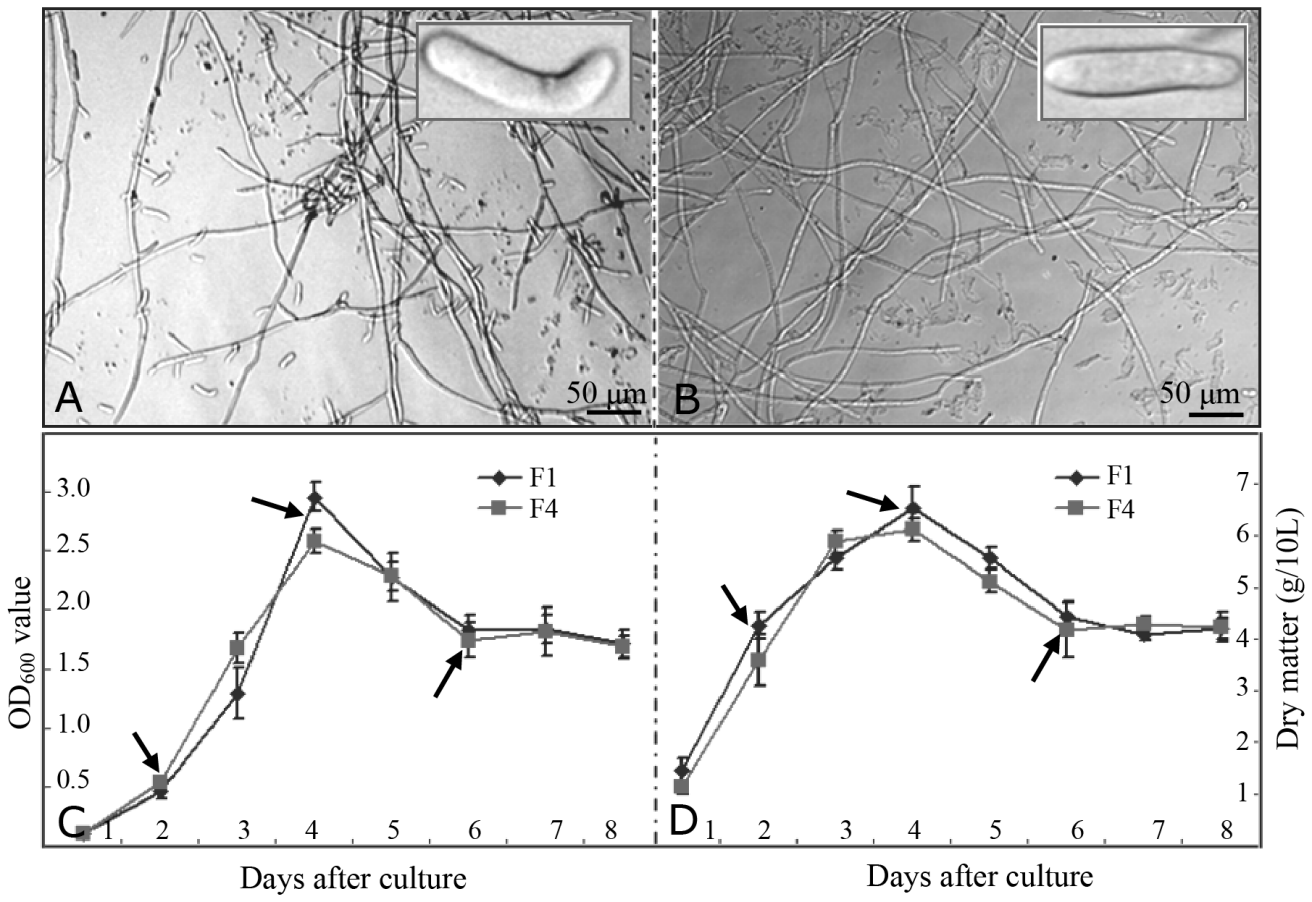
Morphological observation and comparison of growth patterns for different *Fusarium* races. Under light microscopy, both mycelia and spores from 2-day-cultured F1 (A) and F4 (B) showed similar morphology patterns. The OD_600_ values (C) indicating the mycelium concentration and dry matter content (D), were similar in the two race during the 8 days of culture. The data represent the means ± standard deviation (n = 10). Arrows indicate the time points (2, 4, and 6 days) at which the mycelia and spores were collected for further study.

To compare the growth patterns of races F1 and F4, 2-day liquid cultures were examined by light microscopy. Both mycelia and spores from F1 (Figure 1A) and F4 (Figure 1B) demonstrated similar morphology, which is consistent with our previous report that F1 and F4 had similar morphology when grown on potato dextrose agar medium [3]. In a KK liquid medium, F1 and F4 showed similar growth curves (Figure 1C) and similar changes in dry weight content from day 2–8 (Figure 1D). The mycelium concentration increased sharply after being cultured for 2 days, peaked at 4 days, and decreased at 6 days. After culturing for more than 6 days, the mycelium concentration as well as the dry weight content plateaued to some extent (Figure 1C).

**Figure 2:**
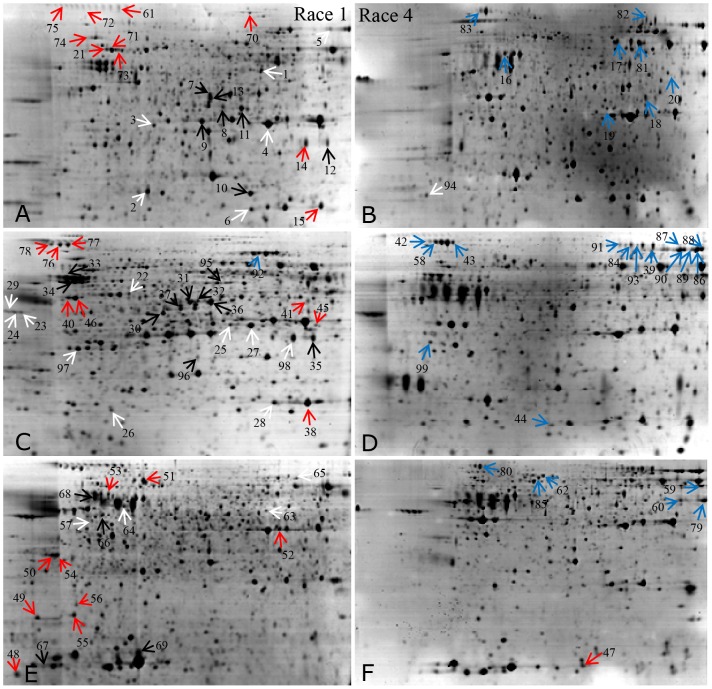
Representative 2-DE profiles of proteins extracted from F1 and F4. The typical 2-DE profiles of proteins extracted from mycelia of F1 cultured for 2 days (A), F4 cultured for 2 days (B), F1 cultured for 4 days (C), F4 cultured for 4 days (D), F1 cultured for 6 days (E), and F4 cultured for 6 days (F). The 99 differentially expressed proteins are marked with arrows and numbers, white arrows represent proteins up-regulated in F1, red arrows represent proteins specific expressed in F1, black arrows represent proteins up-regulated in F4, blue arrows represent proteins specific expressed in F4.

Therefore, we carried out 2-DE for F1 and F4 proteins isolated from mycelia after culturing for 2, 4 and 6 days. The representative gels for both races at the different times are presented in Figures 2A–E. More than 900 protein spots were reproducibly detected on each GAP-stained gel. Some of these spots showed quantitative or qualitative changes over time. Both F1 and F4 showed some changes in their 2-DE profiles over time, and the differences were more obvious at different days (Figure 2). Quantitative image analysis demonstrated that 136 protein spots were significantly changed in the two races (p<0.05) by> two-fold for at least one time point. Compared with F1, there were 24 up-regulated spots, 23 down-regulated spots, and 28 race-specific spots observed in F4 (Data S2).

### Identification of the differentially expressed proteins between F1 and F4

In this study, 99 of the 136 differentially expressed protein spots were positively identified by either PMF or MS/MS (Figure 2; Data S1), including 59 unique proteins. The PMF spectra, peptide sequences, and Mascot searched results are listed in detail in the Data S1–S3. A few spots were identified as being the same protein or representing isoforms. For example, seven spots (spots 5, 86–90, and 92) were identified as catalase-peroxidase, four as ATP synthase (spots 38, 40, 46, and 49), four as phosphoglycerate kinase (spots 7, 13, 31, and 36), four as hexosaminidase (spots 12, 14, 35, and 98), four as galactosidase (spots 42, 43, 80, and 83), and four as l-amino acid oxidase (spots 39, 82, 84, and 91). Many highly abundant proteins, such as Mn-superoxide dismutase (spots 6, 15, and 28), NADP-dependent glycerol dehydrogenase (spots 4, 25, and 27), vacuolar protease A (spots 23, 24, and 29), and phospholipase B (spots 76–78) also had several protein isoforms (Figure 2; Data S6).

To evaluate the MS identification quality, the theoretical and experimental ratios of molecular weight (M_r_) and isoelectric point were determined. These obtained values are presented as radar axis labels (M_r_ ratio for radial value; isoelectric point ratio for annular value) in the radial chart in Data S2. In theory, if the identified proteins are the same ones from the same species, both the radial and annular values will be 1.0, and all the calculated spots will be located on the cyclical line 1.0 in radial chart. The more spots were observed near line 1.0, the higher quality the MS identifications obtained. We found that >95% of spots were located close to the cyclical line 1.0, indicating the good quality of MS identification for these proteins (Data S2).

**Figure 3:**
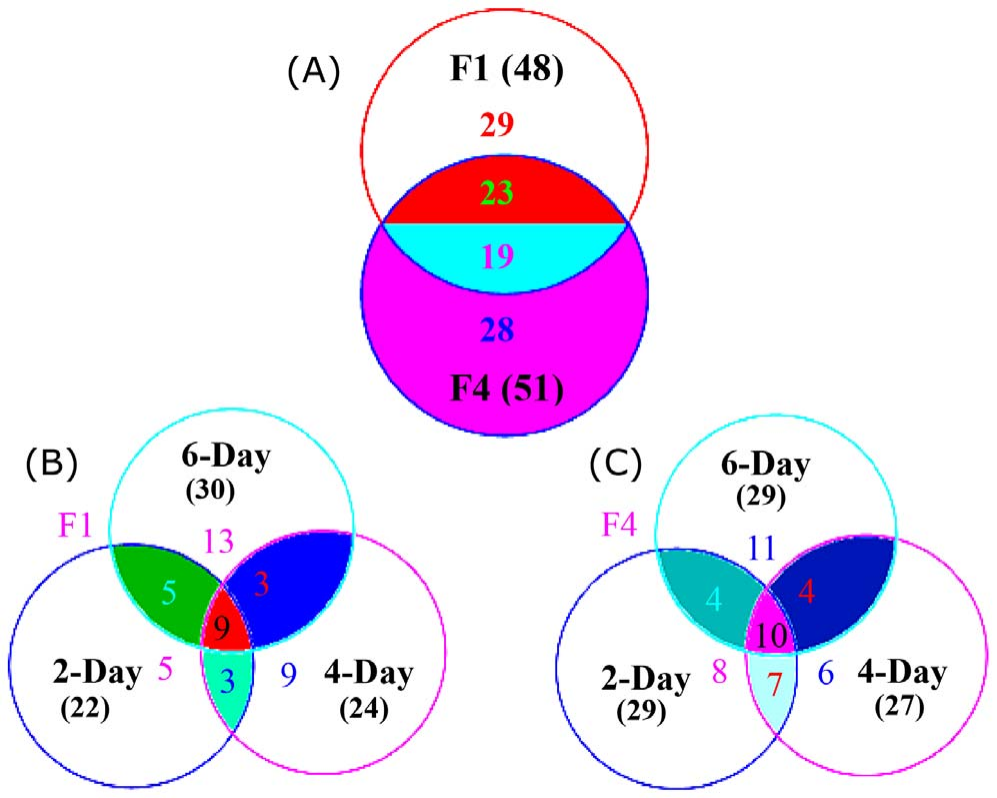
Venn diagrams analysis of the identified proteins from F1 and F4 under different cultured conditions. The identified proteins are presented according to their sources *Fusarium* races (A) or according to F1 (B) and F4 (C) cultured for different number of days. Overlap areas are marked with different colors, and the numbers of protein spots in each cluster are indicated.

Venn diagram analysis revealed that 48 and 51 proteins were respectively identified from F1 and F4 (Figure 3). Among them, 29 proteins were specifically observed only in F1, and 28 proteins were specific to F4. Among the 42 shared proteins, 23 proteins were up-regulated in F4, whereas the other 19 proteins were more abundant in F1 (Figure 3A). For F1, 22, 25, and 30 proteins were respectively identified in the 2-, 4-, and 6-day culture samples (Figure 3B). For F4, 29, 27, and 29 proteins were respectively obtained in the 2-, 4- and 6-day culture mycelia (Figure 3C). Among the F1-specific proteins, three spots were detected only in the 2- and 4- days, five spots at only days 2 and 6, three spots at only days 4 and 6, and nine spots were detected on all three days. For F4, four proteins were specific to day 2 and 6, four proteins to days 2 and 6, 4 proteins at 4 and 6 days, and ten proteins were observed on all three days (Figure 3; Data S2).

To gain insight into the functions of these proteins, we further searched for their homologues with BLAST-P using the protein sequences as queries and/or searched the identified NCBI accession numbers against the UniProt database. A substantial number of spots were identified as protein isoforms or protein homologs, indicating that post-translation modifications (PTMs) are of significant importance for *F. oxysporum*. Further research may focus on uncovering the detailed functions of the isomeric forms and PTMs such as oxidation and phosphorylation.

### Compiled expression profiles and functional classification of the differential proteins between F1 and F4

**Figure 4:**
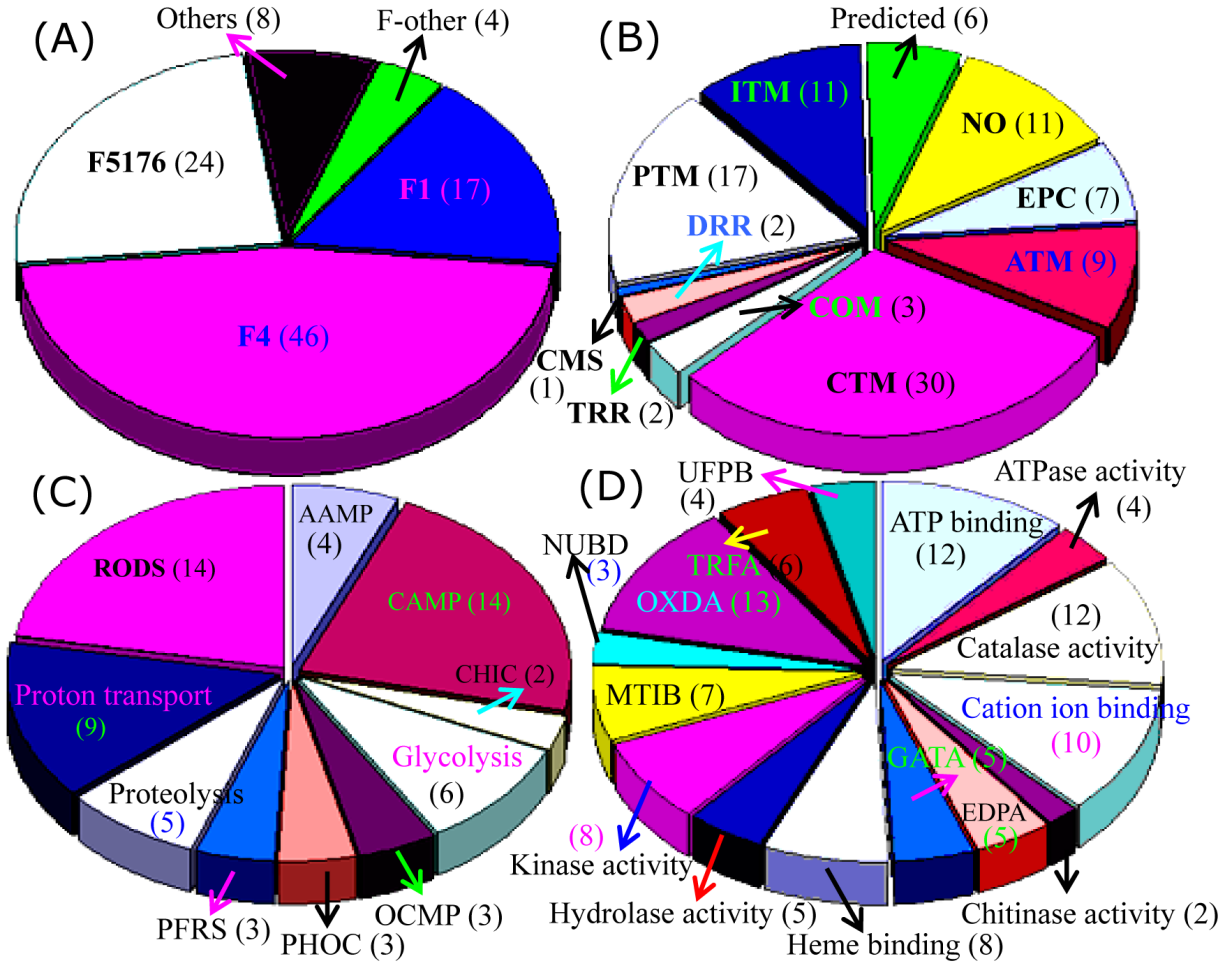
Classification and functional analysis of the identified proteins. Distributions of the 99 identified proteins in different species were determined (A). The COG classifications of identified proteins are provided (B). GO analysis was performed based on biological process (C) and molecular function (D). The numbers of protein spots in each category is presented in parentheses. The COG and GO category abbreviations are defined in Data S2.

We next examined the distribution of the 99 identified proteins in different fungal species and found that 46 of the proteins from F4 and 17 from F1 could be identified. On the other hand, 24 of the proteins were also found in *F. oxysporum* Fo5176, four proteins were seen in other *Fusarium* varieties, and eight proteins showed high similarity to proteins in other fungal species (Figure 4A; Data S1). The 99 proteins were further classified into 11 groups based on their main cellular functions (Figure 4B). Among the 82 proteins to which functions could be assigned, the largest group were involved in carbohydrate transport and metabolism (CTM), followed by the post–translational modification (PTM)-related chaperones, inorganic ion transport and metabolism (ITM)-related proteins, amino acid metabolism (ATM) and energy production and conversion (EPC). A relatively small proportion of the proteins is involved in coenzyme metabolism (COM), DNA replication, recombination and repair (DRR), transcription, translation, ribosome structure and biogenesis (TRR), and cell motility and secretion (CMS). Function could not be assigned to the remaining 17 proteins (Figure 4B; Data S1).

GO biological process analysis of the 99 proteins revealed that most proteins are involved in antioxidation processes such as response to oxidative stress, superoxide metabolism, and cell redox homeostasis. Another large portion was involved in carbohydrate metabolism. Followed proton transport coupled with ATP synthesis or ATP hydrolysis, glycolysis, and proteolysis (Figure 4C). GO molecular function analysis of the 99 proteins revealed that the largest portion was showed strong oxidoreductase activity (OXDA) (Data S2). Another large portion has ATP-binding activities, catalase activities, cation ion-binding activities, and kinase activities. Several of proteins were predicted to enzymes with endopeptidase activity, galactosidase activity, hydrolase activity, and protein binding (Figure 4D). At the GO-cellular component level, 88 of the 99 identified proteins showed no detailed locations. Five proteins deemed to be involved in proton transport, and several enzymes were predicted to be integrated into phosphopyruvate hydratase complex, eukaryotic translation elongation factor complexes, or to be located in the endoplasmic reticulum (Data S2).

The subcellular locations of the 99 proteins were further predicated by TargetP, which demonstrated that 37 proteins function in the secretory pathway, suggesting that secreted proteins are important for banana wilt. The predominant proportion fell into the “other” location category or could not be assigned to a location, which is similar to what was observed in the GO-cellular component classifications (Data S2–S4).

### Subcellular location and functional analysis of F4-specific proteins

As our primary interest in this study was to identify virulence-related proteins, we performed further in-depth analysis of the 46 proteins form F4 (Data S1 and S2). Several of these proteins had more than three isoforms, including catalase-peroxidase, galactosidase, glucanosyltransferase, phosphoglycerate kinase and ATP synthase. TargetP analysis predicted that 18 proteins are in the secretary pathway, 5 localize to mitochondria, 14 are in “other” locations and 10 have no known locations. Compared to TargetP, GO-cellular component analysis produced very limited location information (Data S2).

**Figure 5:**
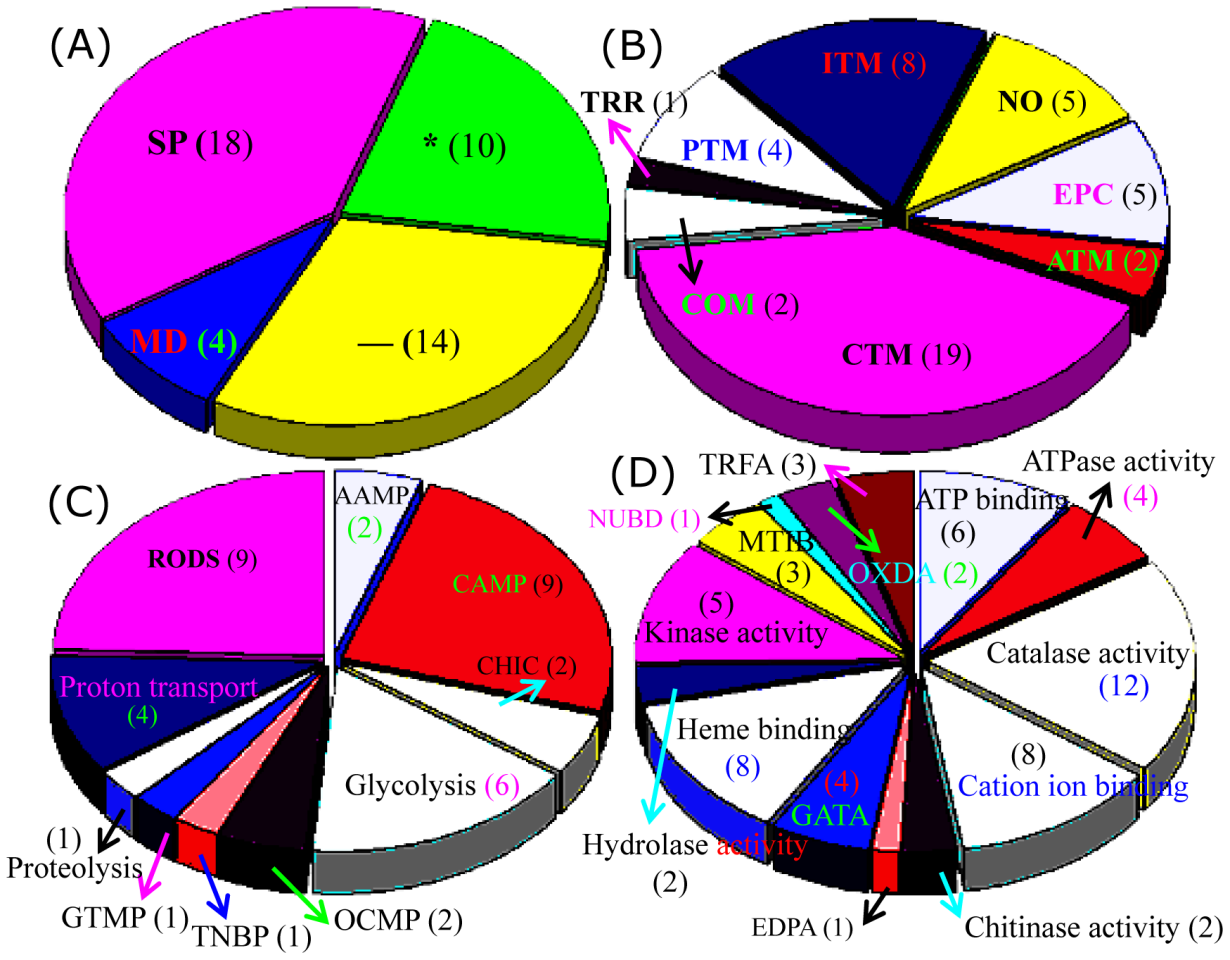
Subcellular location and functional classification of F4 proteins. The 46 proteins from F4 were classified according to subcellular locations predicted by TargetP (A), COG functional classification (B), GO biological process (C), and GO molecular function (D). The numbers of the protein spots in each category is presented in parentheses. COG and GO category abbreviations are defined in Data S2.

Functional analysis by COG revealed that approximately half of the F4-specific proteins are involved in carbohydrate transport and metabolism, including glucanosyltransferase, galactosidase, phosphoglycerate kinase, enolase, and chitinase. Many F4 proteins are also involved in inorganic ion transport and metabolism (8 isoforms of catalase-peroxidase), energy production and conversion (4 isoforms of ATP synthase and one glutathione reductase), and post–translational modification related chaperones (Figure 5B). Molecular function analysis revealed that most of these proteins such as catalase-peroxidase and glucanosyltransferase, have catalytic activity, followed by cation ion binding (glucanosyltransferase, chitinase and enolase), heme binding (catalase-peroxidase), and kinase activity (phosphoglycerate kinase and adenosine kinase). Another six proteins were identified as enzymes involved in ATP binding or hydrogen ion transporting ATP synthase activity. The results obtained from both the GO analysis and COG classification of the 99 differential proteins revealed that enzymes related to carbohydrate metabolism, ion transporting and energy production might play important role in the F4 induced banana wilt.

### Comparison of the gene and protein expressions between F1 and F4

Comparison of the transcript accumulation and protein abundance in F1 and F4 can broaden our understanding of the roles of particular proteins on the induction of banana Fusarium wilt. To further evaluate the correlation between the abundance of the identified proteins and the expression of their corresponding genes, mRNAs encoding 15 proteins were selected for RT-PCR analysis. This subset included 13 F4-specific proteins or proteins that were more abundant in F4: galactosidase (spot 80), chitinase 1 (spot 79), glutathione reductase (spot 20), oxidoreductase (spot 62), glutamine amidotransferase (spot 2), adenosine kinase (spot 3), vacuolar protease A (spot 24), amidase (spot 81), l-amino acid oxidase (spot 82), serine carboxypeptidase (spot 85), serine carboxypeptidase (spot 17), homoserine dehydrogenase (spot 19) and NADP-dependent glycerol dehydrogenase (spot 4). Two F1 abundant proteins named phosphoglycerate kinase (spot 7), and mitochondrial peroxiredoxin (spot 10) were also selected.

**Figure 6:**
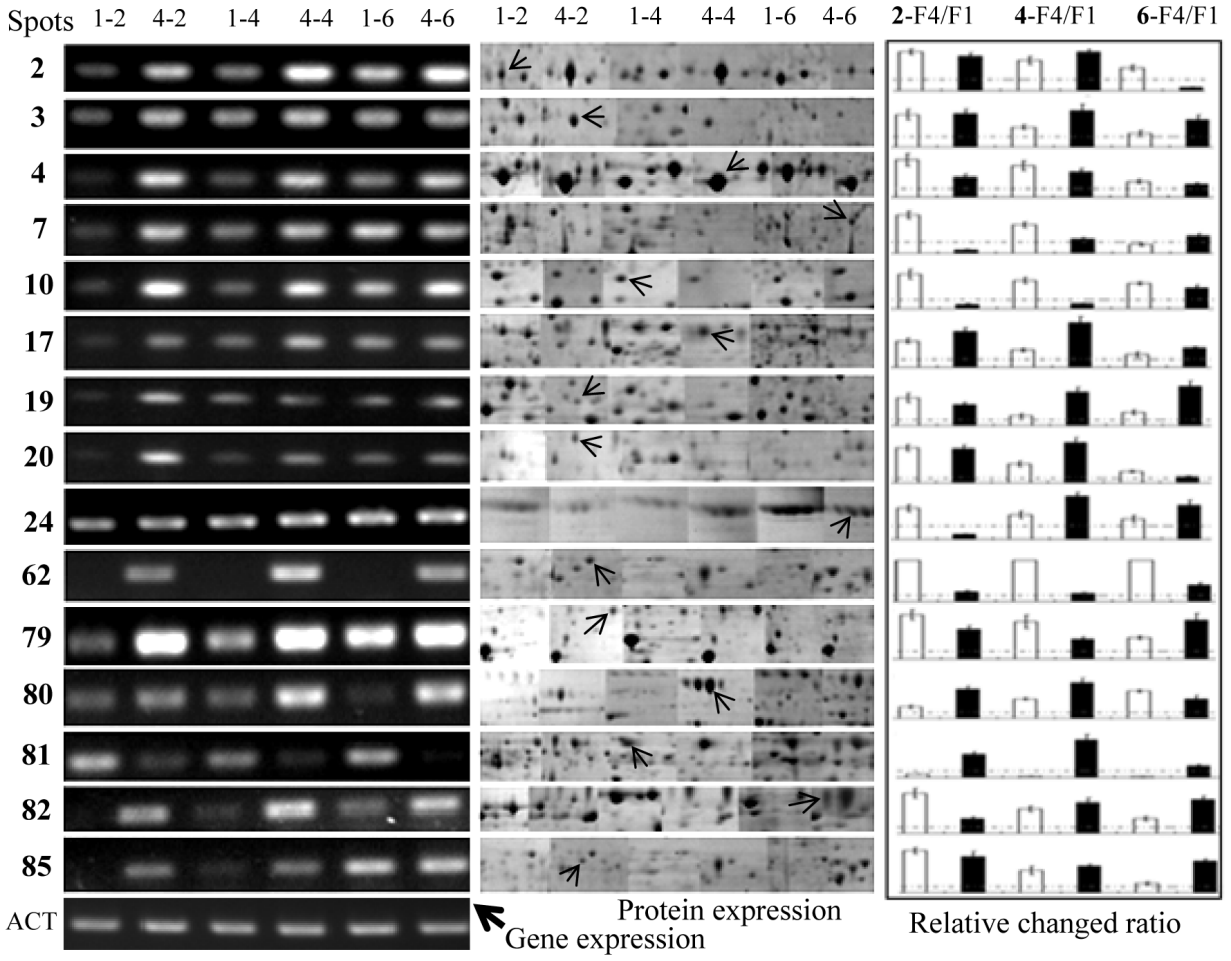
Comparison of gene expression and protein abundance between F1 and F4. To evaluate the correlation between protein and gene expressions in the two *Fusarium* races, mRNAs encoding 15 proteins were selected for analysis by RT-PCR (left) compared with 2-DE (middle) at the same time point. Actin (ACT) gene was used as a reference. Labels at the top denote, for example: 1-2, F1 cultured for 2 days; 4-4, F4 cultured for 4 days. The relative ratios of F4 to F1 mRNA (white rectangles) and protein (black rectangles) are also showed (right). Values represent the mean ± standard deviation of 4 independent replicates.

The accumulation patterns of proteins and their transcripts were similar for all the selected proteins except for amidase (Figure 6; Data S5). After culturing for 2 or 4 days, most of the selected genes showed higher expression in F4 than in F1. At day 6, both proteins and mRNA levels were reduced to some extent (Figure 6). Taken together, functional analysis of the F4-specific proteins revealed that some enzymes, including catalase-peroxidase, galactosidase, glucanosyltransferase, phosphoglycerate kinase, mitochondrial ATP synthase, glutathione reductase, adenosine kinase, S-adenosylmethionine synthetase, enolase, and chitinase, are involved in a variety of metabolic pathways and biological processes in F4.

## Discussion

### Comparative proteomics highlights some differentially-expressed signal proteins between F1 and F4

It is assumed that the pathogenicity of fungi is controlled by multiple proteins. Hence identification of the virulence-related proteins is of great significant importance for uncovering the pathogenic mechanisms. It is also likely that proteins differentially represented in F1 and F4 account for the different pathogenicity of the two races. The virulence proteins that have been characterized from F1 and F4 are involved in signal transduction, transcription, defense system, and colonization [2]. They include G-proteins [7], mitogen-activated protein kinase [22], signal transcription factor [13], [23], glucosidase [23], polygalacturonase [24], and mitochondrial carrier protein [25]. Proteomics studies in *Fusarium graminearum* [26], [27] and *F. oxysporum* [28], [29] identified 174 cell wall proteins and 19 proteins carrying predicted signal peptides in *F. oxysporum*, including glucose-regulated protein, acid phosphatase, cell wall glucanosyltransferase, glucanase, secreted aspartic protease, and vacuolar protease [28]. These studies also identified 197 proteins in *F. oxysporum* MSA 35 that were involve in virulence and/or antagonism other fungi, and some of which may affect the fitness of defense mechanisms [29].

In this research, we found 37 proteins containing predicted signal peptides such as catalase-peroxidase 2, superoxide dismutase, l-amino acid oxidase, carboxy-muconate cyclase and galactosidase. Therefore, we inferred that, during the infection process, proteins with signal peptides in *F. oxysporum* might be secreted into the plant extracellular space, which eventually causes the banana plant wilt. We also obtained some proteins that are involved in virulence, antagonism other fungi, and defense, including serine carboxypeptidase, superoxide dismutase, disulphide isomerase, and lactonohydrolase. Some of these proteins were predicted and/or reported to function as virulence factors (Data S1 and S2), such as peptidase C1 [30], carboxypeptidase [31], [32], glucanosyltransferase [33], [34], S-adenosylmethionine synthetase [35], peroxiredoxin [36], chitinase 1 [37], catalase-peroxidase [38], and amidase [39]. These results indicated that signal peptides–containing proteins and virulence factors might be important contributors to the strong virulence seen in Fusarium-induced banana wilt.

### In-depth proteomics uncovers some potential virulence enzymes in F4

To investigate the strong pathogenicity of F4, Li *et al.* isolated the mutant gene *foABC1* from F4. This gene has 48% sequence similarly with the rice homology and encodes an ABC transporter responsible for pumping toxin out of the fungal cell during the pathogenic process [17]. Chen *et al.* cloned *esyn1* gene from F4, which encodes a transcription factor important for regulation of biosynthesis of enniatin and beauvericin [40]. Our proteomic data demonstrate that almost all of the identified F4 proteins (40 of 46) are functional enzymes (Data S1). One of these enzymes, the cell wall–anchored glycoside hydrolase β-1-3-glucanosyltransferase, can directly affect fungal physiology [41]. Inactivation of a glucanosyltransferase in *F. oxysporum* results in a reduced virulence in tomato [13], [14], whereas deletion of a similar gene in the maize rot fungus of *Fusarium. verticillioides* affects conidiation and cell wall structure but not virulence [42]. The glucanosyltransferase gene *Bbgas1* knockout mutant shows reduced virulence in *Galleria mellonella* [33]. Also in *Aspergillus fumigatus,* mutation of a glucanosyltransferase gene resulted in slow growth, abnormal conidiogenesis, and low virulence [34].

S-adenosylmethionine (SAM) synthetase catalyzes the joining of methionine and ATP to produce the ubiquitous methyl group donor SAM. Overexpression of *samA* improves the development and secondary metabolism of the filamentous fungus *Aspergillus nidulans* [35]. In the budding yeast *Schizosaccharomyces pombe*, low expression of *sam1* reduces the growth, mating, and sporulation, whereas *sam1* overexpression leads to the methionine sensitive-growth [43]. Overaccumulation of SAM synthase in the bacterium *Bacillus subtilis* results in increased spontaneous sporulation [44]. Low abundance of SAM synthase in *Escherichia coli* blocks filament formation in the septal ring [45]. FBA, a class II family aldolase, is widely detected in organisms from fungi to prokaryotes, and the homodimer catalyzes the aldol cleavage of fructose bisphosphate using a zinc ion. In *Candida albicans*, mutation of the FBA gene perturbs growth but is not lethal for this fungus [46]. In *E. coli*, FBA is the primary enzyme that counters of nickel toxicity [47]. Among the potential virulence enzymes, phosphoglycerate kinase is widely considered as a key enzyme in the reversible transfer of a phosphate from 1,3-bisphosphoglycerate to ADP, thereby resulting in the formation of 3-phosphoglycerate and ATP in glycolysis [48].

The upregulation or specific expression of some enzymes in F4 might play a key role in its strong pathogenicity. Among the F4-specific proteins, amidase likely helps this fungus to efficiently adhere to banana root, a key first event in infection by pathogenic microorganism. Autolysin amidase in *Listeria monocytogenes* promotes an efficient listerial adherence to mouse hepatocyte, and thus listerial pathogenicity [39]. This process depends on the activation of glycosaminoglycan in *L. monocytogenes* [39]. Glycosaminoglycan, as a main extracellular matrix component, plays important roles in cell communication through binding to several proteins [49]. Similar to glycans, sulfur is also a vital element for all living organisms. Once sulfide is synthesized, it is condensed with *O*-acetyl-L-serine under the catalytic action of serine lyase or cysteine synthetase. Serine acetyltransferase and *O*-acetyl serine lyase can form a multi-enzyme complex in bacteria. In addition, the cysteine molecule may play a crucial role in the redox signaling of different stress processes [48], [49]. Our proteomic results demonstrate that *O*-acetylhomoserine (thiol)-lyase is upregulated (spot 63) or induced (spot 18) in F4. Therefore, F4 might be relatively more sensitive to changes in redox conditions in plant cells compared with F1. Serine-type carboxypeptidase cleaves the C-terminal peptide bond in peptides, thereby releasing the terminal amino acid [30]. This peptidase is widely observed in fungi, plants, and animals and has diverse biological functions [31]. In *Aspergillus oryzae*, this enzyme is required for normal hyphal growth, and the deletion strain formes fewer conidia on agar plates [32]. We identified two protein spots in F4 as carboxypeptidase, indicating that carboxypeptidase might be important for hyphal growth and conidiation of *F. oxysporum*.

Among the F4-specific proteins, catalase-peroxidase (KatG) is a unique bifunctional oxidoreductase with peroxidatic and catalatic activities within a single active site [38], [50]. KatG can be secreted out of fungal cells to perform distinct functions [50]. Extracellular KatG is exclusively found in phytopathogenic fungi [51]–[53]. In the rice blast fungus *Magnaporthe grisea*, KatG protects the pathogen from the increased level of H_2_O_2_ in rice epidermal cells at the early stage of infection [53]. Secretion KatG, as well as another catalase, is important for both hyphal growth and maintenance of the integrity of the fungal cell wall [52]. In *Verticillium longisporum*, KatG protectes the fungus from the oxidative response of host plants at advanced stages of the disease [54]. In our present study, we identified several protein spots as catalase-peroxidase, one of them being more abundant in F4 than in F1, and the others were specially detected only in F4 (Data S1), indicating that this enzyme might be key in the virulence of *F. oxysporum*.

Many glycoside hydrolases were found to be overrepresented in F4. These enzymes are responsible for the hydrolysis of sugar residues and polysaccharides. The chitinolytic enzymes chitinase and hexosaminidase were identified in F4-specific spots in this research. Chitinase hydrolyzes β-1,4 linkages in chitin polymers, endolytically producing chitooligosaccharide and chitobiose. Although hexosaminidase does not directly hydrolyze chitin polymers, it has high acetylhexosaminidase activity, which helps to degrade chitooligosaccharide into monomers [55]. Overexpression of chitinase 3 gene can improved fungal cell wall formation in both hyphal tips and germinated spores [37]. The exo-acting galactosidase enzyme mainly cleaves α-1-6-linked d-galactosyl residues into oligosaccharides, polysaccharides, and synthetic substrates. Upon infection with *Fusarium moniliforme* and *Fusarium proliferatum*, maize grain produces some hydrolytic enzymes, such as galactosidase, glucosidase and glucosaminidase, which enable the fumonisin-producing *Fusarium* to rapidly infect living maize grain [56]. In our study, chitinase and α-galactosidase were specially expressed in F4 (Data S1), which might help this race to efficiently infect banana plants.

### Enzymes involved in carbohydrate metabolism, energy production and ion transport might play important roles in banana Fusarium wilt

Proteins involved in carbohydrate metabolism are important for energy production because they participate in glycolysis to produce ATP. In *Paracoccidioides Pb 01*, the high abundance of several enzymes, such as enolase, FBA, phosphoglycerate kinase, and triosephosphate isomerase, leads to relatively higher anaerobic metabolism of glucose and energy production [57]. Here, we found that several enzymes involved in carbohydrate metabolism were more highly expressed in F4 than in F1. Among them, chitinase 1 and α-galactosidase 2 are important for the strong virulence of F4 [37], [56]. Glucose-6-phosphate 1-epimerase (spot 99) participates in glycolysis and gluconeogenesis by catalyzing the reversible conversion of α-d-glucose-6-phosphate to β-d-glucose-6-phosphate [58]. Enolase (spot 22), a ubiquitous glycolytic enzyme, catalyzes the dehydration of 2-phospho-d-glycerate to phosphoenolpyruvate in glycolysis. Additionally, adenosine kinase (spot 3) is important for the regulation of cellular levels of adenosine and its nucleotides. The above three enzymes are directly involved in glycolysis, and their higher expression levels in F4 might help this race to survive the plant defense system. ATP synthesis is necessity for all organisms and depends on the transmembrane hydrogen ion/proton transporting activity of ATP synthase. We identified 4 protein spots as ATP synthase (spots 38, 40, 46, and 49). Although there is no direct evidence that these enzymes contribute to the virulence of *F. oxysporum*, these proteins appear to be of some importance for F4. Future knockout studies of these genes to produce weakly pathogenic or non-pathogenic strains will provide additional insight for further investigation of banana Fusarium wilt.

Proteins involved in ion transport activate key components of the enzymatic antioxidant machinery, which is important for scavenging reactive oxygen species (ROS) and maintaining higher redox states in plant cells under high salinity [59]. ROS production by plants is part of the general defense response against pathogenic microorganisms [60], [61]. Expression of catalase in F4 is significantly induced in F4 after exposure to exogenous H_2_O_2_ [62]. Therefore, catalase may participate in the elimination of ROS produced by F4 infection in banana root. Peroxiredoxins, which can regulate H_2_O_2_ levels to cause damage and/or affect signal transduction processes, play important roles in proliferation, differentiation, and apoptosis pathways [36], [63]. Several catalase-peroxidase isoforms were identified in our study, which may scavenge ROS as well as combine with other ion transporters to play important roles in the Fusarium-induced infection of banana wilt.

## Supporting Information

Data S1 Proteins identified by MS/MS from F1 and F4.
(PDF)

Data S2 Protein spots patterns and MS information.
(XLS)

Data S3 Supplemental spectra and MS/MS identification information.
(PDF)

Data S4 Blast2GO analysis results for the identified proteins.
(XLS)

Data S5 Primers and RT-PCR results for 15 typical genes.
(XLS)

Data S6 Summary of protein isoforms.
(XLS)
