# Supplementary material for: Proteomics of Fusarium oxysporum Race 1 and Race 4 Reveals Enzymes Involved in Carbohydrate Metabolism and Ion Transport That Might Play Important Roles in Banana Fusarium Wilt
Source: PLoS One. 2014 Dec 2;9(12):e113818. doi: 10.1371/journal.pone.0113818 (PMC4252058; doi:10.1371/journal.pone.0113818)
Supplement: Data S1 — Proteins identified by MS/MS from F1 and F4. (PDF) [file pone.0113818.s001.pdf]

Suppl Data 1 **Table 1** Proteins identified by MS/MS from F1 and F4

Spot No.[a]	NCBI No.[b]	Function Category Protein name	Fungal species	Exper.[c] *p*I/M*r*	Theor.[d] *p*I/M*r*	MP /TP[e]	SC% (Loc)[f]	MS. S.[g]	Change (F1, F4)[h]
**Carbohydrate transport and metabolism (CTM)**									
**3**	475667672	adenosine kinase	*F. oxysporum*	5.47/33.3	5.34/38.1	15/62	59(_)	143	
**7**	475667960	phospho-kinase	*F. oxysporum*	5.8/48.3	5.77/44.8	18/72	56(_)	90	
**9**	475673861	fructose-aldolase	*F. oxysporum*	5.74/36.2	5.44/39.8	17/49	51(_)	170	
**12**	477513214	beta-hexosaminidase	*F. oxysporum*	6.73/35.3	6.25/34.2	11/69	29(*)	83	
**13**	475667960	Phospho-kinase	*F. oxysporum*	5.92/44.4	5.77/44.8	16/111	48(_)	76	
**16**	3810873	lactonohydrolase	*F. oxysporum*	5.32/50.2	5.10/43.2	10/60	31(_)	78	
**22**	475668982	putative enolase	*F. oxysporum*	5.20/48.4	5.06/47.5	22/77	57(*)	129	
**31**	475667960	Phospho-kinase	*F. oxysporum*	5.80/46.3	5.77/44.8	21/59	57(_)	159	
**33**	475668458	glucanosyltransferase	*F. oxysporum*	4.72/62.5	4.83/59.2	22/77	44(*)	132	
**34**	475668458	glucanosyltransferase	*F. oxysporum*	4.80/60.3	4.87/59.1	19/79	42(*)	92	
**35**	477513214	beta-hexosaminidase	*F. oxysporum*	6.80/37.7	6.25/34.2	(1)	4(*)	(93)	
**36**	475667960	phospho-kinase	*F. oxysporum*	5.92/47.4	5.77/44.8	20/56	55(_)	162	
**41**	342888171	carboxy-cyclase	*F. oxysporum*	6.71/46.3	6.67/42.4	14/58	43(S)	88	
**42**	475672613	Alpha-galactosidase	*F. oxysporum*	4.6/84.32	5.01/83.1	18/65	29(S)	80	
**43**	475672613	alpha-galactosidase	*F. oxysporum*	4.80/84.3	5.01/83.1	24/79	36(S)	113	
**50**	475668982	putative enolase	*F. oxysporum*	4.80/47.7	5.06/47.5	17/63	38(*)	80	
**51**	477507289	acethexosaminidase	*F. oxysporum*	5.5/77.3	5.22/66.1	24/51	48(S)	216	
**53**	475668458	glucanosyltransferase	*F. oxysporum*	5.12/70.2	4.83/59.2	15/62	29(*)	76	
**58**	477514377	alpha-galactosidase	*F. oxysporum*	4.7/84.3	5.13/82.9	15/50	27(S)	77	
**60**	475673339	Chitinase 1	*F. oxysporum*	6.8/62.3	6.31/47.9	16/66	36(S)	93	
**64**	3810873	lactonohydrolase	*F. oxysporum*	5.3/65.65	5.10/43.2	11/58	31(_)	75	
**68**	475668458	glucanosyltransferase	*F. oxysporum*	4.9/67.2	4.83/59.2	26/86	52(*)	144	
**76**	342872804	phospholipase B	*F. oxysporum*	4.67/77.4	4.67/70.5	15/57	25(S)	85	
**77**	342872804	phospholipase B	*F. oxysporum*	4.75/77.4	4.67/70.5	(4)	8(S)	253	
**78**	342872804	phospholipase B	*F. oxysporum*	4.50/77.4	4.67/70.5	(2)	2(S)	(80)	
**79**	475673339	Chitinase 1	*F. oxysporum*	6.9/55.3	6.31/47.9	18/55	39(S)	101	
**80**	475672613	galactosidase	*F. oxysporum*	5.1/84.3	5.01/83.1	35/67	47(S)	211	
**83**	475672613	galactosidase	*F. oxysporum*	5.1/84.3	5.01/83.1	27/62	35(S)	150	
**98**	477513214	hexosaminidase	*F. oxysporum*	6.7/37.65	6.25/34.2	12/64	31(*)	83	
**99**	475666766	Glucose-epimerase	*F. oxysporum*	4.62/35.4	4.95/33.7	10/60	46(*)	83	
**Posttranslational modification, protein turnover, chaperones (PTM)**									
**10**	342888261	peroxiredoxin	*F. oxysporum*	6.17/20.3	5.86/25.4	15/71	71(*)	122	
**17**	342878892	carboxypeptidase	*F. oxysporum*	6.20/62.3	6.04/59.9	21/70	41(*)	135	
**21**	475663822	Hsp 70	*F. oxysporum*	5.04/73.2	5.00/70.9	23/70	44(_)	142	
**23**	342882947	vacuolar protease A	*F. oxysporum*	4.45/44.7	4.66/42.9	(6)	10(S)	218	
**24**	342882947	vacuolar protease A	*F. oxysporum*	4.38/44.7	4.66/42.9	9/39	21(S)	95	
**29**	342882947	vacuolar protease A	*F. oxysporum*	4.38/44.7	4.66/42.9	8/49	19(S)	84	
**32**	475667235	elongation factor 1	*F. oxysporum*	5.90/48.5	5.64/46.2	19/72	51(*)	123	
**45**	342876003	disulfide isomerase	*F. oxysporum*	6.8/39.4	6.29/42.1	21/81	60(S)	148	

**47**	170098594	ubiquitin	*L. bicolor*	6.3/9.7	5.71/8.2	7/61	84(_)	81
**54**	607818	Hsp 70	*N. crassa*	4.90/48.7	5.03/70.8	(3)	5(_)	(83)
**56**	475674506	Peroxiredoxin-1	*F. oxysporum*	4.96/23.5	5.14/23.3	(4)	14(*)	127
**59**	475664853	Carboxypeptidase	*F. oxysporum*	7.0/70.3	6.71/57.5	(1)	2(_)	(62)
**70**	477507989	Hsp 70	*F. oxysporum*	6.1/83.2	5.00/71.1	25/46	53(_)	217
**71**	342889878	Hsp 90	*F. oxysporum*	5.0/70.3	4.85/79.3	26/67	41(_)	174
**73**	477507989	Hsp 70	*F. oxysporum*	5.0/70.3	5.00/71.2	21/61	26(_)	117
**74**	342889878	Hsp 90	*F. oxysporum*	5.0/78.7	4.85/79.3	19/59	34(_)	110
**85**	475670597	Carboxypeptidase	*F. oxysporum*	5.45/68.3	5.94/64.9	14/58	29(S)	78
**94**	322700250	Hsp 90	*M. acridum*	4.74/24.2	4.93/80.1	13/52	26(_)	88
**Inorganic ion transport and metabolism (ITM)**								
**5**	475672437	Catalase-peroxidase	*F. oxysporum*	6.80/80.2	6.57/85.2	29/71	37(S)	147
**6**	477512799	superoxide dismutase	*F. oxysporum*	6.15/16.6	7.07/21.3	11/78	67(S)	95
**15**	477512799	superoxide dismutase	*F. oxysporum*	6.7/17.2	7.07/21.3	12/53	51(S)	106
**28**	477512799	superoxide dismutase	*F. oxysporum*	6.41/22.3	7.07/21.3	10/59	55(S)	122
**65**	475671755	Catalase-peroxidase	*F. oxysporum*	6.5/85.3	5.98/73.3	19/51	39(_)	126
**86**	475672437	Catalase-peroxidase	*F. oxysporum*	6.8/78.3	6.57/85.2	33/64	42(S)	216
**87**	475672437	Catalase-peroxidase	*F. oxysporum*	6.7/78.3	6.57/85.2	37/84	41(S)	219
**88**	475672437	Catalase-peroxidase	*F. oxysporum*	6.9/78.3	6.57/85.2	39/92	49(S)	249
**89**	475672437	Catalase-peroxidase	*F. oxysporum*	6.79/78.3	6.57/85.2	39/61	48(S)	314
**90**	475672437	Catalase-peroxidase	*F. oxysporum*	6.71/78.3	6.57/85.2	33/61	42(S)	236
**92**	475672437	Catalase-peroxidase	*F. oxysporum*	6.69/78.3	6.57/85.2	41/87	49(S)	250
**Amino acid transport and metabolism (ATM)**								
**18**	475675629	Acetyl-lyase	*F. oxysporum*	6.50/44.4	5.89/47.1	13/52	42(_)	88
**19**	477517374	hom-dehydrogenase	*F. oxysporum*	6.14/38.1	6.24/38.6	12/38	50(*)	104
**26**	46128717	peptidase	*F. gramiarum*	5.15/22.3	5.53/55.4	(2)	2(_)	(92)
**39**	477517233	l-amino acid oxidase	*F. oxysporum*	6.2/73.5	5.69/67.8	17/46	24(S)	90
**63**	475675629	Acetyl-lyase	*F. oxysporum*	6.54/58.7	5.89/47.1	15/35	57(_)	134
**82**	477517233	l-amino acid oxidase	*F. oxysporum*	6.5/80.7	5.69/67.8	23/83	35(S)	98
**84**	477517233	l-amino acid oxidase	*F. oxysporum*	6.2/80.6	5.69/67.8	19/60	25(S)	82
**91**	477517233	l-amino acid oxidase	*F. oxysporum*	6.14/80.7	5.69/67.8	19/64	26(S)	86
**93**	342871725	l-amino acid oxidase	*F. oxysporum*	6.14/80.7	5.69/67.8	18/69	27(S)	81
**Energy production and conversion (EPC)**								
**20**	475671099	glutathione reductase	*F. oxysporum*	6.64/53.4	6.06/51.1	19/76	67(_)	121
**38**	475664589	ATP synthase	*F. oxysporum*	6.7/20.3	5.27/55.2	30/68	71(M)	206
**40**	475664589	ATP synthase	*F. oxysporum*	4.76/54.3	5.27/55.2	24/84	64(M)	128
**46**	475664589	ATP synthase	*F. oxysporum*	4.95/54.3	5.27/55.2	28/71	68(M)	168
**49**	475664589	ATP synthase	*F. oxysporum*	4.75/26.5	5.27/55.2	(2)	5(M)	117
**67**	354543047	hypothetical protein	*C. parapilosis*	4.7/18.3	10.1/23.1	9/80	38(_)	80
**75**	342876053	ATP synthase D chain	*F. oxysporum*	4.55/84.3	6.43/19.4	10/59	60(*)	98
**Coenzyme metabolism (COM)**								
**8**	475664008	thionine synthetase	*F. oxysporum*	5.92/45.6	5.77/44.5	16/65	55(*)	90
**11**	475664008	thionine synthetase	*F. oxysporum*	6.05/41.3	5.77/44.5	14/49	48(_)	130
**37**	342873590	Adenosyl-cysteinase	*F. oxysporum*	5.74/46.2	5.57/49.5	28/85	57(*)	191

**DNA replication, recombination and repair (DRR)**									
**55**	342881318	actin binding protein	*F. oxysporum*	4.9/22.7	4.84/18.8	16/86	69(_)	109	
**66**	342890194	DEAD box helicase	*F. oxysporum*	5.2/57.5	4.98/44.9	21/68	55(_)	119	
**Transcription, translation, ribosomal structure and biogenesis (TRR)**									
**14**	477513214	hexosaminidase	*F. oxysporum*	6.5/26.3	6.25/34.2	10/68	26(_)	80	
**81**	475668007	amidase	*F. oxysporum*	6.5/64.3	6.86/63.7	18/64	39(S)	112	
**Cell motility and secretion (CMS)**									
**62**	283806340	oxidoreductase	*F. oxysporum*	5.5/73.2	5.20/71.4	9/24	27(S)	83	
**General function prediction only (Predicted)**									
**2**	342881721	amidotransferase	*F. oxysporum*	5.35/25.3	5.12/24.5	18/35	74(*)	165	
**4**	342888721	Glycer-dehydrogenase	*F. oxysporum*	6.25/37.5	5.91/37.4	18/43	53(*)	157	
**25**	342888721	Glycer-dehydrogenase	*F. oxysporum*	6.1/36.5	5.91/37.4	20/75	59(*)	137	
**27**	342888721	Glycer-dehydrogenase	*F. oxysporum*	6.21/36.5	5.91/37.4	23/47	61(*)	208	
**44**	342878413	Quin-oxidoreductase	*F. oxysporum*	5.60/18.7	5.54/21.7	(5)	18(_)	163	
**52**	342879444	Nitropro-dioxygenase	*F. oxysporum*	6.6/50.3	5.78/36.9	18/61	54(*)	136	
**NO related COG (NO)**									
**1**	475667056	hypothetical protein	*F. oxysporum*	6.24/51.3	6.62/50.5	16/64	37(S)	96	
**30**	475667318	actin	*F. oxysporum*	5.45/44.3	6.08/45.2	19/62	53(_)	119	
**48**	342873137	transport factor 2	*V. dahliae*	4.50/14.4	4.61/13.9	(2)	12(_)	166	
**57**	340924179	hypothetical protein	*C. therhilum*	5.10/54.4	10.6/17.9	9/66	51(_)	79	
**61**	171680085	hypothetical protein	*P. anserina*	4.8/77.4	5.61/41.3	10/24	34(*)	81	
**69**	342872822	Phospho-mutase	*F. oxysporum*	5.3/14.3	5.21/12.4	8/61	70(_)	91	
**95**	475667056	hypothetical protein	*F. oxysporum*	6.06/50.5	6.62/50.5	17/72	38(S)	110	
**96**	475671883	Ribonuclease Trv	*F. oxysporum*	5.95/31.3	5.61/29.7	(3)	13(S)	185	
**97**	477507337	14-3-3 protein	*F. oxysporum*	4.85/34.1	4.87/30.1	13/60	61(_)	92	

Note:
[a]Assigned spot number as indicated in Fig. 2.
[b]NCBI non-redundant database accession numbers.
[c]Experimental [molecular mass (kDa) OR relative molecular mass (M*r*)] and pI of identified proteins.
[d]Theoretical [molecular mass (kDa) OR relative molecular mass (M*r*)] and pI of identified proteins.
[e]Number of matched peptides (MP) with PMF and the total searched peptides (TP). Numbers in parentheses are the MP based on MS/MS.
[f]The amino acid sequence coverage (SC) for the identified proteins and the protein location (Loc) predicted by TargetP. “M” represent the protein located at mitochondria, “S” represent the protein has signal peptidase, “_” represent the protein have multiple location, “*” represent the location of proteins was unkown.
[g]The Mascot searched score (MS) against the NCBI non-redundant database. The numbers in parentheses represent the protein was identified by MS/MS.
[h]The mean relative protein values on 2-DE gels. White, F1; black, F4.
