# Supplementary material for: Proteomics of Fusarium oxysporum Race 1 and Race 4 Reveals Enzymes Involved in Carbohydrate Metabolism and Ion Transport That Might Play Important Roles in Banana Fusarium Wilt
Source: PLoS One. 2014 Dec 2;9(12):e113818. doi: 10.1371/journal.pone.0113818 (PMC4252058; doi:10.1371/journal.pone.0113818)
Supplement: Data S3 — Supplemental spectra and MS/MS identification information. (PDF) [file pone.0113818.s003.pdf]

Supplemental spectra and MALDI TOF/TOF MS/MS identification information

Annotated spectra for Suppl Data 1: 99 proteins identified by MALDI TOF MS and MALDI TOF/TOF MS/MS.

Spot numbers of the 99 proteins correspond to the proteins that listed in Suppl Data 1.

CID: collision induced dissociation

MALDI TOF:

matrix assisted laser desorption/ionization time of flight

MS: mass spectrometry

PMF: peptide mass fingerprinting

Spot No.: **1**

Mascot score: 96

Species: ***Fusarium oxysporum f. sp. cubense race 4***

Protein name: **hypothetical protein FOC4_g10007540**

NCBI accession No.: **gi| 475667056**

Sequence coverage %: **37**

Matched peptides No.: **16**

Total peptides No.: **64**

Calculated Mr: **50542**

Calculated *p*I: **6.62**

Annotated PMF **spectra:**

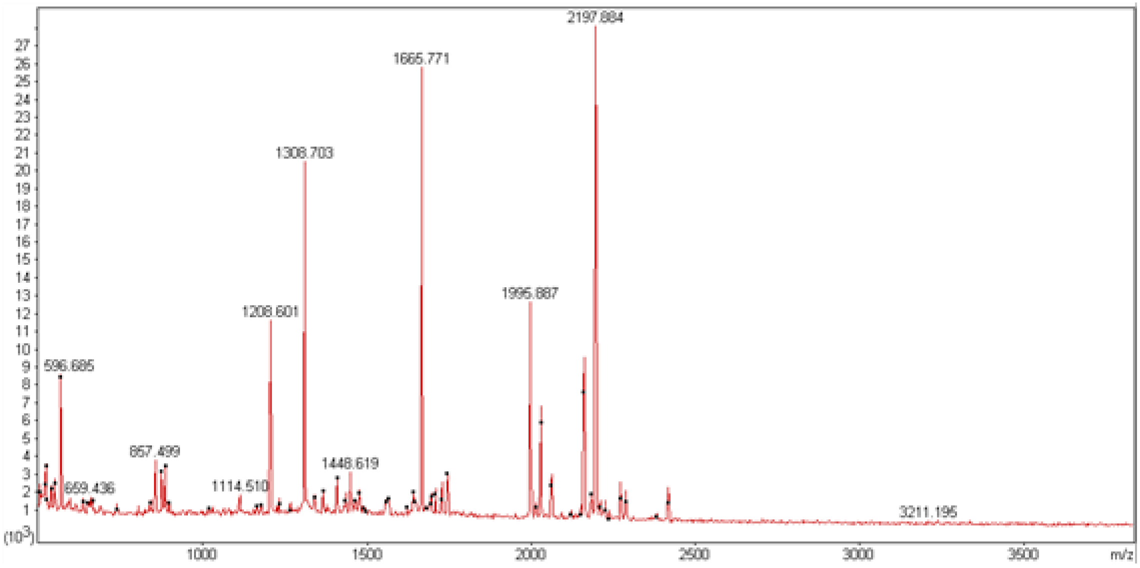

PMF Searched Score:

Protein score is -10*Log(P), where P is the probability that the observed match is a random event.
Protein scores greater than 75 are significant (p<0.05).

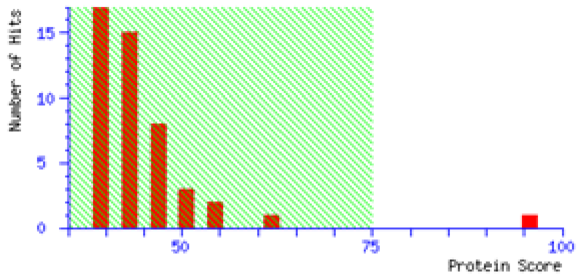

Matched peptide sequences: shown in **Bold Red**

1	MVSSKLLFLA	VIAAVQAIDV	DVAVVGGGGS	GGYAAVQLRE	NYGKKIVVIE
51	KQK**QLGGHAQ**	**SWYDPVTGK**A	YNYGVDAFTN	ITVSIDFFKQ	LK**VPIGPVQS**
101	**EQVRNLYVDF**	**K**DGKTVDYTP	PTTKEAADAM	GNYRDQWLK**Y**	**TDILLPTSEN**
151	**FPRGDKVPSD**	**LLLSWYEFAR**	KYKAEAASPS	IWDTVVVDLN	TALMIDVWK**A**
201	**WNPSVGSFQP**	**ASGDNTEIWQ**	**K**AAKVLGK**DV**	**LYESEVVSAK**	RSKSGVKLQV
251	RDKDGQVINI	NAK**RLLITIG**	**PETINPKYFD**	**LNSEELEVFH**	**SAAGNR**YYTG
301	IVSHPSLPAA	EITNIVPAAI	NENYLAYPTV	PFQAYFHYKG	NSSTGPIHR**A**
351	**LAIVPR**DTSI	EDAKDLIRKS	LQNLIDAGTI	PAGNSTDLDF	R**TFSDHGLLY**
401	**RRWSADQLRG**	**GIFAKANALQ**	**GQR**STWYTGA	FWMNNDCVML	WNTTNAILKE
451	MLKDI				

Matched peptides information:

Start – End	Observed	Mr(expt)	Mr(calc)	ppm	M	Peptide
54 – 69	1743.7194	1742.7121	1742.8376	-72.0	0	K.QLGGHAQSWYDPVTGK.A
93 – 104	1308.7033	1307.6960	1307.7198	-18.1	0	K.VPIGPVQSEQVR.N
105 – 111	898.4591	897.4519	897.4596	-8.63	0	R.NLYVDFK.D
140 – 153	1665.7714	1664.7641	1664.8410	-46.2	0	K.YTDILLPTSENFPR.G
154 – 170	1995.8870	1994.8797	1995.0102	-65.4	1	R.GDKVPSDLLLSWYEFAR.K
157 – 170	1695.7644	1694.7571	1694.8668	-64.7	0	K.VPSDLLLSWYEFAR.K
200 – 221	2418.9644	2417.9571	2418.1240	-69.0	0	K.AWNPSVGSFQPASGDNTEIWQK.A
229 – 240	1338.6190	1337.6117	1337.6714	-44.6	0	K.DVLYESEVVSAK.R
264 – 277	1564.8337	1563.8264	1563.9348	-69.3	1	K.RLLITIGPETINPK.Y
265 – 277	1408.7512	1407.7439	1407.8337	-63.8	0	R.LLITIGPETINPK.Y
278 – 296	2197.8838	2196.8765	2197.0076	-59.7	0	K.YFDLNSEELEVFHSAAGNR.Y
350 – 356	739.4868	738.4795	738.4752	5.86	0	R.ALAIVPR.D
392 – 401	1208.6008	1207.5935	1207.5986	-4.18	0	R.TFSDHGLLYR.R
403 – 409	875.4877	874.4804	874.4297	58.0	0	R.WSADQLR.G
403 – 415	1448.6193	1447.6120	1447.7572	-100	1	R.WSADQLRGGIFAK.A
416 – 423	857.4987	856.4914	856.4515	46.6	0	K.ANALQGQR.S

Spot No.: **2**

Mascot score: **165**

Species: ***Fusarium oxysporum Fo5176***

Protein name: **amidotransferase**

NCBI accession No.: **gi| 342881721**

Sequence coverage %: **74**

Matched peptides No.: **18**

Total peptides No.: **35**

Calculated Mr: **24474**

Calculated *p*I: **5.12**

Annotated PMF **spectra:**

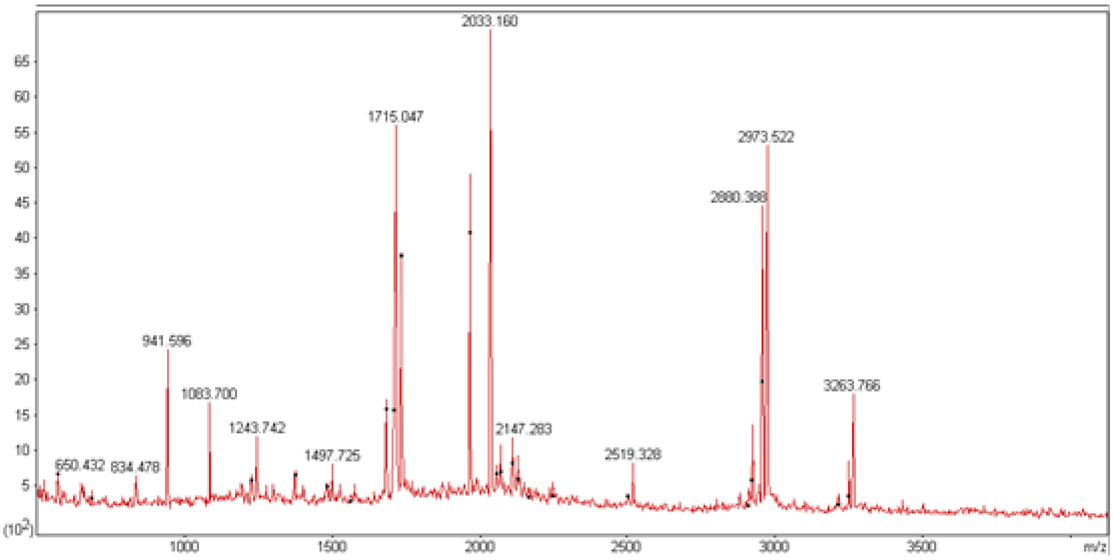

Probability Based Mowse Score:

Protein score is -10*Log(P), where P is the probability that the observed match is a random event.
Protein scores greater than 75 are significant (p<0.05).

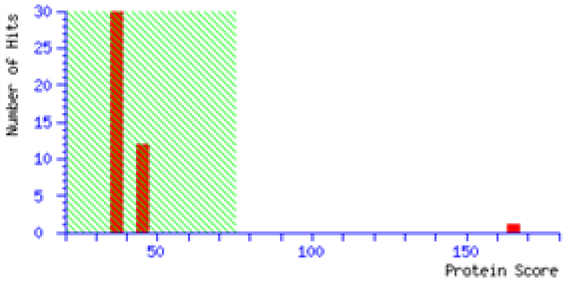

Matched peptide sequences: shown in **Bold Red**

1	MAPKVLVVLT	SQSKMNNGHP	TGWYLPEFAH	PYYDLVNK**GV**	**EITVASPAGG**
51	**EAPLDQASVE**	**MFKGDEESVK**	**FLNEKKQIWE**	**QTTPLK**EFLG	K**ASEFDAIFY**
101	**PGGHGPMFDL**	**VNDETSIKLI**	**EEFYKAGKPV**	**AAVCHGPIVF**	**VNVKVDGKPL**
151	**LEGREATGFS**	**NSEEEAVKLT**	**SAMPVLLEDE**	**IKR**VGGKYVK	**ADDWAEKLAV**
201	**DGQVITGQNP**	**ASAHAVGK**AI	LKAIGA		

Matched peptides information:

Start – End	Observed	Mr(expt)	Mr(calc)	ppm	M	Peptide
39 – 63	2503.3027	2502.2954	2502.2312	25.7	0	K.GVEITVASPAGGEAPLDQASVEMFK.G
39 – 63	2519.3277	2518.3204	2518.2261	37.4	0	K.GVEITVASPAGGEAPLDQASVEMFK.G + Oxidation (M)
39 – 70	3247.7309	3246.7236	3246.5602	50.3	1	K.GVEITVASPAGGEAPLDQASVEMFKGDEESVK.F
39 – 70	3263.7660	3262.7587	3262.5551	62.4	1	K.GVEITVASPAGGEAPLDQASVEMFKGDEESVK.F + Oxidation (M)
71 – 75	650.4320	649.4247	649.3435	125	0	K.FLNEK.K
76 – 86	1371.8022	1370.7949	1370.7558	28.6	1	K.KQIWEQTTPLK.E
77 – 86	1243.7422	1242.7349	1242.6608	59.6	0	K.QIWEQTTPLK.E
92 – 118	2957.4826	2956.4753	2956.3589	39.4	0	K.ASEFDAIFYPGGHGPMFDLVNDETSIK.L
92 – 118	2973.5224	2972.5151	2972.3539	54.3	0	K.ASEFDAIFYPGGHGPMFDLVNDETSIK.L + Oxidation (M)
119 – 125	941.5961	940.5888	940.4906	105	0	K.LIEEFYK.A
126 – 144	1963.1677	1962.1604	1962.0873	37.2	0	K.AGKPVAAVCHGPIVFVNVK.V
145 – 154	1083.6999	1082.6926	1082.6084	77.8	0	K.VDGKPLLEGR.E
155 – 168	1497.7252	1496.7179	1496.6631	36.7	0	R.EATGFSNSEEEAVK.L
169 – 182	1558.8878	1557.8805	1557.8324	30.9	0	K.LTSAMPVLLEDEIK.R
169 – 183	1715.0472	1714.0399	1713.9335	62.1	1	K.LTSAMPVLLEDEIKR.V
169 – 183	1731.0303	1730.0230	1729.9284	54.7	1	K.LTSAMPVLLEDEIKR.V + Oxidation (M)
191 – 197	834.4782	833.4709	833.3555	138	0	K.ADDWAEK.L
198 – 218	2033.1597	2032.1524	2032.0702	40.5	0	K.LAVDGQVITGQNPASAHAVGK.A

Spot No.: **3**

Mascot score: **143**

Species: ***Fusarium oxysporum f. sp. cubense race 4***

Protein name: **Adenosine kinase**

NCBI accession No.: **gi| 475667672**

Sequence coverage %: **59**

Matched peptides No.: **15**

Total peptides No.: **62**

Calculated Mr: **38182**

Calculated *p*I: **5.34**

Annotated PMF **spectra:**

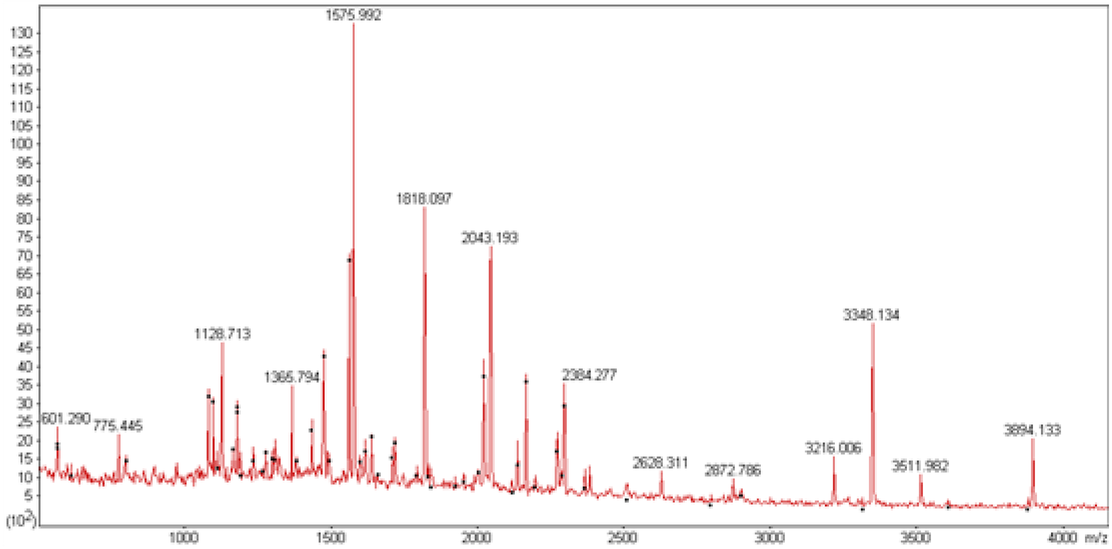

Probability Based Mowse Score:

Protein score is -10*Log(P), where P is the probability that the observed match is a random event.
Protein scores greater than 75 are significant (p<0.05).

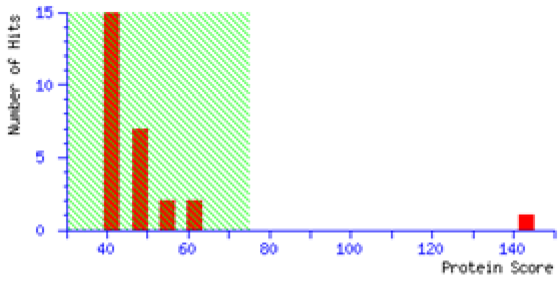

Matched peptide sequences: shown in **Bold Red**

1 MSAVK**EYSLL** **CLENPLLDIQ** **AK**GDQALLDK **YGLKPNDAIL** **AEEKHLPLYE**
51 **DLLNNYDAKL** **IAGGAAQNSA** **RGAQYILPPN** **SVVYLGGAGD** **DKYAAILHDA**
101 **VK**AAGLR**VEY** **R**VDPKEKTGR **CGAIITGHNR** **SLCTDLGAAN** **HYDLDHLKKP**
151 **EIWK**LVENAE VYYVGGFHFT VCPPAIMELA KQAAEHNKPF VLSLSAPFIP
201 QFFK**EVVDAS** **APYWDYIIGN** **ETEAAAYAES** **HDLPSKEPR**D VVKHLANLPK
251 ENTKRKR**IAI** **VTQGTDPTLV** **AIQGEDDIKE** **FPVHAIEK**EK INDTNGAGDA
301 FAGGLLAGIL QNKPLETSID MGQWLAR**LSI** **QELGPSYPFP** **K**QTYQAA

Matched peptides information:

Start	–	End	Observed	Mr(expt)	Mr(calc)	ppm	M	Peptide
6	–	22	2019.2720	2018.2647	2018.0394	112	0	K.EYSLLCLENPLLDIQAK.G
31	–	44	1560.9581	1559.9508	1559.8195	84.2	0	K.YGLKPNDAILAEEK.H
45	–	59	1818.0969	1817.0896	1816.8995	105	0	K.HLPLYEDLLNNYDAK.L
60	–	71	1128.7127	1127.7054	1127.6047	89.3	0	K.LIAGGAAQNSAR.G
72	–	92	2134.3160	2133.3087	2133.0742	110	0	R.GAQYILPPNSVVYLGGAGDDK.Y
72	–	102	3216.0063	3214.9990	3214.6662	104	1	R.GAQYILPPNSVVYLGGAGDDKYAAILHDAVK.A
108	–	111	566.2984	565.2911	565.2860	9.06	0	R.VEYR.V
121	–	130	1098.6394	1097.6321	1097.5400	83.9	0	R.CGAIITGHNR.S
131	–	148	2043.1928	2042.1855	2041.9527	114	0	R.SLCTDLGAANHYDLDHLK.K
149	–	154	800.4406	799.4333	799.4592	-32.4	0	K.KPEIWK.L
205	–	236	3511.9815	3510.9742	3510.6103	104	0	K.EVVDASAPYWDYIIGNETEAAAYAESHDLPSK.E
205	–	239	3894.1332	3893.1259	3892.8067	82.0	1	K.EVVDASAPYWDYIIGNETEAAAYAESHDLPSKEPR.D
258	–	279	2297.5012	2296.4939	2296.2162	121	0	R.IAIVTQGTDPTLVAIQGEDDIK.E
258	–	288	3348.1341	3347.1268	3346.7660	108	1	R.IAIVTQGTDPTLVAIQGEDDIKEFPVHAIEK.E
328	–	341	1575.9920	1574.9847	1574.8344	95.4	0	R.LSIQELGPSYPFPK.Q

Spot No.: **4**

Mascot score: **157**

Species: ***Fusarium oxysporum Fo5176***

Protein name: **NADP-dependent glycerol dehydrogenase**

NCBI accession No.: **gi| 342888721** Sequence coverage %: **53**

Matched peptides No.: **18** Total peptides No.: **43**

Calculated Mr: **37413** Calculated *p*I: **5.91**

Annotated PMF **spectra:**

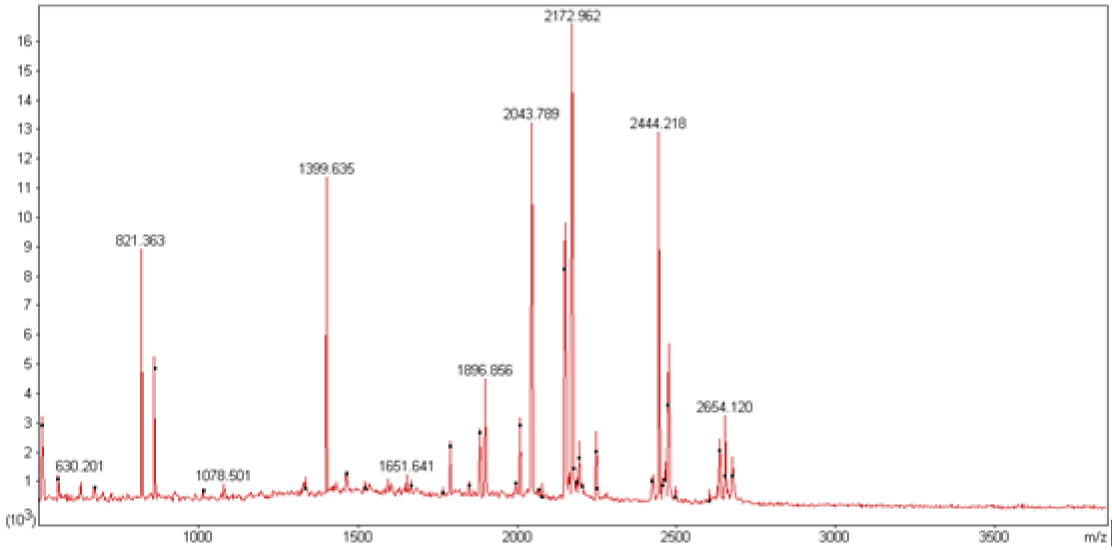

P

robability Based Mowse Score:

Protein score is -10*Log(P), where P is the probability that the observed match is a random event. Protein scores greater than 75 are significant (p<0.05).

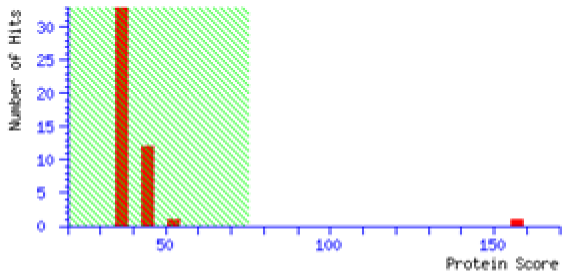

Matched peptide sequences: shown in Bold Red

```
  1 MSFGRTVTLN SGHKMPQIGY GTWQAAPGEV GNGVYEALKA GYRHLDLAKI
 51 YQNQREVGEG IKKALNDVPG LKREDIFITG KLWNNKHRPE EVPGALDDSL
101 EELGLDYLDL WLIHWPVAFK NGPELFPLKA DDKNKTELDQ GVTLSQTWEA
151 VTKLPKEKVR SIGVSNFSIE MLETIIKDTG VTPAINQVER HPRLPQPELV
201 KYQKEKGIYL TAYSAFGNNS WGEPLLINTP EVKAIAERLS KSKGKEVTPA
251 QVILAWSTLD NHIVIPKSVT PARIRSNFEE VELDEEAIKE LEKFGEKPQR
301 FNIPKTYSPD WDIDVFGDEK EKTATHKVVL KL
```

Red (matched) stretches: 15–39 MPQIGYGTWQAAPGEVGNGVYEALK; 50–55 IYQNQR; 73–86 REDIFITGKLWNNK; 121–129 NGPELFPLK; 134–153 NKTELDQGVTLSQTWEAVTK; 161–190 SIGVSNFSIEMLETIIKDTGVTPAINQVER; 234–238 AIAER; 246–273 EVTPAQVILAWSTLDNHIVIPKSVTPAR; 276–300 SNFEEVELDEEAIKELEKFGEKPQR; 306–322 TYSPDWDIDVFGDEKEK.

Matched peptides information:

Start	–	End	Observed	Mr(expt)	Mr(calc)	ppm	M	Peptide
15	–	39	2652.1644	2651.1571	2651.2690	-42.2	0	K.MPQIGYGTWQAAPGEVGNGVYEALK.A + Oxidation (M)
50	–	55	821.3626	820.3553	820.4191	-77.8	0	K.IYQNQR.E
73	–	81	1078.5007	1077.4934	1077.5818	-82.1	1	K.REDIFITGK.L
82	–	86	674.2166	673.2094	673.3547	-216	0	K.LWNNK.H
121	–	129	1014.4226	1013.4153	1013.5546	-137	0	K.NGPELFPLK.A
134	–	153	2248.0157	2247.0084	2247.1383	-57.8	1	K.NKTELDQGVTLSQTWEAVTK.L
136	–	153	2005.8656	2004.8583	2005.0004	-70.9	0	K.TELDQGVTLSQTWEAVTK.L
161	–	177	1880.8605	1879.8532	1879.9965	-76.2	0	R.SIGVSNFSIEMLETIIK.D
161	–	177	1896.8560	1895.8487	1895.9914	-75.2	0	R.SIGVSNFSIEMLETIIK.D + Oxidation (M)
178	–	190	1399.6346	1398.6273	1398.7103	-59.3	0	K.DTGVTPAINQVER.H
234	–	238	559.2314	558.2241	558.3125	-158	0	K.AIAER.L
246	–	267	2444.2185	2443.2112	2443.3475	-55.8	0	K.EVTPAQVILAWSTLDNHIVIPK.S
268	–	273	630.2008	629.1935	629.3497	-248	0	K.SVTPAR.I
276	–	289	1651.6410	1650.6337	1650.7624	-78.0	0	R.SNFEEVELDEEAIK.E
276	–	293	2150.8875	2149.8802	2150.0266	-68.1	1	R.SNFEEVELDEEAIKELEK.F
294	–	300	861.3838	860.3765	860.4504	-85.9	0	K.FGEKPQR.F
306	–	320	1786.6680	1785.6607	1785.7734	-63.1	0	K.TYSPDWDIDVFGDEK.E
306	–	322	2043.7891	2042.7818	2042.9109	-63.2	1	K.TYSPDWDIDVFGDEKEK.T

Spot No.: **5**

Mascot score: **147**

Species: ***Fusarium oxysporum f. sp. cubense race 4***

Protein name: **Catalase-peroxidase 2**

NCBI accession No.: **gi| 475672437** Sequence coverage %: **37**

Matched peptides No.: **29** Total peptides No.: **71**

Calculated Mr: **85233** Calculated *p*I: **6.57**

Annotated PMF **spectra:**

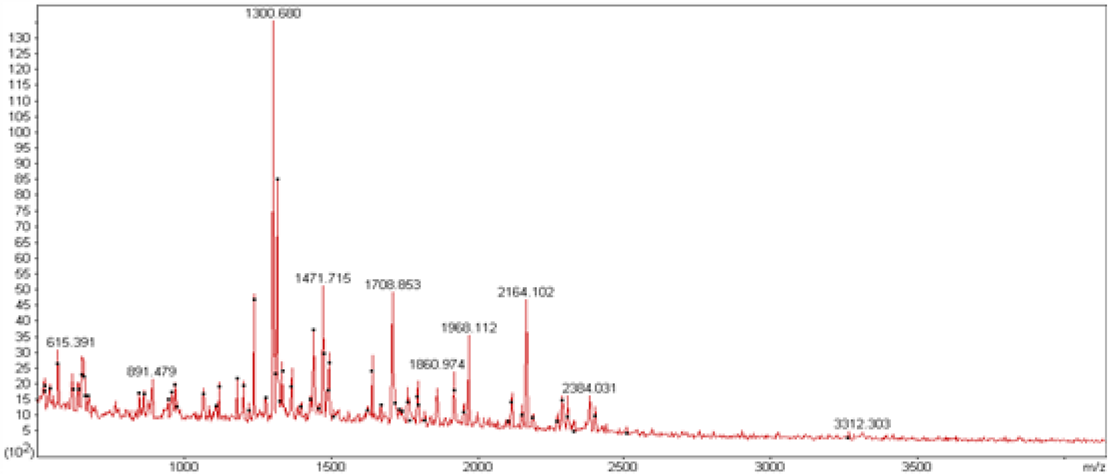

Probability Based Mowse Score:

Protein score is -10*Log(P), where P is the probability that the observed match is a random event.
Protein scores greater than 75 are significant (p<0.05).

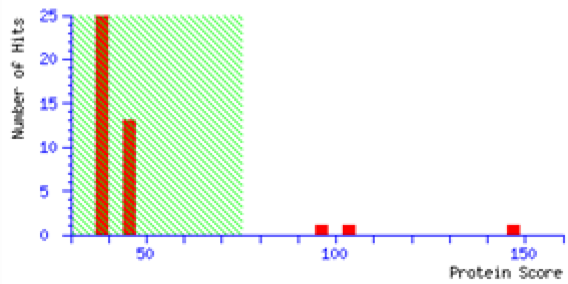

Matched peptide sequences: shown in **Bold Red**

```
  1 MHVQSLLLAS GLVPLAASQG CPFAKRATDT NLVPPREIPE DFGICRVASN
 51 QAGGGTRSRD FWPCALRLDV LRQFSPPYNP LGADFDYTEA FKSLDFAALK
101 KDLNALLTDS QDWWPADHGN YGGLFIRMSW HSAGTYRAMD GRGGSGMGQQ
151 RFAPLDSWPD NQNLDKARRL LWPIKQKYGS KISWADLMVL AGNVALEHSG
201 FETLGFAGGR ADTWEADESI YWGAESTFVP KGNDVRYNGS TDIYERADKL
251 EKPLGATHFG LIYVNPEGPD GSSDPKASAL DIRTAFGRMG MDDEETAALI
301 IGGHTLGKTH GAVPAKNIGP EPMAADLGEM GLGWHNSVNE GNGPDQMTSG
351 LEVIWSTTPT KWSNHFLKSL LGNNWTLVES PAGHKQWEAL NGKLEYPDPF
401 VKGKFRRPTM LTSDLALIND PSYLKICKRW HDNPKELNAA FARAWYKLLH
451 RDLGPVSRYL GPEVAKEKFI WQDPLPERKG DIIGEADISS LKSAILSADG
501 LDVSKLVSTA WNSASTFRGT DKRGGANGAR IALEPQVNWV SNNPKQLKQV
551 LSALKKVQKD FNSKSGSKKV SLADLIVLGG VAAIEKAAQA AGFKDVEVPF
601 TPGRVDATQN QTDLVQFGYL EPLADGFRNY GHGTARARTE EILVDRAALL
651 TLTPPEMTVL VGGLRALNAN YDGSSNGILT EKKGQLTNDF FVNLLSPAYS
701 WAKKDSQGEL WTGTDRATKS VKWTATRADL VFGSHAELRA ISEVYGSADA
751 KEKFVKDFIS AWTKVMNLDR FDVKAEK
```

Matched peptides information:

Start	-	End	Observed	Mr(expt)	Mr(calc)	ppm	M	Peptide
37	-	46	1235.5955	1234.5882	1234.5652	18.6	0	R.EIPEDFGICR.V
58	-	67	1307.6392	1306.6319	1306.6241	6.01	1	R.SRDFWPCALR.L
60	-	67	1064.5356	1063.5283	1063.4909	35.2	0	R.DFWPCALR.L
68	-	72	615.3906	614.3834	614.3752	13.3	0	R.LDVLR.Q
73	-	92	2307.0627	2306.0554	2306.0532	0.96	0	R.QFSPPYNPLGADFDYTEAFK.S
152	-	166	1759.8441	1758.8368	1758.8213	8.82	0	R.FAPLDSWPDNQNLDK.A
211	-	231	2402.0783	2401.0710	2401.0750	-1.66	0	R.ADTWEADESIYWGAESTFVPK.G
237	-	246	1217.6048	1216.5975	1216.5360	50.6	0	R.YNGSTDIYER.A
277	-	288	1277.6509	1276.6436	1276.6888	-35.4	1	K.ASALDIRTAFGR.M
394	-	402	1107.5554	1106.5481	1106.5648	-15.1	0	K.LEYPDPFVK.G
407	-	425	2148.1153	2147.1080	2147.1296	-10.1	0	R.RPTMLTSDLALINDPSYLK.I
407	-	425	2164.1022	2163.0949	2163.1245	-13.7	0	R.RPTMLTSDLALINDPSYLK.I + Oxidation (M)
436	-	443	891.4794	890.4721	890.4610	12.5	0	K.ELNAAFAR.A
469	-	478	1300.6798	1299.6725	1299.6612	8.74	0	K.FIWQDPLPER.K
469	-	479	1428.7609	1427.7536	1427.7561	-1.75	1	K.FIWQDPLPERK.G
506	-	518	1439.7273	1438.7200	1438.7205	-0.31	0	K.LVSTAWNSASTFR.G
531	-	545	1708.8530	1707.8457	1707.8944	-28.5	0	R.IALEPQVNWVSNNPK.Q
569	-	586	1796.0198	1795.0125	1795.0819	-38.6	1	K.KVSLADLIVLGGVAAIEK.A
570	-	586	1667.9532	1666.9459	1666.9869	-24.6	0	K.VSLADLIVLGGVAAIEK.A
587	-	604	1860.9741	1859.9668	1859.9530	7.42	1	K.AAQAAGFKDVEVPFTPGR.V
595	-	604	1116.5647	1115.5574	1115.5611	-3.33	0	K.DVEVPFTPGR.V
637	-	646	1201.6254	1200.6181	1200.6462	-23.4	1	R.ARTEEILVDR.A
647	-	665	1952.1142	1951.1069	1951.1176	-5.48	0	R.AALLTLTPPEMTVLVGGLR.A
647	-	665	1968.1119	1967.1046	1967.1125	-4.02	0	R.AALLTLTPPEMTVLVGGLR.A + Oxidation (M)
666	-	682	1766.8312	1765.8239	1765.8482	-13.8	0	R.ALNANYDGSSNGILTEK.K
704	-	716	1492.7039	1491.6966	1491.6954	0.83	1	K.KDSQGELWTGTDR.A
705	-	716	1364.6050	1363.5977	1363.6004	-1.99	0	K.DSQGELWTGTDR.A
728	-	739	1314.7044	1313.6971	1313.6728	18.5	0	R.ADLVFGSHAELR.A
757	-	764	967.4786	966.4713	966.4811	-10.1	0	K.DFISAWTK.V

Spot No.: **6**

Mascot score: **95**

Species: ***Fusarium oxysporum f. sp. cubense race 1***

Protein name: **Superoxide dismutase [Mn], mitochondrial**

NCBI accession No.: **gi| 477512799** Sequence coverage %: **67**

Matched peptides No.: **11** Total peptides No.: **78**

Calculated Mr: **21308** Calculated *p*I: **7.07**

Annotated PMF **spectra:**

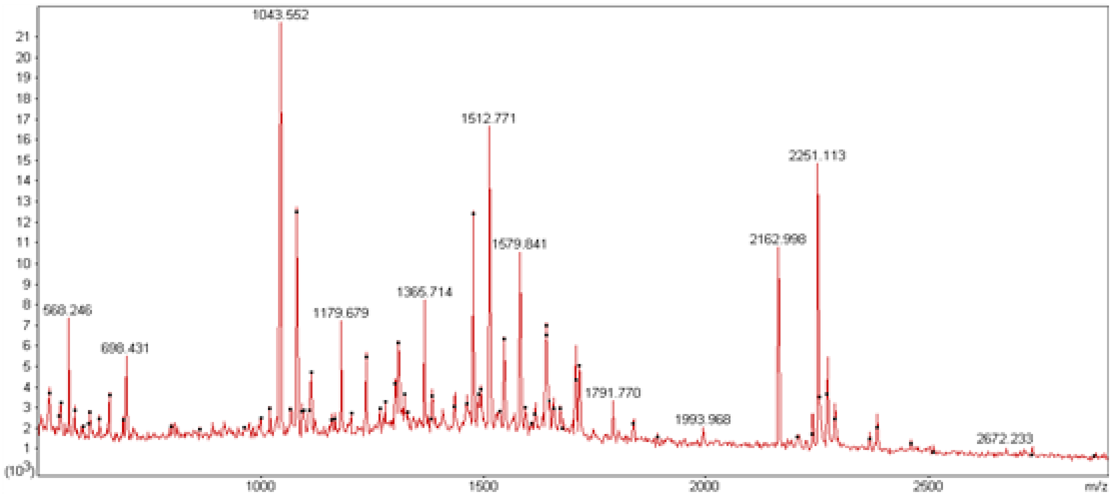

Probability Based Mowse Score:

Protein score is -10*Log(P), where P is the probability that the observed match is a random event.
Protein scores greater than 75 are significant (p<0.05).

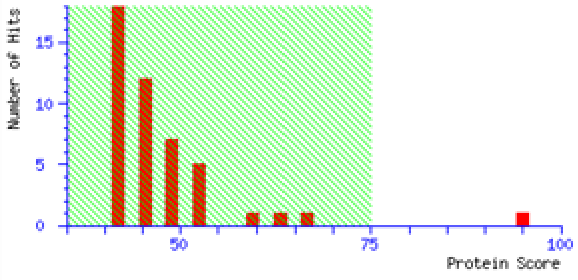

Matched peptide sequences: shown in **Bold Red**

```
  1 MSVGTYSLPA LPYAYDALEP SISAQIMELH HSKHHQAYVT NLNAALKNYA
 51 TATSTNDIAG QIALQSAIKF NGGGHINHSL FWENLSPSSS ADAKPESAPT
101 LSAEISKTWG SIQAFQEAFK KTLLGLQGSG WGWLVKDTHG LRIVTTKDQD
151 PVVGGEYLNG KAAYVDNIWK VINWKTAEAR FTGTREDAFK VLRASI
```

Bold red (matched) regions: 1 HSK**HHQAYVT** **NLNAALKNYA**; 51 **TATSTNDIAG** **QIALQSAIKF** **NGGGHINHSL** **FWENLSPSSS** **ADAKPESAPT**; 101 **LSAEISKTWG** **SIQAFQEAFK** **KTLLGLQGSG** **WGWLVKDTHG** **LR**IVTTKDQD; 151 PVVGGEYLNG **KAAYVDNIWK** **VINWKTAEAR** **FTGTR**EDAFK VLRASI

Matched peptides information:

Start	-	End	Observed	Mr(expt)	Mr(calc)	ppm	M	Peptide
34	-	47	1579.8409	1578.8336	1578.8266	4.42	0	K.HHQAYVTNLNAALK.N
48	-	69	2251.1125	2250.1052	2250.1492	-19.5	0	K.NYATATSTNDIAGQIALQSAIK.F
70	-	107	3983.1864	3982.1791	3981.9133	66.8	0	K.FNGGGHINHSLFWENLSPSSSADAKPESAPTLSAEISK.T
108	-	120	1512.7708	1511.7635	1511.7409	15.0	0	K.TWGSIQAFQEAFK.K
108	-	121	1640.8549	1639.8476	1639.8358	7.19	1	K.TWGSIQAFQEAFKK.T
122	-	136	1614.8704	1613.8631	1613.8930	-18.5	0	K.TLLGLQGSGWGWLVK.D
137	-	142	698.4314	697.4241	697.3507	105	0	K.DTHGLR.I
162	-	170	1079.5939	1078.5866	1078.5447	38.8	0	K.AAYVDNIWK.V
171	-	175	659.4396	658.4323	658.3802	79.0	0	K.VINWK.T
176	-	180	547.3617	546.3544	546.2762	143	0	K.TAEAR.F
181	-	185	581.3940	580.3868	580.2969	155	0	R.FTGTR.E

Spot No.: **7**

Mascot score: **90**

Species: ***Fusarium oxysporum f. sp. cubense race 4***

Protein name: **Phosphoglycerate kinase**

NCBI accession No.: **gi| 475667960** Sequence coverage %: **56**

Matched peptides No.: **18** Total peptides No.: **72**

Calculated Mr: **44835** Calculated *p*I: **5.77**

Annotated PMF **spectra:**

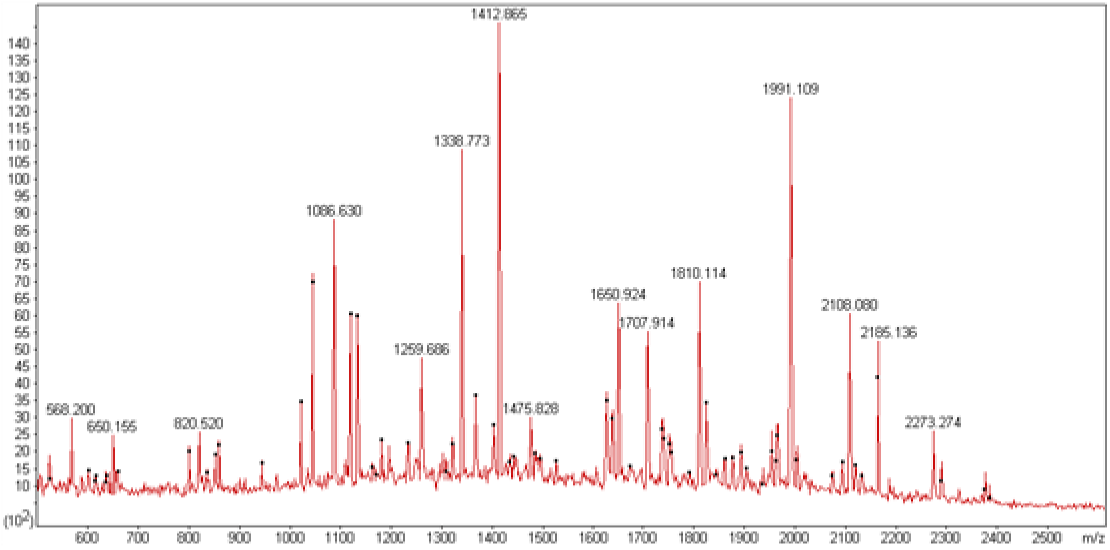

Probability Based Mowse Score:

Protein score is -10*Log(P), where P is the probability that the observed match is a random event.
Protein scores greater than 75 are significant (p<0.05).

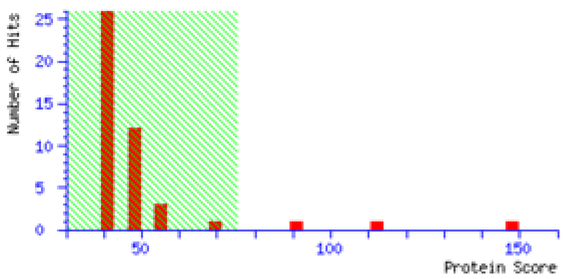

Matched peptide sequences: shown in Bold Red

```
  1 MSLSNKLSIT DVDVKGKRVL IRVDFNVPLD ADKNITNNQR IVGALPTIKY
 51 ALENGAKSVI LMSHLGRPNG SPNEKYSLKP VVPELEKLLG KKVTFAPDCV
101 GPEVEEIVNK AEDGAVILLE NLRFHIEEEG SSKDKEGNKT KADKAQVEAF
151 RKGLTALGDV YINDAFGTAH RAHSSMVGVD LPQKASGFLV KKELEYFAKA
201 LEEPQRPFLA ILGGAKVSDK IQLIDNLLDK VNTLIICGGM AFTFKKTLEG
251 VSIGNSLFDE AGSKTVGNLV EKAKAKGVKL VLPVDYITAD KFDKDANTGY
301 ATDKDGIPDG WQGLDCGEES VKLYKEAIAE AKTILWNGPA GVFEFEKFAS
351 GTKATLDAVV DAVQKDGKIV IIGGGDTATV AKKYGVEDKL SHVSTGGGAS
401 LELLEGKELP GVTALSSK
```

Matched peptides information:

Start	-	End	Observed	Mr(expt)	Mr(calc)	ppm	M	Peptide
23	-	33	1232.6774	1231.6701	1231.6085	50.1	0	R.VDFNVPLDADK.N
34	-	49	1751.9438	1750.9365	1751.0053	-39.3	1	K.NITNNQRIVGALPTIK.Y
58	-	75	1936.0247	1935.0174	1934.9996	9.21	0	K.SVILMSHLGRPNGSPNEK.Y
76	-	87	1401.7975	1400.7902	1400.7915	-0.90	0	K.YSLKPVVPELEK.L
92	-	110	2131.1438	2130.1365	2130.0667	32.8	1	K.KVTFAPDCVGPEVEEIVNK.A
93	-	110	2003.0677	2002.0604	2001.9718	44.3	0	K.VTFAPDCVGPEVEEIVNK.A
111	-	123	1412.8653	1411.8580	1411.7671	64.4	0	K.AEDGAVILLENLR.F
124	-	133	1162.6306	1161.6233	1161.5302	80.2	0	R.FHIEEEGSSK.D
145	-	151	820.5201	819.5129	819.4239	109	0	K.AQVEAFR.K
152	-	171	2119.1825	2118.1752	2118.0858	42.2	1	R.KGLTALGDVYINDAFGTAHR.A
153	-	171	1991.1094	1990.1021	1989.9908	55.9	0	K.GLTALGDVYINDAFGTAHR.A
200	-	216	1810.1135	1809.1062	1809.0148	50.5	0	K.ALEEPQRPFLAILGGAK.V
247	-	264	1823.9783	1822.9710	1822.8949	41.8	0	K.TLEGVSIGNSLFDEAGSK.T
265	-	272	859.5288	858.5215	858.4811	47.1	0	K.TVGNLVEK.A
280	-	294	1736.9988	1735.9915	1735.9396	29.9	1	K.LVLPVDYITADKFDK.D
305	-	322	1961.9684	1960.9611	1960.8473	58.1	0	K.DGIPDGWQGLDCGEESVK.L
333	-	347	1707.9143	1706.9070	1706.8668	23.6	0	K.TILWNGPAGVFEFEK.F
390	-	407	1755.0038	1753.9965	1753.9210	43.0	0	K.LSHVSTGGGASLELLEGK.E

Spot No.: **8**

Mascot score: **90**

Species: ***Fusarium oxysporum f. sp. cubense race 4***

Protein name: **S-adenosylmethionine synthase**

NCBI accession No.: **gi| 475664008** Sequence coverage %: **55**

Matched peptides No.: **16** Total peptides No.: **65**

Calculated Mr: **44513** Calculated *p*I: **5.77**

Annotated PMF **spectra:**

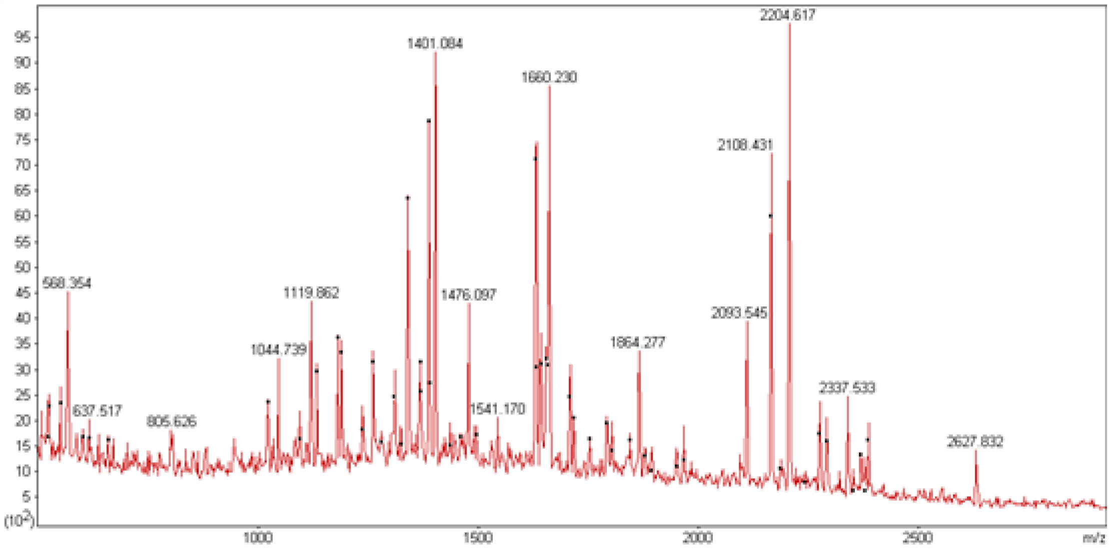

Probability Based Mowse Score:

Protein score is -10*Log(P), where P is the probability that the observed match is a random event.
Protein scores greater than 75 are significant (p<0.05).

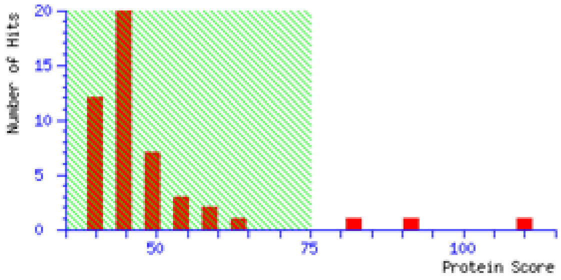

Matched peptide sequences: shown in Bold Red

1 MSATNGTNGV HAELSSWK**HY NEGSFLFTSE SVGEGHPDKI ADQVSDAILD**

51 **ACLR**EDPLSK VACETATK**TG MIMVFGEITT QAKLDYQK**VV RDAIKDIGYD

101 DSAKGFDYK**T CNLLVAIEQQ SPDIAQGLHY EK**ALEQLGAG DQGIMFGYAT

151 DETPELFPLT LLFAHKLNAA MSAAR**RDGSL PWLRPDTK**TQ VTIEYK**HDNG**

201 **AVVPQRVHTV VVSAQHSPDI STEELR**KEIK EKIIKKVIPA K**YLSDETIYH**

251 **IQPSGLFIIG GPQGDAGLTG R**K**IIVDTYGG WGAHGGGAFS GK**DFSKVDRS

301 AAYVGRWIAK SLVNAKLCRR ALVQLSYAIG VAEPLSIYVD SYGTSEK**TSE**

351 **ELVQIIRDNF DLRPGVIVRE LNLDHPIYLQ TAKNGHFGTN QSFSWEQPK**E

401 LKF

Matched peptides information:

Start - End	Observed	Mr(expt)	Mr(calc)	ppm	M	Peptide
19 - 39	2337.5331	2336.5258	2336.0346	210	0	K.HYNEGSFLFTSESVGEGHPDK.I
40 - 54	1660.2298	1659.2225	1658.8298	237	0	K.IADQVSDAILDACLR.E
69 - 83	1627.2119	1626.2046	1625.8157	239	0	K.TGMIMVFGEITTQAK.L
69 - 88	2273.6284	2272.6211	2273.1436	-230	1	K.TGMIMVFGEITTQAKLDYQK.V
69 - 88	2289.6259	2288.6186	2289.1385	-227	1	K.TGMIMVFGEITTQAKLDYQK.V + Oxidation (M)
110 - 132	2627.8322	2626.8249	2626.3061	198	0	K.TCNLLVAIEQQSPDIAQGLHYEK.A
176 - 188	1541.1704	1540.1631	1539.8158	226	1	R.RDGSLPWLRPDTK.T
177 - 188	1385.0441	1384.0368	1383.7147	233	0	R.DGSLPWLRPDTK.T
197 - 206	1092.8272	1091.8199	1091.5472	250	0	K.HDNGAVVPQR.V
207 - 226	2204.6166	2203.6093	2203.1233	221	0	R.VHTVVVSAQHSPDISTEELR.K
242 - 271	3176.2387	3175.2314	3174.5986	199	0	K.YLSDETIYHIQPSGLFIIGGPQGDAGLTGR.K
273 - 292	1950.3641	1949.3568	1948.9432	212	0	K.IIVDTYGGWGAHGGGAFSGK.D
348 - 357	1187.9566	1186.9493	1186.6557	247	0	K.TSEELVQIIR.D
358 - 369	1401.0836	1400.0763	1399.7572	228	0	R.DNFDLRPGVIVR.E
370 - 383	1655.2366	1654.2293	1653.8726	216	0	R.ELNLDHPIYLQTAK.N
384 - 399	1864.2768	1863.2695	1862.8336	234	0	K.NGHFGTNQSFSWEQPK.E

Spot No.: **9**

Mascot score: **170**

Species: ***Fusarium oxysporum f. sp. cubense race 4***

Protein name: **Fructose-bisphosphate aldolase**

NCBI accession No.: **gi| 475673861** Sequence coverage %: **51**

Matched peptides No.: **17** Total peptides No.: **49**

Calculated Mr: **37389** Calculated *p*I: **5.24**

Annotated PMF **spectra:**

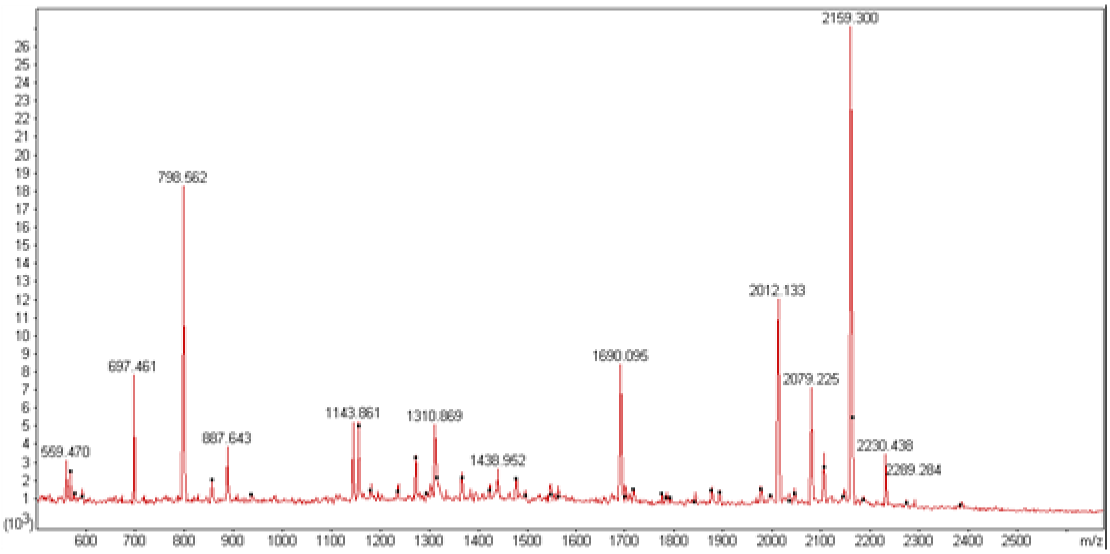

Probability Based Mowse Score:

Protein score is -10*Log(P), where P is the probability that the observed match is a random event. Protein scores greater than 75 are significant (p<0.05).

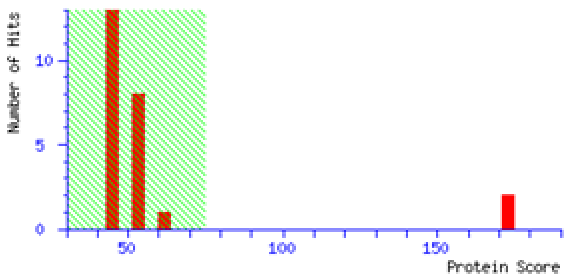

Matched peptide sequences: shown in Bold Red

```
  1 MGVQEVLSRK TGVIVGDDVL RLFEYAREHK FAIPAINVTS SSTVVAALEA
 51 ARDNKAPVIL QFSQGGAAYF AGKGVSNTDQ AASIAGSIAA AHYVRSLAPT
101 YGIPVVLHTD HCAKKLLPWL DGMLDADEAY FKEKGEPLFS SHMIDLSEEP
151 VEWNIETTAK YLKRAAPMKQ WLEMEIGITG GEEDGVNNED VDNNSLYTQP
201 EDILNIYNTL SPISPYFSIA AGFGNVHGVY KPGNVKLHPE LLGKHQAHVK
251 EALKSDNDKP VFFVFHGGSG SSKKEYLDAI GFGVVKVNVD TDMQFAYCSG
301 IRDYMINKRE YVNTTVGNPD GEDKPNKKYF DPRVWVREGE KTMSKRVAEA
351 LQDFNTANQL
```

Matched peptides information:

Start – End	Observed	Mr(expt)	Mr(calc)	ppm	M	Peptide
2 – 9	887.6426	886.6353	886.4872	167	0	M.GVQEVLSR.K
10 – 21	1271.9555	1270.9482	1270.7245	176	1	R.KTGVIVGDDVLR.L
11 – 21	1143.8614	1142.8541	1142.6296	197	0	K.TGVIVGDDVLR.L
22 – 27	798.5619	797.5546	797.4072	185	0	R.LFEYAR.E
74 – 95	2159.2996	2158.2923	2158.0767	99.9	0	K.GVSNTDQAASIAGSIAAAHYVR.S
96 – 114	2079.2248	2078.2175	2078.0619	74.9	0	R.SLAPTYGIPVVLHTDHCAK.K
135 – 160	2975.6335	2974.6262	2974.3906	79.2	0	K.GEPLFSSHMIDLSEEPVEWNIETTAK.Y + Oxidation (M)
255 – 273	2012.1332	2011.1259	2010.9436	90.7	0	K.SDNDKPVFFVFHGGSGSSK.K
274 – 286	1438.9519	1437.9446	1437.7868	110	1	K.KEYLDAIGFGVVK.V
275 – 286	1310.8688	1309.8615	1309.6918	130	0	K.EYLDAIGFGVVK.V
287 – 302	1876.0913	1875.0840	1874.8291	136	0	K.VNVDTDMQFAYCSGIR.D
287 – 302	1892.0401	1891.0328	1890.8241	110	0	K.VNVDTDMQFAYCSGIR.D + Oxidation (M)
310 – 327	1977.1124	1976.1051	1975.9123	97.6	0	R.EYVNTTVGNPDGEDKPNK.K
310 – 328	2105.1994	2104.1921	2104.0073	87.9	1	R.EYVNTTVGNPDGEDKPNKK.Y
329 – 333	697.4610	696.4537	696.3231	188	0	K.YFDPR.V
334 – 337	559.4703	558.4630	558.3278	242	0	R.VWVR.E
346 – 360	1690.0950	1689.0877	1688.8482	142	1	K.RVAEALQDFNTANQL.-

Spot No.: **10**

Mascot score: **122**

Species: ***Fusarium oxysporum Fo5176***

Protein name: **mitochondrial peroxiredoxin PRX1**

NCBI accession No.: **gi| 342888261** Sequence coverage %: **71**

Matched peptides No.: **15** Total peptides No.: **71**

Calculated Mr: **25414** Calculated *p*I: **5.86**

Annotated PMF **spectra:**

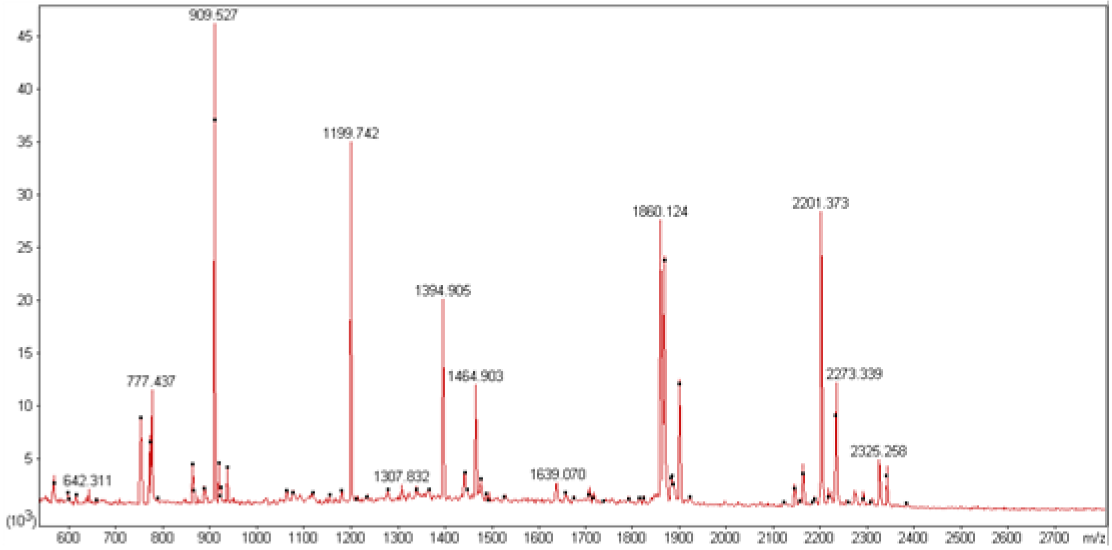

Probability Based Mowse Score:

Protein score is -10*Log(P), where P is the probability that the observed match is a random event.
Protein scores greater than 75 are significant (p<0.05).

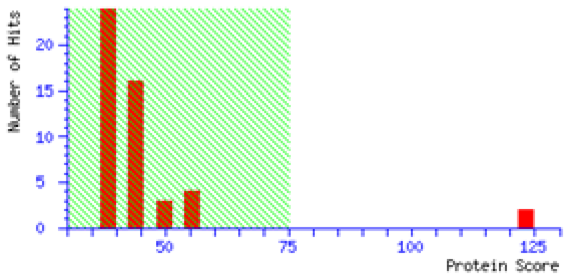

Matched peptide sequences: shown in **Bold Red**

```
  1 MSAPAPLRLG STAPNFQAET TKGKIDFHEF IGDNWVILFS HPEDYTPVCT
 51 TELGAFAKLQ PEFTKRGVKL IGLSANTIES HEGWIKDIGE VTGGNVEFPI
101 IGDKQRQVSL LYDMIDQQDA TNVDEKGIAF TIRSVFIIDP KKTIRTIFSY
151 PASTGRNAAE VLRVIDSLQT GDKYRITTPI NWVPGEDVIV HPSVKNEEAK
201 TLFPEFRIVK PYLRFTPLAK EKVLPPQ
```

Matched peptides information:

Start	-	End	Observed	Mr(expt)	Mr(calc)	ppm	M	Peptide
2	-	22	2157.3226	2156.3153	2156.1226	89.4	1	M.SAPAPLRLGSTAPNFQAETTK.G
9	-	22	1464.9030	1463.8957	1463.7256	116	0	R.LGSTAPNFQAETTK.G
59	-	65	862.4577	861.4504	861.4596	-10.7	0	K.LQPEFTK.R
70	-	86	1868.1964	1867.1891	1866.9839	110	0	K.LIGLSANTIESHEGWIK.D
87	-	104	1860.1240	1859.1167	1858.9313	99.8	0	K.DIGEVTGGNVEFPIIGDK.Q
107	-	126	2325.2584	2324.2511	2324.0842	71.8	0	R.QVSLLYDMIDQQDATNVDEK.G
107	-	126	2341.2715	2340.2642	2340.0791	79.1	0	R.QVSLLYDMIDQQDATNVDEK.G + Oxidation (M)
127	-	133	777.4368	776.4295	776.4545	-32.1	0	K.GIAFTIR.S
134	-	141	918.5604	917.5532	917.5222	33.7	0	R.SVFIIDPK.K
146	-	156	1199.7423	1198.7350	1198.5982	114	0	R.TIFSYPASTGR.N
157	-	163	772.4080	771.4007	771.4239	-30.0	0	R.NAAEVLR.V
164	-	175	1394.9052	1393.8979	1393.7201	128	1	R.VIDSLQTGDKYR.I
176	-	195	2201.3730	2200.3657	2200.1892	80.2	0	R.ITTPINWVPGEDVIVHPSVK.N
201	-	207	909.5272	908.5199	908.4756	48.7	0	K.TLFPEFR.I
208	-	214	888.5714	887.5642	887.5593	5.53	0	R.IVKPYLR.F

Spot No.: **11**

Mascot score: **130**

Species: ***Fusarium oxysporum f. sp. cubense race 4***

Protein name: **S-adenosylmethionine synthetase**

NCBI accession No.: **gi| 475664008**

Sequence coverage %: **48**

Matched peptides No.: **14**

Total peptides No.: **49**

Calculated Mr: **44513**

Calculated *p*I: **5.77**

Annotated PMF **spectra:**

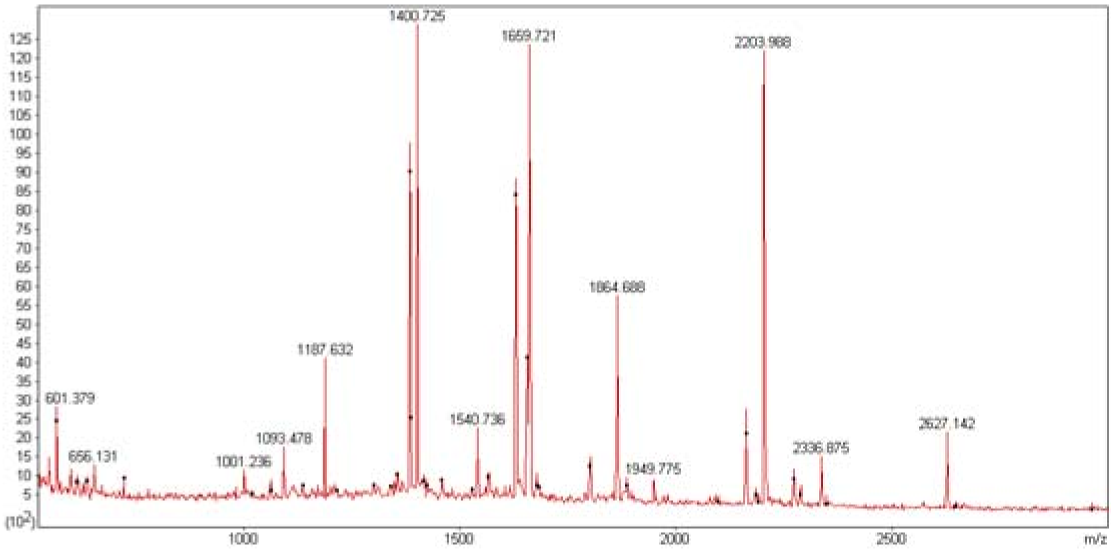

Probability Based Mowse Score:

Protein score is -10*Log(P), where P is the probability that the observed match is a random event.
Protein scores greater than 75 are significant (p<0.05).

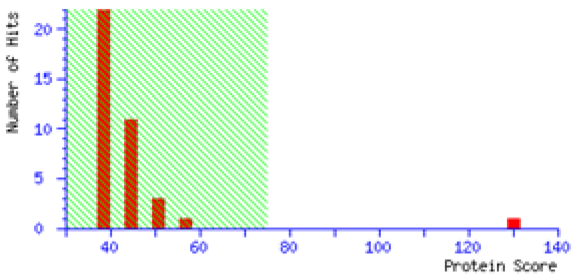

Matched peptide sequences: shown in **Bold Red**

1 MSATNGTNGV HAELSSWK**HY** **NEGSFLFTSE** **SVGEGHPDKI** **ADQVSDAILD**
51 **ACLR**EDPLSK VACETATKTG MIMVFGEITT QAK**LDYQKVV** **R**DAIKDIGYD
101 DSAKGFDYK**T** **CNLLVAIEQQ** **SPDIAQGLHY** **EK**ALEQLGAG DQGIMFGYAT
151 DETPELFPLT LLFAHKLNAA MSAAR**RDGSL** **PWLRPDTK**TQ VTIEYKHDNG
201 AVVPQR**VHTV** **VVSAQHSPDI** **STEELR**KEIK EKIIKKVIPA **KYLSDETIYH**
251 **IQPSGLFIIG** **GPQGDAGLTG** **RKIIVDTYGG** **WGAHGGGAFS** **GK**DFSK**VDRS**
301 **AAYVGR**WIAK SLVNAKLCRR ALVQLSYAIG VAEPLSIYVD SYGTSEK**TSE**
351 **ELVQIIRDNF** **DLRPGVIVRE** **LNLDHPIYLQ** **TAK**NGHFGTN QSFSWEQPKE
401 LKF

Matched peptides information:

Start	-	End	Observed	Mr(expt)	Mr(calc)	ppm	M	Peptide
19	-	39	2336.8748	2335.8675	2336.0346	-71.5	0	K.HYNEGSFLFTSESVGEGHPDK.I
40	-	54	1659.7210	1658.7137	1658.8298	-69.9	0	K.IADQVSDAILDACLR.E
84	-	91	1020.4479	1019.4406	1019.5764	-133	1	K.LDYQKVVR.D
110	-	132	2627.1419	2626.1346	2626.3061	-65.3	0	K.TCNLLVAIEQQSPDIAQGLHYEK.A
176	-	188	1540.7362	1539.7289	1539.8158	-56.4	1	R.RDGSLPWLRPDTK.T
177	-	188	1384.6680	1383.6607	1383.7147	-39.0	0	R.DGSLPWLRPDTK.T
207	-	226	2203.9881	2202.9808	2203.1233	-64.7	0	R.VHTVVVSAQHSPDISTEELR.K
242	-	271	3175.3988	3174.3915	3174.5986	-65.2	0	K.YLSDETIYHIQPSGLFIIGGPQGDAGLTGR.K
273	-	292	1949.7745	1948.7672	1948.9432	-90.3	0	K.IIVDTYGGWGAHGGGAFSGK.D
297	-	306	1093.4779	1092.4706	1092.5676	-88.8	1	K.VDRSAAYVGR.W
300	-	306	723.3985	722.3912	722.3711	27.8	0	R.SAAYVGR.W
348	-	357	1187.6323	1186.6250	1186.6557	-25.9	0	K.TSEELVQIIR.D
358	-	369	1400.7245	1399.7172	1399.7572	-28.6	0	R.DNFDLRPGVIVR.E
370	-	383	1654.7672	1653.7599	1653.8726	-68.1	0	R.ELNLDHPIYLQTAK.N

Spot No.: **12**

Mascot score: **83**

Species: ***Fusarium oxysporum f. sp. cubense race 1***

Protein name: **Beta-hexosaminidase subunit A1**

NCBI accession No.: **gi| 477513214**

Sequence coverage %: **29**

Matched peptides No.: **11**

Total peptides No.: **69**

Calculated Mr: **34198**

Calculated *p*I: **6.25**

Annotated PMF **spectra:**

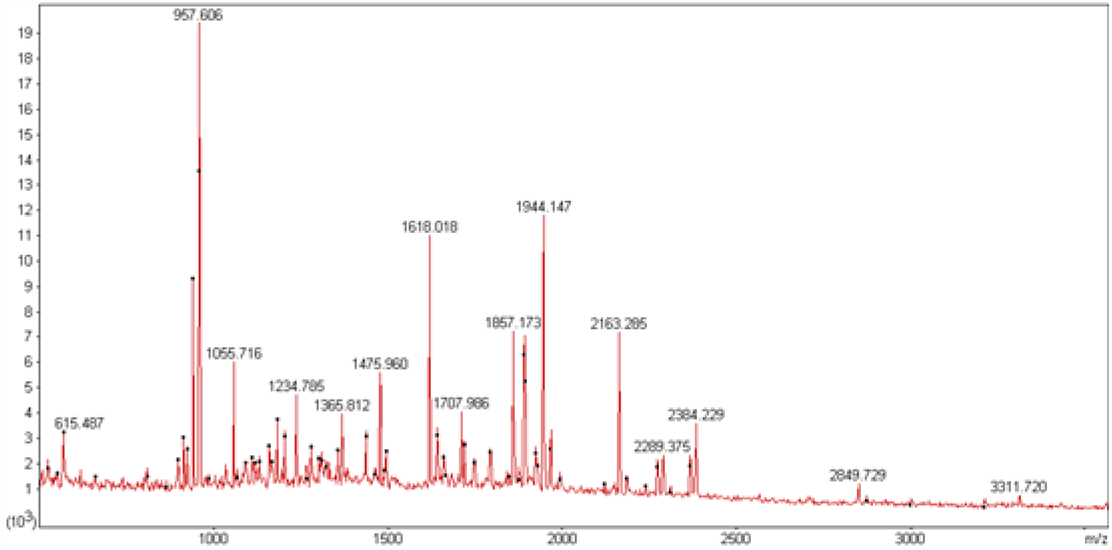

Probability Based Mowse Score:

Protein score is -10*Log(P), where P is the probability that the observed match is a random event.
Protein scores greater than 75 are significant (p<0.05).

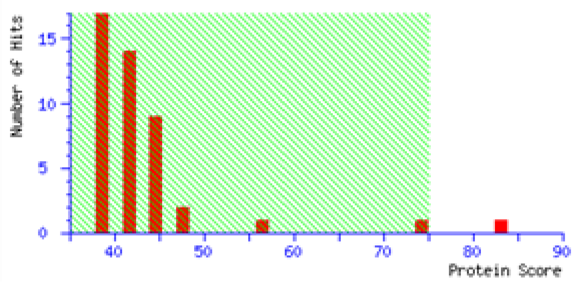

Matched peptide sequences: shown in **Bold Red**

1	M**ASNLYPHRG**	**FMLDTGR**KFF	PVKAILHLLT	LLHQYNFNVF	HWHIYDAESF
51	PLLWPAGEGL	TNASVKYSQT	HTYYTPSDIQ	NVISYAENLG	ILVYPETDMP
101	GHSDIWGIWK	KDLVVGKASL	K**KPDAQLDIR**	QNNK**QVYDYI**	**R**SLVSTVDGY
151	FGSPYHHFGG	DEVAYMWNTR	DDNKLFNSFL	NWLKTLTPK**K**	**SVILWDDPLT**
201	**DSEKSITLSE**	**DWIIQTWHK**G	TTQKILKKGH	R**VIVSESDTF**	**YIGNADADKI**
251	**SSFVFPK**SSK	VLGFEVAWFT	SQDDDPSDLD	QDWIIDPLKA	ASKIRRK

Matched peptides information:

Start	-	End	Observed	Mr(expt)	Mr(calc)	ppm	M	Peptide
2	-	9	957.6061	956.5988	956.4828	121	0	M.ASNLYPHR.G
10	-	17	896.5362	895.5289	895.4222	119	0	R.GFMLDTGR.K
10	-	17	912.5459	911.5386	911.4171	133	0	R.GFMLDTGR.K + Oxidation (M)
122	-	130	1055.7156	1054.7083	1054.5771	124	0	K.KPDAQLDIR.Q
135	-	141	956.6043	955.5970	955.4763	126	0	K.QVYDYIR.S
190	-	204	1746.0961	1745.0888	1744.8883	115	1	K.KSVILWDDPLTDSEK.S
191	-	204	1618.0175	1617.0102	1616.7934	134	0	K.SVILWDDPLTDSEK.S
205	-	219	1857.1735	1856.1662	1855.9468	118	0	K.SITLSEDWIIQTWHK.G
232	-	249	1944.1469	1943.1396	1942.9160	115	0	R.VIVSESDTFYIGNADADK.I
232	-	257	2849.7288	2848.7215	2848.4171	107	1	R.VIVSESDTFYIGNADADKISSFVFPK.S
250	-	257	924.6485	923.6412	923.5117	140	0	K.ISSFVFPK.S

Spot No.: **13**

Mascot score: **76**

Species: ***Fusarium oxysporum f. sp. cubense race 4***

Protein name: **phosphoglycerate kinase**

NCBI accession No.: **gi| 475667960**

Sequence coverage %: **48**

Matched peptides No.: **16**

Total peptides No.: **111**

Calculated Mr: **44835**

Calculated *p*I: **5.77**

Annotated PMF spectra:

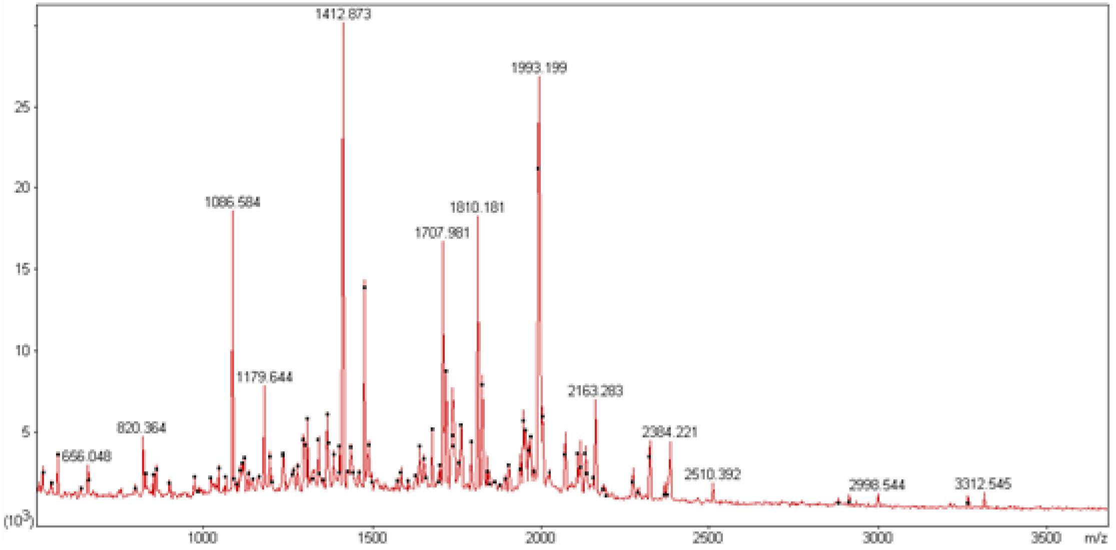

Probability Based Mowse Score:

Protein score is -10*Log(P), where P is the probability that the observed match is a random event.
Protein scores greater than 75 are significant (p<0.05).

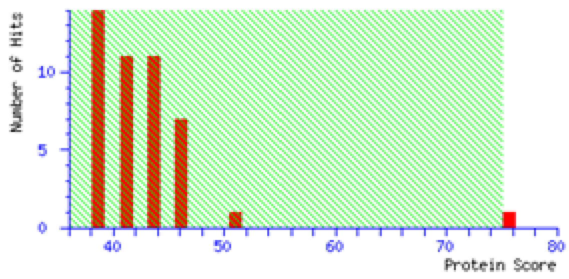

Matched peptide sequences: shown in Bold Red

```
  1 MSLSNKLSIT DVDVKGKRVL IRVDFNVPLD ADKNITNNQR IVGALPTIKY
 51 ALENGAKSVI LMSHLGRPNG SPNEKYSLKP VVPELEKLLG KKVTFAPDCV
101 GPEVEEIVNK AEDGAVILLE NLRFHIEEEG SSKDKEGNKT KADKAQVEAF
151 RKGLTALGDV YINDAFGTAH RAHSSMVGVD LPQKASGFLV KKELEYFAKA
201 LEEPQRPFLA ILGGAKVSDK IQLIDNLLDK VNTLIICGGM AFTFKKTLEG
251 VSIGNSLFDE AGSKTVGNLV EKAKAKGVKL VLPVDYITAD KFDKDANTGY
301 ATDKDGIPDG WQGLDCGEES VKLYKEAIAE AKTILWNGPA GVFEFEKFAS
351 GTKATLDAVV DAVQKDGKIV IIGGGDTATV AKKYGVEDKL SHVSTGGGAS
401 LELLEGKELP GVTALSSK
```

Matched peptides information:

Start	-	End	Observed	Mr(expt)	Mr(calc)	ppm	M	Peptide
23	-	33	1232.6628	1231.6555	1231.6085	38.2	0	R.VDFNVPLDADK.N
23	-	40	2073.2328	2072.2255	2072.0287	95.0	1	R.VDFNVPLDADKNITNNQR.I
34	-	40	859.4081	858.4008	858.4308	-34.9	0	K.NITNNQR.I
76	-	87	1401.8251	1400.8178	1400.7915	18.8	0	K.YSLKPVVPELEK.L
93	-	110	2003.1665	2002.1592	2001.9718	93.6	0	K.VTFAPDCVGPEVEEIVNK.A
111	-	123	1412.8730	1411.8657	1411.7671	69.9	0	K.AEDGAVILLENLR.F
145	-	151	820.3642	819.3569	819.4239	-81.7	0	K.AQVEAFR.K
153	-	171	1991.1904	1990.1831	1989.9908	96.6	0	K.GLTALGDVYINDAFGTAHR.A
172	-	184	1368.7439	1367.7366	1367.6867	36.5	0	R.AHSSMVGVDLPQK.A
193	-	199	899.3770	898.3698	898.4436	-82.2	0	K.ELEYFAK.A
200	-	216	1810.1811	1809.1738	1809.0148	87.9	0	K.ALEEPQRPFLAILGGAK.V
247	-	264	1824.0543	1823.0470	1822.8949	83.5	0	K.TLEGVSIGNSLFDEAGSK.T
295	-	322	2998.5436	2997.5363	2997.2934	81.0	1	K.DANTGYATDKDGIPDGWQGLDCGEESVK.L
305	-	322	1962.0972	1961.0899	1960.8473	124	0	K.DGIPDGWQGLDCGEESVK.L
333	-	347	1707.9813	1706.9740	1706.8668	62.8	0	K.TILWNGPAGVFEFEK.F
390	-	407	1755.0576	1754.0503	1753.9210	73.7	0	K.LSHVSTGGGASLELLEGK.E

Spot No.: **14**

Mascot score: **80**

Species: ***Fusarium oxysporum f. sp. cubense race 1***

Protein name: **beta-hexosaminidase**

NCBI accession No.: **gi| 477513214**

Sequence coverage %: **26**

Matched peptides No.: **10**

Total peptides No.: **68**

Calculated Mr: **34198**

Calculated *p*I: **6.25**

Annotated PMF **spectra:**

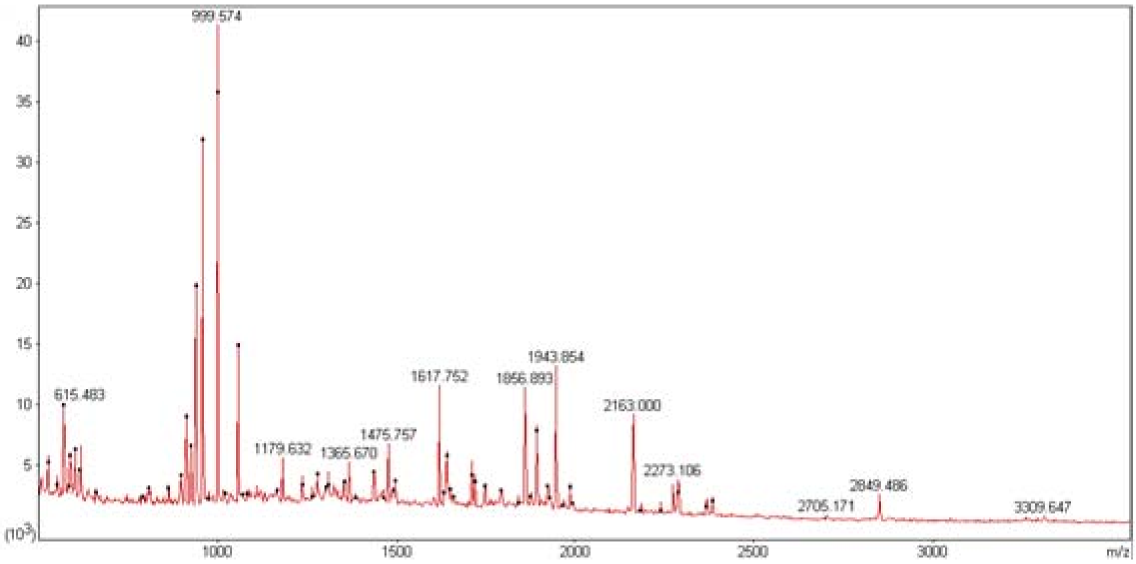

Probability Based Mowse Score:

Protein score is -10*Log(P), where P is the probability that the observed match is a random event. Protein scores greater than 75 are significant (p<0.05).

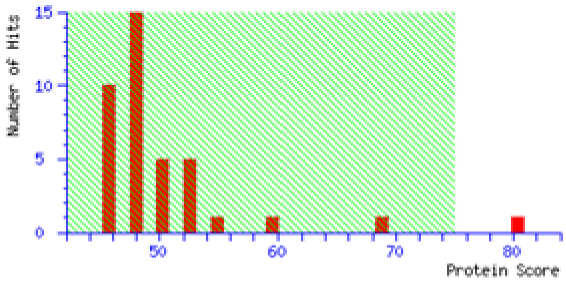

Matched peptide sequences: shown in Bold Red

```
  1 MASNLYPHRG FMLDTGRKFF PVKAILHLLT LLHQYNFNVF HWHIYDAESF
 51 PLLWPAGEGL TNASVKYSQT HTYYTPSDIQ NVISYAENLG ILVYPETDMP
101 GHSDIWGIWK KDLVVGKASL KKPDAQLDIR QNNKQVYDYI RSLVSTVDGY
151 FGSPYHHFGG DEVAYMWNTR DDNKLFNSFL NWLKTLTPKK SVILWDDPLT
201 DSEKSITLSE DWIIQTWHKG TTQKILKKGH RVIVSESDTF YIGNADADKI
251 SSFVFPKSSK VLGFEVAWFT SQDDDPSDLD QDWIIDPLKA ASKIRRK
```

Matched peptides information:

Start	–	End	Observed	Mr(expt)	Mr(calc)	ppm	M	Peptide
10	–	17	896.4766	895.4693	895.4222	52.6	0	R.GFMLDTGR.K
10	–	17	912.4834	911.4761	911.4171	64.7	0	R.GFMLDTGR.K + Oxidation (M)
122	–	130	1055.6256	1054.6183	1054.5771	39.1	0	K.KPDAQLDIR.Q
135	–	141	956.5580	955.5507	955.4763	77.9	0	K.QVYDYIR.S
190	–	204	1745.8459	1744.8386	1744.8883	-28.5	1	K.KSVILWDDPLTDSEK.S
191	–	204	1617.7520	1616.7447	1616.7934	-30.1	0	K.SVILWDDPLTDSEK.S
205	–	219	1856.8935	1855.8862	1855.9468	-32.7	0	K.SITLSEDWIIQTWHK.G
232	–	249	1943.8538	1942.8465	1942.9160	-35.8	0	R.VIVSESDTFYIGNADADK.I
232	–	257	2849.4856	2848.4783	2848.4171	21.5	1	R.VIVSESDTFYIGNADADKISSFVFPK.S
250	–	257	924.5606	923.5533	923.5117	45.1	0	K.ISSFVFPK.S

Spot No.: **15**

Mascot score: **106**

Species: ***Fusarium oxysporum f. sp. cubense race 1***

Protein name: **Superoxide dismutase [Mn], mitochondrial**

NCBI accession No.: **gi| 477512799** Sequence coverage %: **51**

Matched peptides No.: **12** Total peptides No.: **53**

Calculated Mr: **21308** Calculated *p*I: **7.07**

Annotated PMF **spectra:**

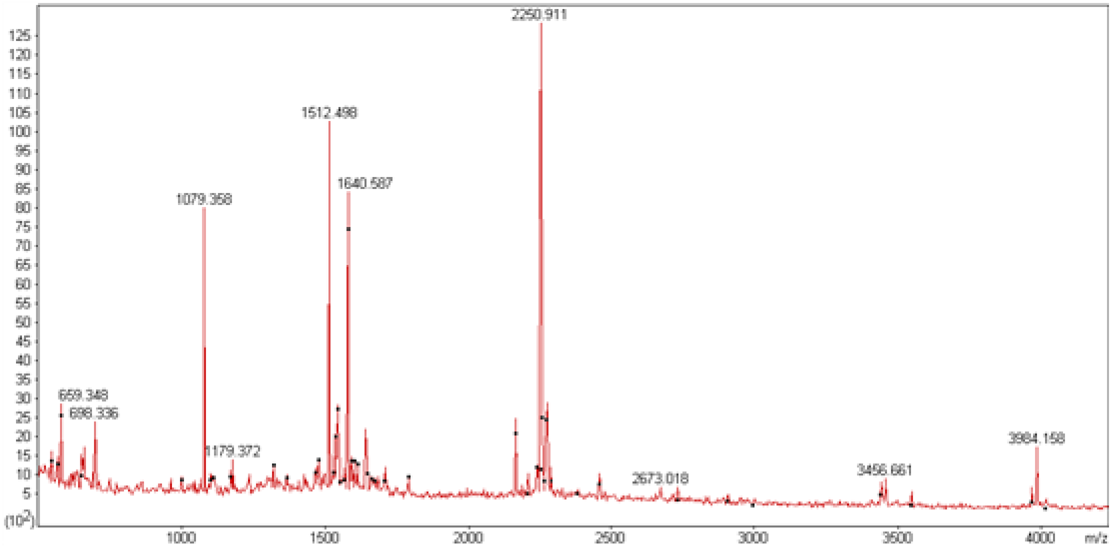

Probability Based Mowse Score:

Protein score is -10*Log(P), where P is the probability that the observed match is a random event.
Protein scores greater than 75 are significant (p<0.05).

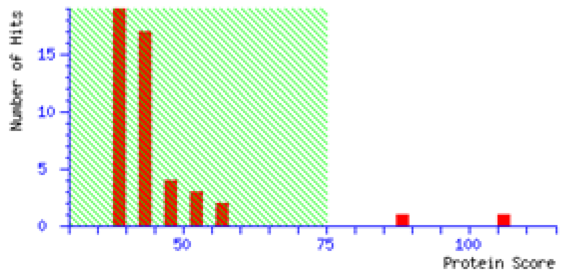

Matched peptide sequences: shown in **Bold Red**

```
  1 MSVGTYSLPA LPYAYDALEP SISAQIMELH HSKHHQAYVT NLNAALKNYA
 51 TATSTNDIAG QIALQSAIKF NGGGHINHSL FWENLSPSSS ADAKPESAPT
101 LSAEISKTWG SIQAFQEAFK KTLLGLQGSG WGWLVKDTHG LRIVTTKDQD
151 PVVGGEYLNG KAAYVDNIWK VINWKTAEAR FTGTREDAFK VLRASI
```

Bold red (matched) residues: 1 … HSK**HHQAYVT** **NLNAALKNYA**; 51 **TATSTNDIAG** **QIALQSAIK**F; 101 LSAEISK**TWG** **SIQAFQEAFK** **KTLLGLQGSG** **WGWLVKDTHG** **LR**IVTTKDQD; 151 PVVGGEYLNG **KAAYVDNIWK** **VINWKTAEAR** **FTGTREDAFK** VLRASI

Matched peptides information:

Start	-	End	Observed	Mr(expt)	Mr(calc)	ppm	M	Peptide
34	-	47	1579.5590	1578.5517	1578.8266	-174	0	K.HHQAYVTNLNAALK.N
48	-	69	2250.9106	2249.9033	2250.1492	-109	0	K.NYATATSTNDIAGQIALQSAIK.F
48	-	69	2251.3287	2250.3214	2250.1492	76.6	0	K.NYATATSTNDIAGQIALQSAIK.F
108	-	120	1512.4980	1511.4907	1511.7409	-165	0	K.TWGSIQAFQEAFK.K
108	-	121	1640.5868	1639.5795	1639.8358	-156	1	K.TWGSIQAFQEAFKK.T
122	-	136	1614.6075	1613.6002	1613.8930	-181	0	K.TLLGLQGSGWGWLVK.D
137	-	142	698.3360	697.3287	697.3507	-31.6	0	K.DTHGLR.I
162	-	170	1079.3579	1078.3506	1078.5447	-180	0	K.AAYVDNIWK.V
171	-	175	659.3476	658.3403	658.3802	-60.6	0	K.VINWK.T
176	-	180	547.3163	546.3090	546.2762	60.1	0	K.TAEAR.F
181	-	185	581.3129	580.3056	580.2969	14.9	0	R.FTGTR.E
181	-	190	1171.3548	1170.3475	1170.5669	-187	1	R.FTGTREDAFK.V

Spot No.: **16**

Mascot score: **78**

Species: ***Fusarium oxysporum***

Protein name: **lactonohydrolase**

NCBI accession No.: **gi| 3810873** Sequence coverage %: **31**

Matched peptides No.: **10** Total peptides No.: **68**

Calculated Mr: **43237** Calculated *p*I: **5.10**

Annotated PMF **spectra:**

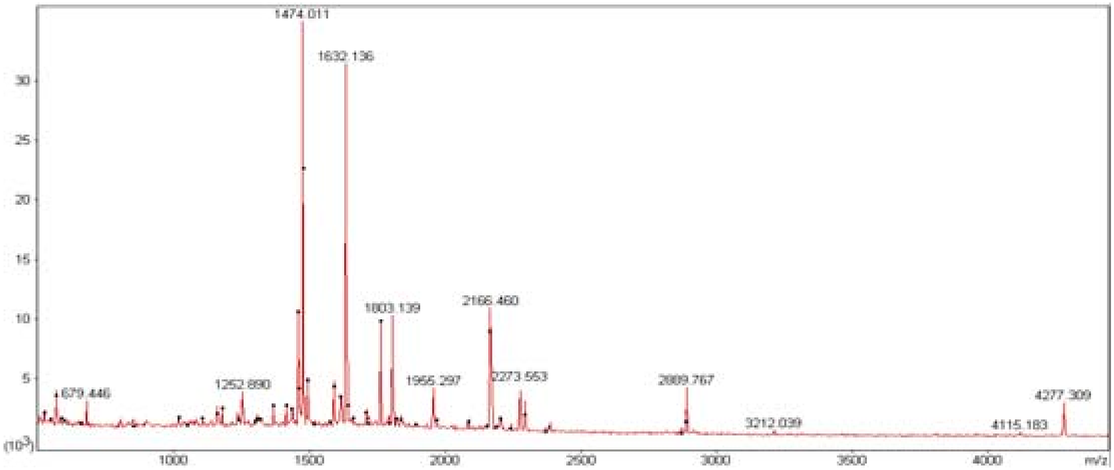

Probability Based Mowse Score:

Protein score is -10*Log(P), where P is the probability that the observed match is a random event.
Protein scores greater than 75 are significant (p<0.05).

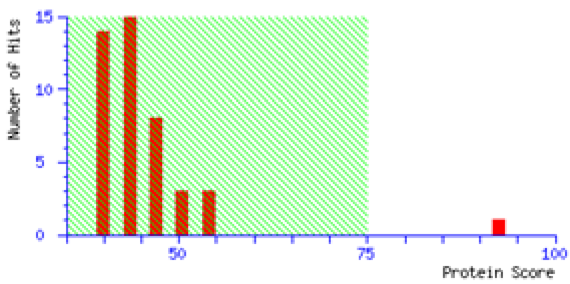

Matched peptide sequences: shown in **Bold Red**

```
  1 MPSSISVLAG VLVPVLGAVA AKLPSTAQII DQKSFNVLKD VPPPAVANDS
 51 LVFTWPGVTE ESLVEKPFHV YDEEFYDVIG KDPSLTLIAT SDTDPIFHEA
101 VVWYPPTEEV FFVQNAGAPA AGTGLNKSSI IQKISLKEAD AVRKGKQDEV
151 KVTVVDSNPQ VINPNGGTYY KGNIIFAGEG QGDDVPSALY LMNPLPPYNT
```

201 TTLLNNYFGR **QFNSLNDVGI** **NPR**NGDLYFT DTLYGYLQDF RPVPGLR**NQV**
251 **YRYNFDTGAV** **TVVADDFTLP** **NGIGFGPDGK** **KVYVTDTGIA** **LGFYGR**NLSS
301 PASVYSFDVN QDGTLQNR**KT** **FAYVASFIPD** **GVHTDSK**GR**V** **YAGCGDGVHV**
351 **WNPSGK**LIGK **IYTGTVAANF** **QFAGK**GRMII TGQTK**LFYVT** **LGASGPK**LYD

Matched peptides information:

Start	-	End	Observed	Mr(expt)	Mr(calc)	ppm	M	Peptide
211	-	223	1474.0106	1473.0033	1472.7372	181	0	R.QFNSLNDVGINPR.N
248	-	252	679.4460	678.4387	678.3449	138	0	R.NQVYR.Y
253	-	280	2887.8779	2886.8706	2886.3713	173	0	R.YNFDTGAVTVVADDFTLPNGIGFGPDGK.K
281	-	296	1760.2485	1759.2412	1758.9305	177	1	K.KVYVTDTGIALGFYGR.N
282	-	296	1632.1362	1631.1289	1630.8355	180	0	K.VYVTDTGIALGFYGR.N
319	-	337	2083.4148	2082.4075	2082.0422	175	1	R.KTFAYVASFIPDGVHTDSK.G
320	-	337	1955.2970	1954.2897	1953.9473	175	0	K.TFAYVASFIPDGVHTDSK.G
340	-	356	1803.1394	1802.1321	1801.8206	173	0	R.VYAGCGDGVHVWNPSGK.L
361	-	375	1588.0773	1587.0700	1586.8093	164	0	K.IYTGTVAANFQFAGK.G
386	-	397	1252.8903	1251.8830	1251.6863	157	0	K.LFYVTLGASGPK.L

Spot No.: **17**

Mascot score: **135**

Species: ***Fusarium oxysporum Fo5176***

Protein name: **serine carboxypeptidase S28**

NCBI accession No.: **gi| 342878892**

Sequence coverage %: **41**

Matched peptides No.: **21**

Total peptides No.: **70**

Calculated Mr: **59955**

Calculated *p*I: **6.04**

Annotated PMF **spectra:**

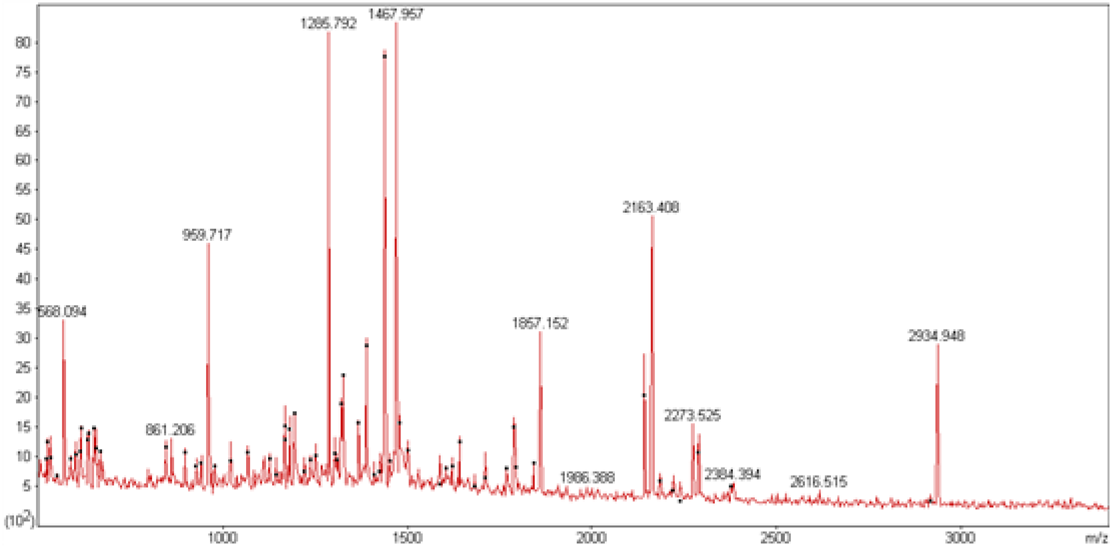

Probability Based Mowse Score:

Protein score is -10*Log(P), where P is the probability that the observed match is a random event. Protein scores greater than 75 are significant (p<0.05).

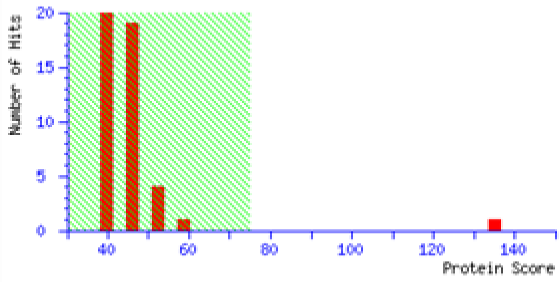

Matched peptide sequences: shown in Bold Red

```
  1 MWFAGCASAA LLALTARGSA YSIPALSARA SSDSGSIKVH NISVPVDHFH
 51 NETKYEPHSD KKFPLRYWFD AQHYREGGPV IILASGETSG EDRIPFLEHG
101 ILKMLANATG GVGVILEHRY YGTSFPVPDL KTKNLRFLST EQALADTAYF
151 AEHVKFPGLE KHNLTASNTP YIIYGGSYAG AFAAFARKIY PEVFWGGISS
201 SGVTEAIIDY WEYFEAARLF APGDCAKVTQ KLTQVVDKIL TGSDKEEKKQ
251 LKIAFGLLGL RDDDFASTIS RGIQGLQGNN WDPAQDSPDF GIYCGSVSSD
301 ALLYASTRPL TPYVKKWLSA HANKNDVKYL TNRFLNYIGY MRSNVESDKQ
351 GGCQGQTVNE CYSIREMYSS TSLNPASSGR QWTYQTCTQW GYWQTGSGVP
401 KNQLPLVSRL VDVEFSTIPC RQEFNITAEP DVESINKLGG WNFSYPRVAF
451 IDGEYDPWRA ATPHKIGLAP RKSTASEPFI LIPYGVHHWD ENGLDPNATE
501 IGLPPPAVAK AQQDIVDFTK AWLEEYEKEK GKRANDL
```

Matched peptides information:

Start	-	End	Observed	Mr(expt)	Mr(calc)	ppm	M	Peptide
2	-	17	1679.1532	1678.1459	1677.8661	167	0	M.WFAGCASAALLALTAR.G
18	-	29	1192.7332	1191.7259	1191.6248	84.9	0	R.GSAYSIPALSAR.A
63	-	66	532.2521	531.2448	531.3169	-136	0	K.FPLR.Y
67	-	75	1285.7917	1284.7844	1284.5676	169	0	R.YWFDAQHYR.E
76	-	93	1787.2184	1786.2111	1785.8745	189	0	R.EGGPVIILASGETSGEDR.I
76	-	103	2934.9482	2933.9409	2933.5498	133	1	R.EGGPVIILASGETSGEDRIPFLEHGILK.M
120	-	131	1386.8980	1385.8907	1385.6867	147	0	R.YYGTSFPVPDLK.T
137	-	155	2141.4238	2140.4165	2140.0477	172	0	R.FLSTEQALADTAYFAEHVK.F
219	-	227	978.5639	977.5566	977.4641	94.7	0	R.LFAPGDCAK.V
253	-	261	959.7170	958.7097	958.5964	118	0	K.IAFGLLGLR.D
262	-	271	1126.6971	1125.6898	1125.4938	174	0	R.DDDFASTISR.G
329	-	333	666.2680	665.2607	665.3497	-134	0	K.YLTNR.F
329	-	342	1840.1351	1839.1278	1838.9137	116	1	K.YLTNRFLNYIGYMR.S + Oxidation (M)
343	-	365	2616.5151	2615.5078	2615.1340	143	1	R.SNVESDKQGGCQGQTVNECYSIR.E
350	-	365	1857.1523	1856.1450	1855.7941	189	0	K.QGGCQGQTVNECYSIR.E
366	-	380	1603.0204	1602.0131	1601.6991	196	0	R.EMYSSTSLNPASSGR.Q + Oxidation (M)
402	-	409	926.5858	925.5785	925.5345	47.6	0	K.NQLPLVSR.L
410	-	421	1435.9761	1434.9688	1434.7177	175	0	R.LVDVEFSTIPCR.Q
448	-	459	1467.9574	1466.9501	1466.6830	182	0	R.VAFIDGEYDPWR.A
521	-	528	1067.5862	1066.5789	1066.4971	76.7	0	K.AWLEEYEK.E
521	-	530	1324.8525	1323.8452	1323.6346	159	1	K.AWLEEYEKEK.G

Spot No.: **18**

Mascot score: **88**

Species: ***Fusarium oxysporum f. sp. cubense race 4***

Protein name: **O-acetylhomoserine (thiol)-lyase-like protein**

NCBI accession No.: **gi| 475675629**

Sequence coverage %: **42**

Matched peptides No.: **13**

Total peptides No.: **52**

Calculated Mr: **47110**

Calculated *p*I: **5.89**

Annotated PMF **spectra:**

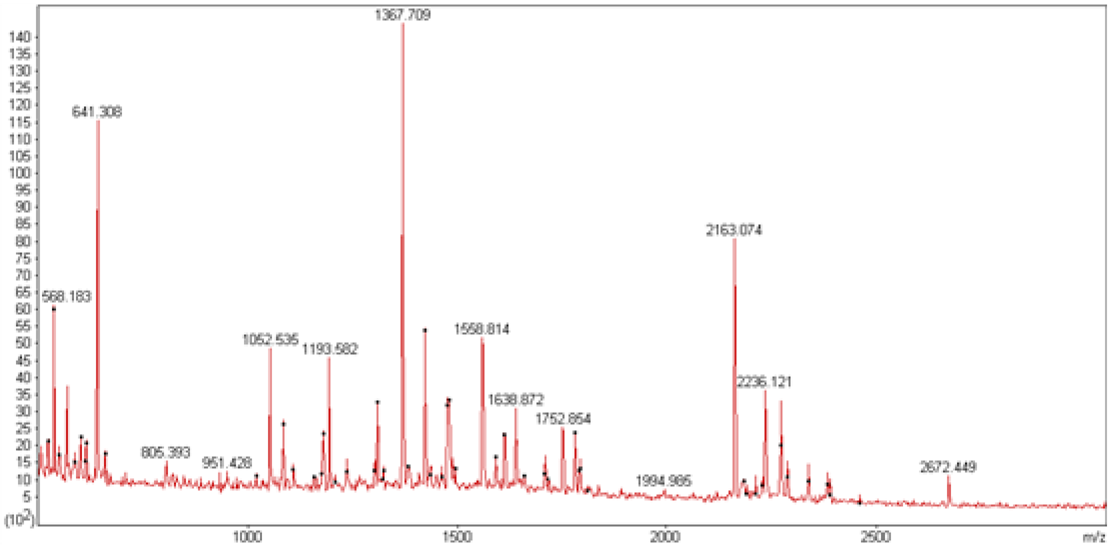

Probability Based Mowse Score:

Protein score is -10*Log(P), where P is the probability that the observed match is a random event.
Protein scores greater than 75 are significant (p<0.05).

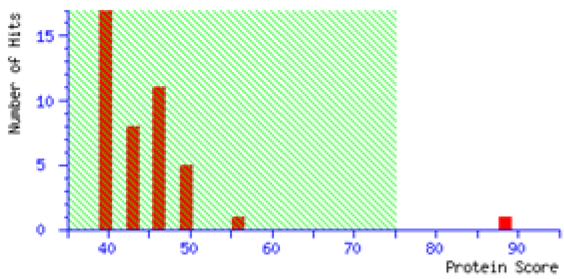

Matched peptide sequences: shown in Bold Red

1 MAEQVFQNFE TLQLHAGYTP DPHTRSTAVP IYATSSYTFN DSAHGARLFG
51 LKELGNIYSR LMNPTVDVFE KRIAALEGGI AAAATSSGQA AQFLTIATLA
101 KAGDNIVASS HLYGGTYNQL NVLLPRFGIK TKFVRSGKLE DYAAAIDDQT
151 RAIYVESMSN PDYVVPDFEG IAKIAHEHGI PLVVDNTLGA GGYYVRPIEH
201 GADIVVHSAT KWIGGHGTTI GGVIVDSGRF NWNKHSDRFP EMVEPSPSYH
251 GLKYWEAFGP ATFITRIRVE MLRDIGACLS PFSAQQLLLG IETLGLRAER
301 HAQNTEKLAK YFESSPNVSW VLWPGSESHP TYAQAKKYLT RGFGAMLSIG
351 VKGDASAGSK VVDGLKLVSN LANVGDAKSL AIHPWSTTHE QLSEDERLAS
401 GVTEDMIRIS VGIEHVDDII ADFEQSFQKA YGS

Matched peptides information:

Start	-	End	Observed	Mr(expt)	Mr(calc)	ppm	M	Peptide
53	-	60	951.4283	950.4210	950.4821	-64.3	0	K.ELGNIYSR.L
61	-	71	1308.6721	1307.6648	1307.6431	16.6	0	R.LMNPTVDVFEK.R + Oxidation (M)
102	-	126	2672.4486	2671.4413	2671.3718	26.0	0	K.AGDNIVASSHLYGGTYNQLNVLLPR.F
136	-	151	1752.8544	1751.8471	1751.8326	8.30	1	R.SGKLEDYAAAIDDQTR.A
139	-	151	1480.7106	1479.7033	1479.6841	13.0	0	K.LEDYAAAIDDQTR.A
152	-	173	2460.2713	2459.2640	2459.1566	43.7	0	R.AIYVESMSNPDYVVPDFEGIAK.I + Oxidation (M)
212	-	229	1781.9500	1780.9427	1780.9221	11.6	0	K.WIGGHGTTIGGVIVDSGR.F
235	-	253	2213.1083	2212.1010	2212.0371	28.9	1	K.HSDRFPEMVEPSPSYHGLK.Y
235	-	253	2229.0744	2228.0671	2228.0320	15.7	1	K.HSDRFPEMVEPSPSYHGLK.Y + Oxidation (M)
254	-	266	1558.8139	1557.8066	1557.7616	28.9	0	K.YWEAFGPATFITR.I
379	-	397	2236.1213	2235.1140	2235.0556	26.1	0	K.SLAIHPWSTTHEQLSEDER.L
398	-	408	1207.5815	1206.5742	1206.5914	-14.3	0	R.LASGVTEDMIR.I + Oxidation (M)
409	-	429	2390.2612	2389.2539	2389.1802	30.9	0	R.ISVGIEHVDDIIADFEQSFQK.A

Spot No.: **19**

Mascot score: **104**

Species: ***Fusarium oxysporum f. sp. cubense race 1***

Protein name: **homoserine dehydrogenase**

NCBI accession No.: **gi| 477517374**

Sequence coverage %: **50**

Matched peptides No.: **12**

Total peptides No.: **38**

Calculated Mr: **38547**

Calculated *p*I: **6.24**

Annotated PMF **spectra:**

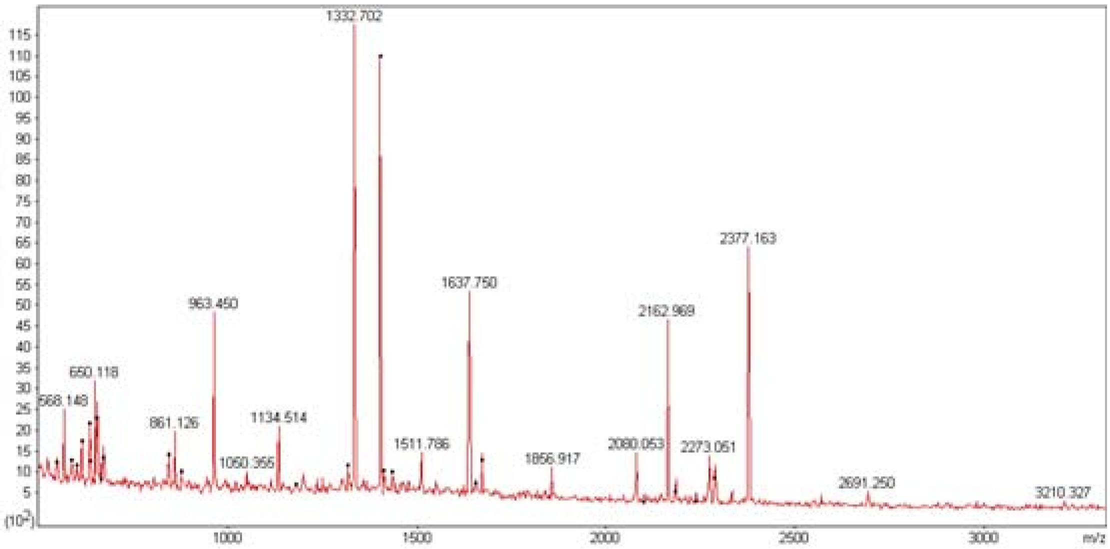

Probability Based Mowse Score:

Protein score is -10*Log(P), where P is the probability that the observed match is a random event.
Protein scores greater than 75 are significant (p<0.05).

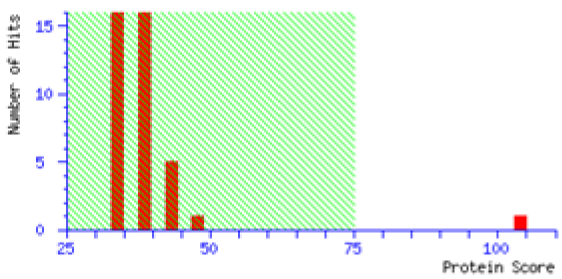

Matched peptide sequences: shown in Bold Red

```
  1 MAAPKQVFIG IIGAGGVGKA FIDQLQSLAA RKPSPKLNLA YIATSRKALF
 51 NDDYSPLNIG NVIETLGSST KAPLALPQVV EYLAKAPAKS VLVDNTSSQD
101 VAELYPLALS RGISIVTPNK KAFSGSYKLW QDIFSAAESS GARVYHESSV
151 GAGLPVISTL KDLVETGDKV TKIEGVFSGT MSFLFNSFAP TEGQGGKWSE
201 EVKKAKSLGY TEPDPRDDLN GLDVARKLTI LARLAGIPVE SPTSFPVQSL
251 IPKELESVSS GDEFLQKLPA FDSQMEETKA AAEKAGKVVR FVGSIDAASK
301 QVKVGLEQFD RSHPIAALKG SDNIISFYTE RYGSNPLIVQ GAGAGGDVTA
351 MGVTADLIKV LSQIA
```

Matched peptides information:

Start - End	Observed	Mr(expt)	Mr(calc)	ppm	M	Peptide
6 - 19	1315.6623	1314.6550	1314.7660	-84.4	0	K.QVFIGIIGAGGVGK.A
20 - 31	1332.7020	1331.6947	1331.7197	-18.8	0	K.AFIDQLQSLAAR.K
72 - 85	1511.7862	1510.7789	1510.8759	-64.2	0	K.APLALPQVVEYLAK.A
90 - 111	2377.1631	2376.1558	2376.2173	-25.9	0	K.SVLVDNTSSQDVAELYPLALSR.G
129 - 143	1637.7497	1636.7424	1636.7845	-25.7	0	K.LWQDIFSAAESSGAR.V
144 - 161	1856.9167	1855.9094	1856.0044	-51.2	0	R.VYHESSVGAGLPVISTLK.D
207 - 216	1134.5143	1133.5070	1133.5353	-24.9	0	K.SLGYTEPDPR.D
234 - 253	2080.0532	2079.0459	2079.1616	-55.6	0	R.LAGIPVESPTSFPVQSLIPK.E
268 - 279	1411.6018	1410.5945	1410.6337	-27.8	0	K.LPAFDSQMEETK.A + Oxidation (M)
304 - 311	963.4501	962.4428	962.4821	-40.9	0	K.VGLEQFDR.S
320 - 331	1401.6396	1400.6323	1400.6572	-17.8	0	K.GSDNIISFYTER.Y
332 - 359	2691.2500	2690.2427	2690.3586	-43.1	0	R.YGSNPLIVQGAGAGGDVTAMGVTADLIK.V + Oxidation (M)

Spot No.: **20**

Mascot score: **121**

Species: ***Fusarium oxysporum f. sp. cubense race 4***

Protein name: **glutathione reductase**

NCBI accession No.: **gi| 475671099** Sequence coverage %: **67**

Matched peptides No.: **19** Total peptides No.: **76**

Calculated Mr: **51031** Calculated *p*I: **6.06**

Annotated PMF **spectra:**

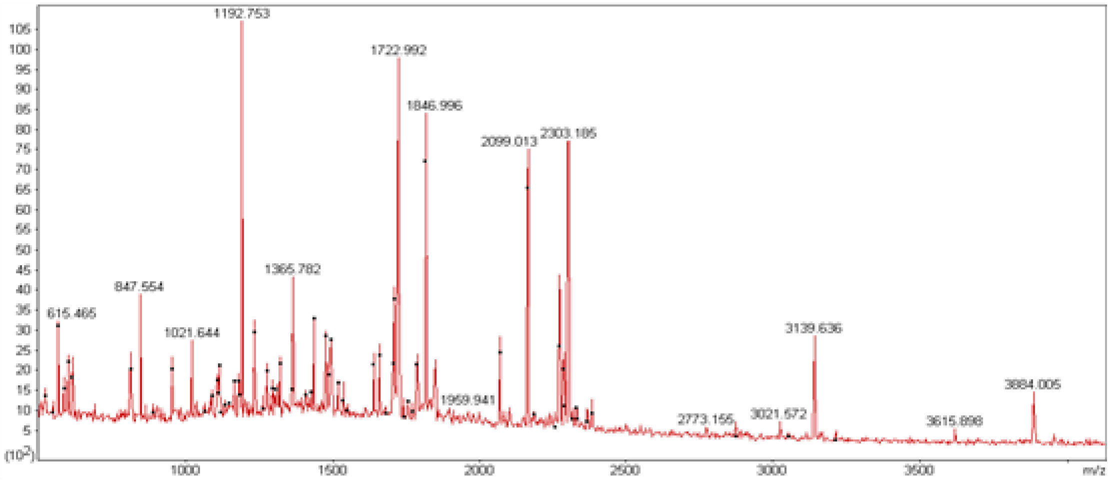

Probability Based Mowse Score:

Protein score is -10*Log(P), where P is the probability that the observed match is a random event.
Protein scores greater than 75 are significant (p<0.05).

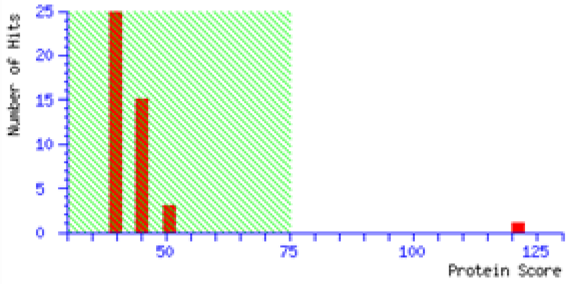

Matched peptide sequences: shown in **Bold Red**

```
  1 MAPITKETDY LVIGGGSGGL ASARMASSKF GVKATIVENK RLGGTCVNVG
 51 CVPKKVTYNA AALAEAIHDA KAYGFSVEQT APFDWSSFKT KRDAYIKRLN
101 GIYERNLNND KVDYLHGWAR LVSKNQAEVT LDDNSKVLVN AKKILVAVGG
151 KPTIPPEIPG AEYGTNSDGF FDISTQPKKV AIVGAGYIAV EFAGMFNALG
201 TETHLFIRYD TFLRNFDPMI QESVTKEYER LGVKLHKRSQ ASKVEKDSNG
251 KLTITYKDDQ GNESVVSDVD NLIWAIGRTP ETKDIGLEEA GVKLGEKGHI
301 LVDEYQNTAV DNIYALGDVT GEVELTPVAI AAGRRLAHRL FGGPEFANLK
351 LDYNNVPSVV FSHPEVGSIG LTEPQAIEKY GKDNIKVYKT SFTAMYYAMM
401 EPEQKGPTNY KLIVAGPEEK VIGLHIMGLG SGEMLQGFGV AVKMGATKAD
451 FDSCVAIHPT SAEELVTLK
```

Matched peptides information:

Start	-	End	Observed	Mr(expt)	Mr(calc)	ppm	M	Peptide
7	-	24	1722.9924	1721.9851	1721.8584	73.6	0	K.ETDYLVIGGGSGGLASAR.M
42	-	54	1360.7744	1359.7671	1359.6639	75.9	0	R.LGGTCVNVGCVPK.K
55	-	71	1786.0142	1785.0069	1784.9420	36.3	1	K.KVTYNAAALAEAIHDAK.A
56	-	71	1657.9265	1656.9192	1656.8471	43.5	0	K.VTYNAAALAEAIHDAK.A
72	-	89	2067.0447	2066.0374	2065.9422	46.1	0	K.AYGFSVEQTAPFDWSSFK.T
106	-	120	1815.0155	1814.0082	1813.8860	67.4	1	R.NLNNDKVDYLHGWAR.L
112	-	120	1116.6739	1115.6666	1115.5512	103	0	K.VDYLHGWAR.L
144	-	178	3615.8984	3614.8911	3614.8508	11.1	0	K.ILVAVGGKPTIPPEIPGAEYGTNSDGFFDISTQPK.K
179	-	208	3211.5416	3210.5343	3210.6900	-48.5	1	K.KVAIVGAGYIAVEFAGMFNALGTETHLFIR.Y + Oxidation (M)
209	-	214	814.5262	813.5189	813.4021	144	0	R.YDTFLR.N
215	-	226	1424.7831	1423.7758	1423.6653	77.6	0	R.NFDPMIQESVTK.E + Oxidation (M)
252	-	278	3021.5723	3020.5650	3020.5091	18.5	1	K.LTITYKDDQGNESVVSDVDNLIWAIGR.T
298	-	334	3884.0048	3882.9975	3882.9640	8.64	0	K.GHILVDEYQNTAVDNIYALGDVTGEVELTPVAIAAGR.R
340	-	350	1192.7531	1191.7458	1191.6288	98.2	0	R.LFGGPEFANLK.L
351	-	379	3139.6359	3138.6286	3138.5873	13.2	0	K.LDYNNVPSVVFSHPEVGSIGLTEPQAIEK.Y
387	-	405	2334.1498	2333.1425	2333.0418	43.2	1	K.VYKTSFTAMYYAMMEPEQK.G + Oxidation (M)
390	-	405	1959.9406	1958.9333	1958.8100	63.0	0	K.TSFTAMYYAMMEPEQK.G + 2 Oxidation (M)
412	-	420	955.6442	954.6369	954.5386	103	0	K.LIVAGPEEK.V
449	-	469	2303.1845	2302.1772	2302.1151	27.0	0	K.ADFDSCVAIHPTSAEELVTLK.-

Spot No.: **21**

Mascot score: **142**

Species: ***Fusarium oxysporum f. sp. cubense race 4***

Protein name: **Hsp 70 kda**

NCBI accession No.: **gi| 475663822**

Sequence coverage %: **44**

Matched peptides No.: **23**

Total peptides No.: **70**

Calculated Mr: **70951**

Calculated *p*I: **5.00**

Annotated PMF spectra:

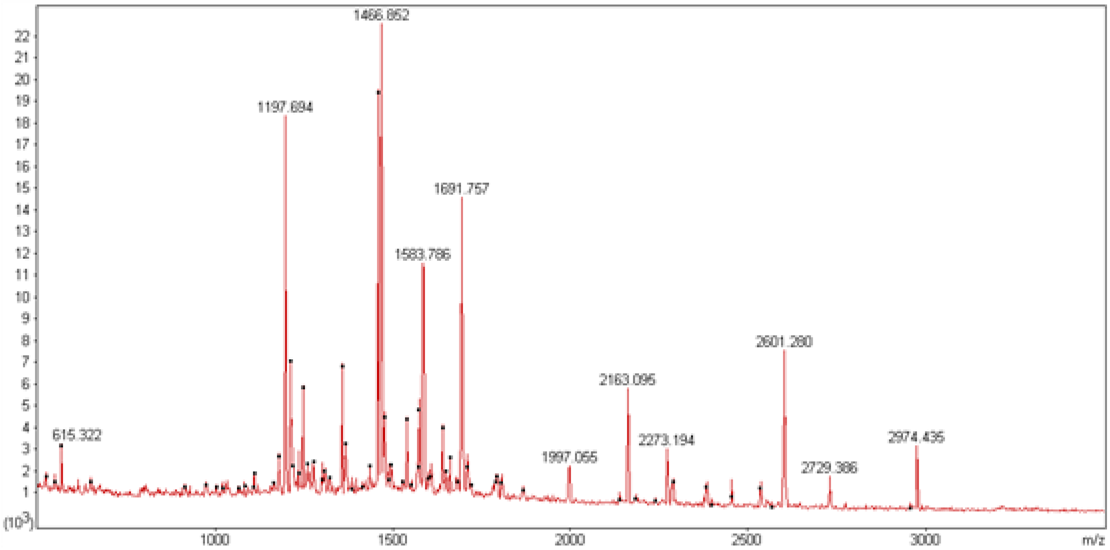

Probability Based Mowse Score:

Protein score is -10*Log(P), where P is the probability that the observed match is a random event.
Protein scores greater than 75 are significant (p<0.05).

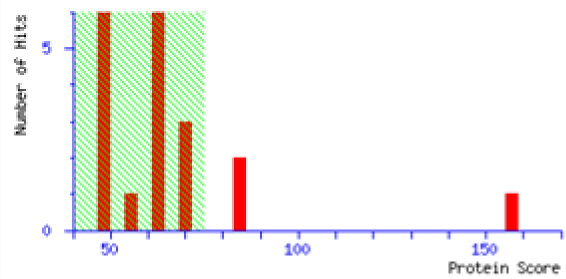

Matched peptide sequences: shown in Bold Red

```
  1 MAPAVGIDLG TTYSCVGIFR EDRCDIIAND QGNRTTPSFV GFTDTERLIG
 51 DAAKNQVAMN PQNTVFDAKR LIGRKFADPE VQADMKHFPF KIVDKGGKPN
101 IEVEFKGETK TFTPEEISAM ILTKMRETAE SYLGETVNNA VVTVPAYFND
151 SQRQATKDAG LIAGLNVLRI INEPTAAAIA YGLDKKVEGE RNVLIFDLGG
201 GTFDVSLLTI EEGIFEVKST AGDTHLGGED FDNRLVNHFV NEFKRKHKKD
251 LSTNVRALRR LRTACERAKR TLSSSAQTSI EIDSLFEGID FYTSITRARF
301 EELCQDLFRS TPVDRVLTDA KIDKSLVHEI VLVGGSTRIP RVQKLITDYF
351 NGKEPNKSIN PDEAVAYGAA VQAAILSGDT SSKATNEILL LDVAPLSLGI
401 ETAGGMMTKL IPRNTTIPTK KSEVFSTFSD NQPGVLIQVY EGERQRTKDN
451 NLMGKFELTG IPPAPRGVPQ IEVTFDLDAN GIMNVSAVEK GTGKSNKIVI
501 TNDKGRLSKE EIERMLNDAE KYKEEDEAEG KRVAAKNGLE SYAYSLRNTL
551 SDPKVEEKIE ASDKETLTAE IDKVVQWLDD NQQATREEYE EHQKELEGKA
601 NPIMMKFYGA GGEGAPGGMP GGPGGFPGAG GPGGAPGAGG DDGPTVEEVD
```

Matched peptides information:

Start	-	End	Observed	Mr(expt)	Mr(calc)	ppm	M	Peptide
2	-	20	1997.0549	1996.0476	1996.0088	19.4	0	M.APAVGIDLGTTYSCVGIFR.E
2	-	23	2397.2212	2396.2139	2396.1795	14.4	1	M.APAVGIDLGTTYSCVGIFREDR.C
24	-	34	1275.6350	1274.6277	1274.5673	47.4	0	R.CDIIANDQGNR.T
35	-	47	1457.7042	1456.6969	1456.6835	9.25	0	R.TTPSFVGFTDTER.L
96	-	106	1217.6469	1216.6396	1216.6452	-4.56	0	K.GGKPNIEVEFK.G
111	-	124	1596.8173	1595.8100	1595.8116	-1.02	0	K.TFTPEEISAMILTK.M + Oxidation (M
127	-	153	2974.4348	2973.4275	2973.3992	9.53	0	R.ETAESYLGETVNNAVVTVPAYFNDSQR.Q
158	-	169	1211.7432	1210.7359	1210.7034	26.9	0	K.DAGLIAGLNVLR.I
170	-	185	1659.8865	1658.8792	1658.8879	-5.23	0	R.IINEPTAAAIAYGLDK.K
170	-	186	1787.9515	1786.9442	1786.9828	-21.6	1	R.IINEPTAAAIAYGLDKK.V
219	-	234	1691.7573	1690.7500	1690.7183	18.8	0	K.STAGDTHLGGEDFDNR.L
235	-	244	1246.6771	1245.6698	1245.6506	15.4	0	R.LVNHFVNEFK.R
298	-	309	1583.7856	1582.7783	1582.7562	14.0	1	R.ARFEELCQDLFR.S
300	-	309	1356.6598	1355.6525	1355.6180	25.5	0	R.FEELCQDLFR.S
325	-	338	1466.8520	1465.8447	1465.8253	13.3	0	K.SLVHEIVLVGGSTR.I
358	-	383	2535.3093	2534.3020	2534.2500	20.5	0	K.SINPDEAVAYGAAVQAAILSGDTSSK.A
421	-	444	2729.3865	2728.3792	2728.3344	16.4	1	K.KSEVFSTFSDNQPGVLIQVYEGER.Q
422	-	444	2601.2798	2600.2725	2600.2395	12.7	0	K.SEVFSTFSDNQPGVLIQVYEGER.Q
447	-	455	1020.5355	1019.5282	1019.5070	20.9	1	R.TKDNNLMGK.F
456	-	466	1197.6939	1196.6866	1196.6553	26.1	0	K.FELTGIPPAPR.G
507	-	514	1003.4869	1002.4796	1002.5345	-54.8	1	R.LSKEEIER.M
574	-	586	1572.7760	1571.7687	1571.7692	-0.32	0	K.VVQWLDDNQQATR.E
587	-	599	1647.7699	1646.7626	1646.7423	12.3	1	R.EEYEEHQKELEGK.A

Spot No.: **22**

Mascot score: **129**

Species: ***Fusarium oxysporum f. sp. cubense race 4***

Protein name: **Enolase**

NCBI accession No.: **gi| 475668982**

Sequence coverage %: **57**

Matched peptides No.: **22**

Total peptides No.: **77**

Calculated Mr: **47469**

Calculated *p*I: **5.06**

Annotated PMF **spectra:**

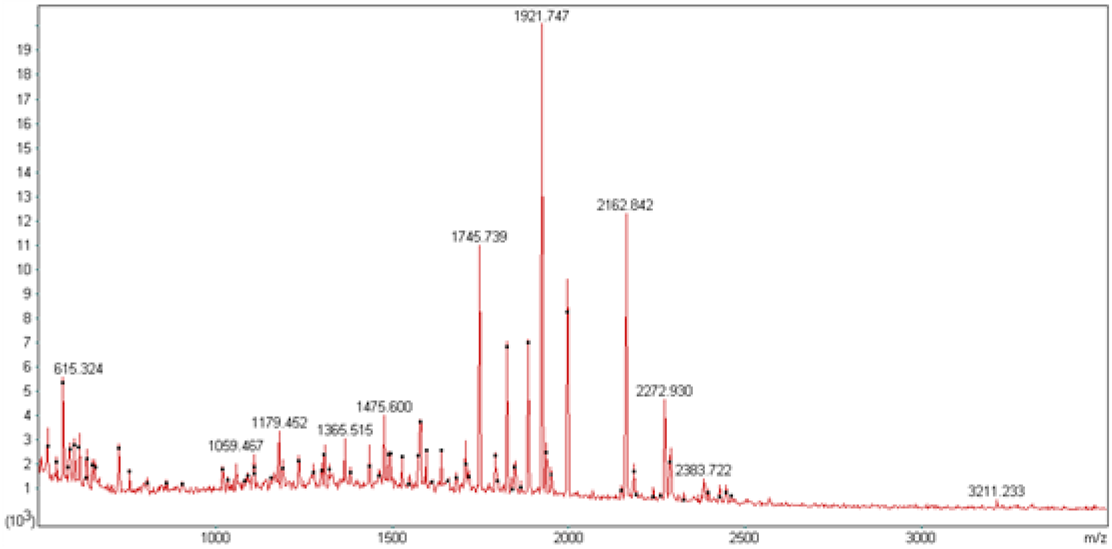

Probability Based Mowse Score:

Protein score is -10*Log(P), where P is the probability that the observed match is a random event.
Protein scores greater than 75 are significant (p<0.05).

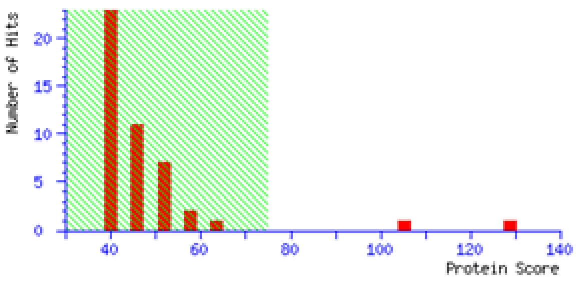

Matched peptide sequences: shown in Bold Red

```
  1 MAVKKVFARS VYDSRGNPTV EVDVVTETGL HRAIVPSGAS TGQHEACELR
 51 DGDKSKWGGK GVTKAVENVN TVIAPALIEK NLDVKDQSAV DAFLNELDGT
101 PNKTKLGANA ILGVSLAVAK AGAAEKGVPL YAHVSDLAGT KKPYVLPVPF
151 MNVLNGGSHA GGRLAFQEFM IVPTEAPTFT EAMRQGAEVY QALKGLAKKR
201 YGQSAGNVGD EGGVAPDIQT AEEALELITD AIEQVGYTGK IKIAMDVASS
251 EFYKVEEKKY DLDFKNPESD PTKWITYEEL ANLYSELCKK YPIVSIEDPF
301 AEDDWEAWSY FSKTQDIQIV GDDLTVTNPL RIKKAIELKS CNALLLKVNQ
351 IGTLTESIQA AKDSYADGWG VMVSHRSGET EDVTIADIAV GLRAGEIKTG
401 APARSERLAK LNQILRIEEE LGDQAIYPGA NFRKSVNL
```

Matched peptides information:

Start – End	Observed	Mr(expt)	Mr(calc)	ppm	M	Peptide
10 – 15	726.3128	725.3055	725.3344	-39.9	0	R.SVYDSR.G
16 – 32	1822.7604	1821.7531	1821.9221	-92.8	0	R.GNPTVEVDVVTETGLHR.A
33 – 50	1882.7346	1881.7273	1881.9003	-91.9	0	R.AIVPSGASTGQHEACELR.D
65 – 80	1680.7465	1679.7392	1679.9458	-123	0	K.AVENVNTVIAPALIEK.N
86 – 103	1933.7213	1932.7140	1932.9065	-99.6	0	K.DQSAVDAFLNELDGTPNK.T
86 – 105	2162.8417	2161.8344	2162.0491	-99.3	1	K.DQSAVDAFLNELDGTPNKTK.L
127 – 141	1527.6143	1526.6070	1526.8093	-132	0	K.GVPLYAHVSDLAGTK.K
142 – 163	2326.8202	2325.8129	2325.2052	261	0	K.KPYVLPVPFMNVLNGGSHAGGR.L + Oxidation (M)
164 – 184	2428.8829	2427.8756	2428.1807	-126	0	R.LAFQEFMIVPTEAPTFTEAMR.Q
164 – 184	2444.9157	2443.9084	2444.1756	-109	0	R.LAFQEFMIVPTEAPTFTEAMR.Q + Oxidation (M)
164 – 184	2460.8860	2459.8787	2460.1705	-119	0	R.LAFQEFMIVPTEAPTFTEAMR.Q + 2 Oxidation (M)
185 – 198	1475.5997	1474.5924	1474.8143	-150	1	R.QGAEVYQALKGLAK.K
243 – 258	1845.6991	1844.6918	1844.8866	-106	1	K.IAMDVASSEFYKVEEK.K
243 – 258	1861.6670	1860.6597	1860.8815	-119	1	K.IAMDVASSEFYKVEEK.K + Oxidation (M)
314 – 331	1997.8631	1996.8558	1997.0430	-93.7	0	K.TQDIQIVGDDLTVTNPLR.I
314 – 333	2238.8872	2237.8799	2238.2220	-153	1	K.TQDIQIVGDDLTVTNPLRIK.K
348 – 362	1572.6496	1571.6423	1571.8519	-133	0	K.VNQIGTLTESIQAAK.D
363 – 376	1579.5457	1578.5384	1578.6885	-95.1	0	K.DSYADGWGVMVSHR.S
363 – 376	1595.5153	1594.5080	1594.6835	-110	0	K.DSYADGWGVMVSHR.S + Oxidation (M)
377 – 393	1745.7390	1744.7317	1744.8843	-87.4	0	R.SGETEDVTIADIAVGLR.A
411 – 416	756.3762	755.3689	755.4653	-128	0	K.LNQILR.I
417 – 433	1921.7475	1920.7402	1920.9217	-94.5	0	R.IEEELGDQAIYPGANFR.K

Spot No.: **23**

NCBI accession No.: **gi|342882947**

Plant species: ***Fusarium oxysporum Fo5176***

Protein name：**vacuolar protease A**

Mascot score: **218** Sequence coverage %: **10**

The number of matched peptides with p≤0.05: **6**

Calculated Mr: **42978** Calculated *p*I: **6.66**

Annotated MS spectra:

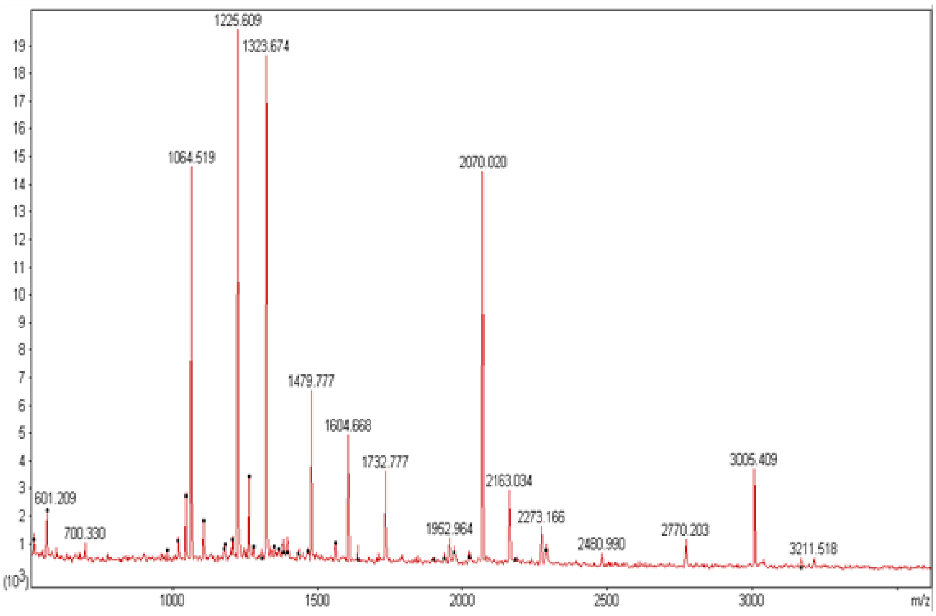

Probability Based Mowse Score:

Mascot Score Histogram

Ions score is -10*Log(P), where P is the probability that the observed match is a random event.
Individual ions scores > 48 indicate identity or extensive homology (p<0.05).
Protein scores are derived from ions scores as a non-probabilistic basis for ranking protein hits.

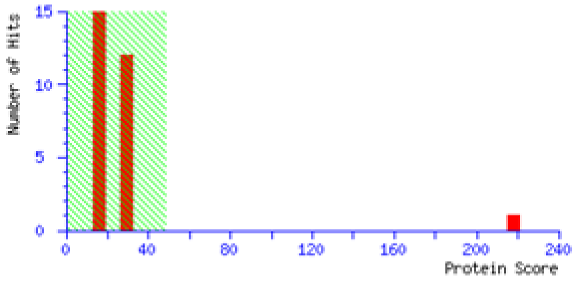

Matched peptide sequences: shown in Bold Red

```
  1  MKGALLTAAA  LLGSAQAGVH  KMKLNKVPLA  DQLATNSVED  HLQSLGQKYL
 51  GASRPKNAAD  YAFATNTVNV  EGGHPVPVSN  FMNAQYFSEI  TIGTPPQSFK
101  VVLDTGSSNL  WVPSQQCGSI  ACYLHSKYDS  SASSTYKENG  TEFEIHYGSG
151  SLSGFVSNDV  VSIGDLEIKD  QDFAEATKEP  GLAFAFGRFD  GILGLGYDRI
201  AVNGMVPPFY  QMVNQKLLDE  PVFAFYLDDQ  EGESEATFGG  IDKSKFTGDI
251  EYIPLRRKAY  WEVDLEAIAF  GDEVAEQENT  GAILDTGTSL  NVLPSALAEL
301  LNKEIGAKKG  YNGQYTIECD  KRASLPDITF  NLAGSNYSLP  ATDYILEVQG
351  SCISTFQGMD  FPEPVGPLVI  LGDAFLRRYY  SVYDLGKNAV  GLARAK
```

Matched peptides information:

Query	Start - End	Observed	Mr(expt)	Mr(calc)	ppm	M	Score	Expect	Rank	U	Peptide
[illegible]	170 - 188	2070.0200	2069.0127	2068.9854	13.2	1	108	4.3e-09	1	U	K.DQDFAEATKEPGLAFAFGR.F
[illegible]	170 - 188	2070.0200	2069.0127	2068.9854	13.2	1	108	4.3e-09	1	U	K.DQDFAEATKEPGLAFAFGR.F
[illegible]	189 - 199	1225.6090	1224.6017	1224.6139	-9.94	0	48	0.057	1	U	R.FDGILGLGYDR.I
[illegible]	189 - 199	1225.6090	1224.6017	1224.6139	-9.94	0	48	0.057	1	U	R.FDGILGLGYDR.I
[illegible]	246 - 256	1323.6740	1322.6667	1322.6870	-15.4	0	62	0.0022	1	U	K.FTGDIEYIPLR.R
[illegible]	246 - 256	1323.6740	1322.6667	1322.6870	-15.4	0	62	0.0022	1	U	K.FTGDIEYIPLR.R

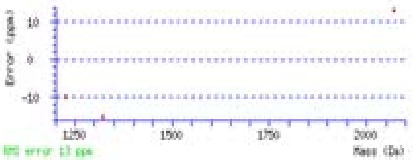

Annotated ion spectra of the matched peptides with p≤0.05:

CID No.: **23-1225.6**

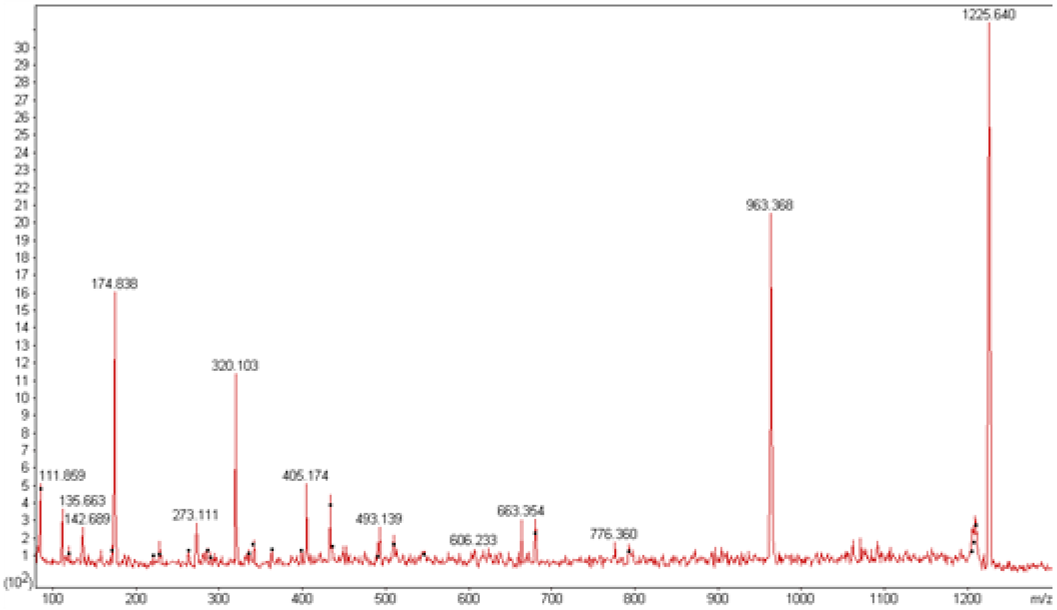

MS/MS Fragmentation of **R.FDGILGLGYDR.I**

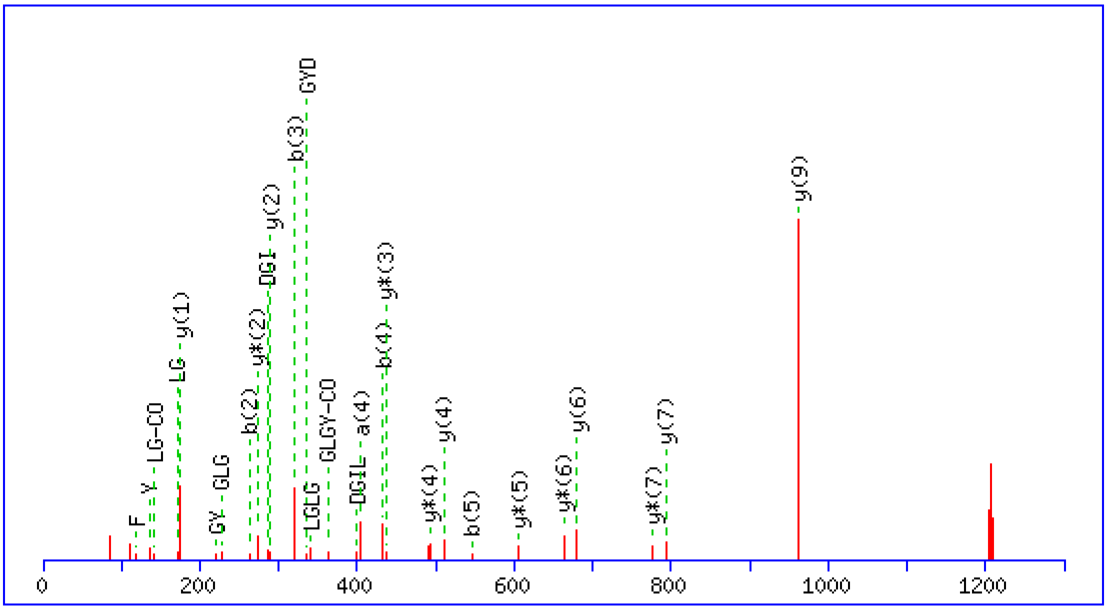

Monoisotopic mass of neutral peptide Mr(calc): 1224.6139

#	Immon.	a	a^0	b	b^0	d	d'	Seq.	v	w	w'	y	y*	y^0	#
1	120.0808	**120.0808**		148.0757		44.0495		F							11
2	88.0393	235.1077	217.0972	**263.1026**	245.0921	191.1179		D	1018.5316	1017.5364		1078.5528	1061.5262	1060.5422	10
3	30.0338	292.1292	274.1186	**320.1241**	302.1135			G				***963.5258***	946.4993	945.5152	9
4	86.0964	**405.2132**	387.2027	**433.2082**	415.1976	377.1819	391.1976	I	848.4261	861.4465	875.4621	906.5043	889.4778	888.4938	8
5	86.0964	518.2973	500.2867	**546.2922**	528.2817	476.2504		L	735.3420	734.3468		***793.4203***	776.3937	775.4097	7
6	30.0338	575.3188	557.3082	603.3137	585.3031			G				***680.3362***	663.3097	662.3257	6
7	86.0964	688.4028	670.3923	716.3978	698.3872	646.3559		L	565.2365	564.2413		623.3148	606.2882	605.3042	5
8	30.0338	745.4243	727.4137	773.4192	755.4087			G				***510.2307***	493.2041	492.2201	4
9	136.0757	908.4876	890.4771	936.4825	918.4720			Y	345.1517			453.2092	436.1827	435.1987	3
10	88.0393	1023.5146	1005.5040	1051.5095	1033.4989	979.5247		D	230.1248	229.1295		***290.1459***	273.1193	272.1353	2
11	129.1135							R	74.0237	73.0284		***175.1190***	158.0924		1

Seq	ya	yb	Seq	ya	yb	Seq	ya	yb
DG	145.0608	173.0557	DGI	258.1448	286.1397	DGIL	371.2289	399.2238
DGILG	428.2504	456.2453	DGILGL	541.3344	569.3293	DGILGLG	598.3559	626.3508
GI	143.1179	171.1128	GIL	256.2020	284.1969	GILG	313.2234	341.2183
GILGL	426.3075	454.3024	GILGLG	483.3289	511.3239	GILGLGY	646.3923	674.3872
IL	199.1805	227.1754	ILG	256.2020	284.1969	ILGL	369.2860	397.2809
ILGLG	426.3075	454.3024	ILGLGY	589.3708	617.3657	LG	143.1179	171.1128
LGL	256.2020	284.1969	LGLG	313.2234	341.2183	LGLGY	476.2867	504.2817
LGLGYD	591.3137	619.3086	GL	143.1179	171.1128	GLG	200.1394	228.1343
GLGY	363.2027	391.1976	GLGYD	478.2296	506.2245	LG	143.1179	171.1128
LGY	306.1812	334.1761	LGYD	421.2082	449.2031	GY	193.0972	221.0921
GYD	308.1241	336.1190	YD	251.1026	279.0975			

Annotated ion spectra of the matched peptides with p≤0.05:

CID No.: **23-1323.67**

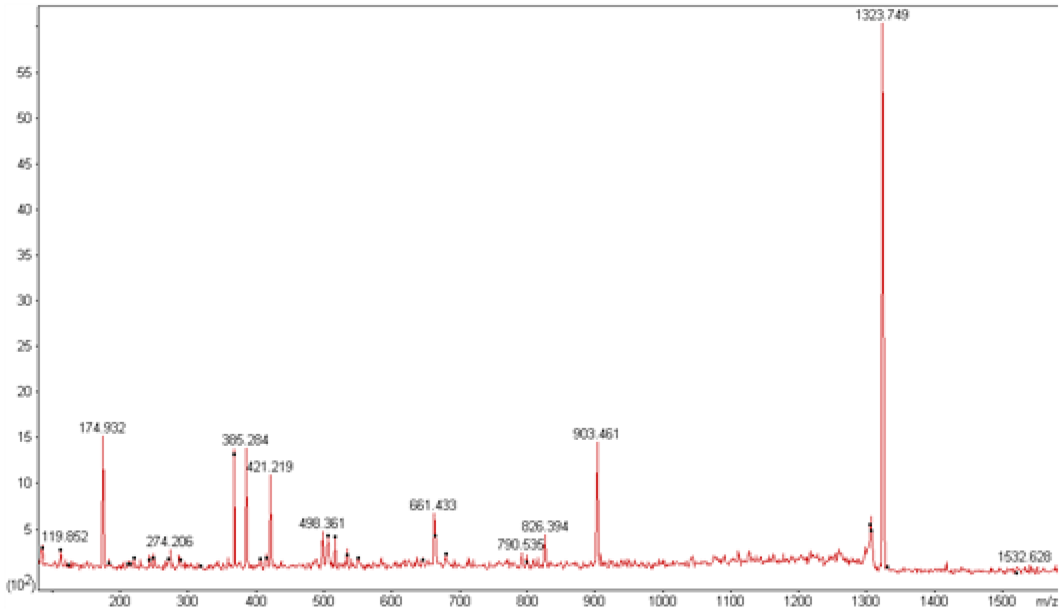

MS/MS Fragmentation of **K.FTGDIEYIPLR.R**

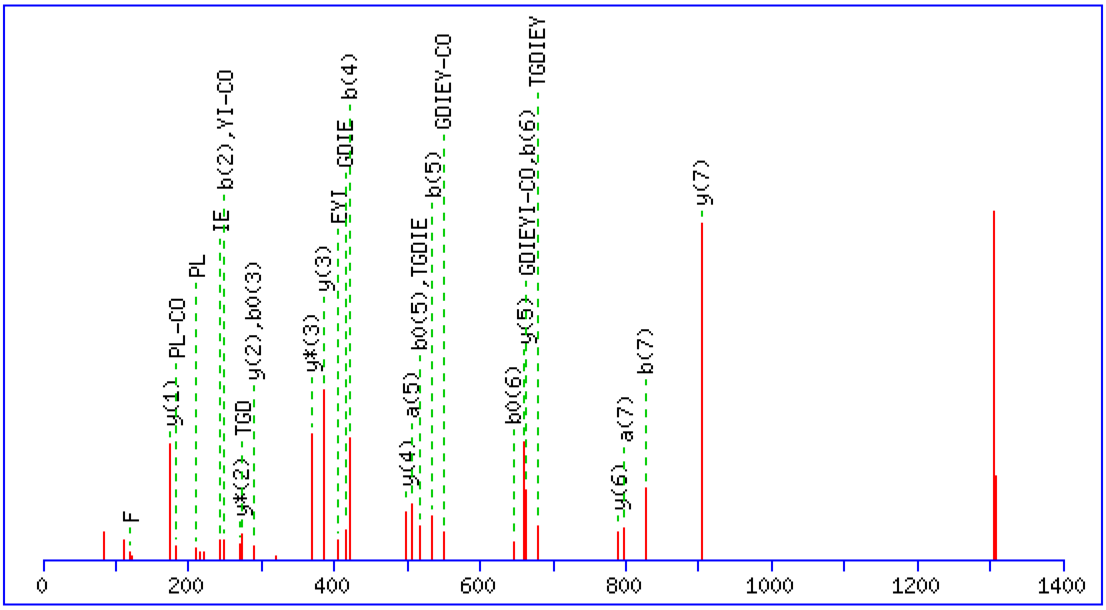

Monoisotopic mass of neutral peptide Mr(calc): 1322.6870

#	Immon.	a	a^0	b	b^0	d	d'	Seq.	v	w	w'	y	y*	y^0	#
1	120.0808	**120.0808**		148.0757		44.0495		F							11
2	74.0600	221.1285	203.1179	**249.1234**	231.1128	205.1335	207.1128	T	1130.5841	1143.6045	1145.5837	1176.6259	1159.5994	1158.6154	10
3	30.0338	278.1499	260.1394	306.1448	288.1343			G				1075.5782	1058.5517	1057.5677	9
4	88.0393	393.1769	375.1663	**421.1718**	403.1612	349.1870		D	958.5356	957.5404		1018.5568	1001.5302	1000.5462	8
5	86.0964	**506.2609**	488.2504	**534.2558**	516.2453	478.2296	492.2453	I	845.4516	858.4720	872.4876	***903.5298***	886.5033	885.5193	7
6	102.0550	635.3035	617.2930	**663.2984**	645.2879	577.2980		E	716.4090	715.4137		***790.4458***	773.4192	772.4352	6
7	136.0757	**798.3668**	780.3563	**826.3618**	808.3512			Y	553.3457			***661.4032***	644.3766		5
8	86.0964	911.4509	893.4403	939.4458	921.4353	883.4196	897.4353	I	440.2616	453.2820	467.2976	***498.3398***	481.3133		4
9	70.0651	1008.5037	990.4931	1036.4986	1018.4880	982.4880		P	343.2088	342.2136		***385.2558***	368.2292		3
10	86.0964	1121.5877	1103.5772	1149.5827	1131.5721	1079.5408		L	230.1248	229.1295		***288.2030***	271.1765		2
11	129.1135							R	74.0237	73.0284		***175.1190***	158.0924		1

Seq	ya	yb	Seq	ya	yb	Seq	ya	yb
TG	131.0815	159.0764	TGD	246.1084	274.1034	TGDI	359.1925	387.1874
TGDIE	488.2351	516.2300	TGDIEY	651.2984	679.2933	GD	145.0608	173.0557
GDI	258.1448	286.1397	GDIE	387.1874	415.1823	GDIEY	550.2508	578.2457
GDIEYI	663.3348	691.3297	DI	201.1234	229.1183	DIE	330.1660	358.1609
DIEY	493.2293	521.2242	DIEYI	606.3134	634.3083	IE	215.1390	243.1339
IEY	378.2023	406.1973	IEYI	491.2864	519.2813	IEYIP	588.3392	616.3341
EY	265.1183	293.1132	EYI	378.2023	406.1973	EYIP	475.2551	503.2500
EYIPL	588.3392	616.3341	YI	249.1598	277.1547	YIP	346.2125	374.2074
YIPL	459.2966	487.2915	IP	183.1492	211.1441	IPL	296.2333	324.2282
PL	183.1492	211.1441						

Annotated ion spectra of the matched peptides with p≤0.05:

CID No.: **23-2070.02**

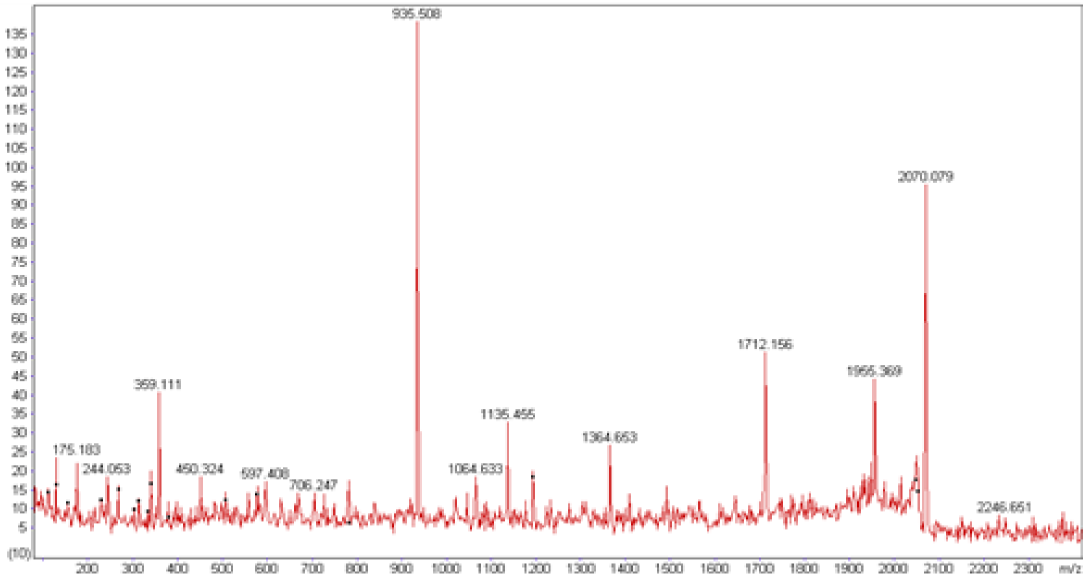

MS/MS Fragmentation of **K.DQDFAEATKEPGLAFAFGR.F**

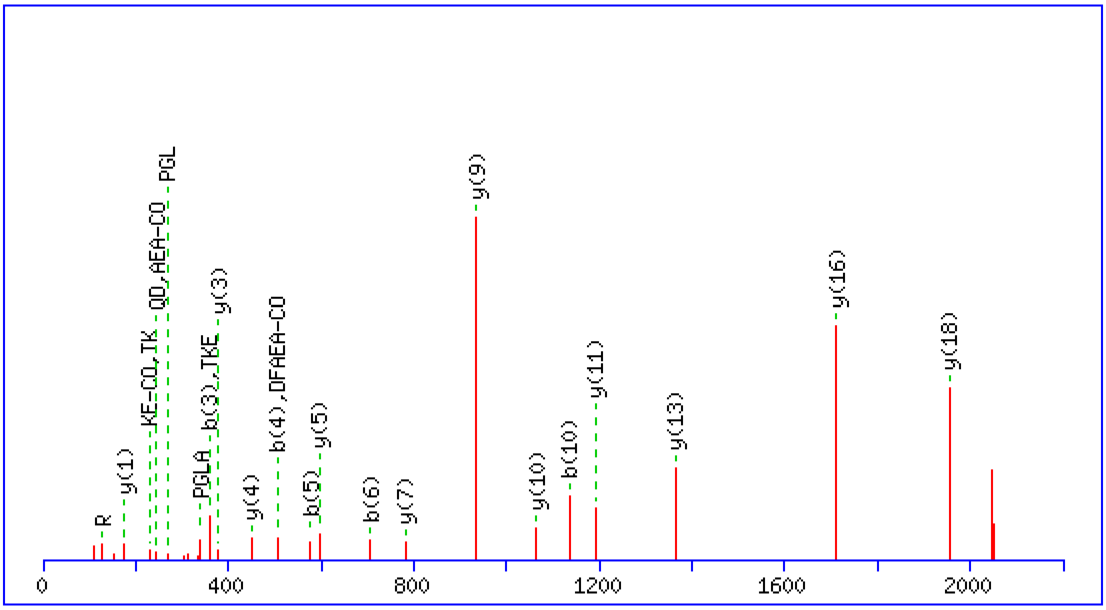

Monoisotopic mass of neutral peptide Mr(calc): 2068.9854

#	Immon.	a	a*	a^0	b	b*	b^0	d	d'	Seq.	v	w	w'	y	y*	y^0	#
1	88.0393	88.0393		70.0287	116.0342		98.0237	44.0495		D							19
2	101.0709	216.0979	199.0713	198.0873	*244.0928*	227.0662	226.0822	159.0764		Q	1881.9130	1880.9177		*1954.9658*	1937.9392	1936.9552	18
3	88.0393	331.1248	314.0983	313.1143	*359.1197*	342.0932	341.1092	287.1350		D	1766.8860	1765.8908		1826.9072	1809.8806	1808.8966	17
4	120.0808	478.1932	461.1667	460.1827	*506.1882*	489.1616	488.1776			F	1619.8176			*1711.8802*	1694.8537	1693.8697	16
5	44.0495	549.2304	532.2038	531.2198	*577.2253*	560.1987	559.2147			A	1548.7805			1564.8118	1547.7853	1546.8013	15
6	102.0550	678.2729	661.2464	660.2624	*706.2679*	689.2413	688.2573	620.2675		E	1419.7379	1418.7427		1493.7747	1476.7482	1475.7641	14
7	44.0495	749.3101	732.2835	731.2995	777.3050	760.2784	759.2944			A	1348.7008			*1364.7321*	1347.7056	1346.7215	13
8	74.0600	850.3577	833.3312	832.3472	878.3527	861.3261	860.3421	834.3628	836.3421	T	1247.6531	1260.6735	1262.6528	1293.6950	1276.6685	1275.6844	12
9	101.1073	978.4527	961.4262	960.4421	1006.4476	989.4211	988.4371	921.3949		K	1119.5582	1118.5629		*1192.6473*	1175.6208	1174.6368	11
10	102.0550	1107.4953	1090.4687	1089.4847	*1135.4902*	1118.4637	1117.4796	1049.4898		E	990.5156	989.5203		*1064.5524*	1047.5258	1046.5418	10
11	70.0651	1204.5481	1187.5215	1186.5375	1232.5430	1215.5164	1214.5324	1178.5324		P	893.4628	892.4676		*935.5098*	918.4832		9
12	30.0338	1261.5695	1244.5430	1243.5590	1289.5644	1272.5379	1271.5539			G				838.4570	821.4305		8
13	86.0964	1374.6536	1357.6270	1356.6430	1402.6485	1385.6220	1384.6379	1332.6066		L	723.3573	722.3620		*781.4355*	764.4090		7
14	44.0495	1445.6907	1428.6642	1427.6801	1473.6856	1456.6591	1455.6751			A	652.3202			668.3515	651.3249		6
15	120.0808	1592.7591	1575.7326	1574.7486	1620.7540	1603.7275	1602.7435			F	505.2518			*597.3144*	580.2878		5
16	44.0495	1663.7962	1646.7697	1645.7857	1691.7911	1674.7646	1673.7806			A	434.2146			*450.2459*	433.2194		4
17	120.0808	1810.8646	1793.8381	1792.8541	1838.8596	1821.8330	1820.8490			F	287.1462			*379.2088*	362.1823		3
18	30.0338	1867.8861	1850.8596	1849.8755	1895.8810	1878.8545	1877.8705			G				232.1404	215.1139		2
19	129.1135									R	74.0237	73.0284		*175.1190*	158.0924		1

Seq	ya	yb	Seq	ya	yb	Seq	ya	yb
QD	216.0979	244.0928	QDF	363.1663	391.1612	QDFA	434.2034	462.1983
QDFAE	563.2460	591.2409	QDFAEA	634.2831	662.2780	DF	235.1077	263.1026
DFA	306.1448	334.1397	DFAE	435.1874	463.1823	DFAEA	506.2245	534.2195
DFAEAT	607.2722	635.2671	FA	191.1179	219.1128	FAE	320.1605	348.1554
FAEA	391.1976	419.1925	FAEAT	492.2453	520.2402	FAEATK	620.3402	648.3352
AE	173.0921	201.0870	AEA	244.1292	272.1241	AEAT	345.1769	373.1718
AEATK	473.2718	501.2667	AEATKE	602.3144	630.3093	AEATKEP	699.3672	727.3621
EA	173.0921	201.0870	EAT	274.1397	302.1347	EATK	402.2347	430.2296
EATKE	531.2773	559.2722	EATKEP	628.3301	656.3250	EATKEPG	685.3515	713.3464
AT	145.0972	173.0921	ATK	273.1921	301.1870	ATKE	402.2347	430.2296
ATKEP	499.2875	527.2824	ATKEPG	556.3089	584.3039	ATKEPGL	669.3930	697.3879
TK	202.1550	230.1499	TKE	331.1976	359.1925	TKEP	428.2504	456.2453
TKEPG	485.2718	513.2667	TKEPGL	598.3559	626.3508	TKEPGLA	669.3930	697.3879
KE	230.1499	258.1448	KEP	327.2027	355.1976	KEPG	384.2241	412.2191
KEPGL	497.3082	525.3031	KEPGLA	568.3453	596.3402	EP	199.1077	227.1026
EPG	256.1292	284.1241	EPGL	369.2132	397.2082	EPGLA	440.2504	468.2453
EPGLAF	587.3188	615.3137	EPGLAFA	658.3559	686.3508	PG	127.0866	155.0815
PGL	240.1707	268.1656	PGLA	311.2078	339.2027	PGLAF	458.2762	486.2711
PGLAFA	529.3133	557.3082	PGLAFAF	676.3817	704.3766	GL	143.1179	171.1128
GLA	214.1550	242.1499	GLAF	361.2234	389.2183	GLAFA	432.2605	460.2554
GLAFAF	579.3289	607.3239	GLAFAFG	636.3504	664.3453	LA	157.1335	185.1285
LAF	304.2020	332.1969	LAFA	375.2391	403.2340	LAFAF	522.3075	550.3024
LAFAFG	579.3289	607.3239	AF	191.1179	219.1128	AFA	262.1550	290.1499
AFAF	409.2234	437.2183	AFAFG	466.2449	494.2398	FA	191.1179	219.1128
FAF	338.1863	366.1812	FAFG	395.2078	423.2027	AF	191.1179	219.1128
AFG	248.1394	276.1343	FG	177.1022	205.0972			

Spot No.: **24**

Mascot score: **95**

Species: ***Fusarium oxysporum Fo5176***

Protein name: **vacuolar protease A**

NCBI accession No.: **gi| 342882947** Sequence coverage %: **21**

Matched peptides No.: **9** Total peptides No.: **39**

Calculated Mr: **42978** Calculated *p*I: **4.66**

Annotated PMF **spectra:**

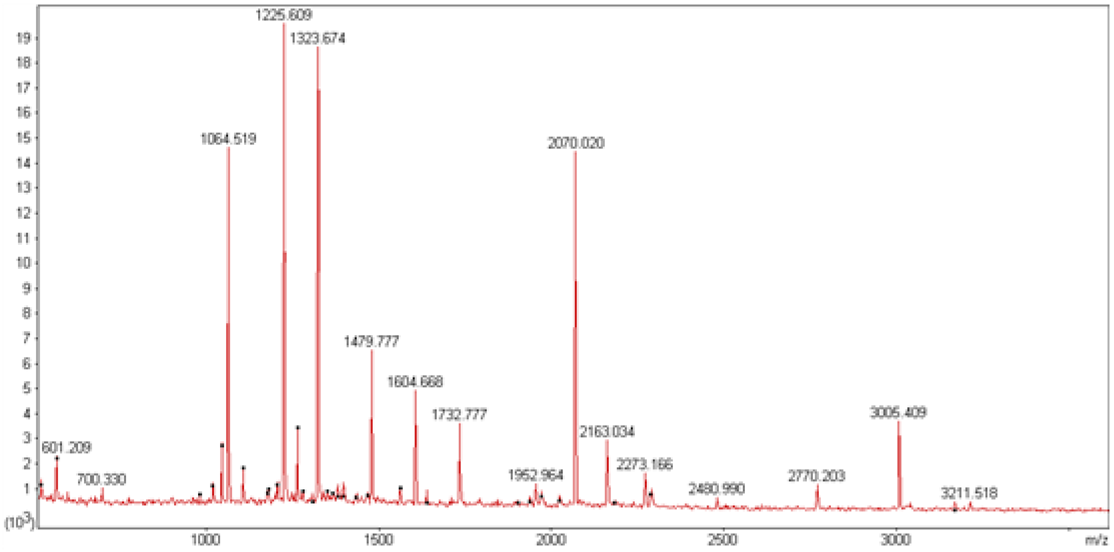

Probability Based Mowse Score:

Protein score is -10*Log(P), where P is the probability that the observed match is a random event.
Protein scores greater than 75 are significant (p<0.05).

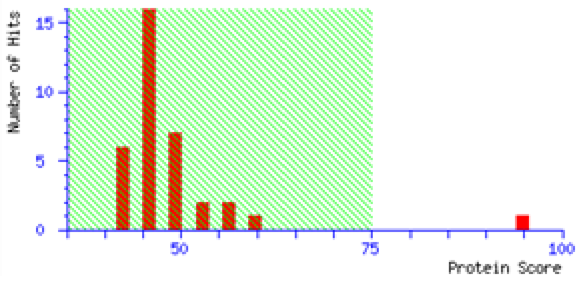

Matched peptide sequences: shown in **Bold Red**

```
  1 MKGALLTAAA LLGSAQAGVH KMKLNKVPLA DQLATNSVED HLQSLGQKYL
 51 GASRPKNAAD YAFATNTVNV EGGHPVPVSN FMNAQYFSEI TIGTPPQSFK
101 VVLDTGSSNL WVPSQQCGSI ACYLHSKYDS SASSTYKENG TEFEIHYGSG
151 SLSGFVSNDV VSIGDLEIKD QDFAEATKEP GLAFAFGRFD GILGLGYDRI
201 AVNGMVPPFY QMVNQKLLDE PVFAFYLDDQ EGESEATFGG IDKSKFTGDI
251 EYIPLRRKAY WEVDLEAIAF GDEVAEQENT GAILDTGTSL NVLPSALAEL
301 LNKEIGAKKG YNGQYTIECD KRASLPDITF NLAGSNYSLP ATDYILEVQG
351 SCISTFQGMD FPEPVGPLVI LGDAFLRRYY SVYDLGKNAV GLARAK
```

Matched peptides information:

Start	-	End	Observed	Mr(expt)	Mr(calc)	ppm	M	Peptide
170	-	188	2070.0204	2069.0131	2068.9854	13.4	1	K.DQDFAEATKEPGLAFAFGR.F
179	-	188	1064.5189	1063.5116	1063.5451	-31.5	0	K.EPGLAFAFGR.F
189	-	199	1225.6092	1224.6019	1224.6139	-9.77	0	R.FDGILGLGYDR.I
217	-	243	3005.4088	3004.4015	3004.3866	4.97	0	K.LLDEPVFAFYLDDQEGESEATFGGIDK.S
246	-	256	1323.6737	1322.6664	1322.6870	-15.6	0	K.FTGDIEYIPLR.R
246	-	257	1479.7771	1478.7698	1478.7881	-12.4	1	K.FTGDIEYIPLRR.K
378	-	387	1263.6303	1262.6230	1262.6295	-5.14	1	R.RYYSVYDLGK.N
379	-	387	1107.4779	1106.4706	1106.5284	-52.2	0	R.YYSVYDLGK.N
388	-	394	700.3298	699.3225	699.4028	-115	0	K.NAVGLAR.A

Spot No.: **25**

Mascot score: **137**

Species: ***Fusarium oxysporum Fo5176***

Protein name: **NADP-dependent glycerol dehydrogenase**

NCBI accession No.: **gi| 342888721** Sequence coverage %: **59**

Matched peptides No.: **20** Total peptides No.: **75**

Calculated Mr: **37413** Calculated *p*I: **5.91**

Annotated PMF **spectra:**

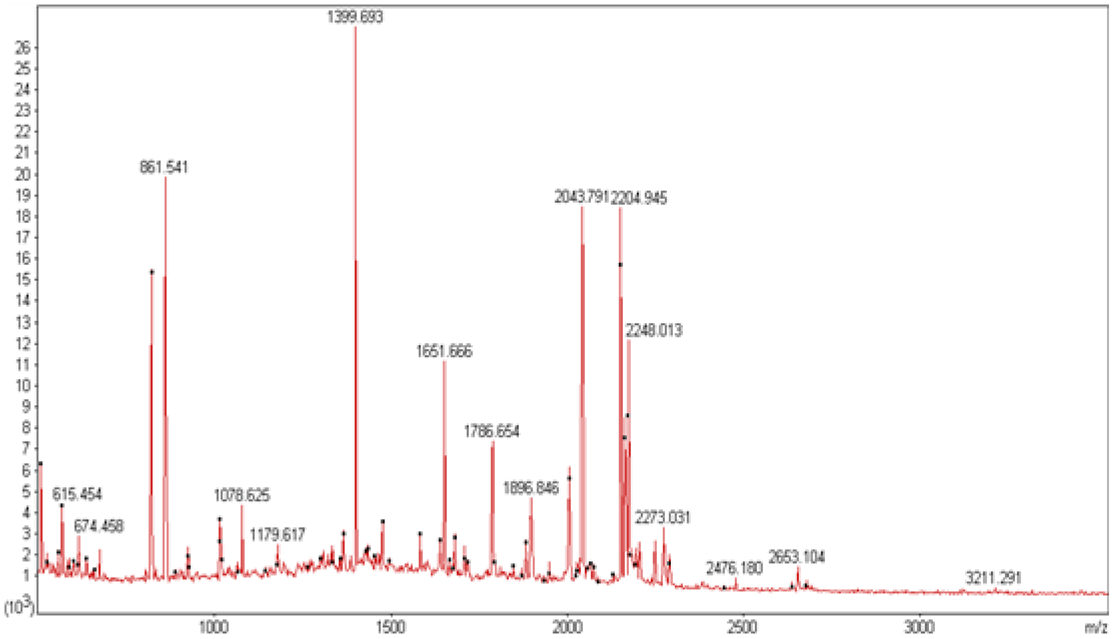

Probability Based Mowse Score:

Protein score is -10*Log(P), where P is the probability that the observed match is a random event.
Protein scores greater than 75 are significant (p<0.05).

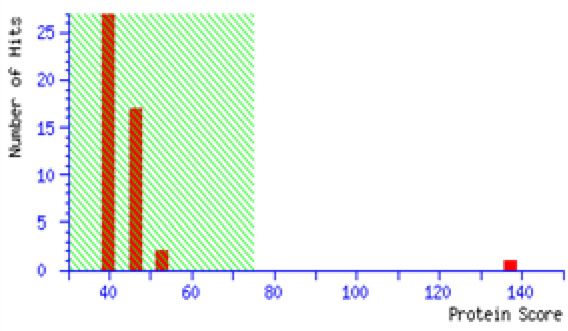

Matched peptide sequences: shown in **Bold Red**

```
  1  MSFGRTVTLN SGHKMPQIGY GTWQAAPGEV GNGVYEALKA GYRHLDLAKI
 51  YQNQREVGEG IKKALNDVPG LKREDIFITG KLWNNKHRPE EVPGALDDSL
101  EELGLDYLDL WLIHWPVAFK NGPELFPLKA DDKNKTELDQ GVTLSQTWEA
151  VTKLPKEKVR SIGVSNFSIE MLETIIKDTG VTPAINQVER HPRLPQPELV
201  KYQKEKGIYL TAYSAFGNNS WGEPLLINTP EVKAIAERLS KSKGKEVTPA
251  QVILAWSTLD NHIVIPKSVT PARIRSNFEE VELDEEAIKE LEKFGEKPQR
301  FNIPKTYSPD WDIDVFGDEK EKTATHKVVL KL
```

Residues in bold red: 1–5 (MSFGR), 50–55 (IYQNQR), 64–72 (ALNDVPGLK), 73–81 (REDIFITGK), 82–86 (LWNNK), 121–129 (NGPELFPLK), 134–160 (NKTELDQ GVTLSQTWEA VTK), 161–201 (SIGVSNFSIE MLETIIKDTG VTPAINQVER … LPQPELV K), 205–238 (EKGIYL TAYSAFGNNS WGEPLLINTP EVKAIAER), 246–267 (EVTPA QVILAWSTLD NHIVIPK), 276–300 (SNFEE VELDEEAIKE LEKFGEKPQR), 306–322 (TYSPD WDIDVFGDEK EK).

Matched peptides information:

Start	-	End	Observed	Mr(expt)	Mr(calc)	ppm	M	Peptide
1	-	5	613.4533	612.4460	612.2690	289	0	-.MSFGR.T + Oxidation (M)
50	-	55	821.5249	820.5177	820.4191	120	0	K.IYQNQR.E
64	-	72	926.5623	925.5551	925.5233	34.3	0	K.ALNDVPGLK.R
73	-	81	1078.6246	1077.6173	1077.5818	32.9	1	K.REDIFITGK.L
82	-	86	674.4577	673.4504	673.3547	142	0	K.LWNNK.H
121	-	129	1014.5672	1013.5599	1013.5546	5.29	0	K.NGPELFPLK.A
134	-	153	2248.0129	2247.0056	2247.1383	-59.0	1	K.NKTELDQGVTLSQTWEAVTK.L
136	-	153	2005.8525	2004.8452	2005.0004	-77.4	0	K.TELDQGVTLSQTWEAVTK.L
161	-	177	1880.8568	1879.8495	1879.9965	-78.2	0	R.SIGVSNFSIEMLETIIK.D
161	-	177	1896.8464	1895.8391	1895.9914	-80.3	0	R.SIGVSNFSIEMLETIIK.D + Oxidation (M)
178	-	190	1399.6925	1398.6852	1398.7103	-17.9	0	K.DTGVTPAINQVER.H
194	-	201	923.5861	922.5789	922.5488	32.7	0	R.LPQPELVK.Y
205	-	233	3211.2914	3210.2841	3210.6237	-106	1	K.EKGIYLTAYSAFGNNSWGEPLLINTPEVK.A
234	-	238	559.4548	558.4476	558.3125	242	0	K.AIAER.L
246	-	267	2444.2407	2443.2334	2443.3475	-46.7	0	K.EVTPAQVILAWSTLDNHIVIPK.S
276	-	289	1651.6655	1650.6582	1650.7624	-63.1	0	R.SNFEEVELDEEAIK.E
276	-	293	2150.9036	2149.8963	2150.0266	-60.6	1	R.SNFEEVELDEEAIKELEK.F
294	-	300	861.5414	860.5341	860.4504	97.3	0	K.FGEKPQR.F
306	-	320	1786.6544	1785.6471	1785.7734	-70.7	0	K.TYSPDWDIDVFGDEK.E
306	-	322	2043.7910	2042.7837	2042.9109	-62.3	1	K.TYSPDWDIDVFGDEKEK.T

Spot No.: **26**

NCBI accession No.: **gi|46128717**

Plant species: ***Fusarium graminearum PH-1***

Protein name： **peptidase C1-like family protein**

Mascot score: **92** Sequence coverage %: **2**

The number of matched peptides with p≤0.05: **2**

Calculated Mr: **55346** Calculated *P*i: **5.53**

Annotated MS spectra:

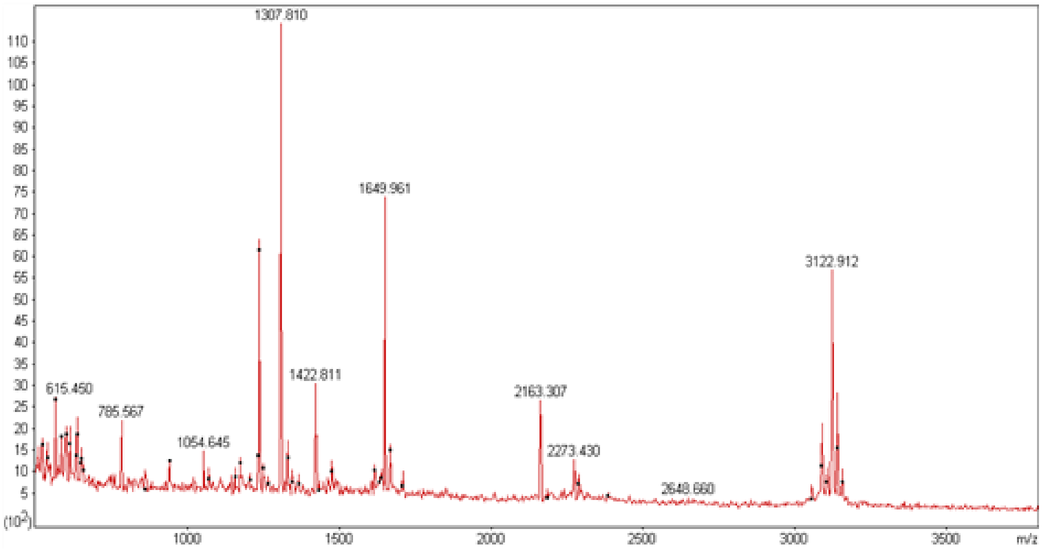

Probability Based Mowse Score:

Ions score is -10*Log(P), where P is the probability that the observed match is a random event.
Individual ions scores > 48 indicate identity or extensive homology (p<0.05).
Protein scores are derived from ions scores as a non-probabilistic basis for ranking protein hits.

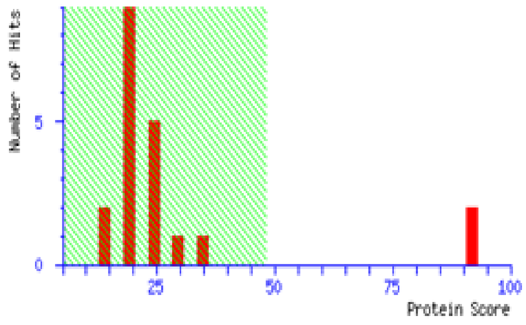

Matched peptide sequences: shown in Bold Red

```
  1  MGAQSSKPTL  HEKAVLDRLH  SLQLQDDDEY  VEISSDSEKA  PLGPLVRDAQ
 51  GLSVHVLESW  QASILKDPKN  KQVLTSLPTQ  IADQQVFNVK  IPFEGDPITN
101  QRSSGRCWLF  ASTNVFRVAL  MKRYNLESFE  LSQQYLFYWD  KLEKSNWFLE
151  QVIDTAGEDL  EGRLVQNLLG  DIVSDGGQWD  MVYNLVEKYG  LVPQTLYPDT
201  WNAQSSGILN  NVIKTKLREF  ALKLRGLINS  QNGVSATALS  SAKDKMMREI
251  SLIMTLLLGP  PPSPEDAFTW  QYNDKNGKAH  EVKATPREFA  KNIYSSEFRI
301  TSTTIDSMIS  LVHDPRHEPL  NLLSVSRLGN  IVGGRGVSYV  NVDIDTLKKT
351  CIKMLKAGIP  IFFGSDVGKF  SDSTSGIMDL  DLFNYELGFN  TSLLGMSKAQ
401  RLTTKESQMT  HAMVLTAVHL  DEETGKPVRW  RVQNSWGTTA  GDKGWFVMSD
451  AWLDEFVYQA  VVDPRFCSKE  VRDVLKKEAI  ILPPWDPMGA  LA
```

Matched peptides information:

Query	Start - End	Observed	Mr(expt)	Mr(calc)	ppm	M	Score	Expect	Rank	U	Peptide
	357 - 369	1307.8101	1306.8028	1306.6922	84.7	0	92	2.7e-06	1	U	K.AGIPIFFGSDVGK.F
	357 - 369	1307.8101	1306.8028	1306.6922	84.7	0	92	2.7e-06	1	U	K.AGIPIFFGSDVGK.F

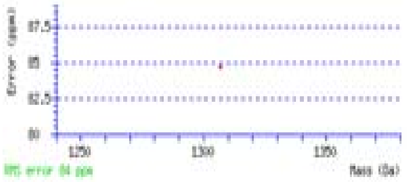

Annotated ion spectra of the matched peptides with p≤0.05:

CID No.: **26-1307.8**

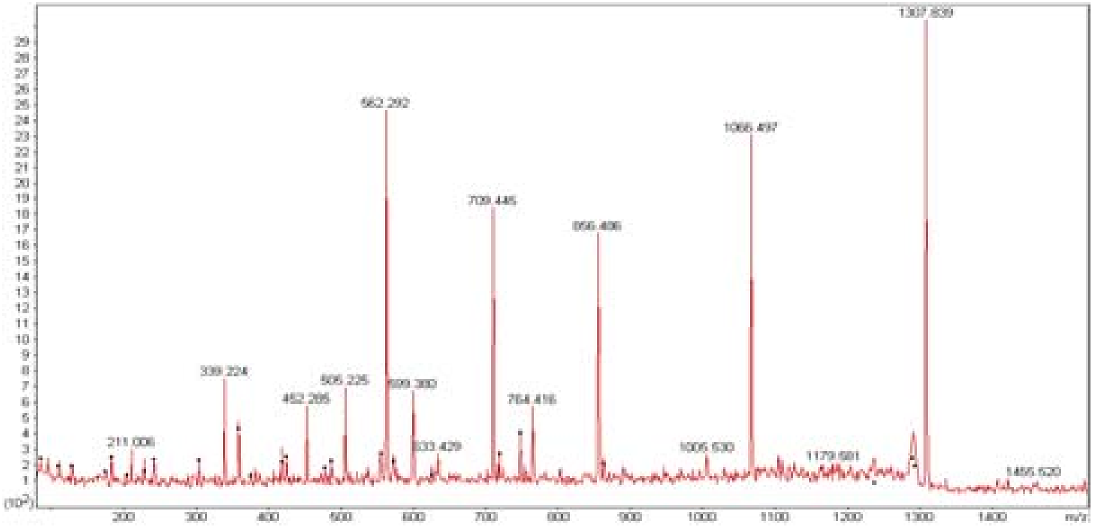

MS/MS Fragmentation of **K.AGIPIFFGSDVGK.F**

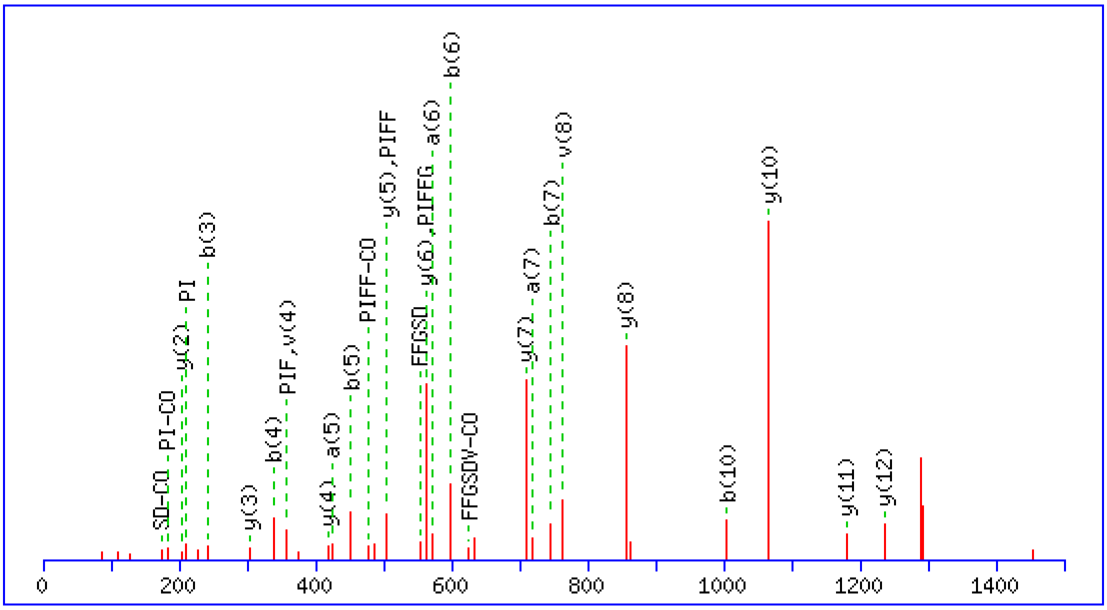

Monoisotopic mass of neutral peptide Mr(calc): 1306.6922

#	Immon.	a	a^0	b	b^0	d	d'	Seq.	v	w	w'	y	y*	y^0	#
1	44.0495	44.0495		72.0444		44.0495		A							13
2	30.0338	101.0709		129.0659				G				1236.6623	1219.6358	1218.6517	12
3	86.0964	214.1550		242.1499		186.1237	200.1394	I	1121.5626	1134.5830	1148.5986	1179.6408	1162.6143	1161.6303	11
4	70.0651	311.2078		339.2027		285.1921		P	1024.5098	1023.5146		1066.5568	1049.5302	1048.5462	10
5	86.0964	424.2918		452.2867		396.2605	410.2762	I	911.4258	924.4462	938.4618	969.5040	952.4775	951.4934	9
6	120.0808	571.3602		599.3552				F	764.3573			856.4199	839.3934	838.4094	8
7	120.0808	718.4287		746.4236				F	617.2889			709.3515	692.3250	691.3410	7
8	30.0338	775.4501		803.4450				G				562.2831	545.2566	544.2726	6
9	60.0444	862.4822	844.4716	890.4771	872.4665	846.4872		S	473.2354	472.2402		505.2617	488.2351	487.2511	5
10	88.0393	977.5091	959.4985	1005.5040	987.4934	933.5193		D	358.2085	357.2132		418.2296	401.2031	400.2191	4
11	72.0808	1076.5775	1058.5669	1104.5724	1086.5619	1062.5619		V	259.1401	272.1605		303.2027	286.1761		3
12	30.0338	1133.5990	1115.5884	1161.5939	1143.5833			G				204.1343	187.1077		2
13	101.1073							K	74.0237	73.0284		147.1128	130.0863		1

Seq	ya	yb	Seq	ya	yb	Seq	ya	yb
GI	143.1179	171.1128	GIP	240.1707	268.1656	GIPI	353.2547	381.2496
GIPIF	500.3231	528.3180	GIPIFF	647.3915	675.3865	IP	183.1492	211.1441
IPI	296.2333	324.2282	IPIF	443.3017	471.2966	IPIFF	590.3701	618.3650
IPIFFG	647.3915	675.3865	PI	183.1492	211.1441	PIF	330.2176	358.2125
PIFF	477.2860	505.2809	PIFFG	534.3075	562.3024	PIFFGS	621.3395	649.3344
IF	233.1648	261.1598	IFF	380.2333	408.2282	IFFG	437.2547	465.2496
IFFGS	524.2867	552.2817	IFFGSD	639.3137	667.3086	FF	267.1492	295.1441
FFG	324.1707	352.1656	FFGS	411.2027	439.1976	FFGSD	526.2296	554.2245
FFGSDV	625.2980	653.2930	FFGSDVG	682.3195	710.3144	FG	177.1022	205.0972
FGS	264.1343	292.1292	FGSD	379.1612	407.1561	FGSDV	478.2296	506.2245
FGSDVG	535.2511	563.2460	GS	117.0659	145.0608	GSD	232.0928	260.0877
GSDV	331.1612	359.1561	GSDVG	388.1827	416.1776	SD	175.0713	203.0662
SDV	274.1397	302.1347	SDVG	331.1612	359.1561	DV	187.1077	215.1026
DVG	244.1292	272.1241	VG	129.1022	157.0972			

Spot No.: **27**

Mascot score: **208**

Species: ***Fusarium oxysporum Fo5176***

Protein name: **NADP-dependent glycerol dehydrogenase**

NCBI accession No.: **gi| 342888721**

Sequence coverage %: **61**

Matched peptides No.: **23**

Total peptides No.: **47**

Calculated Mr: **37413**

Calculated *p*I: **5.91**

Annotated PMF **spectra:**

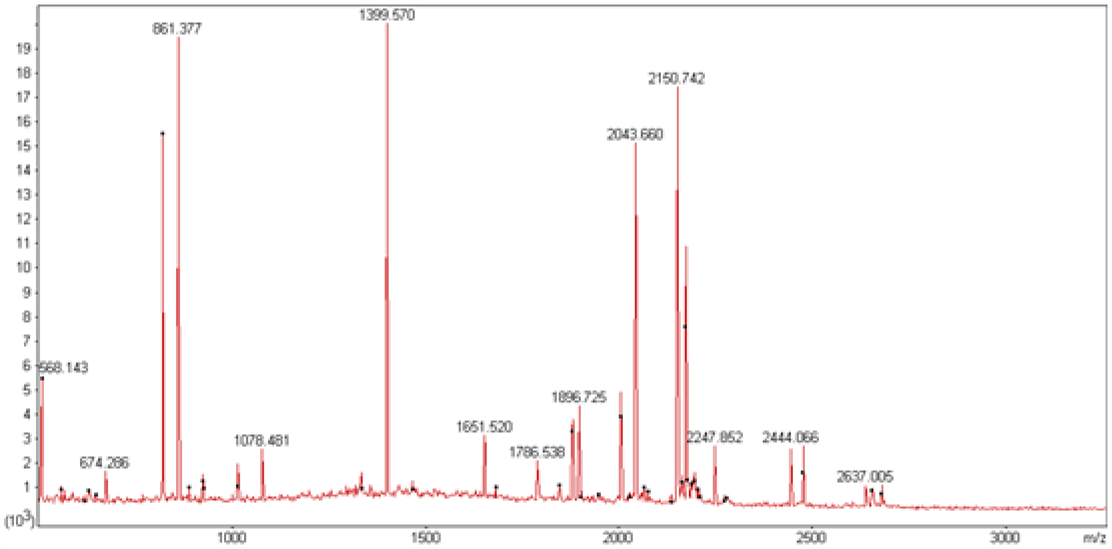

Probability Based Mowse Score:

Protein score is -10*Log(P), where P is the probability that the observed match is a random event.
Protein scores greater than 75 are significant (p<0.05).

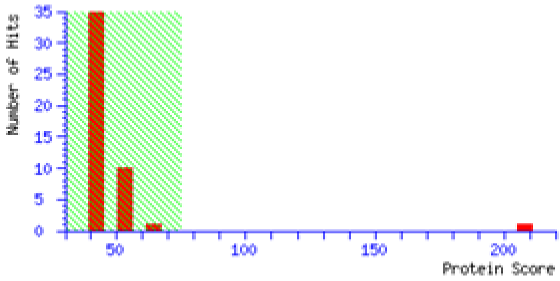

Matched peptide sequences: shown in **Bold Red**

```
  1 MSFGRTVTLN SGHKMPQIGY GTWQAAPGEV GNGVYEALKA GYRHLDLAKI
 51 YQNQREVGEG IKKALNDVPG LKREDIFITG KLWNNKHRPE EVPGALDDSL
101 EELGLDYLDL WLIHWPVAFK NGPELFPLKA DDKNKTELDQ GVTLSQTWEA
151 VTKLPKEKVR SIGVSNFSIE MLETIIKDTG VTPAINQVER HPRLPQPELV
201 KYQKEKGIYL TAYSAFGNNS WGEPLLINTP EVKAIAERLS KSKGKEVTPA
251 QVILAWSTLD NHIVIPKSVT PARIRSNFEE VELDEEAIKE LEKFGEKPQR
301 FNIPKTYSPD WDIDVFGDEK EKTATHKVVL KL
```

Matched peptides information:

Start – End	Observed	Mr(expt)	Mr(calc)	ppm	M	Peptide
15 – 39	2637.0051	2635.9978	2635.2741	275	0	K.MPQIGYGTWQAAPGEVGNGVYEALK.A
15 – 39	2652.9932	2651.9859	2651.2690	270	0	K.MPQIGYGTWQAAPGEVGNGVYEALK.A + Oxidation (M)
50 – 55	821.3569	820.3496	820.4191	-84.7	0	K.IYQNQR.E
64 – 72	926.4301	925.4228	925.5233	-109	0	K.ALNDVPGLK.R
73 – 81	1078.4807	1077.4734	1077.5818	-101	1	K.REDIFITGK.L
82 – 86	674.2861	673.2788	673.3547	-113	0	K.LWNNK.H
121 – 129	1014.4284	1013.4211	1013.5546	-132	0	K.NGPELFPLK.A
134 – 153	2247.8521	2246.8448	2247.1383	-131	1	K.NKTELDQGVTLSQTWEAVTK.L
136 – 153	2005.7168	2004.7095	2005.0004	-145	0	K.TELDQGVTLSQTWEAVTK.L
159 – 177	2135.7486	2134.7413	2135.1660	-199	1	K.VRSIGVSNFSIEMLETIIK.D
161 – 177	1880.7529	1879.7456	1879.9965	-133	0	R.SIGVSNFSIEMLETIIK.D
161 – 177	1896.7250	1895.7177	1895.9914	-144	0	R.SIGVSNFSIEMLETIIK.D + Oxidation (M)
178 – 190	1399.5701	1398.5628	1398.7103	-105	0	K.DTGVTPAINQVER.H
194 – 201	923.4364	922.4292	922.5488	-130	0	R.LPQPELVK.Y
234 – 238	559.2924	558.2851	558.3125	-49.1	0	K.AIAER.L
246 – 267	2444.0661	2443.0588	2443.3475	-118	0	K.EVTPAQVILAWSTLDNHIVIPK.S
268 – 273	630.3376	629.3303	629.3497	-30.8	0	K.SVTPAR.I
276 – 289	1651.5197	1650.5124	1650.7624	-151	0	R.SNFEEVELDEEAIK.E
276 – 293	2150.7421	2149.7348	2150.0266	-136	1	R.SNFEEVELDEEAIKELEK.F
294 – 300	861.3772	860.3699	860.4504	-93.6	0	K.FGEKPQR.F
301 – 305	618.2957	617.2884	617.3537	-106	0	R.FNIPK.T
306 – 320	1786.5385	1785.5312	1785.7734	-136	0	K.TYSPDWDIDVFGDEK.E
306 – 322	2043.6600	2042.6527	2042.9109	-126	1	K.TYSPDWDIDVFGDEKEK.T

Spot No.: **28**

Mascot score: **122**

Species: ***Fusarium oxysporum f. sp. cubense race 1***

Protein name: **Superoxide dismutase [Mn], mitochondrial**

NCBI accession No.: **gi| 477512799** Sequence coverage %: **55**

Matched peptides No.: **10** Total peptides No.: **59**

Calculated Mr: **21308** Calculated *p*I: **7.07**

Annotated PMF **spectra:**

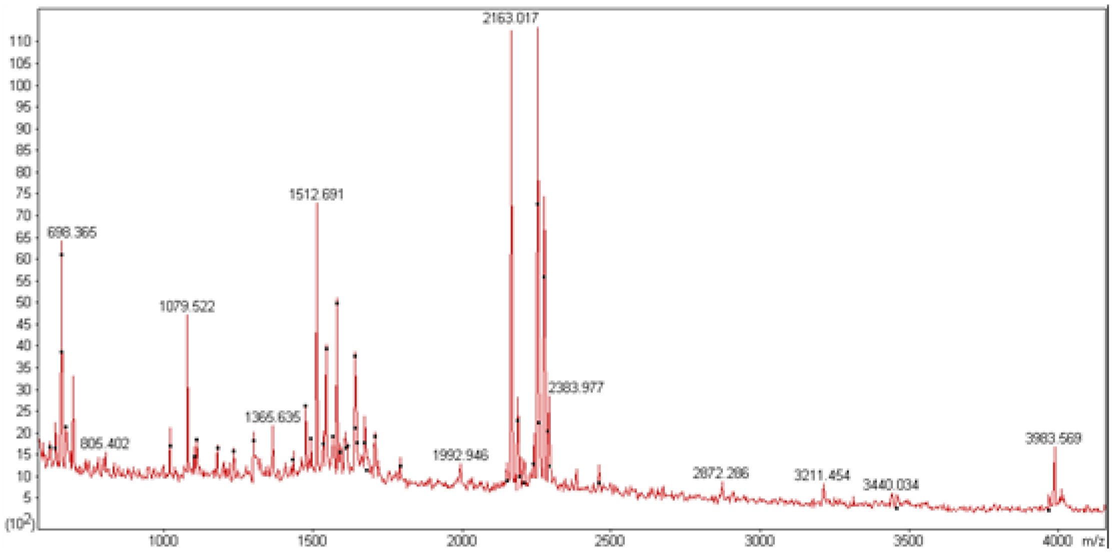

Probability Based Mowse Score:

Protein score is -10*Log(P), where P is the probability that the observed match is a random event.
Protein scores greater than 75 are significant (p<0.05).

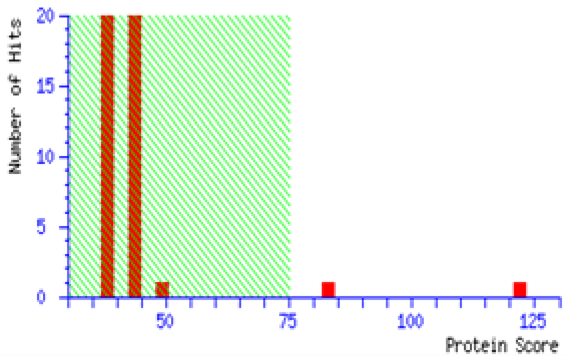

Matched peptide sequences: shown in Bold Red

```
  1  MSVGTYSLPA LPYAYDALEP SISAQIMELH HSKHHQAYVT NLNAALKNYA
 51  TATSTNDIAG QIALQSAIKF NGGGHINHSL FWENLSPSSS ADAKPESAPT
101  LSAEISKTWG SIQAFQEAFK KTLLGLQGSG WGWLVKDTHG LRIVTTKDQD
151  PVVGGEYLNG KAAYVDNIWK VINWKTAEAR FTGTREDAFK VLRASI
```

Red (matched) portions: HHQAYVT NLNAALKNYA (34–50); TATSTNDIAG QIALQSAIKF NGGGHINHSL FWENLSPSSS ADAKPESAPT (51–100); LSAEISKTWG SIQAFQEAFK KTLLGLQGSG WGWLVKDTHG LR (101–142); KAAYVDNIWK VINWK (162–175); FTGTR (181–185).

Matched peptides information:

Start	-	End	Observed	Mr(expt)	Mr(calc)	ppm	M	Peptide
34	-	47	1579.7875	1578.7802	1578.8266	-29.4	0	K.HHQAYVTNLNAALK.N
48	-	69	2251.1317	2250.1244	2250.1492	-11.0	0	K.NYATATSTNDIAGQIALQSAIK.F
70	-	107	3983.5690	3982.5617	3981.9133	163	0	K.FNGGGHINHSLFWENLSPSSSADAKPESAPTLSAEISK.T
108	-	120	1512.6914	1511.6841	1511.7409	-37.5	0	K.TWGSIQAFQEAFK.K
108	-	121	1640.7960	1639.7887	1639.8358	-28.7	1	K.TWGSIQAFQEAFKK.T
122	-	136	1614.8054	1613.7981	1613.8930	-58.8	0	K.TLLGLQGSGWGWLVK.D
137	-	142	698.3650	697.3577	697.3507	10.0	0	K.DTHGLR.I
162	-	170	1079.5220	1078.5147	1078.5447	-27.8	0	K.AAYVDNIWK.V
171	-	175	659.3752	658.3679	658.3802	-18.8	0	K.VINWK.T
181	-	185	581.3272	580.3199	580.2969	39.7	0	R.FTGTR.E

Spot No.: **29**

Mascot score: **84**

Species: ***Fusarium oxysporum Fo5176***

Protein name: **vacuolar protease A**

NCBI accession No.: **gi| 342882947**

Sequence coverage %: **19**

Matched peptides No.: **8**

Total peptides No.: **49**

Calculated Mr: **42978**

Calculated *p*I: **4.66**

Annotated PMF **spectra:**

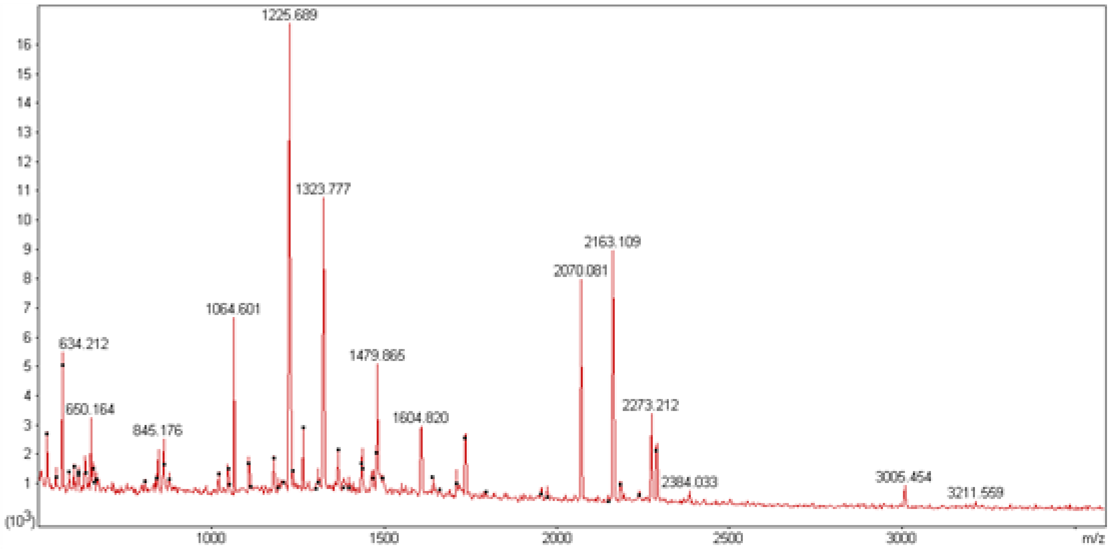

Probability Based Mowse Score:

Protein score is -10*Log(P), where P is the probability that the observed match is a random event.
Protein scores greater than 75 are significant (p<0.05).

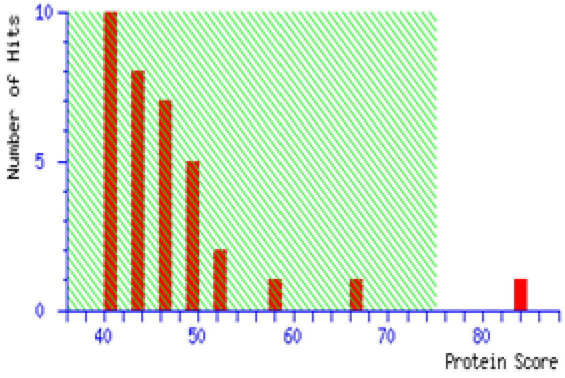

Matched peptide sequences: shown in **Bold Red**

1 MKGALLTAAA LLGSAQAGVH KMKLNKVPLA DQLATNSVED HLQSLGQKYL
51 GASRPKNAAD YAFATNTVNV EGGHPVPVSN FMNAQYFSEI TIGTPPQSFK
101 VVLDTGSSNL WVPSQQCGSI ACYLHSKYDS SASSTYKENG TEFEIHYGSG
151 SLSGFVSNDV VSIGDLEIK**D** **QDFAEATKEP** **GLAFAFGRFD** **GILGLGYDRI**
201 AVNGMVPPFY QMVNQK**LLDE** **PVFAFYLDDQ** **EGESEATFGG** **IDK**SK**FTGDI**
251 **EYIPLRR**KAY WEVDLEAIAF GDEVAEQENT GAILDTGTSL NVLPSALAEL
301 LNKEIGAKKG YNGQYTIECD KRASLPDITF NLAGSNYSLP ATDYILEVQG
351 SCISTFQGMD FPEPVGPLVI LGDAFLR**RYY** **SVYDLGK**NAV GLARAK

Matched peptides information:

Start	-	End	Observed	Mr(expt)	Mr(calc)	ppm	M	Peptide
170	-	188	2070.0807	2069.0734	2068.9854	42.5	1	K.DQDFAEATKEPGLAFAFGR.F
179	-	188	1064.6010	1063.5937	1063.5451	45.7	0	K.EPGLAFAFGR.F
189	-	199	1225.6887	1224.6814	1224.6139	55.1	0	R.FDGILGLGYDR.I
217	-	243	3005.4543	3004.4470	3004.3866	20.1	0	K.LLDEPVFAFYLDDQEGESEATFGGIDK.S
246	-	256	1323.7772	1322.7699	1322.6870	62.7	0	K.FTGDIEYIPLR.R
246	-	257	1479.8653	1478.8580	1478.7881	47.3	1	K.FTGDIEYIPLRR.K
378	-	387	1263.6825	1262.6752	1262.6295	36.2	1	R.RYYSVYDLGK.N
379	-	387	1107.5764	1106.5691	1106.5284	36.8	0	R.YYSVYDLGK.N

Spot No.: **30**

Mascot score: **119**

Species: ***Fusarium oxysporum f. sp. cubense race 4***

Protein name: **Actin**

NCBI accession No.: **gi| 475667318**

Sequence coverage %: **53**

Matched peptides No.: **19**

Total peptides No.: **62**

Calculated Mr: **45188**

Calculated *p*I: **6.08**

Annotated PMF **spectra:**

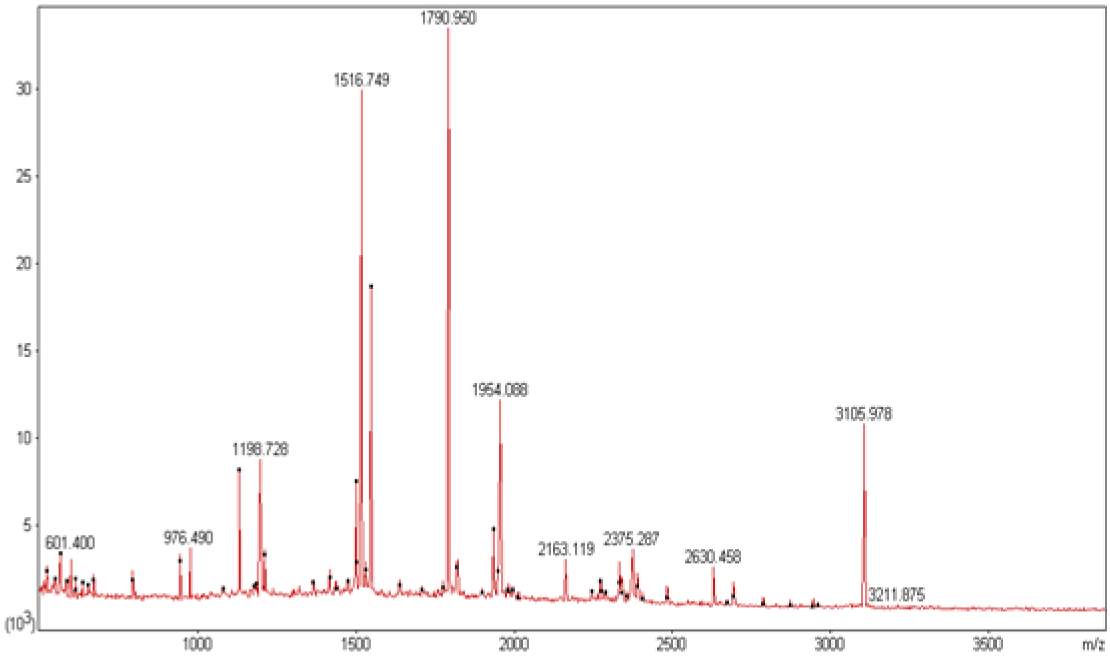

Probability Based Mowse Score:

Protein score is -10*Log(P), where P is the probability that the observed match is a random event. Protein scores greater than 75 are significant (p<0.05).

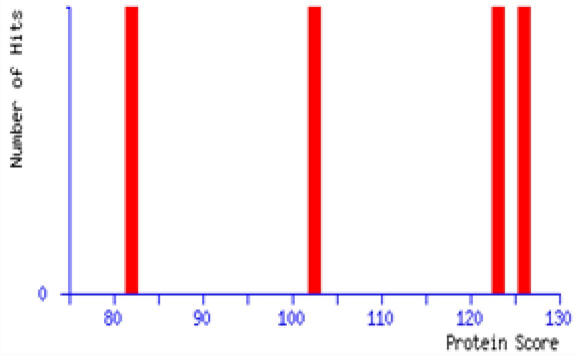

Matched peptide sequences: shown in Bold Red

```
  1  MGKLPRLSHR  PLDYLPASLL  LDQICHPIVR  CPTLASTLML  GDSGMCKAGF
 51  AGDDAPRAVF  PSIVGRPRHH  GIMIGMGQKD  SYVGDEAQSK  RGILTLRYPI
101  EHGVVTNWDD  MEKIWHHTFY  NELRVAPEEH  PVLLTEAPIN  PKSNREKMTQ
151  IVFETFNAPA  FYVSIQAVLS  LYASGRTTGI  VLDSGDGVTH  VVPIYEGFAL
201  PHAIARVDMA  GRDLTDYLMK  ILAERGYTFS  TTAEREIVRD  IKEKLCYVAL
251  DFEQEIQTAA  QSSSLEKSYE  LPDGQVITIG  NERFRAPEAL  FQPSVLGLES
301  GGIHVTTFNS  IMKCDVDVRK  DLYGNIVMSG  GTTMYPGLSD  RMQKEITALA
351  PSSMKVKIIA  PPERKYSVWI  GGSILASLST  FQQMWISKQE  YDESGPSIVH
401  RKCF
```

Matched (red) residues: 48–68, 92–113, 125–142, 177–206, 221–235, 245–283, 286–313, 320–341, 358–364, 389–401.

Matched peptides information:

Start	-	End	Observed	Mr(expt)	Mr(calc)	ppm	M	Peptide
48	-	57	976.4897	975.4824	975.4410	42.4	0	K.AGFAGDDAPR.A
58	-	68	1198.7279	1197.7206	1197.6982	18.7	0	R.AVFPSIVGRPR.H
92	-	97	672.4815	671.4742	671.4330	61.3	0	R.GILTLR.Y
98	-	113	1932.9029	1931.8956	1931.8724	12.0	0	R.YPIEHGVVTNWDDMEK.I
98	-	113	1948.9175	1947.9102	1947.8673	22.0	0	R.YPIEHGVVTNWDDMEK.I + Oxidation (M)
125	-	142	1954.0884	1953.0811	1953.0571	12.3	0	R.VAPEEHPVLLTEAPINPK.S
177	-	206	3105.9781	3104.9708	3104.6295	110	0	R.TTGIVLDSGDGVTHVVPIYEGFALPHAIAR.V
221	-	225	601.4004	600.3932	600.3595	56.1	0	K.ILAER.G
226	-	235	1132.5483	1131.5410	1131.5197	18.9	0	R.GYTFSTTAER.E
245	-	267	2630.4579	2629.4506	2629.2581	73.2	0	K.LCYVALDFEQEIQTAAQSSSLEK.S
268	-	283	1790.9495	1789.9422	1789.8846	32.2	0	K.SYELPDGQVITIGNER.F
286	-	313	2943.8382	2942.8309	2942.5212	105	0	R.APEALFQPSVLGLESGGIHVTTFNSIMK.C
286	-	313	2959.8376	2958.8303	2958.5161	106	0	R.APEALFQPSVLGLESGGIHVTTFNSIMK.C + Oxidation (M)
320	-	341	2375.2868	2374.2795	2374.1297	63.1	1	R.KDLYGNIVMSGGTTMYPGLSDR.M
320	-	341	2391.2738	2390.2665	2390.1246	59.4	1	R.KDLYGNIVMSGGTTMYPGLSDR.M + Oxidation (M)
320	-	341	2407.2989	2406.2916	2406.1196	71.5	1	R.KDLYGNIVMSGGTTMYPGLSDR.M + 2 Oxidation (M)
321	-	341	2247.1802	2246.1729	2246.0348	61.5	0	K.DLYGNIVMSGGTTMYPGLSDR.M
358	-	364	795.4856	794.4783	794.4650	16.8	0	K.IIAPPER.K
389	-	401	1516.7492	1515.7419	1515.6954	30.7	0	K.QEYDESGPSIVHR.K

Spot No.: **31**

Mascot score: **159**

Species: ***Fusarium oxysporum f. sp. cubense race 4***

Protein name: **phosphoglycerate kinase**

NCBI accession No.: **gi| 475667960**

Sequence coverage %: **57**

Matched peptides No.: **21**

Total peptides No.: **59**

Calculated Mr: **44835**

Calculated *p*I: **5.77**

Annotated PMF **spectra:**

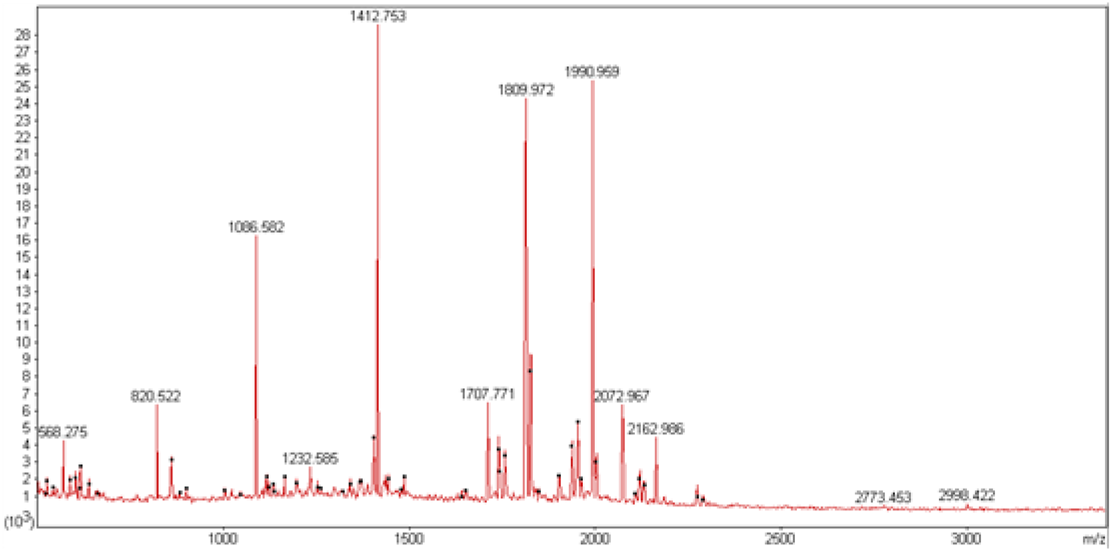

Probability Based Mowse Score:

Protein score is -10*Log(P), where P is the probability that the observed match is a random event.
Protein scores greater than 75 are significant (p<0.05).

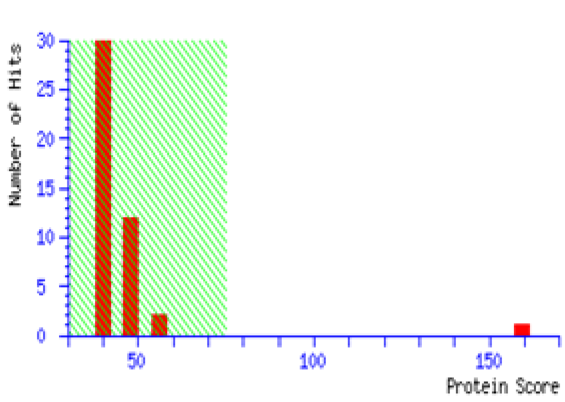

Matched peptide sequences: shown in Bold Red

```
  1  MSLSNKLSIT  DVDVKGKRVL  IRVDFNVPLD  ADKNITNNQR  IVGALPTIKY
 51  ALENGAKSVI  LMSHLGRPNG  SPNEKYSLKP  VVPELEKLLG  KKVTFAPDCV
101  GPEVEEIVNK  AEDGAVILLE  NLRFHIEEEG  SSKDKEGNKT  KADKAQVEAF
151  RKGLTALGDV  YINDAFGTAH  RAHSSMVGVD  LPQKASGFLV  KKELEYFAKA
201  LEEPQRPFLA  ILGGAKVSDK  IQLIDNLLDK  VNTLIICGGM  AFTFKKTLEG
251  VSIGNSLFDE  AGSKTVGNLV  EKAKAKGVKL  VLPVDYITAD  KFDKDANTGY
301  ATDKDGIPDG  WQGLDCGEES  VKLYKEAIAE  AKTILWNGPA  GVFEFEKFAS
351  GTKATLDAVV  DAVQKDGKIV  IIGGGDTATV  AKKYGVEDKL  SHVSTGGGAS
401  LELLEGKELP  GVTALSSK
```

Matched peptides information:

Start	-	End	Observed	Mr(expt)	Mr(calc)	ppm	M	Peptide
23	-	33	1232.5850	1231.5777	1231.6085	-25.0	0	R.VDFNVPLDADK.N
23	-	40	2072.9674	2071.9601	2072.0287	-33.1	1	R.VDFNVPLDADKNITNNQR.I
76	-	87	1401.7413	1400.7340	1400.7915	-41.0	0	K.YSLKPVVPELEK.L
92	-	110	2131.0086	2130.0013	2130.0667	-30.7	1	K.KVTFAPDCVGPEVEEIVNK.A
93	-	110	2002.8771	2001.8698	2001.9718	-50.9	0	K.VTFAPDCVGPEVEEIVNK.A
111	-	123	1412.7534	1411.7461	1411.7671	-14.8	0	K.AEDGAVILLENLR.F
124	-	133	1162.5477	1161.5404	1161.5302	8.80	0	R.FHIEEEGSSK.D
142	-	151	1134.5684	1133.5611	1133.5829	-19.2	1	K.ADKAQVEAFR.K
145	-	151	820.5223	819.5150	819.4239	111	0	K.AQVEAFR.K
152	-	171	2119.0557	2118.0484	2118.0858	-17.6	1	R.KGLTALGDVYINDAFGTAHR.A
153	-	171	1990.9585	1989.9512	1989.9908	-19.9	0	K.GLTALGDVYINDAFGTAHR.A
172	-	184	1368.6914	1367.6841	1367.6867	-1.91	0	R.AHSSMVGVDLPQK.A
193	-	199	899.4973	898.4900	898.4436	51.7	0	K.ELEYFAK.A
200	-	216	1809.9722	1808.9649	1809.0148	-27.6	0	K.ALEEPQRPFLAILGGAK.V
247	-	264	1823.7863	1822.7790	1822.8949	-63.5	0	K.TLEGVSIGNSLFDEAGSK.T
265	-	272	859.4871	858.4798	858.4811	-1.51	0	K.TVGNLVEK.A
280	-	294	1736.8318	1735.8245	1735.9396	-66.3	1	K.LVLPVDYITADKFDK.D
295	-	322	2998.4218	2997.4145	2997.2934	40.4	1	K.DANTGYATDKDGIPDGWQGLDCGEESVK.L
305	-	322	1961.7666	1960.7593	1960.8473	-44.9	0	K.DGIPDGWQGLDCGEESVK.L
333	-	347	1707.7714	1706.7641	1706.8668	-60.2	0	K.TILWNGPAGVFEFEK.F
390	-	407	1754.8226	1753.8153	1753.9210	-60.3	0	K.LSHVSTGGGASLELLEGK.E

Spot No.: **32**

Mascot score: **123**

Species: ***Fusarium oxysporum f. sp. cubense race 4***

Protein name: **elongation factor 1-gamma**

NCBI accession No.: **gi| 475667235** Sequence coverage %: **51**

Matched peptides No.: **19** Total peptides No.: **72**

Calculated Mr: **46170** Calculated *p*I: **5.64**

Annotated PMF **spectra:**

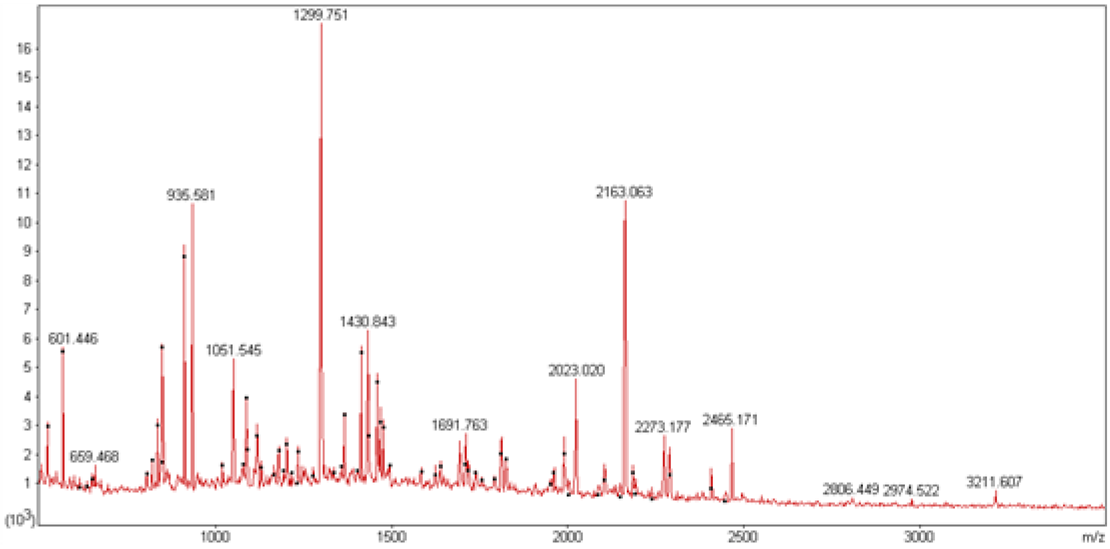

Probability Based Mowse Score:

Protein score is -10*Log(P), where P is the probability that the observed match is a random event.
Protein scores greater than 75 are significant (p<0.05).

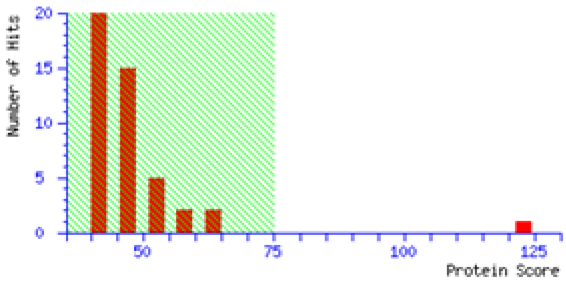

Matched peptide sequences: shown in Bold Red

```
  1 MAFGTLFTRE NNCRSTAIRA VAKANDIELN IVEAEKGNAT VEHLKANGLG
 51 KIPAFIGEDG FALSECIAIA IYITSQNEKT TLLGKTKQDY ASILKWMSFF
101 NSEVLPSLIA WFGPLKGDAP YNKKNVDDAS KASLKAFSVV EEHLIRNTFL
151 VGERITLADL FAAGIAVRGF QYFFDKQWRE ENPAVTRWFE TVRAQPIFAE
201 VAEKVELLET PALTNTPPKK PEQPKKEAKK EVKKEAAPAA EAAPASDEAP
251 AAPKAKHPLE ALGRPSRQYS NIKDHNEAMK YFWDNFNFEE YSIWKVDYKY
301 NEELTLTFMS NNLIGGFNNR LEGSRKYIFG CAAVYGENND SVIQGAFVIR
351 GQEHVPAFDV APDWESYNFE KLDPTNPEHR QFVEDAWGWE KPITVNGKEY
401 KLADGKVFK
```

Matched peptides information:

Start - End	Observed	Mr(expt)	Mr(calc)	ppm	M	Peptide
1 - 14	1716.8667	1715.8594	1715.7872	42.1	1	-.MAFGTLFTRENNCR.S
2 - 9	912.5768	911.5695	911.4865	91.0	0	M.AFGTLFTR.E
24 - 36	1457.7449	1456.7376	1456.7409	-2.24	0	K.ANDIELNIVEAEK.G
24 - 45	2407.2345	2406.2272	2406.2390	-4.90	1	K.ANDIELNIVEAEKGNATVEHLK.A
136 - 146	1299.7508	1298.7435	1298.6983	34.8	0	K.AFSVVEEHLIR.N
147 - 154	935.5809	934.5736	934.4872	92.4	0	R.NTFLVGER.I
155 - 168	1430.8434	1429.8361	1429.8293	4.78	0	R.ITLADLFAAGIAVR.G
169 - 176	1051.5449	1050.5376	1050.4811	53.8	0	R.GFQYFFDK.Q
188 - 193	837.5337	836.5264	836.4181	130	0	R.WFETVR.A
194 - 204	1202.6474	1201.6401	1201.6343	4.88	0	R.AQPIFAEVAEK.V
194 - 219	2806.4489	2805.4416	2805.5164	-26.6	1	R.AQPIFAEVAEKVELLETPALTNTPPK.K
205 - 219	1622.8732	1621.8659	1621.8927	-16.5	0	K.VELLETPALTNTPPK.K
234 - 254	1962.9287	1961.9214	1961.9694	-24.4	1	K.KEAAPAAEAAPASDEAPAAPK.A
257 - 267	1232.6378	1231.6305	1231.6785	-39.0	0	K.HPLEALGRPSR.Q
281 - 295	2088.0489	2087.0416	2086.9101	63.0	0	K.YFWDNFNFEEYSIWK.V
296 - 299	524.3021	523.2948	523.2642	58.6	0	K.VDYK.Y
300 - 320	2447.1908	2446.1835	2446.1587	10.2	0	K.YNEELTLTFMSNNLIGGFNNR.L
351 - 371	2465.1708	2464.1635	2464.0972	26.9	0	R.GQEHVPAFDVAPDWESYNFEK.L
372 - 380	1078.5562	1077.5489	1077.5203	26.6	0	K.LDPTNPEHR.Q

Spot No.: **33**

Mascot score: **132**

Species: ***Fusarium oxysporum f. sp. cubense race 4***

Protein name: **1,3-beta-glucanosyltransferase gel4**

NCBI accession No.: **gi| 475668458**

Sequence coverage %: **44**

Matched peptides No.: **22**

Total peptides No.: **77**

Calculated Mr: **59159**

Calculated *p*I: **4.83**

Annotated PMF **spectra:**

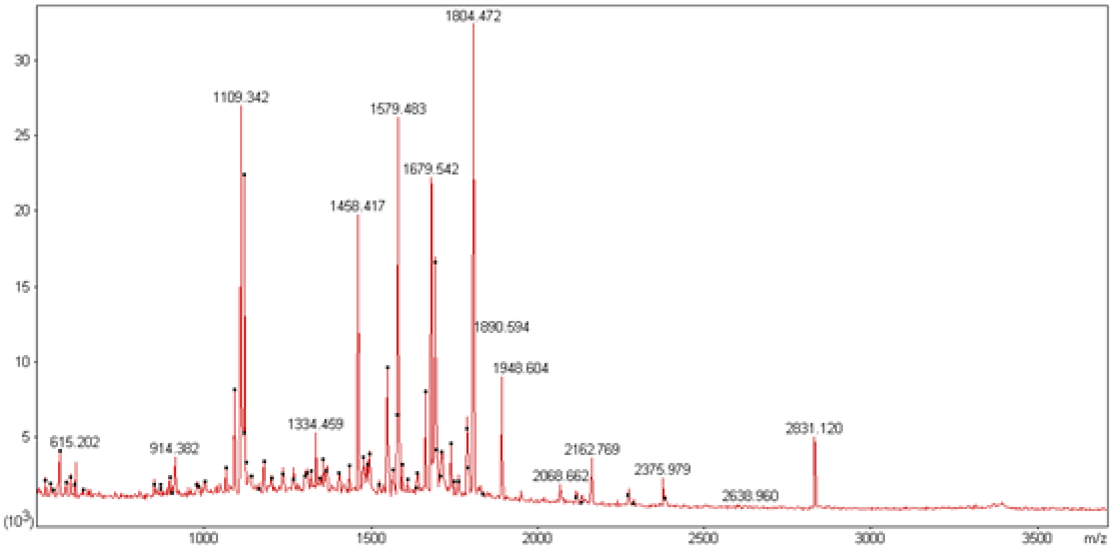

Probability Based Mowse Score:

Protein score is -10*Log(P), where P is the probability that the observed match is a random event.
Protein scores greater than 75 are significant (p<0.05).

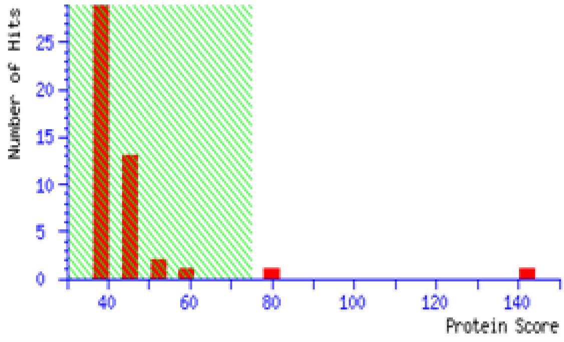

Matched peptide sequences: shown in Bold Red

```
  1 MKFSAAIVAA AATAASAKLE PITMKGSKLF YSNGTQFFMK GVAYQQDTAA
 51 AGETNDKTTK YIDPLADEDA CKRDIPLLKQ LGTNIIRTYA INPKADHKAC
101 MKLLDDAGIY VISDLSEPSV SINRDDPKWD VELYERYIGV VDELGQYDNV
151 VGFFAGNEVS NNVSNTQASA FVKAAVRDTK KHIKSKFSRW LGVGYASNDD
201 VDIREQIADY FNCGDDDSRI DYWGYNIYSW CGKSSMQDSG YADQAKFFEN
251 YSVPVFFAEY GCNEPDGAAG RIFDETTALY EEKVMTDVFS GGIVYMYFQE
301 ANDYGLVKIS KNGDAVKQKD FAQLQKKANA AKPSGVEEDS YKPTGKAATC
351 PEQSKNWRAN SVLPPVPDSD LCDCMVKSRS CVPADNLKAK DFNDIFGYIC
401 GQDKKICTAI NANATAGIYG AYSMCSNEAK LAYILDAYYT SQKSAADACD
451 FKGKATTQKA ESQDSCKSAL ASASKINEEV ATATHAVASS STGGSNSSSE
501 DDENFGLQAA SIARVFSLGD FAVGAYMAVA GVVGAGMVLL
```

Matched peptides information:

Start	-	End	Observed	Mr(expt)	Mr(calc)	ppm	M	Peptide
1	-	18	1695.5374	1694.5301	1694.9025	-220	1	-.MKFSAAIVAAAATAASAK.L + Oxidation (M)
19	-	28	1119.3237	1118.3164	1118.6005	-254	1	K.LEPITMKGSK.L + Oxidation (M)
41	-	57	1738.5057	1737.4984	1737.7806	-162	0	K.GVAYQQDTAAAGETNDK.T
41	-	60	2068.6617	2067.6544	2067.9709	-153	1	K.GVAYQQDTAAAGETNDKTTK.Y
80	-	87	914.3822	913.3749	913.5345	-175	0	K.QLGTNIIR.T
103	-	128	2831.1201	2830.1128	2830.4236	-110	1	K.LLDDAGIYVISDLSEPSVSINRDDPK.W
125	-	136	1564.4549	1563.4476	1563.7205	-175	1	R.DDPKWDVELYER.Y
129	-	136	1109.3417	1108.3344	1108.5189	-166	0	K.WDVELYER.Y
190	-	204	1679.5417	1678.5344	1678.7951	-155	0	R.WLGVGYASNDDVDIR.E
205	-	219	1804.4720	1803.4647	1803.7006	-131	0	R.EQIADYFNCGDDDSR.I
234	-	246	1403.3188	1402.3115	1402.5671	-182	0	K.SSMQDSGYADQAK.F + Oxidation (M)
272	-	283	1458.4172	1457.4099	1457.6926	-194	0	R.IFDETTALYEEK.V
320	-	326	849.2443	848.2371	848.4392	-238	0	K.DFAQLQK.K
328	-	346	1948.6045	1947.5972	1947.9538	-183	0	K.ANAAKPSGVEEDSYKPTGK.A
359	-	377	2116.6857	2115.6784	2115.9639	-135	0	R.ANSVLPPVPDSDLCDCMVK.S
359	-	377	2132.6592	2131.6519	2131.9588	-144	0	R.ANSVLPPVPDSDLCDCMVK.S + Oxidation (M)
359	-	379	2375.9786	2374.9713	2375.0920	-50.8	1	R.ANSVLPPVPDSDLCDCMVKSR.S + Oxidation (M)
380	-	388	1003.2703	1002.2630	1002.4804	-217	0	R.SCVPADNLK.A
389	-	404	1890.5942	1889.5869	1889.8618	-145	1	K.AKDFNDIFGYICGQDK.K
391	-	404	1691.5386	1690.5313	1690.7297	-117	0	K.DFNDIFGYICGQDK.K
431	-	443	1548.5062	1547.4989	1547.7871	-186	0	K.LAYILDAYYTSQK.S
444	-	452	984.2138	983.2065	983.4018	-199	0	K.SAADACDFK.G

Spot No.: **34**

Mascot score: **92**

Species: ***Fusarium oxysporum f. sp. cubense race 4***

Protein name: **1,3-beta-glucanosyltransferase gel4**

NCBI accession No.: **gi| 475668458**

Sequence coverage %: **42**

Matched peptides No.: **19**

Total peptides No.: **79**

Calculated Mr: **59159**

Calculated *p*I: **4.83**

Annotated PMF **spectra:**

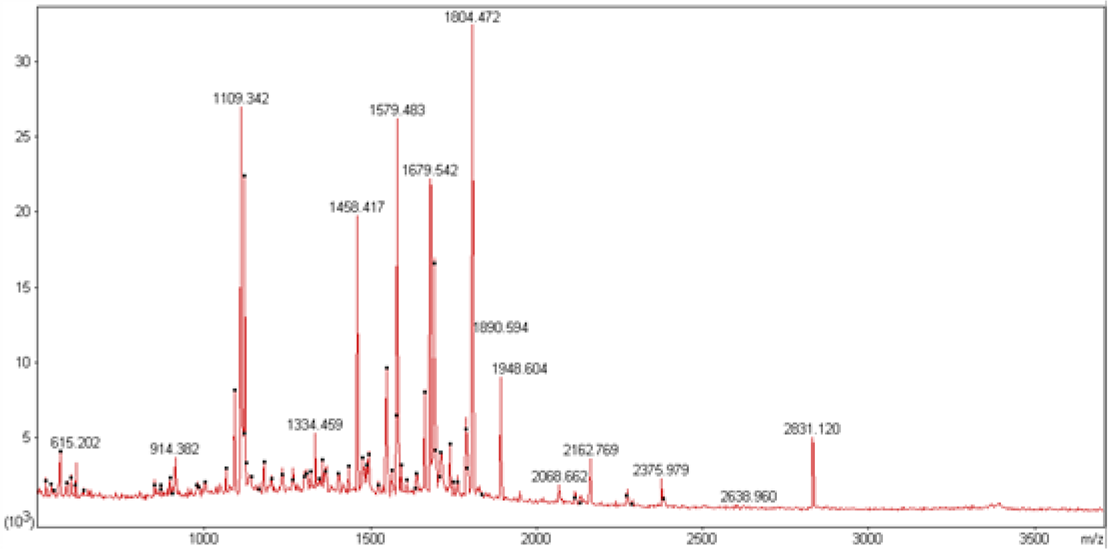

Probability Based Mowse Score:

Protein score is -10*Log(P), where P is the probability that the observed match is a random event.
Protein scores greater than 75 are significant (p<0.05).

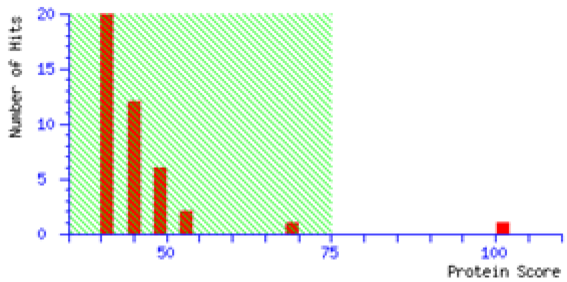

Matched peptide sequences: shown in **Bold Red**

```
  1 MKFSAAIVAA AATAASAKLE PITMKGSKLF YSNGTQFFMK GVAYQQDTAA
 51 AGETNDKTTK YIDPLADEDA CKRDIPLLKQ LGTNIIRTYA INPKADHKAC
101 MKLLDDAGIY VISDLSEPSV SINRDDPKWD VELYERYIGV VDELGQYDNV
151 VGFFAGNEVS NNVSNTQASA FVKAAVRDTK KHIKSKFSRW LGVGYASNDD
201 VDIREQIADY FNCGDDDSRI DYWGYNIYSW CGKSSMQDSG YADQAKFFEN
251 YSVPVFFAEY GCNEPDGAAG RIFDETTALY EEKVMTDVFS GGIVYMYFQE
301 ANDYGLVKIS KNGDAVKQKD FAQLQKKANA AKPSGVEEDS YKPTGKAATC
351 PEQSKNWRAN SVLPPVPDSD LCDCMVKSRS CVPADNLKAK DFNDIFGYIC
401 GQDKKICTAI NANATAGIYG AYSMCSNEAK LAYILDAYYT SQKSAADACD
451 FKGKATTQKA ESQDSCKSAL ASASKINEEV ATATHAVASS STGGSNSSSE
501 DDENFGLQAA SIARVFSLGD FAVGAYMAVA GVVGAGMVLL
```

Matched peptides information:

Start – End	Observed	Mr(expt)	Mr(calc)	ppm	M	Peptide
19 – 28	1119.4431	1118.4358	1118.6005	-147	1	K.LEPITMKGSK.L + Oxidation (M)
41 – 57	1738.5124	1737.5051	1737.7806	-159	0	K.GVAYQQDTAAAGETNDK.T
74 – 87	1593.5119	1592.5046	1592.9613	-287	1	R.DIPLLKQLGTNIIR.T
80 – 87	914.4343	913.4271	913.5345	-118	0	K.QLGTNIIR.T
103 – 128	2831.3179	2830.3106	2830.4236	-39.9	1	K.LLDDAGIYVISDLSEPSVSINRDDPK.W
129 – 136	1109.4175	1108.4102	1108.5189	-98.0	0	K.WDVELYER.Y
190 – 204	1679.5730	1678.5657	1678.7951	-137	0	R.WLGVGYASNDDVDIR.E
205 – 219	1804.4916	1803.4843	1803.7006	-120	0	R.EQIADYFNCGDDDSR.I
234 – 246	1403.3524	1402.3451	1402.5671	-158	0	K.SSMQDSGYADQAK.F + Oxidation (M)
247 – 271	2843.1410	2842.1337	2842.2333	-35.0	0	K.FFENYSVPVFFAEYGCNEPDGAAGR.I
272 – 283	1458.4576	1457.4503	1457.6926	-166	0	R.IFDETTALYEEK.V
320 – 326	849.3285	848.3212	848.4392	-139	0	K.DFAQLQK.K
328 – 346	1948.7043	1947.6970	1947.9538	-132	0	K.ANAAKPSGVEEDSYKPTGK.A
359 – 377	2116.7362	2115.7289	2115.9639	-111	0	R.ANSVLPPVPDSDLCDCMVK.S
359 – 377	2132.7105	2131.7032	2131.9588	-120	0	R.ANSVLPPVPDSDLCDCMVK.S + Oxidation (M)
359 – 379	2376.0800	2375.0727	2375.0920	-8.10	1	R.ANSVLPPVPDSDLCDCMVKSR.S + Oxidation (M)
389 – 404	1890.6650	1889.6577	1889.8618	-108	1	K.AKDFNDIFGYICGQDK.K
391 – 404	1691.5783	1690.5710	1690.7297	-93.9	0	K.DFNDIFGYICGQDK.K
431 – 443	1548.4861	1547.4788	1547.7871	-199	0	K.LAYILDAYYTSQK.S

Spot No.: **35**

NCBI accession No.: **gi|477513214**

Plant species: ***Fusarium oxysporum f. sp. cubense race 1***

Protein name：**Beta-hexosaminidase subunit A1**

Mascot score: **93** Sequence coverage %: **4**

The number of matched peptides with p≤0.05: **1**

Calculated Mr: **34198** Calculated *Pi*: **6.25**

Annotated MS spectra:

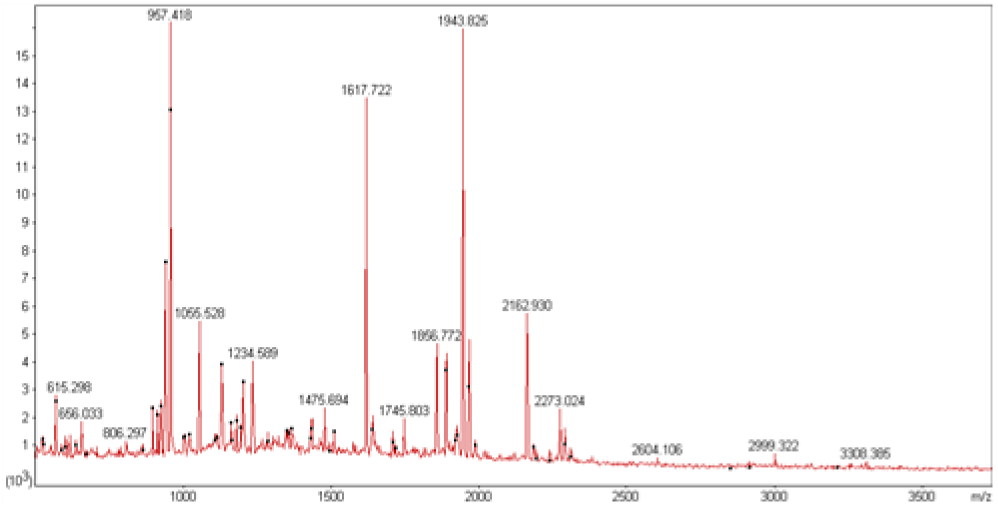

Probability Based Mowse Score:

Ions score is -10*Log(P), where P is the probability that the observed match is a random event.
Individual ions scores > 48 indicate identity or extensive homology (p<0.05).
Protein scores are derived from ions scores as a non-probabilistic basis for ranking protein hits.

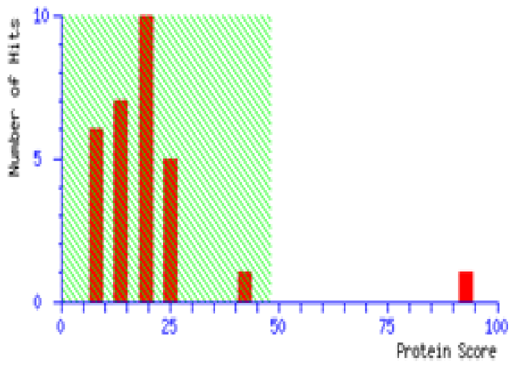

Matched peptide sequences: shown in Bold Red

```
  1  MASNLYPHRG  FMLDTGRKFF  PVKAILHLLT  LLHQYNFNVF  HWHIYDAESF
 51  PLLWPAGEGL  TNASVKYSQT  HTYYTPSDIQ  NVISYAENLG  ILVYPETDMP
101  GHSDIWGIWK  KDLVVGKASL  KKPDAQLDIR  QNNKQVYDYI  RSLVSTVDGY
151  FGSPYHHFGG  DEVAYMWNTR  DDNKLFNSFL  NWLKTLTPKK  SVILWDDPLT
201  DSEKSITLSE  DWIIQTWHKG  TTQKILKKGH  RVIVSESDTF  YIGNADADKI
251  SSFVFPKSSK  VLGFEVAWFT  SQDDDPSDLD  QDWIIDPLKA  ASKIRRK
```

Matched peptides information:

Query	Start - End	Observed	Mr(expt)	Mr(calc)	ppm	M	Score	Expect	Rank	U	Peptide
[illegible]	191 - 204	1617.7220	1616.7147	1616.7934	-48.6	0	93	1.7e-06	1	U	K.SVILWDDPLTDSEK.S

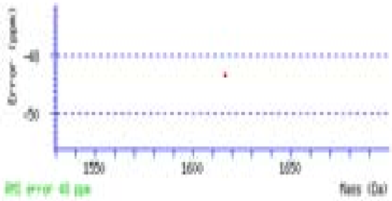

Annotated ion spectra of the matched peptides with p≤0.05:

CID No.: **35-1617.76**

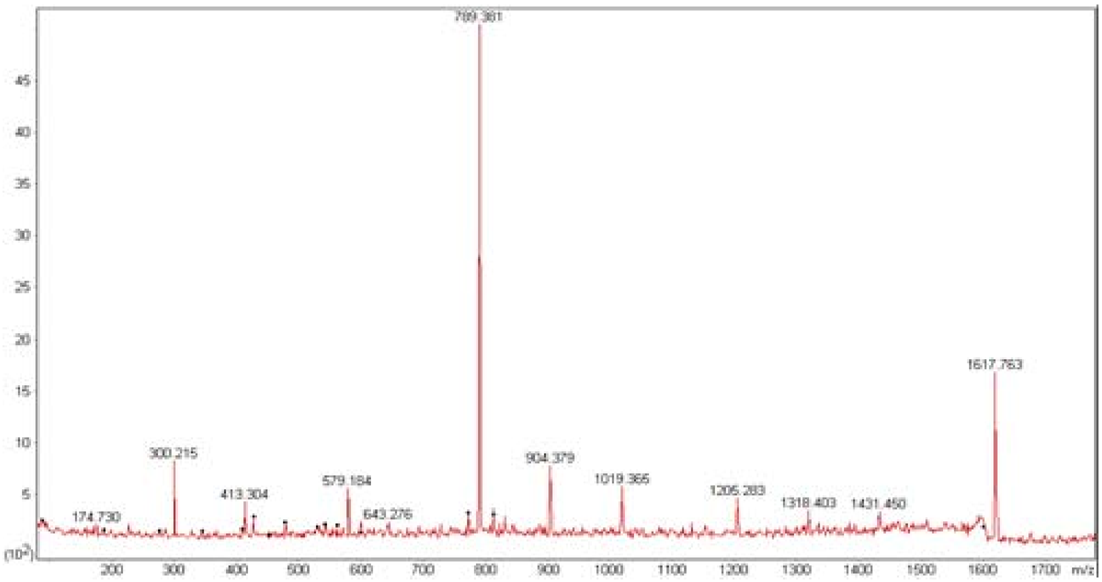

MS/MS Fragmentation of **K.SVILWDDPLTDSEK.S**

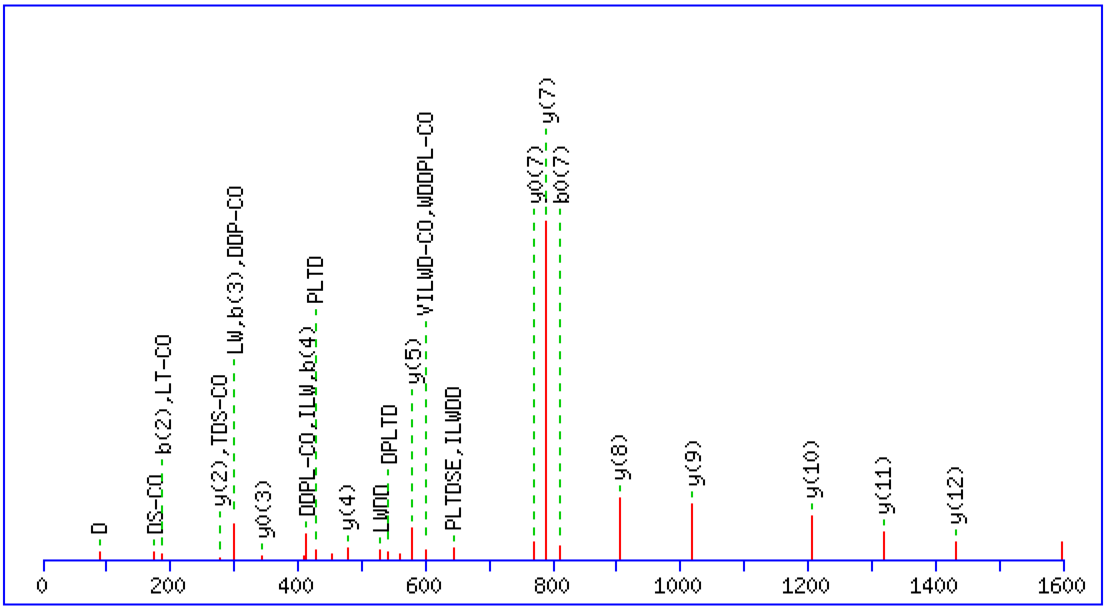

Monoisotopic mass of neutral peptide Mr(calc): 1616.7934

#	Immon.	a	a^0	b	b^0	d	d'	Seq.	v	w	w'	y	y*	y^0	#
1	60.0444	60.0444	42.0338	**88.0393**	70.0287	44.0495		S							14
2	72.0808	159.1128	141.1022	**187.1077**	169.0972	145.0972		V	1486.7060	1499.7264		1530.7686	1513.7421	1512.7581	13
3	86.0964	272.1969	254.1863	**300.1918**	282.1812	244.1656	258.1812	I	1373.6220	1386.6424	1400.6580	***1431.7002***	1414.6737	1413.6896	12
4	86.0964	385.2809	367.2704	**413.2758**	395.2653	343.2340		L	1260.5379	1259.5426		***1318.6161***	1301.5896	1300.6056	11
5	159.0917	571.3602	553.3497	**599.3552**	581.3446			W	1074.4586			***1205.5321***	1188.5055	1187.5215	10
6	88.0393	686.3872	668.3766	714.3821	696.3715	642.3974		D	959.4316	958.4364		***1019.4528***	1002.4262	1001.4422	9
7	88.0393	801.4141	783.4036	829.4090	811.3985	757.4243		D	844.4047	843.4094		***904.4258***	887.3993	886.4153	8
8	70.0651	898.4669	880.4563	926.4618	908.4512	872.4512		P	747.3519	746.3567		***789.3989***	772.3723	771.3883	7
9	86.0964	1011.5510	993.5404	1039.5459	1021.5353	969.5040		L	634.2679	633.2726		692.3461	675.3196	674.3355	6
10	74.0600	1112.5986	1094.5881	1140.5936	1122.5830	1096.6037	1098.5830	T	533.2202	546.2406	548.2198	***579.2620***	562.2355	561.2515	5
11	88.0393	1227.6256	1209.6150	1255.6205	1237.6099	1183.6358		D	418.1932	417.1980		***478.2144***	461.1878	460.2038	4
12	60.0444	1314.6576	1296.6470	1342.6525	1324.6420	1298.6627		S	331.1612	330.1660		363.1874	346.1609	345.1769	3
13	102.0550	1443.7002	1425.6896	1471.6951	1453.6846	1385.6947		E	202.1186	201.1234		***276.1554***	259.1288	258.1448	2
14	101.1073							K	74.0237	73.0284		147.1128	130.0863		1

Seq	ya	yb	Seq	ya	yb	Seq	ya	yb
**VI**	185.1648	213.1598	**VIL**	298.2489	326.2438	**VILW**	484.3282	512.3231
**VILWD**	599.3552	627.3501	**IL**	199.1805	227.1754	**ILW**	385.2598	413.2547
**ILWD**	500.2867	528.2817	**ILWDD**	615.3137	643.3086	**LW**	272.1757	300.1707
**LWD**	387.2027	415.1976	**LWDD**	502.2296	530.2245	**LWDDP**	599.2824	627.2773
**WD**	274.1186	302.1135	**WDD**	389.1456	417.1405	**WDDP**	486.1983	514.1932
**WDDPL**	599.2824	627.2773	**DD**	203.0662	231.0612	**DDP**	300.1190	328.1139
**DDPL**	413.2031	441.1980	**DDPLT**	514.2508	542.2457	**DDPLTD**	629.2777	657.2726
**DP**	185.0921	213.0870	**DPL**	298.1761	326.1710	**DPLT**	399.2238	427.2187
**DPLTD**	514.2508	542.2457	**DPLTDS**	601.2828	629.2777	**PL**	183.1492	211.1441
**PLT**	284.1969	312.1918	**PLTD**	399.2238	427.2187	**PLTDS**	486.2558	514.2508
**PLTDSE**	615.2984	643.2933	**LT**	187.1441	215.1390	**LTD**	302.1710	330.1660
**LTDS**	389.2031	417.1980	**LTDSE**	518.2457	546.2406	**TD**	189.0870	217.0819
**TDS**	276.1190	304.1139	**TDSE**	405.1616	433.1565	**DS**	175.0713	203.0662
**DSE**	304.1139	332.1088	**SE**	189.0870	217.0819			

Spot No.: **36**

Mascot score: **162**

Species: ***Fusarium oxysporum f. sp. cubense race 4***

Protein name: **phosphoglycerate kinase**

NCBI accession No.: **gi| 475667960** Sequence coverage %: **55**

Matched peptides No.: **20** Total peptides No.: **56**

Calculated Mr: **44835** Calculated *p*I: **5.77**

Annotated PMF **spectra:**

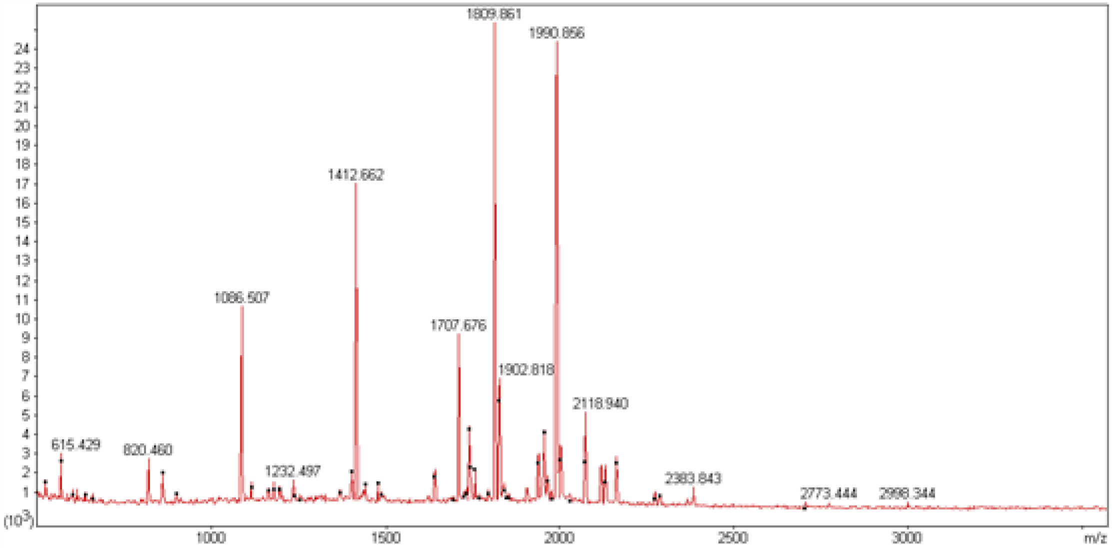

Probability Based Mowse Score:

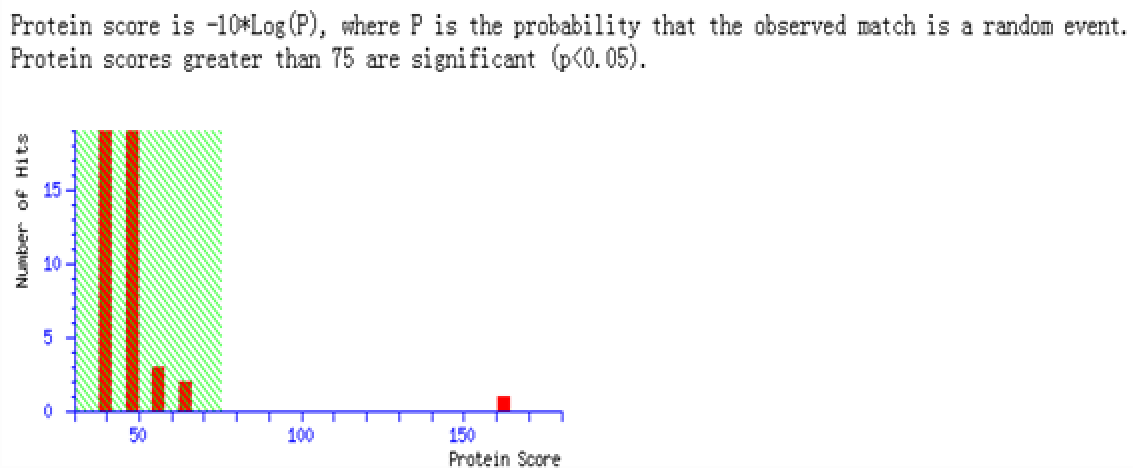

Matched peptide sequences: shown in Bold Red

```
  1  MSLSNKLSIT  DVDVKGKRVL  IRVDFNVPLD  ADKNITNNQR  IVGALPTIKY
 51  ALENGAKSVI  LMSHLGRPNG  SPNEKYSLKP  VVPELEKLLG  KKVTFAPDCV
101  GPEVEEIVNK  AEDGAVILLE  NLRFHIEEEG  SSKDKEGNKT  KADKAQVEAF
151  RKGLTALGDV  YINDAFGTAH  RAHSSMVGVD  LPQKASGFLV  KKELEYFAKA
201  LEEPQRPFLA  ILGGAKVSDK  IQLIDNLLDK  VNTLIICGGM  AFTFKKTLEG
251  VSIGNSLFDE  AGSKTVGNLV  EKAKAKGVKL  VLPVDYITAD  KFDKDANTGY
301  ATDKDGIPDG  WQGLDCGEES  VKLYKEAIAE  AKTILWNGPA  GVFEFEKFAS
351  GTKATLDAVV  DAVQKDGKIV  IIGGGDTATV  AKKYGVEDKL  SHVSTGGGAS
401  LELLEGKELP  GVTALSSK
```

Matched peptides information:

Start	-	End	Observed	Mr(expt)	Mr(calc)	ppm	M	Peptide
23	-	33	1232.4972	1231.4899	1231.6085	-96.3	0	R.VDFNVPLDADK.N
23	-	40	2072.8758	2071.8685	2072.0287	-77.3	1	R.VDFNVPLDADKNITNNQR.I
34	-	40	859.4474	858.4401	858.4308	10.9	0	K.NITNNQR.I
76	-	87	1401.6441	1400.6368	1400.7915	-110	0	K.YSLKPVVPELEK.L
92	-	110	2130.9019	2129.8946	2130.0667	-80.8	1	K.KVTFAPDCVGPEVEEIVNK.A
93	-	110	2002.8168	2001.8095	2001.9718	-81.0	0	K.VTFAPDCVGPEVEEIVNK.A
111	-	123	1412.6620	1411.6547	1411.7671	-79.6	0	K.AEDGAVILLENLR.F
124	-	133	1162.4208	1161.4135	1161.5302	-100	0	R.FHIEEEGSSK.D
145	-	151	820.4603	819.4530	819.4239	35.5	0	K.AQVEAFR.K
152	-	171	2118.9398	2117.9325	2118.0858	-72.4	1	R.KGLTALGDVYINDAFGTAHR.A
153	-	171	1990.8559	1989.8486	1989.9908	-71.5	0	K.GLTALGDVYINDAFGTAHR.A
172	-	184	1368.5882	1367.5809	1367.6867	-77.4	0	R.AHSSMVGVDLPQK.A
193	-	199	899.4319	898.4247	898.4436	-21.1	0	K.ELEYFAK.A
200	-	216	1809.8613	1808.8540	1809.0148	-88.9	0	K.ALEEPQRPFLAILGGAK.V
247	-	264	1823.7140	1822.7067	1822.8949	-103	0	K.TLEGVSIGNSLFDEAGSK.T
280	-	294	1736.7426	1735.7353	1735.9396	-118	1	K.LVLPVDYITADKFDK.D
295	-	322	2998.3440	2997.3367	2997.2934	14.4	1	K.DANTGYATDKDGIPDGWQGLDCGEESVK.L
305	-	322	1961.6835	1960.6762	1960.8473	-87.2	0	K.DGIPDGWQGLDCGEESVK.L
333	-	347	1707.6761	1706.6688	1706.8668	-116	0	K.TILWNGPAGVFEFEK.F
390	-	407	1754.7335	1753.7262	1753.9210	-111	0	K.LSHVSTGGGASLELLEGK.E

Spot No.: **37**

Mascot score: **191**

Species: ***Fusarium oxysporum Fo5176***

Protein name: **adenosylhomocysteinase**

NCBI accession No.: **gi| 342873590**　　Sequence coverage %: **57**

Matched peptides No.: **29**　　Total peptides No.: **85**

Calculated Mr: **49506**　　Calculated *p*I: **5.57**

Annotated PMF **spectra:**

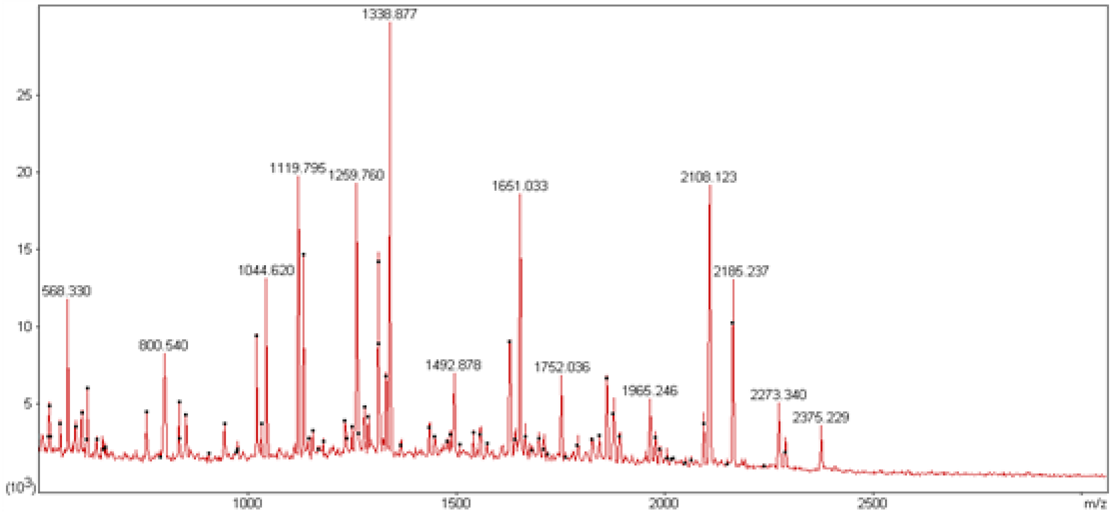

Probability Based Mowse Score:

Protein score is -10*Log(P), where P is the probability that the observed match is a random event.
Protein scores greater than 75 are significant (p<0.05).

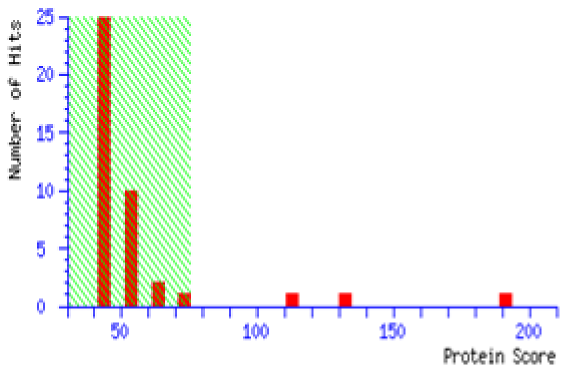

Matched peptide sequences: shown in Bold Red

```
  1 MSAPAHKFKV ADLSLAAFGR KEIELAENEM PGLMQTRAKY AADQPLAGAR
 51 IAGCLHMTIQ TAVLIETLTA LGAEVTWTSC NIFSTQDHAA AAIAAAGVPV
101 FAWKGETEEE YNWCLEQQLT AFKDNKKLNL ILDDGGDLTT LVHQKYPEML
151 KDCFGVSEET TTGVHHLYRM LKDGKLLVPA INVNDSVTKS KFDNLYGCRE
201 SLVDGIKRAT DVMIAGKVAV VAGFGDVGKG CAMALHGMGA RVLVTEIDPI
251 NALQAAMAGY QVTTMEKAAK VGQIFVTTTG CRDILTGEHF EAMPNDAIVC
301 NIGHFDIEID VAWLKANASS VQNIKPQVDR FLMPNGRHII LLAEGRLVNL
351 GCATGHSSFV MSCSFTNQVL AQIMLYKAAD KAWGEKYVEF AKTDKLDVGV
401 YVLPKILDEE VARLHLDHCQ AELSTLSKVQ AEYLGLTVEG PFKADIYRY
```

Matched peptides information:

Start	-	End	Observed	Mr(expt)	Mr(calc)	ppm	M	Peptide
10	-	20	1119.7945	1118.7872	1118.6084	160	0	K.VADLSLAAFGR.K
10	-	21	1247.7431	1246.7358	1246.7034	26.0	1	K.VADLSLAAFGRK.E
21	-	37	1989.1822	1988.1749	1987.9706	103	1	R.KEIELAENEMPGLMQTR.A
22	-	37	1861.0623	1860.0550	1859.8757	96.4	0	K.EIELAENEMPGLMQTR.A
22	-	37	1877.0492	1876.0419	1875.8706	91.3	0	K.EIELAENEMPGLMQTR.A + Oxidation (M)
22	-	37	1893.0562	1892.0489	1891.8655	96.9	0	K.EIELAENEMPGLMQTR.A + 2 Oxidation (M)
40	-	50	1132.7388	1131.7315	1131.5672	145	0	K.YAADQPLAGAR.I
105	-	123	2375.2290	2374.2217	2374.0423	75.6	0	K.GETEEEYNWCLEQQLTAFK.D
127	-	145	2093.3186	2092.3113	2092.1528	75.8	1	K.KLNLILDDGGDLTTLVHQK.Y
128	-	145	1965.2455	1964.2382	1964.0579	91.8	0	K.LNLILDDGGDLTTLVHQK.Y
152	-	169	2108.1231	2107.1158	2106.9429	82.1	0	K.DCFGVSEETTTGVHHLYR.M
176	-	189	1482.9758	1481.9685	1481.8453	83.1	0	K.LLVPAINVNDSVTK.S
190	-	199	1259.7601	1258.7528	1258.5764	140	1	K.SKFDNLYGCR.E
192	-	199	1044.6200	1043.6127	1043.4495	156	0	K.FDNLYGCR.E
209	-	229	2005.1455	2004.1382	2004.0714	33.3	1	R.ATDVMIAGKVAVVAGFGDVGK.G
209	-	229	2021.0990	2020.0917	2020.0663	12.6	1	R.ATDVMIAGKVAVVAGFGDVGK.G + Oxidation (M)
230	-	241	1231.7455	1230.7382	1230.5420	159	0	K.GCAMALHGMGAR.V
230	-	241	1263.7688	1262.7615	1262.5318	182	0	K.GCAMALHGMGAR.V + 2 Oxidation (M)
271	-	282	1338.8774	1337.8701	1337.6762	145	0	K.VGQIFVTTTGCR.D
316	-	330	1627.0193	1626.0120	1625.8465	101	0	K.ANASSVQNIKPQVDR.F
331	-	337	834.5812	833.5740	833.4218	183	0	R.FLMPNGR.H
338	-	346	1021.7539	1020.7466	1020.6080	136	0	R.HIILLAEGR.L
387	-	392	756.5286	755.5214	755.3854	180	0	K.YVEFAK.T
393	-	405	1446.9360	1445.9287	1445.8130	80.0	1	K.TDKLDVGVYVLPK.I
406	-	413	944.6771	943.6698	943.4974	183	0	K.ILDEEVAR.L
414	-	428	1752.0363	1751.0290	1750.8672	92.4	0	R.LHLDHCQAELSTLSK.V
429	-	443	1651.0328	1650.0255	1649.8665	96.4	0	K.VQAEYLGLTVEGPFK.A
444	-	448	637.4961	636.4888	636.3231	260	0	K.ADIYR.Y
444	-	449	800.5403	799.5331	799.3864	183	1	K.ADIYRY.-

Spot No.: **38**

Mascot score: **206**

Species: ***Fusarium oxysporum f. sp. cubense race 4***

Protein name: **ATP synthase subunit beta, mitochondrial**

NCBI accession No.: **gi| 475664589**

Sequence coverage %: **71**

Matched peptides No.: **30**

Total peptides No.: **68**

Calculated Mr: **55197**

Calculated *p*I: **5.27**

Annotated PMF spectra:

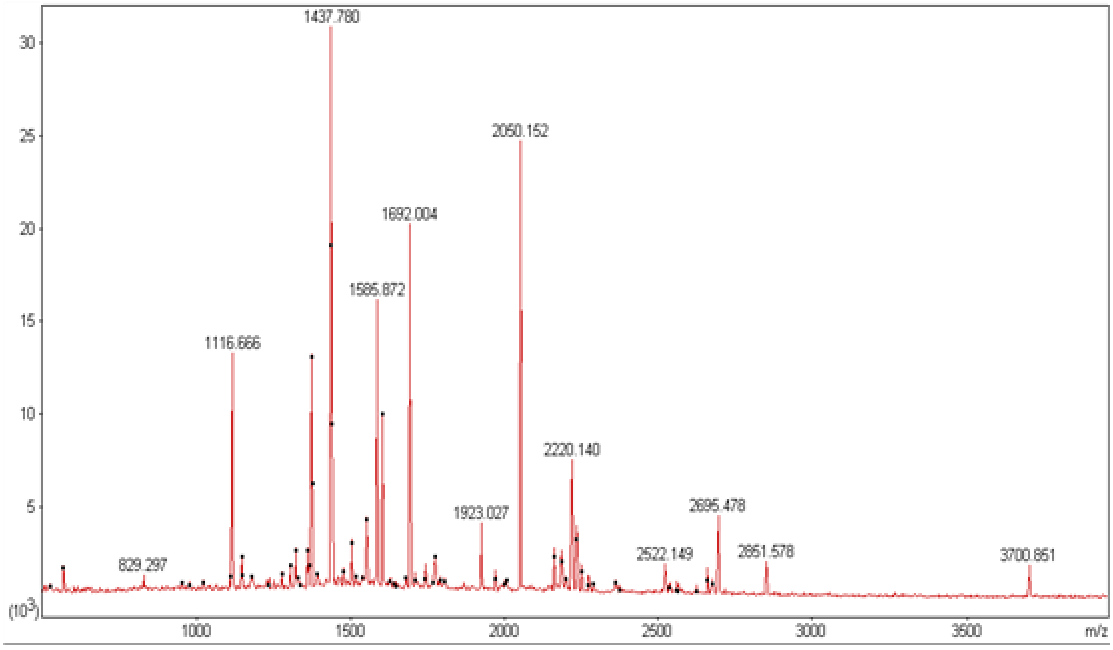

Probability Based Mowse Score:

Protein score is -10*Log(P), where P is the probability that the observed match is a random event. Protein scores greater than 75 are significant (p<0.05).

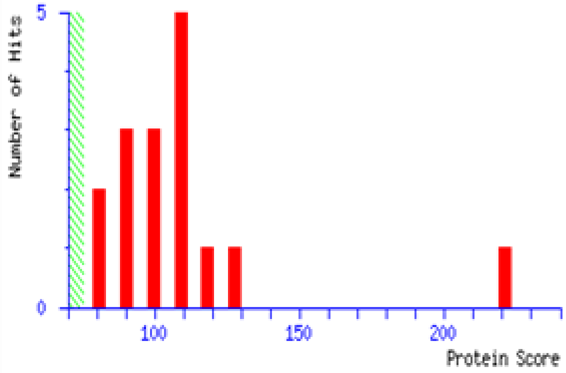

Matched peptide sequences: shown in Bold Red

```
  1 MFKSGISSFA RAARPAVLPR RALRPSSLRL PISSRWASTA SVGTGKIHQV
 51 IGAVVDVKFD TQKLPAILNS LETENNGQKL VLEVSQHLGE NVVRCIAMDG
101 TEGLVRGASA QDTGAPITIP VGPATLGRIM NVTGDPIDER GPIKTDKRLP
151 IHTEAPEFVE QSTSAEVLVT GIKVVDLLAP YARGGKIGLF GGAGVGKTVF
201 IQELINNIAK AHGGYSVFTG VGERTREGND LYHEMQETSV IQLDGESKVA
251 LVFGQMNEPP GARARVALTG LTVAEYFRDE EGQDVLLFID NIFRFTQAGS
301 EVSALLGRIP SAVGYQPTLA VDMGGMQERI TTTTKGSITS VQAVYVPADD
351 LTDPAPATTF AHLDATTVLS RGISELGIYP AVDPLDSKSR MLDPRVVGQE
401 HYDVATRVQQ ILQEYKSLQD IIAILGMDEL SEADKLTVER ARKIQRFLSQ
451 PFTVAQVFTG IEGKLVDLKE TINSFKAILN GEGDNLPEGA FYMVGDFASA
501 KAKGEKILAE LEGQ
```

Matched peptides information:

Start	-	End	Observed	Mr (expt)	Mr (calc)	ppm	M	Peptide
47	-	58	1277.7650	1276.7577	1276.7503	5.79	0	K.IHQVIGAVVDVK.F
64	-	79	1740.9252	1739.9179	1739.9053	7.24	0	K.LPAILNSLETENNGQK.L
80	-	94	1692.0044	1690.9971	1690.9366	35.8	0	K.LVLEVSQHLGENVVR.C
95	-	106	1321.6979	1320.6906	1320.6166	56.0	0	R.CIAMDGTEGLVR.G
95	-	106	1337.6690	1336.6617	1336.6115	37.6	0	R.CIAMDGTEGLVR.G + Oxidation (M)
107	-	128	2050.1518	2049.1445	2049.0855	28.8	0	R.GASAQDTGAPITIPVGPATLGR.I
129	-	140	1359.7078	1358.7005	1358.6500	37.2	0	R.IMNVTGDPIDER.G
129	-	140	1375.6401	1374.6328	1374.6449	-8.81	0	R.IMNVTGDPIDER.G + Oxidation (M)
148	-	173	2851.5783	2850.5710	2850.5127	20.5	1	K.RLPIHTEAPEFVEQSTSAEVLVTGIK.V
149	-	173	2695.4776	2694.4703	2694.4116	21.8	0	R.LPIHTEAPEFVEQSTSAEVLVTGIK.V
174	-	183	1116.6661	1115.6588	1115.6339	22.4	0	K.VVDLLAPYAR.G
187	-	197	975.5551	974.5478	974.5549	-7.34	0	K.IGLFGGAGVGK.T
198	-	210	1502.8862	1501.8789	1501.8504	19.0	0	K.TVFIQELINNIAK.A
227	-	248	2522.1491	2521.1418	2521.1278	5.55	0	R.EGNDLYHEMQETSVIQLDGESK.V
227	-	248	2538.1455	2537.1382	2537.1228	6.10	0	R.EGNDLYHEMQETSVIQLDGESK.V + Oxidation (M)
249	-	263	1585.8717	1584.8644	1584.8082	35.4	0	K.VALVFGQMNEPPGAR.A
249	-	263	1601.8439	1600.8366	1600.8032	20.9	0	K.VALVFGQMNEPPGAR.A + Oxidation (M)
266	-	278	1439.8688	1438.8615	1438.7820	55.3	0	R.VALTGLTVAEYFR.D
279	-	294	1923.0271	1922.0198	1921.9422	40.4	0	R.DEEGQDVLLFIDNIFR.F
295	-	308	1435.7754	1434.7681	1434.7467	14.9	0	R.FTQAGSEVSALLGR.I
309	-	329	2220.1396	2219.1323	2219.0715	27.4	0	R.IPSAVGYQPTLAVDMGGMQER.I
309	-	329	2236.1207	2235.1134	2235.0664	21.0	0	R.IPSAVGYQPTLAVDMGGMQER.I + Oxidation (M)
309	-	329	2252.1076	2251.1003	2251.0613	17.3	0	R.IPSAVGYQPTLAVDMGGMQER.I + 2 Oxidation (M)
336	-	371	3700.6507	3699.6434	3699.6632	-5.35	0	K.GSITSVQAVYVPADDLTDPAPATTFAHLDATTVLSR.G
372	-	388	1773.9372	1772.9299	1772.9196	5.81	0	R.GISELGIYPAVDPLDSK.S
396	-	407	1373.7253	1372.7180	1372.6735	32.4	0	R.VVGQEHYDVATR.V
408	-	416	1148.6559	1147.6486	1147.6237	21.7	0	R.VQQILQEYK.S
417	-	440	2659.4430	2658.4357	2658.3786	21.5	1	K.SLQDIIAILGMDELSEADKLTVER.A
417	-	440	2675.4041	2674.3968	2674.3735	8.73	1	K.SLQDIIAILGMDELSEADKLTVER.A + Oxidation (M)
447	-	464	1969.0687	1968.0614	1968.0357	13.1	0	R.FLSQPFTVAQVFTGIEGK.L

Spot No.: **39**

Mascot score: **90**

Species: ***Fusarium oxysporum f. sp. cubense race 1***

Protein name: **l-amino acid oxidase**

NCBI accession No.: **gi| 477517233**

Sequence coverage %: **24**

Matched peptides No.: **17**

Total peptides No.: **46**

Calculated Mr: **67814**

Calculated *p*I: **5.69**

Annotated PMF **spectra:**

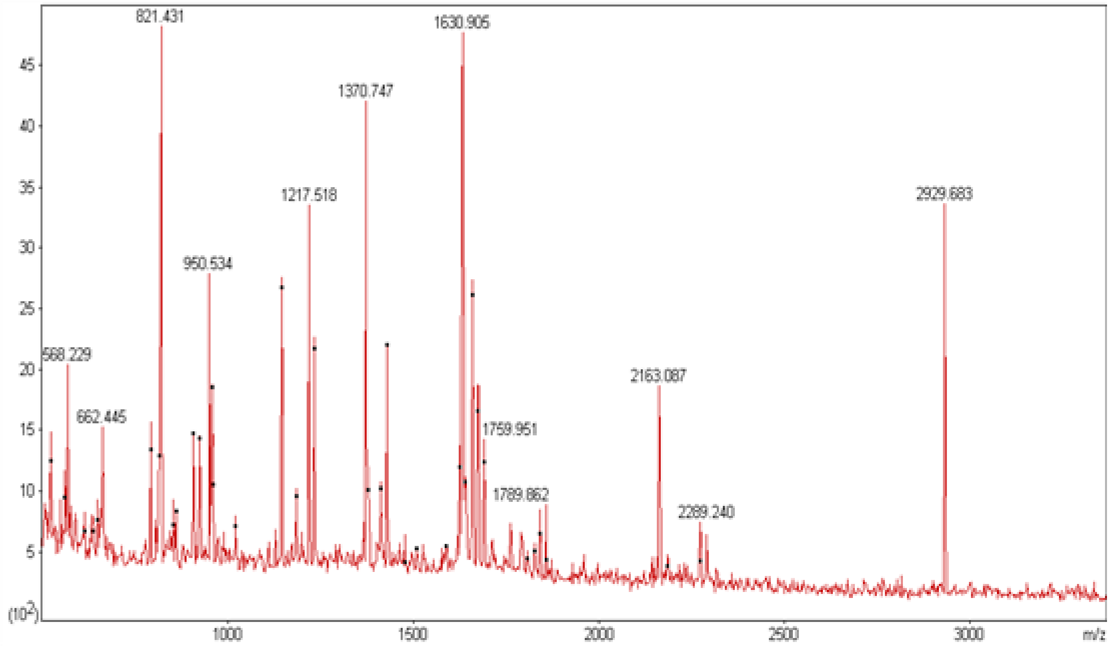

Probability Based Mowse Score:

Protein score is -10*Log(P), where P is the probability that the observed match is a random event.
Protein scores greater than 75 are significant (p<0.05).

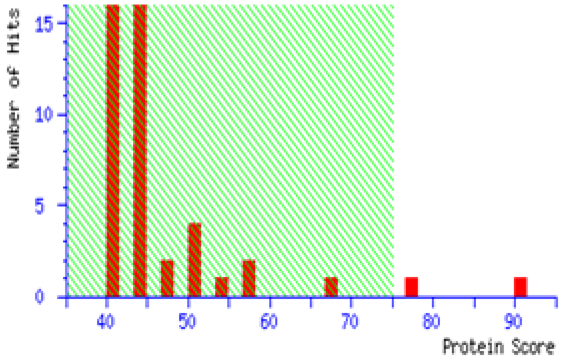

Matched peptide sequences: shown in Bold Red

1 MVKFTDEPWG ASVLVLLFLS NAHGTKLPLK AQQTHLCADK PQMQNFDSVG

51 AWFDDVAKLN CTSVSKAPNA SIAIVGGGVS GLTTALMLDS IGLHNWDIIE

101 ASDRVGGRFR TK**FVGGTKEF** **AEMGPMR**LPY TVTYKSDNST YEYTEHRLTF

151 QLAETLNEMN GNDSKWKVDF ISWIQHHPNE LIAWGTGRHP DGRIPTRADI

201 HANSSLGKPP AIVSTEYNET KHRMNEILKN ETMLK**AIQAD** **VWR**SHKFVMS

251 QGYDDWSEQC MMREAFHASE NITDAIWTAT DYDVVWDEMV HNSNLALDGT

301 KDSLGETEWK **CVDGGFNRLT** **DAFIPHVSDR** LVLNRKIGKL ESVKGEDGQT

351 QTRLSWYPSV KNRTFESK**DY** **DYTIMAVPFT** **MTRFMALPSF** **SSVLGRAISE**

401 **AGLR**FKSACK **VSLLFSER**FW EKGERPIFGG YSIPESRPIG ALYYPVYGLN

451 ESRPGLITHY R**GGDWSDRYV** **SFSDEEHVQT** **VLDAIVSLHG** **EQARELYTGD**

501 **YER**LCWLQDE HTATSWCRPD VEQHNLYIPA YHQTEHNTIF IGEHTAPTQA

551 WISSAIYSAA R**GTIQLLLEL** **GMVEEAK**EIN RRWMGRWIRD ETKP

Matched peptides information:

Start - End	Observed	Mr(expt)	Mr(calc)	ppm	M	Peptide
113 - 127	1656.7831	1655.7758	1655.7800	-2.51	1	K.FVGGTKEFAEMGPMR.L
113 - 127	1672.7587	1671.7514	1671.7749	-14.0	1	K.FVGGTKEFAEMGPMR.L + Oxidation (M)
113 - 127	1688.7613	1687.7540	1687.7698	-9.36	1	K.FVGGTKEFAEMGPMR.L + 2 Oxidation (M)
236 - 243	958.5090	957.5017	957.5032	-1.56	0	K.AIQADVWR.S
311 - 318	924.3931	923.3858	923.3920	-6.63	0	K.CVDGGFNR.L
319 - 330	1370.7465	1369.7392	1369.6990	29.4	0	R.LTDAFIPHVSDR.L
369 - 383	1823.9360	1822.9287	1822.8270	55.8	0	K.DYDYTIMAVPFTMTR.F
369 - 383	1839.8647	1838.8574	1838.8219	19.3	0	K.DYDYTIMAVPFTMTR.F + Oxidation (M)
369 - 383	1855.9075	1854.9002	1854.8168	45.0	0	K.DYDYTIMAVPFTMTR.F + 2 Oxidation (M)
384 - 396	1411.7492	1410.7419	1410.7330	6.36	0	R.FMALPSFSSVLGR.A
384 - 396	1427.7323	1426.7250	1426.7279	-2.00	0	R.FMALPSFSSVLGR.A + Oxidation (M)
397 - 404	816.4371	815.4299	815.4501	-24.8	0	R.AISEAGLR.F
411 - 418	950.5340	949.5268	949.5233	3.69	0	K.VSLLFSER.F
462 - 468	792.3301	791.3228	791.3198	3.78	0	R.GGDWSDR.Y
469 - 494	2929.6827	2928.6754	2928.4254	85.4	0	R.YVSFSDEEHVQTVLDAIVSLHGEQAR.E
495 - 503	1145.5118	1144.5045	1144.5036	0.77	0	R.ELYTGDYER.L
562 - 577	1759.9513	1758.9440	1758.9437	0.18	0	R.GTIQLLLELGMVEEAK.E + Oxidation (M)

Spot No.: **40**

Mascot score: **128**

Species: ***Fusarium oxysporum f. sp. cubense race 4***

Protein name: **ATP synthase subunit beta, mitochondrial**

NCBI accession No.: **gi| 475664589**

Sequence coverage %: **64**

Matched peptides No.: **24**

Total peptides No.: **84**

Calculated Mr: **55197**

Calculated *p*I: **5.27**

Annotated PMF **spectra:**

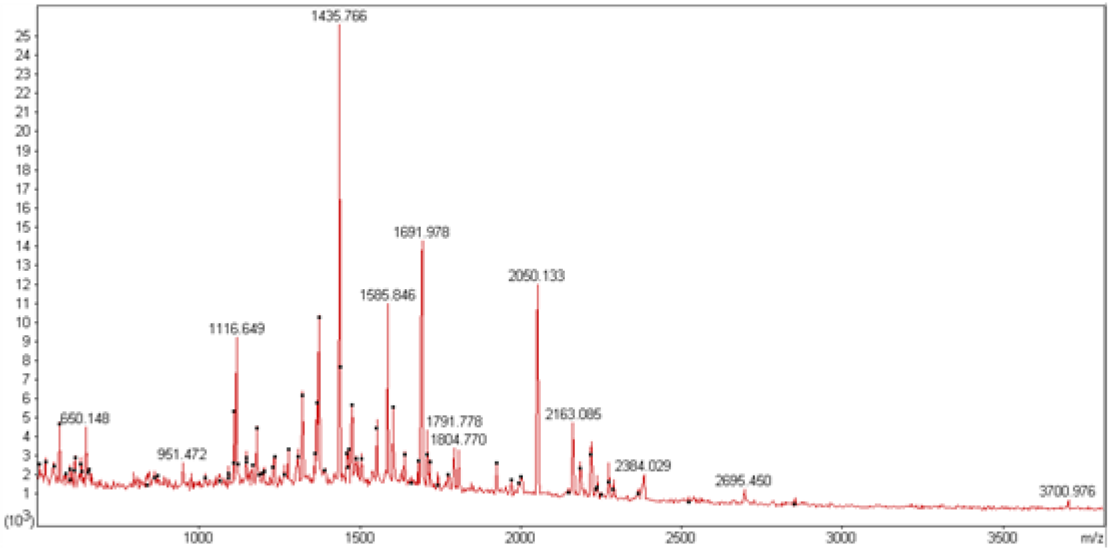

Probability Based Mowse Score:

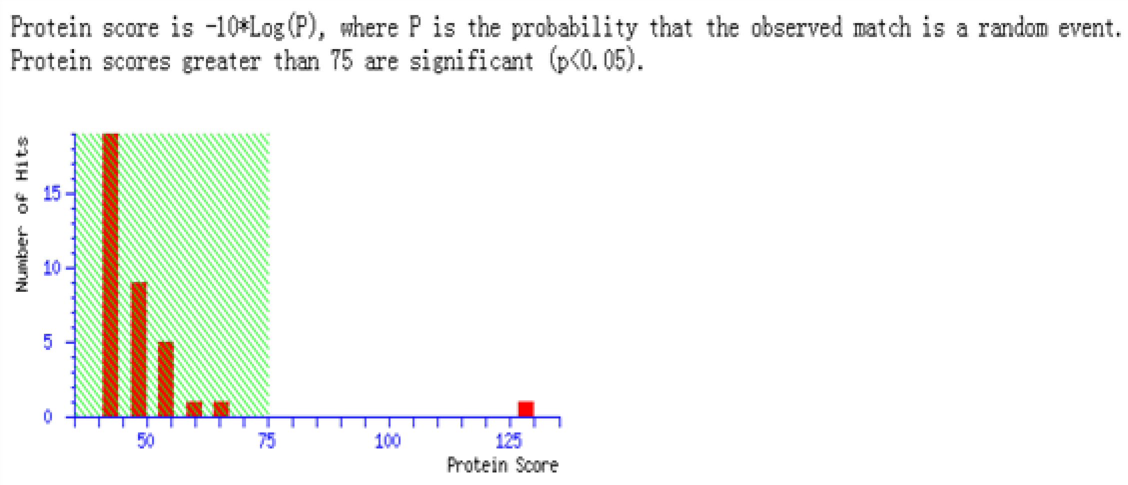

Matched peptide sequences: shown in Bold Red

1 MFKSGISSFA RAARPAVLPR RALRPSSLRL PISSRWASTA SVGTGKIHQV

51 IGAVVDVKFD TQKLPAILNS LETENNGQKL VLEVSQHLGE NVVRCIAMDG

101 TEGLVRGASA QDTGAPITIP VGPATLGRIM NVTGDPIDER GPIKTDKRLP

151 IHTEAPEFVE QSTSAEVLVT GIKVVDLLAP YARGGKIGLF GGAGVGKTVF

201 IQELINNIAK AHGGYSVFTG VGERTREGND LYHEMQETSV IQLDGESKVA

251 LVFGQMNEPP GARARVALTG LTVAEYFRDE EGQDVLLFID NIFRFTQAGS

301 EVSALLGRIP SAVGYQPTLA VDMGGMQERI TTTTKGSITS VQAVYVPADD

351 LTDPAPATTF AHLDATTVLS RGISELGIYP AVDPLDSKSR MLDPRVVGQE

401 HYDVATRVQQ ILQEYKSLQD IIAILGMDEL SEADKLTVER ARKIQRFLSQ

451 PFTVAQVFTG IEGKLVDLKE TINSFKAILN GEGDNLPEGA FYMVGDFASA

501 KAKGEKILAE LEGQ

Matched peptides information:

Start	-	End	Observed	Mr(expt)	Mr(calc)	ppm	M	Peptide
1	-	11	1230.6166	1229.6093	1229.6227	-10.8	1	-.MFKSGISSFAR.A
47	-	58	1277.7267	1276.7194	1276.7503	-24.2	0	K.IHQVIGAVVDVK.F
64	-	79	1740.9089	1739.9016	1739.9053	-2.13	0	K.LPAILNSLETENNGQK.L
80	-	94	1691.9779	1690.9706	1690.9366	20.1	0	K.LVLEVSQHLGENVVR.C
107	-	128	2050.1329	2049.1256	2049.0855	19.6	0	R.GASAQDTGAPITIPVGPATLGR.I
129	-	140	1359.6664	1358.6591	1358.6500	6.71	0	R.IMNVTGDPIDER.G
148	-	173	2851.6013	2850.5940	2850.5127	28.5	1	K.RLPIHTEAPEFVEQSTSAEVLVTGIK.V
149	-	173	2695.4498	2694.4425	2694.4116	11.5	0	R.LPIHTEAPEFVEQSTSAEVLVTGIK.V
174	-	183	1116.6491	1115.6418	1115.6339	7.12	0	K.VVDLLAPYAR.G
198	-	210	1502.8449	1501.8376	1501.8504	-8.51	0	K.TVFIQELINNIAK.A
227	-	248	2522.2189	2521.2116	2521.1278	33.2	0	R.EGNDLYHEMQETSVIQLDGESK.V
249	-	263	1585.8464	1584.8391	1584.8082	19.5	0	K.VALVFGQMNEPPGAR.A
249	-	263	1601.8247	1600.8174	1600.8032	8.91	0	K.VALVFGQMNEPPGAR.A + Oxidation (M)
266	-	278	1439.8319	1438.8246	1438.7820	29.6	0	R.VALTGLTVAEYFR.D
279	-	294	1922.9776	1921.9703	1921.9422	14.7	0	R.DEEGQDVLLFIDNIFR.F
295	-	308	1435.7662	1434.7589	1434.7467	8.53	0	R.FTQAGSEVSALLGR.I
309	-	329	2220.1354	2219.1281	2219.0715	25.5	0	R.IPSAVGYQPTLAVDMGGMQER.I
309	-	329	2236.1455	2235.1382	2235.0664	32.1	0	R.IPSAVGYQPTLAVDMGGMQER.I + Oxidation (M)
309	-	329	2252.0879	2251.0806	2251.0613	8.58	0	R.IPSAVGYQPTLAVDMGGMQER.I + 2 Oxidation (M)
336	-	371	3700.9765	3699.9692	3699.8632	28.7	0	K.GSITSVQAVYVPADDLTDPAPATTFAHLDATTVLSR.G
372	-	388	1773.9640	1772.9567	1772.9196	20.9	0	R.GISELGIYPAVDPLDSK.S
396	-	407	1373.7124	1372.7051	1372.6735	23.0	0	R.VVGQEHYDVATR.V
408	-	416	1148.6050	1147.5977	1147.6237	-22.6	0	R.VQQILQEYK.S
447	-	464	1969.0261	1968.0188	1968.0357	-8.57	0	R.FLSQPFTVAQVFTGIEGK.L

Spot No.: **41**

Mascot score: **88**

Species: ***Fusarium oxysporum Fo5176***

Protein name: **carboxy-cis,cis-muconate cyclase**

NCBI accession No.: **gi| 342888171**

Sequence coverage %: **43**

Matched peptides No.: **14**

Total peptides No.: **58**

Calculated Mr: **42345**

Calculated *p*I: **6.76**

Annotated PMF **spectra:**

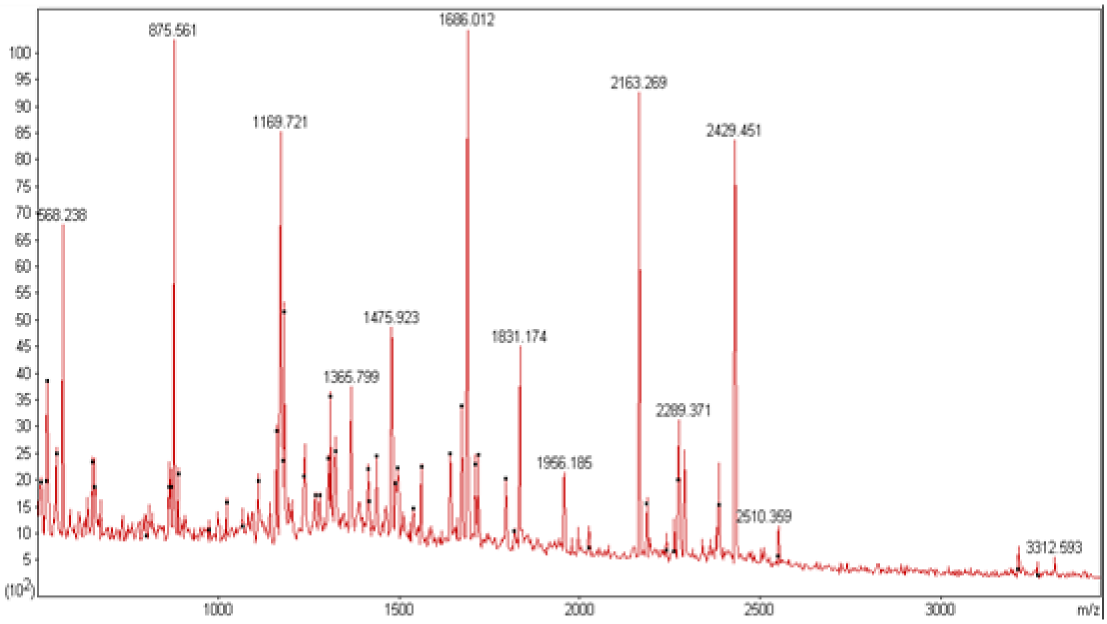

Probability Based Mowse Score:

Protein score is -10*Log(P), where P is the probability that the observed match is a random event.
Protein scores greater than 75 are significant (p<0.05).

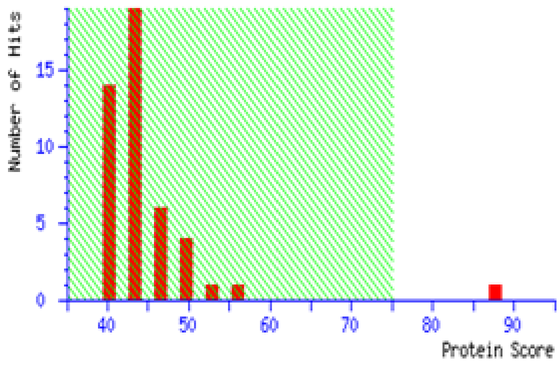

Matched peptide sequences: shown in **Bold Red**

1	MKYTIAGLLA	TLATSASALP	NIAR**SSYPTA**	**SASSSAAARI**	**LLGNSGHIYV**
51	**ADFSPK**TGKF	ELTLNQEIEG	GNSWMAYYDP	NLLYAVDENS	DELR**LFNLDL**
101	**EANK**LTLKTK	**KAGSVGVVHL**	**EFNSDKTR**LV	GAAYGNGTID	VWNTEKGGLE
151	FVKTLKSPGK	LGPDKER**QAA**	**SHPHQANLDP**	**SGRYFAVNDL**	**GTDSVVIIDS**
201	**KDDAYK**IAKN	**IPVEAGCGPR**	**HGVFYPR**GGK	KATHYIVACE	LSNQALVYSV
251	SYEENTLAFK	**HHQSISTYGK**	DAPAKDPK**TA**	**AVGEILLAPN**	**NKDVYISNRL**
301	SGNETDSIAR	**FTIAECGTLT**	**YADTVSSGGL**	**LPR**MMSFSLT	AKHVFVGNQN
351	GTSGLVALQR	GADGKLAEKP	VATLPGSAFG	EPLFGPQYVQ	QILLN

Matched peptides information:

Start	-	End	Observed	Mr(expt)	Mr(calc)	ppm	M	Peptide
25	-	39	1413.8326	1412.8253	1412.6532	122	0	R.SSYPTASASSSAAAR.I
40	-	56	1831.1738	1830.1665	1829.9676	109	0	R.ILLGNSGHIYVADFSPK.T
95	-	104	1176.7299	1175.7226	1175.6186	88.5	0	R.LFNLDLEANK.L
112	-	126	1558.9452	1557.9379	1557.7787	102	0	K.AGSVGVVHLEFNSDK.T
112	-	128	1816.1718	1815.1645	1814.9275	131	1	K.AGSVGVVHLEFNSDKTR.L
168	-	183	1686.0121	1685.0048	1684.8030	120	0	R.QAASHPHQANLDPSGR.Y
184	-	201	1956.1849	1955.1776	1954.9888	96.6	0	R.YFAVNDLGTDSVVIIDSK.D
184	-	206	2548.4749	2547.4676	2547.2381	90.1	1	R.YFAVNDLGTDSVVIIDSKDDAYK.I
210	-	220	1169.7207	1168.7134	1168.5659	126	0	K.NIPVEAGCGPR.H
221	-	227	875.5607	874.5534	874.4450	124	0	R.HGVFYPR.G
261	-	270	1157.6733	1156.6660	1156.5625	89.5	0	K.HHQSISTYGK.D
279	-	292	1410.8854	1409.8781	1409.7878	64.1	0	K.TAAVGEILLAPNNK.D
293	-	299	866.5311	865.5239	865.4294	109	0	K.DVYISNR.L
311	-	333	2429.4513	2428.4440	2428.1944	103	0	R.FTIAECGTLTYADTVSSGGLLPR.M

Spot No.: **42**

Mascot score: **80**

Species: ***Fusarium oxysporum f. sp. cubense race 4***

Protein name: **Alpha-galactosidase 2**

NCBI accession No.: **gi| 475672613** Sequence coverage %: **29**

Matched peptides No.: **18** Total peptides No.: **65**

Calculated Mr: **83037** Calculated *p*I: **5.01**

Annotated PMF **spectra:**

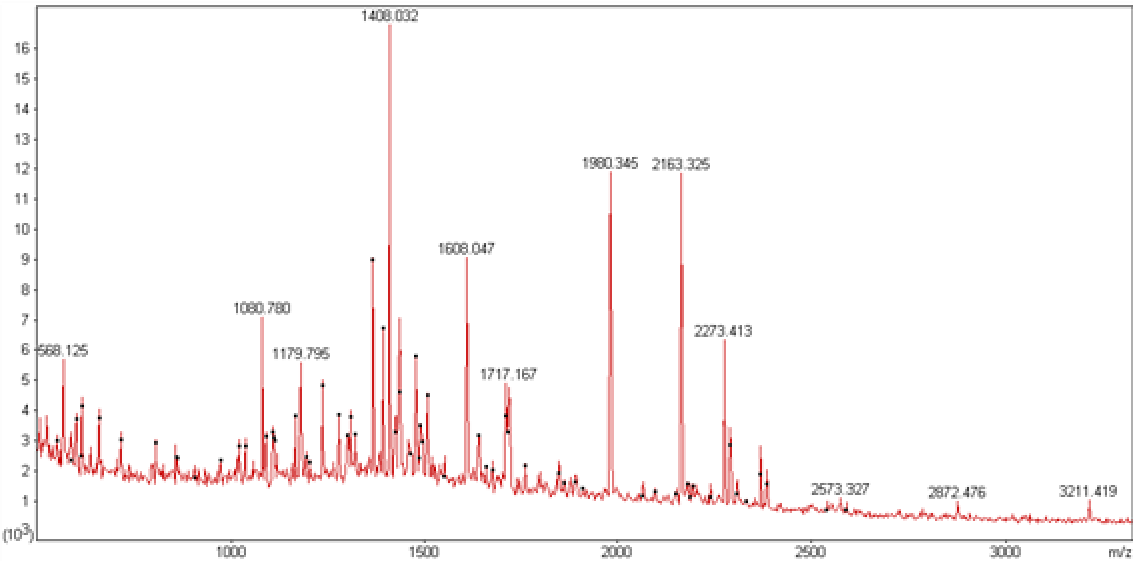

Probability Based Mowse Score:

Protein score is -10*Log(P), where P is the probability that the observed match is a random event.
Protein scores greater than 75 are significant (p<0.05).

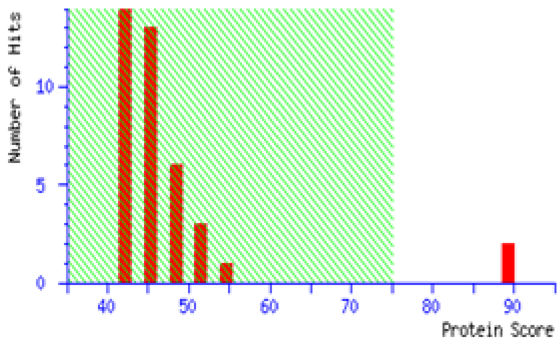

Matched peptide sequences: shown in Bold Red

1 MVLVTSKGIT TAAVLFSQVI SAFAETSDPI RVDGTSFALN GNNVSYRFHV
51 DNTTGDLIND HYGGPVAEDG ITTEIGPIQG WVNLIGRVRR **EFPDHGR**GDF
101 **RIPAFQLQQA SGTTVTDFRY** KSHEVVQGKP GLPGLPSTFG EADDVSTLVV
151 HMYDNYSSIA VDLSYSIFPE YDAIVRSVNI TNQGNATINL R**KVSSWSVDL**
201 **QQDNLDLIEI K**GDWAREGMR VRRKVDFGTQ GFQSSTGYSS HLHNPFLALV
251 SSTTTETQGE AWGFSLVYTG SFAVDVEKSS QGLTRAILGL NPLDFSWPLK
301 PEQTFTTPEV VSVFSNKGVG GMSRQFHRLY RKHLMKSKYA EETRPVLLNS
351 WEGLAFDINE TAIEKIAKQS ADLGIK**LFVM DDGWFGNKYP R**VNDTAGLGD
401 WQPDKSR**FPD GLTPLVENVT DLK**VANSSDE LKFGIWFEPE MVNPESDLYD
451 **KHPDWAIHAG SYPR**TETRNQ LVLNLALPEV QEFIIDFVSK VLR**ESPISYV**
501 **KWDNNRGIHE TPDPTLNYKY** MLGLYHVFET LTSRFPDVLW EGCASGGGRF
551 DPGVLQWFPQ IWTSDDTDAV ERIAIQFGTS LAYPPSAMGA HLSHVPNGNT
601 QRITSVKFR**A HVAMMGGSFG VELDPSDLEP EER**EQIPGLI ELSEK**INPIV**
651 **ITGDFYRLAL PEETNYPAGQ FISEDGKKVV LFAFQTRATI NNSWPWFRLQ**
701 **GLDASAKYK**V DNNQTVSGST LMNLGIQLR**F EGDYDSQVLM IEK**Q

Matched peptides information:

Start	-	End	Observed	Mr(expt)	Mr(calc)	ppm	M	Peptide
91	-	97	857.4496	856.4423	856.3828	69.5	0	R.EFPDHGR.G
102	-	119	1980.3447	1979.3374	1979.0113	165	0	R.IPAFQLQQASGTTVTDFR.Y
192	-	211	2330.4449	2329.4376	2329.2165	94.9	1	R.KVSSWSVDLQQDNLDLIEIK.G
377	-	391	1845.2127	1844.2054	1843.8716	181	1	K.LFVMDDGWFGNKYPR.V
377	-	391	1861.1643	1860.1570	1859.8665	156	1	K.LFVMDDGWFGNKYPR.V + Oxidation (M)
408	-	423	1758.1761	1757.1688	1756.9247	139	0	R.FPDGLTPLVENVTDLK.V
452	-	464	1506.9967	1505.9894	1505.7164	181	0	K.HPDWAIHAGSYPR.T
494	-	506	1608.0469	1607.0396	1606.7739	165	1	R.ESPISYVKWDNNR.G
507	-	519	1484.9839	1483.9766	1483.7307	166	0	R.GIHETPDPTLNYK.Y
610	-	633	2573.3272	2572.3199	2572.1574	63.2	0	R.AHVAMMGGSFGVELDPSDLEPEER.E
610	-	633	2589.3288	2588.3215	2588.1523	65.4	0	R.AHVAMMGGSFGVELDPSDLEPEER.E + Oxidation (M)
646	-	657	1408.0320	1407.0247	1406.7558	191	0	K.INPIVITGDFYR.L
658	-	677	2179.3048	2178.2975	2178.0480	115	0	R.LALPEETNYPAGQFISEDGK.K
658	-	678	2307.4160	2306.4087	2306.1430	115	1	R.LALPEETNYPAGQFISEDGKK.V
679	-	687	1080.7796	1079.7723	1079.6128	148	0	K.VVLFAFQTR.A
688	-	698	1391.9621	1390.9548	1390.6782	199	0	R.ATINNSWPWFR.L
699	-	709	1193.7878	1192.7805	1192.6452	114	1	R.LQGLDASAKYK.V
730	-	743	1674.0918	1673.0845	1672.7654	191	0	R.FEGDYDSQVLMIEK.Q

Spot No.: **43**

Mascot score: **113**

Species: ***Fusarium oxysporum f. sp. cubense race 4***

Protein name: **Alpha-galactosidase 2**

NCBI accession No.: **gi| 475672613**

Sequence coverage %: **36**

Matched peptides No.: **24**

Total peptides No.: **79**

Calculated Mr: **83037**

Calculated *p*I: **5.01**

Annotated PMF **spectra:**

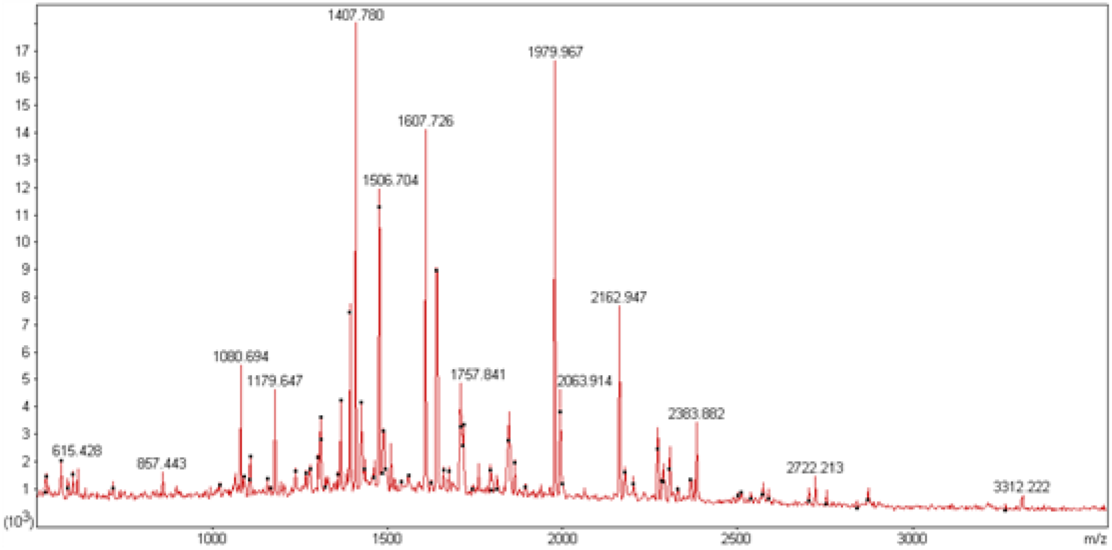

Probability Based Mowse Score:

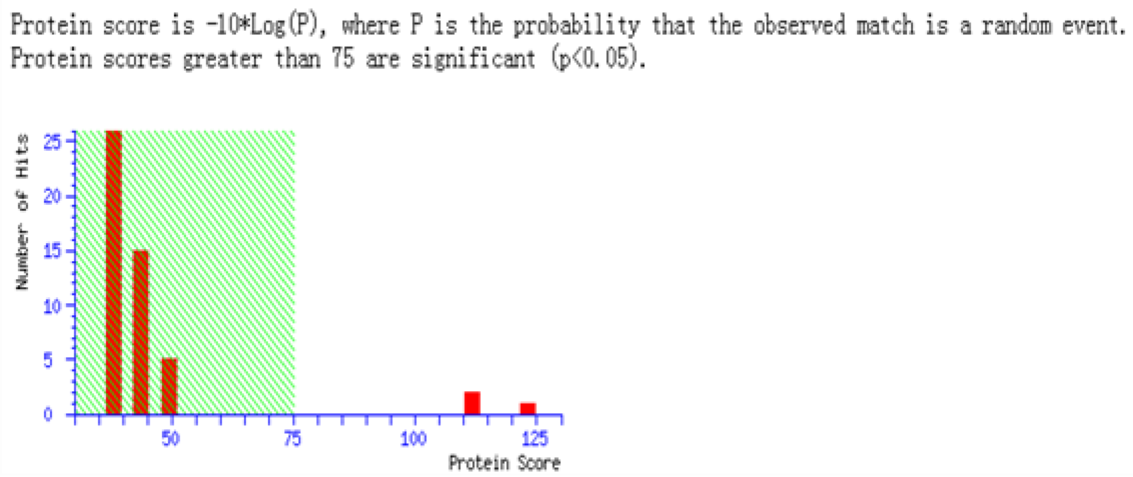

Matched peptide sequences: shown in **Bold Red**

1 MVLVTSKGIT TAAVLFSQVI SAFAETSDPI RVDGTSFALN GNNVSYRFHV

51 DNTTGDLIND HYGGPVAEDG ITTEIGPIQG WVNLIGRVRR EFPDHGRGDF

101 RIPAFQLQQA SGTTVTDFRY KSHEVVQGKP GLPGLPSTFG EADDVSTLVV

151 HMYDNYSSIA VDLSYSIFPE YDAIVRSVNI TNQGNATINL RKVSSWSVDL

201 QQDNLDLIEI KGDWAREGMR VRRKVDFGTQ GFQSSTGYSS HLHNPFLALV

251 SSTTTETQGE AWGFSLVYTG SFAVDVEKSS QGLTRAILGL NPLDFSWPLK

301 PEQTFTTPEV VSVFSNKGVG GMSRQFHRLY RKHLMKSKYA EETRPVLLNS

351 WEGLAFDINE TAIEKIAKQS ADLGIKLFVM DDGWFGNKYP RVNDTAGLGD

401 WQPDKSRFPD GLTPLVENVT DLKVANSSDE LKFGIWFEPE MVNPESDLYD

451 KHPDWAIHAG SYPRTETRNQ LVLNLALPEV QEFIIDFVSK VLRESPISYV

501 KWDNNRGIHE TPDPTLNYKY MLGLYHVFET LTSRFPDVLW EGCASGGGRF

551 DPGVLQWFPQ IWTSDDTDAV ERIAIQFGTS LAYPPSAMGA HLSHVPNGNT

601 QRITSVKFRA HVAMMGGSFG VELDPSDLEP EEREQIPGLI ELSEKINPIV

651 ITGDFYRLAL PEETNYPAGQ FISEDGKKVV LFAFQTRATI NNSWPWFRLQ

701 GLDASAKYKV DNNQTVSGST LMNLGIQLRF EGDYDSQVLM IEKQ

Matched peptides information:

Start - End	Observed	Mr(expt)	Mr(calc)	ppm	M	Peptide
91 - 97	857.4433	856.4360	856.3828	62.2	0	R.EFPDHGR.G
102 - 119	1979.9665	1978.9592	1979.0113	-26.3	0	R.IPAFQLQQASGTTVTDFR.Y
192 - 211	2330.1207	2329.1134	2329.2165	-44.3	1	R.KVSSWSVDLQQDNLDLIEIK.G
193 - 211	2201.9817	2200.9744	2201.1216	-66.8	0	K.VSSWSVDLQQDNLDLIEIK.G
325 - 328	587.2710	586.2638	586.2976	-57.7	0	R.QFHR.L
377 - 391	1844.8396	1843.8323	1843.8716	-21.3	1	K.LFVMDDGWFGNKYPR.V
377 - 391	1860.8034	1859.7961	1859.8665	-37.8	1	K.LFVMDDGWFGNKYPR.V + Oxidation (M)
406 - 423	2000.9523	1999.9450	2000.0579	-56.4	1	K.SRFPDGLTPLVENVTDLK.V
408 - 423	1757.8410	1756.8337	1756.9247	-51.8	0	R.FPDGLTPLVENVTDLK.V
452 - 464	1506.7041	1505.6968	1505.7164	-13.0	0	K.HPDWAIHAGSYPR.T
452 - 468	1993.9295	1992.9222	1992.9554	-16.7	1	K.HPDWAIHAGSYPRTETR.N
507 - 519	1484.6899	1483.6826	1483.7307	-32.4	0	R.GIHETPDPTLNYK.Y
507 - 534	3312.2217	3311.2144	3310.6332	176	1	R.GIHETPDPTLNYKYMLGLYHVFETLTSR.F + Oxidation (M)
535 - 549	1607.7263	1606.7190	1606.7198	-0.51	0	R.FPDVLWEGCASGGGR.F
550 - 572	2722.2134	2721.2061	2721.2711	-23.9	0	R.FDPGVLQWFPQIWTSDDTDAVER.I
610 - 633	2573.1275	2572.1202	2572.1574	-14.4	0	R.AHVAMMGGSFGVELDPSDLEPEER.E
610 - 633	2589.0802	2588.0729	2588.1523	-30.7	0	R.AHVAMMGGSFGVELDPSDLEPEER.E + Oxidation (M)
634 - 645	1355.7315	1354.7242	1354.7344	-7.48	0	R.EQIPGLIELSEK.I
646 - 657	1407.7799	1406.7726	1406.7558	12.0	0	K.INPIVITGDFYR.L
658 - 677	2178.9372	2177.9299	2178.0480	-54.2	0	R.LALPEETNYPAGQFISEDGK.K
658 - 678	2307.0508	2306.0435	2306.1430	-43.1	1	R.LALPEETNYPAGQFISEDGKK.V
679 - 687	1080.6942	1079.6869	1079.6128	68.7	0	K.VVLFAFQTR.A
688 - 698	1391.7010	1390.6937	1390.6782	11.2	0	R.ATINNSWPWFR.L
730 - 743	1673.7111	1672.7038	1672.7654	-36.8	0	R.FEGDYDSQVLMIEK.Q

Spot No.: **44**

NCBI accession No.: **gi|342878413**

Plant species: **quinone oxidoreductase**

Protein name： ***hypothetical protein ARALYDRAFT_486548***

Mascot score: **163** Sequence coverage %: **18**

The number of matched peptides with p≤0.05: **5**

Calculated Mr: **21740** Calculated *Pi*: **5.54**

Annotated MS **spectra:**

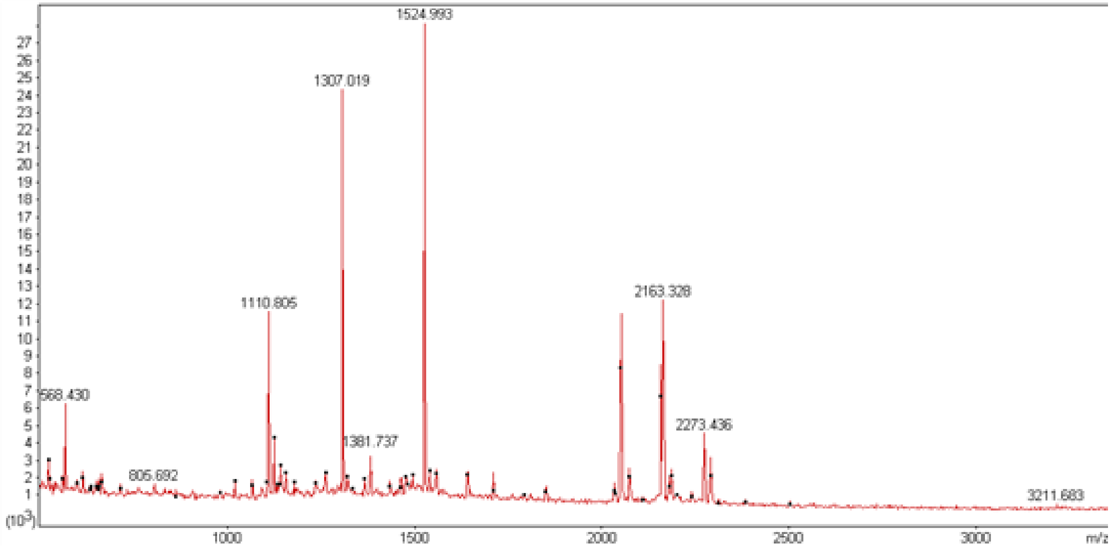

Probability Based Mowse Score:

Ions score is -10*Log(P), where P is the probability that the observed match is a random event.
Individual ions scores > 48 indicate identity or extensive homology (p<0.05).
Protein scores are derived from ions scores as a non-probabilistic basis for ranking protein hits.

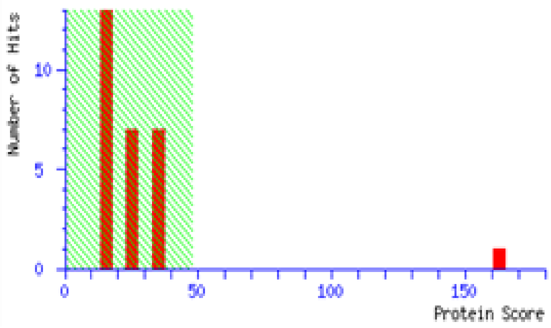

Matched peptide sequences: shown in Bold Red

```
  1 MAPKIAIVYY SMYGHIKQLA EAEKAGIEKA GGSADIFQVP ETLPEDVLAK
 51 MHAPPKPTDV PTLDDPSVLE GYDAFLLGIP TRYGNFPAQW KAFWDKTGKQ
101 WASGGFWGKM AGIFVSTASQ GGGQETTAQN AISTLTHHGI IYVPFGYAKA
151 FGVLTDLSEV RGGSAWGAGT FAGADGSRQP SAKELELAQI QGENFYQTVA
201 KFTG
```

Matched peptides information:

Query	Start - End	Observed	Mr(expt)	Mr(calc)	ppm	M	Score	Expect	Rank	U	Peptide
1	83 - 91	1110.8051	1109.7978	1109.5294	242	0	27	8.4	1	U	R.YGNFPAQWK.A
2	83 - 91	1110.8051	1109.7978	1109.5294	242	0	27	8.4	1	U	R.YGNFPAQWK.A
3	150 - 161	1307.0190	1306.0117	1305.6929	244	0	55	0.0087	1	U	K.AFGVLTDLSEVR.G
4	150 - 161	1307.0190	1306.0117	1305.6929	244	0	55	0.0087	1	U	K.AFGVLTDLSEVR.G
5	162 - 178	1524.9930	1523.9857	1523.6753	204	0	81	2.9e-05	1	U	R.GGSAWGAGTFAGADGSR.Q

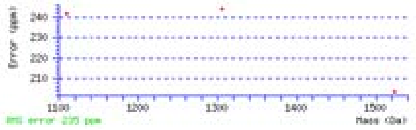

Annotated ion spectra of the matched peptides with p≤0.05:

CID No.: **44-1110.8**

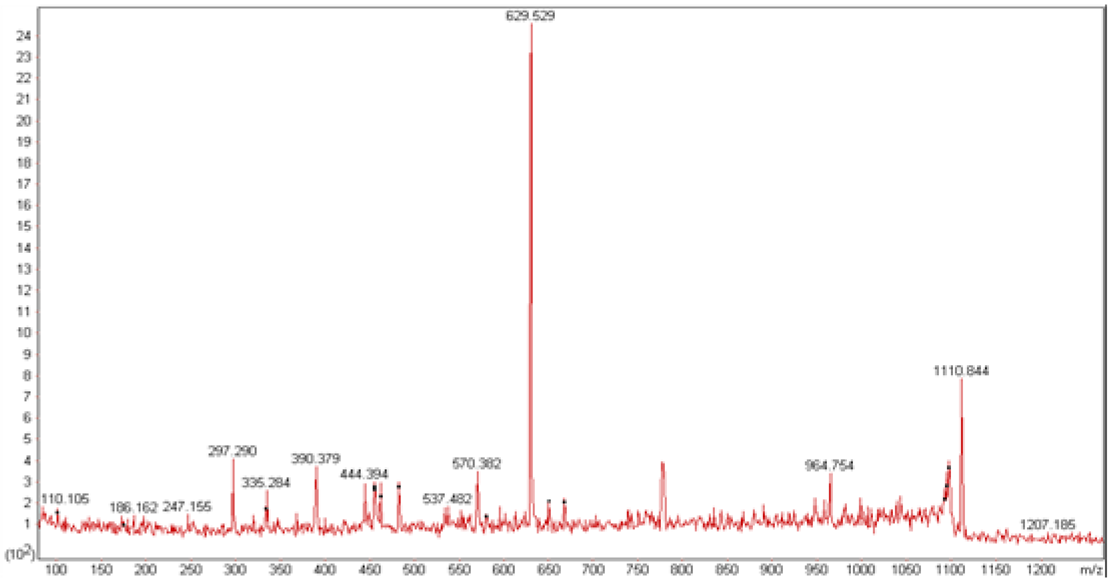

MS/MS Fragmentation of **R.YGNFPAQWK.A**

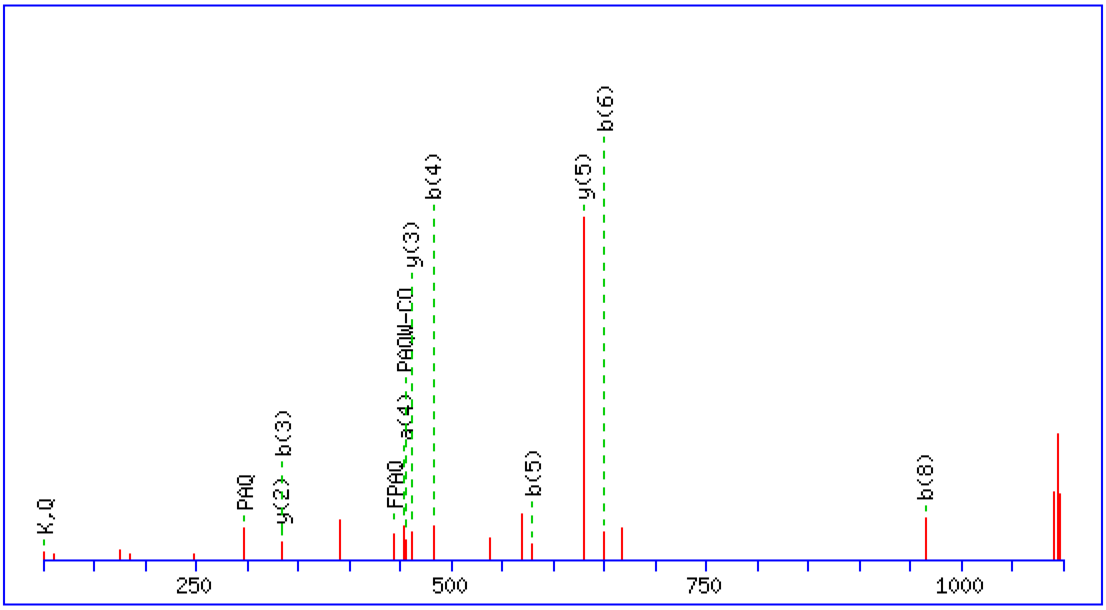

Monoisotopic mass of neutral peptide Mr(calc): 1109.5294

#	Immon.	a	a*	b	b*	d	Seq.	v	w	y	y*	#
1	136.0757	136.0757		164.0706		44.0495	Y					9
2	30.0338	193.0972		221.0921			G			947.4734	930.4468	8
3	87.0553	307.1401	290.1135	***335.1350***	318.1084	264.1343	N	831.4148	830.4196	890.4519	873.4254	7
4	120.0808	454.2085	437.1819	***482.2034***	465.1769		F	684.3464		776.4090	759.3824	6
5	70.0651	551.2613	534.2347	***579.2562***	562.2296	525.2456	P	587.2936	586.2984	**629.3406**	612.3140	5
6	44.0495	622.2984	605.2718	***650.2933***	633.2667		A	516.2565		532.2878	515.2613	4
7	101.0709	750.3570	733.3304	778.3519	761.3253	693.3355	Q	388.1979	387.2027	**461.2507**	444.2241	3
8	159.0917	936.4363	919.4097	***964.4312***	947.4046		W	202.1186		**333.1921**	316.1656	2
9	101.1073						K	74.0237	73.0284	147.1128	130.0863	1

Seq	ya	yb	Seq	ya	yb	Seq	ya	yb
GN	144.0768	172.0717	GNF	291.1452	319.1401	GNFP	388.1979	416.1928
GNFPA	459.2350	487.2300	GNFPAQ	587.2936	615.2885	NF	234.1237	262.1186
NFP	331.1765	359.1714	NFPA	402.2136	430.2085	NFPAQ	530.2722	558.2671
FP	217.1335	245.1285	FPA	288.1707	316.1656	FPAQ	416.2292	444.2241
FPAQW	602.3085	630.3035	PA	141.1022	169.0972	PAQ	269.1608	297.1557
PAQW	455.2401	483.2350	AQ	172.1081	200.1030	AQW	358.1874	386.1823
QW	287.1503	315.1452						

Annotated ion spectra of the matched peptides with p≤0.05:

CID No.: **44-1307.08**

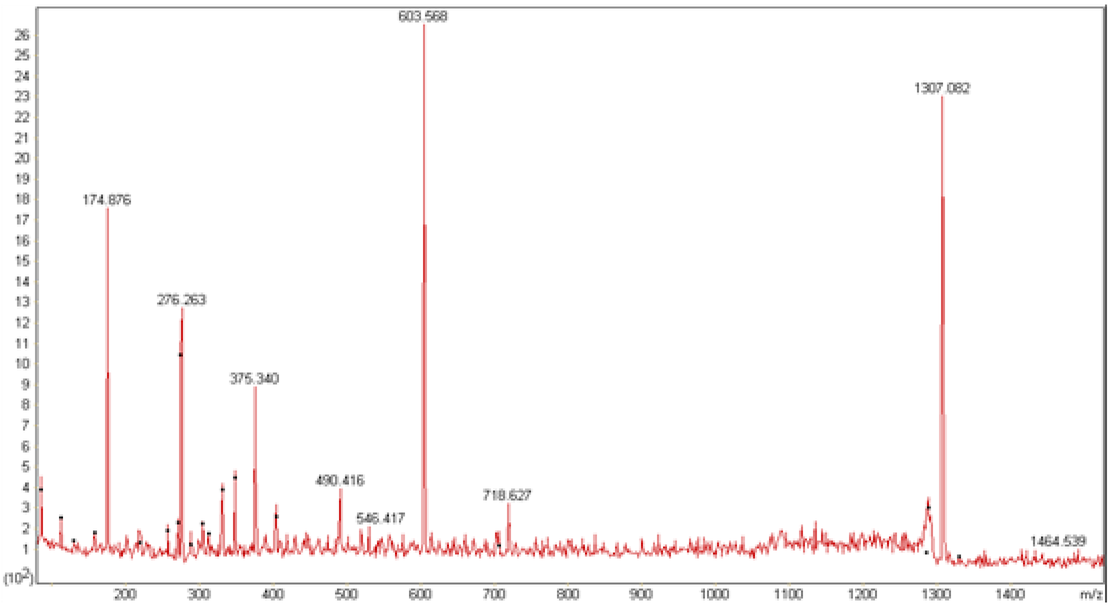

MS/MS Fragmentation of **K.AFGVLTDLSEVR.G**

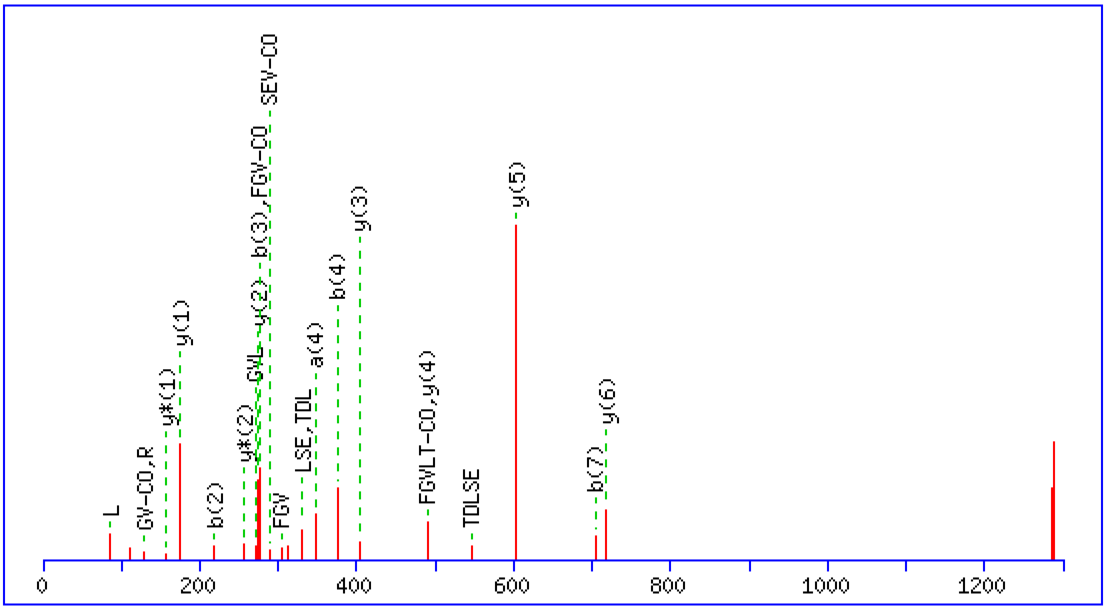

Monoisotopic mass of neutral peptide Mr(calc): 1305.6929

#	Immon.	a	a^0	b	b^0	d	d'	Seq.	v	w	w'	y	y*	y^0	#
1	44.0495	44.0495		72.0444		44.0495		A							12
2	120.0808	191.1179		*219.1128*				F	1143.6004			1235.6630	1218.6365	1217.6525	11
3	30.0338	248.1394		*276.1343*				G				1088.5946	1071.5681	1070.5840	10
4	72.0808	347.2078		*375.2027*		333.1921		V	987.5106	1000.5310		1031.5732	1014.5466	1013.5626	9
5	86.0964	460.2918		488.2867		418.2449		L	874.4265	873.4312		932.5047	915.4782	914.4942	8
6	74.0600	561.3395	543.3289	589.3344	571.3239	545.3446	547.3239	T	773.3788	786.3992	788.3785	819.4207	802.3941	801.4101	7
7	88.0393	676.3665	658.3559	*704.3614*	686.3508	632.3766		D	658.3519	657.3566		*718.3730*	701.3464	700.3624	6
8	86.0964	789.4505	771.4400	817.4454	799.4349	747.4036		L	545.2678	544.2726		*603.3461*	586.3195	585.3355	5
9	60.0444	876.4825	858.4720	904.4775	886.4669	860.4876		S	458.2358	457.2405		*490.2620*	473.2354	472.2514	4
10	102.0550	1005.5251	987.5146	1033.5201	1015.5095	947.5197		E	329.1932	328.1979		*403.2300*	386.2034	385.2194	3
11	72.0808	1104.5936	1086.5830	1132.5885	1114.5779	1090.5779		V	230.1248	243.1452		*274.1874*	257.1608		2
12	129.1135							R	74.0237	73.0284		*175.1190*	158.0924		1

Seq	ya	yb	Seq	ya	yb	Seq	ya	yb
**FG**	177.1022	205.0972	**FGV**	276.1707	304.1656	**FGVL**	389.2547	417.2496
**FGVLT**	490.3024	518.2973	**FGVLTD**	605.3293	633.3243	**GV**	129.1022	157.0972
**GVL**	242.1863	270.1812	**GVLT**	343.2340	371.2289	**GVLTD**	458.2609	486.2558
**GVLTDL**	571.3450	599.3399	**GVLTDLS**	658.3770	686.3719	**VL**	185.1648	213.1598
**VLT**	286.2125	314.2074	**VLTD**	401.2395	429.2344	**VLTDL**	514.3235	542.3184
**VLTDLS**	601.3556	629.3505	**LT**	187.1441	215.1390	**LTD**	302.1710	330.1660
**LTDL**	415.2551	443.2500	**LTDLS**	502.2871	530.2821	**LTDLSE**	631.3297	659.3246
**TD**	189.0870	217.0819	**TDL**	302.1710	330.1660	**TDLS**	389.2031	417.1980
**TDLSE**	518.2457	546.2406	**TDLSEV**	617.3141	645.3090	**DL**	201.1234	229.1183
**DLS**	288.1554	316.1503	**DLSE**	417.1980	445.1929	**DLSEV**	516.2664	544.2613
**LS**	173.1285	201.1234	**LSE**	302.1710	330.1660	**LSEV**	401.2395	429.2344
**SE**	189.0870	217.0819	**SEV**	288.1554	316.1503	**EV**	201.1234	229.1183

Annotated ion spectra of the matched peptides with p≤0.05:

CID No.: **44-1525.02**

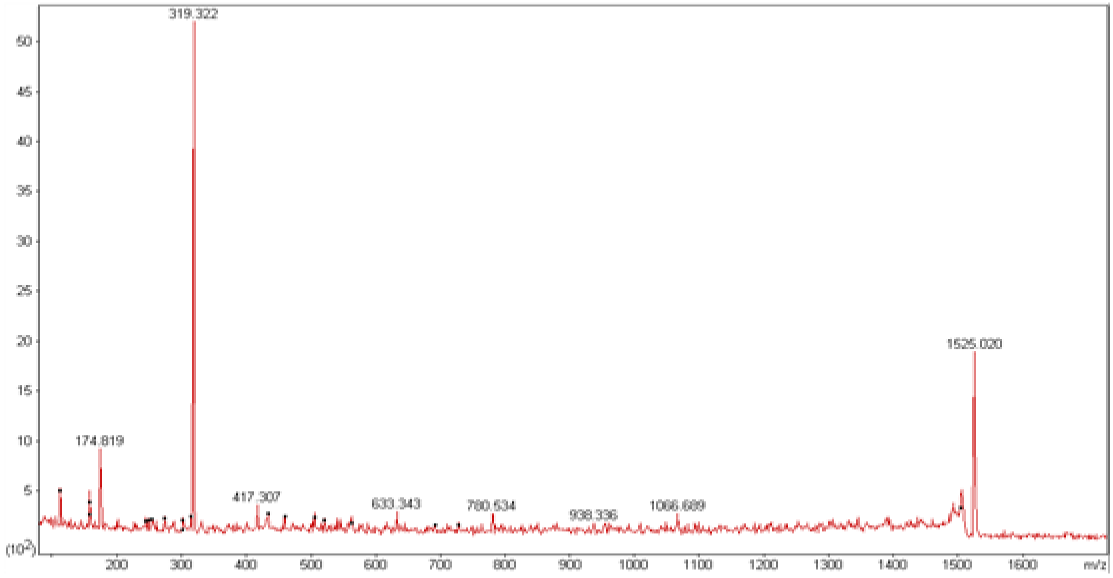

MS/MS Fragmentation of **R.GGSAWGAGTFAGADGSR.Q**

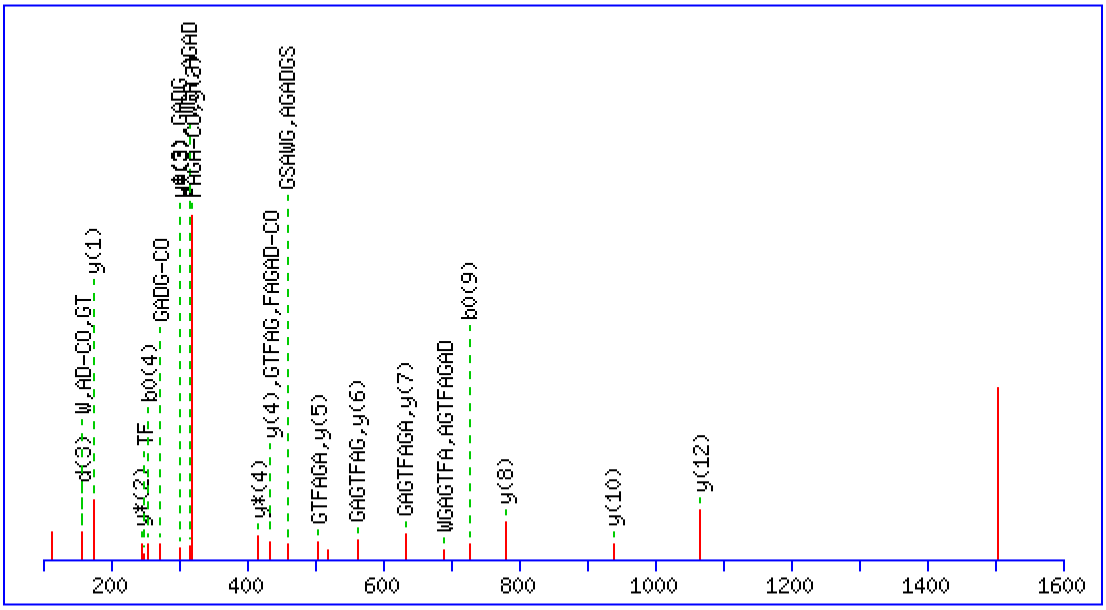

Monoisotopic mass of neutral peptide Mr(calc): 1523.6753

#	Immon.	a	a^0	b	b^0	d	d'	Seq.	v	w	w'	y	y*	y^0	#
1	30.0338	30.0338		58.0287		44.0495		G							17
2	30.0338	87.0553		115.0502				G				1467.6611	1450.6346	1449.6506	16
3	60.0444	174.0873	156.0768	202.0822	184.0717	158.0924		S	1378.6135	1377.6182		1410.6397	1393.6131	1392.6291	15
4	44.0495	245.1244	227.1139	**273.1193**	255.1088			A	1307.5763			1323.6076	1306.5811	1305.5971	14
5	159.0917	431.2037	413.1932	**459.1987**	441.1881			W	1121.4970			1252.5705	1235.5440	1234.5600	13
6	30.0338	488.2252	470.2146	516.2201	498.2096			G				***1066.4912***	1049.4647	1048.4806	12
7	44.0495	559.2623	541.2518	587.2572	569.2467			A	993.4385			1009.4698	992.4432	991.4592	11
8	30.0338	616.2838	598.2732	644.2787	626.2681			G				***938.4326***	921.4061	920.4221	10
9	74.0600	717.3315	699.3209	745.3264	727.3158	701.3365	703.3158	T	835.3693	848.3897	850.3690	881.4112	864.3846	863.4006	9
10	120.0808	864.3999	846.3893	892.3948	874.3842			F	688.3009			***780.3635***	763.3369	762.3529	8
11	44.0495	935.4370	917.4264	963.4319	945.4213			A	617.2638			***633.2951***	616.2685	615.2845	7
12	30.0338	992.4585	974.4479	1020.4534	1002.4428			G				***562.2580***	545.2314	544.2474	6
13	44.0495	1063.4956	1045.4850	1091.4905	1073.4799			A	489.2052			***505.2365***	488.2100	487.2259	5
14	88.0393	1178.5225	1160.5119	1206.5174	1188.5069	1134.5327		D	374.1783	373.1830		***434.1994***	417.1728	416.1888	4
15	30.0338	1235.5440	1217.5334	1263.5389	1245.5283			G				***319.1724***	302.1459	301.1619	3
16	60.0444	1322.5760	1304.5654	1350.5709	1332.5604	1306.5811		S	230.1248	229.1295		262.1510	245.1244	244.1404	2
17	129.1135							R	74.0237	73.0284		***175.1190***	158.0924		1

Seq	ya	yb	Seq	ya	yb	Seq	ya	yb
GS	117.0659	145.0608	GSA	188.1030	216.0979	GSAW	374.1823	402.1772
GSAWG	431.2037	459.1987	GSAWGA	502.2409	530.2358	GSAWGAG	559.2623	587.2572
GSAWGAGT	660.3100	688.3049	SA	131.0815	159.0764	SAW	317.1608	345.1557
SAWG	374.1823	402.1772	SAWGA	445.2194	473.2143	SAWGAG	502.2409	530.2358
SAWGAGT	603.2885	631.2835	AW	230.1288	258.1237	AWG	287.1503	315.1452
AWGA	358.1874	386.1823	AWGAG	415.2088	443.2037	AWGAGT	516.2565	544.2514
AWGAGTF	663.3249	691.3198	WG	216.1131	244.1081	WGA	287.1503	315.1452
WGAG	344.1717	372.1666	WGAGT	445.2194	473.2143	WGAGTF	592.2878	620.2827
WGAGTFA	663.3249	691.3198	GA	101.0709	129.0659	GAG	158.0924	186.0873
GAGT	259.1401	287.1350	GAGTF	406.2085	434.2034	GAGTFA	477.2456	505.2405
GAGTFAG	534.2671	562.2620	GAGTFAGA	605.3042	633.2991	AG	101.0709	129.0659
AGT	202.1186	230.1135	AGTF	349.1870	377.1819	AGTFA	420.2241	448.2191
AGTFAG	477.2456	505.2405	AGTFAGA	548.2827	576.2776	AGTFAGAD	663.3097	691.3046
GT	131.0815	159.0764	GTF	278.1499	306.1448	GTFA	349.1870	377.1819
GTFAG	406.2085	434.2034	GTFAGA	477.2456	505.2405	GTFAGAD	592.2726	620.2675
GTFAGADG	649.2940	677.2889	TF	221.1285	249.1234	TFA	292.1656	320.1605
TFAG	349.1870	377.1819	TFAGA	420.2241	448.2191	TFAGAD	535.2511	563.2460
TFAGADG	592.2726	620.2675	TFAGADGS	679.3046	707.2995	FA	191.1179	219.1128
FAG	248.1394	276.1343	FAGA	319.1765	347.1714	FAGAD	434.2034	462.1983
FAGADG	491.2249	519.2198	FAGADGS	578.2569	606.2518	AG	101.0709	129.0659
AGA	172.1081	200.1030	AGAD	287.1350	315.1299	AGADG	344.1565	372.1514
AGADGS	431.1885	459.1834	GA	101.0709	129.0659	GAD	216.0979	244.0928
GADG	273.1193	301.1143	GADGS	360.1514	388.1463	AD	159.0764	187.0713
ADG	216.0979	244.0928	ADGS	303.1299	331.1248	DG	145.0608	173.0557
DGS	232.0928	260.0877	GS	117.0659	145.0608			

Spot No.: **45**

Mascot score: **148**

Species: ***Fusarium oxysporum Fo5176***

Protein name: **protein disulfide-isomerase tigA precursor**

NCBI accession No.: **gi| 342876003** Sequence coverage %: **60**

Matched peptides No.: **21** Total peptides No.: **81**

Calculated Mr: **44513** Calculated *p*I: **5.77**

Annotated PMF **spectra:**

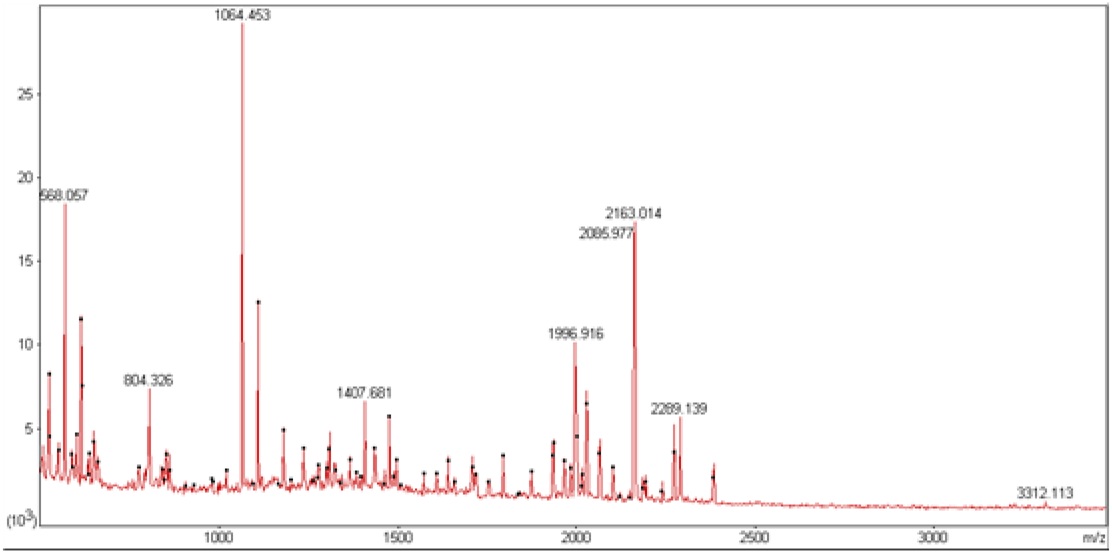

Probability Based Mowse Score:

Protein score is -10*Log(P), where P is the probability that the observed match is a random event. Protein scores greater than 75 are significant (p<0.05).

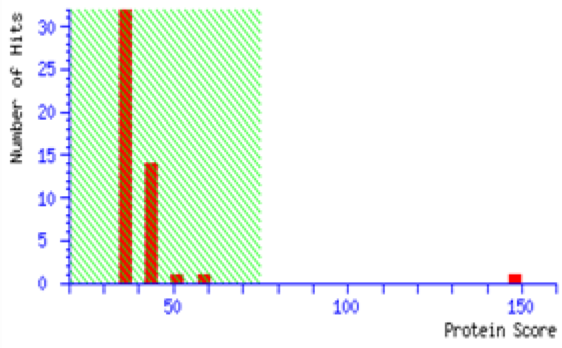

Matched peptide sequences: shown in **Bold Red**

```
  1 MVLIKSFVLS ALAATVAAKS AVIELLPSNF DDIVLKSGKP TLVEFFAPWC
 51 GHCKTLAPVW EDLANTYEYA KDKVQIAKVD ADAQRELGKR FGIQGFPTLK
101 FFDGKSSKPQ DYKSGRDLES LTNFIVEKTG VKPKKKLELP SEVTYLNDAT
151 FPKAIGGDKH VLVAFTAPWC GHCKSLAPTW EDLANTFVNE KNVLIAKVDA
201 EAPNSKAVAE EQGVKSYPTI KWFPAGSKKA VAYESGRSEQ AFVDWINEHA
251 GTHRVTGGGL DTVAGTVESL DTLVAKITGG AAIADVAEEV KKEVETLTDS
301 AQKTYAEYYV RVFDKLSSNN DWVSKELARL DGILTKGGLA PAKRDQIQQK
351 TNVLRKFTQK VEEKVEEIKD EL
```

Matched peptides information:

Start	-	End	Observed	Mr(expt)	Mr(calc)	ppm	M	Peptide
20	-	36	1872.9733	1871.9660	1872.0244	-31.2	0	K.SAVIELLPSNFDDIVLK.S
37	-	54	2120.9629	2119.9556	2119.9972	-19.6	0	K.SGKPTLVEFFAPWCGHCK.T
55	-	71	1983.8875	1982.8802	1982.9625	-41.5	0	K.TLAPVWEDLANTYEYAK.D
79	-	85	774.3139	773.3066	773.3668	-77.8	0	K.VDADAQR.E
79	-	89	1201.5342	1200.5269	1200.6098	-69.1	1	K.VDADAQRELGK.R
90	-	100	1263.6246	1262.6173	1262.7135	-76.2	1	K.RFGIQGFPTLK.F
91	-	100	1107.5455	1106.5382	1106.6124	-67.1	0	R.FGIQGFPTLK.F
101	-	105	613.2265	612.2192	612.2908	-117	0	K.FFDGK.S
114	-	128	1707.7616	1706.7543	1706.8839	-75.9	1	K.SGRDLESLTNFIVEK.T
117	-	128	1407.6812	1406.6739	1406.7293	-39.4	0	R.DLESLTNFIVEK.T
136	-	153	2065.0480	2064.0407	2064.0779	-18.0	1	K.KLELPSEVTYLNDATFPK.A
137	-	153	1936.9603	1935.9530	1935.9829	-15.5	0	K.LELPSEVTYLNDATFPK.A
175	-	191	1934.8967	1933.8894	1933.9421	-27.3	0	K.SLAPTWEDLANTFVNEK.N
198	-	206	930.3850	929.3778	929.4454	-72.8	0	K.VDAEAPNSK.A
229	-	237	980.4152	979.4079	979.5087	-103	1	K.KAVAYESGR.S
230	-	237	852.3518	851.3445	851.4137	-81.3	0	K.AVAYESGR.S
238	-	254	1996.9164	1995.9091	1995.9187	-4.81	0	R.SEQAFVDWINEHAGTHR.V
255	-	276	2103.0571	2102.0498	2102.1107	-29.0	0	R.VTGGGLDTVAGTVESLDTLVAK.I
277	-	292	1571.8090	1570.8017	1570.8566	-34.9	1	K.ITGGAAIADVAEEVKK.E
304	-	311	1064.4527	1063.4454	1063.4974	-48.9	0	K.TYAEYYVR.V
312	-	325	1638.8045	1637.7972	1637.8049	-4.71	1	R.VFDKLSSNNDWVSK.E

Spot No.: **46**

Mascot score: **168**

Species: ***Fusarium oxysporum f. sp. cubense race 4***

Protein name: **ATP synthase subunit beta, mitochondrial**

NCBI accession No.: **gi| 475664589** Sequence coverage %: **68**

Matched peptides No.: **28** Total peptides No.: **71**

Calculated Mr: **55197** Calculated *p*I: **5.27**

Annotated PMF **spectra:**

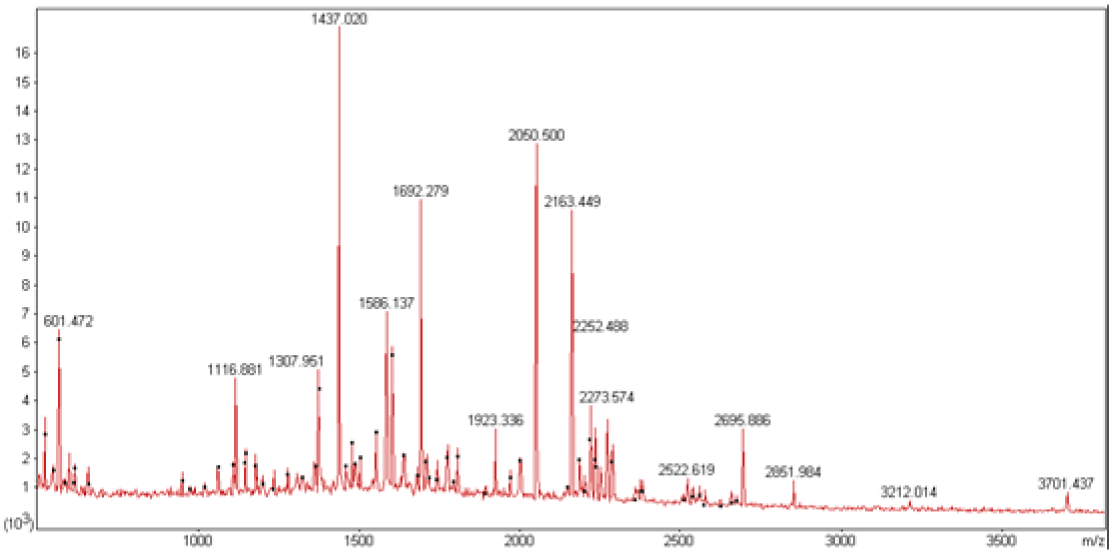

Probability Based Mowse Score:

Protein score is -10*Log(P), where P is the probability that the observed match is a random event.
Protein scores greater than 75 are significant (p<0.05).

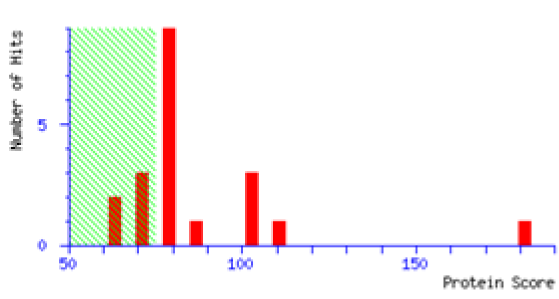

Matched peptide sequences: shown in **Bold Red**

1 MFKSGISSFA RAARPAVLPR RALRPSSLRL PISSRWASTA SVGTGK**IHQV**
51 **IGAVVDVKFD TQKLPAILNS LETENNGQKL VLEVSQHLGE NVVRCIAMDG**
101 **TEGLVRGASA QDTGAPITIP VGPATLGRIM** NVTGDPIDER GPIKTDK**RLP**
151 **IHTEAPEFVE QSTSAEVLVT GIKVVDLLAP YARGGKIGLF GGAGVGKTVF**
201 **IQELINNIAK AHGGYSVFTG VGERTREGND LYHEMQETSV IQLDGESKVA**
251 **LVFGQMNEPP GAR**ARVALTG LTVAEYFR**DE EGQDVLLFID NIFR**FTQAGS
301 EVSALLGR**IP SAVGYQPTLA VDMGGMQERI** TTTTKGSITS **VQAVYVPADD**
351 **LTDPAPATTF AHLDATTVLS RGISELGIYP AVDPLDSK**SR MLDPR**VVGQE**
401 **HYDVATRVQQ ILQEYKSLQD IIAILGMDEL SEADKLTVER** ARKIQR**FLSQ**
451 **PFTVAQVFTG IEGKLVDLK**E TINSFKAILN GEGDNLPEGA FYMVGDFASA
501 KAKGEKILAE LEGQ

Matched peptides information:

Start	-	End	Observed	Mr(expt)	Mr(calc)	ppm	M	Peptide
47	-	58	1277.9769	1276.9696	1276.7503	172	0	K.IHQVIGAVVDVK.F
59	-	79	2360.6399	2359.6326	2359.2019	183	1	K.FDTQKLPAILNSLETENNGQK.L
64	-	79	1741.1681	1740.1608	1739.9053	147	0	K.LPAILNSLETENNGQK.L
80	-	94	1692.2787	1691.2714	1690.9368	198	0	K.LVLEVSQHLGENVVR.C
95	-	106	1321.9203	1320.9130	1320.6166	224	0	R.CIAMDGTEGLVR.G
107	-	128	2050.5003	2049.4930	2049.0855	199	0	R.GASAQDTGAPITIPVGPATLGR.I
148	-	173	2851.9845	2850.9772	2850.5127	163	1	K.RLPIHTEAPEFVEQSTSAEVLVTGIK.V
149	-	173	2695.8861	2694.8788	2694.4116	173	0	R.LPIHTEAPEFVEQSTSAEVLVTGIK.V
174	-	183	1116.8809	1115.8736	1115.6339	215	0	K.VVDLLAPYAR.G
187	-	197	975.7881	974.7808	974.5549	232	0	K.IGLFGGAGVGK.T
198	-	210	1503.0998	1502.0925	1501.8504	161	0	K.TVFIQELINNIAK.A
211	-	224	1437.0200	1436.0127	1435.6844	229	0	K.AHGGYSVFTGVGER.T
227	-	248	2522.6193	2521.6120	2521.1278	192	0	R.EGNDLYHEMQETSVIQLDGESK.V
227	-	248	2538.6523	2537.6450	2537.1228	206	0	R.EGNDLYHEMQETSVIQLDGESK.V + Oxidation (M)
249	-	263	1586.1365	1585.1292	1584.8082	203	0	K.VALVFGQMNEPPGAR.A
249	-	263	1602.0982	1601.0909	1600.8032	180	0	K.VALVFGQMNEPPGAR.A + Oxidation (M)
279	-	294	1923.3358	1922.3285	1921.9422	201	0	R.DEEGQDVLLFIDNIFR.F
309	-	329	2220.4822	2219.4749	2219.0715	182	0	R.IPSAVGYQPTLAVDMGGMQER.I
309	-	329	2236.4704	2235.4631	2235.0664	178	0	R.IPSAVGYQPTLAVDMGGMQER.I + Oxidation (M)
309	-	329	2252.4883	2251.4810	2251.0613	166	0	R.IPSAVGYQPTLAVDMGGMQER.I + 2 Oxidation (M)
336	-	371	3701.4373	3700.4300	3699.8632	153	0	K.GSITSVQAVYVPADDLTDPAPATTFAHLDATTVLSR.G
372	-	388	1774.2213	1773.2140	1772.9198	166	0	R.GISELGIYPAVDPLDSK.S
396	-	407	1373.9492	1372.9419	1372.6735	196	0	R.VVGQEHYDVATR.V
408	-	416	1148.8514	1147.8441	1147.6237	192	0	R.VQQILQEYK.S
417	-	440	2659.8357	2658.8284	2658.3788	169	1	K.SLQDIIAILGMDELSEADKLTVER.A
417	-	440	2675.8070	2674.7997	2674.3735	159	1	K.SLQDIIAILGMDELSEADKLTVER.A + Oxidation (M)
447	-	464	1969.3905	1968.3832	1968.0357	177	0	R.FLSQPFTVAQVFTGIEGK.L
465	-	469	587.3447	586.3374	586.3690	-53.9	0	K.LVDLK.E

Spot No.: **47**

Mascot score: **81**

Species: ***Laccaria bicolor S238N-H82***

Protein name: **ubiquitin**

NCBI accession No.: **gi| 170098594**

Sequence coverage %: **84**

Matched peptides No.: **7**

Total peptides No.: **61**

Calculated Mr: **8152**

Calculated p*I*: **5.71**

Annotated PMF **spectra:**

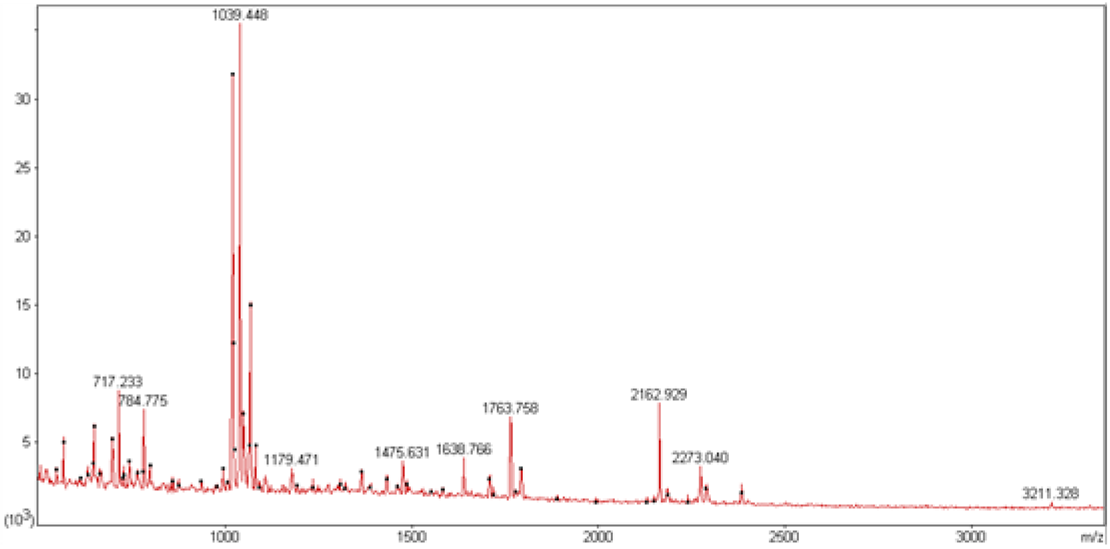

Probability Based Mowse Score:

Protein score is -10*Log(P), where P is the probability that the observed match is a random event.
Protein scores greater than 75 are significant (p<0.05).

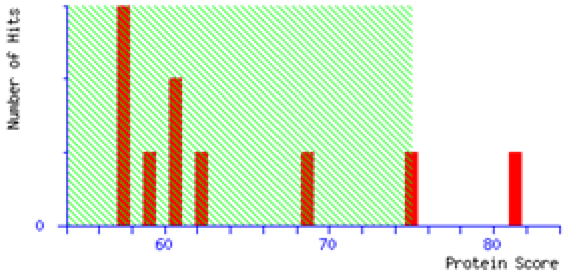

Matched peptide sequences: shown in Bold Red

```
 1 MQIFVKTLTG KTITLEVESS DTIDNVKAKI QDKEGIPPDQ QRLIFAGKQL
51 EDGRTLSDYN IQKESTLHLV LR
```

Matched peptides information:

Start	–	End	Observed	Mr(expt)	Mr(calc)	ppm	M	Peptide
1	–	6	765.3025	764.2952	764.4255	-170	0	-.MQIFVK.T
12	–	27	1763.7576	1762.7503	1762.8836	-75.6	0	K.TITLEVESSDTIDNVK.A
34	–	42	1039.4483	1038.4410	1038.5094	-65.9	0	K.EGIPPDQQR.L
43	–	48	648.2649	647.2576	647.4006	-221	0	R.LIFAGK.Q
49	–	54	717.2329	716.2256	716.3453	-167	0	K.QLEDGR.T
55	–	63	1081.4277	1080.4204	1080.5451	-115	0	R.TLSDYNIQK.E
64	–	72	1067.5175	1066.5102	1066.6135	-96.8	0	K.ESTLHLVLR.-

Spot No.: **48**

NCBI accession No.: **gi|342873137**

Plant species: ***Verticillium dahliae VdLs.17***

Protein name： **nuclear transport factor 2**

Mascot score: **166** Sequence coverage %: **12**

The number of matched peptides with p≤0.05: **2**

Calculated Mr: **13980** Calculated *Pi*: **4.61**

Annotated MS **spectra:**

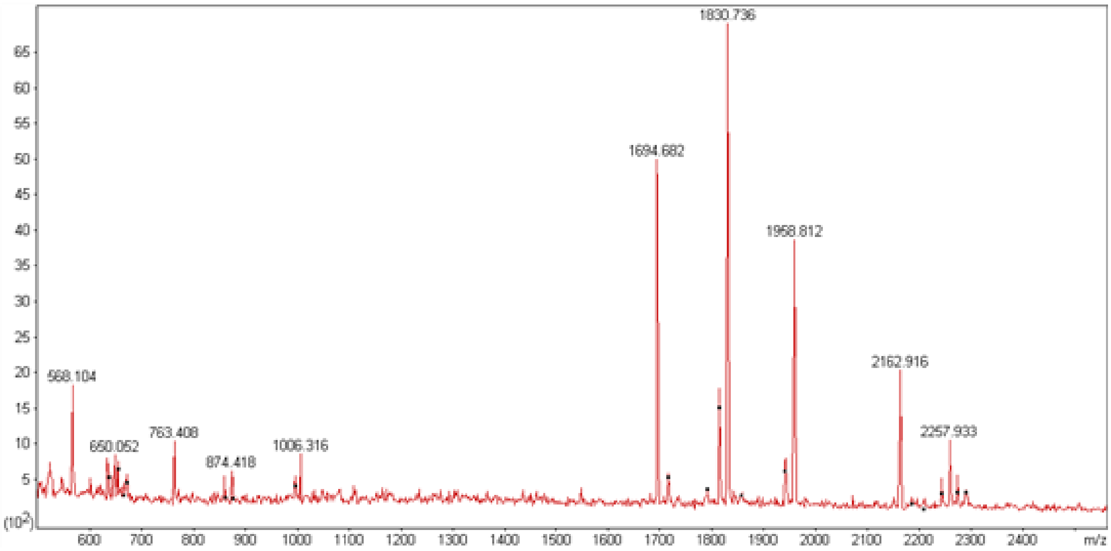

Probability Based Mowse Score:

Ions score is -10*Log(P), where P is the probability that the observed match is a random event.
Individual ions scores > 47 indicate identity or extensive homology (p<0.05).
Protein scores are derived from ions scores as a non-probabilistic basis for ranking protein hits.

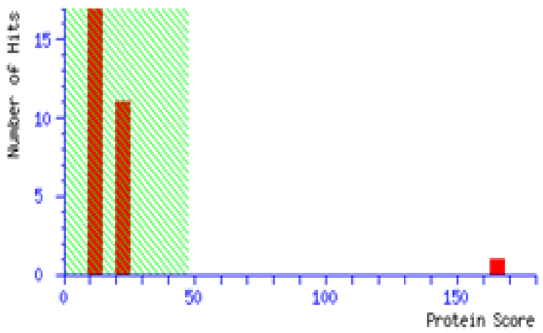

Matched peptide sequences: shown in Bold Red

1 MAGNFEEVAK QFVEFYYNTF DSDRKGLAAL YRDNSMLTFE SASVLGTQAI
51 TEKLAGLPFE KVKHQVSTLD AQPSNDQGGV IILITGALLV DEEQRPMNFS
101 QSFQLARDAN GQYFVYNDIF KLVFG

Matched peptides information:

Query	Start - End	Observed	Mr(expt)	Mr(calc)	ppm	M	Score	Expect	Rank	U	Peptide
[illegible]	11 - 24	1830.7360	1829.7287	1829.7897	-33.3	0	80	2.0e-05	1	U	K.QFVEFYYNTFDSDR.K
[illegible]	11 - 25	1958.8120	1957.8047	1957.8846	-40.8	1	86	7.3e-06	1	U	K.QFVEFYYNTFDSDRK.G

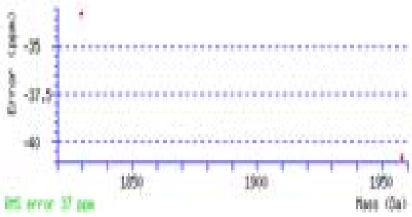

Annotated ion spectra of the matched peptides with p≤0.05:

CID No.: **48-1830.7**

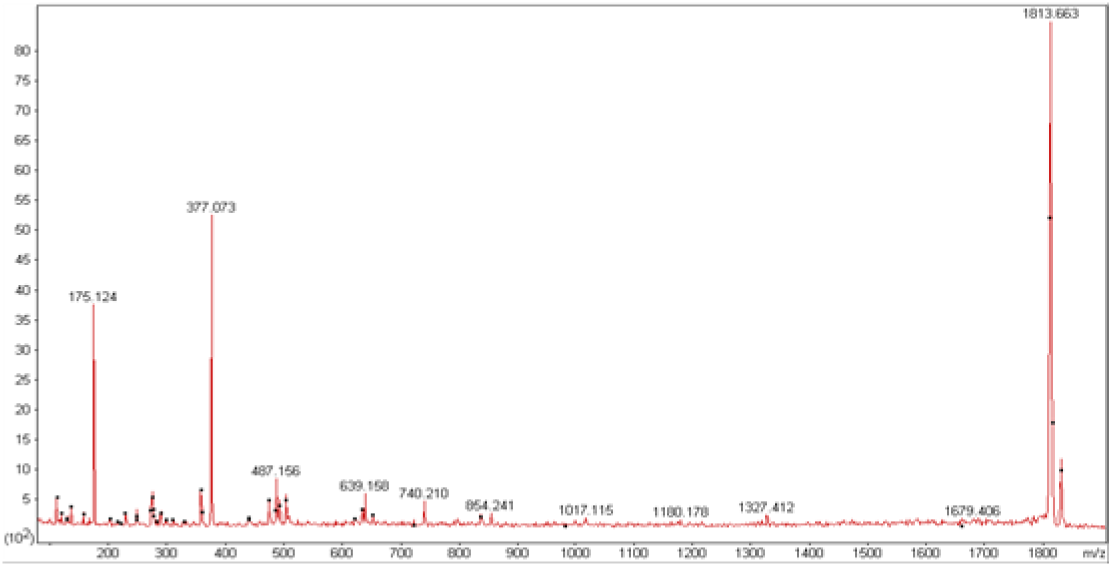

MS/MS Fragmentation of **K.QFVEFYYNTFDSDR.K**

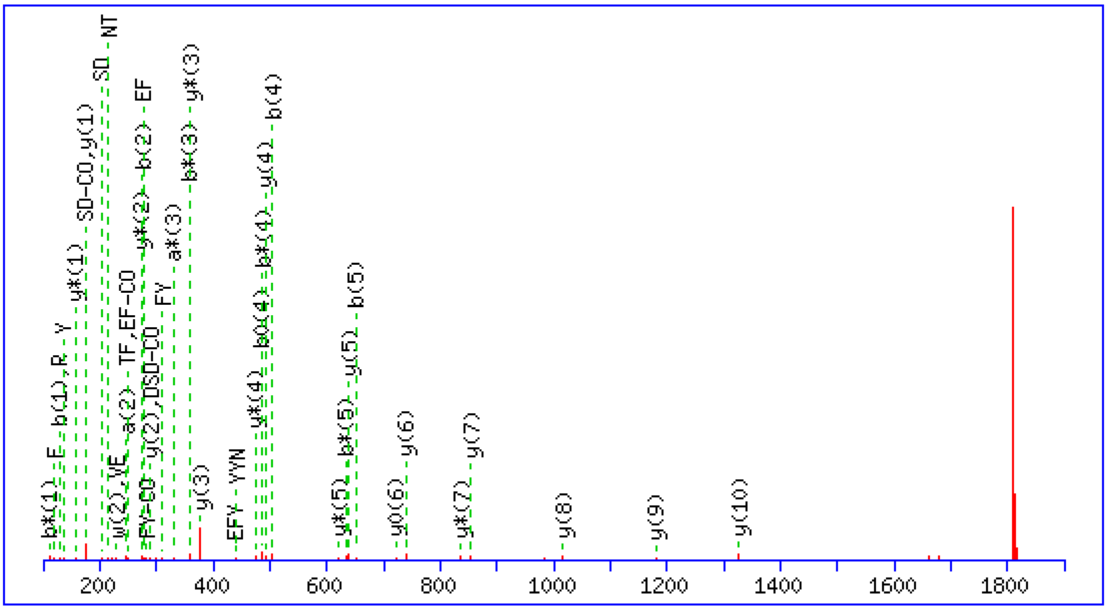

Monoisotopic mass of neutral peptide Mr(calc): 1829.7897

#	Immon.	a	a*	a^0	b	b*	b^0	d	d'	Seq.	v	w	w'	y	y*	y^0	#
1	101.0709	101.0709	84.0444		**129.0659**	112.0393		44.0495		**Q**							14
2	120.0808	248.1394	231.1128		**276.1343**	259.1077				**F**	1610.6758			1702.7384	1685.7118	1684.7278	13
3	72.0808	347.2078	330.1812		375.2027	358.1761		333.1921		**V**	1511.6074	1524.6278		1555.6700	1538.6434	1537.6594	12
4	102.0550	476.2504	459.2238	458.2398	**504.2453**	487.2187	486.2347	418.2449		**E**	1382.5648	1381.5695		1456.6016	1439.5750	1438.5910	11
5	120.0808	623.3188	606.2922	605.3082	**651.3137**	634.2871	633.3031			**F**	1235.4964			***1327.5590***	1310.5324	1309.5484	10
6	136.0757	786.3821	769.3556	768.3715	814.3770	797.3505	796.3665			**Y**	1072.4330			***1180.4905***	1163.4640	1162.4800	9
7	136.0757	949.4454	932.4189	931.4349	977.4403	960.4138	959.4298			**Y**	909.3697			***1017.4272***	1000.4007	999.4167	8
8	87.0553	1063.4884	1046.4618	1045.4778	1091.4833	1074.4567	1073.4727	1020.4825		**N**	795.3268	794.3315		***854.3639***	837.3373	836.3533	7
9	74.0600	1164.5360	1147.5095	1146.5255	1192.5310	1175.5044	1174.5204	1148.5411	1150.5204	**T**	694.2791	707.2995	709.2788	***740.3210***	723.2944	722.3104	6
10	120.0808	1311.6045	1294.5779	1293.5939	1339.5994	1322.5728	1321.5888			**F**	547.2107			***639.2733***	622.2467	621.2627	5
11	88.0393	1426.6314	1409.6048	1408.6208	1454.6263	1437.5998	1436.6157	1382.6416		**D**	432.1837	431.1885		***492.2049***	475.1783	474.1943	4
12	60.0444	1513.6634	1496.6369	1495.6529	1541.6583	1524.6318	1523.6478	1497.6685		**S**	345.1517	344.1565		***377.1779***	360.1514	359.1674	3
13	88.0393	1628.6904	1611.6638	1610.6798	1656.6853	1639.6587	1638.6747	1584.7005		**D**	230.1248	229.1295		***290.1459***	273.1193	272.1353	2
14	129.1135									**R**	74.0237	73.0284		***175.1190***	158.0924		1

Seq	ya	yb	Seq	ya	yb	Seq	ya	yb
FV	219.1492	247.1441	FVE	348.1918	376.1867	FVEF	495.2602	523.2551
FVEFY	658.3235	686.3184	VE	201.1234	229.1183	VEF	348.1918	376.1867
VEFY	511.2551	539.2500	VEFYY	674.3184	702.3134	EF	249.1234	277.1183
EFY	412.1867	440.1816	EFYY	575.2500	603.2449	EFYYN	689.2930	717.2879
FY	283.1441	311.1390	FYY	446.2074	474.2023	FYYN	560.2504	588.2453
FYYNT	661.2980	689.2930	YY	299.1390	327.1339	YYN	413.1819	441.1769
YYNT	514.2296	542.2245	YYNTF	661.2980	689.2930	YN	250.1186	278.1135
YNT	351.1663	379.1612	YNTF	498.2347	526.2296	YNTFD	613.2617	641.2566
NT	188.1030	216.0979	NTF	335.1714	363.1663	NTFD	450.1983	478.1932
NTFDS	537.2304	565.2253	NTFDSD	652.2573	680.2522	TF	221.1285	249.1234
TFD	336.1554	364.1503	TFDS	423.1874	451.1823	TFDSD	538.2144	566.2093
FD	235.1077	263.1026	FDS	322.1397	350.1347	FDSD	437.1667	465.1616
DS	175.0713	203.0662	DSD	290.0983	318.0932	SD	175.0713	203.0662

Annotated ion spectra of the matched peptides with p≤0.05:

CID No.: **48-1958.8**

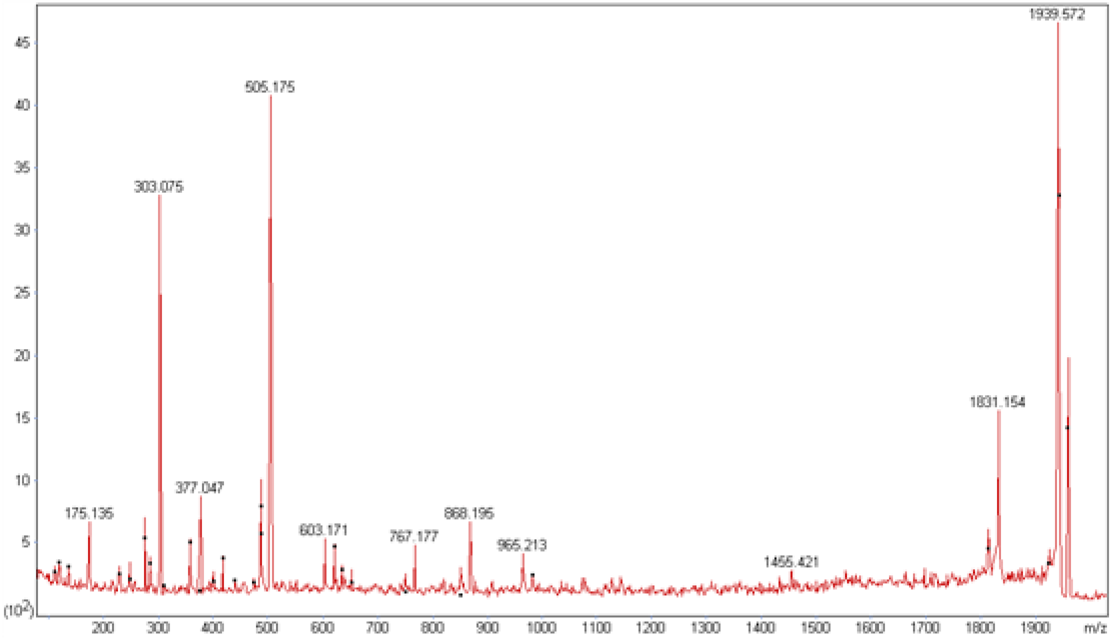

MS/MS Fragmentation of **K.QFVEFYYNTFDSDRK.G**

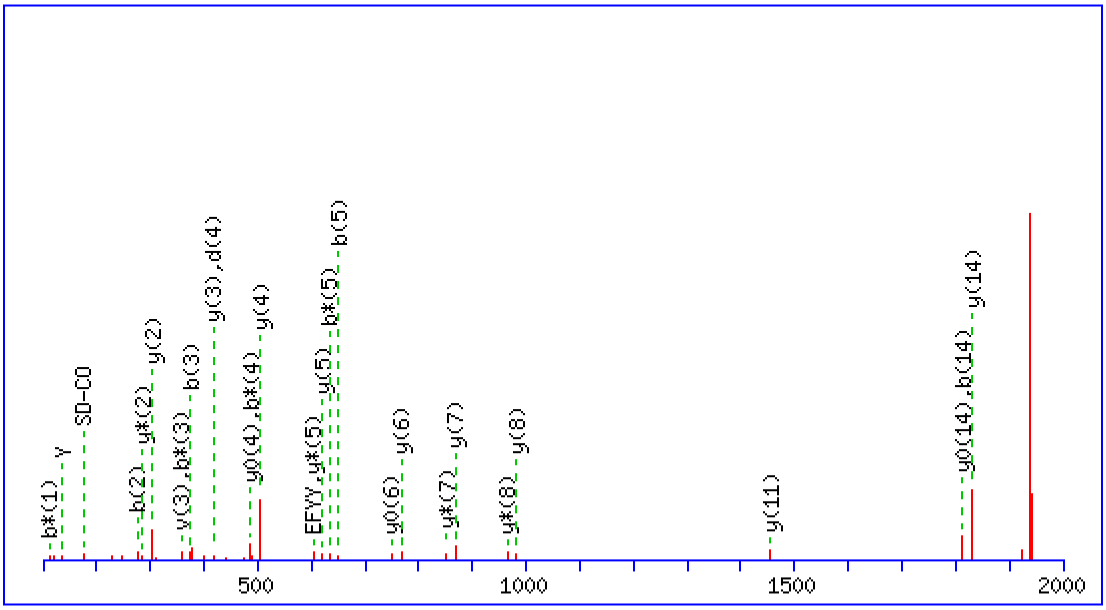

Monoisotopic mass of neutral peptide Mr(calc): 1957.8846

#	Immon.	a	a*	a^0	b	b*	b^0	d	d'	Seq.	v	w	w'	y	y*	y^0	#
1	101.0709	101.0709	84.0444		129.0659	112.0393		44.0495		Q							15
2	120.0808	248.1394	231.1128		*276.1343*	259.1077				F	1738.7707			*1830.8333*	1813.8068	1812.8228	14
3	72.0808	347.2078	330.1812		*375.2027*	358.1761		333.1921		V	1639.7023	1652.7227		1683.7649	1666.7384	1665.7544	13
4	102.0550	476.2504	459.2238	458.2398	504.2453	487.2187	486.2347	418.2449		E	1510.6597	1509.6645		1584.6965	1567.6700	1566.6859	12
5	120.0808	623.3188	606.2922	605.3082	*651.3137*	634.2871	633.3031			F	1363.5913			*1455.6539*	1438.6274	1437.6434	11
6	136.0757	786.3821	769.3556	768.3715	814.3770	797.3505	796.3665			Y	1200.5280			1308.5855	1291.5590	1290.5749	10
7	136.0757	949.4454	932.4189	931.4349	977.4403	960.4138	959.4298			Y	1037.4647			1145.5222	1128.4956	1127.5116	9
8	87.0553	1063.4884	1046.4618	1045.4778	1091.4833	1074.4567	1073.4727	1020.4825		N	923.4217	922.4265		*982.4588*	965.4323	964.4483	8
9	74.0600	1164.5360	1147.5095	1146.5255	1192.5310	1175.5044	1174.5204	1148.5411	1150.5204	T	822.3741	835.3945	837.3737	*868.4159*	851.3894	850.4054	7
10	120.0808	1311.6045	1294.5779	1293.5939	1339.5994	1322.5728	1321.5888			F	675.3056			*767.3682*	750.3417	749.3577	6
11	88.0393	1426.6314	1409.6048	1408.6208	1454.6263	1437.5998	1436.6157	1382.6416		D	560.2787	559.2835		*620.2998*	603.2733	602.2893	5
12	60.0444	1513.6634	1496.6369	1495.6529	1541.6583	1524.6318	1523.6478	1497.6685		S	473.2467	472.2514		*505.2729*	488.2463	487.2623	4
13	88.0393	1628.6904	1611.6638	1610.6798	1656.6853	1639.6587	1638.6747	1584.7005		D	358.2197	357.2245		*418.2409*	401.2143	400.2303	3
14	129.1135	1784.7915	1767.7649	1766.7809	*1812.7864*	1795.7598	1794.7758	1699.7275		R	202.1186	201.1234		*303.2139*	286.1874		2
15	101.1073									K	74.0237	73.0284		147.1128	130.0863		1

Seq	ya	yb	Seq	ya	yb	Seq	ya	yb
**FV**	219.1492	247.1441	**FVE**	348.1918	376.1867	**FVEF**	495.2602	523.2551
**FVEFY**	658.3235	686.3184	**VE**	201.1234	229.1183	**VEF**	348.1918	376.1867
**VEFY**	511.2551	539.2500	**VEFYY**	674.3184	702.3134	**EF**	249.1234	277.1183
**EFY**	412.1867	440.1816	**EFYY**	575.2500	603.2449	**EFYYN**	689.2930	717.2879
**FY**	283.1441	311.1390	**FYY**	446.2074	474.2023	**FYYN**	560.2504	588.2453
**FYYNT**	661.2980	689.2930	**YY**	299.1390	327.1339	**YYN**	413.1819	441.1769
**YYNT**	514.2296	542.2245	**YYNTF**	661.2980	689.2930	**YN**	250.1186	278.1135
**YNT**	351.1663	379.1612	**YNTF**	498.2347	526.2296	**YNTFD**	613.2617	641.2566
**NT**	188.1030	216.0979	**NTF**	335.1714	363.1663	**NTFD**	450.1983	478.1932
**NTFDS**	537.2304	565.2253	**NTFDSD**	652.2573	680.2522	**TF**	221.1285	249.1234
**TFD**	336.1554	364.1503	**TFDS**	423.1874	451.1823	**TFDSD**	538.2144	566.2093
**TFDSDR**	694.3155	722.3104	**FD**	235.1077	263.1026	**FDS**	322.1397	350.1347
**FDSD**	437.1667	465.1616	**FDSDR**	593.2678	621.2627	**DS**	175.0713	203.0662
**DSD**	290.0983	318.0932	**DSDR**	446.1994	474.1943	**SD**	175.0713	203.0662
**SDR**	331.1724	359.1674	**DR**	244.1404	272.1353			

Spot No.: **49**

NCBI accession No.: **gi|475664589**

Plant species: ***Fusarium oxysporum f. sp. cubense race 4***

Protein name： **ATP synthase subunit beta, mitochondrial**

Mascot score: **117** Sequence coverage %: **5**

The number of matched peptides with p≤0.05: **2**

Calculated Mr: **41859** Calculated *P*i: **4.74**

Annotated MS **spectra:**

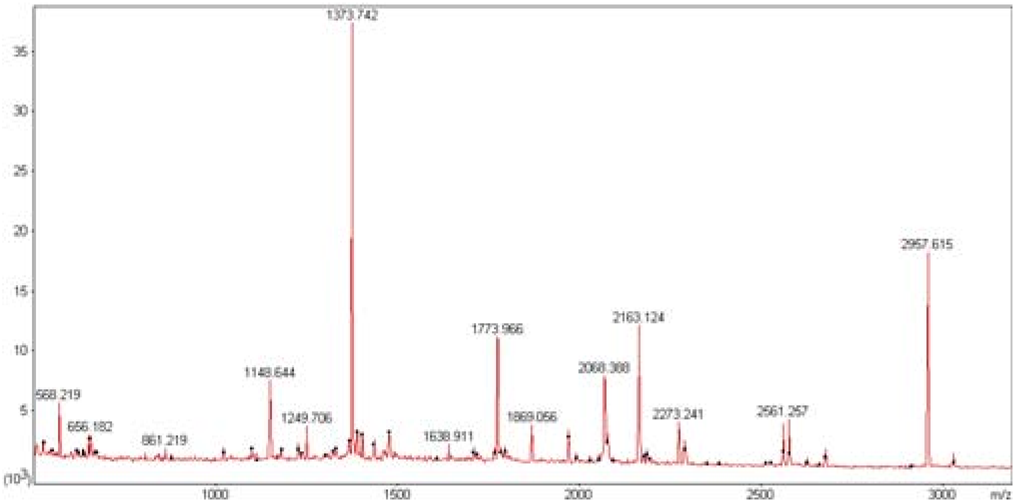

Probability Based Mowse Score:

Ions score is -10*Log(P), where P is the probability that the observed match is a random event.
Individual ions scores > 47 indicate identity or extensive homology (p<0.05).
Protein scores are derived from ions scores as a non-probabilistic basis for ranking protein hits.

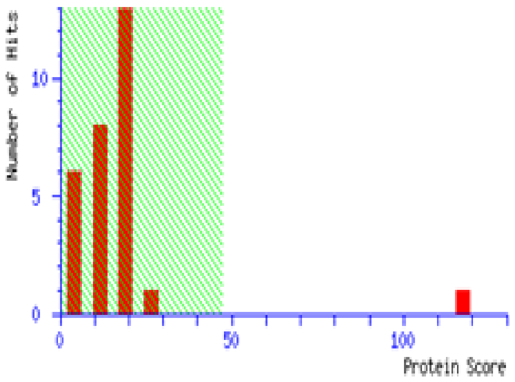

Matched peptide sequences: shown in Bold Red

```
  1 MLSTSGSLIF DIDVEFKTDK RLPIHTEAPE FVEQSTSAEV LVTGIKVVDL
 51 LAPYARGGKI GLFGGAGVGK TVFIQELINN IAKAHGGYSV FTGVGERTRE
101 GNDLYHEMQE TSVIQLDGES KVALVFGQMN EPPGARARVA LTGLTVAEYF
151 RDEEGQDVLL FIDNIFRFTQ AGSEVSALLG RIPSAVGYQP TLAVDMGGMQ
201 ERITTTTKGS ITSVQAVYVP ADDLTDPAPA TTFAHLDATT VLSRGISELG
251 IYPAVDPLDS KSRMLDPRVV GQEHYDVATR VQQILQEYKS LQDIIAILGM
301 DELSEADKLT VERARKIQRF LSQPFTVAQV FTGIEGKLVD LKETINSFKA
351 ILNGEGDNLP EGAFYMVGDF ASAKAKGEKI LAELEGQ
```

Matched peptides information:

Query	Start - End	Observed	Mr(expt)	Mr(calc)	ppm	M	Score	Expect	Rank	U	Peptide
[illegible]	245 - 261	1773.9659	1772.9586	1772.9196	22.0	0	50	0.029	1	U	R.GISELGIYPAVDPLDSK.S
[illegible]	269 - 280	1373.7419	1372.7346	1372.6735	44.5	0	67	0.00077	1	U	R.VVGQEHYDVATR.V

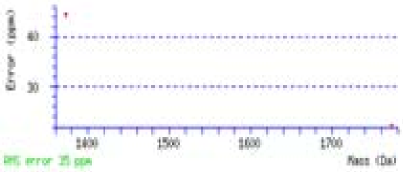

Annotated ion spectra of the matched peptides with p≤0.05:

CID No.: **49-1373.7**

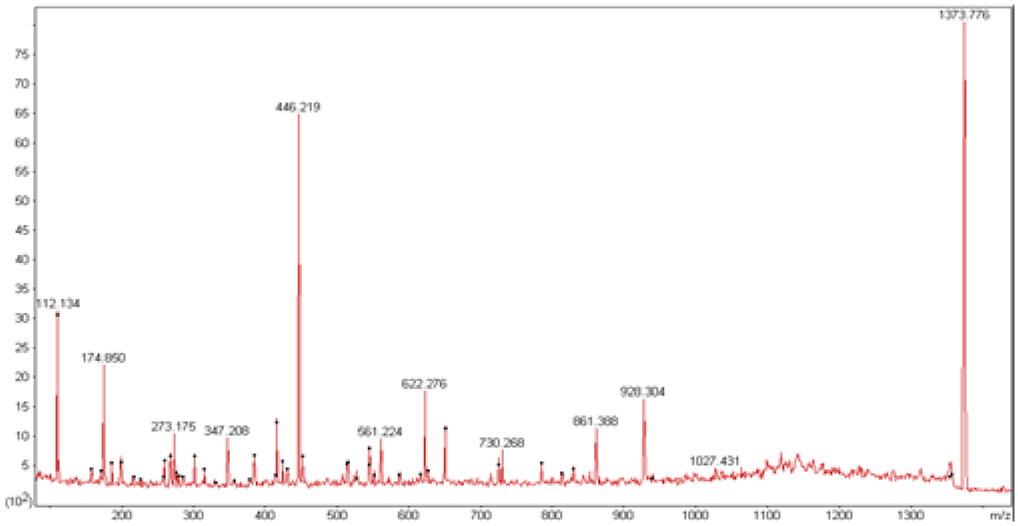

MS/MS Fragmentation of **R.VVGQEHYDVATR.V**

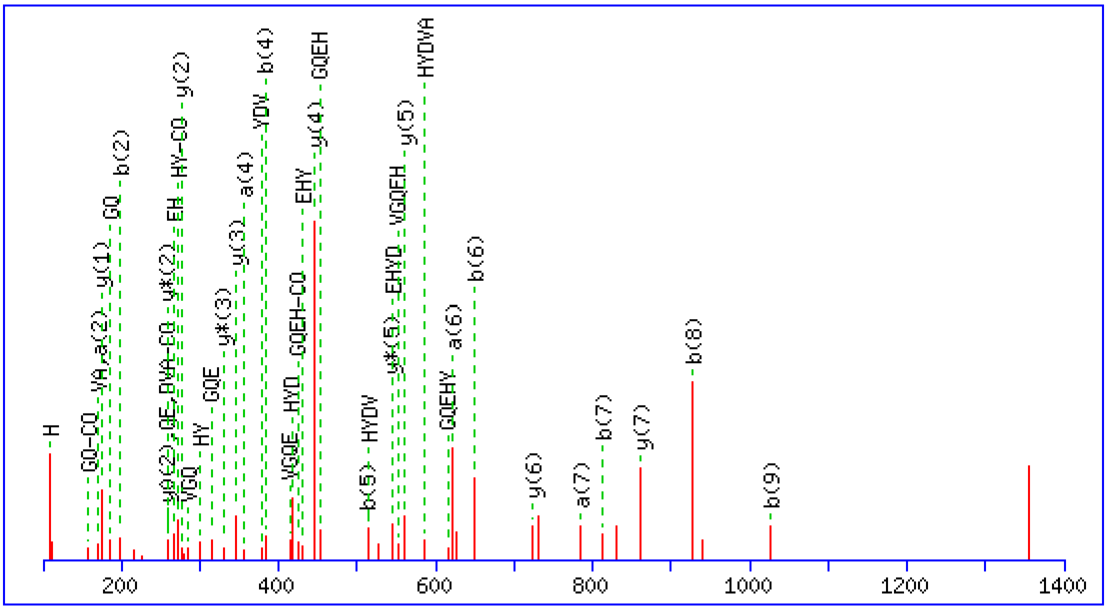

Monoisotopic mass of neutral peptide Mr(calc): 1372.6735

#	Immon.	a	a*	a^0	b	b*	b^0	d	d'	Seq.	v	w	w'	y	y*	y^0	#
1	72.0808	72.0808			100.0757			44.0495		V							12
2	72.0808	**171.1492**			***199.1441***			157.1335		V	1230.5498	1243.5702		1274.6124	1257.5858	1256.6018	11
3	30.0338	228.1707			256.1656					G				1175.5440	1158.5174	1157.5334	10
4	101.0709	**356.2292**	339.2027		***384.2241***	367.1976		299.2078		Q	1045.4698	1044.4745		1118.5225	1101.4960	1100.5119	9
5	102.0550	485.2718	468.2453	467.2613	***513.2667***	496.2402	495.2562	427.2663		E	916.4272	915.4319		990.4639	973.4374	972.4534	8
6	110.0713	**622.3307**	605.3042	604.3202	***650.3257***	633.2991	632.3151			H	779.3682			***861.4213***	844.3948	843.4108	7
7	136.0757	**785.3941**	768.3675	767.3835	***813.3890***	796.3624	795.3784			Y	616.3049			***724.3624***	707.3359	706.3519	6
8	88.0393	900.4210	883.3945	882.4104	***928.4159***	911.3894	910.4054	856.4312		D	501.2780	500.2827		***561.2991***	544.2726	543.2885	5
9	72.0808	999.4894	982.4629	981.4789	***1027.4843***	1010.4578	1009.4738	985.4738		V	402.2096	415.2300		***446.2722***	429.2456	428.2616	4
10	44.0495	1070.5265	1053.5000	1052.5160	1098.5215	1081.4949	1080.5109			A	331.1724			***347.2037***	330.1772	329.1932	3
11	74.0600	1171.5742	1154.5477	1153.5637	1199.5691	1182.5426	1181.5586	1155.5793	1157.5586	T	230.1248	243.1452	245.1244	***276.1666***	259.1401	258.1561	2
12	129.1135									R	74.0237	73.0284		***175.1190***	158.0924		1

Seq	ya	yb	Seq	ya	yb	Seq	ya	yb
VG	129.1022	157.0972	VGQ	257.1608	285.1557	VGQE	386.2034	414.1983
VGQEH	523.2623	551.2572	VGQEHY	686.3257	714.3206	GQ	158.0924	186.0873
GQE	287.1350	315.1299	GQEH	424.1939	452.1888	GQEHY	587.2572	615.2522
QE	230.1135	258.1084	QEH	367.1724	395.1674	QEHY	530.2358	558.2307
QEHYD	645.2627	673.2576	EH	239.1139	267.1088	EHY	402.1772	430.1721
EHYD	517.2041	545.1991	EHYDV	616.2726	644.2675	EHYDVA	687.3097	715.3046
HY	273.1346	301.1295	HYD	388.1615	416.1565	HYDV	487.2300	515.2249
HYDVA	558.2671	586.2620	HYDVAT	659.3148	687.3097	YD	251.1026	279.0975
YDV	350.1710	378.1660	YDVA	421.2082	449.2031	YDVAT	522.2558	550.2508
DV	187.1077	215.1026	DVA	258.1448	286.1397	DVAT	359.1925	387.1874
VA	143.1179	171.1128	VAT	244.1656	272.1605	AT	145.0972	173.0921

Annotated ion spectra of the matched peptides with p≤0.05:

CID No.: **49-1773.9**

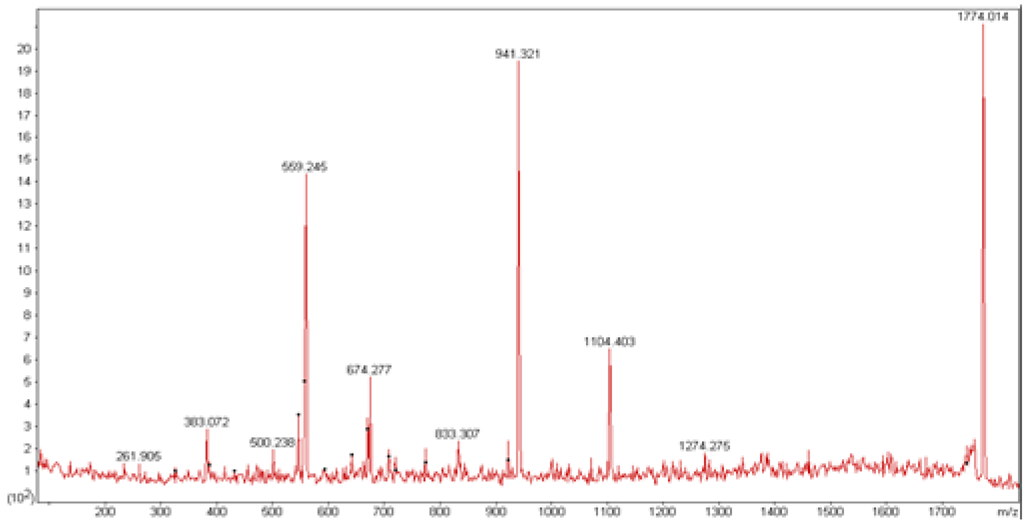

MS/MS Fragmentation of **R.GISELGIYPAVDPLDSK.S**

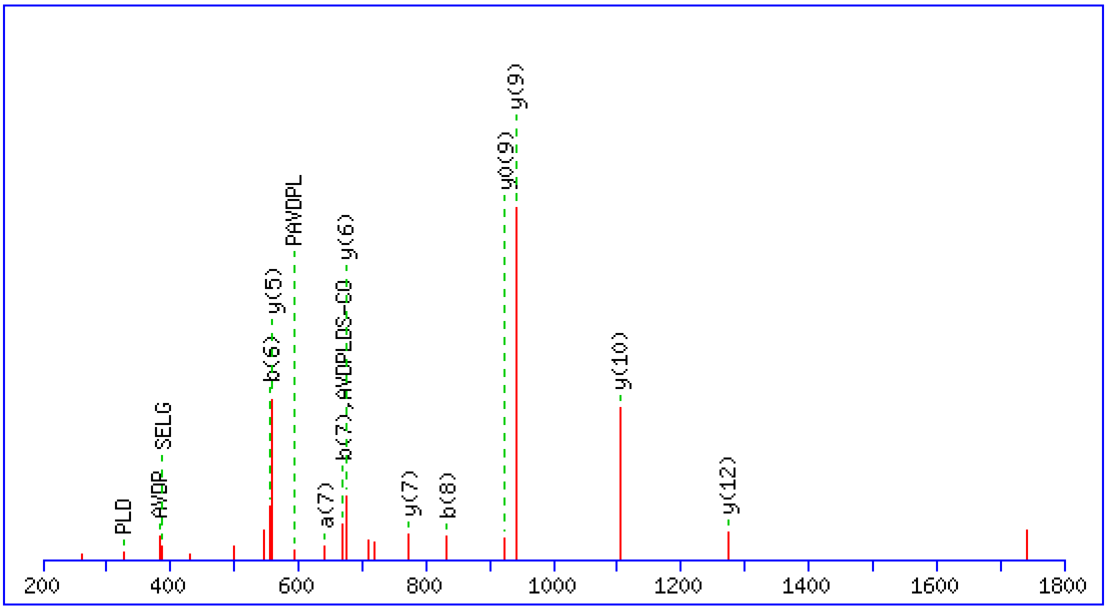

Monoisotopic mass of neutral peptide Mr(calc): 1772.9196

#	Immon.	a	a^0	b	b^0	d	d'	Seq.	v	w	w'	y	y*	y^0	#
1	30.0338	30.0338		58.0287		44.0495		G							17
2	86.0964	143.1179		171.1128		115.0866	129.1022	I	1658.8272	1671.8476	1685.8632	1716.9054	1699.8789	1698.8949	16
3	60.0444	230.1499	212.1394	258.1448	240.1343	214.1550		S	1571.7952	1570.7999		1603.8214	1586.7948	1585.8108	15
4	102.0550	359.1925	341.1819	***387.1874***	369.1769	301.1870		E	1442.7526	1441.7573		1516.7894	1499.7628	1498.7788	14
5	86.0964	472.2766	454.2660	500.2715	482.2609	430.2296		L	1329.6685	1328.6733		1387.7468	1370.7202	1369.7362	13
6	30.0338	529.2980	511.2875	***557.2930***	539.2824			G				***1274.6627***	1257.6361	1256.6521	12
7	86.0964	642.3821	624.3715	***670.3770***	652.3665	614.3508	628.3665	I	1159.5630	1172.5834	1186.5990	1217.6412	1200.6147	1199.6307	11
8	136.0757	805.4454	787.4349	***833.4403***	815.4298			Y	996.4997			***1104.5572***	1087.5306	1086.5466	10
9	70.0651	902.4982	884.4876	930.4931	912.4825	876.4825		P	899.4469	898.4516		***941.4938***	924.4673	923.4833	9
10	44.0495	973.5353	955.5247	1001.5302	983.5197			A	828.4098			844.4411	827.4145	826.4305	8
11	72.0808	1072.6037	1054.5932	1100.5986	1082.5881	1058.5881		V	729.3414	742.3618		***773.4040***	756.3774	755.3934	7
12	88.0393	1187.6307	1169.6201	1215.6256	1197.6150	1143.6408		D	614.3144	613.3192		***674.3355***	657.3090	656.3250	6
13	70.0651	1284.6834	1266.6729	1312.6783	1294.6678	1258.6678		P	517.2617	516.2664		***559.3086***	542.2821	541.2980	5
14	86.0964	1397.7675	1379.7569	1425.7624	1407.7518	1355.7205		L	404.1776	403.1823		462.2558	445.2293	444.2453	4
15	88.0393	1512.7944	1494.7839	1540.7894	1522.7788	1468.8046		D	289.1506	288.1554		349.1718	332.1452	331.1612	3
16	60.0444	1599.8265	1581.8159	1627.8214	1609.8108	1583.8316		S	202.1186	201.1234		234.1448	217.1183	216.1343	2
17	101.1073							K	74.0237	73.0284		147.1128	130.0863		1

Seq	ya	yb	Seq	ya	yb	Seq	ya	yb
IS	173.1285	201.1234	ISE	302.1710	330.1660	ISEL	415.2551	443.2500
ISELG	472.2766	500.2715	ISELGI	585.3606	613.3556	SE	189.0870	217.0819
SEL	302.1710	330.1660	SELG	359.1925	387.1874	SELGI	472.2766	500.2715
SELGIY	635.3399	663.3348	EL	215.1390	243.1339	ELG	272.1605	300.1554
ELGI	385.2445	413.2395	ELGIY	548.3079	576.3028	ELGIYP	645.3606	673.3556
LG	143.1179	171.1128	LGI	256.2020	284.1969	LGIY	419.2653	447.2602
LGIYP	516.3180	544.3130	LGIYPA	587.3552	615.3501	LGIYPAV	686.4236	714.4185
GI	143.1179	171.1128	GIY	306.1812	334.1761	GIYP	403.2340	431.2289
GIYPA	474.2711	502.2660	GIYPAV	573.3395	601.3344	GIYPAVD	688.3665	716.3614
IY	249.1598	277.1547	IYP	346.2125	374.2074	IYPA	417.2496	445.2445
IYPAV	516.3180	544.3130	IYPAVD	631.3450	659.3399	YP	233.1285	261.1234
YPA	304.1656	332.1605	YPAV	403.2340	431.2289	YPAVD	518.2609	546.2558
YPAVDP	615.3137	643.3086	PA	141.1022	169.0972	PAV	240.1707	268.1656
PAVD	355.1976	383.1925	PAVDP	452.2504	480.2453	PAVDPL	565.3344	593.3293
PAVDPLD	680.3614	708.3563	AV	143.1179	171.1128	AVD	258.1448	286.1397
AVDP	355.1976	383.1925	AVDPL	468.2817	496.2766	AVDPLD	583.3086	611.3035
AVDPLDS	670.3406	698.3355	VD	187.1077	215.1026	VDP	284.1605	312.1554
VDPL	397.2445	425.2395	VDPLD	512.2715	540.2664	VDPLDS	599.3035	627.2984
DP	185.0921	213.0870	DPL	298.1761	326.1710	DPLD	413.2031	441.1980
DPLDS	500.2351	528.2300	PL	183.1492	211.1441	PLD	298.1761	326.1710
PLDS	385.2082	413.2031	LD	201.1234	229.1183	LDS	288.1554	316.1503
DS	175.0713	203.0662						

Spot No.: **50**

Mascot score: **80**

Species: ***Fusarium oxysporum f. sp. cubense race 4***

Protein name: **Enolase**

NCBI accession No.: **gi| 475668982**

Sequence coverage %: **38**

Matched peptides No.: **17**

Total peptides No.: **63**

Calculated Mr: **47469**

Calculated *p*I: **5.06**

Annotated PMF **spectra:**

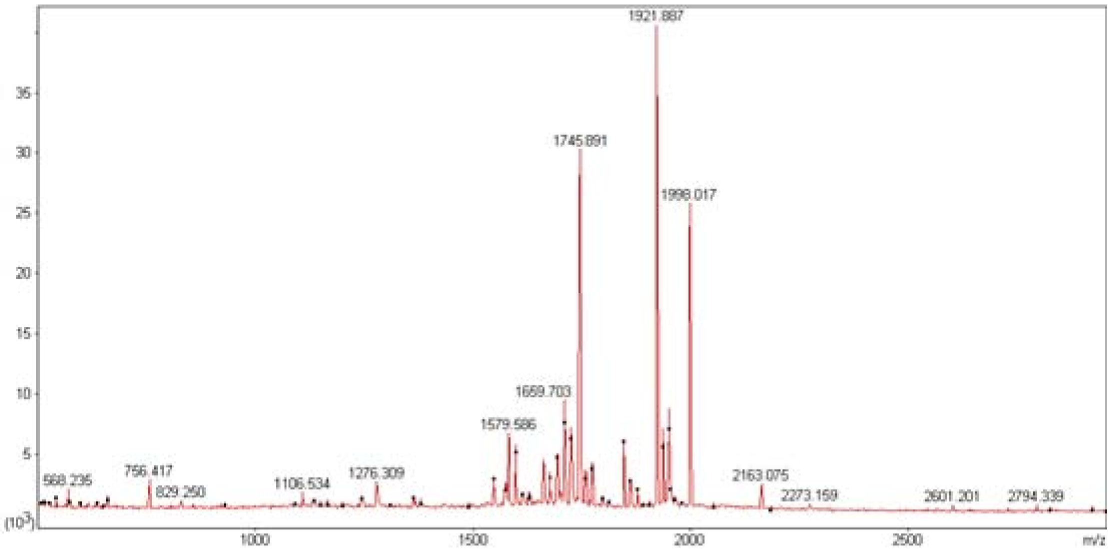

Probability Based Mowse Score:

Protein score is -10*Log(P), where P is the probability that the observed match is a random event.
Protein scores greater than 75 are significant (p<0.05).

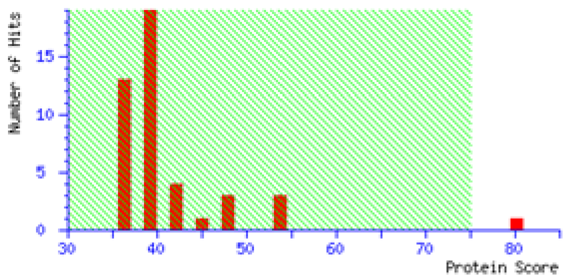

Matched peptide sequences: shown in Bold Red

1 MAVKKVFARS VYDSRGNPTV EVDVVTETGL HRAIVPSGAS TGQHEACELR

51 DGDKSKWGGK GVTKAVENVN TVIAPALIEK NLDVK**DQSAV** **DAFLNELDGT**

101 **PNKTK**LGANA ILGVSLAVAK AGAAEKGVPL YAHVSDLAGT KKPYVLPVPF

151 MNVLNGGSHA GGRLAFQEFM IVPTEAPTFT EAMR**QGAEVY** **QALK**GLAKKR

201 YGQSAGNVGD EGGVAPDIQT AEEALELITD AIEQVGYTGK IK**IAMDVASS**

251 **EFYKVEEKKY** **DLDFK**NPESD PTKWITYEEL ANLYSELCK**K** **YPIVSIEDPF**

301 **AEDDWEAWSY** **FSKTQDIQIV** **GDDLTVTNPL** **R**IKKAIELKS CNALLLKVNQ

351 **IGTLTESIQA** **AKDSYADGWG** **VMVSHRSGET** **EDVTIADIAV** **GLRAGEIKTG**

401 **APARSERLAK** **LNQILRIEEE** **LGDQAIYPGA** **NFR**KSVNL

Matched peptides information:

Start	-	End	Observed	Mr(expt)	Mr(calc)	ppm	M	Peptide
86	-	105	2163.0749	2162.0676	2162.0491	8.55	1	K.DQSAVDAFLNELDGTPNKTK.L
185	-	194	1106.5336	1105.5263	1105.5768	-45.6	0	R.QGAEVYQALK.G
243	-	254	1360.5955	1359.5882	1359.6380	-36.6	0	K.IAMDVASSEFYK.V
243	-	254	1376.6424	1375.6351	1375.6329	1.58	0	K.IAMDVASSEFYK.V + Oxidation (M)
243	-	258	1845.9591	1844.9518	1844.8866	35.4	1	K.IAMDVASSEFYKVEEK.K
243	-	258	1861.9336	1860.9263	1860.8815	24.1	1	K.IAMDVASSEFYKVEEK.K + Oxidation (M)
259	-	265	928.3601	927.3529	927.4702	-126	1	K.KYDLDFK.N
290	-	313	2922.4245	2921.4172	2921.3436	25.2	1	K.KYPIVSIEDPFAEDDWEAWSYFSK.T
291	-	313	2794.3394	2793.3321	2793.2486	29.9	0	K.YPIVSIEDPFAEDDWEAWSYFSK.T
314	-	331	1998.0174	1997.0101	1997.0430	-16.4	0	K.TQDIQIVGDDLTVTNPLR.I
348	-	362	1572.8010	1571.7937	1571.8519	-37.0	0	K.VNQIGTLTESIQAAK.D
363	-	376	1579.5855	1578.5782	1578.6885	-69.9	0	K.DSYADGWGVMVSHR.S
363	-	376	1595.5588	1594.5515	1594.6835	-82.7	0	K.DSYADGWGVMVSHR.S + Oxidation (M)
377	-	393	1745.8907	1744.8834	1744.8843	-0.51	0	R.SGETEDVTIADIAVGLR.A
399	-	404	572.2901	571.2828	571.3078	-43.8	0	K.TGAPAR.S
411	-	416	756.4167	755.4094	755.4653	-74.0	0	K.LNQILR.I
417	-	433	1921.8874	1920.8801	1920.9217	-21.7	0	R.IEEELGDQAIYPGANFR.K

Spot No.: **51**

Mascot score: **216**

Species: ***Fusarium oxysporum f. sp. cubense race 1***

Protein name: **Chitooligosaccharidolytic beta-N-acetylglucosaminidase**

NCBI accession No.: **gi| 477507289**

Sequence coverage %: **48**

Matched peptides No.: **24**

Total peptides No.: **51**

Calculated Mr: **66058**

Calculated *p*I: **5.31**

Annotated PMF **spectra:**

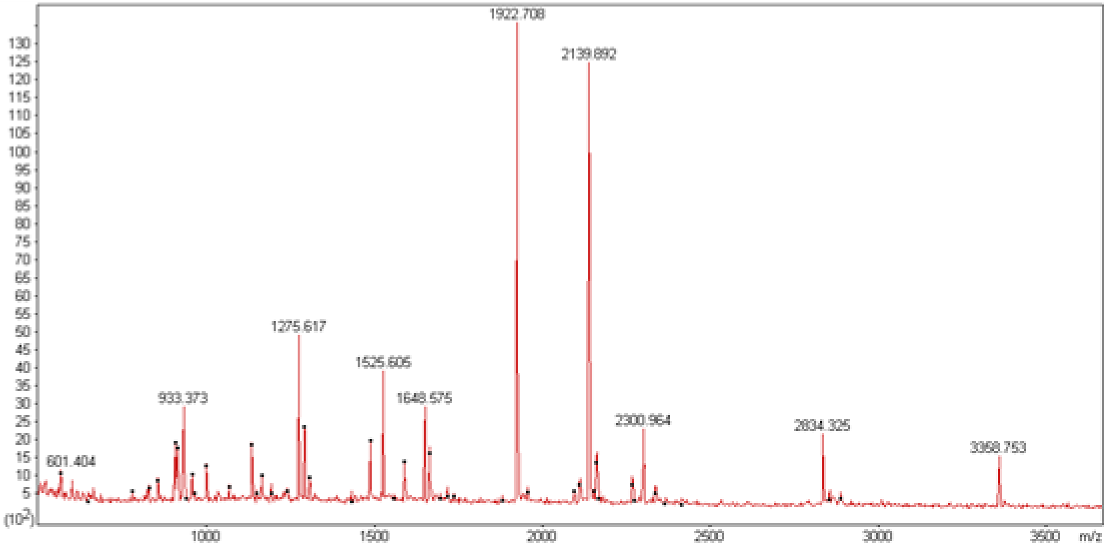

Probability Based Mowse Score:

Protein score is -10*Log(P), where P is the probability that the observed match is a random event.
Protein scores greater than 75 are significant (p<0.05).

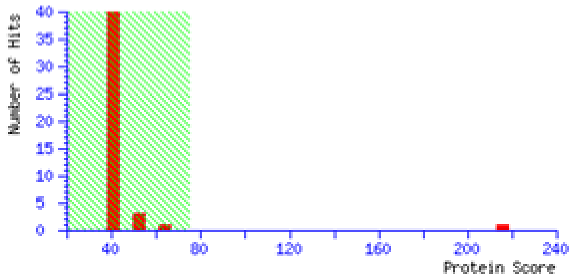

Matched peptide sequences: shown in Bold Red

```
  1 MWSKAKALLA IAAFALTPVD AIWPVPKKIS TGDKVLFIDQ SLDITYNGDF
 51 MPYTYKFQPD AGSKFNSKQI VQAGVSRALQ AIFNDNFVPW KLRERNSDFE
101 PDLQKKQWVK SLKIVQTEED DKSTFKPLAG EVDESYSLTL SEKGEASIKA
151 KSSTGILHGL ETFLQLFFKH SSGTSWYTPH APVTIQDAPE YPHRGILLDV
201 ARSFFEVEHI KRTIDAMSWS KLNRLHLHIT DSQSWPLEIP ALPKLAEKGA
251 YRKGLTYSPE DLAGIYEYGV HRGVEVIMEI DMPGHIGVVE LAYKDLIVAY
301 NEKPYQWWCK EPPCGAFRMN STDVYDFLDT LFEDLFPRIS PYSAYFHAGG
351 DELNHNDSML DPGVRSNKTE VLAPLLQKFV DYTHGKIRDA GLTPFVWEEM
401 ITEWNMTLGK DVVIQSWLGN GAVKAMAEAG HKVIDSDYNF WYLDCGRGQW
451 LNFDNGEAFK TYYPFNDWCG PTKSWRLIYS HDPRAGLSEE AAKLVLGGEA
501 AVWTETIDSV NLDTIVWPRA AVMGEVLWSG RTDASGQNRS QYDAAPRLAE
551 LRERMVARGV SASPIQMPFC TQGNATECAQ FDG
```

Matched peptides information:

Start	-	End	Observed	Mr(expt)	Mr(calc)	ppm	M	Peptide
69	-	77	957.4909	956.4837	956.5403	-59.2	0	K.QIVQAGVSR.A
78	-	91	1662.6921	1661.6848	1661.8566	-103	0	R.ALQAIFNDNFVPWK.L
96	-	105	1192.4834	1191.4761	1191.5408	-54.3	0	R.NSDFEPDLQK.K
114	-	143	3358.7529	3357.7456	3357.6351	32.9	1	K.IVQTEEDDKSTFKPLAGEVDESYSLTLSEK.G
123	-	143	2300.9642	2299.9569	2300.1423	-80.6	0	K.STFKPLAGEVDESYSLTLSEK.G
123	-	149	2886.5368	2885.5295	2885.4546	26.0	1	K.STFKPLAGEVDESYSLTLSEKGEASIK.A
170	-	194	2834.3254	2833.3181	2833.3208	-0.96	0	K.HSSGTSWYTPHAPVTIQDAPEYPHR.G
195	-	202	856.4777	855.4704	855.5178	-55.3	0	R.GILLDVAR.S
203	-	211	1135.5106	1134.5033	1134.5710	-59.6	0	R.SFFEVEHIK.R
253	-	272	2267.9497	2266.9424	2267.1222	-79.3	1	R.KGLTYSPEDLAGIYEYGVHR.G
254	-	272	2139.8918	2138.8845	2139.0273	-66.7	0	K.GLTYSPEDLAGIYEYGVHR.G
273	-	294	2415.1153	2414.1080	2414.2225	-47.4	0	R.GVEVIMEIDMPGHIGVVELAYK.D + Oxidation (M)
295	-	310	2112.8413	2111.8340	2112.0139	-85.1	0	K.DLIVAYNEKPYQWWCK.E
311	-	318	933.3735	932.3662	932.4174	-54.9	0	K.EPPCGAFR.M
379	-	386	966.3873	965.3801	965.4607	-83.5	0	K.FVDYTHGK.I
433	-	447	1922.7082	1921.7009	1921.8305	-67.4	0	K.VIDSDYNFWYLDCGR.G
448	-	460	1525.6055	1524.5982	1524.6997	-66.6	0	R.GQWLNFDNGEAFK.T
461	-	473	1648.5751	1647.5678	1647.7028	-81.9	0	K.TYYPFNDWCGPTK.S
477	-	484	1000.4477	999.4404	999.5138	-73.4	0	R.LIYSHDPR.A
494	-	519	2854.5070	2853.4997	2853.4913	2.96	0	K.LVLGGEAAVWTETIDSVNLDTIVWPR.A
520	-	531	1275.6169	1274.6096	1274.6441	-27.1	0	R.AAVMGEVLWSGR.T
520	-	531	1291.5974	1290.5901	1290.6391	-37.9	0	R.AAVMGEVLWSGR.T + Oxidation (M)
540	-	547	907.3954	906.3881	906.4195	-34.7	0	R.SQYDAAPR.L
548	-	552	601.4044	600.3971	600.3595	62.7	0	R.LAELR.E

Spot No.: **52**

Mascot score: **81**

Species: ***Fusarium oxysporum Fo5176***

Protein name: **2-nitropropane dioxygenase**

NCBI accession No.: **gi| 342879444**

Sequence coverage %: **54**

Matched peptides No.: **18**

Total peptides No.: **61**

Calculated Mr: **36989**

Calculated *p*I: **5.78**

Annotated PMF **spectra:**

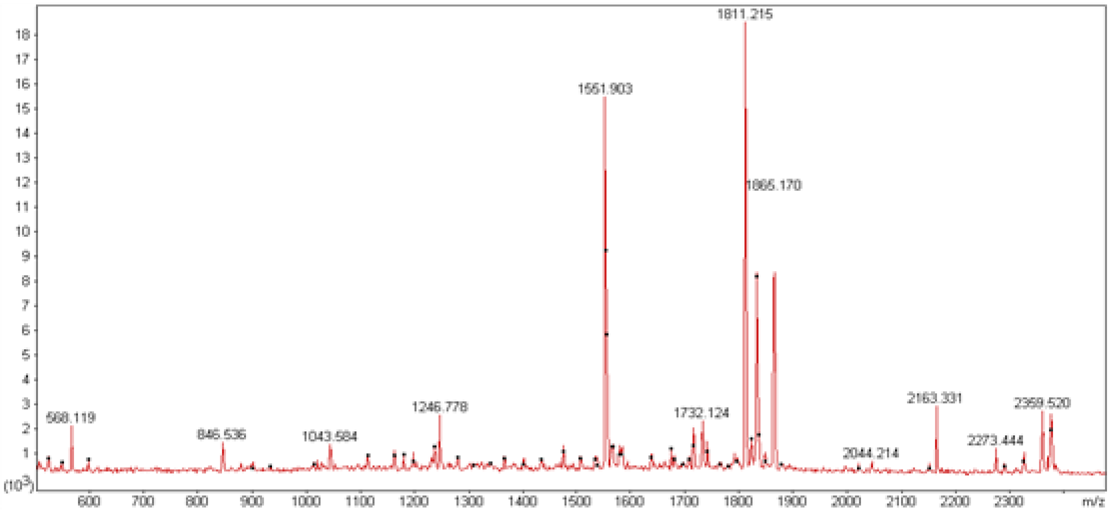

Probability Based Mowse Score:

Protein score is -10*Log(P), where P is the probability that the observed match is a random event.
Protein scores greater than 75 are significant (p<0.05).

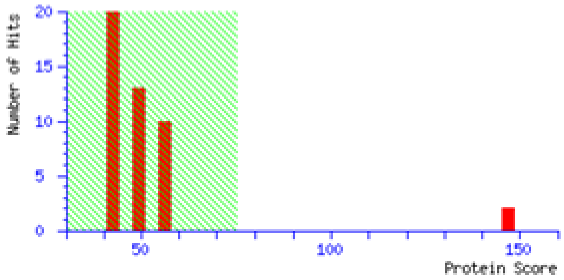

Matched peptide sequences: shown in Bold Red

1 MASPQKLR**TP** **ITDLFKIQHP** **VLLAGMNVAA** **GPKLAAAVSN** **AGGLGVIGGV**
51 **GYTPDMLREQ** **IAELKSFLND** **KNAPFGVDLL** **LPQVGGSARK** TNYDYTK**GKL**
101 **NELVDIIIEE** **GAKLFVSAVG** **VPPKAVVDKL** **HANGIVYMNM** **IGHVKHVQK**C
151 LDLGVDIICA QGGEGGGHTG DIPTTVLIPA VVDICKKHKS PLTGGPVQVI
201 AAGGIHNGQL LAAALMMGAG AVWEAVR**TSG** **HDDNIRTIIW** **TGRPMR**V**RNN**
251 **DYINDWEVNR** QAEIKELVSK GVIPYEADLD KLAEAASDND SDDDEDLLDK
301 **YRPYLMGK**CA AVVNEQKPAK **AVVDEFVDDA** **VAWLQR**GNKM IAKL

Matched peptides information:

Start	-	End	Observed	Mr(expt)	Mr(calc)	ppm	M	Peptide
9	-	16	934.5848	933.5775	933.5171	64.6	0	R.TPITDLFK.I
17	-	33	1716.1213	1715.1140	1714.9552	92.6	0	K.IQHPVLLAGMNVAAGPK.L
17	-	33	1732.1245	1731.1172	1730.9501	96.5	0	K.IQHPVLLAGMNVAAGPK.L + Oxidation (M)
34	-	58	2359.5204	2358.5131	2358.2366	117	0	K.LAAAVSNAGGLGVIGGVGYTPDMLR.E
34	-	58	2375.5307	2374.5234	2374.2315	123	0	K.LAAAVSNAGGLGVIGGVGYTPDMLR.E + Oxidation (M)
59	-	71	1534.8879	1533.8806	1533.8038	50.1	1	R.EQIAELKSFLNDK.N
72	-	89	1811.2150	1810.2077	1809.9737	129	0	K.NAPFGVDLLLPQVGGSAR.K
98	-	113	1741.1677	1740.1604	1739.9669	111	1	K.GKLNELVDIIIEEGAK.L
100	-	113	1556.0631	1555.0558	1554.8504	132	0	K.LNELVDIIIEEGAK.L
114	-	124	1113.7355	1112.7282	1112.6594	61.9	0	K.LFVSAVGVPPK.A
125	-	145	2325.5425	2324.5352	2324.2133	139	1	K.AVVDKLHANGIVYMNMIGHVK.H + Oxidation (M)
130	-	145	1797.1317	1796.1244	1795.9225	112	0	K.LHANGIVYMNMIGHVK.H
130	-	149	2289.4226	2288.4153	2288.2034	92.6	1	K.LHANGIVYMNMIGHVKHVQK.C
228	-	236	1014.5485	1013.5412	1013.4526	87.4	0	R.TSGHDDNIR.T
237	-	246	1246.7781	1245.7708	1245.6652	84.8	0	R.TIIWTGRPMR.V + Oxidation (M)
249	-	260	1551.9027	1550.8954	1550.6750	142	0	R.NNDYINDWEVNR.Q
301	-	308	1043.5837	1042.5764	1042.5270	47.4	0	K.YRPYLMGK.C + Oxidation (M)
321	-	336	1833.1703	1832.1630	1831.9105	138	0	K.AVVDEFVDDAVAWLQR.G

Spot No.: **53**

Mascot score: **76**

Species: ***Fusarium oxysporum f. sp. cubense race 4***

Protein name: **1,3-beta-glucanosyltransferase gel4**

NCBI accession No.: **gi| 475668458**

Sequence coverage %: **29**

Matched peptides No.: **15**

Total peptides No.: **62**

Calculated Mr: **59159**

Calculated *p*I: **4.83**

Annotated PMF **spectra:**

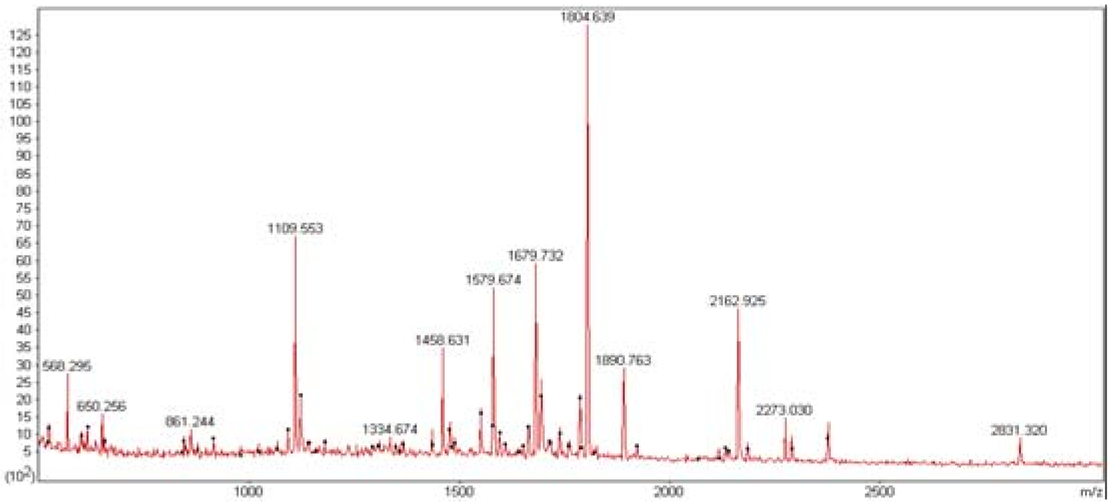

Probability Based Mowse Score:

Protein score is -10*Log(P), where P is the probability that the observed match is a random event. Protein scores greater than 75 are significant (p<0.05).

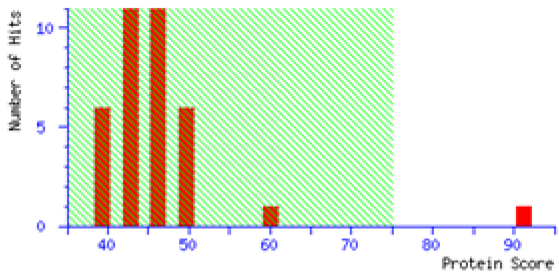

Matched peptide sequences: shown in Bold Red

1 MKFSAAIVAA AATAASAKLE PITMKGSKLF YSNGTQFFMK **GVAYQQDTAA**
51 **AGETNDKTTK** YIDPLADEDA CKR**DIPLLKQ** **LGTNIIR**TYA INPKADHKAC
101 MK**LLDDAGIY** **VISDLSEPSV** **SINRDDPKWD** **VELYER**YIGV VDELGQYDNV
151 VGFFAGNEVS NNVSNTQASA FVKAAVRDTK KHIKSKFSR**W** **LGVGYASNDD**
201 **VDIREQIADY** **FNCGDDDSRI** DYWGYNIYSW CGKSSMQDSG YADQAKFFEN
251 YSVPVFFAEY GCNEPDGAAG R**IFDETTALY** **EEK**VMTDVFS GGIVYMYFQE
301 ANDYGLVKIS KNGDAVKQKD FAQLQKKANA AKPSGVEEDS YKPTGKAATC
351 PEQSKNWR**AN** **SVLPPVPDSD** **LCDCMVKSR**S CVPADNLK**AK** **DFNDIFGYIC**
401 **GQDK**KICTAI NANATAGIYG AYSMCSNEAK **LAYILDAYYT** **SQK**SAADACD
451 FKGKATTQKA ESQDSCKSAL ASASKINEEV ATATHAVASS STGGSNSSSE
501 DDENFGLQAA SIARVFSLGD FAVGAYMAVA GVVGAGMVLL

Matched peptides information:

Start – End	Observed	Mr(expt)	Mr(calc)	ppm	M	Peptide
41 – 57	1738.6554	1737.6481	1737.7806	-76.2	0	K.GVAYQQDTAAAGETNDK.T
41 – 60	2068.8149	2067.8076	2067.9709	-78.9	1	K.GVAYQQDTAAAGETNDKTTK.Y
74 – 87	1593.6572	1592.6499	1592.9613	-196	1	R.DIPLLKQLGTNIIR.T
80 – 87	914.5843	913.5770	913.5345	46.5	0	K.QLGTNIIR.T
103 – 128	2831.3203	2830.3130	2830.4236	-39.1	1	K.LLDDAGIYVISDLSEPSVSINRDDPK.W
129 – 136	1109.5526	1108.5453	1108.5189	23.8	0	K.WDVELYER.Y
190 – 204	1679.7317	1678.7244	1678.7951	-42.1	0	R.WLGVGYASNDDVDIR.E
205 – 219	1804.6387	1803.6314	1803.7006	-38.4	0	R.EQIADYFNCGDDDSR.I
272 – 283	1458.6307	1457.6234	1457.6926	-47.4	0	R.IFDETTALYEEK.V
359 – 377	2116.8474	2115.8401	2115.9639	-58.5	0	R.ANSVLPPVPDSDLCDCMVK.S
359 – 377	2132.8154	2131.8081	2131.9588	-70.7	0	R.ANSVLPPVPDSDLCDCMVK.S + Oxidation (M)
359 – 379	2376.0964	2375.0891	2375.0920	-1.20	1	R.ANSVLPPVPDSDLCDCMVKSR.S + Oxidation (M)
389 – 404	1890.7630	1889.7557	1889.8618	-56.1	1	K.AKDFNDIFGYICGQDK.K
391 – 404	1691.7118	1690.7045	1690.7297	-14.9	0	K.DFNDIFGYICGQDK.K
431 – 443	1548.6985	1547.6912	1547.7871	-62.0	0	K.LAYILDAYYTSQK.S

Spot No.: **54**

NCBI accession No.: **gi|607818**

Plant species: ***Neurospora crassa***

Protein name：**70 kDa heat shock protein**

Mascot score: **83** Sequence coverage %: **5**

The number of matched peptides with p≤0.05: **3**

Calculated Mr: **70786** Calculated *P*i: **5.03**

Annotated MS **spectra:**

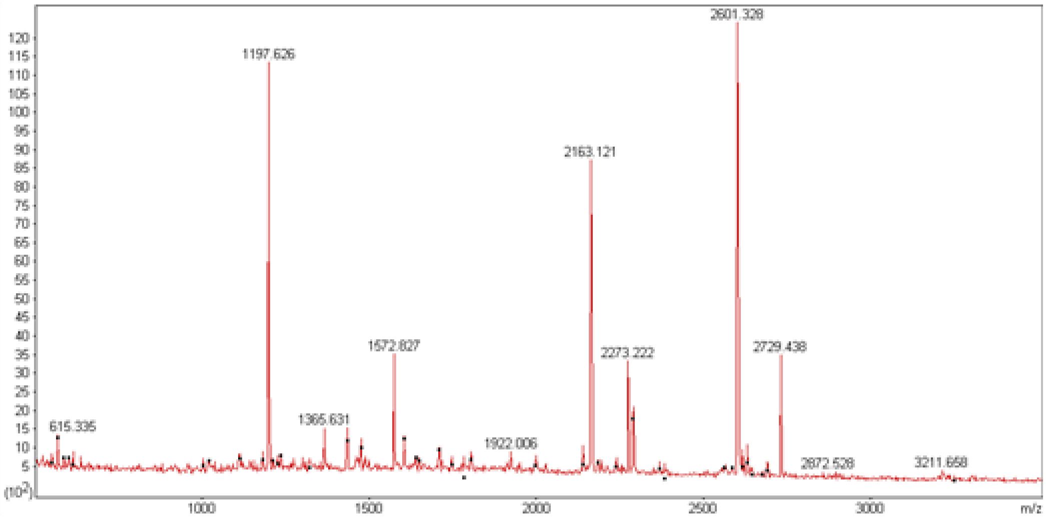

Probability Based Mowse Score:

Ions score is -10*Log(P), where P is the probability that the observed match is a random event.
Individual ions scores > 47 indicate identity or extensive homology (p<0.05).
Protein scores are derived from ions scores as a non-probabilistic basis for ranking protein hits.

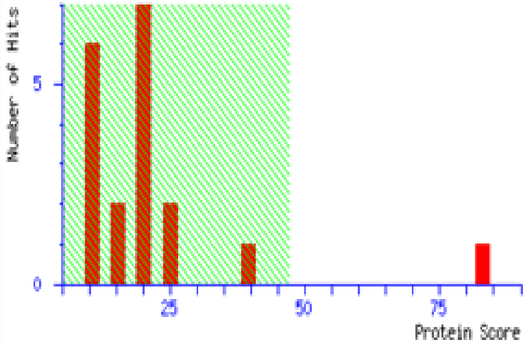

Matched peptide sequences: shown in Bold Red

1	MAPAVGIDLG	TTYSCVGVFR	EDRCEIIAND	QGNRTTPSFV	AFTDTERLVG
51	DAAKNQVAMN	PANTVFDAKR	LIGRKFSDPE	VQADMKHFPF	KVIDRGGKPV
101	IQVEFKGETK	VFTPEEISAM	ILQKMKETAE	AYLGGTVNNA	VVTVPAYFND
151	SQRQATKDAG	LIAGLNVLRI	INEPTAAAIA	YGLDKKVEGE	RNVLIFDLGG
201	GTFDVSLLTI	EEGIFEVKST	AGDTHLGGED	FDNRLVNHFV	QEFKRKDKKD
251	LSTNARALRR	LRTACERAKR	TLSSSAQTSI	EIDSLFEGID	FYTSITRARF
301	EELCQDLFRS	TLQPVDRVLT	DAKIDKSQVH	EIVLVGGSTR	IPRIQKLISD
351	YFDGKEPNKR	INPDEAVAYG	AAVQAAILSG	DTSSKSTSEI	LLLDVAPLSL
401	GIETAGGMMT	KLIPRNTTIP	TKKSEVFSTF	SDNQPGVLIQ	VYEGERQRTK
451	DNNLLGKFEL	TGIPPAPRGV	PQIEVTFDVD	ANGIMNVSAL	EKGTGKTNQI
501	TITNDKGRLS	KEEIERMLAE	AEKFKEEDEA	EAKRVAAKNG	LESYAYSLRN
551	TLSDSKVDEK	LDAADKEKLK	SEIDKIVAWL	DENQQATREE	YEERQKELEA
601	IANPIMMKFY	GAGGAPGGMP	GAAPGGFPGG	APGSNDNEGP	TVEEVD

Matched peptides information:

Query	Start - End	Observed	Mr(expt)	Mr(calc)	ppm	M	Score	Expect	Rank	U	Peptide
[illegible]	424 - 446	2601.3279	2600.3206	2600.2395	31.2	0	38	0.33	1	U	K.SEVFSTFSDNQPGVLIQVYEGER.Q
[illegible]	424 - 446	2601.3279	2600.3206	2600.2395	31.2	0	38	0.33	1	U	K.SEVFSTFSDNQPGVLIQVYEGER.Q
[illegible]	458 - 468	1197.6260	1196.6187	1196.6553	-30.6	0	45	0.12	1	U	K.FELTGIPPAPR.G

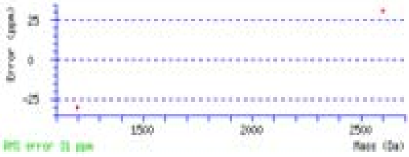

Annotated ion spectra of the matched peptides with p≤0.05:

CID No.: **54-1197.6**

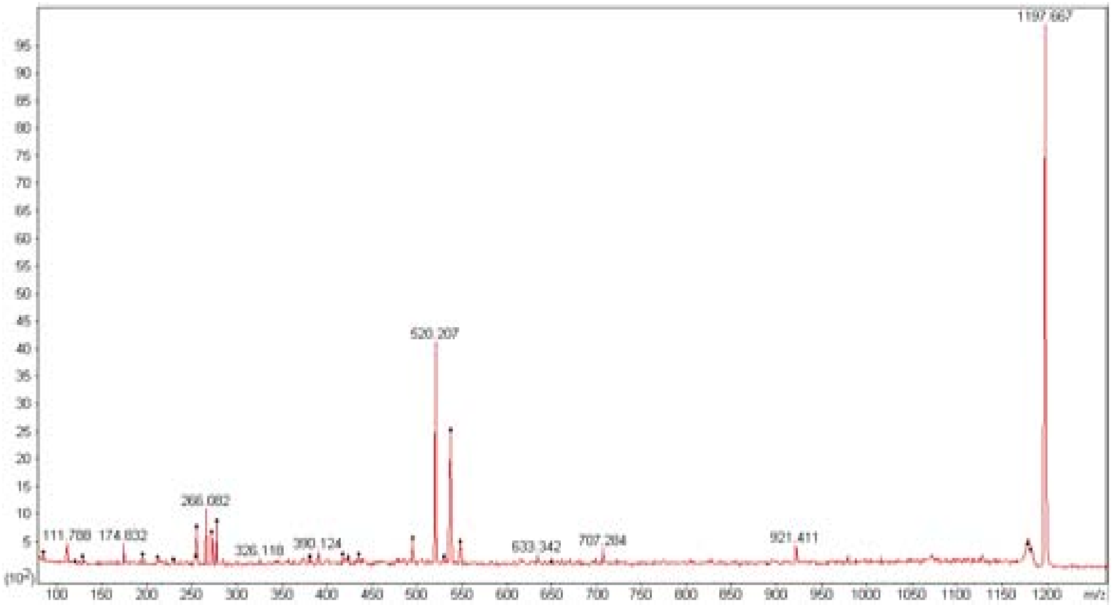

MS/MS Fragmentation of **K.FELTGIPPAPR.G**

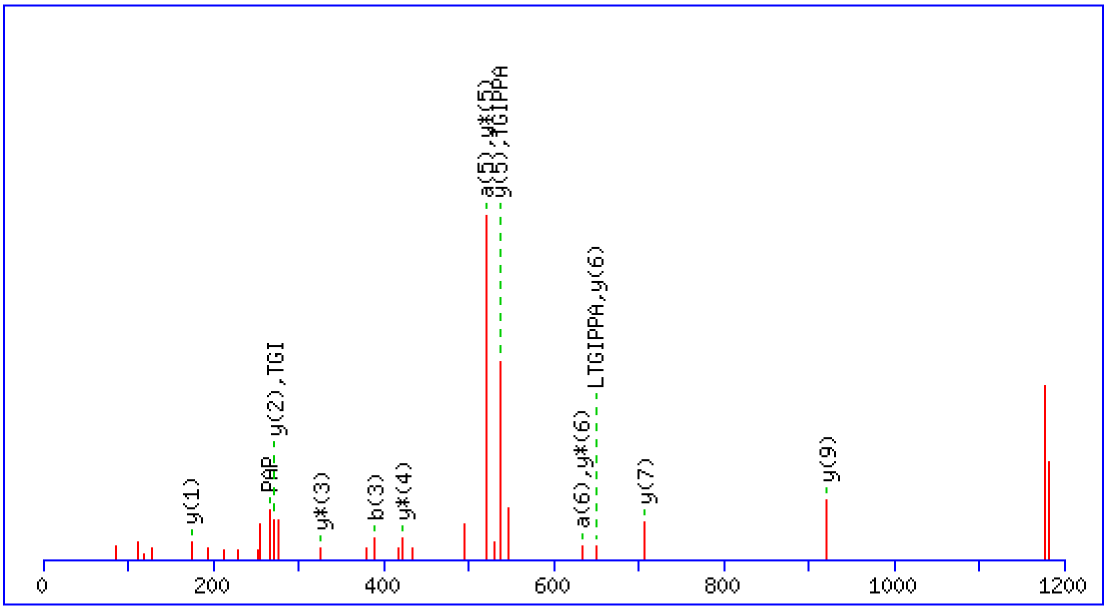

Monoisotopic mass of neutral peptide Mr(calc): 1196.6553

#	Immon.	a	a^0	b	b^0	d	d'	Seq.	v	w	w'	y	y*	y^0	#
1	120.0808	120.0808		148.0757		44.0495		F							11
2	102.0550	249.1234	231.1128	277.1183	259.1077	191.1179		E	976.5574	975.5622		1050.5942	1033.5677	1032.5837	10
3	86.0964	362.2074	344.1969	390.2023	372.1918	320.1605		L	863.4734	862.4781		***921.5516***	904.5251	903.5411	9
4	74.0600	463.2551	445.2445	491.2500	473.2395	447.2602	449.2395	T	762.4257	775.4461	777.4254	808.4676	791.4410	790.4570	8
5	30.0338	**520.2766**	502.2660	548.2715	530.2609			G				***707.4199***	690.3933		7
6	86.0964	**633.3606**	615.3501	661.3556	643.3450	605.3293	619.3450	I	592.3202	605.3406	619.3562	***650.3984***	633.3719		6
7	70.0651	730.4134	712.4028	758.4083	740.3978	704.3978		P	495.2674	494.2722		***537.3144***	520.2878		5
8	70.0651	827.4662	809.4556	855.4611	837.4505	801.4505		P	398.2146	397.2194		440.2616	423.2350		4
9	44.0495	898.5033	880.4927	926.4982	908.4876			A	327.1775			343.2088	326.1823		3
10	70.0651	995.5560	977.5455	1023.5510	1005.5404	969.5404		P	230.1248	229.1295		***272.1717***	255.1452		2
11	129.1135							R	74.0237	73.0284		***175.1190***	158.0924		1

Seq	ya	yb	Seq	ya	yb	Seq	ya	yb
EL	215.1390	243.1339	ELT	316.1867	344.1816	ELTG	373.2082	401.2031
ELTGI	486.2922	514.2871	ELTGIP	583.3450	611.3399	ELTGIPP	680.3978	708.3927
LT	187.1441	215.1390	LTG	244.1656	272.1605	LTGI	357.2496	385.2445
LTGIP	454.3024	482.2973	LTGIPP	551.3552	579.3501	LTGIPPA	622.3923	650.3872
TG	131.0815	159.0764	TGI	244.1656	272.1605	TGIP	341.2183	369.2132
TGIPP	438.2711	466.2660	TGIPPA	509.3082	537.3031	TGIPPAP	606.3610	634.3559
GI	143.1179	171.1128	GIP	240.1707	268.1656	GIPP	337.2234	365.2183
GIPPA	408.2605	436.2554	GIPPAP	505.3133	533.3082	IP	183.1492	211.1441
IPP	280.2020	308.1969	IPPA	351.2391	379.2340	IPPAP	448.2918	476.2867
PP	167.1179	195.1128	PPA	238.1550	266.1499	PPAP	335.2078	363.2027
PA	141.1022	169.0972	PAP	238.1550	266.1499	AP	141.1022	169.0972

Annotated ion spectra of the matched peptides with p≤0.05:

CID No.: **54-2601.3**

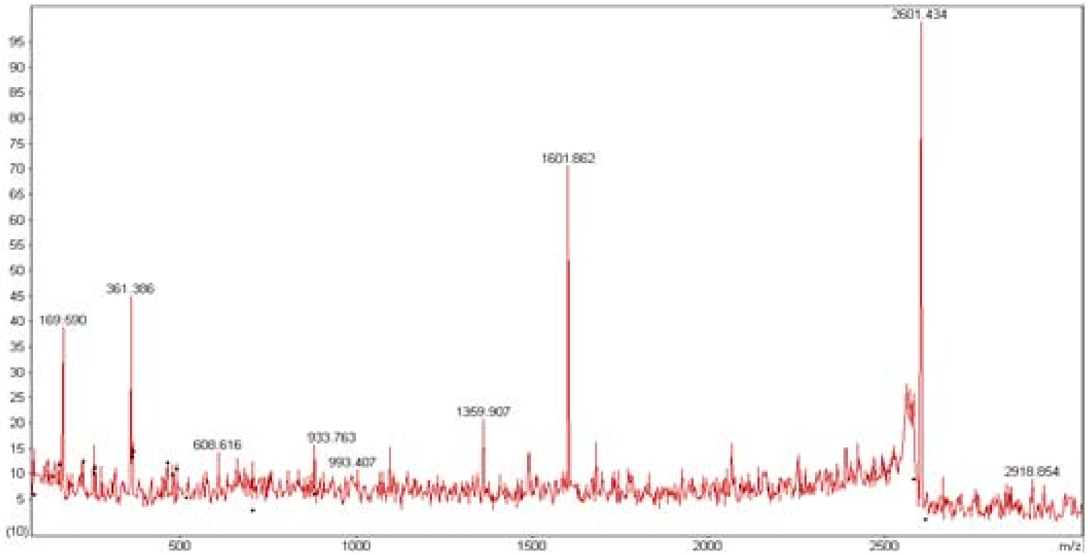

MS/MS Fragmentation of **K.SEVFSTFSDNQPGVLIQVYEGER.Q**

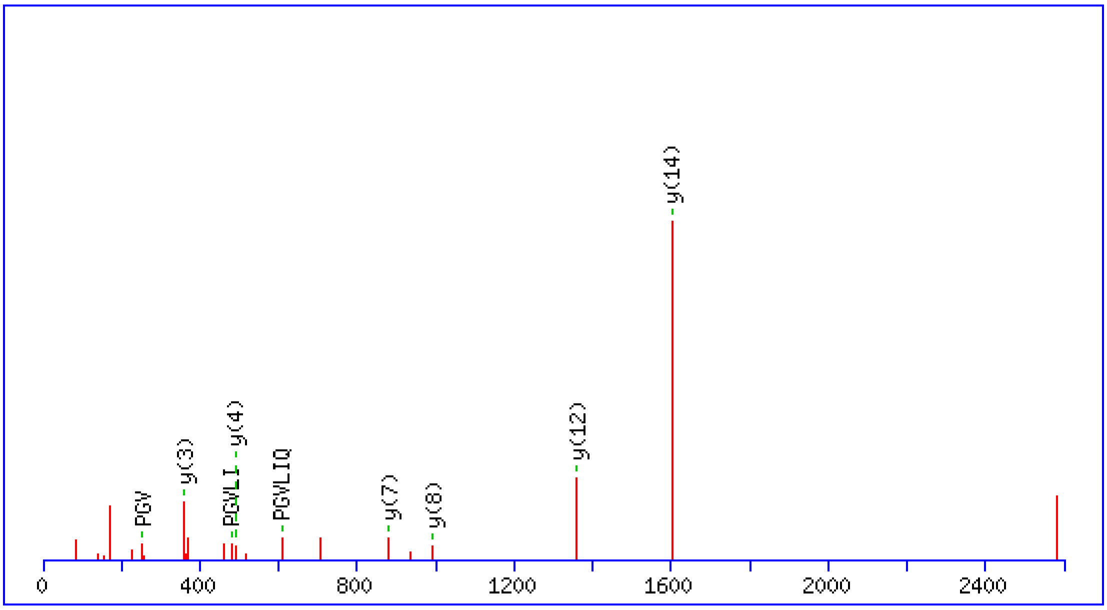

Monoisotopic mass of neutral peptide Mr(calc): 1196.6553

#	Immon.	a	a*	a^0	b	b*	b^0	d	d'	Seq.	v	w	w'	y	y*	y^0	#
1	60.0444	60.0444		42.0338	88.0393		70.0287	44.0495		S							23
2	102.0550	189.0870		171.0764	217.0819		199.0713	131.0815		E	2440.1779	2439.1827		2514.2147	2497.1882	2496.2041	22
3	72.0808	288.1554		270.1448	316.1503		298.1397	274.1397		V	2341.1095	2354.1299		2385.1721	2368.1456	2367.1616	21
4	120.0808	435.2238		417.2132	463.2187		445.2082			F	2194.0411			2286.1037	2269.0772	2268.0931	20
5	60.0444	522.2558		504.2453	550.2508		532.2402	506.2609		S	2107.0091	2106.0138		2139.0353	2122.0087	2121.0247	19
6	74.0600	623.3035		605.2930	651.2984		633.2879	607.3086	609.2879	T	2005.9614	2018.9818	2020.9611	2052.0033	2034.9767	2033.9927	18
7	120.0808	770.3719		752.3614	798.3668		780.3563			F	1858.8930			1950.9556	1933.9290	1932.9450	17
8	60.0444	857.4040		839.3934	885.3989		867.3883	841.4090		S	1771.8610	1770.8657		1803.8872	1786.8606	1785.8766	16
9	88.0393	972.4309		954.4203	1000.4258		982.4153	928.4411		D	1656.8340	1655.8388		1716.8551	1699.8286	1698.8446	15
10	87.0553	1086.4738	1069.4473	1068.4633	1114.4687	1097.4422	1096.4582	1043.4680		N	1542.7911	1541.7958		*1601.8282*	1584.8016	1583.8176	14
11	101.0709	1214.5324	1197.5059	1196.5218	1242.5273	1225.5008	1224.5168	1157.5109		Q	1414.7325	1413.7373		1487.7853	1470.7587	1469.7747	13
12	70.0651	1311.5852	1294.5586	1293.5746	1339.5801	1322.5535	1321.5695	1285.5695		P	1317.6797	1316.6845		*1359.7267*	1342.7001	1341.7161	12
13	30.0338	1368.6066	1351.5801	1350.5961	1396.6016	1379.5750	1378.5910			G				1262.6739	1245.6474	1244.6634	11
14	72.0808	1467.6750	1450.6485	1449.6645	1495.6700	1478.6434	1477.6594	1453.6594		V	1161.5899	1174.6103		1205.6525	1188.6259	1187.6419	10
15	86.0964	1580.7591	1563.7326	1562.7485	1608.7540	1591.7275	1590.7435	1538.7122		L	1048.5058	1047.5106		1106.5841	1089.5575	1088.5735	9

16	86.0964	1693.8432	1676.8166	1675.8326	1721.8381	1704.8115	1703.8275	1665.8119	1679.8275	I	935.4217	948.4421	962.4578	*993.5000*	976.4734	975.4894	8
17	101.0709	1821.9018	1804.8752	1803.8912	1849.8967	1832.8701	1831.8861	1764.8803		Q	807.3632	806.3679		*880.4159*	863.3894	862.4054	7
18	72.0808	1920.9702	1903.9436	1902.9596	1948.9651	1931.9385	1930.9545	1906.9545		V	708.2947	721.3151		752.3573	735.3308	734.3468	6
19	136.0757	2084.0335	2067.0069	2066.0229	2112.0284	2095.0019	2094.0178			Y	545.2314			653.2889	636.2624	635.2784	5
20	102.0550	2213.0761	2196.0495	2195.0655	2241.0710	2224.0445	2223.0604	2155.0706		E	416.1888	415.1936		*490.2256*	473.1991	472.2150	4
21	30.0338	2270.0976	2253.0710	2252.0870	2298.0925	2281.0659	2280.0819			G				*361.1830*	344.1565	343.1724	3
22	102.0550	2399.1401	2382.1136	2381.1296	2427.1351	2410.1085	2409.1245	2341.1347		E	230.1248	229.1295		304.1615	287.1350	286.1510	2
23	129.1135									R	74.0237	73.0284		175.1190	158.0924		1

Seq	ya	yb	Seq	ya	yb	Seq	ya	yb
EV	201.1234	229.1183	EVF	348.1918	376.1867	EVFS	435.2238	463.2187
EVFST	536.2715	564.2664	EVFSTF	683.3399	711.3348	VF	219.1492	247.1441
VFS	306.1812	334.1761	VFST	407.2289	435.2238	VFSTF	554.2973	582.2922
VFSTFS	641.3293	669.3243	FS	207.1128	235.1077	FST	308.1605	336.1554
FSTF	455.2289	483.2238	FSTFS	542.2609	570.2558	FSTFSD	657.2879	685.2828
ST	161.0921	189.0870	STF	308.1605	336.1554	STFS	395.1925	423.1874
STFSD	510.2195	538.2144	STFSDN	624.2624	652.2573	TF	221.1285	249.1234
TFS	308.1605	336.1554	TFSD	423.1874	451.1823	TFSDN	537.2304	565.2253
TFSDNQ	665.2889	693.2838	FS	207.1128	235.1077	FSD	322.1397	350.1347
FSDN	436.1827	464.1776	FSDNQ	564.2413	592.2362	FSDNQP	661.2940	689.2889
SD	175.0713	203.0662	SDN	289.1143	317.1092	SDNQ	417.1728	445.1678
SDNQP	514.2256	542.2205	SDNQPG	571.2471	599.2420	SDNQPGV	670.3155	698.3104
DN	202.0822	230.0771	DNQ	330.1408	358.1357	DNQP	427.1936	455.1885
DNQPG	484.2150	512.2100	DNQPGV	583.2835	611.2784	DNQPGVL	696.3675	724.3624
NQ	215.1139	243.1088	NQP	312.1666	340.1615	NQPG	369.1881	397.1830
NQPGV	468.2565	496.2514	NQPGVL	581.3406	609.3355	NQPGVLI	694.4246	722.4196
QP	198.1237	226.1186	QPG	255.1452	283.1401	QPGV	354.2136	382.2085
QPGVL	467.2976	495.2926	QPGVLI	580.3817	608.3766	PG	127.0866	155.0815
PGV	226.1550	254.1499	PGVL	339.2391	367.2340	PGVLI	452.3231	480.3180
PGVLIQ	580.3817	608.3766	PGVLIQV	679.4501	707.4450	GV	129.1022	157.0972
GVL	242.1863	270.1812	GVLI	355.2704	383.2653	GVLIQ	483.3289	511.3239
GVLIQV	582.3974	610.3923	VL	185.1648	213.1598	VLI	298.2489	326.2438
VLIQ	426.3075	454.3024	VLIQV	525.3759	553.3708	VLIQVY	688.4392	716.4341
LI	199.1805	227.1754	LIQ	327.2391	355.2340	LIQV	426.3075	454.3024
LIQVY	589.3708	617.3657	IQ	214.1550	242.1499	IQV	313.2234	341.2183
IQVY	476.2867	504.2817	IQVYE	605.3293	633.3243	IQVYEG	662.3508	690.3457
QV	200.1394	228.1343	QVY	363.2027	391.1976	QVYE	492.2453	520.2402
QVYEG	549.2667	577.2617	QVYEGE	678.3093	706.3042	VY	235.1441	263.1390
VYE	364.1867	392.1816	VYEG	421.2082	449.2031	VYEGE	550.2508	578.2457
YE	265.1183	293.1132	YEG	322.1397	350.1347	YEGE	451.1823	479.1773
EG	159.0764	187.0713	EGE	288.1190	316.1139	GE	159.0764	187.0713

Spot No.: **55**

Mascot score: **109**

Species: ***Fusarium oxysporum Fo5176***

Protein name: **actin lateral binding protein(Tropomyosin)**

NCBI accession No.: **gi| 342881318**

Sequence coverage %: **69**

Matched peptides No.: **16**

Total peptides No.: **89**

Calculated Mr: **18809**

Calculated *p*I: **4.84**

Annotated PMF **spectra:**

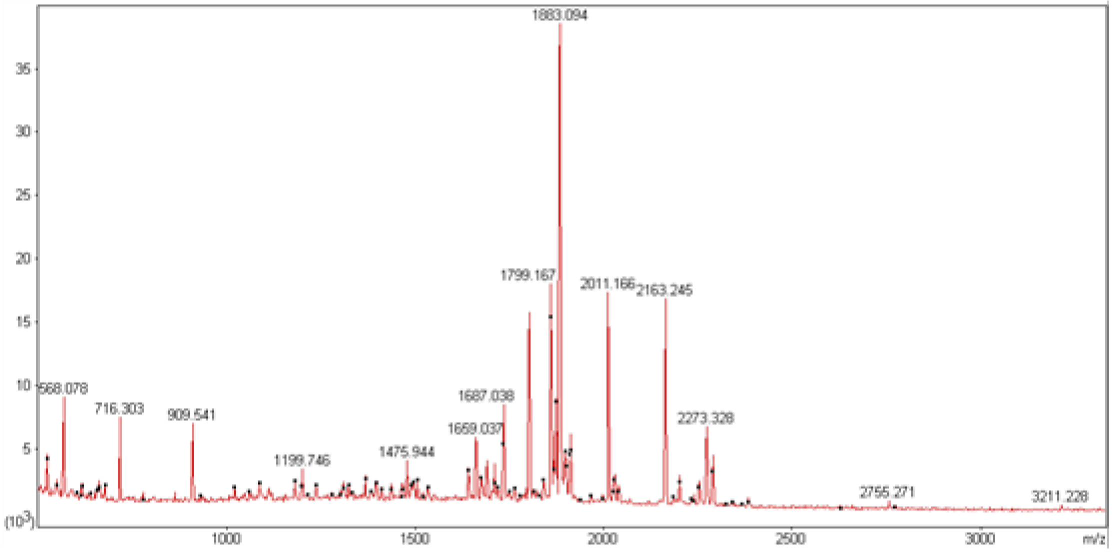

Probability Based Mowse Score:

Protein score is -10*Log(P), where P is the probability that the observed match is a random event. Protein scores greater than 75 are significant (p<0.05).

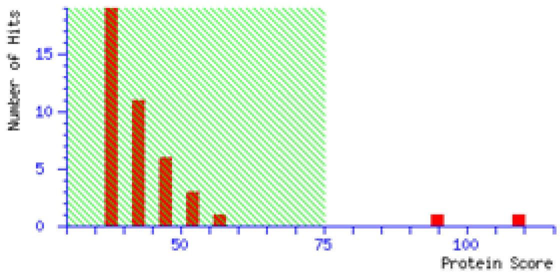

Matched peptide sequences: shown in Bold Red

1 MDRIKEK**MNQ** **LR**LEADEASG **KVEELQSKVK** **VLEQENLSKE** **QEITSLQHKN**
51 **NLLEGEVEKL** **ENAVK**DFK**KA** **ADEGQQHGTQ** **NETLQRRLQL** **LEEEAEDADK**
101 **TLR**EANEKLR QTDVK**AGHFE** **RKVQALENER** **DQWESKYEEM** **SQK**YNALQKE
151 LEELQAEIGN I

Matched peptides information:

Start	-	End	Observed	Mr(expt)	Mr(calc)	ppm	M	Peptide
8	-	12	661.2949	660.2876	660.3377	-75.8	0	K.MNQLR.L
8	-	12	677.2972	676.2899	676.3326	-63.2	0	K.MNQLR.L + Oxidation (M)
22	-	30	1059.6364	1058.6291	1058.5972	30.2	1	K.VEELQSKVK.V
31	-	49	2253.3065	2252.2992	2252.1648	59.7	1	K.VLEQENLSKEQEITSLQHK.N
40	-	49	1212.7576	1211.7503	1211.6146	112	0	K.EQEITSLQHK.N
50	-	65	1799.1667	1798.1594	1797.9472	118	1	K.NNLLEGEVEKLENAVK.D
69	-	86	2011.1663	2010.1590	2009.9515	103	1	K.KAADEGQQHGTQNETLQR.R
70	-	86	1883.0942	1882.0869	1881.8565	122	0	K.AADEGQQHGTQNETLQR.R
70	-	87	2039.1972	2038.1899	2037.9576	114	1	K.AADEGQQHGTQNETLQRR.L
87	-	100	1659.0369	1658.0296	1657.8158	129	1	R.RLQLLEEEAEDADK.T
88	-	100	1502.9069	1501.8996	1501.7147	123	0	R.LQLLEEEAEDADK.T
88	-	103	1873.1659	1872.1586	1871.9476	113	1	R.LQLLEEEAEDADKTLR.E
116	-	121	716.3030	715.2957	715.3402	-62.2	0	K.AGHFER.K
122	-	130	1086.7206	1085.7133	1085.5829	120	1	R.KVQALENER.D
123	-	136	1732.0531	1731.0458	1730.8223	129	1	K.VQALENERDQWESK.Y
137	-	143	930.4523	929.4450	929.3800	69.9	0	K.YEEMSQK.Y + Oxidation (M)

Spot No.: **56**

NCBI accession No.: **gi|475674506**

Plant species: ***Fusarium oxysporum f. sp. cubense race 4***

Protein name: **Peroxiredoxin-1**

Mascot score: **127** Sequence coverage %: **14**

The number of matched peptides with p≤0.05: **4**

Calculated Mr: **23683** Calculated *Pi*: **5.14**

Annotated MS **spectra:**

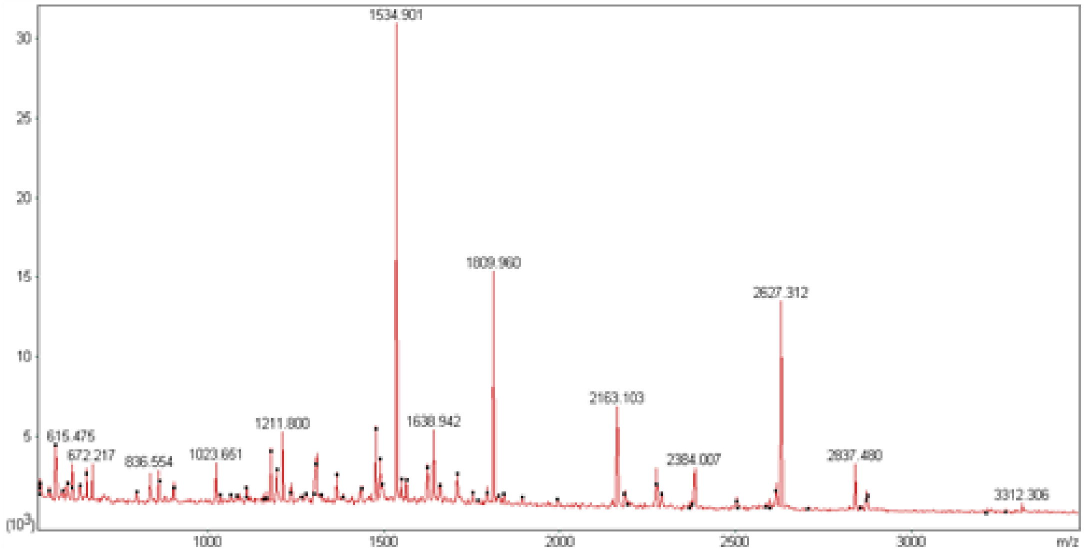

Probability Based Mowse Score:

Ions score is -10*Log(P), where P is the probability that the observed match is a random event.
Individual ions scores > 47 indicate identity or extensive homology (p<0.05).
Protein scores are derived from ions scores as a non-probabilistic basis for ranking protein hits.

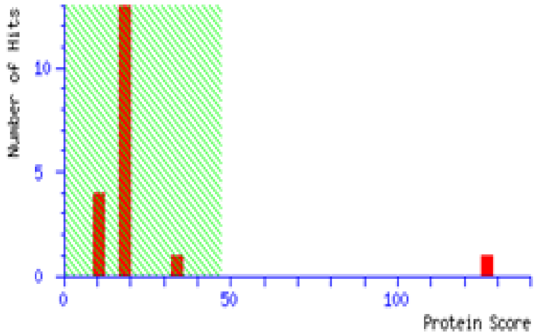

Matched peptide sequences: shown in Bold Red

1	MSSAFVQRPA	PDFSATTLFP	GGEFRDIKLS	DFKGQWVVLL	FYPMDFTFVC
51	PTEIIQYNNA	LDRFREINTT	VLGVSTDSHF	THLAWVEKPR	KQGGLGPDLE
101	LPLVADKSTK	ISRSYGVLIE	DEGIALRGLF	IIDPKGVLRQ	ITVNDLPVGR
151	DVEETIRLVK	AFQFTDEYGE	VCPAGWQEGG	KTMKADPKGS	LEYFSEQGEN
201	GESRKRPRTE				

Matched peptides information:

Query	Start - End	Observed	Mr(expt)	Mr(calc)	ppm	M	Score	Expect	Rank	U	Peptide
	9 - 25	1809.9600	1808.9527	1808.8734	43.9	0	64	0.0011	1	U	R.PAPDFSATTLFPGGEFR.D
	9 - 25	1809.9600	1808.9527	1808.8734	43.9	0	64	0.0011	1	U	R.PAPDFSATTLFPGGEFR.D
	114 - 127	1534.9010	1533.8937	1533.8038	58.6	0	63	0.0018	1	U	R.SYGVLIEDEGIALR.G
	114 - 127	1534.9010	1533.8937	1533.8038	58.6	0	63	0.0018	1	U	R.SYGVLIEDEGIALR.G

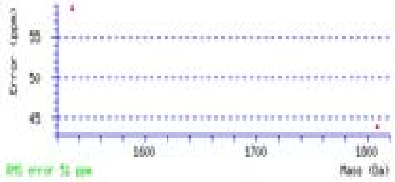

Annotated ion spectra of the matched peptides with p≤0.05:

CID No.: **56-1534.9**

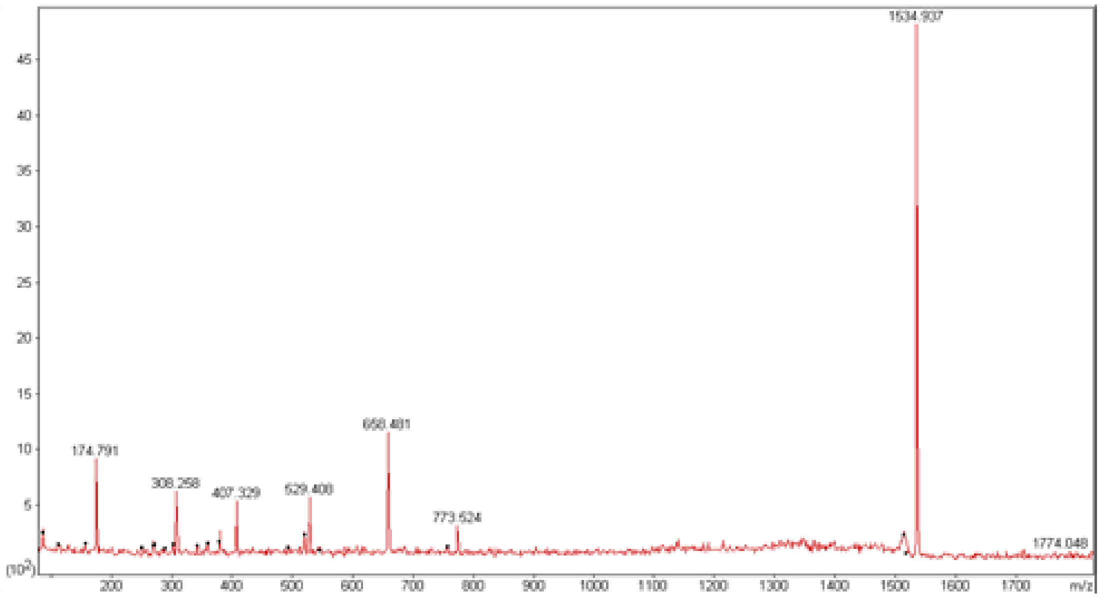

MS/MS Fragmentation of **R.SYGVLIEDEGIALR.G**

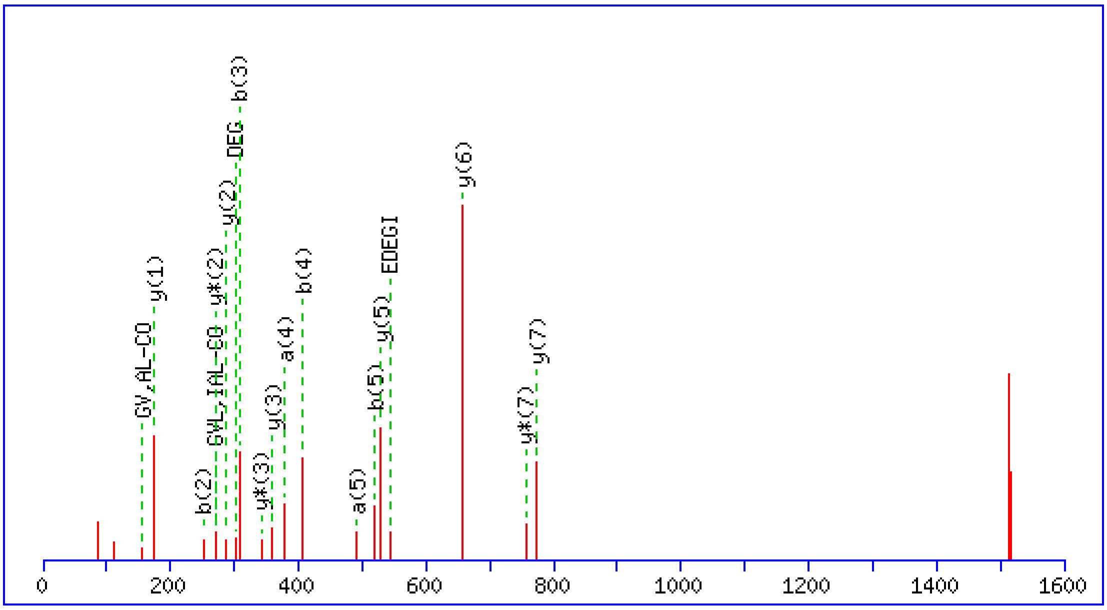

Monoisotopic mass of neutral peptide Mr(calc): 1533.8038

#	Immon.	a	a^0	b	b^0	d	d'	Seq.	v	w	w'	y	y*	y^0	#
1	60.0444	60.0444	42.0338	88.0393	70.0287	44.0495		S							14
2	136.0757	223.1077	205.0972	251.1026	233.0921			Y	1339.7216			1447.7791	1430.7526	1429.7686	13
3	30.0338	280.1292	262.1186	308.1241	290.1135			G				1284.7158	1267.6892	1266.7052	12
4	72.0808	379.1976	361.1870	407.1925	389.1819	365.1819		V	1183.6317	1196.6521		1227.6943	1210.6678	1209.6838	11
5	86.0964	492.2817	474.2711	520.2766	502.2660	450.2347		L	1070.5477	1069.5524		1128.6259	1111.5994	1110.6153	10
6	86.0964	605.3657	587.3552	633.3606	615.3501	577.3344	591.3501	I	957.4636	970.4840	984.4997	1015.5419	998.5153	997.5313	9
7	102.0550	734.4083	716.3978	762.4032	744.3927	676.4028		E	828.4210	827.4258		902.4578	885.4312	884.4472	8
8	88.0393	849.4353	831.4247	877.4302	859.4196	805.4454		D	713.3941	712.3988		773.4152	756.3886	755.4046	7
9	102.0550	978.4779	960.4673	1006.4728	988.4622	920.4724		E	584.3515	583.3562		658.3883	641.3617	640.3777	6
10	30.0338	1035.4993	1017.4888	1063.4942	1045.4837			G				529.3457	512.3191		5
11	86.0964	1148.5834	1130.5728	1176.5783	1158.5677	1120.5521	1134.5677	I	414.2459	427.2663	441.2820	472.3242	455.2976		4
12	44.0495	1219.6205	1201.6099	1247.6154	1229.6048			A	343.2088			359.2401	342.2136		3
13	86.0964	1332.7046	1314.6940	1360.6995	1342.6889	1290.6576		L	230.1248	229.1295		288.2030	271.1765		2
14	129.1135							R	74.0237	73.0284		175.1190	158.0924		1

Seq	ya	yb	Seq	ya	yb	Seq	ya	yb
YG	193.0972	221.0921	YGV	292.1656	320.1605	YGVL	405.2496	433.2445
YGVLI	518.3337	546.3286	YGVLIE	647.3763	675.3712	GV	129.1022	157.0972
GVL	242.1863	270.1812	GVLI	355.2704	383.2653	GVLIE	484.3130	512.3079
GVLIED	599.3399	627.3348	VL	185.1648	213.1598	VLI	298.2489	326.2438
VLIE	427.2915	455.2864	VLIED	542.3184	570.3134	VLIEDE	671.3610	699.3559
LI	199.1805	227.1754	LIE	328.2231	356.2180	LIED	443.2500	471.2449
LIEDE	572.2926	600.2875	LIEDEG	629.3141	657.3090	IE	215.1390	243.1339
IED	330.1660	358.1609	IEDE	459.2086	487.2035	IEDEG	516.2300	544.2249
IEDEGI	629.3141	657.3090	ED	217.0819	245.0768	EDE	346.1245	374.1194
EDEG	403.1460	431.1409	EDEGI	516.2300	544.2249	EDEGIA	587.2671	615.2620
DE	217.0819	245.0768	DEG	274.1034	302.0983	DEGI	387.1874	415.1823
DEGIA	458.2245	486.2195	DEGIAL	571.3086	599.3035	EG	159.0764	187.0713
EGI	272.1605	300.1554	EGIA	343.1976	371.1925	EGIAL	456.2817	484.2766
GI	143.1179	171.1128	GIA	214.1550	242.1499	GIAL	327.2391	355.2340
IA	157.1335	185.1285	IAL	270.2176	298.2125	AL	157.1335	185.1285

Annotated ion spectra of the matched peptides with p≤0.05:

CID No.: **56-1809.9**

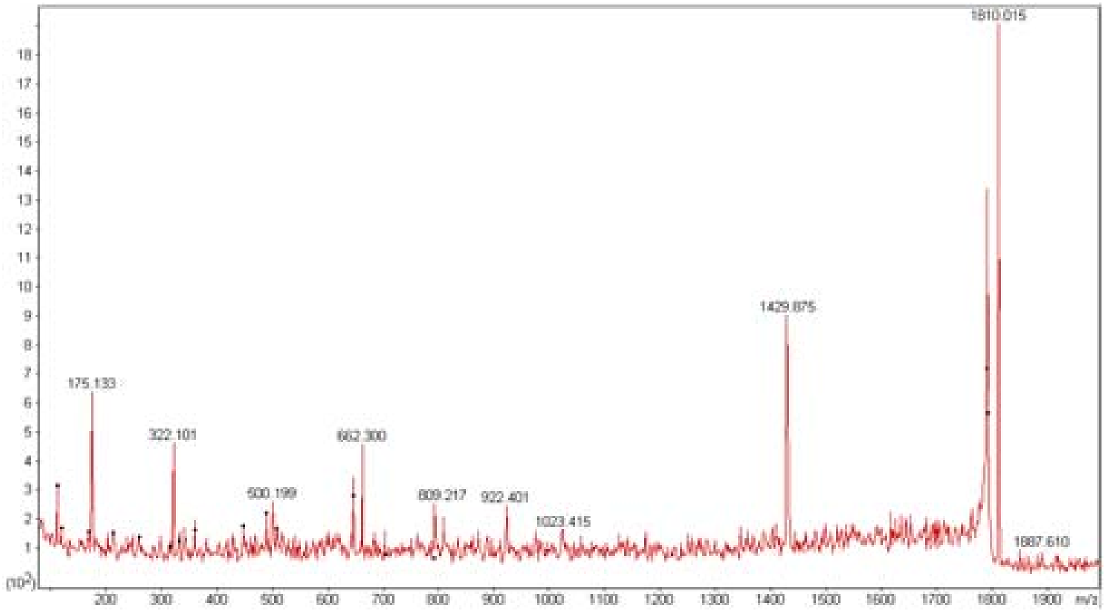

MS/MS Fragmentation of **R.PAPDFSATTLFPGGEFR.D**

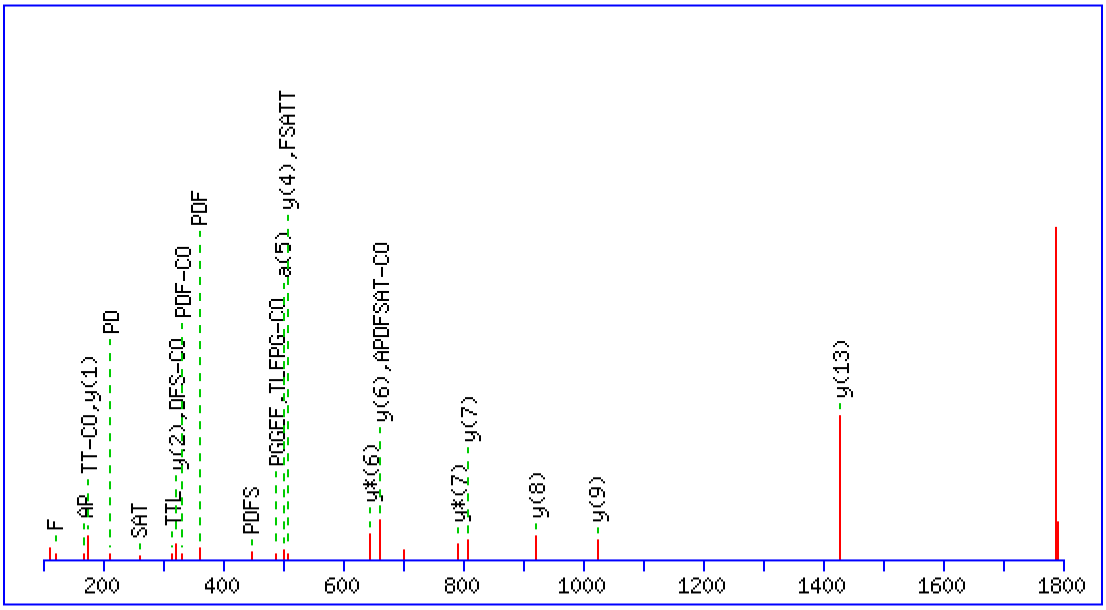

Monoisotopic mass of neutral peptide Mr(calc): 1808.8734

#	Immon.	a	a^0	b	b^0	d	d'	Seq.	v	w	w'	y	y*	y^0	#
1	70.0651	70.0651		98.0600		44.0495		P							17
2	44.0495	141.1022		169.0972				A	1696.7966			1712.8279	1695.8013	1694.8173	16
3	70.0651	238.1550		266.1499		212.1394		P	1599.7438	1598.7486		1641.7907	1624.7642	1623.7802	15
4	88.0393	353.1819	335.1714	381.1769	363.1663	309.1921		D	1484.7169	1483.7216		1544.7380	1527.7114	1526.7274	14
5	120.0808	500.2504	482.2398	528.2453	510.2347			F	1337.6484			*1429.7110*	1412.6845	1411.7005	13
6	60.0444	587.2824	569.2718	615.2773	597.2667	571.2875		S	1250.6164	1249.6212		1282.6426	1265.6161	1264.6321	12
7	44.0495	658.3195	640.3089	686.3144	668.3039			A	1179.5793			1195.6106	1178.5841	1177.6000	11
8	74.0600	759.3672	741.3566	787.3621	769.3515	743.3723	745.3515	T	1078.5316	1091.5520	1093.5313	1124.5735	1107.5469	1106.5629	10
9	74.0600	860.4149	842.4043	888.4098	870.3992	844.4199	846.3992	T	977.4839	990.5043	992.4836	*1023.5258*	1006.4993	1005.5152	9
10	86.0964	973.4989	955.4884	1001.4938	983.4833	931.4520		L	864.3999	863.4046		*922.4781*	905.4516	904.4676	8
11	120.0808	1120.5673	1102.5568	1148.5623	1130.5517			F	717.3315			*809.3941*	792.3675	791.3835	7
12	70.0651	1217.6201	1199.6095	1245.6150	1227.6045	1191.6045		P	620.2787	619.2835		*662.3257*	645.2991	644.3151	6
13	30.0338	1274.6416	1256.6310	1302.6365	1284.6259			G				565.2729	548.2463	547.2623	5
14	30.0338	1331.6630	1313.6525	1359.6579	1341.6474			G				*508.2514*	491.2249	490.2409	4
15	102.0550	1460.7056	1442.6951	1488.7005	1470.6900	1402.7001		E	377.1932	376.1979		451.2300	434.2034	433.2194	3
16	120.0808	1607.7740	1589.7635	1635.7690	1617.7584			F	230.1248			*322.1874*	305.1608		2
17	129.1135							R	74.0237	73.0284		*175.1190*	158.0924		1

Seq	ya	yb	Seq	ya	yb	Seq	ya	yb
**AP**	141.1022	169.0972	**APD**	256.1292	284.1241	**APDF**	403.1976	431.1925
**APDFS**	490.2296	518.2245	**APDFSA**	561.2667	589.2617	**APDFSAT**	662.3144	690.3093
**PD**	185.0921	213.0870	**PDF**	332.1605	360.1554	**PDFS**	419.1925	447.1874
**PDFSA**	490.2296	518.2245	**PDFSAT**	591.2773	619.2722	**PDFSATT**	692.3250	720.3199
**DF**	235.1077	263.1026	**DFS**	322.1397	350.1347	**DFSA**	393.1769	421.1718
**DFSAT**	494.2245	522.2195	**DFSATT**	595.2722	623.2671	**FS**	207.1128	235.1077
**FSA**	278.1499	306.1448	**FSAT**	379.1976	407.1925	**FSATT**	480.2453	508.2402
**FSATTL**	593.3293	621.3243	**SA**	131.0815	159.0764	**SAT**	232.1292	260.1241
**SATT**	333.1769	361.1718	**SATTL**	446.2609	474.2558	**SATTLF**	593.3293	621.3243
**SATTLFP**	690.3821	718.3770	**AT**	145.0972	173.0921	**ATT**	246.1448	274.1397
**ATTL**	359.2289	387.2238	**ATTLF**	506.2973	534.2922	**ATTLFP**	603.3501	631.3450
**ATTLFPG**	660.3715	688.3665	**TT**	175.1077	203.1026	**TTL**	288.1918	316.1867
**TTLF**	435.2602	463.2551	**TTLFP**	532.3130	560.3079	**TTLFPG**	589.3344	617.3293
**TTLFPGG**	646.3559	674.3508	**TL**	187.1441	215.1390	**TLF**	334.2125	362.2074
**TLFP**	431.2653	459.2602	**TLFPG**	488.2867	516.2817	**TLFPGG**	545.3082	573.3031
**TLFPGGE**	674.3508	702.3457	**LF**	233.1648	261.1598	**LFP**	330.2176	358.2125
**LFPG**	387.2391	415.2340	**LFPGG**	444.2605	472.2554	**LFPGGE**	573.3031	601.2980
**FP**	217.1335	245.1285	**FPG**	274.1550	302.1499	**FPGG**	331.1765	359.1714
**FPGGE**	460.2191	488.2140	**FPGGEF**	607.2875	635.2824	**PG**	127.0866	155.0815
**PGG**	184.1081	212.1030	**PGGE**	313.1506	341.1456	**PGGEF**	460.2191	488.2140
**GG**	87.0553	115.0502	**GGE**	216.0979	244.0928	**GGEF**	363.1663	391.1612
**GE**	159.0764	187.0713	**GEF**	306.1448	334.1397	**EF**	249.1234	277.1183

Spot No.: **57**

Mascot score: **79**

Species: ***Chaetomium thermophilum var.***

Protein name: **hypothetical protein CTHT_0057040**

NCBI accession No.: **gi| 340924179** Sequence coverage %: **51**

Matched peptides No.: **9** Total peptides No.: **66**

Calculated Mr: **17921** Calculated *p*I: **10.69**

Annotated PMF **spectra:**

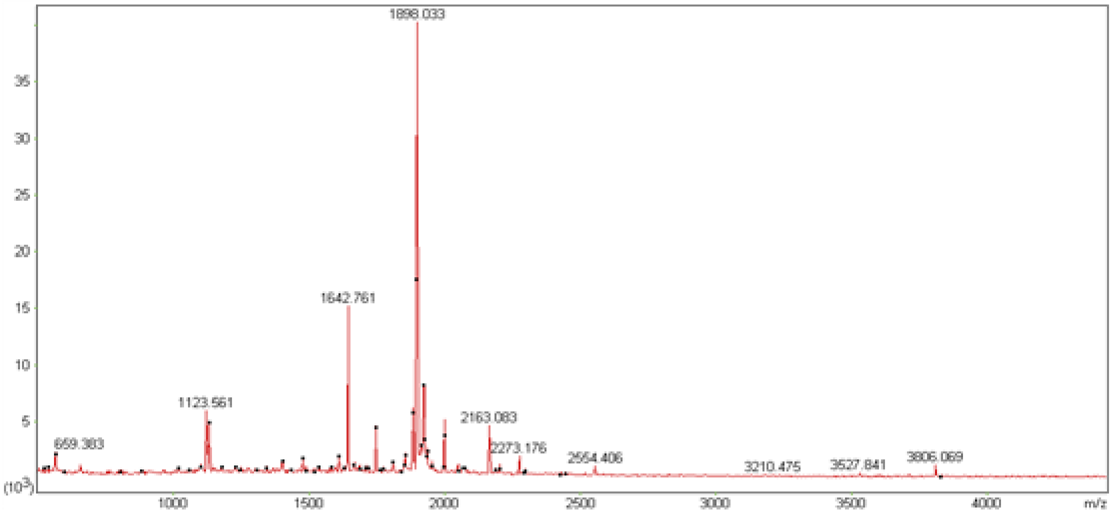

Probability Based Mowse Score:

Protein score is -10*Log(P), where P is the probability that the observed match is a random event.
Protein scores greater than 75 are significant (p<0.05).

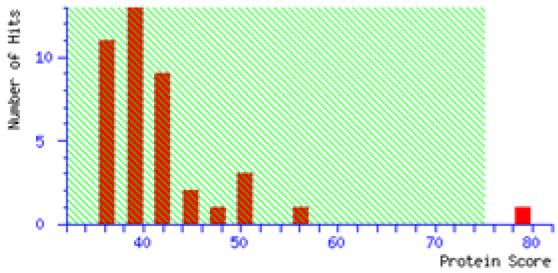

Matched peptide sequences: shown in **Bold Red**

```
  1 MAPTANTKQR RGRGAATTAD SDSDIEYQAY NGRLREASAA RARLKKAQEN
 51 RNQKRAALSA AHSAAIARVE ARLRSSIAKH HALRSAIILS HLNRLREALQ
101 RRDSILTQIA NKVAAQRRRM LNLGIQLSTL YEGRKEDIKG LIAELNGEKD
151 PDTEEQELIR G
```

Matched peptides information:

Start	-	End	Observed	Mr(expt)	Mr(calc)	ppm	M	Peptide
1	-	10	1133.5593	1132.5520	1132.5659	-12.2	1	-.MAPTANTKQR.R + Oxidation (M)
56	-	72	1664.7681	1663.7608	1663.9117	-90.7	1	R.AALSAAHSAAIARVEAR.L
85	-	94	1123.5611	1122.5538	1122.6509	-86.5	0	R.SAIILSHLNR.L
95	-	101	885.4245	884.4172	884.5192	-115	1	R.LREALQR.R
103	-	112	1102.7415	1101.7342	1101.6030	119	0	R.DSILTQIANK.V
119	-	134	1879.9300	1878.9227	1878.9985	-40.3	1	R.RMLNLGIQLSTLYEGR.K + Oxidation (M)
120	-	134	1707.8062	1706.7989	1706.9025	-60.7	0	R.MLNLGIQLSTLYEGR.K
120	-	135	1851.9429	1850.9356	1850.9924	-30.7	1	R.MLNLGIQLSTLYEGRK.E + Oxidation (M)
150	-	161	1401.7547	1400.7474	1400.6419	75.3	1	K.DPDTEEQELIRG.-

Spot No.: **58**

Mascot score: **77**

Species: ***Fusarium oxysporum f. sp. cubense race 1***

Protein name: **Alpha-galactosidase 2**

NCBI accession No.: **gi| 477514377** Sequence coverage %: **27**

Matched peptides No.: **15** Total peptides No.: **50**

Calculated Mr: **82979** Calculated *p*I: **5.13**

Annotated PMF **spectra:**

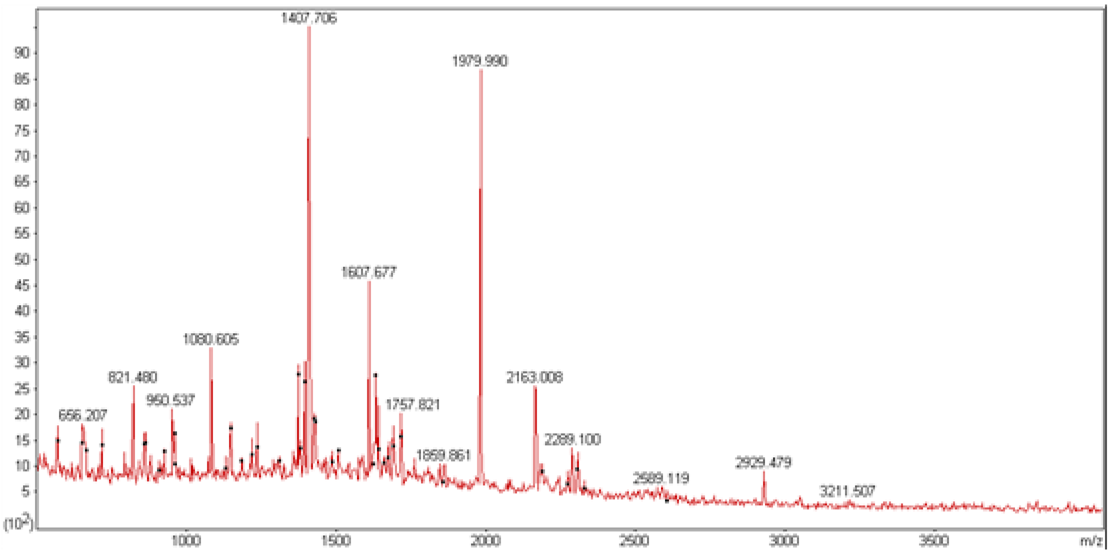

Probability Based Mowse Score:

Protein score is -10*Log(P), where P is the probability that the observed match is a random event.
Protein scores greater than 75 are significant (p<0.05).

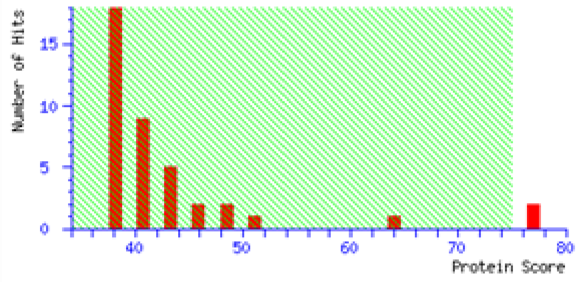

Matched peptide sequences: shown in **Bold Red**

1	MVLVTSKGIT	TAAVLFCQVI	SAFAESSDPI	R**VDGTSFALN**	**GDNVSYR**FHV
51	DNTTGDLIND	HYGGPVAEDG	ITTEIGPIQG	WVNLIGRVRR	**EFPDHGR**GDF
101	R**IPAFQLQQA**	**SGTTVTDFR**Y	KSHEVVQGKP	GLPGLPSTFG	EADDVSTLVV
151	HMYDNYSSIA	VDLSYSIFPK	YDAIVRSVNI	TNQGNATINL	R**KVSSWSVDL**
201	**QQDNLDLIEI**	**K**GDWAREGMR	VRRKVDFGTQ	GFQSSTGYSS	HLHNPFLALV
251	SSTTTETQGE	AWGFSLVYTG	SFAVDVEKSS	QGLTRAILGL	NPLDFSWPLK
301	AGQTFTTPEV	VSVFSSKGVG	GMSRQFHRLY	R**KHLMK**SKYA	EETRPVLLNS
351	WEGLAFDINE	TAIEKIAKQS	ADLGIKLFVM	DDGWFGNKYP	RVNDTAGLGD
401	WQPDKSR**FPD**	**GLTPLVENVT**	**DLK**VANSSDE	LKFGIWFEPE	MVNPESDLYD
451	**KHPDWAIHAG**	**SYPR**TETRNQ	LVLNLALPEV	QEFIIDFVSK	VLRESPISYV
501	KWDNNRGIHE	TPNPTLNYKY	MLGLYHVFET	LTSR**FPDVLW**	**EGCASGGGR**F
551	DPGVLQWFPQ	IWTSDDTDAV	ERIAIQFGTS	LAYPPSAMGA	HLSHVPNGNT
601	QRITSVKFR**A**	**HVAMMGGSFG**	**VELDPSDLEP**	**EER**EQIPGLI	ELSEK**INPIV**
651	**ITGDFYRLAL**	**PEETNYPAGQ**	**FISEDGKKVV**	**LFAFQTRATI**	**NNSWPWFR**LQ
701	GLDASAKYK**V**	**DNNHTVSGST**	**LMNLGIQLR**F	EGDYDSQVLM	IEKQ

Matched peptides information:

Start – End	Observed	Mr(expt)	Mr(calc)	ppm	M	Peptide
32 – 47	1714.7763	1713.7690	1713.7958	-15.6	0	R.VDGTSFALNGDNVSYR.F
91 – 97	857.4355	856.4283	856.3828	53.1	0	R.EFPDHGR.G
102 – 119	1979.9902	1978.9829	1979.0113	-14.3	0	R.IPAFQLQQASGTTVTDFR.Y
192 – 211	2330.0526	2329.0453	2329.2165	-73.5	1	R.KVSSWSVDLQQDNLDLIEIK.G
332 – 336	656.2073	655.2000	655.3839	-281	1	R.KHLMK.S
408 – 423	1757.8211	1756.8138	1756.9247	-63.1	0	R.FPDGLTPLVENVTDLK.V
452 – 464	1506.6105	1505.6032	1505.7164	-75.2	0	K.HPDWAIHAGSYPR.T
535 – 549	1607.6771	1606.6698	1606.7198	-31.1	0	R.FPDVLWEGCASGGGR.F
610 – 633	2589.1186	2588.1113	2588.1523	-15.8	0	R.AHVAMMGGSFGVELDPSDLEPEER.E + Oxidation (M)
610 – 633	2605.1522	2604.1449	2604.1472	-0.88	0	R.AHVAMMGGSFGVELDPSDLEPEER.E + 2 Oxidation (M)
646 – 657	1407.7059	1406.6986	1406.7558	-40.6	0	K.INPIVITGDFYR.L
658 – 678	2307.0835	2306.0762	2306.1430	-29.0	1	R.LALPEETNYPAGQFISEDGKK.V
679 – 687	1080.6052	1079.5979	1079.6128	-13.8	0	K.VVLFAFQTR.A
688 – 698	1391.6203	1390.6130	1390.6782	-46.9	0	R.ATINNSWPWFR.L
710 – 729	2184.9318	2183.9245	2184.0957	-78.4	0	K.VDNNHTVSGSTLMNLGIQLR.F + Oxidation (M)

Spot No.: **59**

NCBI accession No.: **gi|475664853**

Plant species: ***Fusarium oxysporum f. sp. cubense race 4***

Protein name： **Carboxypeptidase cpdS**

Mascot score: **62**　　Sequence coverage %: **2**

The number of matched peptides with p≤0.05: **1**

Calculated Mr: **57426**　　Calculated *Pi*: **6.71**

Annotated MS **spectra:**

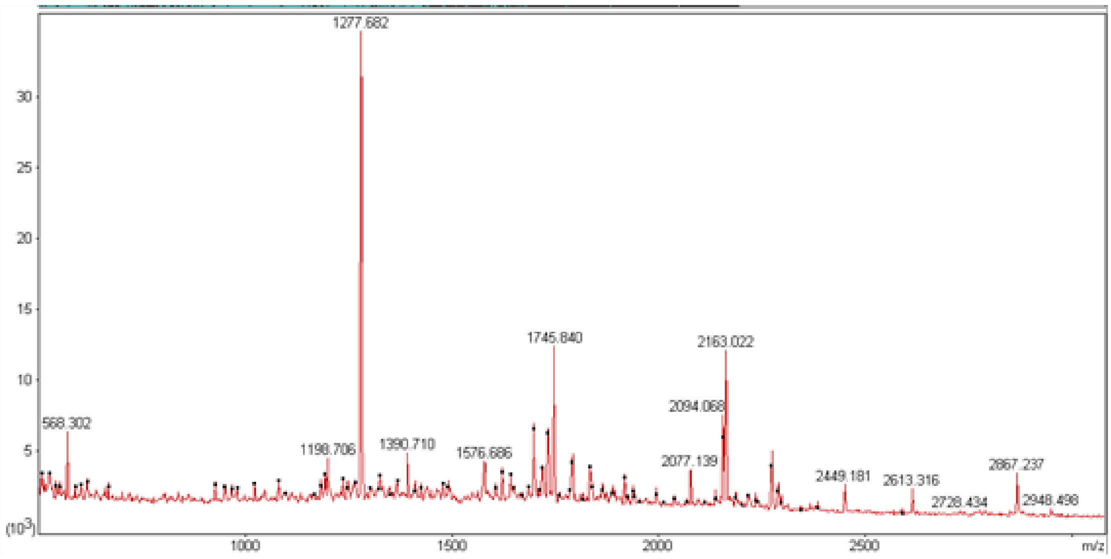

Probability Based Mowse Score:

Ions score is -10*Log(P), where P is the probability that the observed match is a random event.
Individual ions scores > 45 indicate peptides with significant homology.
Individual ions scores > 49 indicate identity or extensive homology (p<0.05).
Protein scores are derived from ions scores as a non-probabilistic basis for ranking protein hits.

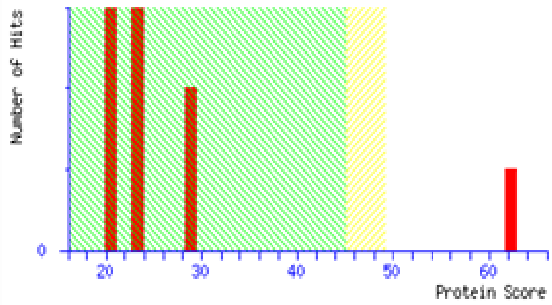

Matched peptide sequences: shown in Bold Red

```
  1 MGLKTALLSV LGFVALANAS VIHPRTSKPA HAQYYNKKTS PYYVPSTGIP
 51 EVSFDIGESY AGQIPVDWKK TGSKDPKFFY WFFPTVNPAG KDDVVIWFNG
101 GPGCSSLEGL TQENGPFSWK YGTYKPVPNA WSWHKLANVI WVEYPIGTGF
151 STGHVTAMNN TETAAQFVEW WKNLVDTFGL QGKKLYVTGE SYAGVYVPYV
201 GAAMLEKKDK TYYNVKGALY YDPVMPYADK LRLDHAAFPG FFRHWESVFA
251 IPDKNKKILD NDNEKCGLDR YREAHLTYPP PPAPWKPVQV KGCDITAHFD
301 EISTVINPCF NVYHVQDTCP VLWDVLGFPS VQYTPPGATL FFNIPGVRKA
351 IHAPAAPKEW ASCSGPVFVG DDDRYDPAEH ETKFQTLVEK TNNVMIGSGM
401 ADYIITSNTT ALAVQGLKWN GKQGFQTAPS AEFVVPVINN TESNVENWAG
451 GSVQGSVHSE RGFTLATVKT SGHMVPQYAP PAAFRQLEHI LGRVKSLTDA
501 EPFSVNISTS FKWPY
```

Matched peptides information:

Query	Start - End	Observed	Mr(expt)	Mr(calc)	ppm	M	Score	Expect	Rank	U	Peptide
1	233 - 243	1277.6820	1276.6747	1276.6353	30.9	0	62	0.0024	1	U	R.LDHAAFPGFFR.H

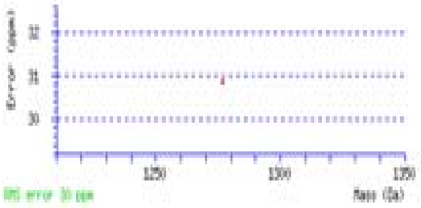

Annotated ion spectra of the matched peptides with p≤0.05:

CID No.: **59-1277.6**

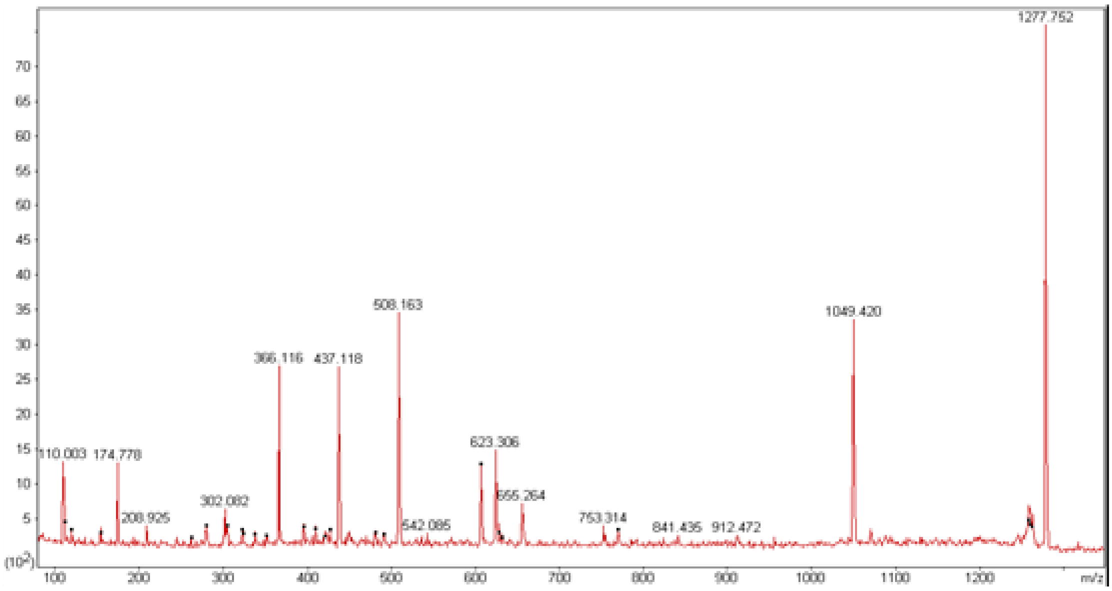

MS/MS Fragmentation of **R.LDHAAFPGFFR.H**

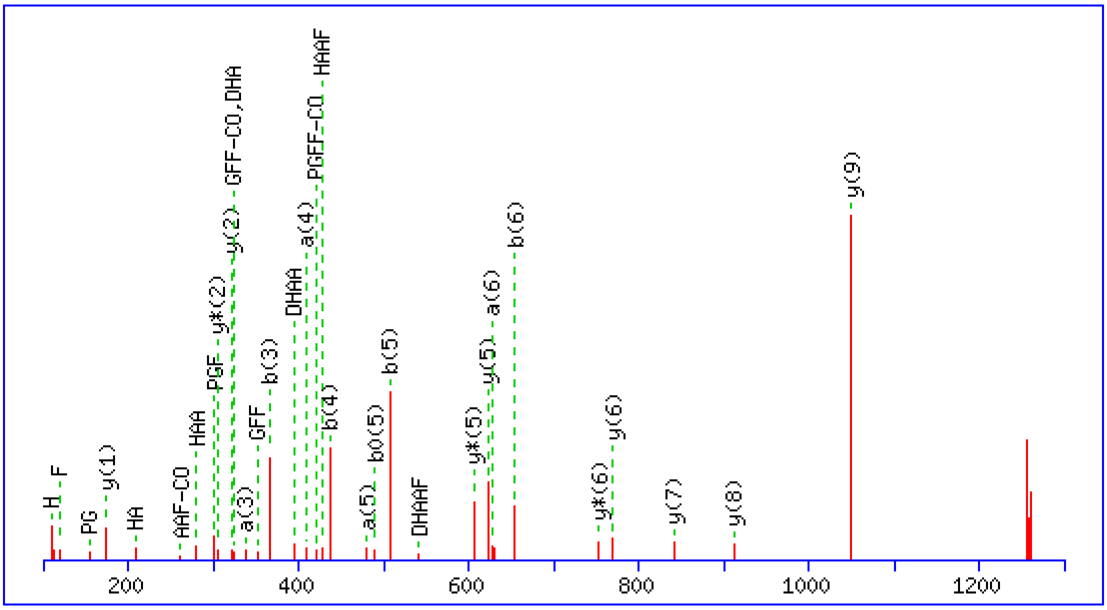

Monoisotopic mass of neutral peptide Mr(calc): 1276.6353

#	Immon.	a	a^0	b	b^0	d	Seq.	v	w	y	y*	y^0	#
1	86.0964	86.0964		114.0913		44.0495	L						11
2	88.0393	201.1234	183.1128	229.1183	211.1077	157.1335	D	1104.5374	1103.5421	1164.5585	1147.5320	1146.5479	10
3	110.0713	**338.1823**	320.1717	**366.1772**	348.1666		H	967.4785		***1049.5316***	1032.5050		9
4	44.0495	**409.2194**	391.2088	**437.2143**	419.2037		A	896.4414		***912.4727***	895.4461		8
5	44.0495	**480.2565**	462.2459	**508.2514**	490.2409		A	825.4042		***841.4355***	824.4090		7
6	120.0808	**627.3249**	609.3144	**655.3198**	637.3093		F	678.3358		***770.3984***	753.3719		6
7	70.0651	724.3777	706.3671	752.3726	734.3620	698.3620	P	581.2831	580.2878	***623.3300***	606.3035		5
8	30.0338	781.3992	763.3886	809.3941	791.3835		G			526.2772	509.2507		4
9	120.0808	928.4676	910.4570	956.4625	938.4519		F	377.1932		469.2558	452.2292		3
10	120.0808	1075.5360	1057.5254	1103.5309	1085.5203		F	230.1248		***322.1874***	305.1608		2
11	129.1135						R	74.0237	73.0284	***175.1190***	158.0924		1

Seq	ya	yb	Seq	ya	yb	Seq	ya	yb
DH	225.0982	253.0931	DHA	296.1353	324.1302	DHAA	367.1724	395.1674
DHAAF	514.2409	542.2358	DHAAFP	611.2936	639.2885	DHAAFPG	668.3151	696.3100
HA	181.1084	209.1033	HAA	252.1455	280.1404	HAAF	399.2139	427.2088
HAAFP	496.2667	524.2616	HAAFPG	553.2881	581.2831	AA	115.0866	143.0815
AAF	262.1550	290.1499	AAFP	359.2078	387.2027	AAFPG	416.2292	444.2241
AAFPGF	563.2976	591.2926	AF	191.1179	219.1128	AFP	288.1707	316.1656
AFPG	345.1921	373.1870	AFPGF	492.2605	520.2554	AFPGFF	639.3289	667.3239
FP	217.1335	245.1285	FPG	274.1550	302.1499	FPGF	421.2234	449.2183
FPGFF	568.2918	596.2867	PG	127.0866	155.0815	PGF	274.1550	302.1499
PGFF	421.2234	449.2183	GF	177.1022	205.0972	GFF	324.1707	352.1656
FF	267.1492	295.1441						

Spot No.: **60**

Mascot score: **93**

Species: ***Fusarium oxysporum f. sp. cubense race 4***

Protein name: **Chitinase 1**

NCBI accession No.: **gi| 475673339**

Sequence coverage %: **36**

Matched peptides No.: **16**

Total peptides No.: **66**

Calculated Mr: **47886**

Calculated *p*I: **6.31**

Annotated PMF **spectra:**

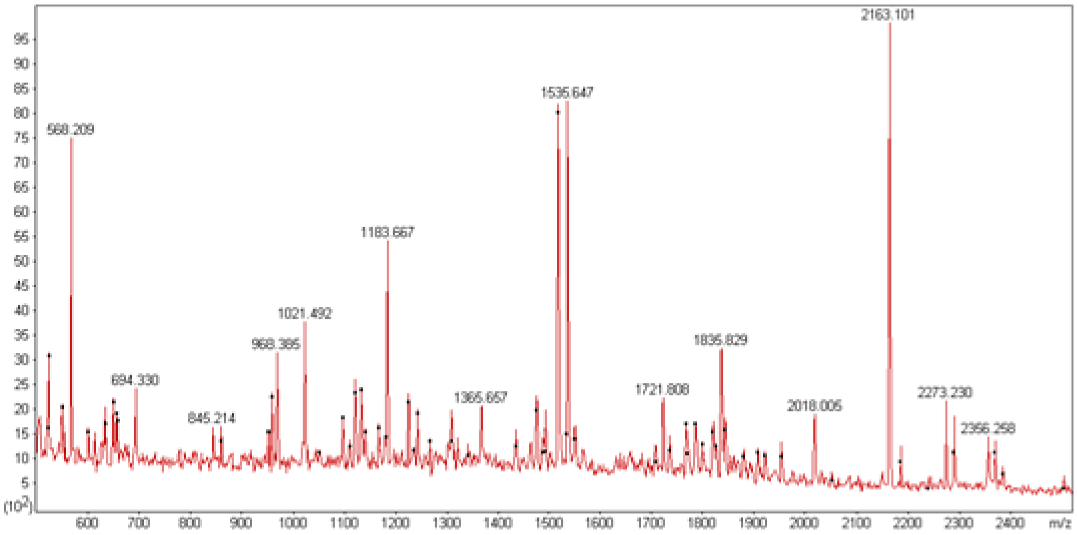

Probability Based Mowse Score:

Protein score is -10*Log(P), where P is the probability that the observed match is a random event.
Protein scores greater than 75 are significant (p<0.05).

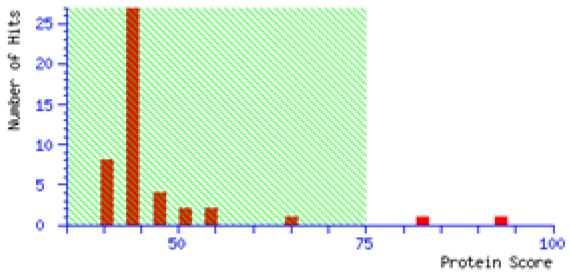

Matched peptide sequences: shown in Bold Red

```
  1 MRVSTLLGLS AYAVAEASCS RNIIYYDQWH TDDLPPKDVT HSVTHVMMSF
 51 ANSSLFTTEP SGKYEPFQPL KQVRALFDHD IKVCLAIGGW GDNAGFDAGL
101 KTDRSRERFA RNVASTLDRL GYDCVDIDME YPGGNGADYK QVVNSKKTYE
151 IQAFPKLLKE IKKFIGSKEL SIAVPGLERD MIAYVPSETP LIEKSVDFVN
201 VMTYDLMNRR DSYTTHHVSV KGAARAIDKY LSLGFPAHKL GIPFYAKWFT
251 TKQGYKCTNP IGCPTELLEN PKDGSDTGKS GSMTFEAANF VSAPTNLTTT
301 PDATCGAGTF FKCATGGCCA ASGWCGDTAA HCGTGCQSAY GHCDGIDLSA
351 SFHEALDKGK TDKANGGQWY WDAPNRIFWS WDTPELIAEK ISLLAKTRGV
401 KSVMAWALAL DSHDWSHLKA MQQGFDRVNA
```

Matched peptides information:

Start	-	End	Observed	Mr(expt)	Mr(calc)	ppm	M	Peptide
22	-	37	2018.0049	2016.9976	2016.9581	19.6	0	R.NIIYYDQWHTDDLPPK.D
64	-	71	1021.4915	1020.4842	1020.5280	-42.9	0	K.YEPFQPLK.Q
75	-	82	958.4709	957.4637	957.4920	-29.6	0	R.ALFDHDIK.V
83	-	101	1920.9716	1919.9643	1919.9200	23.1	0	K.VCLAIGGWGDNAGFDAGLK.T
102	-	106	634.2155	633.2082	633.3194	-176	1	K.TDRSR.E
147	-	156	1224.6042	1223.5969	1223.6550	-47.5	1	K.KTYEIQAFPK.L
148	-	156	1096.5087	1095.5014	1095.5600	-53.5	0	K.TYEIQAFPK.L
169	-	179	1183.6673	1182.6600	1182.6608	-0.67	0	K.ELSIAVPGLER.D
180	-	194	1721.8081	1720.8008	1720.8593	-34.0	0	R.DMIAYVPSETPLIEK.S + Oxidation (M)
195	-	209	1819.8527	1818.8454	1818.8281	9.54	0	K.SVDFVNVMTYDLMNR.R + Oxidation (M)
195	-	209	1835.8294	1834.8221	1834.8230	-0.47	0	K.SVDFVNVMTYDLMNR.R + 2 Oxidation (M)
230	-	239	1132.5596	1131.5523	1131.6077	-48.9	0	K.YLSLGFPAHK.L
257	-	272	1842.8576	1841.8503	1841.8652	-8.05	0	K.CTNPIGCPTELLENPK.D
377	-	390	1734.8698	1733.8625	1733.8665	-2.27	0	R.IFWSWDTPELIAEK.I
420	-	427	952.4206	951.4133	951.4232	-10.5	0	K.AMQQGFDR.V
420	-	427	968.3851	967.3778	967.4182	-41.7	0	K.AMQQGFDR.V + Oxidation (M)

Spot No.: **61**

Mascot score: **81**

Species: ***Podospora anserina S mat+***

Protein name: **hypothetical protein**

NCBI accession No.: **gi| 171680085**

Sequence coverage %: **34**

Matched peptides No.: **10**

Total peptides No.: **42**

Calculated Mr: **41333**

Calculated *p*I: **5.61**

Annotated PMF spectra:

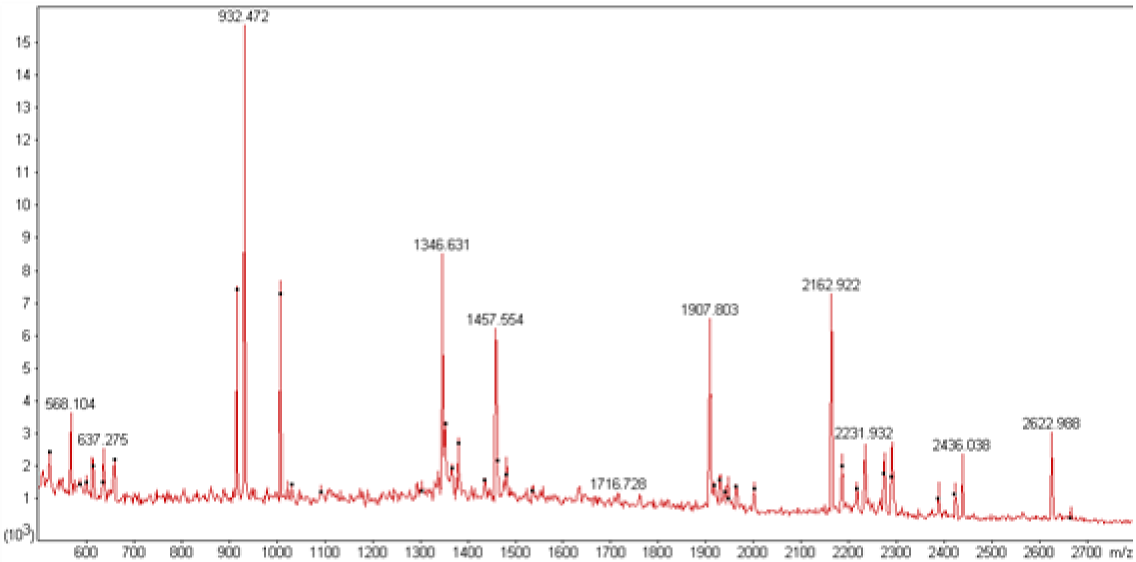

Probability Based Mowse Score:

Protein score is -10*Log(P), where P is the probability that the observed match is a random event.
Protein scores greater than 75 are significant (p<0.05).

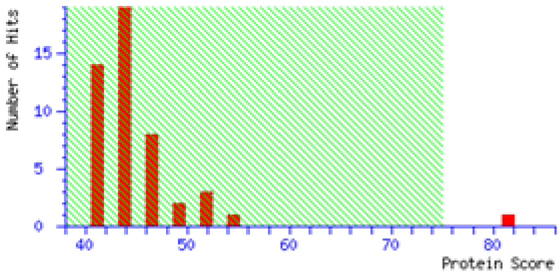

Matched peptide sequences: shown in **Bold Red**

```
  1 TLALLDYYHI PFILLTNGGG KFEADRVAEL NEKLGSHMTT ENFCQSHTPF
 51 QELLPVYRDK TILVTGSDYE KCREIMEGYG FRSVVTPGDI FRAAPEVFPF
101 DTVRGKVGRD LPKPIWRPRK EAGEHHQGSK GMVRDTEQEE REGKLEDHLK
151 VEAMFVLNDP RDWALDVQVF IDLLQSKQGY VGTYSEENNK GRWQGDGQPK
201 LFFSNSDLIW AAKYHLPRFG QGAFQHALVG IWNEVTEGKK ELVRTSFGKP
251 HRETYEYAEE MLVRHRGGWL RAKGYKEGEI EGGLKRVYMV GDNPESDIAG
301 ANDYDGKGKY GTEWVSLLVE TGVFDATRMN FKDGDVRKAD VVKPNVAEAV
351 KWALRNEGWV DE
```

Matched peptides information:

Start	–	End	Observed	Mr(expt)	Mr(calc)	ppm	M	Peptide
22	–	26	637.2748	636.2675	636.2867	-30.2	0	K.FEADR.V
72	–	82	1433.6489	1432.6416	1432.6227	13.2	1	K.CREIMEGYGFR.S + Oxidation (M)
83	–	92	1090.3986	1089.3913	1089.5819	-175	0	R.SVVTPGDIFR.A
83	–	104	2420.0623	2419.0550	2419.2536	-82.1	1	R.SVVTPGDIFRAAPEVFPFDTVR.G
93	–	106	1533.6514	1532.6441	1532.7987	-101	1	R.AAPEVFPFDTVRGK.V
131	–	141	1365.5978	1364.5905	1364.5990	-6.23	1	K.GMVRDTEQEER.E + Oxidation (M)
193	–	200	915.4566	914.4493	914.4246	27.0	0	R.WQGDGQPK.L
219	–	239	2287.9604	2286.9531	2287.1386	-81.1	0	R.FGQGAFQHALVGIWNEVTEGK.K
286	–	307	2386.0714	2385.0641	2385.0543	4.12	1	K.RVYMVGDNPESDIAGANDYDGK.G
310	–	332	2662.9905	2661.9832	2662.3101	-123	1	K.YGTEWVSLLVETGVFDATRMNFK.D

Spot No.: **62**

Mascot score: **83**

Species: ***Fusarium oxysporum***

Protein name: **predicted GMC oxidoreductase**

NCBI accession No.: **gi| 283806340** Sequence coverage %: **27**

Matched peptides No.: **9** Total peptides No.: **24**

Calculated Mr: **71355** Calculated *p*I: **5.20**

Annotated PMF **spectra:**

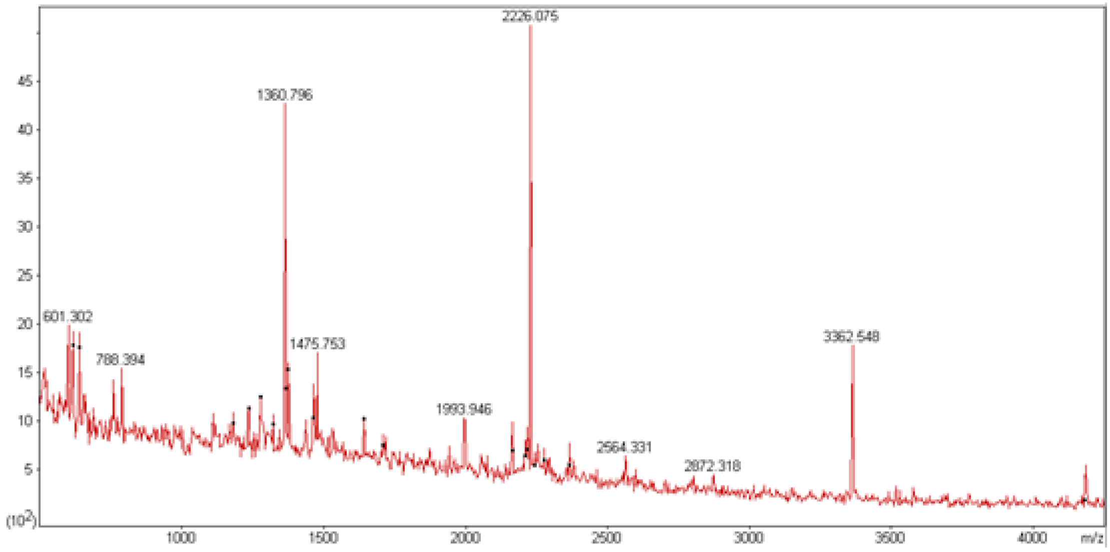

Probability Based Mowse Score:

Protein score is -10*Log(P), where P is the probability that the observed match is a random event. Protein scores greater than 75 are significant (p<0.05).

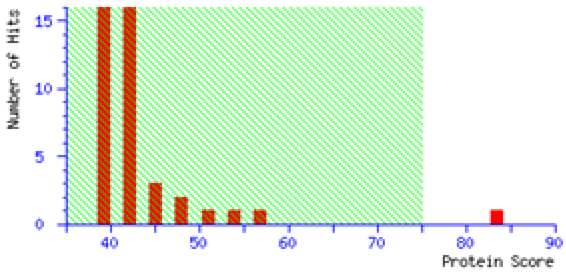

Matched peptide sequences: shown in **Bold Red**

1 MRCNVLSPSL LALSLSTSVT ARPEIPFIST LLDPLQHIVK SAYAGDGLK**V**
51 **GHLGELGGIP GVDAEYDYVV VGGGTAGNTI GYR**LAK**AGYS VAIVEAGPFL**
101 **EISKPILATS PGGVTAGSGA SLLDSNPLR**D WRFTTTAQTG ADDR**EIHYAR**
151 GMVLGGSSQL NFLVYHRPTN GTMAKWAEDV GDDSYTWEQM LPHFQK**SPSF**
201 **TPPDNEKR**NV TTDYDASAFS KEKGPLQISY ANYVPSWAVL VEKGLKSLGF
251 KGIDGFSSGS LLGYSYTTTT VQPKTATRSA SDDFVKTARS EKLKTLKVYT
301 ESLAKKVTFD DDKK**ATGVEV SSVGIDYTLK** AKKEVIVSGG AFQSPQVLMV
351 SGVGPKQHLE ELDIPVVADL PGVGQNLWDH VLFGPSFEVQ GLEDTLSAAI
401 NNDTVFQGAL DAYAQQQAGP LTSNQVELLG WEKAPEKYRS KFSAETIKDL
451 ATFPDDWPEM EIIPLNVYSE DWSFPILQQP TDGKKYTSLN GALVAPLSRG
501 NITLRTNSTT DAPLINPNFL TK**KADQEVAI ALFR**RLREIA KSTPLKDTVL
551 **KEVYPGEQHE TDEQILAVLR** DTLMTVWHAA CTCKMGKK**DD EMAVLDSKAR**
601 VYGVKGLR**VV DASAFPLLIP GHPVGTVYAL AEK**IAHDIIT EEKSEGSAYK
651 VDIDVEVS

Matched peptides information:

Start - End	Observed	Mr(expt)	Mr(calc)	ppm	M	Peptide
50 - 83	3362.5479	3361.5406	3361.6579	-34.9	0	K.VGHLGELGGIPGVDAEYDYVVVGGGTAGNTIGYR.L
87 - 129	4183.0748	4182.0675	4182.2212	-36.8	0	K.AGYSVAIVEAGPFLEISKPILATSPGGVTAGSGASLLDSNPLR.D
145 - 150	788.3944	787.3871	787.3977	-13.4	0	R.EIHYAR.G
197 - 208	1374.6924	1373.6851	1373.6575	20.1	1	K.SPSFTPPDNEKR.N
315 - 330	1638.8490	1637.8417	1637.8512	-5.80	0	K.ATGVEVSSVGIDYTLK.A
523 - 534	1360.7964	1359.7891	1359.7510	28.0	1	K.KADQEVAIALFR.R
552 - 570	2226.0751	2225.0678	2225.0964	-12.8	0	K.EVYPGEQHETDEQILAVLR.D
589 - 600	1365.6718	1364.6645	1364.6242	29.6	1	K.DDEMAVLDSKAR.V + Oxidation (M)
609 - 633	2564.3314	2563.3241	2563.4050	-31.6	0	R.VVDASAFPLLIPGHPVGTVYALAEK.I

Spot No.: **63**

Mascot score: **134**

Species: ***Fusarium oxysporum f. sp. cubense race 4***

Protein name: **O-acetylhomoserine (thiol)-lyase**

NCBI accession No.: **gi| 475675629**

Sequence coverage %: **57**

Matched peptides No.: **15**

Total peptides No.: **35**

Calculated Mr: **47110**

Calculated *p*I: **5.89**

Annotated PMF **spectra:**

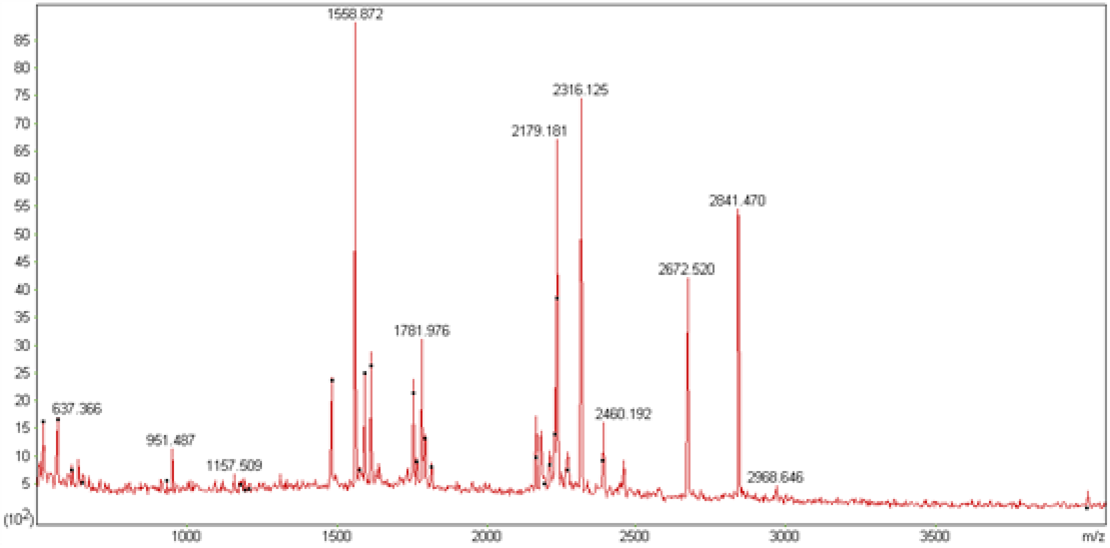

Probability Based Mowse Score:

Protein score is -10*Log(P), where P is the probability that the observed match is a random event.
Protein scores greater than 75 are significant (p<0.05).

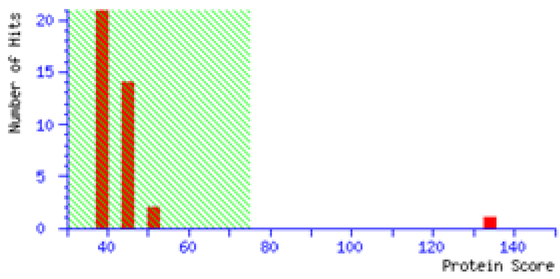

Matched peptide sequences: shown in Bold Red

```
  1 MAEQVFQNFE TLQLHAGYTP DPHTRSTAVP IYATSSYTFN DSAHGARLFG
 51 LKELGNIYSR LMNPTVDVFE KRIAALEGGI AAAATSSGQA AQFLTIATLA
101 KAGDNIVASS HLYGGTYNQL NVLLPRFGIK TKFVRSGKLE DYAAAIDDQT
151 RAIYVESMSN PDYVVPDFEG IAKIAHEHGI PLVVDNTLGA GGYYVRPIEH
201 GADIVVHSAT KWIGGHGTTI GGVIVDSGRF NWNKHSDRFP EMVEPSPSYH
251 GLKYWEAFGP ATFITRIRVE MLRDIGACLS PFSAQQLLLG IETLGLRAER
301 HAQNTEKLAK YFESSPNVSW VLWPGSESHP TYAQAKKYLT RGFGAMLSIG
351 VKGDASAGSK VVDGLKLVSN LANVGDAKSL AIHPWSTTHE QLSEDERLAS
401 GVTEDMIRIS VGIEHVDDII ADFEQSFQKA YGS
```

Matched peptides information:

Start - End	Observed	Mr(expt)	Mr(calc)	ppm	M	Peptide
26 - 47	2316.1253	2315.1180	2315.0818	15.6	0	R.STAVPIYATSSYTFNDSAHGAR.L
53 - 60	951.4869	950.4796	950.4821	-2.62	0	K.ELGNIYSR.L
102 - 126	2672.5203	2671.5130	2671.3718	52.9	0	K.AGDNIVASSHLYGGTYNQLNVLLPR.F
139 - 151	1480.7845	1479.7772	1479.6841	62.9	0	K.LEDYAAAIDDQTR.A
152 - 173	2460.1916	2459.1843	2459.1566	11.3	0	R.AIYVESMSNPDYVVPDFEGIAK.I + Oxidation (M)
174 - 211	4005.9917	4004.9844	4005.0861	-25.4	0	K.IAHEHGIPLVVDNTLGAGGYYVRPIEHGADIVVHSATK.W
212 - 229	1781.9760	1780.9687	1780.9221	26.2	0	K.WIGGHGTTIGGVIVDSGR.F
235 - 253	2213.0963	2212.0890	2212.0371	23.5	1	K.HSDRFPEMVEPSPSYHGLK.Y
235 - 253	2229.0015	2227.9942	2228.0320	-17.0	1	K.HSDRFPEMVEPSPSYHGLK.Y + Oxidation (M)
254 - 266	1558.8718	1557.8645	1557.7616	66.1	0	K.YWEAFGPATFITR.I
342 - 360	1752.8887	1751.8814	1751.8876	-3.54	1	R.GFGAMLSIGVKGDASAGSK.V
379 - 397	2236.1104	2235.1031	2235.0556	21.3	0	K.SLAIHPWSTTHEQLSEDER.L
398 - 408	1191.5454	1190.5381	1190.5965	-49.0	0	R.LASGVTEDMIR.I
398 - 408	1207.6235	1206.6162	1206.5914	20.5	0	R.LASGVTEDMIR.I + Oxidation (M)
409 - 429	2390.2758	2389.2685	2389.1802	37.0	0	R.ISVGIEHVDDIIADFEQSFQK.A

Spot No.: **64**

Mascot score: **75**

Species: ***Fusarium oxysporum***

Protein name: **lactonohydrolase**

NCBI accession No.: **gi| 3810873**

Sequence coverage %: **31**

Matched peptides No.: **11**

Total peptides No.: **58**

Calculated Mr: **43237**

Calculated *p*I: **5.10**

Annotated PMF **spectra:**

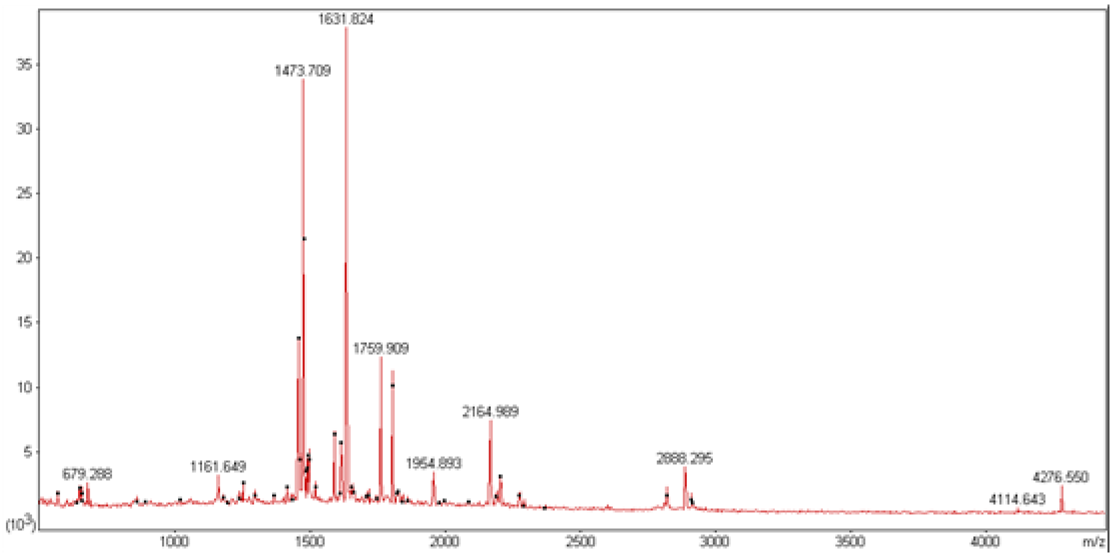

Probability Based Mowse Score:

Protein score is -10*Log(P), where P is the probability that the observed match is a random event.
Protein scores greater than 75 are significant (p<0.05).

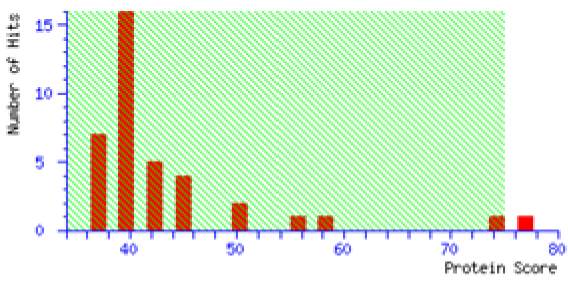

Matched peptide sequences: shown in Bold Red

1	MPSSISVLAG	VLVPVLGAVA	AKLPSTAQII	DQKSFNVLKD	VPPPAVANDS
51	LVFTWPGVTE	ESLVEKPFHV	YDEEFYDVIG	KDPSLTLIAT	SDTDPIFHEA
101	VVWYPPTEEV	FFVQNAGAPA	AGTGLNKSSI	IQKISLKEAD	AVRKGKQDEV
151	K**VTVVDSNPQ**	**VINPNGGTYY**	**K**GNIIFAGEG	QGDDVPSALY	LMNPLPPYNT
201	TTLLNNYFGR	**QFNSLNDVGI**	**NPR**NGDLYFT	DTLYGYLQDF	RPVPGLR**NQV**
251	**YR**YNFDTGAV	TVVADDFTLP	NGIGFGPDGK	**KVYVTDTGIA**	**LGFYGR**NLSS
301	PASVYSFDVN	QDGTLQNR**KT**	**FAYVASFIPD**	**GVHTDSK**GR**V**	**YAGCGDGVHV**
351	**WNPSGK**LIGK	**IYTGTVAANF**	**QFAGK**GR**MII**	**TGQTKLFYVT**	**LGASGPK**LYD

Matched peptides information:

Start	–	End	Observed	Mr(expt)	Mr(calc)	ppm	M	Peptide
152	–	171	2164.9885	2163.9812	2164.0801	-45.7	0	K.VTVVDSNPQVINPNGGTYYK.G
211	–	223	1473.7092	1472.7019	1472.7372	-23.9	0	R.QFNSLNDVGINPR.N
248	–	252	679.2879	678.2806	678.3449	-94.8	0	R.NQVYR.Y
281	–	296	1759.9087	1758.9014	1758.9305	-16.5	1	K.KVYVTDTGIALGFYGR.N
282	–	296	1631.8239	1630.8166	1630.8355	-11.6	0	K.VYVTDTGIALGFYGR.N
319	–	337	2082.9745	2081.9672	2082.0422	-36.0	1	R.KTFAYVASFIPDGVHTDSK.G
320	–	337	1954.8934	1953.8861	1953.9473	-31.3	0	K.TFAYVASFIPDGVHTDSK.G
340	–	356	1802.7742	1801.7669	1801.8206	-29.8	0	R.VYAGCGDGVHVWNPSGK.L
361	–	375	1587.7483	1586.7410	1586.8093	-43.0	0	K.IYTGTVAANFQFAGK.G
378	–	385	891.4544	890.4471	890.4895	-47.6	0	R.MIITGQTK.L
386	–	397	1252.5909	1251.5836	1251.6863	-82.1	0	K.LFYVTLGASGPK.L

Spot No.: **65**

Mascot score: **126**

Species: ***Fusarium oxysporum f. sp. cubense race 4***

Protein name: **Catalase-peroxidase**

NCBI accession No.: **gi| 475671755**

Sequence coverage %: **39**

Matched peptides No.: **15**

Total peptides No.: **51**

Calculated Mr: **73292**

Calculated *p*I: **5.98**

Annotated PMF **spectra:**

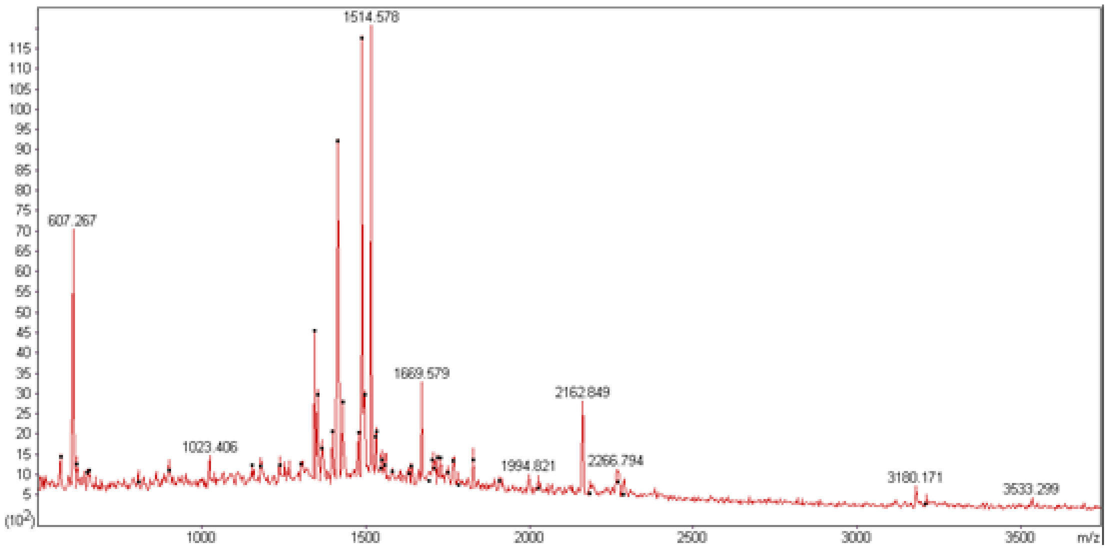

Probability Based Mowse Score:

Protein score is -10*Log(P), where P is the probability that the observed match is a random event.
Protein scores greater than 75 are significant (p<0.05).

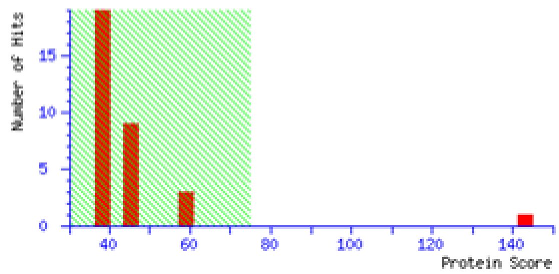

Matched peptide sequences: shown in **Bold Red**

```
  1 MSWHAVGTYR IFDGRGGGRQ GQQRFAPLNS WPDNVSLDKA RRLLWPVKQK
 51 YGNKVSWADL IVMAGNVALE DMGFKTIGFA AGRPDTWEAD EATYYGGEDT
101 WLGNDVRYSD GHPGTTKPGA TDSDQAPHKN IHTRELEKPL AAAHHGLIYV
151 NPEGPDGNPD PVAAARDIRE TFGRMAMNDE ETVALIAGGH TVGKTHGAGS
201 TDHVGPEPEA ADLAQQGLGW SNSYKTGKGP HTTTSGLEVT WTSTPVKWSH
251 DYLKYLFQFE WELTKSPAGA HQWVAKDANA TVPDAFDPNK KQKPTMLTTD
301 LSLRFDPEYE KISRRFLENP DQFNEAFAKA WFKLTHRDMG PRDRYLGPEV
351 PKEVFLWQDP VPERDYKLVD DGDISAIKNE ILKSGVDVSK LVSTAWASAS
401 TFRGSDLRGG ANGARIRLQP QKDWEVNNPA QLSKVLSTLE GIQKKFNDSQ
451 SSGKAISLAD VIVLAGSAAV EKAAKDAGVN ITVPFAPGRT DATQEQTDVK
501 SVNHLQPFAD GFRNYGSSTD RVKLEHQLID RAQLLTLSVP ELTALIGGLR
551 ALNTNYDGSS HGIFTNRPGV LTNDFFVNLL DMSTEWKAVG NGDIFEGTDR
601 KTGAKKWTGT RVDLVFGSHA ELRATAETYA EAGGQEKLVK DFVAAWTKVM
651 NLDRFDLANG SSPKASPRL
```

Matched peptides information:

Start	–	End	Observed	Mr(expt)	Mr(calc)	ppm	M	Peptide
11	–	15	607.2666	606.2593	606.3126	-87.8	0	R.IFDGR.G
20	–	24	616.2804	615.2731	615.3089	-58.1	0	R.QGQQR.F
25	–	39	1702.6239	1701.6166	1701.8362	-129	0	R.FAPLNSWPDNVSLDK.A
76	–	107	3533.2993	3532.2920	3532.5808	-81.7	0	K.TIGFAAGRPDTWEADEATYYGGEDTWLGNDVR.Y
108	–	129	2266.7940	2265.7867	2266.0251	-105	0	R.YSDGHPGTTKPGATDSDQAPHK.N
195	–	225	3180.1714	3179.1641	3179.4544	-91.3	0	K.THGAGSTDHVGPEPEAADLAQQGLGWSNSYK.T
266	–	276	1151.3901	1150.3828	1150.5883	-179	0	K.SPAGAHQWVAK.D
315	–	329	1825.6899	1824.6826	1824.8795	-108	1	R.RFLENPDQFNEAFAK.A
316	–	329	1669.5787	1668.5714	1668.7784	-124	0	R.FLENPDQFNEAFAK.A
353	–	364	1514.5778	1513.5705	1513.7565	-123	0	K.EVFLWQDPVPER.D
391	–	403	1396.5324	1395.5251	1395.7147	-136	0	K.LVSTAWASASTFR.G
418	–	434	1994.8207	1993.8134	1994.0221	-105	1	R.LQPQKDWEVNNPAQLSK.V
455	–	472	1726.7163	1725.7090	1725.9876	-161	0	K.AISLADVIVLAGSAAVEK.A
476	–	489	1413.5334	1412.5261	1412.7412	-152	0	K.DAGVNITVPFAPGR.T
490	–	500	1235.4230	1234.4157	1234.5677	-123	0	R.TDATQEQTDVK.S
501	–	513	1487.5296	1486.5223	1486.7317	-141	0	K.SVNHLQPFADGFR.N
514	–	521	899.2688	898.2615	898.3781	-130	0	R.NYGSSTDR.V
524	–	531	1023.4065	1022.3992	1022.5509	-148	0	K.LEHQLIDR.A
612	–	623	1342.5117	1341.5044	1341.7041	-149	0	R.VDLVFGSHAELR.A

Spot No.: **66**

Mascot score: **119**

Species: ***Fusarium oxysporum Fo5176***

Protein name: **DEAD/DEAH box helicase**

NCBI accession No.: **gi| 342890194**

Sequence coverage %: **55**

Matched peptides No.: **21**

Total peptides No.: **68**

Calculated Mr: **44984**

Calculated *p*I: **4.98**

Annotated PMF **spectra:**

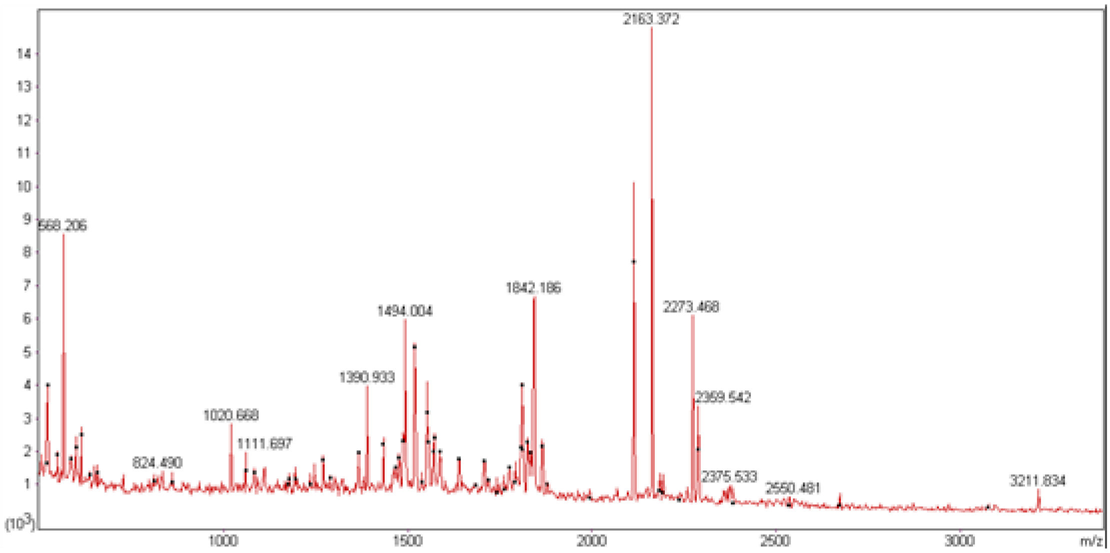

Probability Based Mowse Score:

Protein score is -10*Log(P), where P is the probability that the observed match is a random event.
Protein scores greater than 75 are significant (p<0.05).

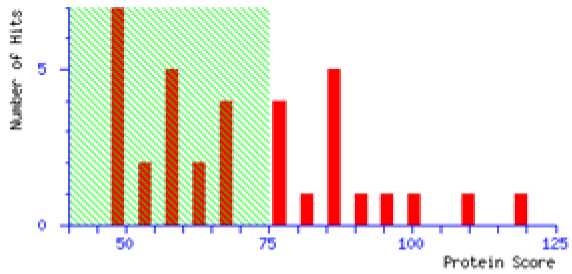

Matched peptide sequences: shown in Bold Red

```
  1 MADKGLEDVP EGQIESNYDE TVDSFDDMNL KSELLRGVYA YGFERPSAIQ
 51 QRAIMPVIKG HDVIAQAQSG TGKTATFSIS VLQKIDTNVK QCQALILAPT
101 RELAQQIQKV VVAIGDFMQI ECHACIGGTS VREDMKALQD GPQVVVGTPG
151 RVQDMIQRRF LKTDSMKMFV LDEADEMLSR GFTEQIYDIF QLLPQSTQVV
201 LLSATMPQDV LEVTTKFMRD PVRILVKKDE LTLEGIKQFY IAVEKEEWKL
251 DTLSDLYETV TITQAVIFCN TRRKVDWLTD KLTARDFTVS AMHGDMDQAQ
301 RDLIMKEFRS GSSRVLIATD LLARGIDVQQ VSLVINYDLP ANRENYIHRI
351 GRGGRFGRKG VAINFVTAED VRMMREIEQF YSTQIEEMPM NVADLI
```

Matched peptides information:

Start - End	Observed	Mr(expt)	Mr(calc)	ppm	M	Peptide
5 - 31	3075.6342	3074.6269	3074.3186	100	0	K.GLEDVPEGQIESNYDETVDSFDDMNLK.S + Oxidation (M)
37 - 52	1842.1860	1841.1787	1840.9220	139	0	R.GVYAYGFERPSAIQQR.A
74 - 84	1194.7873	1193.7800	1193.6656	95.9	0	K.TATFSISVLQK.I
74 - 90	1865.1717	1864.1644	1864.0306	71.8	1	K.TATFSISVLQKIDTNVK.Q
91 - 101	1270.8607	1269.8534	1269.6863	132	0	K.QCQALILAPTR.E
110 - 132	2535.5519	2534.5446	2534.2080	133	0	K.VVVAIGDFMQIECHACIGGTSVR.E + Oxidation (M)
133 - 136	522.2575	521.2502	521.2155	66.5	0	R.EDMK.A
137 - 151	1494.0043	1492.9970	1492.7998	132	0	K.ALQDGPQVVVGTPGR.V
152 - 159	1061.6510	1060.6437	1060.5448	93.3	1	R.VQDMIQRR.F + Oxidation (M)
168 - 180	1556.0257	1555.0184	1554.7058	201	0	K.MFVLDEADEMLSR.G
168 - 180	1571.9530	1570.9457	1570.7007	156	0	K.MFVLDEADEMLSR.G + Oxidation (M)
168 - 180	1587.9343	1586.9270	1586.6956	146	0	K.MFVLDEADEMLSR.G + 2 Oxidation (M)
238 - 249	1570.0028	1568.9955	1568.7875	133	1	K.QFYIAVEKEEWK.L
250 - 272	2673.7022	2672.6949	2672.3367	134	0	K.LDTLSDLYETVTITQAVIFCNTR.R
315 - 324	1084.7952	1083.7879	1083.6652	113	0	R.VLIATDLLAR.G
325 - 343	2114.4375	2113.4302	2113.1168	148	0	R.GIDVQQVSLVINYDLPANR.E
350 - 355	615.3811	614.3738	614.3612	20.5	1	R.IGRGGR.F
359 - 372	1519.0417	1518.0344	1517.8202	141	1	R.KGVAINFVTAEDVR.M
360 - 372	1390.9326	1389.9253	1389.7252	144	0	K.GVAINFVTAEDVR.M
360 - 375	1809.1141	1808.1068	1807.9073	110	1	K.GVAINFVTAEDVRMMR.E
360 - 375	1825.0638	1824.0565	1823.9022	84.6	1	K.GVAINFVTAEDVRMMR.E + Oxidation (M)

Spot No.: **67**

Mascot score: **80**

Species: ***Candida parapsilosis***

Protein name: **hypothetical protein CPAR2_601850**

NCBI accession No.: **gi| 354543047** Sequence coverage %: **38**

Matched peptides No.: **9** Total peptides No.: **80**

Calculated Mr: **23020** Calculated *p*I: **10.12**

Annotated PMF **spectra:**

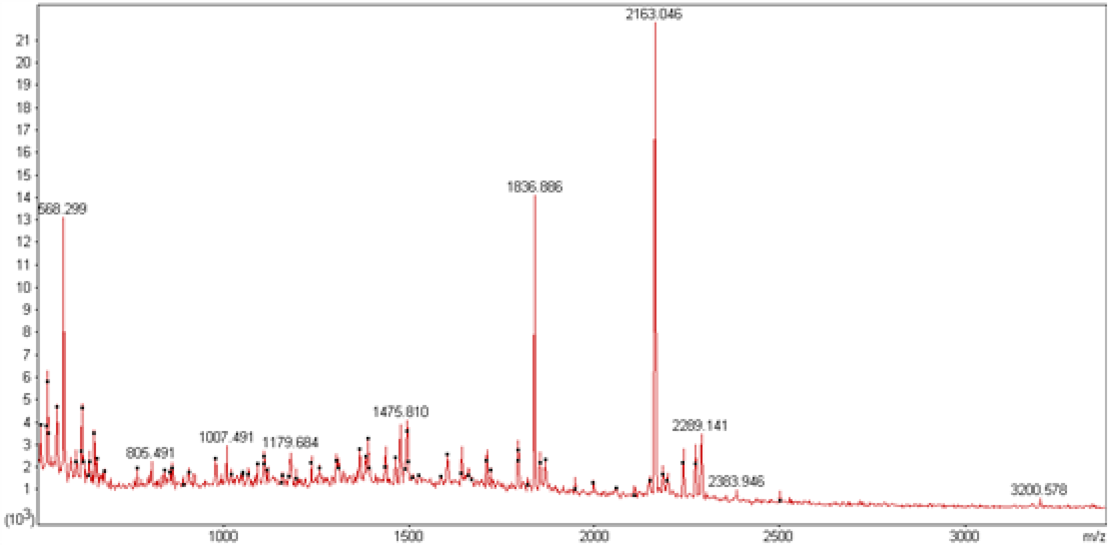

Probability Based Mowse Score:

Protein score is -10*Log(P), where P is the probability that the observed match is a random event.
Protein scores greater than 75 are significant (p<0.05).

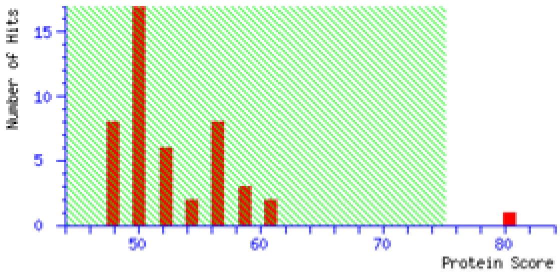

Matched peptide sequences: shown in Bold Red

1 **MSSTPSPTKR** SALSPK**VFNV** **NQLSPQR**K**NL** **GLSR**RHTNKS PIRSSTEVRT
51 PSPK**KPKPAT** **TLGFTIWEDN** **VDTR**SHEIVG TPTSNELNHN DQENILQPKS
101 TYSRRVHGSP LRDLSINSFK GYITSNGVTT QLEELYQPIN FENEFKSAHR
151 FNGLPPFVTP TKK**DKYLVKS** **GQCADGSVGK** HSR**SFSAGIN** **EAKR**DLVQKP
201 **RFAISS**

Matched peptides information:

Start	-	End	Observed	Mr(expt)	Mr(calc)	ppm	M	Peptide
1	-	10	1107.5959	1106.5886	1106.5390	44.8	1	-.MSSTPSPTKR.S + Oxidation (M)
17	-	27	1301.6968	1300.6895	1300.6888	0.57	0	K.VFNVNQLSPQR.K
29	-	34	659.4844	658.4772	658.3762	153	0	K.NLGLSR.R
55	-	74	2289.1414	2288.1341	2288.1801	-20.1	0	K.KPKPATTLGFTIWEDNVDTR.S
164	-	169	765.4716	764.4643	764.4432	27.6	1	K.DKYLVK.S
166	-	169	522.3586	521.3513	521.3213	57.6	0	K.YLVK.S
170	-	180	1065.6342	1064.6269	1064.4557	161	0	K.SGQCADGSVGK.H
184	-	194	1179.6836	1178.6763	1178.6044	61.1	1	R.SFSAGINEAKR.D
202	-	206	524.3430	523.3358	523.2642	137	0	R.FAISS.-

Spot No.: **68**

Mascot score: **144**

Species: ***Fusarium oxysporum f. sp. cubense race 4***

Protein name: **1,3-beta-glucanosyltransferase gel4**

NCBI accession No.: **gi| 475668458**

Sequence coverage %: **52**

Matched peptides No.: **26**

Total peptides No.: **86**

Calculated Mr: **59159**

Calculated *p*I: **4.83**

Annotated PMF **spectra:**

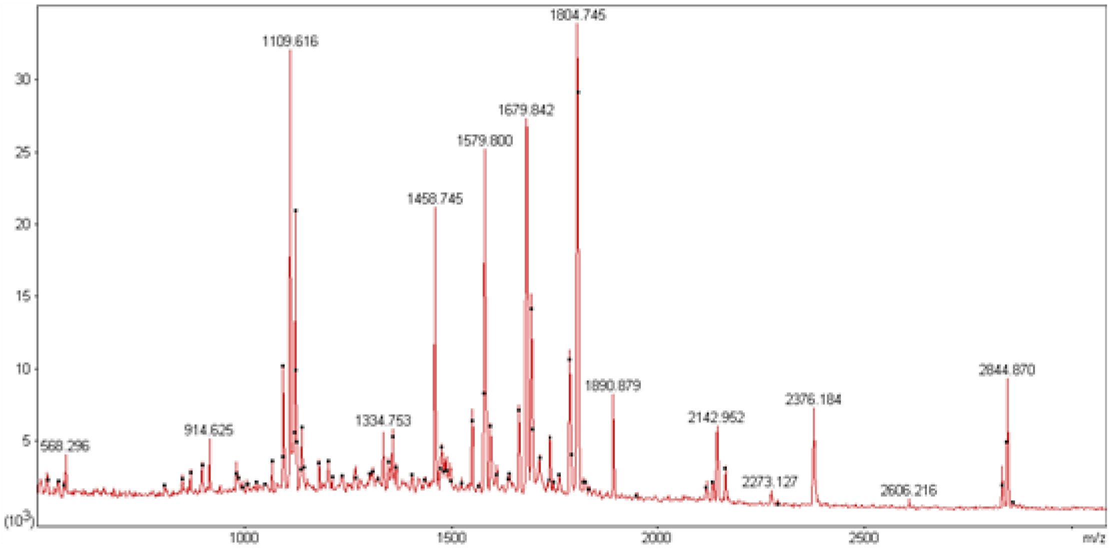

Probability Based Mowse Score:

Protein score is -10*Log(P), where P is the probability that the observed match is a random event.
Protein scores greater than 75 are significant (p<0.05).

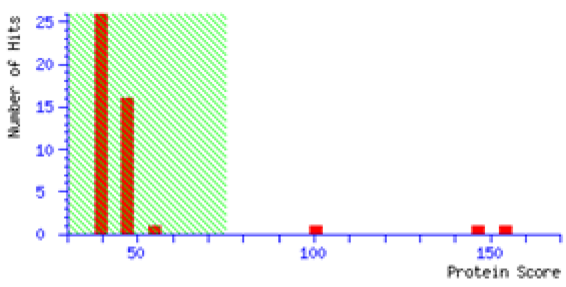

Matched peptide sequences: shown in Bold Red

```
  1  MKFSAAIVAA AATAASAKLE PITMKGSKLF YSNGTQFFMK GVAYQQDTAA
 51  AGETNDKTTK YIDPLADEDA CKRDIPLLKQ LGTNIIRTYA INPKADHKAC
101  MKLLDDAGIY VISDLSEPSV SINRDDPKWD VELYERYIGV VDELGQYDNV
151  VGFFAGNEVS NNVSNTQASA FVKAAVRDTK KHIKSKFSRW LGVGYASNDD
201  VDIREQIADY FNCGDDDSRI DYWGYNIYSW CGKSSMQDSG YADQAKFFEN
251  YSVPVFFAEY GCNEPDGAAG RIFDETTALY EEKVMTDVFS GGIVYMYFQE
301  ANDYGLVKIS KNGDAVKQKD FAQLQKKANA AKPSGVEEDS YKPTGKAATC
351  PEQSKNWRAN SVLPPVPDSD LCDCMVKSRS CVPADNLKAK DFNDIFGYIC
401  GQDKKICTAI NANATAGIYG AYSMCSNEAK LAYILDAYYT SQKSAADACD
451  FKGKATTQKA ESQDSCKSAL ASASKINEEV ATATHAVASS STGGSNSSSE
501  DDENFGLQAA SIARVFSLGD FAVGAYMAVA GVVGAGMVLL
```

Matched peptides information:

Start - End	Observed	Mr(expt)	Mr(calc)	ppm	M	Peptide
19 - 28	1119.6308	1118.6235	1118.6005	20.6	1	K.LEPITMKGSK.L + Oxidation (M)
29 - 40	1498.7480	1497.7407	1497.6962	29.7	0	K.LFYSNGTQFFMK.G + Oxidation (M)
41 - 57	1738.7533	1737.7460	1737.7806	-19.9	0	K.GVAYQQDTAAAGETNDK.T
74 - 87	1593.7867	1592.7794	1592.9613	-114	1	R.DIPLLKQLGTNIIR.T
80 - 87	914.6248	913.6174	913.5345	90.7	0	K.QLGTNIIR.T
88 - 94	806.5183	805.5110	805.4334	96.4	0	R.TYAINPK.A
103 - 124	2376.1840	2375.1767	2375.2220	-19.1	0	K.LLDDAGIYVISDLSEPSVSINR.D
103 - 128	2831.2972	2830.2899	2830.4236	-47.2	1	K.LLDDAGIYVISDLSEPSVSINRDDPK.W
125 - 136	1564.7377	1563.7304	1563.7205	6.33	1	R.DDPKWDVELYER.Y
129 - 136	1109.6158	1108.6085	1108.5189	80.8	0	K.WDVELYER.Y
190 - 204	1679.8419	1678.8346	1678.7951	23.5	0	R.WLGVGYASNDDVDIR.E
205 - 219	1804.7452	1803.7379	1803.7006	20.7	0	R.EQIADYFNCGDDDSR.I
220 - 233	1824.7874	1823.7801	1823.7977	-9.65	0	R.IDYWGYNIYSWCGK.S
234 - 246	1403.6371	1402.6298	1402.5671	44.7	0	K.SSMQDSGYADQAK.F + Oxidation (M)
247 - 271	2843.1309	2842.1236	2842.2333	-38.6	0	K.FFENYSVPVFFAEYGCNEPDGAAGR.I
272 - 283	1458.7453	1457.7380	1457.6926	31.2	0	R.IFDETTALYEEK.V
320 - 326	849.5226	848.5153	848.4392	89.7	0	K.DFAQLQK.K
328 - 346	1948.8530	1947.8457	1947.9538	-55.5	0	K.ANAAKPSGVEEDSYKPTGK.A
359 - 377	2116.9422	2115.9349	2115.9639	-13.7	0	R.ANSVLPPVPDSDLCDCMVK.S
359 - 377	2132.9126	2131.9053	2131.9588	-25.1	0	R.ANSVLPPVPDSDLCDCMVK.S + Oxidation (M)
380 - 388	1003.5582	1002.5509	1002.4804	70.3	0	R.SCVPADNLK.A
389 - 404	1890.8792	1889.8719	1889.8618	5.36	1	K.AKDFNDIFGYICGQDK.K
391 - 404	1691.8289	1690.8216	1690.7297	54.3	0	K.DFNDIFGYICGQDK.K
391 - 405	1819.7631	1818.7558	1818.8247	-37.9	1	K.DFNDIFGYICGQDKK.I
431 - 443	1548.8049	1547.7976	1547.7871	6.78	0	K.LAYILDAYYTSQK.S
444 - 452	984.4819	983.4746	983.4018	74.0	0	K.SAADACDFK.G

Spot No.: **69**

Mascot score: **91**

Species: ***Fusarium oxysporum Fo5176***

Protein name: **phosphoglycerate mutase family protein, putative**

NCBI accession No.: **gi| 342872822**   Sequence coverage %: **70**

Matched peptides No.: **8**   Total peptides No.: **61**

Calculated Mr: **12416**   Calculated *p*I: **5.21**

Annotated PMF **spectra:**

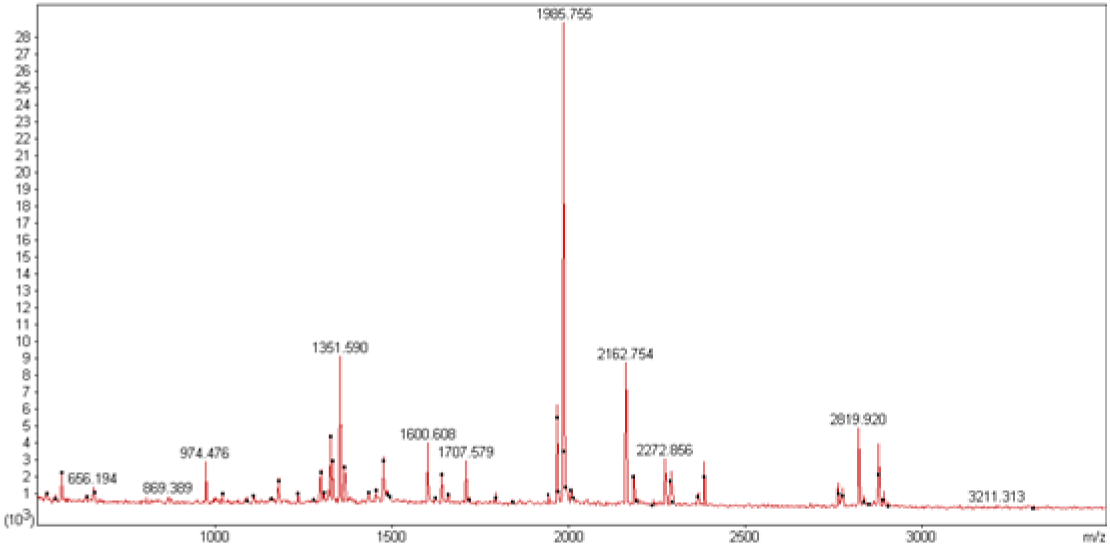

Probability Based Mowse Score:

Protein score is -10*Log(P), where P is the probability that the observed match is a random event. Protein scores greater than 75 are significant (p<0.05).

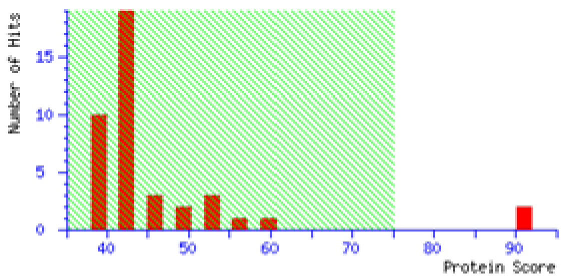

Matched peptide sequences: shown in **Bold Red**

```
  1 MGWFDNNTEV VENFNEYNQN SENREHHAKL SHEIIGGAAA YEAAKAYEEH
 51 VARNGKPDSH AQAKEFIAGA VGAFVDREFE TKGLDFFDRE EAKRHGERKA
101 HRELEEQY
```

Matched peptides information:

Start	-	End	Observed	Mr(expt)	Mr(calc)	ppm	M	Peptide
2	-	24	2819.9198	2818.9125	2818.1855	258	0	M.GWFDNNTEVVENFNEYNQNSENR.E
30	-	45	1600.6083	1599.6010	1599.8256	-140	0	K.LSHEIIGGAAAYEAAK.A
46	-	53	974.4764	973.4691	973.4617	7.56	0	K.AYEEHVAR.N
65	-	77	1351.5898	1350.5825	1350.6932	-81.9	0	K.EFIAGAVGAFVDR.E
65	-	82	1985.7550	1984.7477	1984.9894	-122	1	K.EFIAGAVGAFVDREFETK.G
65	-	82	1986.1287	1985.1214	1984.9894	66.5	1	K.EFIAGAVGAFVDREFETK.G
83	-	89	869.3893	868.3820	868.4079	-29.8	0	K.GLDFFDR.E
83	-	93	1326.5130	1325.5057	1325.6252	-90.1	1	K.GLDFFDREEAK.R

Spot No.: **70**

Mascot score: **217**

Species: ***Fusarium oxysporum f. sp. cubense race 1***

Protein name: **Hsp 70 kda**

NCBI accession No.: **gi| 477507989**

Sequence coverage %: **53**

Matched peptides No.: **25**

Total peptides No.: **46**

Calculated Mr: **71132**

Calculated *p*I: **5.00**

Annotated PMF **spectra:**

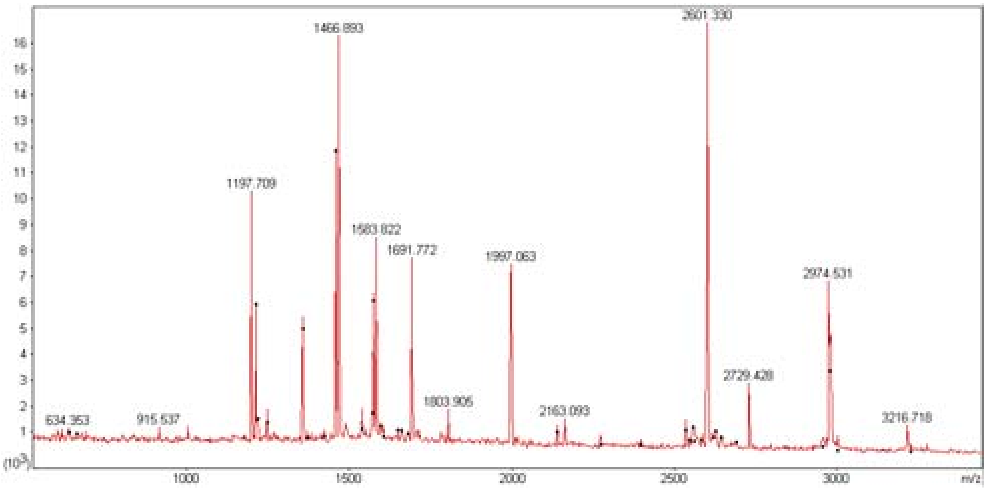

Probability Based Mowse Score:

Protein score is -10*Log(P), where P is the probability that the observed match is a random event.
Protein scores greater than 75 are significant (p<0.05).

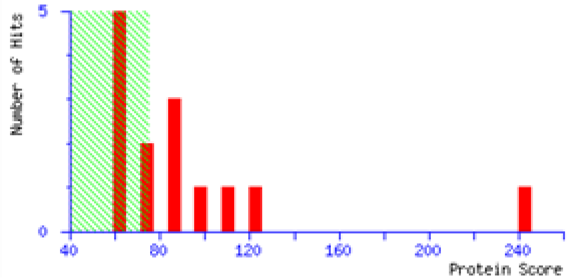

Matched peptide sequences: shown in Bold Red

```
  1 MAPAVGIDLG TTYSCVGIFR EDRCDIIAND QGNRTTPSFV GFTDTERLIG
 51 DAAKNQVAMN PQNTVFDAKR LIGRKFADPE VQADMKHFPF KIVDKGGKPN
101 IEVEFKGETK TFTPEEISAM ILTKMRETAE SYLGETVNNA VVTVPAYFND
151 SQRQATKDAG LIAGLNVLRI INEPTAAAIA YGLDKKVEGE RNVLIFDLGG
201 GTFDVSLLTI EEGIFEVKST AGDTHLGGED FDNRLVNHFV NEFKRKHKKD
251 LSTNVRALRR LRTACERAKR TLSSSAQTSI EIDSLFEGID FYTSITRARF
301 EELCQDLSRS TIQPVDRVLT DAKIDKSLVH EIVLVGGSTR IPRVQKLITD
351 YFNGKEPNKS INPDEAVAYG AAVQAAILSG DTSSKATNEI LLLDVAPLSL
401 GIETAGGMMT KLIPRNTTIP TKKSEVFSTF SDNQPGVLIQ VYEGERQRTK
451 DNNLMGKFEL TGIPPAPRGV PQIEVTFDLD ANGIMNVSAV EKGTGKSNKI
501 VITNDKGRLS KEEIERMLND AEKYKEEDEA EGKRVAAKNG LESYAYSLRN
551 TLSDPKVEEK IEASDKETLT AEIDKVVQWL DDNQQATREE YEEHQKELEG
601 KANPIMMKFY GAGGEGAPGG MPGGPGGFPG AGGPGGAPGA GGDDGPTVEE
651 VD
```

Matched peptides information:

Start	–	End	Observed	Mr(expt)	Mr(calc)	ppm	M	Peptide
2	–	20	1997.0625	1996.0552	1996.0088	23.2	0	M.APAVGIDLGTTYSCVGIFR.E
2	–	23	2397.2500	2396.2427	2396.1795	26.4	1	M.APAVGIDLGTTYSCVGIFREDR.C
35	–	47	1457.7492	1456.7419	1456.6835	40.1	0	R.TTPSFVGFTDTER.L
96	–	106	1217.6915	1216.6842	1216.6452	32.1	0	K.GGKPNIEVEFK.G
111	–	124	1596.8277	1595.8204	1595.8116	5.50	0	K.TFTPEEISAMILTK.M + Oxidation (M)
127	–	153	2974.5312	2973.5239	2973.3992	41.9	0	R.ETAESYLGETVNNAVVTVPAYFNDSQR.Q
158	–	169	1211.7682	1210.7609	1210.7034	47.6	0	K.DAGLIAGLNVLR.I
170	–	185	1659.9102	1658.9029	1658.8879	9.06	0	R.IINEPTAAAIAYGLDK.K
219	–	234	1691.7720	1690.7647	1690.7183	27.4	0	K.STAGDTHLGGEDFDNR.L
235	–	244	1246.6876	1245.6803	1245.6506	23.9	0	R.LVNHFVNEFK.R
271	–	297	2981.6042	2980.5969	2980.4553	47.5	0	R.TLSSSAQTSIEIDSLFEGIDFYTSITR.A
310	–	317	915.5368	914.5295	914.4821	51.8	0	R.STIQPVDR.V
327	–	340	1466.8928	1465.8855	1465.8253	41.1	0	K.SLVHEIVLVGGSTR.I
347	–	359	1538.8155	1537.8082	1537.7776	19.9	1	K.LITDYFNGKEPNK.S
360	–	385	2535.3462	2534.3389	2534.2500	35.1	0	K.SINPDEAVAYGAAVQAAILSGDTSSK.A
386	–	411	2690.4662	2689.4589	2689.3918	25.0	0	K.ATNEILLLDVAPLSLGIETAGGMMTK.L + 2 Oxidation (M)
423	–	446	2729.4277	2728.4204	2728.3344	31.5	1	K.KSEVFSTFSDNQPGVLIQVYEGER.Q
424	–	446	2601.3303	2600.3230	2600.2395	32.1	0	K.SEVFSTFSDNQPGVLIQVYEGER.Q
458	–	468	1197.7095	1196.7022	1196.6553	39.2	0	K.FELTGIPPAPR.G
469	–	492	2546.3613	2545.3540	2545.2734	31.7	0	R.GVPQIEVTFDLDANGIMNVSAVEK.G
469	–	492	2562.3555	2561.3482	2561.2683	31.2	0	R.GVPQIEVTFDLDANGIMNVSAVEK.G + Oxidation (M)
561	–	566	662.3615	661.3542	661.3282	39.3	0	K.IEASDK.E
576	–	588	1572.8122	1571.8049	1571.7692	22.7	0	K.VVQWLDDNQQATR.E
576	–	596	2645.3388	2644.3315	2644.2153	43.9	1	K.VVQWLDDNQQATREEYEEHQK.E
589	–	601	1647.8036	1646.7963	1646.7423	32.8	1	R.EEYEEHQKELEGK.A

Spot No.: **71**

Mascot score: **174**

Species: ***Fusarium oxysporum Fo5176***

Protein name: **hsp 90**

NCBI accession No.: **gi| 342889878**

Sequence coverage %: **41**

Matched peptides No.: **26**

Total peptides No.: **67**

Calculated Mr: **79285**

Calculated *p*I: **4.85**

Annotated PMF **spectra:**

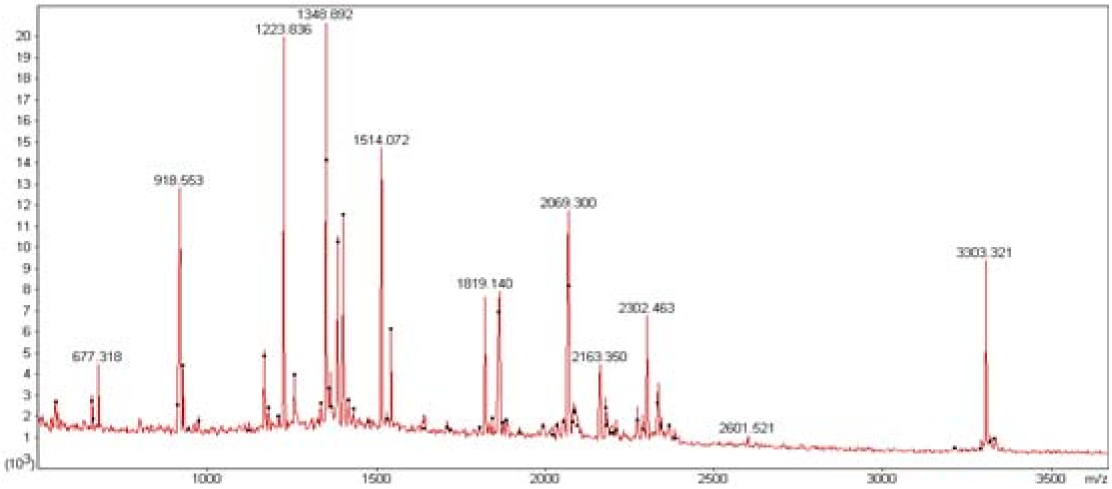

Probability Based Mowse Score:

Protein score is -10*Log(P), where P is the probability that the observed match is a random event. Protein scores greater than 75 are significant (p<0.05).

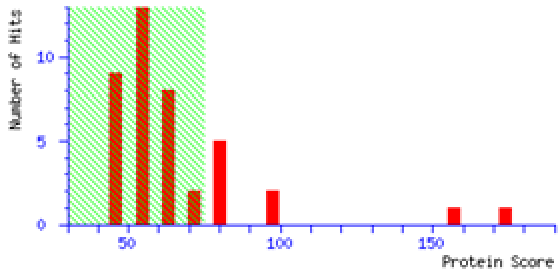

Matched peptide sequences: shown in Bold Red

1	MSETFEFQAE	ISQLLSLIIN	TVYSNKEIFL	RELVSNASDA	LDKIRYKALS
51	DPSQLDSGKD	LRIDIIPDKA	NKTLTIRDTG	IGMTKADLVN	NLGTIARSGT
101	KQFMEALTAG	ADVSMIGQFG	VGFYSAYLVA	DQVRVISKNN	DDEQYVWESS
151	AGGTFSITED	TEGEPLGRGT	AIILHLKDEQ	TEYLNESKIK	EVIKKHSEFI
201	SYPIYLHVEK	ETEKEVPDEE	AEEVTEEGDD	KKPKIEEVDD	DEEEKKPKTK
251	KIKETKIEEE	ELNKQKPIWT	RNPQDISQEE	YASFYKSLSN	DWEDHLAVKH
301	FSVEGQLEFR	AILFVPKRAP	FDLFETKKTK	NNIKLYVRRV	FITDDATDLI
351	PEWLGFVKGV	VDSEDLPLNL	SRETLQQNKI	MKVIKKNIVK	KSLELFQEIA
401	EDKEQFDKFY	SAFSKNLKLG	IHEDSQNRSI	LAKLLRFNST	KSGDELTSLS
451	DYVTRMPEHQ	NNMYYITGES	IKAVSKSPFL	DALREKGFEV	LFLVDPIDEY
501	AMTQLKEFEG	KKLVDITKDF	ELEETEDEKK	AREAEEKEYE	SLAKALKNVL
551	GDKVEKVVVS	HKLGTSPCAI	RTGQFGWSAN	MERIMKAQAL	RDTSMSSYMS
601	SKKTFEISPK	SPIVQELKKK	VETDGENDRT	VKSIVQLLFE	TSLLVSGFTI
651	DEPAGFADRI	HKLVQLGLNI	EEDDSAPAEA	DAATDAPAAA	TGDSAMEEVD

Matched peptides information:

Start	-	End	Observed	Mr(expt)	Mr(calc)	ppm	M	Peptide
27	-	31	677.3177	676.3104	676.3908	-119	0	K.EIFLR.E
86	-	97	1256.8894	1255.8821	1255.6884	154	0	K.ADLVNNLGTIAR.S
139	-	168	3303.3214	3302.3141	3302.4124	-29.7	0	K.NNDDEQYVWESSAGGTFSITEDTEGEPLGR.G
169	-	188	2302.4627	2301.4554	2301.1852	117	1	R.GTAIILHLKDEQTEYLNESK.I
196	-	210	1862.2485	1861.2412	1860.9410	161	0	K.HSEFISYPIYLHVEK.E
215	-	234	2273.4426	2272.4353	2272.0230	181	1	K.EVPDEEAEEVTEEGDDKKPK.I
235	-	245	1349.6935	1348.6862	1348.5518	99.7	0	K.IEEVDDDEEEK.K
265	-	271	928.5720	927.5647	927.5290	38.5	0	K.QKPIWTR.N
272	-	286	1819.1402	1818.1329	1817.8108	177	0	R.NPQDISQEEYASFYK.S
300	-	310	1348.8922	1347.8849	1347.6572	169	0	K.HFSVEGQLEFR.A
311	-	318	943.5204	942.5131	942.6015	-93.8	1	R.AILFVPKR.A
318	-	327	1223.8355	1222.8282	1222.6346	158	1	K.RAPFDLFETK.K
339	-	358	2335.5074	2334.5001	2334.2260	117	1	R.RVFITDDATDLIPEWLGFVK.G
340	-	358	2179.4058	2178.3985	2178.1249	126	0	R.VFITDDATDLIPEWLGFVK.G
359	-	372	1514.0720	1513.0647	1512.7784	189	0	K.GVVDSEDLPLNLSR.E
392	-	408	2069.2999	2068.2926	2068.0000	141	1	K.SLELFQEIAEDKEQFDK.F
419	-	428	1168.7413	1167.7340	1167.5632	146	0	K.LGIHEDSQNR.S
456	-	472	2055.2621	2054.2548	2053.9237	161	0	R.MPEHQNNMYYITGESIK.A
456	-	472	2071.1950	2070.1877	2069.9186	130	0	R.MPEHQNNMYYITGESIK.A + Oxidation (M)
456	-	472	2087.2394	2086.2321	2085.9135	153	0	R.MPEHQNNMYYITGESIK.A + 2 Oxidation (M)
477	-	484	918.5528	917.5455	917.4971	52.8	0	K.SPFLDALR.E
485	-	506	2601.5215	2600.5142	2600.3084	79.2	1	R.EKGFEVLFLVDPIDEYAMTQLK.E + Oxidation (M)
487	-	506	2344.4203	2343.4130	2343.1708	103	0	K.GFEVLFLVDPIDEYAMTQLK.E + Oxidation (M)
563	-	571	974.5456	973.5383	973.5015	37.8	0	K.LGTSPCAIR.T
572	-	583	1383.8868	1382.8795	1382.6037	199	0	R.TGQFGWSANMER.I
572	-	583	1399.8642	1398.8569	1398.5986	185	0	R.TGQFGWSANMER.I + Oxidation (M)

Spot No.: **72**

Mascot score: **81**

Species: ***Ogataea parapolymorpha DL-1***

Protein name: **tRNA ligase**

NCBI accession No.: **gi| 320580493** Sequence coverage %: **25**

Matched peptides No.: **17** Total peptides No.: **91**

Calculated Mr: **91382** Calculated *p*I: **6.15**

Annotated PMF **spectra:**

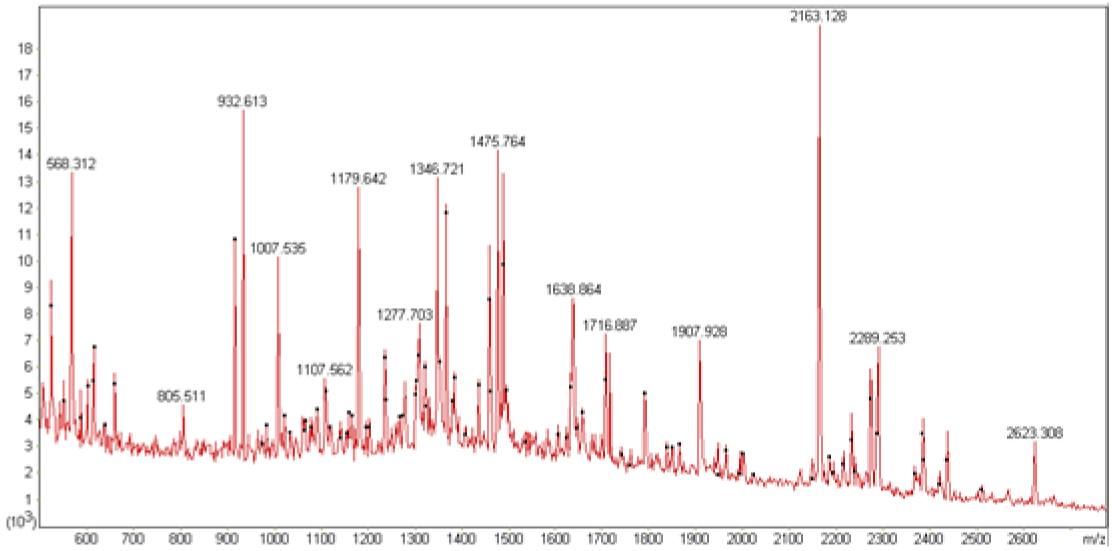

Probability Based Mowse Score:

Protein score is -10*Log(P), where P is the probability that the observed match is a random event.
Protein scores greater than 75 are significant (p<0.05).

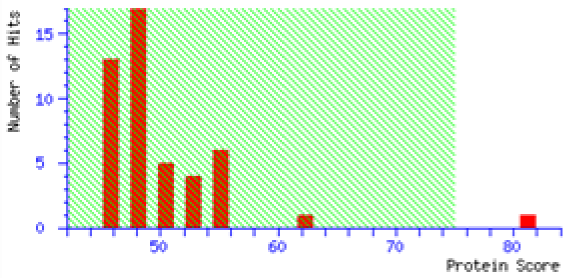

Matched peptide sequences: shown in Bold Red

```
  1 MNILDLQIER VSQDESEKSK QLVSELEELS RSTAKRGIVK KYTNTIDGSD
 51 VQISSWRFNE WDYSSKKVKL PIYARGLFTV GDRIVCRGYD KFFNVDELGS
101 VSRKALQETT TGPYTLTVKA NGCIVFISGL EDGTLVVCSK HSTGYRDDLT
151 RNHALAAQAA LKRQLEKNDI NSTDLAKTLY QLGITAVAEY CDDSFEEHIL
201 EYTQEKAGLY LHGLNYNTCN FKTCSMAVVT KFAQAFGFKT IDYFEVPTFE
251 ETMELLEKAS ETGLHNGEEI EGFVVRCQKD SSDFFFKYKF EEPYLLYREL
301 REVTKQYLTK GLDNVSFKNH KLICMDYLKF VIPLFDADPS LKENYLKDKG
351 IVKLRKLYME HKQKSGSEII KEEQSMEKLE EELKCAGYGQ DTCKYVLVTV
401 ATIGCGKTTT SMTLANLFPD LIGVVQSDDI PSPIKNKQVA KCLEVLVEKP
451 IVILDRNNHK FIERQQTFEY FADLNKLIPT SKLKFICLNF LGNMSKNDPK
501 LWEITRARVL ERGDNHQSIK VERDGSYKAE MIMKGFLGRF QPVEKDKFPD
551 SRFDHVIDLR VDKDSSLENA KLIARRLAEI ATDVNLQYPS EEQFLEAYQK
601 ALDFKPVVTK NFKTKKEKPQ YFGILVDDID KLAQLHEIGF FKQLQEANRV
651 KKEFHITLIH LGSTKKNPPM KTIYQNYCEL IKDMEKVNDQ IELPYKADAR
701 LFRICWNSRV MCIEAEVKRI YGEEGERLDS LGIGNKYPHI TVGTVSEKVR
751 ALESNKLLSD LHDFGDENIN TLDLNIELNQ LPVFVHY
```

Matched peptides information:

Start	–	End	Observed	Mr(expt)	Mr(calc)	ppm	M	Peptide
1	–	18	2163.1276	2162.1203	2162.0525	31.4	1	-.MNILDLQIERVSQDESEK.S + Oxidation (M)
21	–	31	1302.6616	1301.6543	1301.6827	-21.8	0	K.QLVSELEELSR.S
120	–	140	2239.2893	2238.2820	2238.1025	80.2	0	K.ANGCIVFISGLEDGTLVVCSK.H
141	–	151	1320.6265	1319.6192	1319.6218	-1.97	1	K.HSTGYRDDLTR.N
147	–	162	1707.8016	1706.7943	1706.9063	-65.6	1	R.DDLTRNHALAAQAALK.R
152	–	162	1107.5616	1106.5543	1106.6196	-59.0	0	R.NHALAAQAALK.R
168	–	177	1090.5502	1089.5429	1089.5302	11.7	0	K.NDINSTDLAK.T
232	–	239	915.5906	914.5833	914.4650	129	0	K.FAQAFGFK.T
302	–	310	1109.5407	1108.5334	1108.6128	-71.6	1	R.EVTKQYLTK.G
319	–	329	1434.7611	1433.7538	1433.7159	26.5	1	K.NHKLICMDYLK.F
348	–	353	659.5137	658.5064	658.4014	160	1	K.DKGIVK.L
442	–	460	2289.2526	2288.2453	2288.2674	-9.67	1	K.CLEVLVEKPIVILDRNNHK.F
535	–	545	1277.7030	1276.6957	1276.6928	2.28	1	K.GFLGRFQPVEK.D
616	–	631	1907.9278	1906.9205	1907.0040	-43.8	1	K.KEKPQYFGILVDDIDK.L
672	–	686	1964.0324	1963.0251	1962.9067	60.4	1	K.TIYQNYCELIKDMEK.V + Oxidation (M)
710	–	719	1234.6850	1233.6777	1233.6209	46.0	1	R.VMCIEAEVKR.I
720	–	736	1849.9357	1848.9284	1848.9217	3.62	1	R.IYGEEGERLDSLGIGNK.Y

Spot No.: **73**

Mascot score: **117**

Species: ***Fusarium oxysporum f. sp. cubense race 1***

Protein name: **Hsp 70 kda**

NCBI accession No.: **gi| 477507989**

Sequence coverage %: **46**

Matched peptides No.: **21**

Total peptides No.: **61**

Calculated Mr: **71132**

Calculated *p*I: **5.00**

Annotated PMF **spectra:**

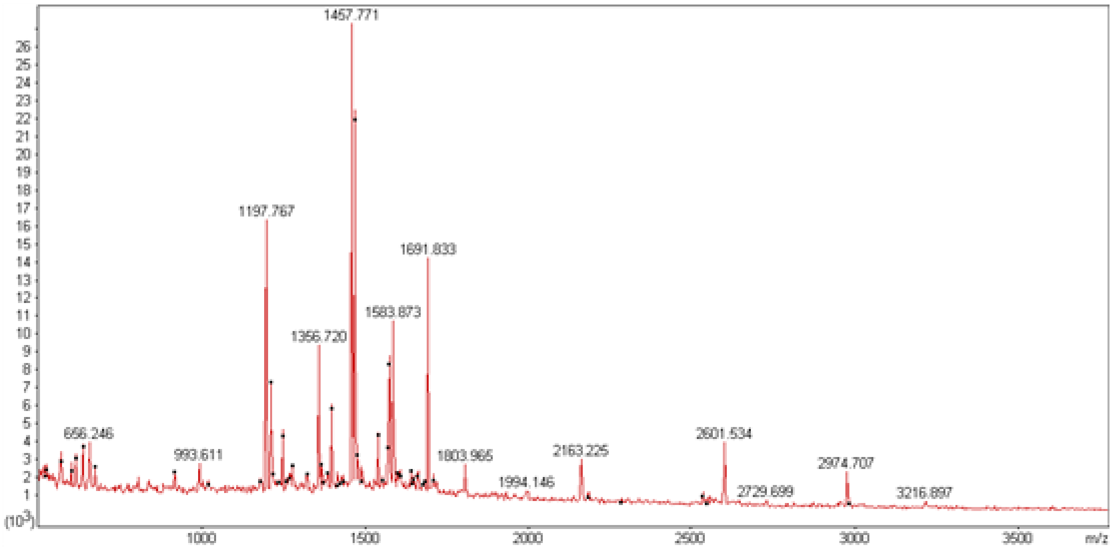

Probability Based Mowse Score:

Protein score is -10*Log(P), where P is the probability that the observed match is a random event.
Protein scores greater than 75 are significant (p<0.05).

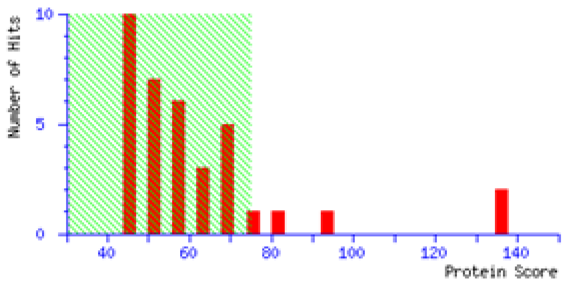

Matched peptide sequences: shown in Bold Red

```
  1 MAPAVGIDLG TTYSCVGIFR EDRCDIIAND QGNRTTPSFV GFTDTERLIG
 51 DAAKNQVAMN PQNTVFDAKR LIGRKFADPE VQADMKHFPF KIVDKGGKPN
101 IEVEFKGETK TFTPEEISAM ILTKMRETAE SYLGETVNNA VVTVPAYFND
151 SQRQATKDAG LIAGLNVLRI INEPTAAAIA YGLDKKVEGE RNVLIFDLGG
201 GTFDVSLLTI EEGIFEVKST AGDTHLGGED FDNRLVNHFV NEFKRKHKKD
251 LSTNVRALRR LRTACERAKR TLSSSAQTSI EIDSLFEGID FYTSITRARF
301 EELCQDLSRS TIQPVDRVLT DAKIDKSLVH EIVLVGGSTR IPRVQKLITD
351 YFNGKEPNKS INPDEAVAYG AAVQAAILSG DTSSKATNEI LLLDVAPLSL
401 GIETAGGMMT KLIPRNTTIP TKKSEVFSTF SDNQPGVLIQ VYEGERQRTK
451 DNNLMGKFEL TGIPPAPRGV PQIEVTFDLD ANGIMNVSAV EKGTGKSNKI
501 VITNDKGRLS KEEIERMLND AEKYKEEDEA EGKRVAAKNG LESYAYSLRN
551 TLSDPKVEEK IEASDKETLT AEIDKVVQWL DDNQQATREE YEEHQKELEG
601 KANPIMMKFY GAGGEGAPGG MPGGPGGFPG AGGPGGAPGA GGDDGPTVEE
651 VD
```

Matched peptides information:

Start	-	End	Observed	Mr(expt)	Mr(calc)	ppm	M	Peptide
24	-	34	1275.6539	1274.6466	1274.5673	62.2	0	R.CDIIANDQGNR.T
35	-	47	1457.7706	1456.7633	1456.6835	54.8	0	R.TTPSFVGFTDTER.L
55	-	69	1676.8830	1675.8757	1675.7988	45.9	0	K.NQVAMNPQNTVFDAK.R
76	-	86	1266.6897	1265.6824	1265.5598	96.9	0	K.FADPEVQADMK.H + Oxidation (M)
96	-	106	1217.7190	1216.7117	1216.6452	54.7	0	K.GGKPNIEVEFK.G
111	-	124	1596.8711	1595.8638	1595.8116	32.7	0	K.TFTPEEISAMILTK.M + Oxidation (M)
127	-	153	2974.7066	2973.6993	2973.3992	101	0	R.ETAESYLGETVNNAVVTVPAYFNDSQR.Q
158	-	169	1211.7991	1210.7918	1210.7034	73.1	0	K.DAGLIAGLNVLR.I
170	-	185	1659.9481	1658.9408	1658.8879	31.9	0	R.IINEPTAAAIAYGLDK.K
219	-	234	1691.8326	1690.8253	1690.7183	63.3	0	K.STAGDTHLGGEDFDNR.L
235	-	244	1246.7109	1245.7036	1245.6506	42.6	0	R.LVNHFVNEFK.R
271	-	297	2981.8155	2980.8082	2980.4553	118	0	R.TLSSSAQTSIEIDSLFEGIDFYTSITR.A
310	-	317	915.5988	914.5915	914.4821	120	0	R.STIQPVDR.V
327	-	340	1466.9096	1465.9023	1465.8253	52.6	0	K.SLVHEIVLVGGSTR.I
360	-	385	2535.4723	2534.4650	2534.2500	84.8	0	K.SINPDEAVAYGAAVQAAILSGDTSSK.A
423	-	446	2729.6986	2728.6913	2728.3344	131	1	K.KSEVFSTFSDNQPGVLIQVYEGER.Q
424	-	446	2601.5344	2600.5271	2600.2395	111	0	K.SEVFSTFSDNQPGVLIQVYEGER.Q
449	-	457	1020.5838	1019.5765	1019.5070	68.2	1	R.TKDNNLMGK.F
458	-	468	1197.7666	1196.7593	1196.6553	86.9	0	K.FELTGIPPAPR.G
576	-	588	1572.8589	1571.8516	1571.7692	52.4	0	K.VVQWLDDNQQATR.E
589	-	601	1647.8075	1646.8002	1646.7423	35.2	1	R.EEYEEHQKELEGK.A

Spot No.: **74**

Mascot score: **110**

Species: ***Fusarium oxysporum Fo5176***

Protein name: **hsp 90**

NCBI accession No.: **gi| 342889878**

Sequence coverage %: **34**

Matched peptides No.: **19**

Total peptides No.: **59**

Calculated Mr: **79285**

Calculated *p*I: **4.85**

Annotated PMF **spectra:**

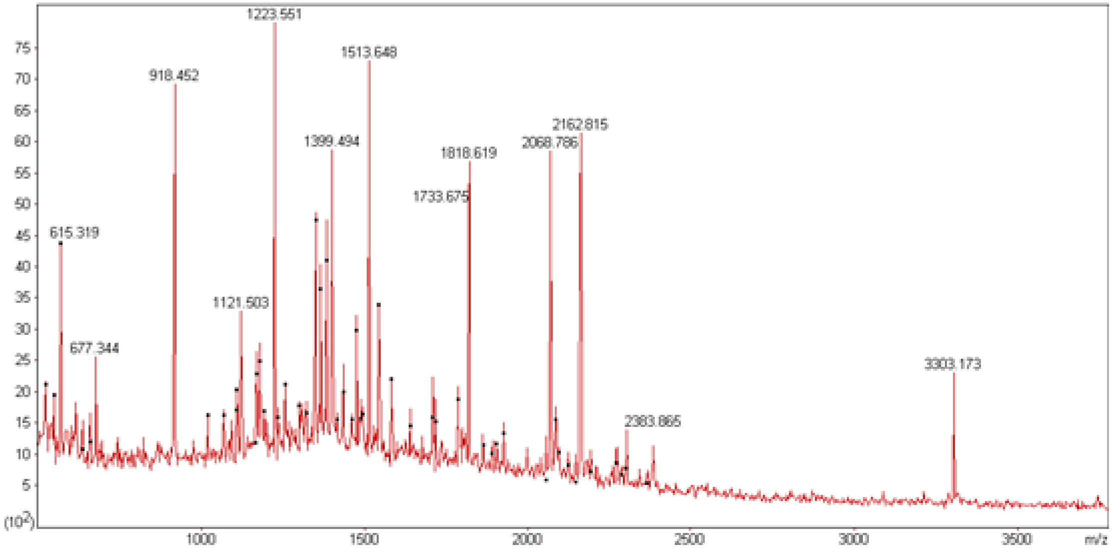

Probability Based Mowse Score:

Protein score is -10*Log(P), where P is the probability that the observed match is a random event.
Protein scores greater than 75 are significant (p<0.05).

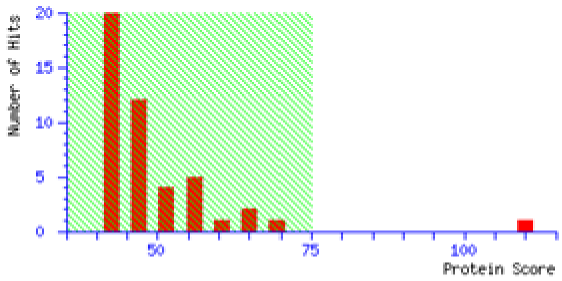

Matched peptide sequences: shown in Bold Red

```
  1 MSETFEFQAE ISQLLSLIIN TVYSNKEIFL RELVSNASDA LDKIRYKALS
 51 DPSQLDSGKD LRIDIIPDKA NKTLTIRDTG IGMTKADLVN NLGTIARSGT
101 KQFMEALTAG ADVSMIGQFG VGFYSAYLVA DQVRVISKNN DDEQYVWESS
151 AGGTFSITED TEGEPLGRGT AIILHLKDEQ TEYLNESKIK EVIKKHSEFI
201 SYPIYLHVEK ETEKEVPDEE AEEVTEEGDD KKPKIEEVDD DEEEKKPKTK
251 KIKETKIEEE ELNKQKPIWT RNPQDISQEE YASFYKSLSN DWEDHLAVKH
301 FSVEGQLEFR AILFVPKRAP FDLFETKKTK NNIKLYVRRV FITDDATDLI
351 PEWLGFVKGV VDSEDLPLNL SRETLQQNKI MKVIKKNIVK KSLELFQEIA
401 EDKEQFDKFY SAFSKNLKLG IHEDSQNRSI LAKLLRFNST KSGDELTSLS
451 DYVTRMPEHQ NNMYYITGES IKAVSKSPFL DALREKGFEV LFLVDPIDEY
501 AMTQLKEFEG KKLVDITKDF ELEETEDEKK AREAEEKEYE SLAKALKNVL
551 GDKVEKVVVS HKLGTSPCAI RTGQFGWSAN MERIMKAQAL RDTSMSSYMS
601 SKKTFEISPK SPIVQELKKK VETDGENDRT VKSIVQLLFE TSLLVSGFTI
651 DEPAGFADRI HKLVQLGLNI EEDDSAPAEA DAATDAPAAA TGDSAMEEVD
```

Matched peptides information:

Start – End	Observed	Mr(expt)	Mr(calc)	ppm	M	Peptide
27 – 31	677.3439	676.3366	676.3908	-80.1	0	K.EIFLR.E
86 – 97	1256.5688	1255.5615	1255.6884	-101	0	K.ADLVNNLGTIAR.S
139 – 168	3303.1726	3302.1653	3302.4124	-74.8	0	K.NNDDEQYVWESSAGGTFSITEDTEGEPLGR.G
169 – 188	2301.9511	2300.9438	2301.1852	-105	1	R.GTAIILHLKDEQTEYLNESK.I
196 – 210	1861.7062	1860.6989	1860.9410	-130	0	K.HSEFISYPIYLHVEK.E
215 – 234	2272.8961	2271.8888	2272.0230	-59.1	1	K.EVPDEEAEEVTEEGDDKKPK.I
235 – 245	1349.4603	1348.4530	1348.5518	-73.2	0	K.IEEVDDDEEEK.K
272 – 286	1818.6185	1817.6112	1817.8108	-110	0	R.NPQDISQEEYASFYK.S
287 – 299	1513.6480	1512.6407	1512.7209	-53.0	0	K.SLSNDWEDHLAVK.H
319 – 327	1067.4489	1066.4416	1066.5335	-86.2	0	R.APFDLFETK.K
335 – 338	550.2576	549.2504	549.3275	-140	0	K.LYVR.R
392 – 408	2068.7855	2067.7782	2068.0000	-107	1	K.SLELFQEIAEDKEQFDK.F
419 – 428	1168.5224	1167.5151	1167.5632	-41.2	0	K.LGIHEDSQNR.S
442 – 455	1542.6073	1541.6000	1541.7209	-78.4	0	K.SGDELTSLSDYVTR.M
456 – 472	2086.7197	2085.7124	2085.9135	-96.4	0	R.MPEHQNNMYYITGESIK.A + 2 Oxidation (M)
477 – 484	918.4522	917.4450	917.4971	-56.8	0	K.SPFLDALR.E
572 – 583	1399.4944	1398.4871	1398.5986	-79.7	0	R.TGQFGWSANMER.I + Oxidation (M)
592 – 602	1223.5511	1222.5438	1222.4846	48.5	0	R.DTSMSSYMSSK.K
592 – 603	1383.5033	1382.4960	1382.5694	-53.0	1	R.DTSMSSYMSSKK.T + 2 Oxidation (M)

Spot No.: **75**

Mascot score: **98**

Species: ***Fusarium oxysporum Fo5176***

Protein name: **ATP synthase D chain, mitochondrial**

NCBI accession No.: **gi| 342876053**

Sequence coverage %: **60**

Matched peptides No.: **10**

Total peptides No.: **59**

Calculated Mr: **19411**

Calculated *p*I: **6.43**

Annotated PMF **spectra:**

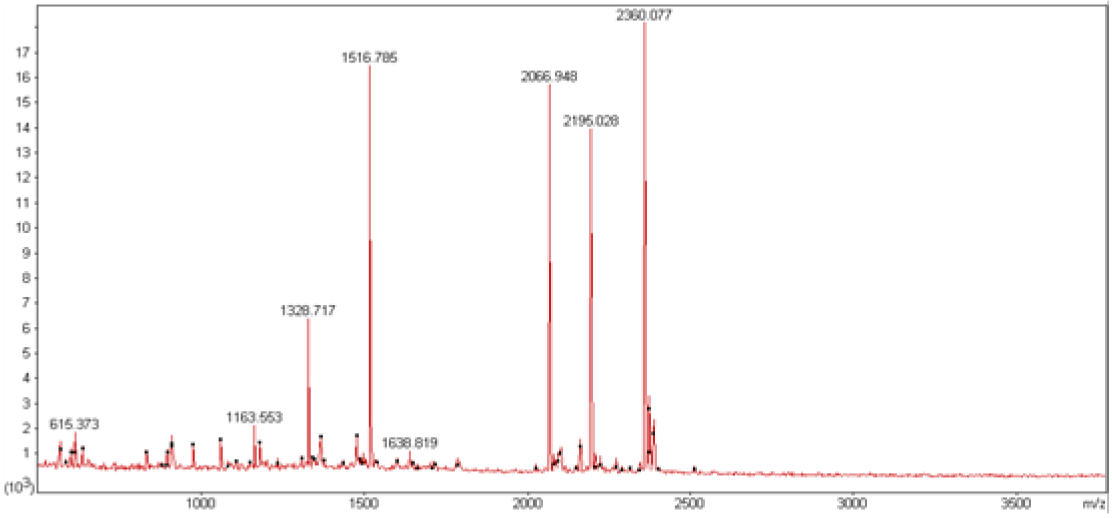

Probability Based Mowse Score:

Protein score is -10*Log(P), where P is the probability that the observed match is a random event.
Protein scores greater than 75 are significant (p<0.05).

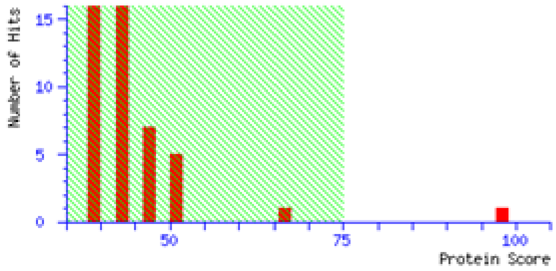

Matched peptide sequences: shown in **Bold Red**

1 MATRSAALK**L** **DWTKVTSSLG** **LRGQTVASLQ** **AFK**KRNEDVR R**KVQQLQEQP**

51 **TTVDFSQYR**S VLK**NQAIIDE** **IEKRFSAFKP** **VTYDVSR**QLK **AIDAFEAEAV**

101 **K**NAEATKEAV DLELKDLAAT LK**NIEEARPF** **EELTVDEVAA** **AEK**SIDEKTA

151 QLVSKGR**WMV** **PGYK**EKFGDL AVV

Matched peptides information:

Start	-	End	Observed	Mr(expt)	Mr(calc)	ppm	M	Peptide
10	-	22	1475.7354	1474.7281	1474.8144	-58.5	1	K.LDWTKVTSSLGLR.G
15	-	22	832.4769	831.4697	831.4814	-14.1	0	K.VTSSLGLR.G
23	-	33	1149.5896	1148.5823	1148.6190	-31.9	0	R.GQTVASLQAFK.K
42	-	59	2195.0277	2194.0204	2194.1019	-37.1	1	R.KVQQLQEQPTTVDFSQYR.S
43	-	59	2066.9481	2065.9408	2066.0069	-32.0	0	K.VQQLQEQPTTVDFSQYR.S
64	-	74	1328.7168	1327.7095	1327.7095	-0.0075	1	K.NQAIIDEIEKR.F
75	-	87	1516.7850	1515.7777	1515.7722	3.65	0	R.FSAFKPVTYDVSR.Q
91	-	101	1163.5528	1162.5455	1162.5870	-35.7	0	K.AIDAFEAEAVK.N
123	-	143	2360.0773	2359.0700	2359.1543	-35.7	0	K.NIEEARPFEELTVDEVAAAEK.S
158	-	164	896.4524	895.4452	895.4262	21.2	0	R.WMVPGYK.E + Oxidation (M)

Spot No.: **76**

Mascot score: **85**

Species: ***Fusarium oxysporum Fo5176***

Protein name: **Phospholipase B**

NCBI accession No.: **gi| 342872804** Sequence coverage %: **25**

Matched peptides No.: **15** Total peptides No.: **57**

Calculated Mr: **70452** Calculated *p*I: **4.67**

Annotated PMF **spectra:**

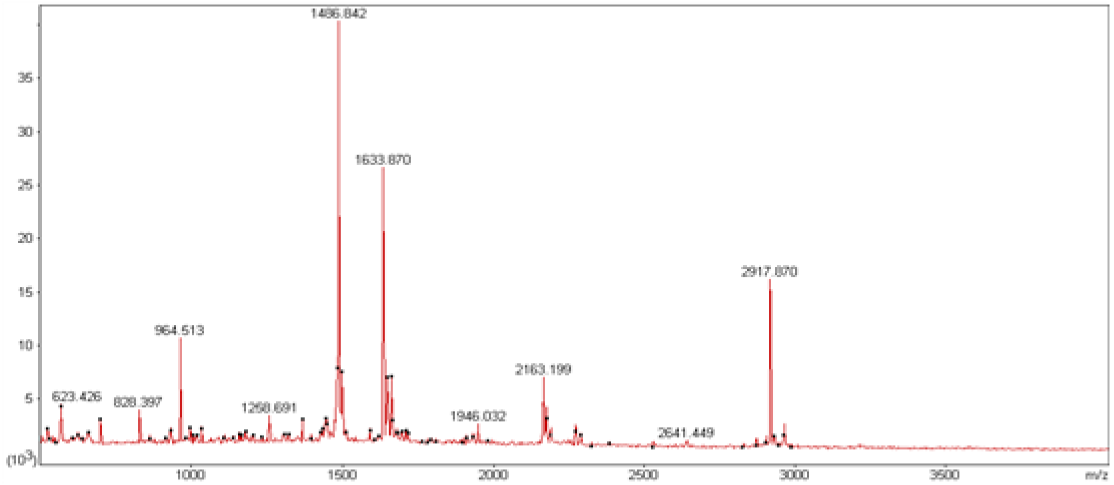

Probability Based Mowse Score:

Protein score is -10*Log(P), where P is the probability that the observed match is a random event.
Protein scores greater than 75 are significant (p<0.05).

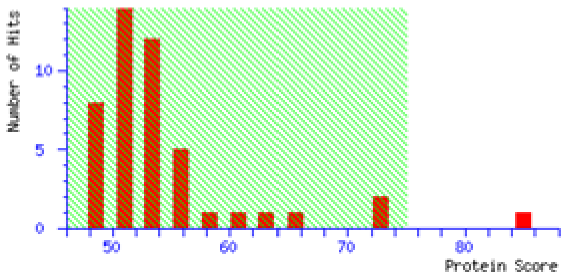

Matched peptide sequences: shown in **Bold Red**

1	MLGFVALTLW	LSTAIAAPDD	TALIPRVNSV	EIR**ALPNSPS**	**GGYAPKVVDC**
51	**PSTRPK**VR**LA**	**DELSSEEEAW**	**VR**RRR**NNTID**	**DLKTFLSR**AN	ISGFDAESFV
101	EKHKNNATGL	PNIAIAASGG	GYRALMNGAG	FLSAADSRNN	KTGPISGLLQ
151	SATYLAGLSG	GGWLVGSIFA	NNFSTVPDLQ	SGEKVWRFDR	SIFKGPK**SSG**
201	**ISLLNTAEYW**	**TEMK**DAVDDK	DKGWNTTLTD	YWGR**ALSYQL**	**VNAPEGGPSY**
251	**TFSSIADTSN**	**FKDADTPFPI**	**LVADGR**APGE	RVISLNATVY	EFNPYEFGTW
301	DPTTFGFVPT	EYLASNFTNG	SISSKGECVR	GFDQIGFVMG	TSSSLFNQFL
351	LNNITK**VGKE**	**NDIPDIVVK**A	IEGVLVGLDQ	DDEDIAQYTP	NPFFGWNPTD
401	KSVNSK**DHQL**	**TLVDGGEDLQ**	**NIPLHPLIQP**	**VR**GVDIIFAI	DSSADTNNNW
451	PNGTALR**ATY**	**DR**VGSSIGNG	TQFPSVPSAE	TFVNEKLNQR	PTLFGCDANN
501	FTLSDGEVPP	PLIFYIPNAP	YTYHSNVSTF	DMSYTTAERD	NIILNALNGA
551	TQGNATIDKE	WPTCVACAVM	SR**SWWK**ANET	VPDACK**TCFD**	**RYCWDGK**SNN
601	TAVKSYEPEY	IIGGNATADA	ADNAAGARLG	PSWFVSAGVG	AAALFALM

Matched peptides information:

Start	-	End	Observed	Mr(expt)	Mr(calc)	ppm	M	Peptide
34	-	46	1258.6914	1257.6841	1257.6353	38.8	0	R.ALPNSPSGGYAPK.V
47	-	56	1158.6505	1157.6432	1157.5863	49.2	0	K.VVDCPSTRPK.V
59	-	72	1633.8699	1632.8626	1632.7631	61.0	0	R.LADELSSEEEAWVR.R
76	-	83	932.5717	931.5644	931.4611	111	0	R.NNTIDDLK.T
84	-	88	623.4257	622.4184	622.3439	120	0	K.TFLSR.A
198	-	214	1930.0682	1929.0609	1928.9189	73.6	0	K.SSGISLLNTAEYWTEMK.D
198	-	214	1946.0323	1945.0250	1944.9139	57.2	0	K.SSGISLLNTAEYWTEMK.D + Oxidation (M)
235	-	262	2964.7941	2963.7868	2963.4189	124	0	R.ALSYQLVNAPEGGPSYTFSSIADTSNFK.D
263	-	276	1486.8424	1485.8351	1485.7464	59.7	0	K.DADTPFPILVADGR.A
357	-	369	1425.8177	1424.8104	1424.7875	16.1	1	K.VGKENDIPDIVVK.A
407	-	432	2917.8699	2916.8626	2916.5458	109	0	K.DHQLTLVDGGEDLQNIPLHPLIQPVR.G
458	-	462	625.3598	624.3525	624.2867	105	0	R.ATYDR.V
573	-	576	606.3591	605.3519	605.2962	92.0	0	R.SWWK.A
587	-	591	698.3931	697.3858	697.2854	144	0	K.TCFDR.Y
592	-	597	828.3966	827.3893	827.3272	75.0	0	R.YCWDGK.S

Spot No.: **77**

NCBI accession No.: **gi|342872804**

Plant species: ***Fusarium oxysporum Fo5176***

Protein name： **Phospholipase B**

Mascot score: **253** Sequence coverage %: **8**

The number of matched peptides with p≤0.05: **4**

Calculated Mr: **70452** Calculated *P*i: **4.67**

Annotated MS **spectra:**

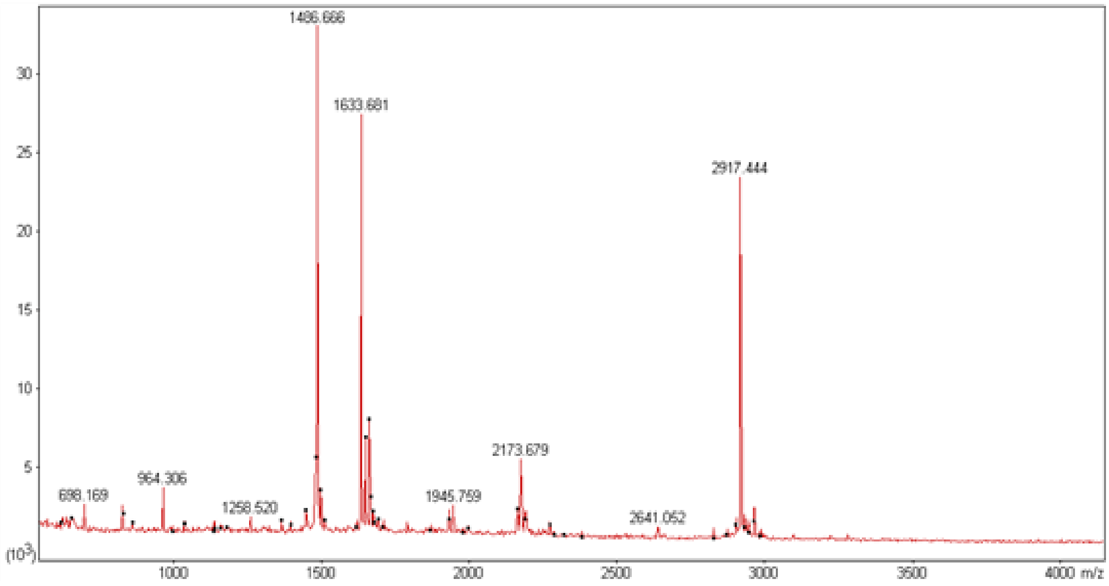

Probability Based Mowse Score:

Ions score is -10*Log(P), where P is the probability that the observed match is a random event.
Individual ions scores > 47 indicate identity or extensive homology (p<0.05).
Protein scores are derived from ions scores as a non-probabilistic basis for ranking protein hits.

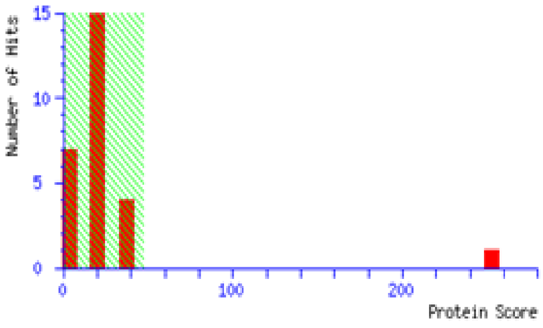

Matched peptide sequences: shown in Bold Red

```
  1 MLGFVALTLW LSTAIAAPDD TALIPRVNSV EIRALPNSPS GGYAPKVVDC
 51 PSTRPKVRLA DELSSEEEAW VRRRRNNTID DLKTFLSRAN ISGFDAESFV
101 EKHKNNATGL PNIAIAASGG GYRALMNGAG FLSAADSRNN KTGPISGLLQ
151 SATYLAGLSG GGWLVGSIFA NNFSTVPDLQ SGEKVWRFDR SIFKGPKSSG
201 ISLLNTAEYW TEMKDAVDDK DKGWNTTLTD YWGRALSYQL VNAPEGGPSY
251 TFSSIADTSN FKDADTPFPI LVADGRAPGE RVISLNATVY EFNPYEFGTW
301 DPTTFGFVPT EYLASNFTNG SISSKGECVR GFDQIGFVMG TSSSLFNQFL
351 LNNITKVGKE NDIPDIVVKA IEGVLVGLDQ DDEDIAQYTP NPFFGWNPTD
401 KSVNSKDHQL TLVDGGEDLQ NIPLHPLIQP VRGVDIIFAI DSSADTNNNW
451 PNGTALRATY DRVGSSIGNG TQFPSVPSAE TFVNEKLNQR PTLFGCDANN
501 FTLSDGEVPP PLIFYIPNAP YTYHSNVSTF DMSYTTAERD NIILNALNGA
551 TQGNATIDKE WPTCVACAVM SRSWWKANET VPDACKTCFD RYCWDGKSNN
601 TAVKSYEPEY IIGGNATADA ADNAAGARLG PSWFVSAGVG AAALFALM
```

Matched peptides information:

Query	Start - End	Observed	Mr(expt)	Mr(calc)	ppm	M	Score	Expect	Rank	U	Peptide
	59 - 72	1633.6810	1632.6737	1632.7631	-54.7	0	91	2.6e-06	1	U	R.LADELSSEEEAWVR.R
	263 - 276	1486.6660	1485.6587	1485.7464	-59.0	0	102	2.1e-07	1	U	K.DADTPFPILVADGR.A
	263 - 276	1486.6660	1485.6587	1485.7464	-59.0	0	102	2.1e-07	1	U	K.DADTPFPILVADGR.A
	407 - 432	2917.4441	2916.4368	2916.5458	-37.4	0	59	0.0021	1	U	K.DHQLTLVDGGEDLQNIPLHPLIQPVR.G

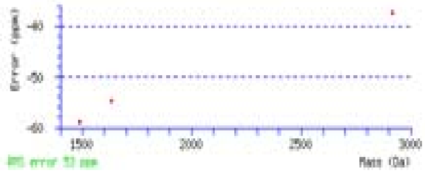

Annotated ion spectra of the matched peptides with p≤0.05:

CID No.: **77-1486.6**

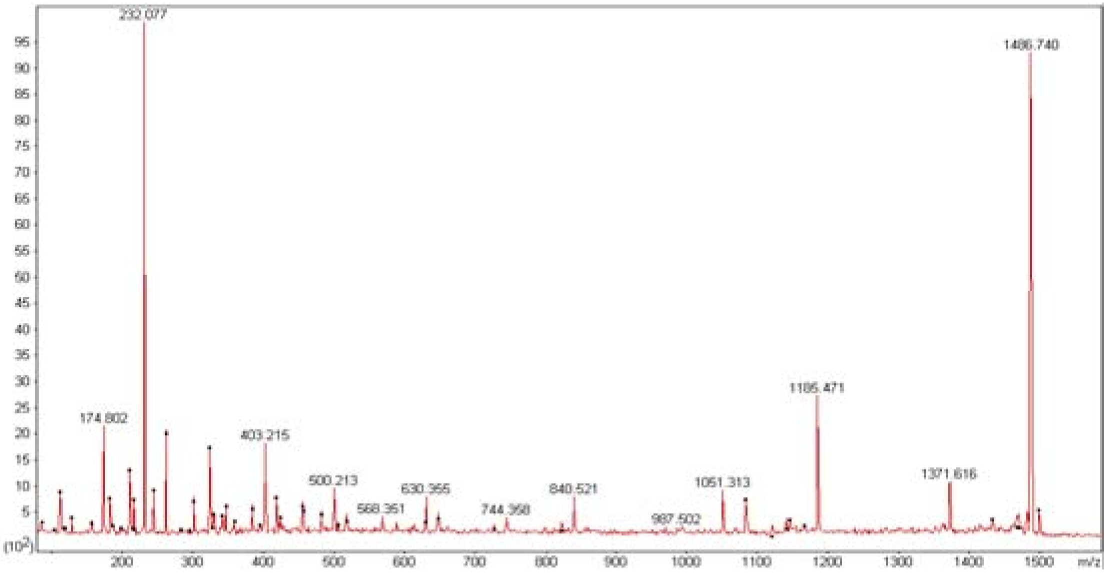

MS/MS Fragmentation of **K.DADTPFPILVADGR.A**

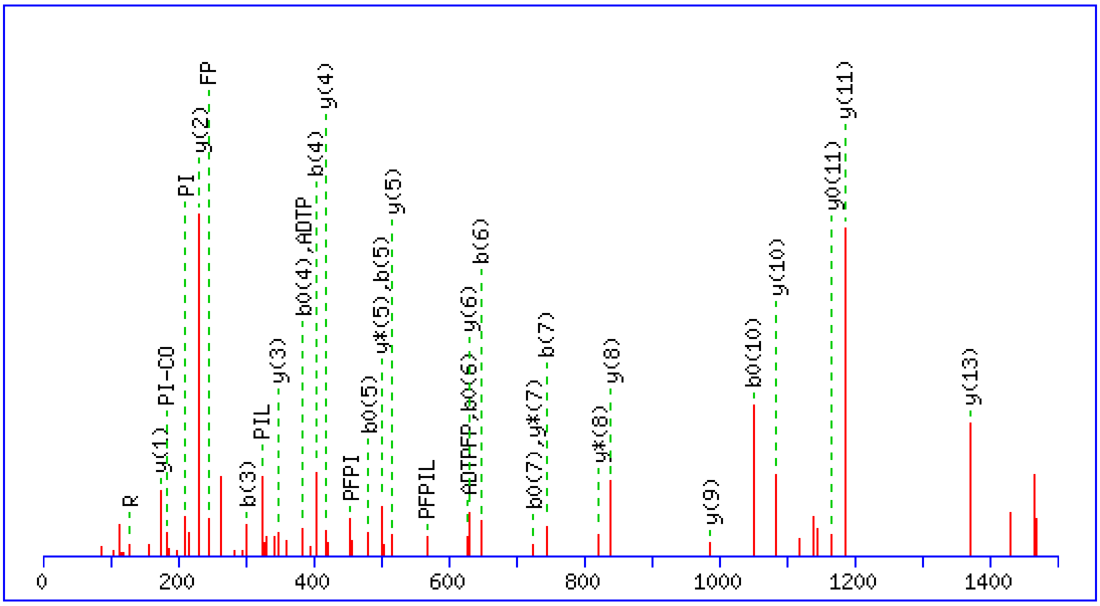

Monoisotopic mass of neutral peptide Mr(calc): 1485.7464

#	Immon.	a	a0	b	b0	d	d'	Seq.	v	w	w'	y	y*	y0	#
1	88.0393	88.0393	70.0287	116.0342	98.0237	44.0495		D							14
2	44.0495	159.0764	141.0659	187.0713	169.0608			A	1355.6954			***1371.7267***	1354.7001	1353.7161	13
3	88.0393	274.1034	256.0928	**302.0983**	284.0877	230.1135		D	1240.6685	1239.6732		1300.6896	1283.6630	1282.6790	12
4	74.0600	375.1510	357.1405	**403.1460**	385.1354	359.1561	361.1354	T	1139.6208	1152.6412	1154.6204	***1185.6626***	1168.6361	1167.6521	11
5	70.0651	472.2038	454.1932	**500.1987**	482.1882	446.1882		P	1042.5680	1041.5728		***1084.6150***	1067.5884	1066.6044	10
6	120.0808	619.2722	601.2617	**647.2671**	629.2566			F	895.4996			***987.5622***	970.5356	969.5516	9
7	70.0651	716.3250	698.3144	**744.3199**	726.3093	690.3093		P	798.4468	797.4516		***840.4938***	823.4672	822.4832	8
8	86.0964	829.4090	811.3985	857.4040	839.3934	801.3777	815.3934	I	685.3628	698.3832	712.3988	743.4410	726.4145	725.4305	7
9	86.0964	942.4931	924.4825	970.4880	952.4775	900.4462		L	572.2787	571.2835		***630.3570***	613.3304	612.3464	6
10	72.0808	1041.5615	1023.5510	1069.5564	1051.5459	1027.5459		V	473.2103	486.2307		***517.2729***	500.2463	499.2623	5
11	44.0495	1112.5986	1094.5881	1140.5936	1122.5830			A	402.1732			***418.2045***	401.1779	400.1939	4
12	88.0393	1227.6256	1209.6150	1255.6205	1237.6099	1183.6358		D	287.1462	286.1510		***347.1674***	330.1408	329.1568	3
13	30.0338	1284.6470	1266.6365	1312.6420	1294.6314			G				***232.1404***	215.1139		2
14	129.1135							R	74.0237	73.0284		***175.1190***	158.0924		1

Seq	ya	yb	Seq	ya	yb	Seq	ya	yb
AD	159.0764	187.0713	ADT	260.1241	288.1190	ADTP	357.1769	385.1718
ADTPF	504.2453	532.2402	ADTPFP	601.2980	629.2930	DT	189.0870	217.0819
DTP	286.1397	314.1347	DTPF	433.2082	461.2031	DTPFP	530.2609	558.2558
DTPFPI	643.3450	671.3399	TP	171.1128	199.1077	TPF	318.1812	346.1761
TPFP	415.2340	443.2289	TPFPI	528.3180	556.3130	TPFPIL	641.4021	669.3970
PF	217.1335	245.1285	PFP	314.1863	342.1812	PFPI	427.2704	455.2653
PFPIL	540.3544	568.3493	PFPILV	639.4228	667.4178	FP	217.1335	245.1285
FPI	330.2176	358.2125	FPIL	443.3017	471.2966	FPILV	542.3701	570.3650
FPILVA	613.4072	641.4021	PI	183.1492	211.1441	PIL	296.2333	324.2282
PILV	395.3017	423.2966	PILVA	466.3388	494.3337	PILVAD	581.3657	609.3606
PILVADG	638.3872	666.3821	IL	199.1805	227.1754	ILV	298.2489	326.2438
ILVA	369.2860	397.2809	ILVAD	484.3130	512.3079	ILVADG	541.3344	569.3293
LV	185.1648	213.1598	LVA	256.2020	284.1969	LVAD	371.2289	399.2238
LVADG	428.2504	456.2453	VA	143.1179	171.1128	VAD	258.1448	286.1397
VADG	315.1663	343.1612	AD	159.0764	187.0713	ADG	216.0979	244.0928
DG	145.0608	173.0557						

Annotated ion spectra of the matched peptides with p≤0.05:

CID No.: **77-1633.6**

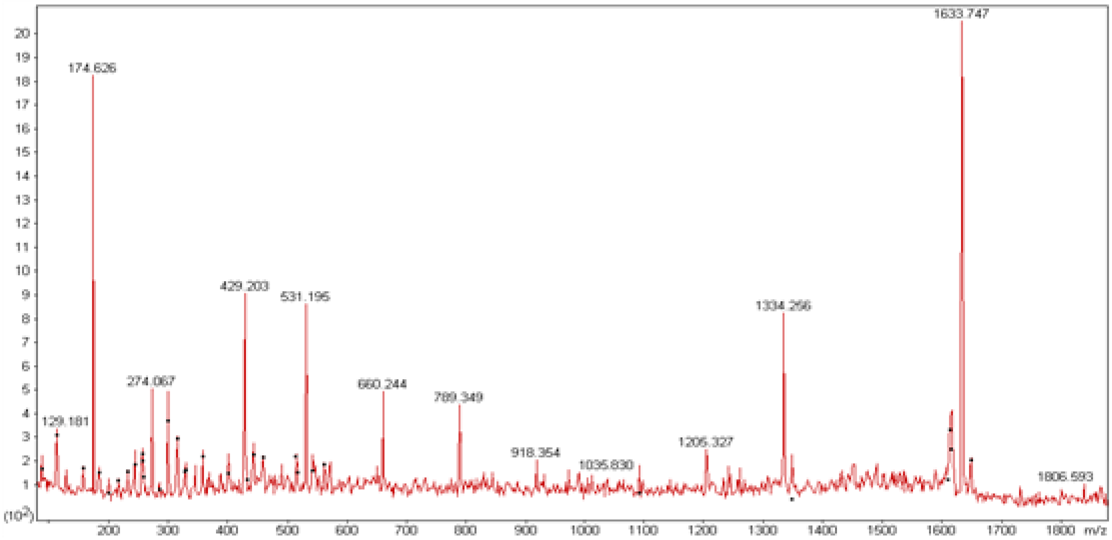

MS/MS Fragmentation of **R.LADELSSEEEAWVR.R**

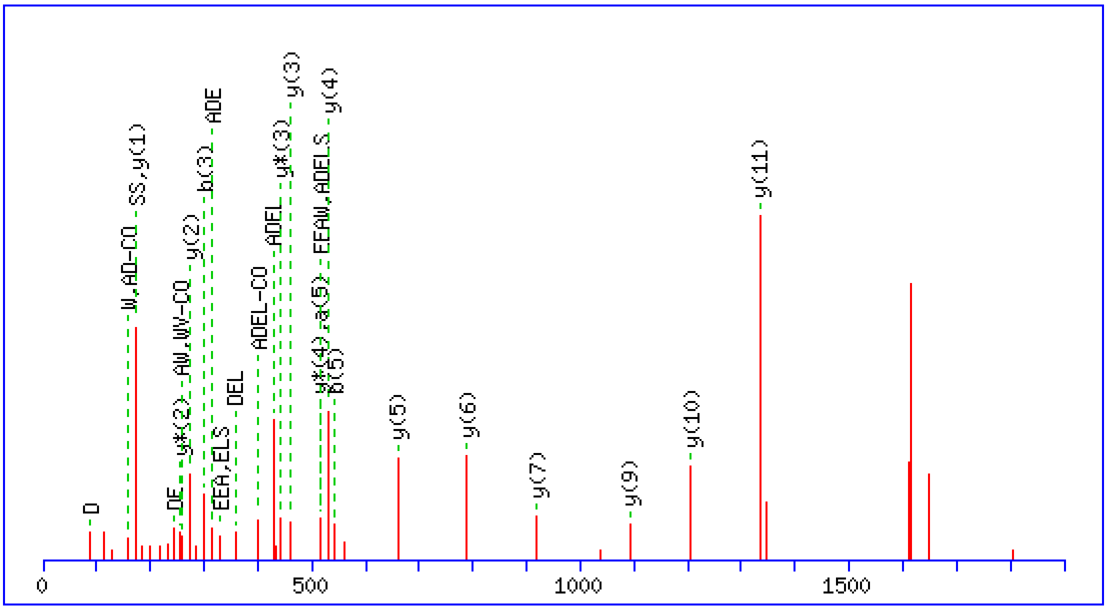

Monoisotopic mass of neutral peptide Mr(calc): 1632.7631

#	Immon.	a	a^0	b	b^0	d	Seq.	v	w	y	y*	y^0	#
1	86.0964	86.0964		114.0913		44.0495	L						14
2	44.0495	157.1335		185.1285			A	1504.6550		1520.6863	1503.6598	1502.6758	13
3	88.0393	272.1605	254.1499	**300.1554**	282.1448	228.1707	D	1389.6281	1388.6329	1449.6492	1432.6227	1431.6387	12
4	102.0550	**401.2031**	383.1925	**429.1980**	411.1874	343.1976	E	1260.5855	1259.5903	***1334.6223***	1317.5957	1316.6117	11
5	86.0964	**514.2871**	496.2766	**542.2821**	524.2715	472.2402	L	1147.5014	1146.5062	***1205.5797***	1188.5531	1187.5691	10
6	60.0444	601.3192	583.3086	629.3141	611.3035	585.3243	S	1060.4694	1059.4742	***1092.4956***	1075.4691	1074.4851	9
7	60.0444	688.3512	670.3406	716.3461	698.3355	672.3563	S	973.4374	972.4421	1005.4636	988.4371	987.4530	8
8	102.0550	817.3938	799.3832	845.3887	827.3781	759.3883	E	844.3948	843.3995	***918.4316***	901.4050	900.4210	7
9	102.0550	946.4364	928.4258	974.4313	956.4207	888.4309	E	715.3522	714.3570	***789.3890***	772.3624	771.3784	6
10	102.0550	1075.4790	1057.4684	1103.4739	1085.4633	1017.4735	E	586.3096	585.3144	***660.3464***	643.3198	642.3358	5
11	44.0495	1146.5161	1128.5055	1174.5110	1156.5004		A	515.2725		***531.3038***	514.2772		4
12	159.0917	1332.5954	1314.5848	1360.5903	1342.5798		W	329.1932		***460.2667***	443.2401		3
13	72.0808	1431.6638	1413.6533	1459.6587	1441.6482	1417.6482	V	230.1248	243.1452	***274.1874***	257.1608		2
14	129.1135						R	74.0237	73.0284	***175.1190***	158.0924		1

Seq	ya	yb	Seq	ya	yb	Seq	ya	yb
AD	159.0764	187.0713	ADE	288.1190	316.1139	ADEL	401.2031	429.1980
ADELS	488.2351	516.2300	ADELSS	575.2671	603.2620	DE	217.0819	245.0768
DEL	330.1660	358.1609	DELS	417.1980	445.1929	DELSS	504.2300	532.2249
DELSSE	633.2726	661.2675	EL	215.1390	243.1339	ELS	302.1710	330.1660
ELSS	389.2031	417.1980	ELSSE	518.2457	546.2406	ELSSEE	647.2883	675.2832
LS	173.1285	201.1234	LSS	260.1605	288.1554	LSSE	389.2031	417.1980
LSSEE	518.2457	546.2406	LSSEEE	647.2883	675.2832	SS	147.0764	175.0713
SSE	276.1190	304.1139	SSEE	405.1616	433.1565	SSEEE	534.2042	562.1991
SSEEEA	605.2413	633.2362	SE	189.0870	217.0819	SEE	318.1296	346.1245
SEEE	447.1722	475.1671	SEEEA	518.2093	546.2042	EE	231.0975	259.0925
EEE	360.1401	388.1351	EEEA	431.1773	459.1722	EEEAW	617.2566	645.2515
EE	231.0975	259.0925	EEA	302.1347	330.1296	EEAW	488.2140	516.2089
EEAWV	587.2824	615.2773	EA	173.0921	201.0870	EAW	359.1714	387.1663
EAWV	458.2398	486.2347	AW	230.1288	258.1237	AWV	329.1972	357.1921
WV	258.1601	286.1550						

Annotated ion spectra of the matched peptides with p≤0.05:

CID No.: **77-2917.4**

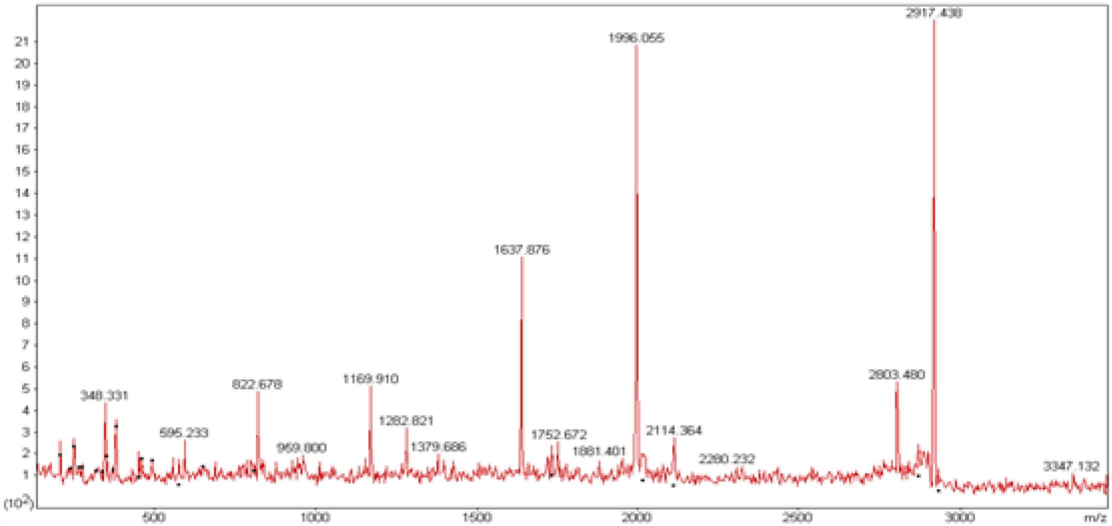

MS/MS Fragmentation of **K.DHQLTLVDGGEDLQNIPLHPLIQPVR.G**

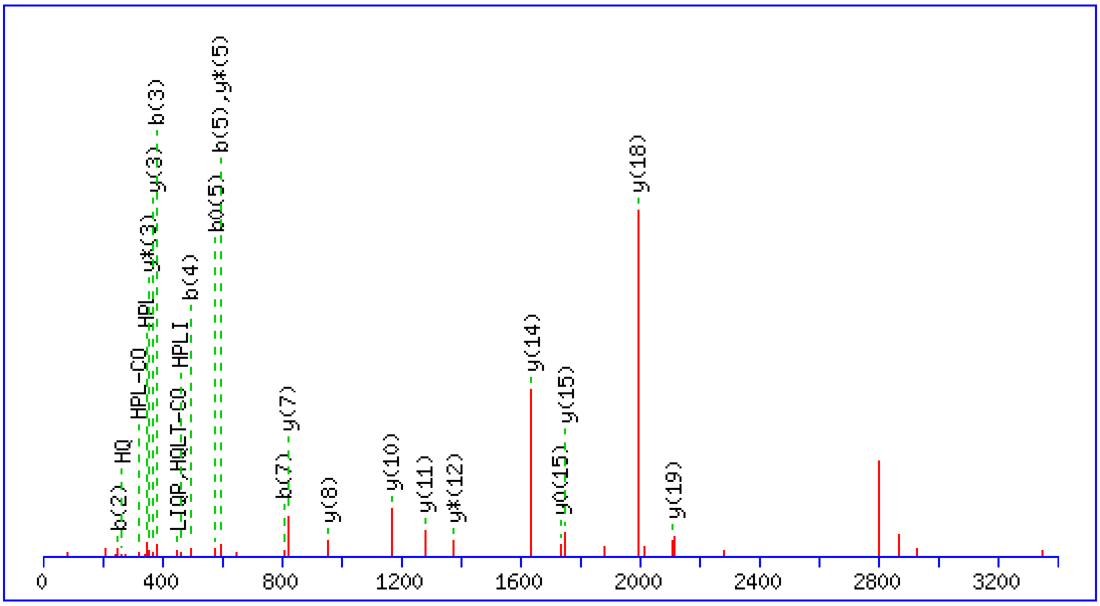

Monoisotopic mass of neutral peptide Mr(calc): 1485.7464

#	Immon.	a	a*	a^0	b	b*	b^0	d	d'	Seq.	v	w	w'	y	y*	y^0	#
1	88.0393	88.0393		70.0287	116.0342		98.0237	44.0495		D							26
2	110.0713	225.0982		207.0877	***253.0931***		235.0826			H	2720.4730			2802.5261	2785.4995	2784.5155	25
3	101.0709	353.1568	336.1302	335.1462	***381.1517***	364.1252	363.1411	296.1353		Q	2592.4144	2591.4192		2665.4672	2648.4406	2647.4566	24
4	86.0964	466.2409	449.2143	448.2303	***494.2358***	477.2092	476.2252	424.1939		L	2479.3304	2478.3351		2537.4086	2520.3821	2519.3980	23
5	74.0600	567.2885	550.2620	549.2780	***595.2835***	578.2569	577.2729	551.2936	553.2729	T	2378.2827	2391.3031	2393.2823	2424.3245	2407.2980	2406.3140	22
6	86.0964	680.3726	663.3461	662.3620	708.3675	691.3410	690.3570	638.3257		L	2265.1986	2264.2034		2323.2769	2306.2503	2305.2663	21
7	72.0808	779.4410	762.4145	761.4304	***807.4359***	790.4094	789.4254	765.4254		V	2166.1302	2179.1506		2210.1928	2193.1662	2192.1822	20
8	88.0393	894.4680	877.4414	876.4574	922.4629	905.4363	904.4523	850.4781		D	2051.1033	2050.1080		***2111.1244***	2094.0978	2093.1138	19
9	30.0338	951.4894	934.4629	933.4789	979.4843	962.4578	961.4738			G				***1996.0974***	1979.0709	1978.0869	18
10	30.0338	1008.5109	991.4843	990.5003	1036.5058	1019.4793	1018.4952			G				1939.0760	1922.0494	1921.0654	17
11	102.0550	1137.5535	1120.5269	1119.5429	1165.5484	1148.5218	1147.5378	1079.5480		E	1808.0177	1807.0225		1882.0545	1865.0280	1864.0439	16
12	88.0393	1252.5804	1235.5539	1234.5699	1280.5753	1263.5488	1262.5648	1208.5906		D	1692.9908	1691.9955		***1753.0119***	1735.9854	1735.0014	15
13	86.0964	1365.6645	1348.6379	1347.6539	1393.6594	1376.6329	1375.6488	1323.6175		L	1579.9067	1578.9115		***1637.9850***	1620.9584		14
14	101.0709	1493.7231	1476.6965	1475.7125	1521.7180	1504.6914	1503.7074	1436.7016		Q	1451.8481	1450.8529		1524.9009	1507.8744		13
15	87.0553	1607.7660	1590.7394	1589.7554	1635.7609	1618.7344	1617.7503	1564.7602		N	1337.8052	1336.8100		1396.8423	1379.8158		12
16	86.0964	1720.8501	1703.8235	1702.8395	1748.8450	1731.8184	1730.8344	1692.8188	1706.8344	I	1224.7212	1237.7416	1251.7572	***1282.7994***	1265.7729		11

**17**	70.0651	1817.9028	1800.8763	1799.8923	1845.8977	1828.8712	1827.8872	1791.8872		**P**	1127.6684	1126.6731		*1169.7153*	1152.6888		**10**
**18**	86.0964	1930.9869	1913.9603	1912.9763	1958.9818	1941.9552	1940.9712	1888.9399		**L**	1014.5843	1013.5891		1072.6626	1055.6360		**9**
**19**	110.0713	2068.0458	2051.0192	2050.0352	2096.0407	2079.0142	2078.0301			**H**	877.5254			*959.5785*	942.5520		**8**
**20**	70.0651	2165.0986	2148.0720	2147.0880	2193.0935	2176.0669	2175.0829	2139.0829		**P**	780.4727	779.4774		*822.5196*	805.4931		**7**
**21**	86.0964	2278.1826	2261.1561	2260.1721	2306.1775	2289.1510	2288.1670	2236.1357		**L**	667.3886	666.3933		725.4668	708.4403		**6**
**22**	86.0964	2391.2667	2374.2401	2373.2561	2419.2616	2402.2351	2401.2510	2363.2354	2377.2510	**I**	554.3045	567.3249	581.3406	612.3828	595.3562		**5**
**23**	101.0709	2519.3253	2502.2987	2501.3147	2547.3202	2530.2936	2529.3096	2462.3038		**Q**	426.2459	425.2507		499.2987	482.2722		**4**
**24**	70.0651	2616.3780	2599.3515	2598.3675	2644.3729	2627.3464	2626.3624	2590.3624		**P**	329.1932	328.1979		*371.2401*	354.2136		**3**
**25**	72.0808	2715.4464	2698.4199	2697.4359	2743.4414	2726.4148	2725.4308	2701.4308		**V**	230.1248	243.1452		274.1874	257.1608		**2**
**26**	129.1135									**R**	74.0237	73.0284		175.1190	158.0924		**1**

Seq	ya	yb	Seq	ya	yb	Seq	ya	yb
**HQ**	238.1299	266.1248	**HQL**	351.2139	379.2088	**HQLT**	452.2616	480.2565
**HQLTL**	565.3457	593.3406	**HQLTLV**	664.4141	692.4090	**QL**	214.1550	242.1499
**QLT**	315.2027	343.1976	**QLTL**	428.2867	456.2817	**QLTLV**	527.3552	555.3501
**QLTLVD**	642.3821	670.3770	**QLTLVDG**	699.4036	727.3985	**LT**	187.1441	215.1390
**LTL**	300.2282	328.2231	**LTLV**	399.2966	427.2915	**LTLVD**	514.3235	542.3184
**LTLVDG**	571.3450	599.3399	**LTLVDGG**	628.3665	656.3614	**TL**	187.1441	215.1390
**TLV**	286.2125	314.2074	**TLVD**	401.2395	429.2344	**TLVDG**	458.2609	486.2558
**TLVDGG**	515.2824	543.2773	**TLVDGGE**	644.3250	672.3199	**LV**	185.1648	213.1598
**LVD**	300.1918	328.1867	**LVDG**	357.2132	385.2082	**LVDGG**	414.2347	442.2296
**LVDGGE**	543.2773	571.2722	**LVDGGED**	658.3042	686.2992	**VD**	187.1077	215.1026
**VDG**	244.1292	272.1241	**VDGG**	301.1506	329.1456	**VDGGE**	430.1932	458.1882
**VDGGED**	545.2202	573.2151	**VDGGEDL**	658.3042	686.2992	**DG**	145.0608	173.0557
**DGG**	202.0822	230.0771	**DGGE**	331.1248	359.1197	**DGGED**	446.1518	474.1467
**DGGEDL**	559.2358	587.2307	**DGGEDLQ**	687.2944	715.2893	**GG**	87.0553	115.0502
**GGE**	216.0979	244.0928	**GGED**	331.1248	359.1197	**GGEDL**	444.2089	472.2038
**GGEDLQ**	572.2675	600.2624	**GGEDLQN**	686.3104	714.3053	**GE**	159.0764	187.0713
**GED**	274.1034	302.0983	**GEDL**	387.1874	415.1823	**GEDLQ**	515.2460	543.2409
**GEDLQN**	629.2889	657.2838	**ED**	217.0819	245.0768	**EDL**	330.1660	358.1609
**EDLQ**	458.2245	486.2195	**EDLQN**	572.2675	600.2624	**EDLQNI**	685.3515	713.3464

**DL**	201.1234	229.1183	**DLQ**	329.1819	357.1769	**DLQN**	443.2249	471.2198
**DLQNI**	556.3089	584.3039	**DLQNIP**	653.3617	681.3566	**LQ**	214.1550	242.1499
**LQN**	328.1979	356.1928	**LQNI**	441.2820	469.2769	**LQNIP**	538.3348	566.3297
**LQNIPL**	651.4188	679.4137	**QN**	215.1139	243.1088	**QNI**	328.1979	356.1928
**QNIP**	425.2507	453.2456	**QNIPL**	538.3348	566.3297	**QNIPLH**	675.3937	703.3886
**NI**	200.1394	228.1343	**NIP**	297.1921	325.1870	**NIPL**	410.2762	438.2711
**NIPLH**	547.3351	575.3300	**NIPLHP**	644.3879	672.3828	**IP**	183.1492	211.1441
**IPL**	296.2333	324.2282	**IPLH**	433.2922	461.2871	**IPLHP**	530.3449	558.3398
**IPLHPL**	643.4290	671.4239	**PL**	183.1492	211.1441	**PLH**	320.2081	348.2030
**PLHP**	417.2609	445.2558	**PLHPL**	530.3449	558.3398	**PLHPLI**	643.4290	671.4239
**LH**	223.1553	251.1503	**LHP**	320.2081	348.2030	**LHPL**	433.2922	461.2871
**LHPLI**	546.3762	574.3711	**LHPLIQ**	674.4348	702.4297	**HP**	207.1240	235.1190
**HPL**	320.2081	348.2030	**HPLI**	433.2922	461.2871	**HPLIQ**	561.3507	589.3457
**HPLIQP**	658.4035	686.3984	**PL**	183.1492	211.1441	**PLI**	296.2333	324.2282
**PLIQ**	424.2918	452.2867	**PLIQP**	521.3446	549.3395	**PLIQPV**	620.4130	648.4079
**LI**	199.1805	227.1754	**LIQ**	327.2391	355.2340	**LIQP**	424.2918	452.2867
**LIQPV**	523.3602	551.3552	**IQ**	214.1550	242.1499	**IQP**	311.2078	339.2027
**IQPV**	410.2762	438.2711	**QP**	198.1237	226.1186	**QPV**	297.1921	325.1870
**PV**	169.1335	197.1285						

Spot No.: **78**

NCBI accession No.: **gi|342872804**

Plant species: ***Fusarium oxysporum Fo5176***

Protein name：**Phospholipase B**

Mascot score: **80** Sequence coverage %: **2**

The number of matched peptides with p≤0.05: **2**

Calculated Mr: **70452** Calculated *Pi*: **4.67**

Annotated MS **spectra:**

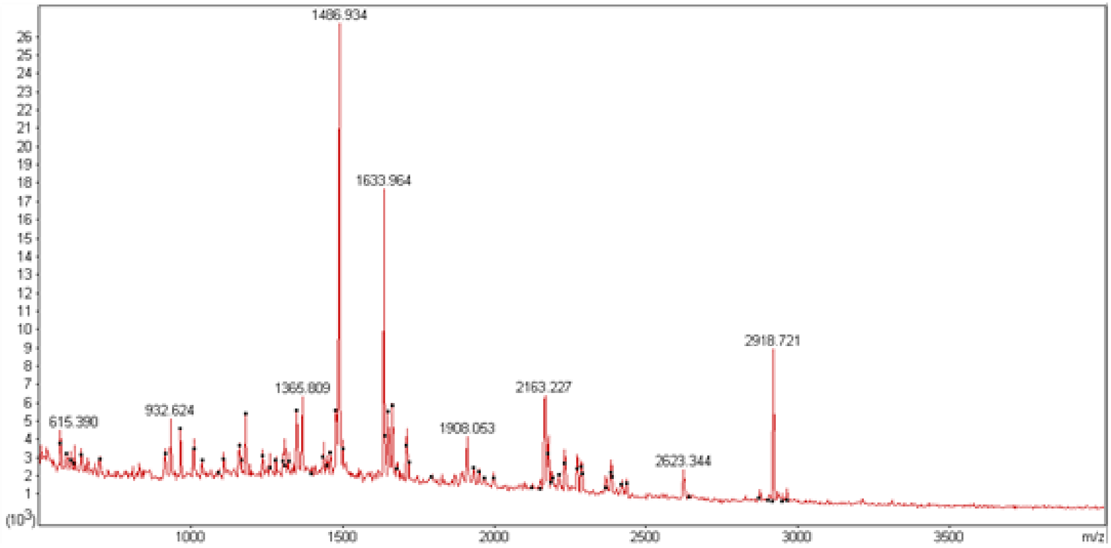

Probability Based Mowse Score:

Ions score is -10*Log(P), where P is the probability that the observed match is a random event.
Individual ions scores > 48 indicate identity or extensive homology (p<0.05).
Protein scores are derived from ions scores as a non-probabilistic basis for ranking protein hits.

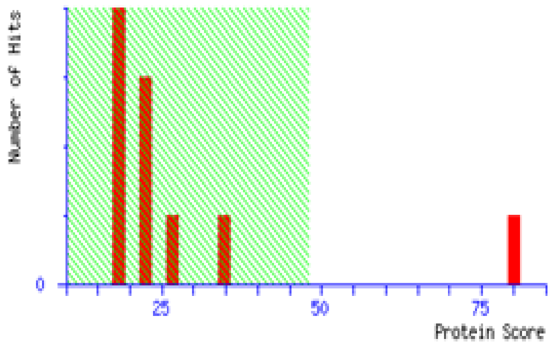

Matched peptide sequences: shown in Bold Red

1	MLGFVALTLW	LSTAIAAPDD	TALIPRVNSV	EIRALPNSPS	GGYAPKVVDC
51	PSTRPKVRLA	DELSSEEEAW	VRRRRNNTID	DLKTFLSRAN	ISGFDAESFV
101	EKHKNNATGL	PNIAIAASGG	GYRALMNGAG	FLSAADSRNN	KTGPISGLLQ
151	SATYLAGLSG	GGWLVGSIFA	NNFSTVPDLQ	SGEKVWRFDR	SIFKGPKSSG
201	ISLLNTAEYW	TEMKDAVDDK	DKGWNTTLTD	YWGRALSYQL	VNAPEGGPSY
251	TFSSIADTSN	FKDADTPFPI	LVADGRAPGE	RVISLNATVY	EFNPYEFGTW
301	DPTTFGFVPT	EYLASNFTNG	SISSKGECVR	GFDQIGFVMG	TSSSLFNQFL
351	LNNITKVGKE	NDIPDIVVKA	IEGVLVGLDQ	DDEDIAQYTP	NPFFGWNPTD
401	KSVNSKDHQL	TLVDGGEDLQ	NIPLHPLIQP	VRGVDIIFAI	DSSADTNNNW
451	PNGTALRATY	DRVGSSIGNG	TQFPSVPSAE	TFVNEKLNQR	PTLFGCDANN
501	FTLSDGEVPP	PLIFYIPNAP	YTYHSNVSTF	DMSYTTAERD	NIILNALNGA
551	TQGNATIDKE	WPTCVACAVM	SRSWWKANET	VPDACKTCFD	RYCWDGKSNN
601	TAVKSYEPEY	IIGGNATADA	ADNAAGARLG	PSWFVSAGVG	AAALFALM

Matched peptides information:

Query	Start - End	Observed	Mr(expt)	Mr(calc)	ppm	M	Score	Expect	Rank	U	Peptide
1	263 - 276	1486.9340	1485.9267	1485.7464	121	0	60	3.4e-05	1	U	K.DADTPFPILVADGR.A
[illegible]	263 - 276	1486.9340	1485.9267	1485.7464	121	0	60	3.4e-05	1	U	K.DADTPFPILVADGR.A

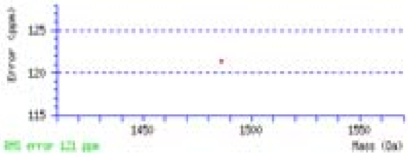

Annotated ion spectra of the matched peptides with p≤0.05:

CID No.: **78-1486.9**

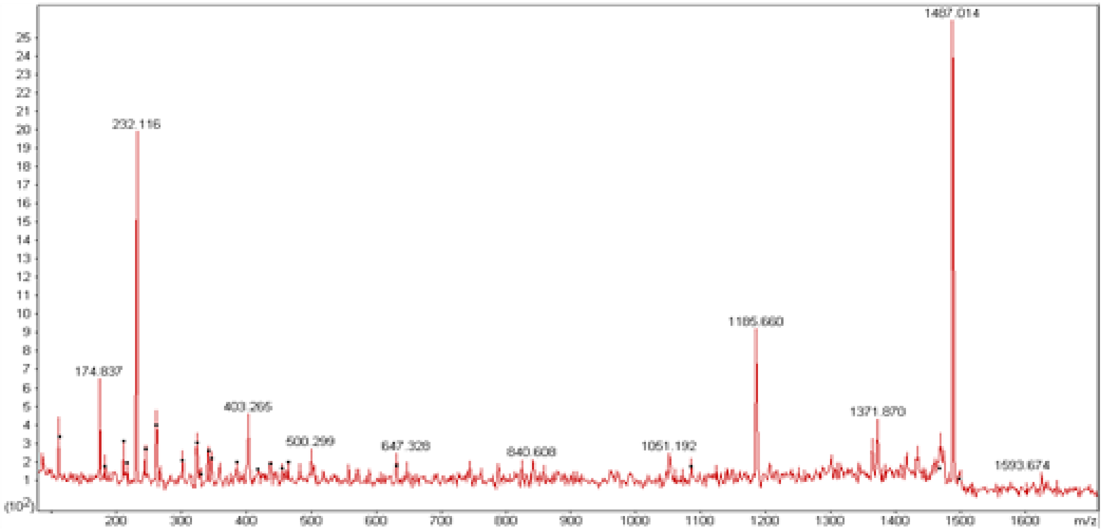

MS/MS Fragmentation of **K.DADTPFPILVADGR.A**

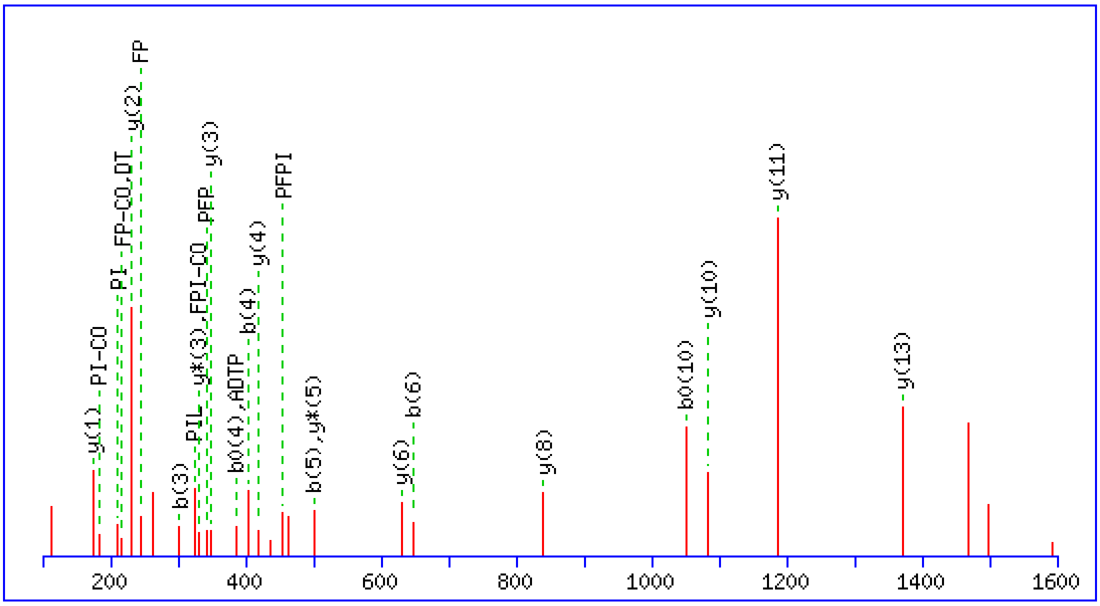

Monoisotopic mass of neutral peptide Mr(calc): 1485.7464

#	Immon.	a	a^0	b	b^0	d	d'	Seq.	v	w	w'	y	y*	y^0	#
1	88.0393	88.0393	70.0287	116.0342	98.0237	44.0495		D							14
2	44.0495	159.0764	141.0659	187.0713	169.0608			A	1355.6954			**1371.7267**	1354.7001	1353.7161	13
3	88.0393	274.1034	256.0928	**302.0983**	284.0877	230.1135		D	1240.6685	1239.6732		1300.6896	1283.6630	1282.6790	12
4	74.0600	375.1510	357.1405	**403.1460**	385.1354	359.1561	361.1354	T	1139.6208	1152.6412	1154.6204	**1185.6626**	1168.6361	1167.6521	11
5	70.0651	472.2038	454.1932	**500.1987**	482.1882	446.1882		P	1042.5680	1041.5728		**1084.6150**	1067.5884	1066.6044	10
6	120.0808	619.2722	601.2617	**647.2671**	629.2566			F	895.4996			987.5622	970.5356	969.5516	9
7	70.0651	716.3250	698.3144	744.3199	726.3093	690.3093		P	798.4468	797.4516		**840.4938**	823.4672	822.4832	8
8	86.0964	829.4090	811.3985	857.4040	839.3934	801.3777	815.3934	I	685.3628	698.3832	712.3988	743.4410	726.4145	725.4305	7
9	86.0964	942.4931	924.4825	970.4880	952.4775	900.4462		L	572.2787	571.2835		**630.3570**	613.3304	612.3464	6
10	72.0808	1041.5615	1023.5510	1069.5564	1051.5459	1027.5459		V	473.2103	486.2307		517.2729	500.2463	499.2623	5
11	44.0495	1112.5986	1094.5881	1140.5936	1122.5830			A	402.1732			**418.2045**	401.1779	400.1939	4
12	88.0393	1227.6256	1209.6150	1255.6205	1237.6099	1183.6358		D	287.1462	286.1510		**347.1674**	330.1408	329.1568	3
13	30.0338	1284.6470	1266.6365	1312.6420	1294.6314			G				**232.1404**	215.1139		2
14	129.1135							R	74.0237	73.0284		**175.1190**	158.0924		1

Seq	ya	yb	Seq	ya	yb	Seq	ya	yb
AD	159.0764	187.0713	ADT	260.1241	288.1190	ADTP	357.1769	385.1718
ADTPF	504.2453	532.2402	ADTPFP	601.2980	629.2930	DT	189.0870	217.0819
DTP	286.1397	314.1347	DTPF	433.2082	461.2031	DTPFP	530.2609	558.2558
DTPFPI	643.3450	671.3399	TP	171.1128	199.1077	TPF	318.1812	346.1761
TPFP	415.2340	443.2289	TPFPI	528.3180	556.3130	TPFPIL	641.4021	669.3970
PF	217.1335	245.1285	PFP	314.1863	342.1812	PFPI	427.2704	455.2653
PFPIL	540.3544	568.3493	PFPILV	639.4228	667.4178	FP	217.1335	245.1285
FPI	330.2176	358.2125	FPIL	443.3017	471.2966	FPILV	542.3701	570.3650
FPILVA	613.4072	641.4021	PI	183.1492	211.1441	PIL	296.2333	324.2282
PILV	395.3017	423.2966	PILVA	466.3388	494.3337	PILVAD	581.3657	609.3606
PILVADG	638.3872	666.3821	IL	199.1805	227.1754	ILV	298.2489	326.2438
ILVA	369.2860	397.2809	ILVAD	484.3130	512.3079	ILVADG	541.3344	569.3293
LV	185.1648	213.1598	LVA	256.2020	284.1969	LVAD	371.2289	399.2238
LVADG	428.2504	456.2453	VA	143.1179	171.1128	VAD	258.1448	286.1397
VADG	315.1663	343.1612	AD	159.0764	187.0713	ADG	216.0979	244.0928
DG	145.0608	173.0557						

Spot No.: **79**

Mascot score: **101**

Species: ***Fusarium oxysporum f. sp. cubense race 4***

Protein name: **Chitinase 1**

NCBI accession No.: **gi| 475673339** Sequence coverage %: **39**

Matched peptides No.: **18** Total peptides No.: **55**

Calculated Mr: **47886** Calculated *p*I: **6.31**

Annotated PMF **spectra:**

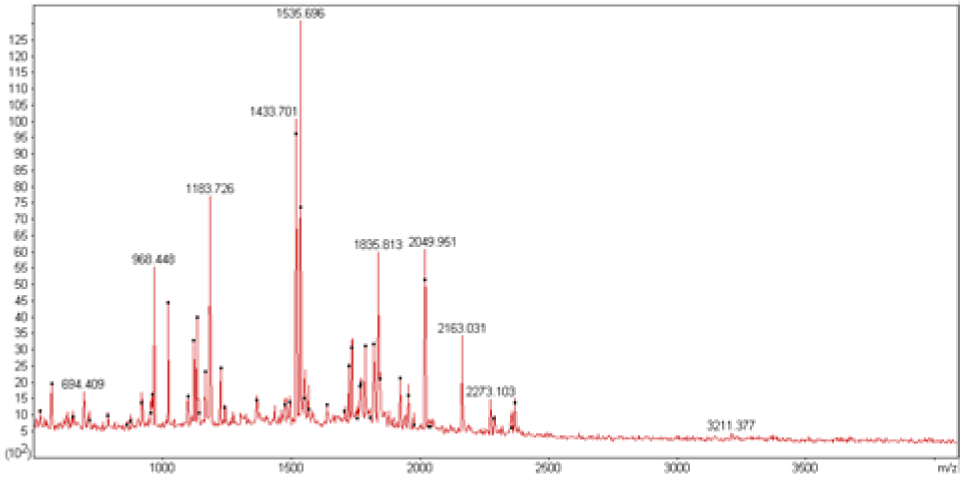

Probability Based Mowse Score:

Protein score is -10*Log(P), where P is the probability that the observed match is a random event.
Protein scores greater than 75 are significant (p<0.05).

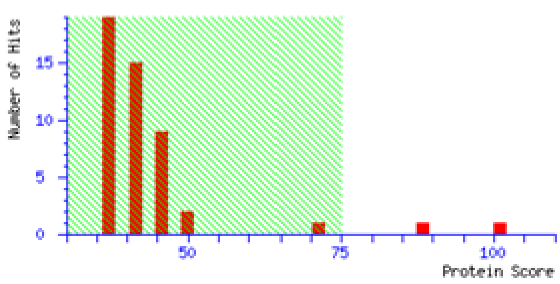

Matched peptide sequences: shown in Bold Red

```
  1 MRVSTLLGLS AYAVAEASCS RNIIYYDQWH TDDLPPKDVT HSVTHVMMSF
 51 ANSSLFTTEP SGKYEPFQPL KQVRALFDHD IKVCLAIGGW GDNAGFDAGL
101 KTDRSRERFA RNVASTLDRL GYDCVDIDME YPGGNGADYK QVVNSKKTYE
151 IQAFPKLLKE IKKFIGSKEL SIAVPGLERD MIAYVPSETP LIEKSVDFVN
201 VMTYDLMNRR DSYTTHHVSV KGAARAIDKY LSLGFPAHKL GIPFYAKWFT
251 TKQGYKCTNP IGCPTELLEN PKDGSDTGKS GSMTFEAANF VSAPTNLTTT
301 PDATCGAGTF FKCATGGCCA ASGWCGDTAA HCGTGCQSAY GHCDGIDLSA
351 SFHEALDKGK TDKANGGQWY WDAPNRIFWS WDTPELIAEK ISLLAKTRGV
401 KSVMAWALAL DSHDWSHLKA MQQGFDRVNA
```

Matched peptides information:

Start	-	End	Observed	Mr(expt)	Mr(calc)	ppm	M	Peptide
22	-	37	2017.9709	2016.9636	2016.9581	2.72	0	R.NIIYYDQWHTDDLPPK.D
64	-	71	1021.5588	1020.5515	1020.5280	23.0	0	K.YEPFQPLK.Q
75	-	82	958.5822	957.5749	957.4920	86.6	0	R.ALFDHDIK.V
83	-	101	1920.9393	1919.9320	1919.9200	6.26	0	K.VCLAIGGWGDNAGFDAGLK.T
112	-	119	875.5063	874.4991	874.4508	55.1	0	R.NVASTLDR.L
147	-	156	1224.6737	1223.6664	1223.6550	9.34	1	K.KTYEIQAFPK.L
148	-	156	1096.6094	1095.6021	1095.5600	38.4	0	K.TYEIQAFPK.L
169	-	179	1183.7258	1182.7185	1182.6608	48.8	0	K.ELSIAVPGLER.D
180	-	194	1721.8104	1720.8031	1720.8593	-32.7	0	R.DMIAYVPSETPLIEK.S + Oxidation (M)
195	-	209	1803.8354	1802.8281	1802.8331	-2.79	0	K.SVDFVNVMTYDLMNR.R
195	-	209	1819.8205	1818.8132	1818.8281	-8.16	0	K.SVDFVNVMTYDLMNR.R + Oxidation (M)
195	-	209	1835.8132	1834.8059	1834.8230	-9.30	0	K.SVDFVNVMTYDLMNR.R + 2 Oxidation (M)
230	-	239	1132.6021	1131.5948	1131.6077	-11.3	0	K.YLSLGFPAHK.L
257	-	272	1842.8769	1841.8696	1841.8652	2.42	0	K.CTNPIGCPTELLENPK.D
364	-	376	1534.6967	1533.6894	1533.6749	9.46	0	K.ANGGQWYWDAPNR.I
377	-	390	1734.8898	1733.8825	1733.8665	9.27	0	R.IFWSWDTPELIAEK.I
420	-	427	952.4906	951.4833	951.4232	63.2	0	K.AMQQGFDR.V
420	-	427	968.4479	967.4407	967.4182	23.3	0	K.AMQQGFDR.V + Oxidation (M)

Spot No.: **80**

Mascot score: **211**

Species: ***Fusarium oxysporum f. sp. cubense race 4***

Protein name: **Alpha-galactosidase 2**

NCBI accession No.: **gi| 475672613** Sequence coverage %: **47**

Matched peptides No.: **35** Total peptides No.: **67**

Calculated Mr: **83037** Calculated *p*I: **5.01**

Annotated PMF **spectra:**

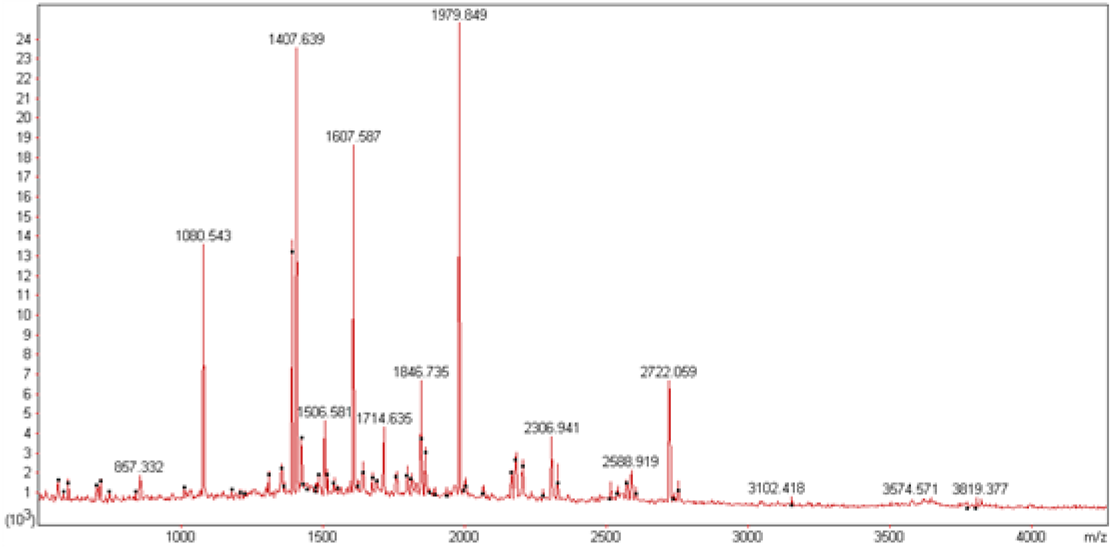

Probability Based Mowse Score:

Protein score is -10*Log(P), where P is the probability that the observed match is a random event.
Protein scores greater than 75 are significant (p<0.05).

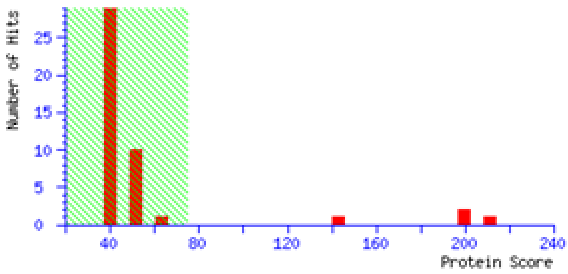

Matched peptide sequences: shown in Bold Red

```
  1 MVLVTSKGIT TAAVLFSQVI SAFAETSDPI RVDGTSFALN GNNVSYRFHV
 51 DNTTGDLIND HYGGPVAEDG ITTEIGPIQG WVNLIGRVRR EFPDHGRGDF
101 RIPAFQLQQA SGTTVTDFRY KSHEVVQGKP GLPGLPSTFG EADDVSTLVV
151 HMYDNYSSIA VDLSYSIFPE YDAIVRSVNI TNQGNATINL RKVSSWSVDL
201 QQDNLDLIEI KGDWAREGMR VRRKVDFGTQ GFQSSTGYSS HLHNPFLALV
251 SSTTTETQGE AWGFSLVYTG SFAVDVEKSS QGLTRAILGL NPLDFSWPLK
301 PEQTFTTPEV VSVFSNKGVG GMSRQFHRLY RKHLMKSKYA EETRPVLLNS
351 WEGLAFDINE TAIEKIAKQS ADLGIKLFVM DDGWFGNKYP RVNDTAGLGD
401 WQPDKSRFPD GLTPLVENVT DLKVANSSDE LKFGIWFEPE MVNPESDLYD
451 KHPDWAIHAG SYPRTETRNQ LVLNLALPEV QEFIIDFVSK VLRESPISYV
501 KWDNNRGIHE TPDPTLNYKY MLGLYHVFET LTSRFPDVLW EGCASGGGRF
551 DPGVLQWFPQ IWTSDDTDAV ERIAIQFGTS LAYPPSAMGA HLSHVPNGNT
601 QRITSVKFRA HVAMMGGSFG VELDPSDLEP EEREQIPGLI ELSEKINPIV
651 ITGDFYRLAL PEETNYPAGQ FISEDGKKVV LFAFQTRATI NNSWPWFRLQ
701 GLDASAKYKV DNNQTVSGST LMNLGIQLRF EGDYDSQVLM IEKQ
```

Matched peptides information:

Start	-	End	Observed	Mr(expt)	Mr(calc)	ppm	M	Peptide
90	-	97	1013.4303	1012.4230	1012.4839	-60.1	1	R.REFPDHGR.G
91	-	97	857.3317	856.3244	856.3828	-68.1	0	R.EFPDHGR.G
102	-	119	1979.0491	1978.0418	1979.0113	-65.6	0	R.IPAFQLQQASGTTVTDFR.Y
192	-	211	2329.9838	2328.9765	2329.2165	-103	1	R.KVSSWSVDLQQDNLDLIEIK.G
193	-	211	2201.8890	2200.8817	2201.1216	-109	0	K.VSSWSVDLQQDNLDLIEIK.G
212	-	216	604.2859	603.2786	603.2765	3.50	0	K.GDWAR.E
279	-	285	748.3720	747.3647	747.3875	-30.5	0	K.SSQGLTR.A
286	-	317	3574.5706	3573.5633	3573.8759	-87.5	0	R.AILGLNPLDFSWPLKPEQTFTTPEVVSVFSNK.G
325	-	328	587.3044	586.2971	586.2976	-0.82	0	R.QFHR.L
377	-	388	1428.5250	1427.5177	1427.6544	-95.7	0	K.LFVMDDGWFGNK.Y
377	-	388	1444.5522	1443.5449	1443.6493	-72.3	0	K.LFVMDDGWFGNK.Y + Oxidation (M)
377	-	391	1844.7063	1843.6990	1843.8716	-93.6	1	K.LFVMDDGWFGNKYPR.V
377	-	391	1860.7121	1859.7048	1859.8665	-86.9	1	K.LFVMDDGWFGNKYPR.V + Oxidation (M)
406	-	423	2000.6681	1999.6608	2000.0579	-98.5	1	K.SRFPDGLTPLVENVTDLK.V
408	-	423	1757.7653	1756.7580	1756.9247	-94.9	0	R.FPDGLTPLVENVTDLK.V
433	-	464	3819.3765	3818.3692	3818.7464	-98.8	1	K.FGIWFEPEMVNPESDLYDKHPDWAIHAGSYPR.T + Oxidation (M)
452	-	464	1506.5805	1505.5732	1505.7164	-95.1	0	K.HPDWAIHAGSYPR.T
452	-	468	1993.0126	1992.0053	1992.9554	-75.3	1	K.HPDWAIHAGSYPRTETR.N
502	-	506	704.2916	703.2843	703.3038	-27.7	0	K.WDNNR.G
507	-	519	1484.5745	1483.5672	1483.7307	-110	0	R.GIHETPDPTLNYK.Y
535	-	549	1607.5867	1606.5794	1606.7198	-87.4	0	R.FPDVLWEGCASGGGR.F
550	-	572	2722.0586	2721.0513	2721.2711	-80.8	0	R.FDPGVLQWFPQIWTSDDTDAVER.I
573	-	602	3152.2151	3151.2078	3150.5669	203	0	R.IAIQFGTSLAYPPSAMGAHLSHVPNGNTQR.I + Oxidation (M)
610	-	633	2572.9111	2571.9038	2572.1574	-98.6	0	R.AHVAMMGGSFGVELDPSDLEPEER.E
610	-	633	2588.9194	2587.9121	2588.1523	-92.8	0	R.AHVAMMGGSFGVELDPSDLEPEER.E + Oxidation (M)
610	-	633	2604.8808	2603.8735	2604.1472	-105	0	R.AHVAMMGGSFGVELDPSDLEPEER.E + 2 Oxidation (M)
634	-	645	1355.5484	1354.5411	1354.7344	-143	0	R.EQIPGLIELSEK.I
646	-	657	1407.6391	1406.6318	1406.7558	-88.1	0	K.INPIVITGDFYR.L
658	-	677	2178.8326	2177.8253	2178.0480	-102	0	R.LALPEETNYPAGQFISEDGK.K
658	-	678	2306.9409	2305.9336	2306.1430	-90.8	1	R.LALPEETNYPAGQFISEDGKK.V
678	-	687	1208.6076	1207.6003	1207.7077	-88.9	1	K.KVVLFAFQTR.A
679	-	687	1080.5430	1079.5357	1079.6128	-71.4	0	K.VVLFAFQTR.A
688	-	698	1391.5577	1390.5504	1390.6782	-91.9	0	R.ATINNSWPWFR.L
730	-	743	1673.5923	1672.5850	1672.7654	-108	0	R.FEGDYDSQVLMIEK.Q

Spot No.: **81**

Mascot score: **112**

Species: ***Fusarium oxysporum f. sp. cubense race 4***

Protein name: **Amidase Family Protein**

NCBI accession No.: **gi| 475668007**

Sequence coverage %: **39**

Matched peptides No.: **18**

Total peptides No.: **64**

Calculated Mr: **63645**

Calculated *p*I: **6.86**

Annotated PMF **spectra:**

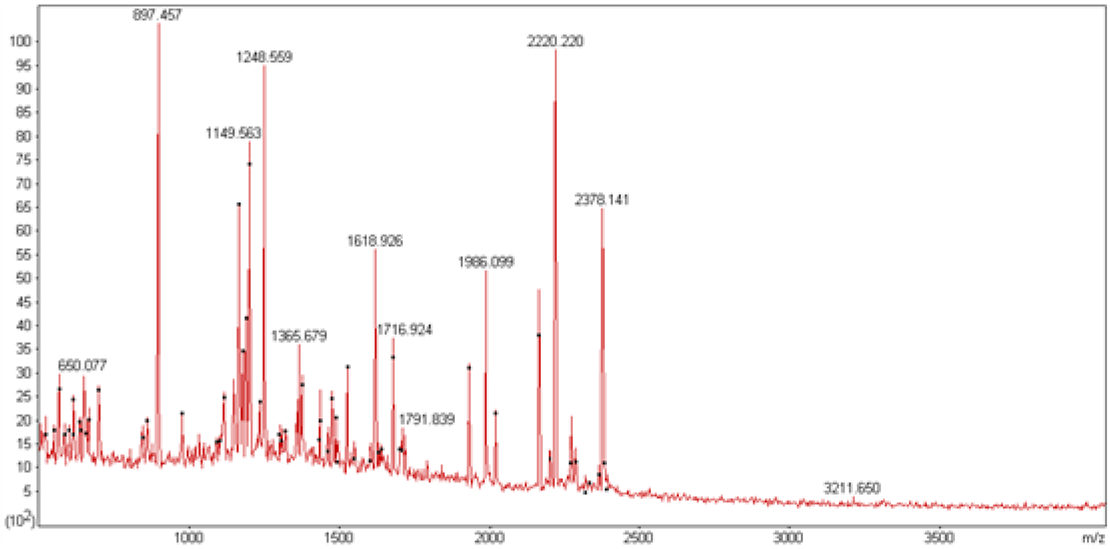

Probability Based Mowse Score:

Protein score is -10*Log(P), where P is the probability that the observed match is a random event.
Protein scores greater than 75 are significant (p<0.05).

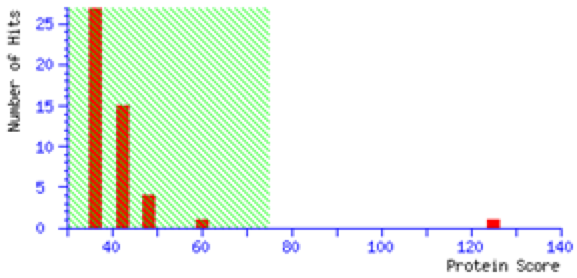

Matched peptide sequences: shown in Bold Red

1	MKLLGLSLAT	GLIAQGVSAT	PKQSKNEPLF	AIQPNMIPLE	KNAGSPDLFP
51	MPDCNGFKLE	EATFTEMQDA	MKAGKLTSVQ	LVTCYLMRTY	QTKEYLNSVL
101	QVNPDAFAIA	AERDAERAKG	KCRGPLHGIP	FTVKDNIATK	DSLETTAGSW
151	ALLGNVVPRD	AHVVKKLRDA	GAVLFGKAAL	SEWADMRSND	YSEGYSARGG
201	QVRSAYNLTV	NPGGSSSGSG	VGVGANVIAF	SLGTETDGSV	INPANRNALV
251	GIKPTVGLTS	RAGVIPESEH	QDSVGCFAKN	VKDAALVLDA	IYGVDKRDNY
301	TEGQKNKTPK	GGYAQYLTDK	KALKGATFGL	PWKSFWALAD	EDMQSQLLEL
351	VDLIKSAGAT	IINGTEITNY	ETIVSPDGWN	WDYGTTRGFP	NESEYTYIKV
401	DFYRNIETYL	SEVKNTNVRN	LEDIVKFNKQ	YDGVEGGYPY	KNGKGIPAFA
451	SGQDGFLASL	KSKGVQDETY	WQALEFCQTS	CRKGINEALT	YKGKKLSGLL
501	VPPQVAQAPQ	IAAQAGYPVI	TIPGGYAKDS	GMPFGLGIMQ	TAWAEAELVK
551	WASAIEDLQR	STDAPSKRRL	PKFLGYLERN	VPVPF	

Matched peptides information:

Start - End	Observed	Mr(expt)	Mr(calc)	ppm	M	Peptide
76 - 88	1599.8270	1598.8197	1598.8160	2.30	0	K.LTSVQLVTCYLMR.T + Oxidation (M)
94 - 113	2220.2205	2219.2132	2219.1222	41.0	0	K.EYLNSVLQVNPDAFAIAAER.D
141 - 159	1986.0992	1985.0919	1985.0218	35.3	0	K.DSLETTAGSWALLGNVVPR.D
178 - 187	1149.5633	1148.5560	1148.5284	24.0	0	K.AALSEWADMR.S
178 - 187	1165.5567	1164.5494	1164.5233	22.4	0	K.AALSEWADMR.S + Oxidation (M)
188 - 198	1248.5590	1247.5517	1247.5054	37.1	0	R.SNDYSEGYSAR.G
247 - 261	1525.9544	1524.9471	1524.8988	31.7	0	R.NALVGIKPTVGLTSR.A
262 - 279	1930.9415	1929.9342	1929.8891	23.4	0	R.AGVIPESEHQDSVGCFAK.N
283 - 297	1618.9264	1617.9191	1617.8726	28.8	1	K.DAALVLDAIYGVDKR.D
311 - 320	1115.3672	1114.3599	1114.5295	-152	0	K.GGYAQYLTDK.K
325 - 333	976.4867	975.4794	975.5178	-39.4	0	K.GATFGLPWK.S
400 - 404	699.2947	698.2874	698.3388	-73.6	0	K.VDFYR.N
430 - 441	1375.6571	1374.6498	1374.6092	29.6	0	K.QYDGVEGGYPYK.N
445 - 461	1678.8943	1677.8870	1677.8726	8.58	0	K.GIPAFASGQDGFLASLK.S
464 - 482	2378.1411	2377.1338	2377.0103	51.9	0	K.GVQDETYWQALEFCQTSCR.K
529 - 550	2367.2641	2366.2568	2366.1286	54.2	0	K.DSGMPFGLGIMQTAWAEAELVK.W + Oxidation (M)
551 - 560	1188.6239	1187.6166	1187.5935	19.5	0	K.WASAIEDLQR.S
573 - 579	897.4572	896.4500	896.4756	-28.6	0	K.FLGYLER.N

Spot No.: **82**

Mascot score: **98**

Species: ***Fusarium oxysporum f. sp. cubense race 1***

Protein name: **l-amino acid oxidase**

NCBI accession No.: **gi| 477517233**

Sequence coverage %: **35**

Matched peptides No.: **23**

Total peptides No.: **83**

Calculated Mr: **67814**

Calculated *p*I: **5.69**

Annotated PMF **spectra:**

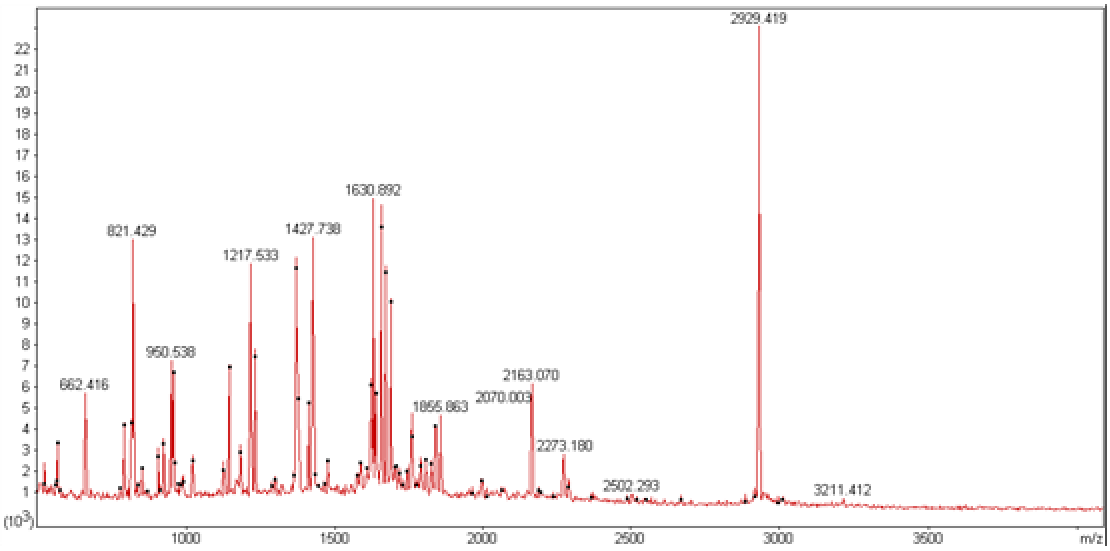

Probability Based Mowse Score:

Protein score is -10*Log(P), where P is the probability that the observed match is a random event.
Protein scores greater than 75 are significant (p<0.05).

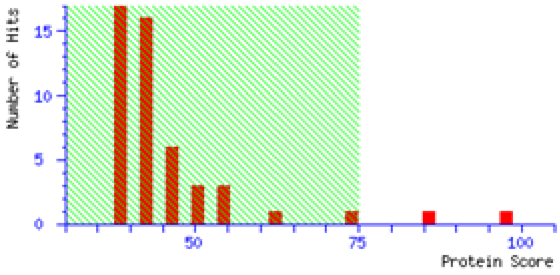

Matched peptide sequences: shown in **Bold Red**

1	MVK**FTDEPWG**	**ASVLVLLFLS**	**NAHGTK**LPLK	AQQTHLCADK	PQMQNFDSVG
51	AWFDDVAKLN	CTSVSKAPNA	SIAIVGGGVS	GLTTALMLDS	IGLHNWDIIE
101	ASDRVGGRFR	**TKFVGGTKEF**	**AEMGPMRLPY**	**TVTYK**SDNST	YEYTEHRLTF
151	QLAETLNEMN	GNDSKWKVDF	ISWIQHHPNE	LIAWGTGRHP	DGR**IPTRADI**
201	**HANSSLGKPP**	**AIVSTEYNET**	**K**HRMNEILKN	ETMLK**AIQAD**	**VWR**SHKFVMS
251	QGYDDWSEQC	MMREAFHASE	NITDAIWTAT	DYDVVWDEMV	HNSNLALDGT
301	KDSLGETEWK	**CVDGGFNRLT**	**DAFIPHVSDR**	LVLNRKIGKL	ESVKGEDGQT
351	QTRLSWYPSV	KNRTFESK**DY**	**DYTIMAVPFT**	**MTRFMALPSF**	**SSVLGRAISE**
401	**AGLR**FKSACK	**VSLLFSER**FW	EKGERPIFGG	YSIPESRPIG	ALYYPVYGLN
451	ESRPGLITHY	R**GGDWSDRYV**	**SFSDEEHVQT**	**VLDAIVSLHG**	**EQARELYTGD**
501	**YER**LCWLQDE	HTATSWCRPD	VEQHNLYIPA	YHQTEHNTIF	IGEHTAPTQA
551	WISSAIYSAA	R**GTIQLLLEL**	**GMVEEAK**EIN	RR**WMGR**WIRD	ETKP	

Matched peptides information:

Start - End	Observed	Mr(expt)	Mr(calc)	ppm	M	Peptide
4 - 26	2502.2934	2501.2861	2501.2955	-3.74	0	K.FTDEPWGASVLVLLFLSNAHGTK.L
111 - 118	837.4418	836.4346	836.4756	-49.1	1	R.TKFVGGTK.E
113 - 127	1656.7644	1655.7571	1655.7800	-13.8	1	K.FVGGTKEFAEMGPMR.L
113 - 127	1672.7480	1671.7407	1671.7749	-20.4	1	K.FVGGTKEFAEMGPMR.L + Oxidation (M)
113 - 127	1688.7530	1687.7457	1687.7698	-14.3	1	K.FVGGTKEFAEMGPMR.L + 2 Oxidation (M)
128 - 135	984.4756	983.4684	983.5328	-65.5	0	R.LPYTVTYK.S
194 - 221	3010.3758	3009.3685	3008.5567	270	1	R.IPTRADIHANSSLGKPPAIVSTEYNETK.H
236 - 243	958.5124	957.5052	957.5032	2.06	0	K.AIQADVWR.S
311 - 318	924.4034	923.3961	923.3920	4.46	0	K.CVDGGFNR.L
319 - 330	1370.7336	1369.7263	1369.6990	19.9	0	R.LTDAFIPHVSDR.L
369 - 383	1823.8696	1822.8623	1822.8270	19.4	0	K.DYDYTIMAVPFTMTR.F
369 - 383	1839.8654	1838.8581	1838.8219	19.7	0	K.DYDYTIMAVPFTMTR.F + Oxidation (M)
369 - 383	1855.8626	1854.8553	1854.8168	20.8	0	K.DYDYTIMAVPFTMTR.F + 2 Oxidation (M)
384 - 396	1411.7520	1410.7447	1410.7330	8.34	0	R.FMALPSFSSVLGR.A
384 - 396	1427.7375	1426.7302	1426.7279	1.65	0	R.FMALPSFSSVLGR.A + Oxidation (M)
397 - 404	816.4532	815.4459	815.4501	-5.11	0	R.AISEAGLR.F
411 - 418	950.5379	949.5306	949.5233	7.69	0	K.VSLLFSER.F
462 - 468	792.3092	791.3019	791.3198	-22.7	0	R.GGDWSDR.Y
469 - 494	2929.4189	2928.4116	2928.4254	-4.69	0	R.YVSFSDEEHVQTVLDAIVSLHGEQAR.E
495 - 503	1145.5067	1144.4994	1144.5036	-3.69	0	R.ELYTGDYER.L
562 - 577	1743.8978	1742.8905	1742.9488	-33.4	0	R.GTIQLLLELGMVEEAK.E
562 - 577	1759.9462	1758.9389	1758.9437	-2.72	0	R.GTIQLLLELGMVEEAK.E + Oxidation (M)
583 - 586	565.2663	564.2590	564.2478	19.8	0	R.WMGR.W + Oxidation (M)

Spot No.: **83**

Mascot score: **150**

Species: ***Fusarium oxysporum f. sp. cubense race 4***

Protein name: **Alpha-galactosidase 2**

NCBI accession No.: **gi| 475672613**

Sequence coverage %: **35**

Matched peptides No.: **27**

Total peptides No.: **62**

Calculated Mr: **83037**

Calculated *p*I: **5.01**

Annotated PMF **spectra:**

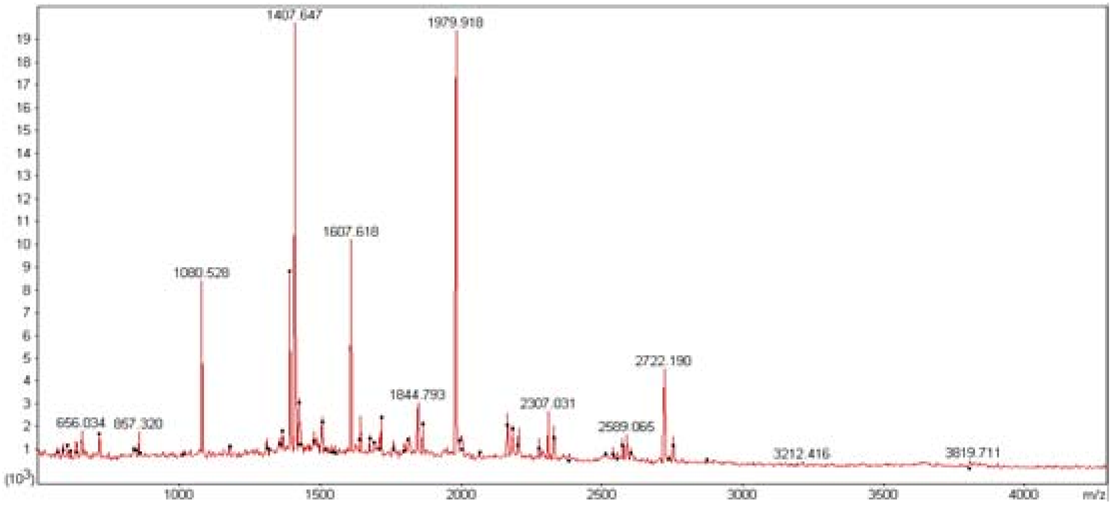

Probability Based Mowse Score:

Protein score is -10*Log(P), where P is the probability that the observed match is a random event.
Protein scores greater than 75 are significant (p<0.05).

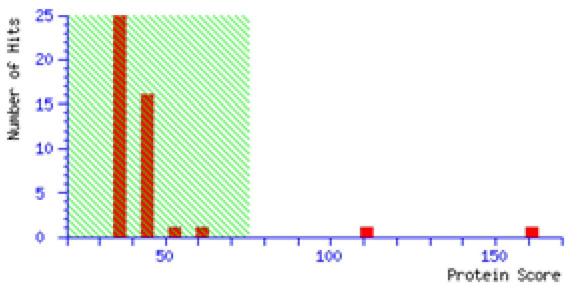

Matched peptide sequences: shown in Bold Red

```
  1 MVLVTSKGIT TAAVLFSQVI SAFAETSDPI RVDGTSFALN GNNVSYRFHV
 51 DNTTGDLIND HYGGPVAEDG ITTEIGPIQG WVNLIGRVRR EFPDHGRGDF
101 RIPAFQLQQA SGTTVTDFRY KSHEVVQGKP GLPGLPSTFG EADDVSTLVV
151 HMYDNYSSIA VDLSYSIFPE YDAIVRSVNI TNQGNATINL RKVSSWSVDL
201 QQDNLDLIEI KGDWAREGMR VRRKVDFGTQ GFQSSTGYSS HLHNPFLALV
251 SSTTTETQGE AWGFSLVYTG SFAVDVEKSS QGLTRAILGL NPLDFSWPLK
301 PEQTFTTPEV VSVFSNKGVG GMSRQFHRLY RKHLMKSKYA EETRPVLLNS
351 WEGLAFDINE TAIEKIAKQS ADLGIKLFVM DDGWFGNKYP RVNDTAGLGD
401 WQPDKSRFPD GLTPLVENVT DLKVANSSDE LKFGIWFEPE MVNPESDLYD
451 KHPDWAIHAG SYPRTETRNQ LVLNLALPEV QEFIIDFVSK VLRESPISYV
501 KWDNNRGIHE TPDPTLNYKY MLGLYHVFET LTSRFPDVLW EGCASGGGRF
551 DPGVLQWFPQ IWTSDDTDAV ERIAIQFGTS LAYPPSAMGA HLSHVPNGNT
601 QRITSVKFRA HVAMMGGSFG VELDPSDLEP EEREQIPGLI ELSEKINPIV
651 ITGDFYRLAL PEETNYPAGQ FISEDGKKVV LFAFQTRATI NNSWPWFRLQ
701 GLDASAKYKV DNNQTVSGST LMNLGIQLRF EGDYDSQVLM IEKQ
```

Matched peptides information:

Start	-	End	Observed	Mr(expt)	Mr(calc)	ppm	M	Peptide
90	-	97	1013.3387	1012.3314	1012.4839	-151	1	R.REFPDHGR.G
91	-	97	857.3204	856.3131	856.3828	-81.4	0	R.EFPDHGR.G
102	-	119	1979.9182	1978.9109	1979.0113	-50.7	0	R.IPAFQLQQASGTTVTDFR.Y
192	-	211	2330.0866	2329.0793	2329.2165	-58.9	1	R.KVSSWSVDLQQDNLDLIEIK.G
212	-	216	604.2408	603.2335	603.2765	-71.2	0	K.GDWAR.E
325	-	328	587.1770	586.1697	586.2976	-218	0	R.QFHR.L
377	-	388	1428.5860	1427.5787	1427.6544	-53.0	0	K.LFVMDDGWFGNK.Y
377	-	391	1844.7926	1843.7853	1843.8716	-46.8	1	K.LFVMDDGWFGNKYPR.V
377	-	391	1860.7449	1859.7376	1859.8665	-69.3	1	K.LFVMDDGWFGNKYPR.V + Oxidation (M)
406	-	423	2000.9543	1999.9470	2000.0579	-55.4	1	K.SRFPDGLTPLVENVTDLK.V
408	-	423	1757.7697	1756.7624	1756.9247	-92.4	0	R.FPDGLTPLVENVTDLK.V
433	-	464	3819.7109	3818.7036	3818.7464	-11.2	1	K.FGIWFEPEMVNPESDLYDKHPDWAIHAGSYPR.T + Oxidation (M)
452	-	464	1506.6064	1505.5991	1505.7164	-77.9	0	K.HPDWAIHAGSYPR.T
452	-	468	1993.8879	1992.8806	1992.9554	-37.5	1	K.HPDWAIHAGSYPRTETR.N
535	-	549	1607.6176	1606.6103	1606.7198	-68.2	0	R.FPDVLWEGCASGGGR.F
550	-	572	2722.1904	2721.1831	2721.2711	-32.3	0	R.FDPGVLQWFPQIWTSDDTDAVER.I
610	-	633	2573.0957	2572.0884	2572.1574	-26.8	0	R.AHVAMMGGSFGVELDPSDLEPEER.E
610	-	633	2589.0654	2588.0581	2588.1523	-36.4	0	R.AHVAMMGGSFGVELDPSDLEPEER.E + Oxidation (M)
610	-	633	2605.0608	2604.0535	2604.1472	-36.0	0	R.AHVAMMGGSFGVELDPSDLEPEER.E + 2 Oxidation (M)
634	-	645	1355.6293	1354.6220	1354.7344	-82.9	0	R.EQIPGLIELSEK.I
646	-	657	1407.6475	1406.6402	1406.7558	-82.2	0	K.INPIVITGDFYR.L
658	-	677	2178.9480	2177.9407	2178.0480	-49.3	0	R.LALPEETNYPAGQFISEDGK.K
658	-	678	2307.0308	2306.0235	2306.1430	-51.8	1	R.LALPEETNYPAGQFISEDGKK.V
679	-	687	1080.5278	1079.5205	1079.6128	-85.5	0	K.VVLFAFQTR.A
688	-	698	1391.5839	1390.5766	1390.6782	-73.0	0	R.ATINNSWPWFR.L
730	-	743	1673.6236	1672.6163	1672.7654	-89.1	0	R.FEGDYDSQVLMIEK.Q

Spot No.: **84**

Mascot score: **82**

Species: ***Fusarium oxysporum f. sp. cubense race 1***

Protein name: **l-amino acid oxidase**

NCBI accession No.: **gi| 477517233**

Sequence coverage %: **25**

Matched peptides No.: **19**

Total peptides No.: **60**

Calculated Mr: **67814**

Calculated *p*I: **5.69**

Annotated PMF **spectra:**

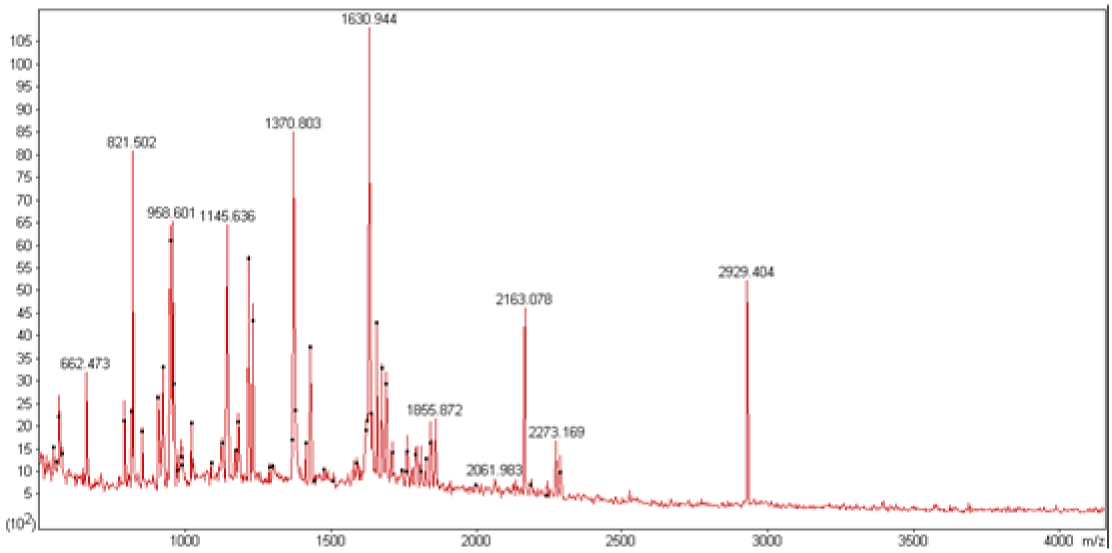

Probability Based Mowse Score:

Protein score is -10*Log(P), where P is the probability that the observed match is a random event.
Protein scores greater than 75 are significant (p<0.05).

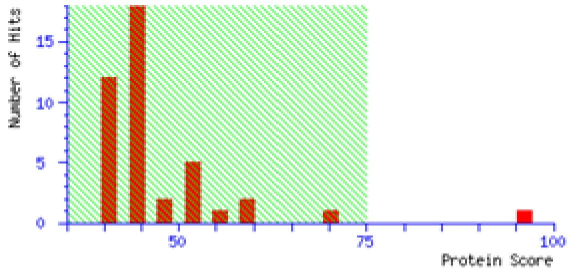

Matched peptide sequences: shown in Bold Red

1 MVKFTDEPWG ASVLVLLFLS NAHGTKLPLK AQQTHLCADK PQMQNFDSVG
51 AWFDDVAKLN CTSVSKAPNA SIAIVGGGVS GLTTALMLDS IGLHNWDIIE
101 ASDRVGGRFR TK**FVGGTKEF** **AEMGPMRLPY** **TVTYK**SDNST YEYTEHRLTF
151 QLAETLNEMN GNDSKWKVDF ISWIQHHPNE LIAWGTGRHP DGRIPTRADI
201 HANSSLGKPP AIVSTEYNET KHRMNEILKN ETMLK**AIQAD** **VWR**SHKFVMS
251 QGYDDWSEQC MMREAFHASE NITDAIWTAT DYDVVWDEMV HNSNLALDGT
301 KDSLGETEWK **CVDGGFNRLT** **DAFIPHVSDR** LVLNRKIGKL ESVKGEDGQT
351 QTRLSWYPSV KNRTFESK**DY** **DYTIMAVPFT** **MTRFMALPSF** **SSVLGRAISE**
401 **AGLR**FKSACK **VSLLFSER**FW EKGERPIFGG YSIPESRPIG ALYYPVYGLN
451 ESRPGLITHY **RGGDWSDRYV** **SFSDEEHVQT** **VLDAIVSLHG** **EQARELYTGD**
501 **YER**LCWLQDE HTATSWCRPD VEQHNLYIPA YHQTEHNTIF IGEHTAPTQA
551 WISSAIYSAA **RGTIQLLLEL** **GMVEEAK**EIN RRWMGRWIRD ETKP

Matched peptides information:

Start	-	End	Observed	Mr(expt)	Mr(calc)	ppm	M	Peptide
113	-	127	1656.8566	1655.8493	1655.7800	41.9	1	K.FVGGTKEFAEMGPMR.L
113	-	127	1672.8276	1671.8203	1671.7749	27.2	1	K.FVGGTKEFAEMGPMR.L + Oxidation (M)
113	-	127	1688.8064	1687.7991	1687.7698	17.4	1	K.FVGGTKEFAEMGPMR.L + 2 Oxidation (M)
128	-	135	984.6421	983.6348	983.5328	104	0	R.LPYTVTYK.S
236	-	243	958.6008	957.5935	957.5032	94.3	0	K.AIQADVWR.S
311	-	318	924.4700	923.4627	923.3920	76.6	0	K.CVDGGFNR.L
319	-	330	1370.8030	1369.7957	1369.6990	70.6	0	R.LTDAFIPHVSDR.L
369	-	383	1823.8939	1822.8866	1822.8270	32.7	0	K.DYDYTIMAVPFTMTR.F
369	-	383	1839.9061	1838.8988	1838.8219	41.8	0	K.DYDYTIMAVPFTMTR.F + Oxidation (M)
369	-	383	1855.8716	1854.8643	1854.8168	25.6	0	K.DYDYTIMAVPFTMTR.F + 2 Oxidation (M)
384	-	396	1411.8488	1410.8415	1410.7330	77.0	0	R.FMALPSFSSVLGR.A
384	-	396	1427.8049	1426.7976	1426.7279	48.9	0	R.FMALPSFSSVLGR.A + Oxidation (M)
397	-	404	816.4888	815.4815	815.4501	38.5	0	R.AISEAGLR.F
411	-	418	950.6230	949.6158	949.5233	97.4	0	K.VSLLFSER.F
462	-	468	792.3802	791.3729	791.3198	67.1	0	R.GGDWSDR.Y
469	-	494	2929.4037	2928.3964	2928.4254	-9.88	0	R.YVSFSDEEHVQTVLDAIVSLHGEQAR.E
495	-	503	1145.6365	1144.6292	1144.5036	110	0	R.ELYTGDYER.L
562	-	577	1743.9656	1742.9583	1742.9488	5.47	0	R.GTIQLLLELGMVEEAK.E
562	-	577	1759.9793	1758.9720	1758.9437	16.1	0	R.GTIQLLLELGMVEEAK.E + Oxidation (M)

Spot No.: **85**

Mascot score: **78**

Species: ***Fusarium oxysporum f. sp. cubense race 4***

Protein name: **Carboxypeptidase cpdS**

NCBI accession No.: **gi| 475670597**

Sequence coverage %: **29**

Matched peptides No.: **14**

Total peptides No.: **58**

Calculated Mr: **64882**

Calculated *p*I: **5.94**

Annotated PMF spectra:

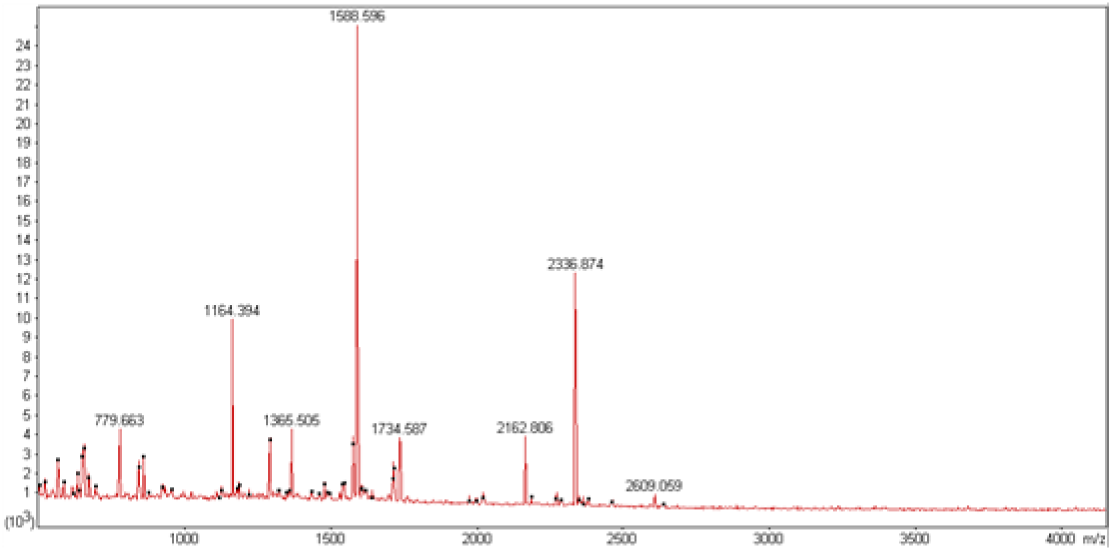

Probability Based Mowse Score:

Protein score is -10*Log(P), where P is the probability that the observed match is a random event.
Protein scores greater than 75 are significant (p<0.05).

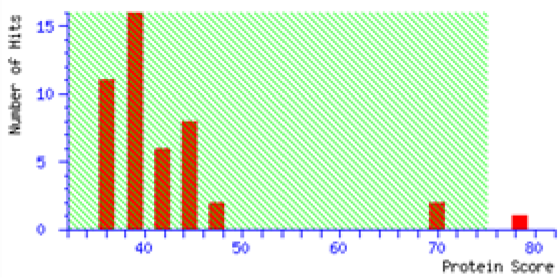

Matched peptide sequences: shown in **Bold Red**

```
  1 MRFSLISTLL AFGTLSQAAF NKGAINAFNR VHPRRYDERR AAAPAPEQPA
 51 FEKRSKSKFL NKHSEKFVVN GSAIPEVKFD VGESYAGLLP ISQDPDEERK
101 LYFWFFPSTN PKAKKEEVVI WLNGGPGCSS LSGLLTENGP FLWQEGTLAP
151 VPNTYSWTNL TNVIWIEQPV GVGYSQGKPN ITNEVELGKQ FIGFWKNFID
201 TFELKGATTY ITGESYAGYY VPYIADAFIT ANDDDYYKLG GVAINDPIIG
251 DGTLQQQAVI FPYIEYWSNL FYLNQTYTNA LRWTHQHCGY EKYLKKYGTF
301 PPPEEKFPVL PDPYADTNPK SNYTCDIFDY AYAAALDSNP CFNIYHITDT
351 CPHVYSQLGI VNQGDYSPPG AKVYFNRTDV KKALNAPIDQ TWYQCTPNKV
401 FGFGDPNSNR SDTSLAPAQN DVLKRVIEHT NNTIIGVGRL DFLLPPNGTL
451 FAIQNATWNG KKGFQKYPQD KQFYVPFHID YNGGRLSEEG IVGQWGEERG
501 LTWYEVQLAG HELPGYTAGA GYRVVEKLLG RIKNLGTIEN FTTQKGNFQG
551 NPHERDFSVV NPLGLPWGHG FTYA
```

(Red/matched regions: 79–100, 190–205, 283–292, 296–320, 383–399, 411–424, 472–523, 534–545.)

Matched peptides information:

Start	-	End	Observed	Mr(expt)	Mr(calc)	ppm	M	Peptide
79	-	99	2336.8737	2335.8664	2336.0808	-91.8	0	K.FDVGESYAGLLPISQDPDEER.K
79	-	100	2464.9405	2463.9332	2464.1758	-98.4	1	K.FDVGESYAGLLPISQDPDEERK.L
190	-	196	925.3358	924.3285	924.4858	-170	0	K.QFIGFWK.N
197	-	205	1126.4359	1125.4286	1125.5706	-126	0	K.NFIDTFELK.G
283	-	292	1345.4104	1344.4031	1344.5670	-122	0	R.WTHQHCGYEK.Y
296	-	306	1292.4542	1291.4469	1291.6448	-153	1	K.KYGTFPPPEEK.F
297	-	306	1164.3935	1163.3862	1163.5499	-141	0	K.YGTFPPPEEK.F
307	-	320	1573.5692	1572.5619	1572.7824	-140	0	K.FPVLPDPYADTNPK.S
383	-	399	2019.7018	2018.6945	2018.9520	-128	0	K.ALNAPIDQTWYQCTPNK.V
411	-	424	1458.5409	1457.5336	1457.7362	-139	0	R.SDTSLAPAQNDVLK.R
472	-	485	1712.6325	1711.6252	1711.8107	-108	0	K.QFYVPFHIDYNGGR.L
486	-	499	1588.5963	1587.5890	1587.7529	-103	0	R.LSEEGIVGQWGEER.G
500	-	523	2609.0589	2608.0516	2608.2710	-84.1	0	R.GLTWYEVQLAGHELPGYTAGAGYR.V
534	-	545	1365.5048	1364.4975	1364.6936	-144	0	K.NLGTIENFTTQK.G

Spot No.: **86**

Mascot score: **216**

Species: ***Fusarium oxysporum f. sp. cubense race 4***

Protein name: **Catalase-peroxidase 2**

NCBI accession No.: **gi| 475672437**

Sequence coverage %: **42**

Matched peptides No.: **33**

Total peptides No.: **64**

Calculated Mr: **85233**

Calculated *p*I: **6.57**

Annotated PMF **spectra:**

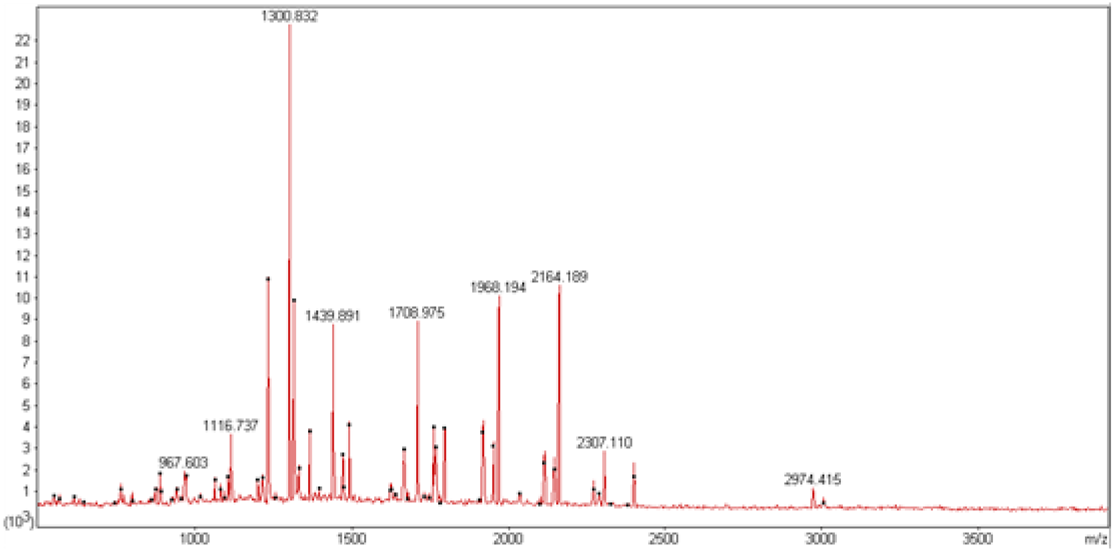

Probability Based Mowse Score:

Protein score is -10*Log(P), where P is the probability that the observed match is a random event.
Protein scores greater than 75 are significant (p<0.05).

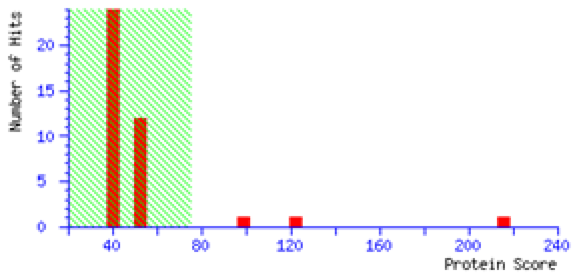

Matched peptide sequences: shown in Bold Red

1 MHVQSLLLAS GLVPLAASQG CPFAKRATDT NLVPPREIPE DFGICRVASN
51 QAGGGTRSRD FWPCALRLDV LRQFSPPYNP LGADFDYTEA FKSLDFAALK
101 KDLNALLTDS QDWWPADHGN YGGLFIRMSW HSAGTYRAMD GRGGSGMGQQ
151 RFAPLDSWPD NQNLDKARRL LWPIKQKYGS KISWADLMVL AGNVALEHSG
201 FETLGFAGGR ADTWEADESI YWGAESTFVP KGNDVRYNGS TDIYERADKL
251 EKPLGATHFG LIYVNPEGPD GSSDPKASAL DIRTAFGRMG MDDEETAALI
301 IGGHTLGKTH GAVPAKNIGP EPMAADLGEM GLGWHNSVNE GNGPDQMTSG
351 LEVIWSTTPT KWSNHFLKSL LGNNWTLVES PAGHKQWEAL NGKLEYPDPF
401 VKGKFRRPTM LTSDLALIND PSYLKICKRW HDNPKELNAA FARAWYKLLH
451 RDLGPVSRYL GPEVAKEKFI WQDPLPERKG DIIGEADISS LKSAILSADG
501 LDVSKLVSTA WNSASTFRGT DKRGGANGAR IALEPQVNWV SNNPKQLKQV
551 LSALKKVQKD FNSKSGSKKV SLADLIVLGG VAAIEKAAQA AGFKDVEVPF
601 TPGRVDATQN QTDLVQFGYL EPLADGFRNY GHGTARARTE EILVDRAALL
651 TLTPPEMTVL VGGLRALNAN YDGSSNGILT EKKGQLTNDF FVNLLSPAYS
701 WAKKDSQGEL WTGTDRATKS VKWTATRADL VFGSHAELRA ISEVYGSADA
751 KEKFVKDFIS AWTKVMNLDR FDVKAEK

Matched peptides information:

Start	-	End	Observed	Mr(expt)	Mr(calc)	ppm	M	Peptide
27	-	36	1083.7202	1082.7129	1082.5720	130	0	R.ATDTNLVPPR.E
37	-	46	1235.7377	1234.7304	1234.5652	134	0	R.EIPEDFGICR.V
47	-	57	1017.6507	1016.6434	1016.4999	141	0	R.VASNQAGGGTR.S
60	-	67	1064.6588	1063.6515	1063.4909	151	0	R.DFWPCALR.L
68	-	72	615.5239	614.5166	614.3752	230	0	R.LDVLR.Q
73	-	92	2307.1104	2306.1031	2306.0532	21.7	0	R.QFSPPYNPLGADFDYTEAFK.S
93	-	100	864.6738	863.6666	863.4752	222	0	K.SLDFAALK.K
102	-	127	2974.4148	2973.4075	2973.4046	0.99	0	K.DLNALLTDSQDWWPADHGNYGGLFIR.M
143	-	151	893.5207	892.5134	892.3621	147	0	R.GGSGMGQQR.F + Oxidation (M)
152	-	166	1759.9020	1758.8947	1758.8213	41.7	0	R.FAPLDSWPDNQNLDK.A
169	-	175	925.7282	924.7209	924.5909	141	1	R.RLLWPIK.Q
211	-	231	2402.1228	2401.1155	2401.0750	16.9	0	R.ADTWEADESIYWGAESTFVPK.G
237	-	246	1217.7362	1216.7289	1216.5360	159	0	R.YNGSTDIYER.A
277	-	283	745.6024	744.5951	744.4130	245	0	K.ASALDIR.T
394	-	402	1107.6756	1106.6683	1106.5648	93.6	0	K.LEYPDPFVK.G
407	-	425	2148.1850	2147.1777	2147.1296	22.4	0	R.RPTMLTSDLALINDPSYLK.I
407	-	425	2164.1885	2163.1812	2163.1245	26.2	0	R.RPTMLTSDLALINDPSYLK.I + Oxidation (M)
436	-	443	891.6459	890.6386	890.4610	199	0	K.ELNAAFAR.A
436	-	447	1439.8910	1438.8837	1438.7357	103	1	K.ELNAAFARAWYK.L
469	-	478	1300.8316	1299.8243	1299.6612	126	0	K.FIWQDPLPER.K
531	-	545	1708.9754	1707.9681	1707.8944	43.2	0	R.IALEPQVNWVSNNPK.Q
569	-	586	1796.1672	1795.1599	1795.0619	43.5	1	K.KVSLADLIVLGGVAAIEK.A
570	-	586	1668.0672	1667.0599	1666.9669	43.9	0	K.VSLADLIVLGGVAAIEK.A
595	-	604	1116.7374	1115.7301	1115.5611	151	0	K.DVEVPFTPGR.V
629	-	636	875.6174	874.6101	874.4046	235	0	R.NYGHGTAR.A
637	-	646	1201.7989	1200.7916	1200.6462	113	1	R.ARTEEILVDR.A
639	-	646	974.6962	973.6889	973.5080	186	0	R.TEEILVDR.A
647	-	665	1952.2189	1951.2116	1951.1176	48.2	0	R.AALLTLTPPEMTVLVGGLR.A
647	-	665	1968.1937	1967.1864	1967.1125	37.6	0	R.AALLTLTPPEMTVLVGGLR.A + Oxidation (M)
704	-	716	1492.8523	1491.8450	1491.6954	100	1	K.KDSQGELWTGTDR.A
705	-	716	1364.7695	1363.7622	1363.6004	119	0	K.DSQGELWTGTDR.A
728	-	739	1314.8446	1313.8373	1313.6728	125	0	R.ADLVFGSHAELR.A
757	-	764	967.6031	966.5958	966.4811	119	0	K.DFISAWTK.V

Spot No.: **87**

Mascot score: **219**

Species: ***Fusarium oxysporum f. sp. cubense race 4***

Protein name: **Catalase-peroxidase 2**

NCBI accession No.: **gi| 475672437**

Sequence coverage %: **41**

Matched peptides No.: **37**

Total peptides No.: **84**

Calculated Mr: **85233**

Calculated *p*I: **6.57**

Annotated PMF **spectra:**

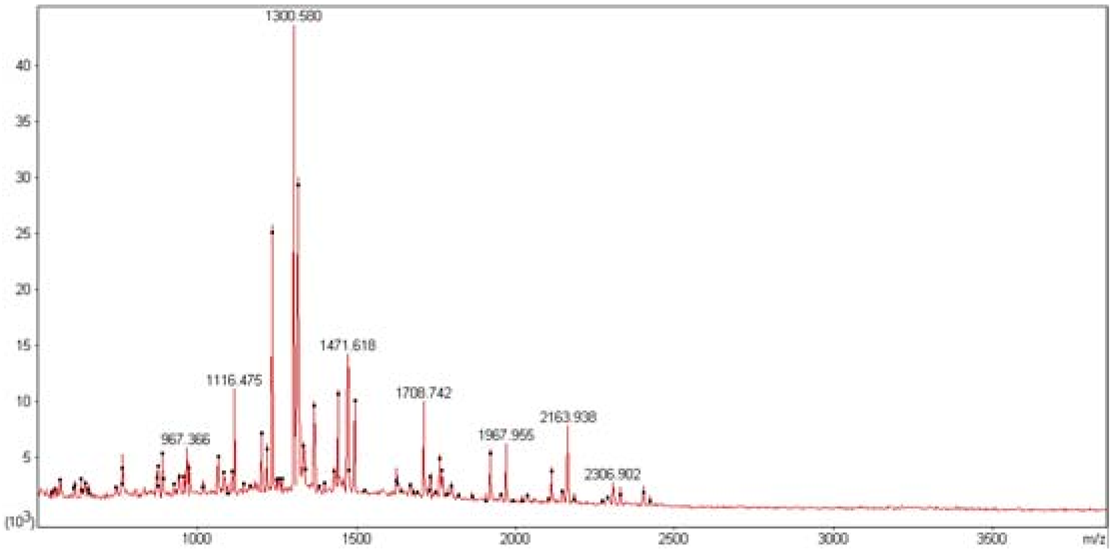

Probability Based Mowse Score:

Protein score is -10*Log(P), where P is the probability that the observed match is a random event.
Protein scores greater than 75 are significant (p<0.05).

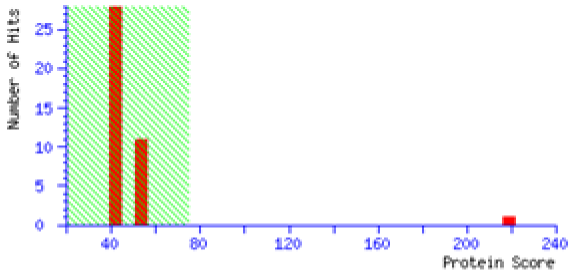

Matched peptide sequences: shown in **Bold Red**

```
  1 MHVQSLLLAS GLVPLAASQG CPFAKRATDT NLVPPREIPE DFGICRVASN
 51 QAGGGTRSRD FWPCALRLDV LRQFSPPYNP LGADFDYTEA FKSLDFAALK
101 KDLNALLTDS QDWWPADHGN YGGLFIRMSW HSAGTYRAMD GRGGSGMGQQ
151 RFAPLDSWPD NQNLDKARRL LWPIKQKYGS KISWADLMVL AGNVALEHSG
201 FETLGFAGGR ADTWEADESI YWGAESTFVP KGNDVRYNGS TDIYERADKL
251 EKPLGATHFG LIYVNPEGPD GSSDPKASAL DIRTAFGRMG MDDEETAALI
301 IGGHTLGKTH GAVPAKNIGP EPMAADLGEM GLGWHNSVNE GNGPDQMTSG
351 LEVIWSTTPT KWSNHFLKSL LGNNWTLVES PAGHKQWEAL NGKLEYPDPF
401 VKGKFRRPTM LTSDLALIND PSYLKICKRW HDNPKELNAA FARAWYKLLH
451 RDLGPVSRYL GPEVAKEKFI WQDPLPERKG DIIGEADISS LKSAILSADG
501 LDVSKLVSTA WNSASTFRGT DKRGGANGAR IALEPQVNWV SNNPKQLKQV
551 LSALKKVQKD FNSKSGSKKV SLADLIVLGG VAAIEKAAQA AGFKDVEVPF
601 TPGRVDATQN QTDLVQFGYL EPLADGFRNY GHGTARARTE EILVDRAALL
651 TLTPPEMTVL VGGLRALNAN YDGSSNGILT EKKGQLTNDF FVNLLSPAYS
701 WAKKDSQGEL WTGTDRATKS VKWTATRADL VFGSHAELRA ISEVYGSADA
751 KEKFVKDFIS AWTKVMNLDR FDVKAEK
```

Matched peptides information:

Start	-	End	Observed	Mr(expt)	Mr(calc)	ppm	M	Peptide
27	-	36	1083.4942	1082.4869	1082.5720	-78.6	0	R.ATDTNLVPPR.E
37	-	46	1235.5008	1234.4935	1234.5652	-58.1	0	R.EIPEDFGICR.V
47	-	57	1017.4461	1016.4388	1016.4999	-60.1	0	R.VASNQAGGGTR.S
60	-	67	1064.4210	1063.4137	1063.4909	-72.6	0	R.DFWPCALR.L
68	-	72	615.3749	614.3676	614.3752	-12.2	0	R.LDVLR.Q
73	-	92	2306.9020	2305.8947	2306.0532	-68.7	0	R.QFSPPYNPLGADFDYTEAFK.S
138	-	151	1439.6219	1438.6146	1438.6042	7.28	1	R.AMDGRGGSGMGQQR.F + 2 Oxidation (M)
143	-	151	877.3822	876.3749	876.3872	-14.0	0	R.GGSGMGQQR.F
143	-	151	893.2968	892.2895	892.3821	-104	0	R.GGSGMGQQR.F + Oxidation (M)
152	-	166	1759.6510	1758.6437	1758.8213	-101	0	R.FAPLDSWPDNQNLDK.A
169	-	175	925.4869	924.4797	924.5909	-120	1	R.RLLWPIK.Q
211	-	231	2401.9427	2400.9354	2401.0750	-58.1	0	R.ADTWEADESIYWGAESTFVPK.G
237	-	246	1217.4764	1216.4691	1216.5360	-55.0	0	R.YNGSTDIYER.A
277	-	283	745.4133	744.4060	744.4130	-9.33	0	K.ASALDIR.T
284	-	288	551.3281	550.3208	550.2863	62.6	0	R.TAFGR.M
394	-	402	1107.4458	1106.4385	1106.5648	-114	0	K.LEYPDPFVK.G
407	-	425	2147.9567	2146.9494	2147.1296	-83.9	0	R.RPTMLTSDLALINDPSYLK.I
407	-	425	2163.9376	2162.9303	2163.1245	-89.8	0	R.RPTMLTSDLALINDPSYLK.I + Oxidation (M)
436	-	443	891.4160	890.4087	890.4610	-58.7	0	K.ELNAAFAR.A
469	-	478	1300.5799	1299.5726	1299.6612	-68.1	0	K.FIWQDPLPER.K
469	-	479	1428.6051	1427.5978	1427.7561	-111	1	K.FIWQDPLPERK.G
531	-	545	1708.7415	1707.7342	1707.8944	-93.8	0	R.IALEPQVNWVSNNPK.Q
569	-	586	1795.8607	1794.8534	1795.0819	-127	1	K.KVSLADLIVLGGVAAIEK.A
570	-	586	1667.8038	1666.7965	1666.9869	-114	0	K.VSLADLIVLGGVAAIEK.A
587	-	604	1860.7919	1859.7846	1859.9530	-90.5	1	K.AAQAAGFKDVEVPFTPGR.V
595	-	604	1116.4754	1115.4681	1115.5611	-83.4	0	K.DVEVPFTPGR.V
629	-	636	875.3719	874.3646	874.4046	-45.6	0	R.NYGHGTAR.A
637	-	646	1201.5422	1200.5349	1200.6462	-92.7	1	R.ARTEEILVDR.A
639	-	646	974.4553	973.4480	973.5080	-61.7	0	R.TEEILVDR.A
647	-	665	1951.9878	1950.9805	1951.1176	-70.3	0	R.AALLTLTPPEMTVLVGGLR.A
647	-	665	1967.9550	1966.9477	1967.1125	-83.8	0	R.AALLTLTPPEMTVLVGGLR.A + Oxidation (M)
704	-	716	1492.6264	1491.6191	1491.6954	-51.1	1	K.KDSQGELWTGTDR.A
705	-	716	1364.5451	1363.5378	1363.6004	-45.9	0	K.DSQGELWTGTDR.A
723	-	727	634.2317	633.2244	633.3235	-156	0	K.WTATR.A
728	-	739	1314.5848	1313.5775	1313.6728	-72.5	0	R.ADLVFGSHAELR.A
757	-	764	967.3664	966.3591	966.4811	-126	0	K.DFISAWTK.V
765	-	770	763.3562	762.3489	762.3694	-26.9	0	K.VMNLDR.F + Oxidation (M)

Spot No.: **88**

Mascot score: **249**

Species: ***Fusarium oxysporum f. sp. cubense race 4***

Protein name: **Catalase-peroxidase 2**

NCBI accession No.: **gi| 475672437**

Sequence coverage %: **41**

Matched peptides No.: **39**

Total peptides No.: **84**

Calculated Mr: **85233**

Calculated *p*I: **6.57**

Annotated PMF **spectra:**

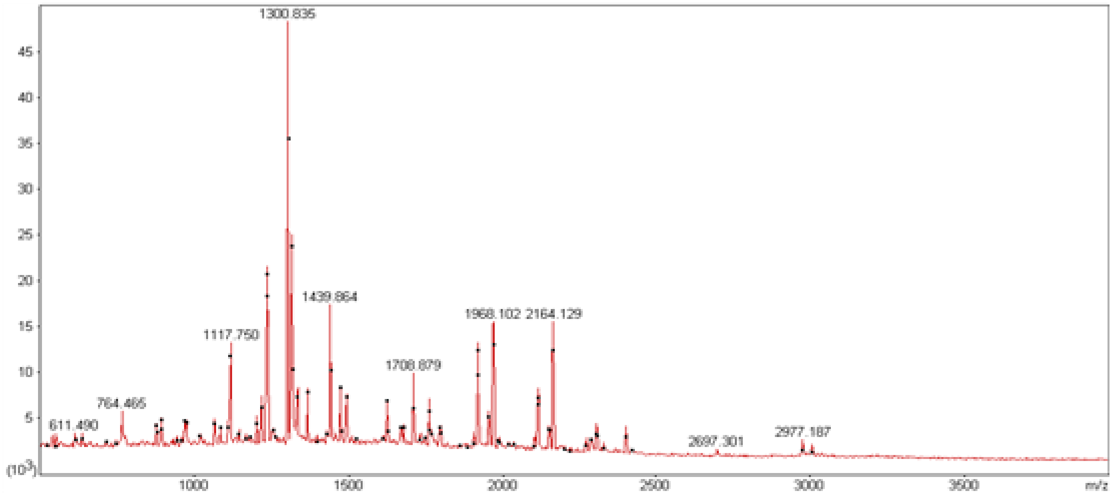

Probability Based Mowse Score:

Protein score is -10*Log(P), where P is the probability that the observed match is a random event.
Protein scores greater than 75 are significant (p<0.05).

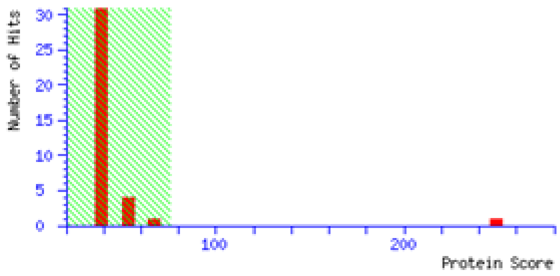

Matched peptide sequences: shown in Bold Red

```
  1 MHVQSLLLAS GLVPLAASQG CPFAKRATDT NLVPPREIPE DFGICRVASN
 51 QAGGGTRSRD FWPCALRLDV LRQFSPPYNP LGADFDYTEA FKSLDFAALK
101 KDLNALLTDS QDWWPADHGN YGGLFIRMSW HSAGTYRAMD GRGGSGMGQQ
151 RFAPLDSWPD NQNLDKARRL LWPIKQKYGS KISWADLMVL AGNVALEHSG
201 FETLGFAGGR ADTWEADESI YWGAESTFVP KGNDVRYNGS TDIYERADKL
251 EKPLGATHFG LIYVNPEGPD GSSDPKASAL DIRTAFGRMG MDDEETAALI
301 IGGHTLGKTH GAVPAKNIGP EPMAADLGEM GLGWHNSVNE GNGPDQMTSG
351 LEVIWSTTPT KWSNHFLKSL LGNNWTLVES PAGHKQWEAL NGKLEYPDPF
401 VKGKFRRPTM LTSDLALIND PSYLKICKRW HDNPKELNAA FARAWYKLLH
451 RDLGPVSRYL GPEVAKEKFI WQDPLPERKG DIIGEADISS LKSAILSADG
501 LDVSKLVSTA WNSASTFRGT DKRGGANGAR IALEPQVNWV SNNPKQLKQV
551 LSALKKVQKD FNSKSGSKKV SLADLIVLGG VAAIEKAAQA AGFKDVEVPF
601 TPGRVDATQN QTDLVQFGYL EPLADGFRNY GHGTARARTE EILVDRAALL
651 TLTPPEMTVL VGGLRALNAN YDGSSNGILT EKKGQLTNDF FVNLLSPAYS
701 WAKKDSQGEL WTGTDRATKS VKWTATRADL VFGSHAELRA ISEVYGSADA
751 KEKFVKDFIS AWTKVMNLDR FDVKAEK
```

Matched peptides information:

Start	-	End	Observed	Mr(expt)	Mr(calc)	ppm	M	Peptide
27	-	36	1083.6983	1082.6910	1082.5720	110	0	R.ATDTNLVPPR.E
37	-	46	1235.7349	1234.7276	1234.5652	132	0	R.EIPEDFGICR.V
47	-	57	1017.6541	1016.6468	1016.4999	145	0	R.VASNQAGGGTR.S
60	-	67	1064.6431	1063.6358	1063.4909	136	0	R.DFWPCALR.L
68	-	72	615.5136	614.5063	614.3752	213	0	R.LDVLR.Q
73	-	92	2307.0814	2306.0741	2306.0532	9.09	0	R.QFSPPYNPLGADFDYTEAFK.S
102	-	127	2974.3414	2973.3341	2973.4046	-23.7	0	K.DLNALLTDSQDWWPADHGNYGGLFIR.M
143	-	151	877.5025	876.4953	876.3872	123	0	R.GGSGMGQQR.F
143	-	151	893.4499	892.4426	892.3821	67.8	0	R.GGSGMGQQR.F + Oxidation (M)
152	-	166	1759.8306	1758.8233	1758.8213	1.15	0	R.FAPLDSWPDNQNLDK.A
211	-	231	2402.0773	2401.0700	2401.0750	-2.08	0	R.ADTWEADESIYWGAESTFVPK.G
237	-	246	1217.7330	1216.7257	1216.5360	156	0	R.YNGSTDIYER.A
277	-	283	745.4472	744.4399	744.4130	36.2	0	K.ASALDIR.T
284	-	288	551.1975	550.1902	550.2863	-175	0	R.TAFGR.M
394	-	402	1107.6843	1106.6770	1106.5648	101	0	K.LEYPDPFVK.G
407	-	425	2148.1275	2147.1202	2147.1296	-4.37	0	R.RPTMLTSDLALINDPSYLK.I
407	-	425	2164.1287	2163.1214	2163.1245	-1.43	0	R.RPTMLTSDLALINDPSYLK.I + Oxidation (M)
436	-	443	891.5706	890.5633	890.4610	115	0	K.ELNAAFAR.A
436	-	447	1439.8640	1438.8567	1438.7357	84.1	1	K.ELNAAFARAWYK.L
469	-	478	1300.8349	1299.8276	1299.6612	128	0	K.FIWQDPLPER.K
469	-	479	1428.8909	1427.8836	1427.7561	89.3	1	K.FIWQDPLPERK.G
480	-	492	1317.8960	1316.8887	1316.6823	157	0	K.GDIIGEADISSLK.S
531	-	545	1708.8793	1707.8720	1707.8944	-13.1	0	R.IALEPQVNWVSNNPK.Q
569	-	586	1796.0385	1795.0312	1795.0819	-28.2	1	K.KVSLADLIVLGGVAAIEK.A
570	-	586	1667.9806	1666.9733	1666.9869	-8.15	0	K.VSLADLIVLGGVAAIEK.A
587	-	604	1860.8967	1859.8894	1859.9530	-34.2	1	K.AAQAAGFKDVEVPFTPGR.V
595	-	604	1116.7027	1115.6954	1115.5611	120	0	K.DVEVPFTPGR.V
605	-	628	2697.3005	2696.2932	2696.3082	-5.56	0	R.VDATQNQTDLVQFGYLEPLADGFR.N
629	-	636	875.5100	874.5027	874.4046	112	0	R.NYGHGTAR.A
637	-	646	1201.7845	1200.7772	1200.6462	109	1	R.ARTEEILVDR.A
639	-	646	974.5810	973.5737	973.5080	67.5	0	R.TEEILVDR.A
647	-	665	1952.1036	1951.0963	1951.1176	-10.9	0	R.AALLTLTPPEMTVLVGGLR.A
647	-	665	1968.1019	1967.0946	1967.1125	-9.10	0	R.AALLTLTPPEMTVLVGGLR.A + Oxidation (M)
704	-	716	1492.8115	1491.8042	1491.6954	73.0	1	K.KDSQGELWTGTDR.A
705	-	716	1364.7727	1363.7654	1363.6004	121	0	K.DSQGELWTGTDR.A
723	-	727	634.4462	633.4389	633.3235	182	0	K.WTATR.A
728	-	739	1314.8647	1313.8574	1313.6728	141	0	R.ADLVFGSHAELR.A
757	-	764	967.5294	966.5222	966.4811	42.5	0	K.DFISAWTK.V
765	-	774	1236.8832	1235.8759	1235.6332	196	1	K.VMNLDRFDVK.A

Spot No.: **89**

Mascot score: **314**

Species: ***Fusarium oxysporum f. sp. cubense race 4***

Protein name: **Catalase-peroxidase 2**

NCBI accession No.: **gi| 475672437** Sequence coverage %: **48**

Matched peptides No.: **39** Total peptides No.: **61**

Calculated Mr: **85233** Calculated *p*I: **6.57**

Annotated PMF **spectra:**

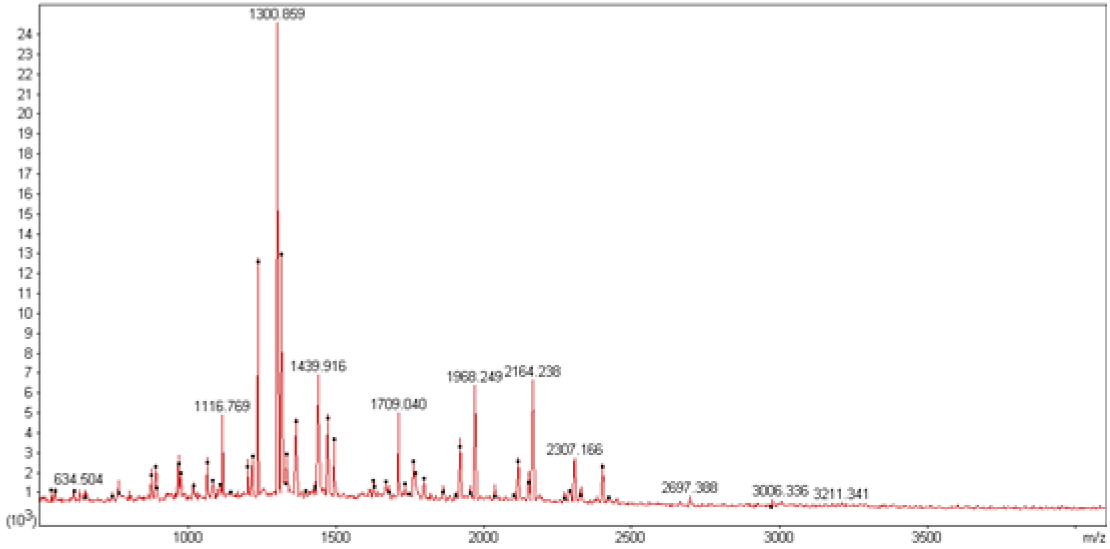

Probability Based Mowse Score:

Protein score is -10*Log(P), where P is the probability that the observed match is a random event.
Protein scores greater than 75 are significant (p<0.05).

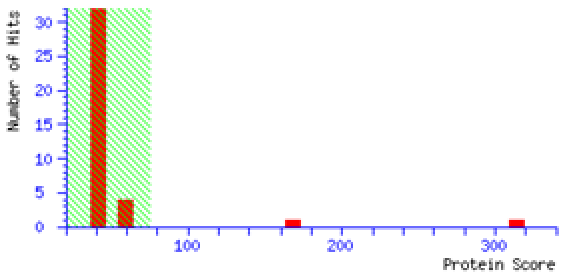

Matched peptide sequences: shown in Bold Red

```
  1 MHVQSLLLAS GLVPLAASQG CPFAKRATDT NLVPPREIPE DFGICRVASN
 51 QAGGGTRSRD FWPCALRLDV LRQFSPPYNP LGADFDYTEA FKSLDFAALK
101 KDLNALLTDS QDWWPADHGN YGGLFIRMSW HSAGTYRAMD GRGGSGMGQQ
151 RFAPLDSWPD NQNLDKARRL LWPIKQKYGS KISWADLMVL AGNVALEHSG
201 FETLGFAGGR ADTWEADESI YWGAESTFVP KGNDVRYNGS TDIYERADKL
251 EKPLGATHFG LIYVNPEGPD GSSDPKASAL DIRTAFGRMG MDDEETAALI
301 IGGHTLGKTH GAVPAKNIGP EPMAADLGEM GLGWHNSVNE GNGPDQMTSG
351 LEVIWSTTPT KWSNHFLKSL LGNNWTLVES PAGHKQWEAL NGKLEYPDPF
401 VKGKFRRPTM LTSDLALIND PSYLKICKRW HDNPKELNAA FARAWYKLLH
451 RDLGPVSRYL GPEVAKEKFI WQDPLPERKG DIIGEADISS LKSAILSADG
501 LDVSKLVSTA WNSASTFRGT DKRGGANGAR IALEPQVNWV SNNPKQLKQV
551 LSALKKVQKD FNSKSGSKKV SLADLIVLGG VAAIEKAAQA AGFKDVEVPF
601 TPGRVDATQN QTDLVQFGYL EPLADGFRNY GHGTARARTE EILVDRAALL
651 TLTPPEMTVL VGGLRALNAN YDGSSNGILT EKKGQLTNDF FVNLLSPAYS
701 WAKKDSQGEL WTGTDRATKS VKWTATRADL VFGSHAELRA ISEVYGSADA
751 KEKFVKDFIS AWTKVMNLDR FDVKAEK
```

Matched peptides information:

Start – End	Observed	Mr(expt)	Mr(calc)	ppm	M	Peptide
27 – 36	1083.7479	1082.7406	1082.5720	156	0	R.ATDTNLVPPR.E
37 – 46	1235.7670	1234.7597	1234.5652	158	0	R.EIPEDFGICR.V
47 – 57	1017.7138	1016.7065	1016.4999	203	0	R.VASNQAGGGTR.S
60 – 67	1064.6957	1063.6884	1063.4909	186	0	R.DFWPCALR.L
68 – 72	615.5299	614.5226	614.3752	240	0	R.LDVLR.Q
73 – 92	2307.1662	2306.1589	2306.0532	45.9	0	R.QFSPPYNPLGADFDYTEAFK.S
102 – 127	2974.4704	2973.4631	2973.4046	19.7	0	K.DLNALLTDSQDWWPADHGNYGGLFIR.M
143 – 151	893.5427	892.5354	892.3821	172	0	R.GGSGMGQQR.F + Oxidation (M)
152 – 166	1759.9623	1758.9550	1758.8213	76.0	0	R.FAPLDSWPDNQNLDK.A
211 – 231	2402.1893	2401.1820	2401.0750	44.6	0	R.ADTWEADESIYWGAESTFVPK.G
237 – 246	1217.7515	1216.7442	1216.5360	171	0	R.YNGSTDIYER.A
277 – 283	745.5842	744.5769	744.4130	220	0	K.ASALDIR.T
284 – 288	551.4576	550.4504	550.2863	298	0	R.TAFGR.M
386 – 402	2034.1669	2033.1596	2033.0258	65.8	1	K.QWEALNGKLEYPDPFVK.G
394 – 402	1107.7698	1106.7625	1106.5648	179	0	K.LEYPDPFVK.G
407 – 425	2148.2741	2147.2668	2147.1296	63.9	0	R.RPTMLTSDLALINDPSYLK.I
407 – 425	2164.2382	2163.2309	2163.1245	49.2	0	R.RPTMLTSDLALINDPSYLK.I + Oxidation (M)
436 – 443	891.6639	890.6566	890.4610	220	0	K.ELNAAFAR.A
436 – 447	1439.9163	1438.9090	1438.7357	120	1	K.ELNAAFARAWYK.L
448 – 451	538.4916	537.4844	537.3387	271	0	K.LLHR.D
469 – 478	1300.8591	1299.8518	1299.6612	147	0	K.FIWQDPLPER.K
469 – 479	1428.9318	1427.9245	1427.7561	118	1	K.FIWQDPLPERK.G
531 – 545	1709.0399	1708.0326	1707.8944	80.9	0	R.IALEPQVNWVSNNPK.Q
569 – 586	1796.2117	1795.2044	1795.0819	68.3	1	K.KVSLADLIVLGGVAAIEK.A
570 – 586	1668.1021	1667.0948	1666.9869	64.7	0	K.VSLADLIVLGGVAAIEK.A
587 – 604	1861.0738	1860.0665	1859.9530	61.0	1	K.AAQAAGFKDVEVPFTPGR.V
595 – 604	1116.7692	1115.7619	1115.5611	180	0	K.DVEVPFTPGR.V
605 – 628	2697.3880	2696.3807	2696.3082	26.9	0	R.VDATQNQTDLVQFGYLEPLADGFR.N
629 – 636	875.6029	874.5956	874.4046	219	0	R.NYGHGTAR.A
637 – 646	1201.8179	1200.8106	1200.6462	137	1	R.ARTEEILVDR.A
639 – 646	974.6950	973.6877	973.5080	185	0	R.TEEILVDR.A
647 – 665	1952.2371	1951.2298	1951.1176	57.5	0	R.AALLTLTPPEMTVLVGGLR.A
647 – 665	1968.2491	1967.2418	1967.1125	65.7	0	R.AALLTLTPPEMTVLVGGLR.A + Oxidation (M)
704 – 716	1492.8706	1491.8633	1491.6954	113	1	K.KDSQGELWTGTDR.A
705 – 716	1364.8034	1363.7961	1363.6004	144	0	K.DSQGELWTGTDR.A
723 – 727	634.5036	633.4963	633.3235	273	0	K.WTATR.A
728 – 739	1314.8818	1313.8745	1313.6728	154	0	R.ADLVFGSHAELR.A
752 – 756	650.2174	649.2101	649.3799	-261	1	K.EKFVK.D
757 – 764	967.6236	966.6163	966.4811	140	0	K.DFISAWTK.V

Spot No.: **90**

Mascot score: **236**

Species: ***Fusarium oxysporum f. sp. cubense race 4***

Protein name: **Catalase-peroxidase 2**

NCBI accession No.: **gi| 475672437**

Sequence coverage %: **42**

Matched peptides No.: **33**

Total peptides No.: **61**

Calculated Mr: **85233**

Calculated *p*I: **6.57**

Annotated PMF **spectra:**

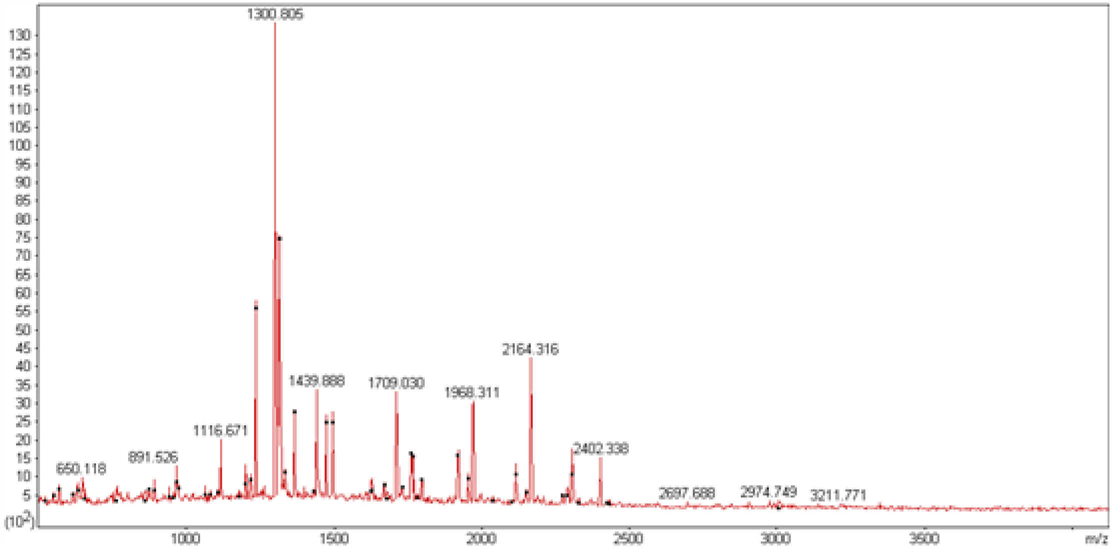

Probability Based Mowse Score:

Protein score is -10*Log(P), where P is the probability that the observed match is a random event.
Protein scores greater than 75 are significant (p<0.05).

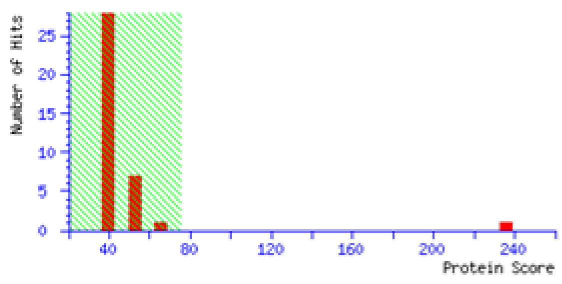

Matched peptide sequences: shown in **Bold Red**

```
  1 MHVQSLLLAS GLVPLAASQG CPFAKRATDT NLVPPREIPE DFGICRVASN
 51 QAGGGTRSRD FWPCALRLDV LRQFSPPYNP LGADFDYTEA FKSLDFAALK
101 KDLNALLTDS QDWWPADHGN YGGLFIRMSW HSAGTYRAMD GRGGSGMGQQ
151 RFAPLDSWPD NQNLDKARRL LWPIKQKYGS KISWADLMVL AGNVALEHSG
201 FETLGFAGGR ADTWEADESI YWGAESTFVP KGNDVRYNGS TDIYERADKL
251 EKPLGATHFG LIYVNPEGPD GSSDPKASAL DIRTAFGRMG MDDEETAALI
301 IGGHTLGKTH GAVPAKNIGP EPMAADLGEM GLGWHNSVNE GNGPDQMTSG
351 LEVIWSTTPT KWSNHFLKSL LGNNWTLVES PAGHKQWEAL NGKLEYPDPF
401 VKGKFRRPTM LTSDLALIND PSYLKICKRW HDNPKELNAA FARAWYKLLH
451 RDLGPVSRYL GPEVAKEKFI WQDPLPERKG DIIGEADISS LKSAILSADG
501 LDVSKLVSTA WNSASTFRGT DKRGGANGAR IALEPQVNWV SNNPKQLKQV
551 LSALKKVQKD FNSKSGSKKV SLADLIVLGG VAAIEKAAQA AGFKDVEVPF
601 TPGRVDATQN QTDLVQFGYL EPLADGFRNY GHGTARARTE EILVDRAALL
651 TLTPPEMTVL VGGLRALNAN YDGSSNGILT EKKGQLTNDF FVNLLSPAYS
701 WAKKDSQGEL WTGTDRATKS VKWTATRADL VFGSHAELRA ISEVYGSADA
751 KEKFVKDFIS AWTKVMNLDR FDVKAEK
```

Matched peptides information:

Start	-	End	Observed	Mr(expt)	Mr(calc)	ppm	M	Peptide
27	-	36	1083.6377	1082.6304	1082.5720	53.9	0	R.ATDTNLVPPR.E
37	-	46	1235.6966	1234.6893	1234.5652	101	0	R.EIPEDFGICR.V
60	-	67	1064.6206	1063.6133	1063.4909	115	0	R.DFWPCALR.L
68	-	72	615.3795	614.3722	614.3752	-4.85	0	R.LDVLR.Q
73	-	92	2307.3063	2306.2990	2306.0532	107	0	R.QFSPPYNPLGADFDYTEAFK.S
102	-	127	2974.7491	2973.7418	2973.4046	113	0	K.DLNALLTDSQDWWPADHGNYGGLFIR.M
143	-	151	893.3922	892.3849	892.3821	3.11	0	R.GGSGMGQQR.F + Oxidation (M)
152	-	166	1759.9803	1758.9730	1758.8213	86.3	0	R.FAPLDSWPDNQNLDK.A
211	-	231	2402.3377	2401.3304	2401.0750	106	0	R.ADTWEADESIYWGAESTFVPK.G
237	-	246	1217.6778	1216.6705	1216.5360	111	0	R.YNGSTDIYER.A
284	-	288	551.3557	550.3485	550.2863	113	0	R.TAFGR.M
394	-	402	1107.6227	1106.6154	1106.5648	45.7	0	K.LEYPDPFVK.G
407	-	425	2148.3478	2147.3405	2147.1298	98.2	0	R.RPTMLTSDLALINDPSYLK.I
407	-	425	2164.3156	2163.3083	2163.1245	85.0	0	R.RPTMLTSDLALINDPSYLK.I + Oxidation (M)
436	-	443	891.5257	890.5185	890.4610	64.6	0	K.ELNAAFAR.A
436	-	447	1439.8880	1438.8807	1438.7357	101	1	K.ELNAAFARAWYK.L
469	-	478	1300.8052	1299.7979	1299.6612	105	0	K.FIWQDPLPER.K
469	-	479	1428.8894	1427.8821	1427.7561	88.3	1	K.FIWQDPLPERK.G
531	-	545	1709.0297	1708.0224	1707.8944	75.0	0	R.IALEPQVNWVSNNPK.Q
569	-	586	1796.2221	1795.2148	1795.0819	74.1	1	K.KVSLADLIVLGGVAAIEK.A
570	-	586	1668.1369	1667.1296	1666.9869	85.6	0	K.VSLADLIVLGGVAAIEK.A
595	-	604	1116.6711	1115.6638	1115.5611	92.0	0	K.DVEVPFTPGR.V
605	-	628	2697.6879	2696.6806	2696.3052	139	0	R.VDATQNQTDLVQFGYLEPLADGFR.N
629	-	636	875.4841	874.4768	874.4046	82.7	0	R.NYGHGTAR.A
637	-	646	1201.7272	1200.7199	1200.6462	61.4	1	R.ARTEEILVDR.A
639	-	646	974.5279	973.5206	973.5080	13.0	0	R.TEEILVDR.A
647	-	665	1952.3297	1951.3224	1951.1176	105	0	R.AALLTLTPPEMTVLVGGLR.A
647	-	665	1968.3114	1967.3041	1967.1125	97.4	0	R.AALLTLTPPEMTVLVGGLR.A + Oxidation (M)
704	-	716	1492.8794	1491.8721	1491.6954	118	1	K.KDSQGELWTGTDR.A
705	-	716	1364.7804	1363.7731	1363.6004	127	0	K.DSQGELWTGTDR.A
723	-	727	634.2480	633.2408	633.3235	-131	0	K.WTATR.A
728	-	739	1314.8139	1313.8066	1313.6728	102	0	R.ADLVFGSHAELR.A
757	-	764	967.4975	966.4902	966.4811	9.45	0	K.DFISAWTK.V

Spot No.: **91**

Mascot score: **86**

Species: ***Fusarium oxysporum f. sp. cubense race 1***

Protein name: **l-amino acid oxidase**

NCBI accession No.: **gi| 477517233** Sequence coverage %: **26**

Matched peptides No.: **19** Total peptides No.: **64**

Calculated Mr: **67814** Calculated *p*I: **5.69**

Annotated PMF **spectra:**

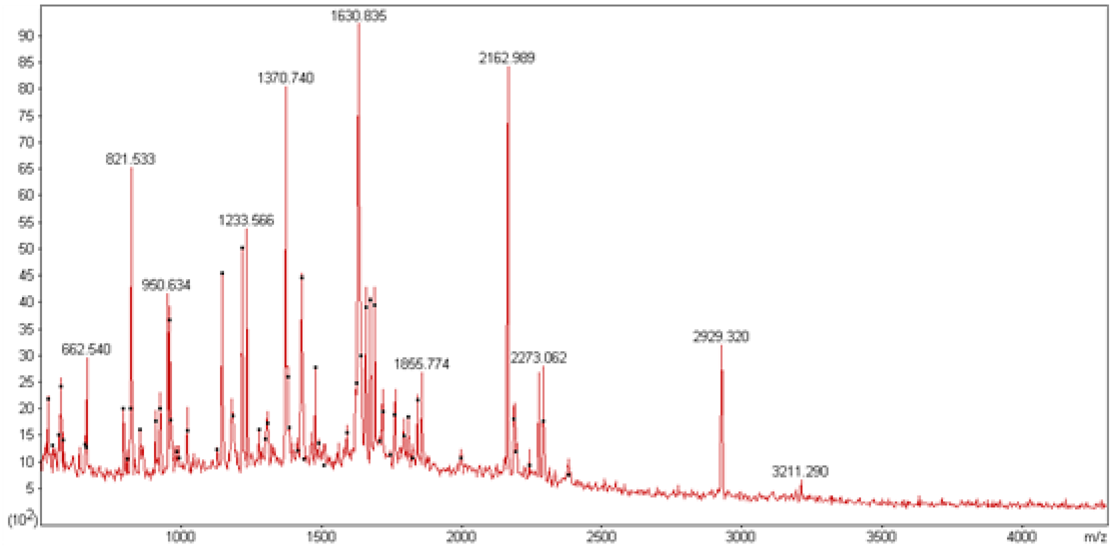

Probability Based Mowse Score:

Protein score is -10*Log(P), where P is the probability that the observed match is a random event.
Protein scores greater than 75 are significant (p<0.05).

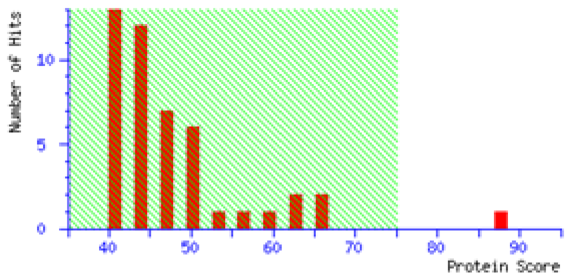

Matched peptide sequences: shown in Bold Red

1 MVKFTDEPWG ASVLVLLFLS NAHGTKLPLK AQQTHLCADK PQMQNFDSVG
51 AWFDDVAKLN CTSVSKAPNA SIAIVGGGVS GLTTALMLDS IGLHNWDIIE
101 ASDRVGGRFR TK**FVGGTKEF** **AEMGPMRLPY** **TVTYK**SDNST YEYTEHRLTF
151 QLAETLNEMN GNDSKWKVDF ISWIQHHPNE LIAWGTGRHP DGRIPTRADI
201 HANSSLGKPP AIVSTEYNET KHRMNEILKN ETMLK**AIQAD** **VWR**SHKFVMS
251 QGYDDWSEQC MMREAFHASE NITDAIWTAT DYDVVWDEMV HNSNLALDGT
301 KDSLGETEWK **CVDGGFNRLT** **DAFIPHVSDR** LVLNRKIGKL ESVKGEDGQT
351 QTRLSWYPSV KNRTFESK**DY** **DYTIMAVPFT** **MTRFMALPSF** **SSVLGRAISE**
401 **AGLR**FKSACK **VSLLFSER**FW EKGERPIFGG YSIPESRPIG ALYYPVYGLN
451 ESRPGLITHY R**GGDWSDRYV** **SFSDEEHVQT** **VLDAIVSLHG** **EQARELYTGD**
501 **YER**LCWLQDE HTATSWCRPD VEQHNLYIPA YHQTEHNTIF IGEHTAPTQA
551 WISSAIYSAA R**GTIQLLLEL** **GMVEEAK**EIN RR**WMGRWIR**D ETKP

Matched peptides information:

Start	-	End	Observed	Mr(expt)	Mr(calc)	ppm	M	Peptide
113	-	127	1656.7789	1655.7716	1655.7800	-5.05	1	K.FVGGTKEFAEMGPMR.L
113	-	127	1672.7169	1671.7096	1671.7749	-39.0	1	K.FVGGTKEFAEMGPMR.L + Oxidation (M)
113	-	127	1688.7081	1687.7008	1687.7698	-40.9	1	K.FVGGTKEFAEMGPMR.L + 2 Oxidation (M)
128	-	135	984.5974	983.5901	983.5328	58.3	0	R.LPYTVTYK.S
236	-	243	958.5856	957.5784	957.5032	78.5	0	K.AIQADVWR.S
311	-	318	924.4823	923.4751	923.3920	90.0	0	K.CVDGGFNR.L
319	-	330	1370.7400	1369.7327	1369.6990	24.6	0	R.LTDAFIPHVSDR.L
369	-	383	1839.8032	1838.7959	1838.8219	-14.1	0	K.DYDYTIMAVPFTMTR.F + Oxidation (M)
369	-	383	1855.7742	1854.7669	1854.8168	-26.9	0	K.DYDYTIMAVPFTMTR.F + 2 Oxidation (M)
384	-	396	1411.7705	1410.7632	1410.7330	21.5	0	R.FMALPSFSSVLGR.A
384	-	396	1427.7428	1426.7355	1426.7279	5.36	0	R.FMALPSFSSVLGR.A + Oxidation (M)
397	-	404	816.5812	815.5739	815.4501	152	0	R.AISEAGLR.F
411	-	418	950.6344	949.6271	949.5233	109	0	K.VSLLFSER.F
462	-	468	792.4645	791.4572	791.3198	174	0	R.GGDWSDR.Y
469	-	494	2929.3199	2928.3126	2928.4254	-38.5	0	R.YVSFSDEEHVQTVLDAIVSLHGEQAR.E
495	-	503	1145.5776	1144.5703	1144.5036	58.3	0	R.ELYTGDYER.L
562	-	577	1743.8093	1742.8020	1742.9488	-84.2	0	R.GTIQLLLELGMVEEAK.E
562	-	577	1759.8664	1758.8591	1758.9437	-48.1	0	R.GTIQLLLELGMVEEAK.E + Oxidation (M)
583	-	589	1020.5305	1019.5232	1019.5123	10.7	1	R.WMGRWIR.D + Oxidation (M)

Spot No.: **92**

Mascot score: **250**

Species: ***Fusarium oxysporum f. sp. cubense race 4***

Protein name: **Catalase-peroxidase 2**

NCBI accession No.: **gi| 475672437**

Sequence coverage %: **49**

Matched peptides No.: **41**

Total peptides No.: **87**

Calculated Mr: **85233**

Calculated *p*I: **6.57**

Annotated PMF **spectra:**

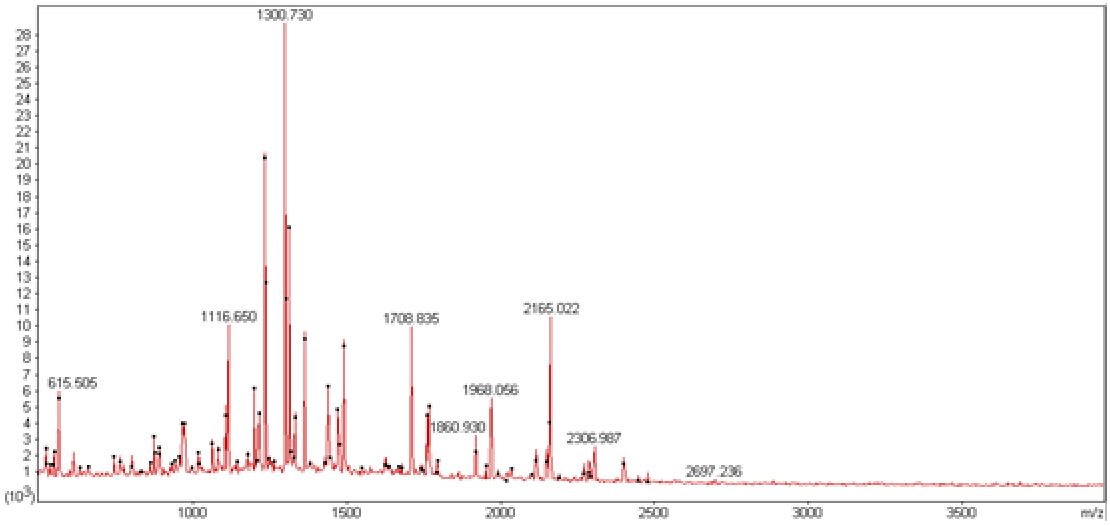

Probability Based Mowse Score:

Protein score is -10*Log(P), where P is the probability that the observed match is a random event.
Protein scores greater than 75 are significant (p<0.05).

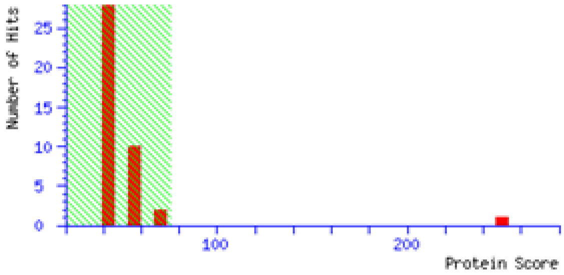

Matched peptide sequences: shown in **Bold Red**

```
  1 MHVQSLLLAS GLVPLAASQG CPFAKRATDT NLVPPREIPE DFGICRVASN
 51 QAGGGTRSRD FWPCALRLDV LRQFSPPYNP LGADFDYTEA FKSLDFAALK
101 KDLNALLTDS QDWWPADHGN YGGLFIRMSW HSAGTYRAMD GRGGSGMGQQ
151 RFAPLDSWPD NQNLDKARRL LWPIKQKYGS KISWADLMVL AGNVALEHSG
201 FETLGFAGGR ADTWEADESI YWGAESTFVP KGNDVRYNGS TDIYERADKL
251 EKPLGATHFG LIYVNPEGPD GSSDPKASAL DIRTAFGRMG MDDEETAALI
301 IGGHTLGKTH GAVPAKNIGP EPMAADLGEM GLGWHNSVNE GNGPDQMTSG
351 LEVIWSTTPT KWSNHFLKSL LGNNWTLVES PAGHKQWEAL NGKLEYPDPF
401 VKGKFRRPTM LTSDLALIND PSYLKICKRW HDNPKELNAA FARAWYKLLH
451 RDLGPVSRYL GPEVAKEKFI WQDPLPERKG DIIGEADISS LKSAILSADG
501 LDVSKLVSTA WNSASTFRGT DKRGGANGAR IALEPQVNWV SNNPKQLKQV
551 LSALKKVQKD FNSKSGSKKV SLADLIVLGG VAAIEKAAQA AGFKDVEVPF
601 TPGRVDATQN QTDLVQFGYL EPLADGFRNY GHGTARARTE EILVDRAALL
651 TLTPPEMTVL VGGLRALNAN YDGSSNGILT EKKGQLTNDF FVNLLSPAYS
701 WAKKDSQGEL WTGTDRATKS VKWTATRADL VFGSHAELRA ISEVYGSADA
751 KEKFVKDFIS AWTKVMNLDR FDVKAEK
```

Matched peptides information:

Start	-	End	Observed	Mr(expt)	Mr(calc)	ppm	M	Peptide
27	-	36	1083.6332	1082.6259	1082.5720	49.8	0	R.ATDTNLVPPR.E
37	-	46	1235.6343	1234.6270	1234.5652	50.1	0	R.EIPEDFGICR.V
47	-	57	1017.5933	1016.5860	1016.4999	84.7	0	R.VASNQAGGGTR.S
60	-	67	1064.5898	1063.5825	1063.4909	86.1	0	R.DFWPCALR.L
68	-	72	615.5055	614.4982	614.3752	200	0	R.LDVLR.Q
73	-	92	2306.9875	2305.9802	2306.0532	-31.6	0	R.QFSPPYNPLGADFDYTEAFK.S
93	-	100	864.5270	863.5197	863.4752	51.5	0	K.SLDFAALK.K
143	-	151	877.4938	876.4866	876.3872	113	0	R.GGSGMGQQR.F
143	-	151	893.4635	892.4562	892.3821	83.0	0	R.GGSGMGQQR.F + Oxidation (M)
152	-	166	1759.7790	1758.7717	1758.8213	-28.2	0	R.FAPLDSWPDNQNLDK.A
211	-	231	2401.9719	2400.9646	2401.0750	-46.0	0	R.ADTWEADESIYWGAESTFVPK.G
237	-	246	1217.6057	1216.5984	1216.5360	51.3	0	R.YNGSTDIYER.A
277	-	283	745.5402	744.5329	744.4130	161	0	K.ASALDIR.T
284	-	288	551.4124	550.4051	550.2863	216	0	R.TAFGR.M
362	-	368	931.5517	930.5444	930.4712	78.7	0	K.WSNHFLK.S
394	-	402	1107.6134	1106.6061	1106.5648	37.3	0	K.LEYPDPFVK.G
407	-	425	2148.0167	2147.0094	2147.1296	-56.0	0	R.RPTMLTSDLALINDPSYLK.I
436	-	443	891.5399	890.5327	890.4610	80.5	0	K.ELNAAFAR.A
436	-	447	1439.7660	1438.7587	1438.7357	16.0	1	K.ELNAAFARAWYK.L
448	-	451	538.4558	537.4485	537.3387	204	0	K.LLHR.D
469	-	478	1300.7304	1299.7231	1299.6612	47.7	0	K.FIWQDPLPER.K
469	-	479	1428.7789	1427.7716	1427.7561	10.9	1	K.FIWQDPLPERK.G
479	-	492	1445.7564	1444.7491	1444.7773	-19.5	1	R.KGDIIGEADISSLK.S
524	-	545	2292.8668	2291.8595	2291.1770	298	1	R.GGANGARIALEPQVNWVSNNPK.Q
531	-	545	1708.8348	1707.8275	1707.8944	-39.2	0	R.IALEPQVNWVSNNPK.Q
569	-	586	1795.9732	1794.9659	1795.0819	-64.6	1	K.KVSLADLIVLGGVAAIEK.A
570	-	586	1667.8795	1666.8722	1666.9869	-68.8	0	K.VSLADLIVLGGVAAIEK.A
587	-	604	1860.9300	1859.9227	1859.9530	-16.3	1	K.AAQAAGFKDVEVPFTPGR.V
595	-	604	1116.6498	1115.6425	1115.5611	73.0	0	K.DVEVPFTPGR.V
605	-	628	2697.2363	2696.2290	2696.3082	-29.4	0	R.VDATQNQTDLVQFGYLEPLADGFR.N
629	-	636	875.5154	874.5082	874.4046	118	0	R.NYGHGTAR.A
637	-	646	1201.6701	1200.6628	1200.6462	13.8	1	R.ARTEEILVDR.A
639	-	646	974.5779	973.5706	973.5080	64.3	0	R.TEEILVDR.A
647	-	665	1952.0510	1951.0437	1951.1176	-37.9	0	R.AALLTLTPPEMTVLVGGLR.A
647	-	665	1968.0565	1967.0492	1967.1125	-32.2	0	R.AALLTLTPPEMTVLVGGLR.A + Oxidation (M)
704	-	716	1492.7171	1491.7098	1491.6954	9.68	1	K.KDSQGELWTGTDR.A
705	-	716	1364.6443	1363.6370	1363.6004	26.8	0	K.DSQGELWTGTDR.A
723	-	727	634.4443	633.4370	633.3235	179	0	K.WTATR.A
728	-	739	1314.7274	1313.7201	1313.6728	36.0	0	R.ADLVFGSHAELR.A
740	-	751	1210.5937	1209.5864	1209.5877	-1.06	0	R.AISEVYGSADAK.E
757	-	764	967.5011	966.4939	966.4811	13.2	0	K.DFISAWTK.V

Spot No.: **93**

Mascot score: **81**

Species: ***Fusarium oxysporum Fo5176***

Protein name: **l-amino acid oxidase**

NCBI accession No.: **gi| 342871725**

Sequence coverage %: **27**

Matched peptides No.: **18**

Total peptides No.: **69**

Calculated Mr: **66173**

Calculated *p*I: **5.58**

Annotated PMF **spectra:**

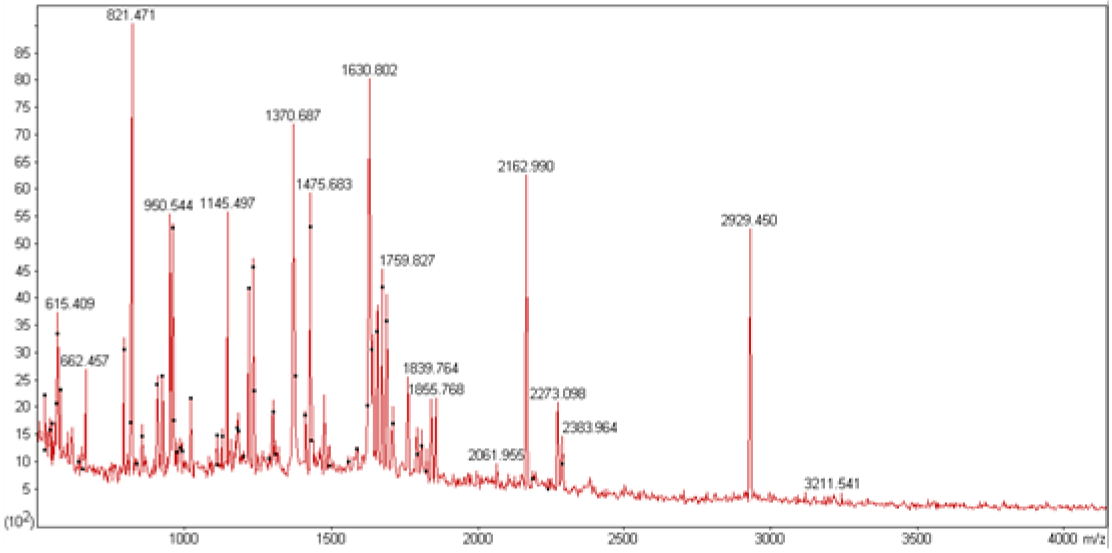

Probability Based Mowse Score:

Protein score is -10*Log(P), where P is the probability that the observed match is a random event.
Protein scores greater than 75 are significant (p<0.05).

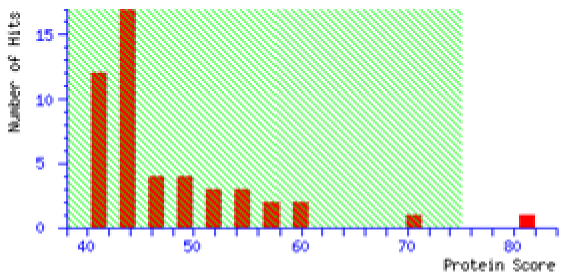

Matched peptide sequences: shown in **Bold Red**

1	MVQFTDKRWG	ASVLLLLFLS	TAHGSKLPPK	AQQPSSCTER	PRMQNFNSIG
51	AWFDDVAKLN	CNSVSKAPNA	SVAIIGGGVS	GLTTALMLDS	IGLHNWEIIE
101	ASDRVGGRFR	TK**FVGGTQEF**	**AEMGPMRLPY**	**TVTYK**SDDST	HEYTEHRLTF
151	QLAETLNEMN	GNDSNIILMS	LLPGELAAIQ	MAEFQQDANS	SLAKPPAMVS
201	TEFNETKHRM	NEILKNETML	K**AIQADVWR**S	HKFVMSKGYD	DWSEQCMMRE
251	AFHASENITD	AIWTATDYDV	FWDEMVHNSN	LALDGTKDSL	GETEWK**CVDG**
301	**GFNRLTDAFI**	**PHVSDR**LVLN	RKIRKLESVK	**GEDGQAQTR**L	SWYPSVKNRT
351	FESK**DYDYTI**	**MAVPFTMTRF**	**MALPSFSSVL**	**GRAISEAGLR**	FKSACK**VSLL**
401	**FSER**FWEKGE	RPIFGGYSIP	ESRPIGALYY	PVYGLNESRP	GLITHYR**GGD**
451	**WSDRYVSFSD**	**EEHVQTVLDA**	**IVSLHGEQAR**	**ELYTGDYER**L	CWLKDEHTAT
501	SWCRPDVEQH	NLYIPAYHQT	EHNTIFIGEH	TAPTQAWISS	AIYSAAR**GTI**
551	**QLLLELGMVE**	**EAK**EINRRWM	GRWIRDETKP		

Matched peptides information:

Start	-	End	Observed	Mr(expt)	Mr(calc)	ppm	M	Peptide
113	-	127	1656.7168	1655.7095	1655.7436	-20.6	0	K.FVGGTQEFAEMGPMR.L
113	-	127	1672.6753	1671.6680	1671.7385	-42.2	0	K.FVGGTQEFAEMGPMR.L + Oxidation (M)
113	-	127	1688.6606	1687.6533	1687.7334	-47.5	0	K.FVGGTQEFAEMGPMR.L + 2 Oxidation (M)
128	-	135	984.4982	983.4909	983.5328	-42.5	0	R.LPYTVTYK.S
222	-	229	958.5107	957.5034	957.5032	0.25	0	K.AIQADVWR.S
297	-	304	924.3967	923.3895	923.3920	-2.72	0	K.CVDGGFNR.L
305	-	316	1370.6872	1369.6799	1369.6990	-13.9	0	R.LTDAFIPHVSDR.L
331	-	339	961.5282	960.5209	960.4261	98.7	0	K.GEDGQAQTR.L
355	-	369	1839.7640	1838.7567	1838.8219	-35.5	0	K.DYDYTIMAVPFTMTR.F + Oxidation (M)
355	-	369	1855.7683	1854.7610	1854.8168	-30.1	0	K.DYDYTIMAVPFTMTR.F + 2 Oxidation (M)
370	-	382	1411.6746	1410.6673	1410.7330	-46.5	0	R.FMALPSFSSVLGR.A
370	-	382	1427.6801	1426.6728	1426.7279	-38.6	0	R.FMALPSFSSVLGR.A + Oxidation (M)
383	-	390	816.4800	815.4728	815.4501	27.8	0	R.AISEAGLR.F
397	-	404	950.5444	949.5372	949.5233	14.6	0	K.VSLLFSER.F
448	-	454	792.3647	791.3574	791.3198	47.5	0	R.GGDWSDR.Y
455	-	480	2929.4501	2928.4428	2928.4254	5.96	0	R.YVSFSDEEHVQTVLDAIVSLHGEQAR.E
481	-	489	1145.4971	1144.4898	1144.5036	-12.1	0	R.ELYTGDYER.L
548	-	563	1759.8271	1758.8198	1758.9437	-70.4	0	R.GTIQLLLELGMVEEAK.E + Oxidation (M)

Spot No.: **94**

Mascot score: **88**

Species: ***Metarhizium acridum CQMa 102***

Protein name: **heat shock protein 90**

NCBI accession No.: **gi| 322700250**

Sequence coverage %: **26**

Matched peptides No.: **13**

Total peptides No.: **52**

Calculated Mr: **80088**

Calculated *p*I: **4.93**

Annotated PMF **spectra:**

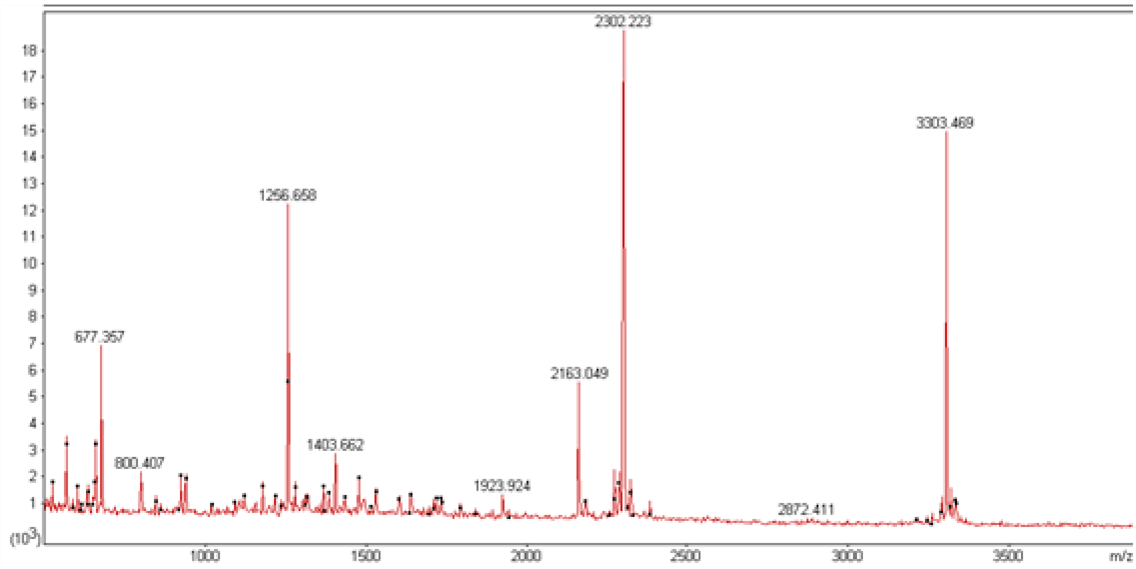

Probability Based Mowse Score:

Protein score is -10*Log(P), where P is the probability that the observed match is a random event.
Protein scores greater than 75 are significant (p<0.05).

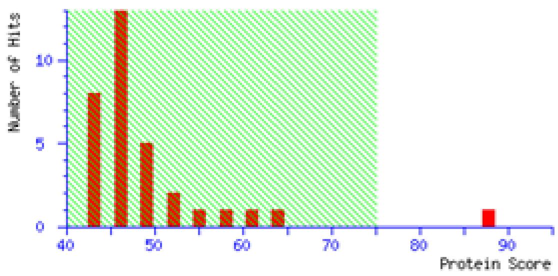

Matched peptide sequences: shown in Bold Red

```
  1  MSETFEFQAE  ISQLLSLIIN  TVYSNKEIFL  RELVSNASDA  LDKIRYKALS
 51  DPSQLDSGKD  LRIDIIPNKE  AKTLTIRDTG  IGMTKADLVN  NLGTIARSGT
101  KQFMEALTAG  ADVSMIGQFG  VGFYSAYLVA  DKVTVVSKNN  DDEQYIWESS
151  AGGTFSITAD  TEGKQLGRGT  SIILHLKDEQ  AEYLNESKIK  EVIKKHSEFI
201  SYPIYLHVQK  EIEKEVPDED  AEAEEAKEEE  GDDKKPRIEE  VDDEDEEKEK
251  KKKTKKVKET  TIEEEELNKQ  KPIWTRNPQD  ITQEEYASFY  KSLSNDWEDH
301  LAVKHFSVEG  QLEFRAILFV  PKRAPFDLFE  TKKTKNNIKL  YVRRVFITDD
351  ATELIPEWLS  FVKGVVDSED  LPLNLSRETL  QQNKIMKVIK  KNIVKKSLEL
401  FQEIAEDKEQ  FDKFYSAFSK  NLKLGIHEDS  QNRQQLAKLL  RFNSTKSGDE
451  MTSLSDYVTR  MPEHQKNMYY  ITGESIKAVS  KSPFLDTLKE  KGFEVLFLVD
501  PIDEYAMTQL  KEFEEKKLVD  ITKDFELEET  DEEKKAREEE  EKEYESLAKS
551  LKNVLGEKVE  KVVVSHKLGL  SPCAIRTGQF  GWSANMERIM  KAQALRDTSM
601  SSYMSSKKTF  EISPKSPIIK  ELKKKVEADG  ENDRTVKSIV  QLLFETSLLV
651  SGFTIEEPAG  FAERIHKLVQ  LGLNIEEDDS  APAEAPAEAA  PAAETGDSAM
701  EEVD
```

Matched peptides information:

Start	-	End	Observed	Mr(expt)	Mr(calc)	ppm	M	Peptide
27	-	31	677.3574	676.3501	676.3908	-60.2	0	K.EIFLR.E
32	-	45	1530.8175	1529.8102	1529.8049	3.48	1	R.ELVSNASDALDKIR.Y
48	-	59	1217.5648	1216.5575	1216.5935	-29.6	0	K.ALSDPSQLDSGK.D
48	-	62	1601.8038	1600.7965	1600.8056	-5.70	1	K.ALSDPSQLDSGKDLR.I
73	-	77	603.3235	602.3162	602.3752	-97.9	0	K.TLTIR.D
86	-	97	1256.6581	1255.6508	1255.6884	-29.9	0	K.ADLVNNLGTIAR.S
102	-	132	3303.4686	3302.4613	3301.5676	271	0	K.QFMEALTAGADVSMIGQFGVGFYSAYLVADK.V + Oxidation (M)
139	-	168	3289.3475	3288.3402	3288.4807	-42.7	1	K.NNDDEQYIWESSAGGTFSITADTEGKQLGR.G
235	-	248	1730.8546	1729.8473	1729.8006	27.0	1	K.KPRIEEVDDEDEEK.E
553	-	558	659.3396	658.3323	658.3650	-49.6	0	K.NVLGEK.V
577	-	588	1383.6584	1382.6511	1382.6037	34.3	0	R.TGQFGWSANMER.I
597	-	607	1255.5411	1254.5338	1254.4744	47.4	0	R.DTSMSSYMSSK.K + 2 Oxidation (M)
638	-	667	3332.5234	3331.5161	3330.7864	219	1	K.SIVQLLFETSLLVSGFTIEEPAGFAERIHK.L

Spot No.: **95**

Mascot score: **110**

Species: ***Fusarium oxysporum f. sp. cubense race 4***

Protein name: **hypothetical protein FOC4_g10007540**

NCBI accession No.: **gi| 475667056**

Sequence coverage %: **38**

Matched peptides No.: **17**

Total peptides No.: **72**

Calculated Mr: **50452**

Calculated *p*I: **6.62**

Annotated PMF **spectra:**

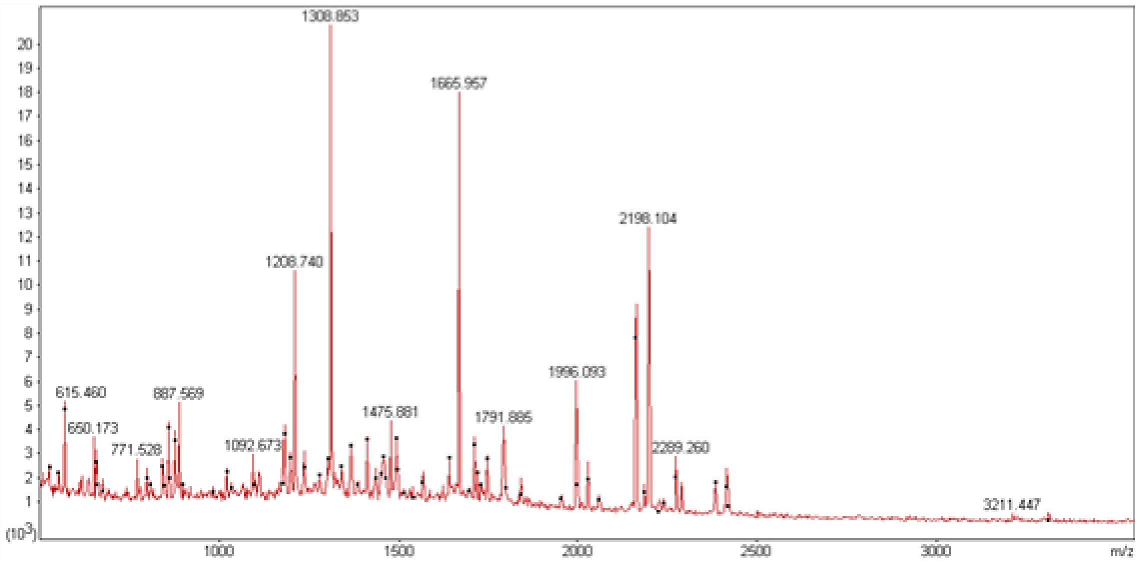

Probability Based Mowse Score:

Protein score is -10*Log(P), where P is the probability that the observed match is a random event. Protein scores greater than 75 are significant (p<0.05).

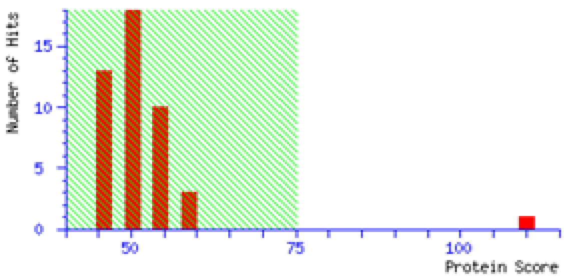

Matched peptide sequences: shown in **Bold Red**

```
  1  MVSSKLLFLA VIAAVQAIDV DVAVVGGGGS GGYAAVQLRE NYGKKIVVIE
 51  KQKQLGGHAQ SWYDPVTGKA YNYGVDAFTN ITVSIDFFKQ LKVPIGPVQS
101  EQVRNLYVDF KDGKTVDYTP PTTKEAADAM GNYRDQWLKY TDILLPTSEN
151  FPRGDKVPSD LLLSWYEFAR KYKAEAASPS IWDTVVVDLN TALMIDVWKA
201  WNPSVGSFQP ASGDNTEIWQ KAAKVLGKDV LYESEVVSAK RSKSGVKLQV
251  RDKDGQVINI NAKRLLITIG PETINPKYFD LNSEELEVFH SAAGNRYYTG
301  IVSHPSLPAA EITNIVPAAI NENYLAYPTV PFQAYFHYKG NSSTGPIHRA
351  LAIVPRDTSI EDAKDLIRKS LQNLIDAGTI PAGNSTDLDF RTFSDHGLLY
401  RRWSADQLRG GIFAKANALQ GQRSTWYTGA FWMNNDCVML WNTTNAILKE
451  MLKDI
```

Matched peptides information:

Start	-	End	Observed	Mr(expt)	Mr(calc)	ppm	M	Peptide
52	-	69	2000.1163	1999.1090	1998.9912	59.0	1	K.QKQLGGHAQSWYDPVTGK.A
54	-	69	1743.9508	1742.9435	1742.8376	60.8	0	K.QLGGHAQSWYDPVTGK.A
93	-	104	1308.8529	1307.8456	1307.7198	96.2	0	K.VPIGPVQSEQVR.N
105	-	111	898.4947	897.4874	897.4596	31.0	0	R.NLYVDFK.D
125	-	134	1097.6195	1096.6122	1096.4607	138	0	K.EAADAMGNYR.D
140	-	153	1665.9566	1664.9493	1664.8410	65.1	0	K.YTDILLPTSENFPR.G
154	-	170	1996.0927	1995.0854	1995.0102	37.7	1	R.GDKVPSDLLLSWYEFAR.K
200	-	221	2419.2079	2418.2006	2418.1240	31.7	0	K.AWNPSVGSFQPASGDNTEIWQK.A
229	-	240	1338.7752	1337.7679	1337.6714	72.1	0	K.DVLYESEVVSAK.R
264	-	277	1565.0035	1563.9962	1563.9348	39.3	1	K.RLLITIGPETINPK.Y
265	-	277	1408.9490	1407.9417	1407.8337	76.7	0	R.LLITIGPETINPK.Y
278	-	296	2198.1039	2197.0966	2197.0076	40.5	0	K.YFDLNSEELEVFHSAAGNR.Y
392	-	401	1208.7396	1207.7323	1207.5986	111	0	R.TFSDHGLLYR.R
402	-	409	1031.6618	1030.6545	1030.5308	120	1	R.RWSADQLR.G
403	-	409	875.5438	874.5365	874.4297	122	0	R.WSADQLR.G
403	-	415	1448.7861	1447.7788	1447.7572	14.9	1	R.WSADQLRGGIFAK.A
416	-	423	857.5740	856.5667	856.4515	135	0	K.ANALQGQR.S

Spot No.: **96**

NCBI accession No.: **gi|475671883**

Plant species: ***Fusarium oxysporum f. sp. cubense race 4***

Protein name: **Ribonuclease Trv**

Mascot score: **185** Sequence coverage %: **13**

The number of matched peptides with p≤0.05: **3**

Calculated Mr: **29672** Calculated *Pi*: **5.61**

Annotated MS **spectra:**

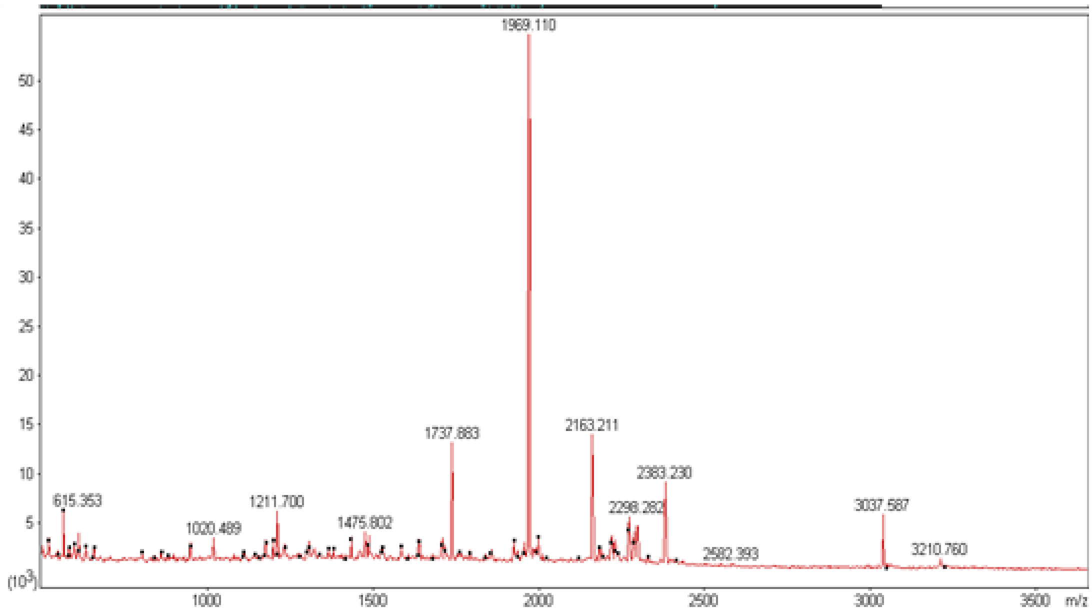

Probability Based Mowse Score:

Ions score is -10*Log(P), where P is the probability that the observed match is a random event.
Individual ions scores > 47 indicate identity or extensive homology (p<0.05).
Protein scores are derived from ions scores as a non-probabilistic basis for ranking protein hits.

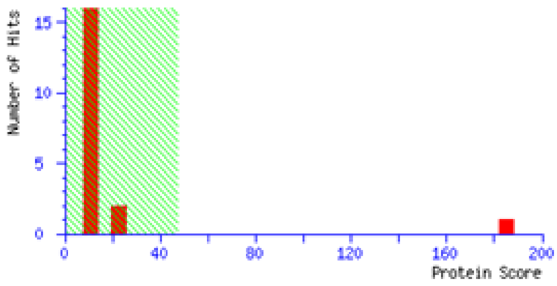

Matched peptide sequences: shown in Bold Red

1	MTFIKALIPL	ALYLAGVQAK	SCSAGGKPTA	EFCSKDLPLS	CHNTTAVEDT
51	CCFIPAGQLL	QTQFWDSDPV	AGPHDSWTIH	GLWPDYCDGT	YPQFCDKSRE
101	YTNIKDLVTK	FLGKKTVSYM	DKYWVSQDGN	DESLWEHEFN	KHGTCISTLE
151	PSCYTNYETG	AEAADYVKKT	ISLFKTLPTY	KWLAEAGIKP	SKTKTYTADE
201	IVDALAEHHG	ARVTIGCSNG	SLSEVWYHFN	VKGSLQDGQF	VSSEPDGSKS
251	SCPDSGIKYA	PKK			

Matched peptides information:

Query	Start - End	Observed	Mr(expt)	Mr(calc)	ppm	M	Score	Expect	Rank	U	Peptide
[illegible]	195 - 212	1969.1100	1968.1027	1967.9337	85.9	0	127	5e-10	1	U	K.TYTADEIVDALAEHHGAR.V
[illegible]	233 - 249	1737.8831	1736.8758	1736.7853	52.1	0	58	0.005	1	U	K.GSLQDGQFVSSEPDGSK.S
[illegible]	233 - 249	1737.8831	1736.8758	1736.7853	52.1	0	58	0.005	1	U	K.GSLQDGQFVSSEPDGSK.S

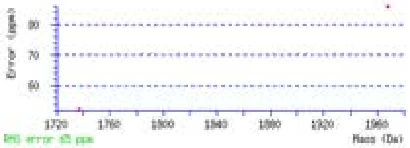

Annotated ion spectra of the matched peptides with p≤0.05:

CID No.: **96-1737.8**

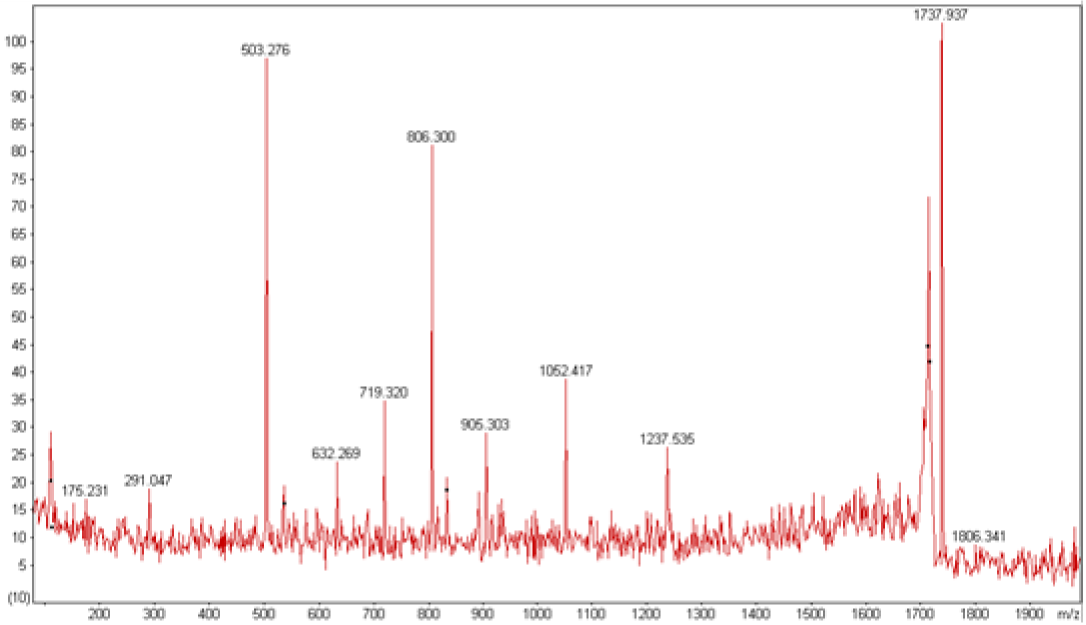

MS/MS Fragmentation of **K.GSLQDGQFVSSEPDGSK.S**

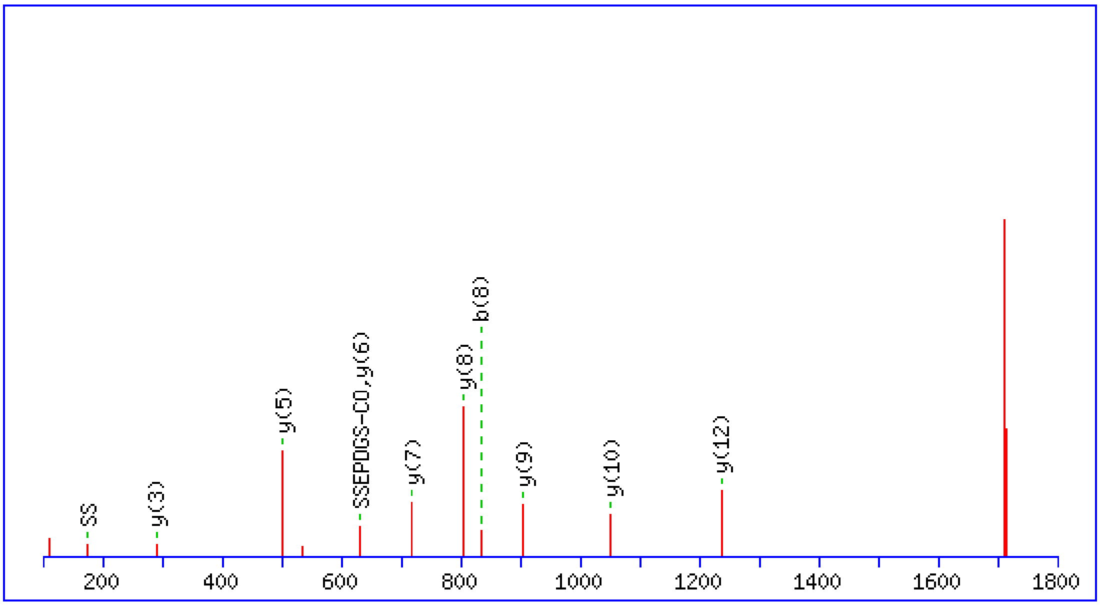

Monoisotopic mass of neutral peptide Mr(calc): 1736.7853

#	Immon.	a	a*	a^0	b	b*	b^0	d	Seq.	v	w	y	y*	y^0	#
1	30.0338	30.0338			58.0287			44.0495	G						17
2	60.0444	117.0659		99.0553	145.0608		127.0502	101.0709	S	1648.7449	1647.7497	1680.7711	1663.7446	1662.7606	16
3	86.0964	230.1499		212.1394	258.1448		240.1343	188.1030	L	1535.6609	1534.6656	1593.7391	1576.7126	1575.7285	15
4	101.0709	358.2085	341.1819	340.1979	386.2034	369.1769	368.1928	301.1870	Q	1407.6023	1406.6070	1480.6550	1463.6285	1462.6445	14
5	88.0393	473.2354	456.2089	455.2249	501.2304	484.2038	483.2198	429.2456	D	1292.5753	1291.5801	1352.5965	1335.5699	1334.5859	13
6	30.0338	530.2569	513.2304	512.2463	558.2518	541.2253	540.2413		G			*1237.5695*	1220.5430	1219.5590	12
7	101.0709	658.3155	641.2889	640.3049	686.3104	669.2838	668.2998	601.2940	Q	1107.4953	1106.5000	1180.5481	1163.5215	1162.5375	11
8	120.0808	805.3839	788.3573	787.3733	833.3788	816.3523	815.3682		F	960.4269		*1052.4895*	1035.4629	1034.4789	10
9	72.0808	904.4523	887.4258	886.4417	932.4472	915.4207	914.4367	890.4367	V	861.3585	874.3789	*905.4211*	888.3945	887.4105	9
10	60.0444	991.4843	974.4578	973.4738	1019.4793	1002.4527	1001.4687	975.4894	S	774.3264	773.3312	*806.3527*	789.3261	788.3421	8
11	60.0444	1078.5164	1061.4898	1060.5058	1106.5113	1089.4847	1088.5007	1062.5214	S	687.2944	686.2992	*719.3206*	702.2941	701.3101	7
12	102.0550	1207.5590	1190.5324	1189.5484	1235.5539	1218.5273	1217.5433	1149.5535	E	558.2518	557.2566	*632.2886*	615.2620	614.2780	6
13	70.0651	1304.6117	1287.5852	1286.6012	1332.6066	1315.5801	1314.5961	1278.5961	P	461.1991	460.2038	*503.2460*	486.2195	485.2354	5
14	88.0393	1419.6387	1402.6121	1401.6281	1447.6336	1430.6070	1429.6230	1375.6488	D	346.1721	345.1769	406.1932	389.1667	388.1827	4
15	30.0338	1476.6601	1459.6336	1458.6496	1504.6550	1487.6285	1486.6445		G			*291.1663*	274.1397	273.1557	3
16	60.0444	1563.6922	1546.6656	1545.6816	1591.6871	1574.6605	1573.6765	1547.6972	S	202.1186	201.1234	234.1448	217.1183	216.1343	2
17	101.1073								K	74.0237	73.0284	147.1128	130.0863		1

Seq	ya	yb	Seq	ya	yb	Seq	ya	yb
SL	173.1285	201.1234	SLQ	301.1870	329.1819	SLQD	416.2140	444.2089
SLQDG	473.2354	501.2304	SLQDGQ	601.2940	629.2889	LQ	214.1550	242.1499
LQD	329.1819	357.1769	LQDG	386.2034	414.1983	LQDGQ	514.2620	542.2569
LQDGQF	661.3304	689.3253	QD	216.0979	244.0928	QDG	273.1193	301.1143
QDGQ	401.1779	429.1728	QDGQF	548.2463	576.2413	QDGQFV	647.3148	675.3097
DG	145.0608	173.0557	DGQ	273.1193	301.1143	DGQF	420.1878	448.1827
DGQFV	519.2562	547.2511	DGQFVS	606.2882	634.2831	DGQFVSS	693.3202	721.3151
GQ	158.0924	186.0873	GQF	305.1608	333.1557	GQFV	404.2292	432.2241
GQFVS	491.2613	519.2562	GQFVSS	578.2933	606.2882	QF	248.1394	276.1343
QFV	347.2078	375.2027	QFVS	434.2398	462.2347	QFVSS	521.2718	549.2667
QFVSSE	650.3144	678.3093	FV	219.1492	247.1441	FVS	306.1812	334.1761
FVSS	393.2132	421.2082	FVSSE	522.2558	550.2508	FVSSEP	619.3086	647.3035
VS	159.1128	187.1077	VSS	246.1448	274.1397	VSSE	375.1874	403.1823
VSSEP	472.2402	500.2351	VSSEPD	587.2671	615.2620	VSSEPDG	644.2886	672.2835
SS	147.0764	175.0713	SSE	276.1190	304.1139	SSEP	373.1718	401.1667
SSEPD	488.1987	516.1936	SSEPDG	545.2202	573.2151	SSEPDGS	632.2522	660.2471
SE	189.0870	217.0819	SEP	286.1397	314.1347	SEPD	401.1667	429.1616
SEPDG	458.1882	486.1831	SEPDGS	545.2202	573.2151	EP	199.1077	227.1026
EPD	314.1347	342.1296	EPDG	371.1561	399.1510	EPDGS	458.1882	486.1831
PD	185.0921	213.0870	PDG	242.1135	270.1084	PDGS	329.1456	357.1405
DG	145.0608	173.0557	DGS	232.0928	260.0877	GS	117.0659	145.0608

Annotated ion spectra of the matched peptides with p≤0.05:

CID No.: **96-1969.1**

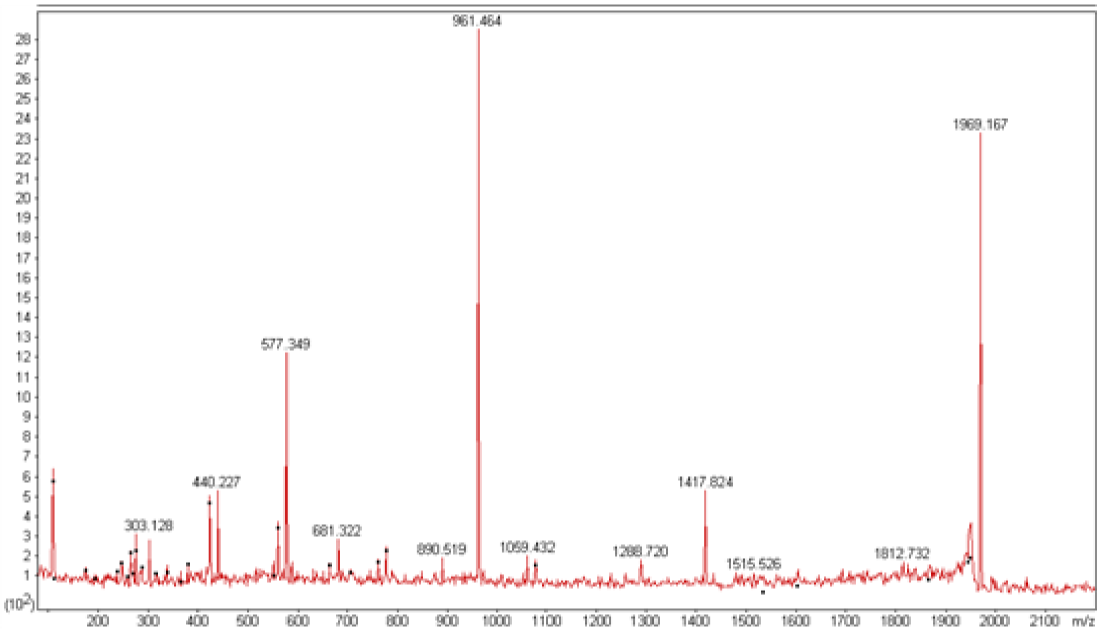

MS/MS Fragmentation of **K.TYTADEIVDALAEHHGAR.V**

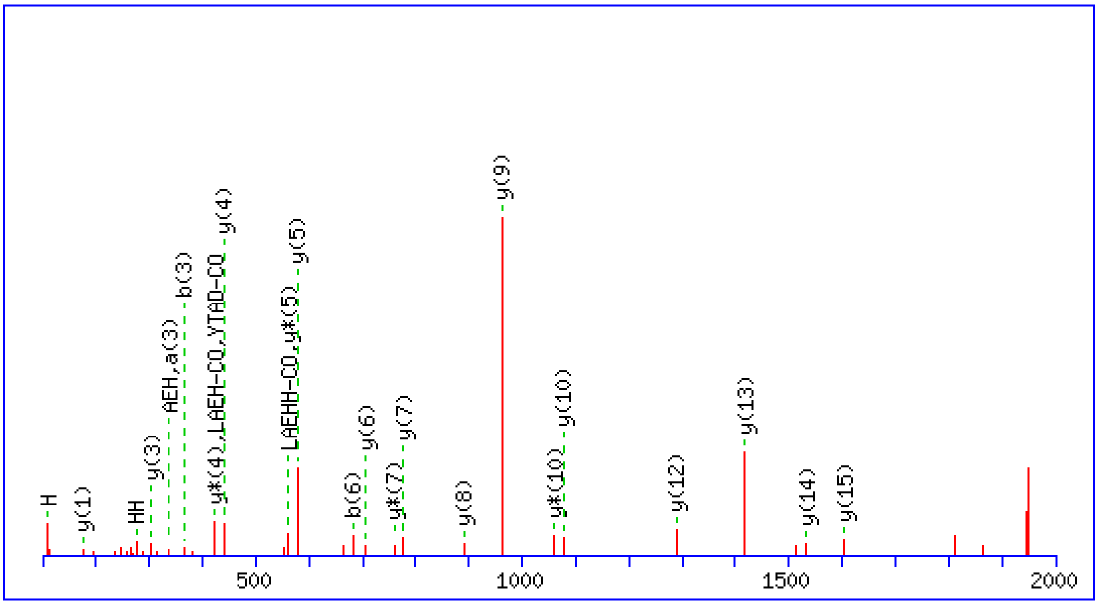

Monoisotopic mass of neutral peptide Mr(calc): 1967.9337

#	Immon.	a	a^0	b	b^0	d	d'	Seq.	v	w	w'	y	y*	y^0	#
1	74.0600	74.0600	56.0495	102.0550	84.0444	44.0495		T							18
2	136.0757	237.1234	219.1128	265.1183	247.1077			Y	1759.8358			1867.8933	1850.8668	1849.8828	17
3	74.0600	338.1710	320.1605	**366.1660**	348.1554	322.1761	324.1554	T	1658.7881	1671.8085	1673.7878	1704.8300	1687.8034	1686.8194	16
4	44.0495	409.2082	391.1976	437.2031	419.1925			A	1587.7510			***1603.7823***	1586.7558	1585.7717	15
5	88.0393	524.2351	506.2245	552.2300	534.2195	480.2453		D	1472.7241	1471.7288		***1532.7452***	1515.7186	1514.7346	14
6	102.0550	653.2777	635.2671	**681.2726**	663.2620	595.2722		E	1343.6815	1342.6862		***1417.7183***	1400.6917	1399.7077	13
7	86.0964	766.3618	748.3512	794.3567	776.3461	738.3305	752.3461	I	1230.5974	1243.6178	1257.6335	***1288.6757***	1271.6491	1270.6651	12
8	72.0808	865.4302	847.4196	893.4251	875.4145	851.4145		V	1131.5290	1144.5494		1175.5916	1158.5650	1157.5810	11
9	88.0393	980.4571	962.4466	1008.4520	990.4415	936.4673		D	1016.5021	1015.5068		***1076.5232***	1059.4966	1058.5126	10
10	44.0495	1051.4942	1033.4837	1079.4891	1061.4786			A	945.4649			***961.4962***	944.4697	943.4857	9
11	86.0964	1164.5783	1146.5677	1192.5732	1174.5626	1122.5313		L	832.3809	831.3856		***890.4591***	873.4326	872.4486	8
12	44.0495	1235.6154	1217.6048	1263.6103	1245.5998			A	761.3438			***777.3751***	760.3485	759.3645	7
13	102.0550	1364.6580	1346.6474	1392.6529	1374.6424	1306.6525		E	632.3012	631.3059		***706.3379***	689.3114	688.3274	6
14	110.0713	1501.7169	1483.7064	1529.7118	1511.7013			H	495.2423			***577.2954***	560.2688		5
15	110.0713	1638.7758	1620.7653	1666.7707	1648.7602			H	358.1833			***440.2364***	423.2099		4
16	30.0338	1695.7973	1677.7867	1723.7922	1705.7816			G				***303.1775***	286.1510		3
17	44.0495	1766.8344	1748.8238	1794.8293	1776.8188			A	230.1248			246.1561	229.1295		2
18	129.1135							R	74.0237	73.0284		***175.1190***	158.0924		1

Seq	ya	yb	Seq	ya	yb	Seq	ya	yb
**YT**	237.1234	265.1183	**YTA**	308.1605	336.1554	**YTAD**	423.1874	451.1823
**YTADE**	552.2300	580.2249	**YTADEI**	665.3141	693.3090	**TA**	145.0972	173.0921
**TAD**	260.1241	288.1190	**TADE**	389.1667	417.1616	**TADEI**	502.2508	530.2457
**TADEIV**	601.3192	629.3141	**AD**	159.0764	187.0713	**ADE**	288.1190	316.1139
**ADEI**	401.2031	429.1980	**ADEIV**	500.2715	528.2664	**ADEIVD**	615.2984	643.2933
**ADEIVDA**	686.3355	714.3305	**DE**	217.0819	245.0768	**DEI**	330.1660	358.1609
**DEIV**	429.2344	457.2293	**DEIVD**	544.2613	572.2562	**DEIVDA**	615.2984	643.2933
**EI**	215.1390	243.1339	**EIV**	314.2074	342.2023	**EIVD**	429.2344	457.2293
**EIVDA**	500.2715	528.2664	**EIVDAL**	613.3556	641.3505	**EIVDALA**	684.3927	712.3876
**IV**	185.1648	213.1598	**IVD**	300.1918	328.1867	**IVDA**	371.2289	399.2238
**IVDAL**	484.3130	512.3079	**IVDALA**	555.3501	583.3450	**IVDALAE**	684.3927	712.3876
**VD**	187.1077	215.1026	**VDA**	258.1448	286.1397	**VDAL**	371.2289	399.2238
**VDALA**	442.2660	470.2609	**VDALAE**	571.3086	599.3035	**DA**	159.0764	187.0713
**DAL**	272.1605	300.1554	**DALA**	343.1976	371.1925	**DALAE**	472.2402	500.2351
**DALAEH**	609.2991	637.2940	**AL**	157.1335	185.1285	**ALA**	228.1707	256.1656
**ALAE**	357.2132	385.2082	**ALAEH**	494.2722	522.2671	**ALAEHH**	631.3311	659.3260
**ALAEHHG**	688.3525	716.3474	**LA**	157.1335	185.1285	**LAE**	286.1761	314.1710
**LAEH**	423.2350	451.2300	**LAEHH**	560.2940	588.2889	**LAEHHG**	617.3154	645.3103
**LAEHHGA**	688.3525	716.3474	**AE**	173.0921	201.0870	**AEH**	310.1510	338.1459
**AEHH**	447.2099	475.2048	**AEHHG**	504.2314	532.2263	**AEHHGA**	575.2685	603.2634
**EH**	239.1139	267.1088	**EHH**	376.1728	404.1677	**EHHG**	433.1942	461.1892
**EHHGA**	504.2314	532.2263	**HH**	247.1302	275.1251	**HHG**	304.1516	332.1466
**HHGA**	375.1888	403.1837	**HG**	167.0927	195.0877	**HGA**	238.1299	266.1248
**GA**	101.0709	129.0659						

Spot No.: **97**

Mascot score: **92**

Species: ***Fusarium oxysporum f. sp. cubense race 1***

Protein name: **14-3-3 protein like protein**

NCBI accession No.: **gi| 477507337**

Sequence coverage %: **61**

Matched peptides No.: **13**

Total peptides No.: **60**

Calculated Mr: **30058**

Calculated *p*I: **4.87**

Annotated PMF spectra:

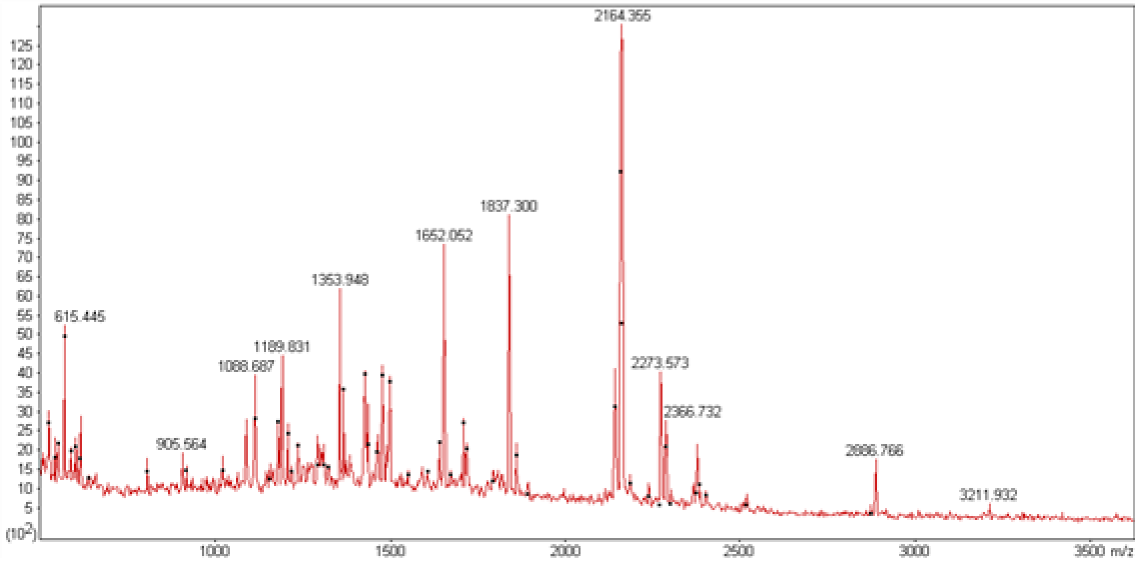

Probability Based Mowse Score:

Protein score is -10*Log(P), where P is the probability that the observed match is a random event.
Protein scores greater than 75 are significant (p<0.05).

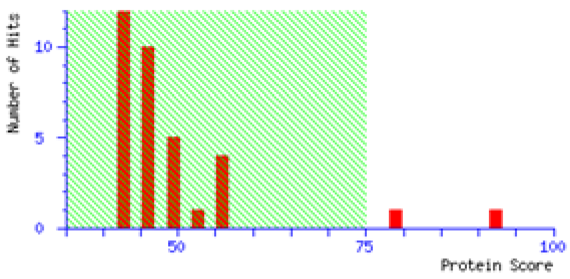

Matched peptide sequences: shown in Bold Red

```
  1 MATETFLARL CEQAERYDEM VTYMKEVAKL GGELTVDERN LLSVAYKNVV
 51 GTRRASWRII SSIEQKEESK GSDKHVSTIK DYRNKIETEL EKVCQDVLDV
101 LDDFLIPNAA TGESKVFYHK MKGDYHRYLA EFASGEKRKG AATAAHDAYK
151 SATDVAQTEL TPTHPIRLGL ALNFSVFYYE ILNSPDRACH LAKQAFDDAI
201 AELDSLSEES YRDSTLIMQL LRDNLTLWTS SDSAEGEAAG AADAPKKEEE
251 AAKPAEPAAA PAEEPAPAAA S
```

Matched peptides information:

Start	-	End	Observed	Mr(expt)	Mr(calc)	ppm	M	Peptide
10	-	16	905.5637	904.5564	904.4072	165	0	R.LCEQAER.Y
17	-	25	1179.7910	1178.7837	1178.4988	242	0	R.YDEMVTYMK.E
17	-	29	1639.1938	1638.1865	1637.7317	278	1	R.YDEMVTYMKEVAK.L + 2 Oxidation (M)
30	-	39	1088.6870	1087.6797	1087.5509	118	0	K.LGGELTVDER.N
59	-	66	917.5289	916.5217	916.5229	-1.37	0	R.IISSIEQK.E
93	-	115	2518.8010	2517.7937	2518.2261	-172	0	K.VCQDVLDVLDDFLIPNAATGESK.V
128	-	137	1114.6283	1113.6210	1113.5342	78.0	0	R.YLAEFASGEK.R
151	-	167	1837.2996	1836.2923	1835.9377	193	0	K.SATDVAQTELTPTHPIR.L
194	-	212	2159.3618	2158.3545	2157.9702	178	0	K.QAFDDAIAELDSLSEESYR.D
213	-	222	1189.8305	1188.8232	1188.6536	143	0	R.DSTLIMQLLR.D
213	-	222	1205.8528	1204.8455	1204.6485	164	0	R.DSTLIMQLLR.D + Oxidation (M)
223	-	246	2376.5861	2375.5788	2376.0717	-207	0	R.DNLTLWTSSDSAEGEAAGAADAPK.K
247	-	271	2403.5536	2402.5463	2402.1600	161	1	K.KEEEAAKPAEPAAAPAEEPAPAAAS.-

Spot No.: **98**

Mascot score: **83**

Species: ***Fusarium oxysporum f. sp. cubense race 1***

Protein name: **Beta-hexosaminidase subunit A1**

NCBI accession No.: **gi| 477513214** Sequence coverage %: **31**

Matched peptides No.: **12** Total peptides No.: **64**

Calculated Mr: **34198** Calculated *p*I: **6.25**

Annotated PMF **spectra:**

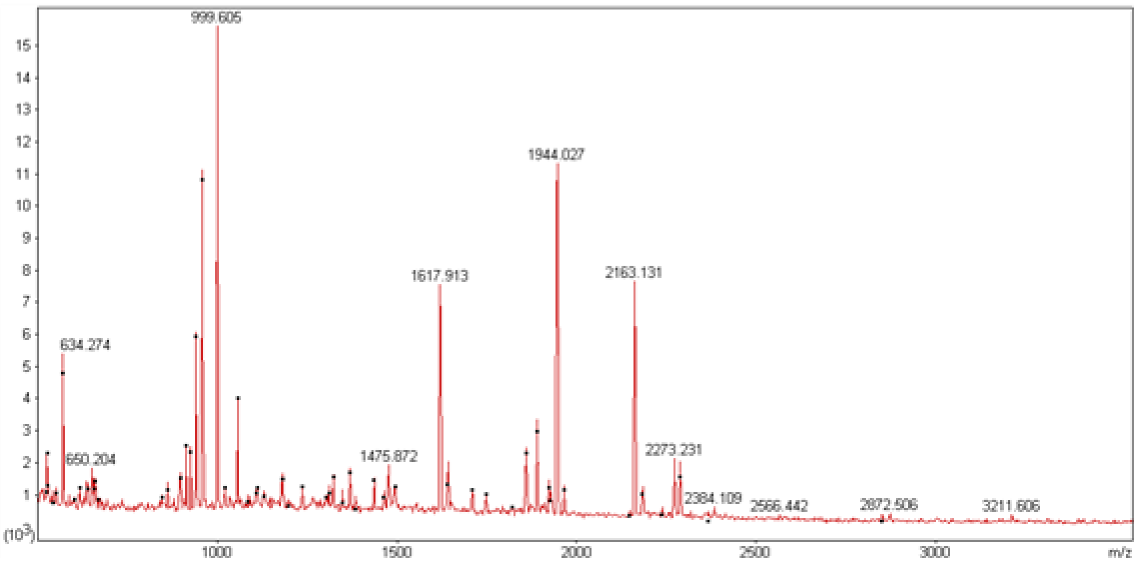

Probability Based Mowse Score:

Protein score is -10*Log(P), where P is the probability that the observed match is a random event.
Protein scores greater than 75 are significant (p<0.05).

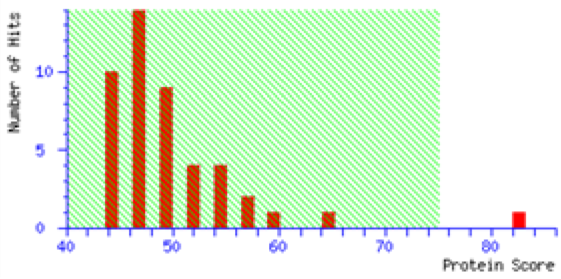

Matched peptide sequences: shown in Bold Red

1	**MASNLYPHRG**	**FMLDTGR**K**FF**	**PVK**AILHLLT	LLHQYNFNVF	HWHIYDAESF
51	PLLWPAGEGL	TNASVKYSQT	HTYYTPSDIQ	NVISYAENLG	ILVYPETDMP
101	GHSDIWGIWK	KDLVVGKASL	K**KPDAQLDIR**	QNNK**QVYDYI**	**R**SLVSTVDGY
151	FGSPYHHFGG	DEVAYMWNTR	DDNKLFNSFL	NWLKTLTPK**K**	**SVILWDDPLT**
201	**DSEKSITLSE**	**DWIIQTWHK**G	TTQKILKKGH	R**VIVSESDTF**	**YIGNADADKI**
251	**SSFVFPK**SSK	VLGFEVAWFT	SQDDDPSDLD	QDWIIDPLKA	ASKIRRK

Matched peptides information:

Start	–	End	Observed	Mr(expt)	Mr(calc)	ppm	M	Peptide
1	–	17	1966.0669	1965.0596	1964.9349	63.5	1	-.MASNLYPHRGFMLDTGR.K
10	–	17	896.5097	895.5025	895.4222	89.6	0	R.GFMLDTGR.K
10	–	17	912.4974	911.4901	911.4171	80.1	0	R.GFMLDTGR.K + Oxidation (M)
19	–	23	637.4001	636.3928	636.3635	46.0	0	K.FFPVK.A
122	–	130	1055.6778	1054.6705	1054.5771	88.6	0	K.KPDAQLDIR.Q
135	–	141	956.5820	955.5747	955.4763	103	0	K.QVYDYIR.S
190	–	204	1745.9789	1744.9716	1744.8883	47.7	1	K.KSVILWDDPLTDSEK.S
191	–	204	1617.9132	1616.9059	1616.7934	69.6	0	K.SVILWDDPLTDSEK.S
205	–	219	1857.0505	1856.0432	1855.9468	51.9	0	K.SITLSEDWIIQTWHK.G
232	–	249	1944.0272	1943.0199	1942.9160	53.5	0	R.VIVSESDTFYIGNADADK.I
232	–	257	2849.5086	2848.5013	2848.4171	29.6	1	R.VIVSESDTFYIGNADADKISSFVFPK.S
250	–	257	924.5977	923.5904	923.5117	85.2	0	K.ISSFVFPK.S

Spot No.: **99**

Mascot score: **83**

Species: ***Fusarium oxysporum f. sp. cubense race 4***

Protein name: **Glucose-6-phosphate 1-epimerase**

NCBI accession No.: **gi| 475666766**

Sequence coverage %: **46**

Matched peptides No.: **10**

Total peptides No.: **60**

Calculated Mr: **33685**

Calculated *p*I: **4.95**

Annotated PMF **spectra:**

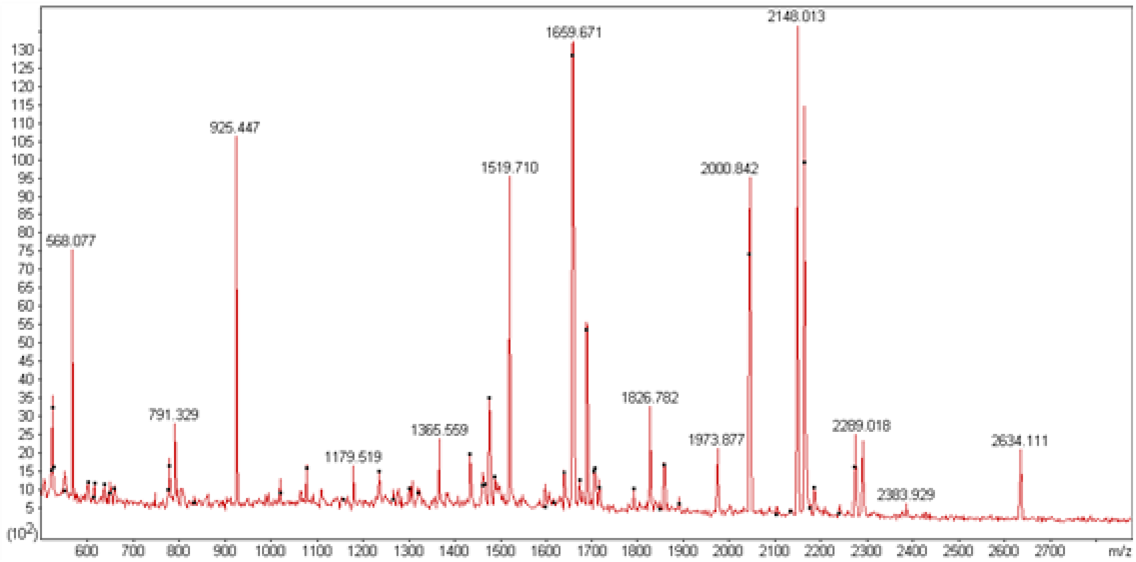

Probability Based Mowse Score:

Protein score is -10*Log(P), where P is the probability that the observed match is a random event.
Protein scores greater than 75 are significant (p<0.05).

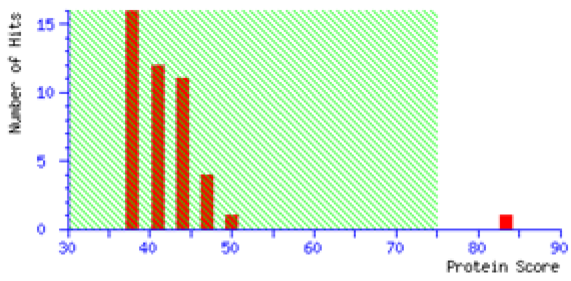

Matched peptide sequences: shown in Bold Red

1 MVDRPNKPSA LASTPAAPPQ PAVDITHDNS RVKAVLPTGE SVEVLLHGAT
51 VISWKDASGA EK**LWVSESAA LDGSAPVRGG IPIVFPVFGT APDHEPVAKL**
101 **PQHGFAR**NSR **WEFLGK**STSE GSSSSVK**LDF GLSSESISDD FKALWPYK**FA
151 LIYSVSLDPE SLNTTIVVTN DGDTAFDFQT LLHTYFKISD ISSAEVTGLE
201 DSVYFSK**VSS SEATQSGAIT FSAETDSVYT PAKGPSHPVV ISESGTPR**FR
251 IVR**DNLDQVV VWNPWVDK**SA GIKDFTPKDG WKNMLCVEPG SVKGWQK**LEK**
301 **GDAFEAAQTI TLV**

Matched peptides information:

Start	-	End	Observed	Mr(expt)	Mr(calc)	ppm	M	Peptide
63	-	78	1657.7545	1656.7472	1656.8471	-60.3	0	K.LWVSESAALDGSAPVR.G
79	-	99	2148.0129	2147.0056	2147.1416	-63.3	0	R.GGIPIVFPVFGTAPDHEPVAK.L
100	-	107	925.4469	924.4397	924.4930	-57.7	0	K.LPQHGFAR.N
111	-	116	779.3233	778.3160	778.4014	-110	0	R.WEFLGK.S
128	-	142	1659.6711	1658.6638	1658.7675	-62.5	0	K.LDFGLSSESISDDFK.A
143	-	148	777.3251	776.3178	776.4221	-134	0	K.ALWPYK.F
208	-	233	2634.1111	2633.1038	2633.2344	-49.6	0	K.VSSSEATQSGAITFSAETDSVYTPAK.G
234	-	248	1519.7096	1518.7023	1518.7791	-50.5	0	K.GPSHPVVISESGTPR.F
254	-	268	1826.7824	1825.7751	1825.8999	-68.3	0	R.DNLDQVVVWNPWVDK.S
298	-	313	1705.7509	1704.7436	1704.8934	-87.9	1	K.LEKGDAFEAAQTITLV.-
